# Supplementary material for: A theory-driven synthesis of symmetric and unsymmetric 1,2-bis(diphenylphosphino)ethane analogues via radical difunctionalization of ethylene
Source: Nat Commun. 2022 Nov 21;13:7034. doi: 10.1038/s41467-022-34546-5 (PMC9678890; doi:10.1038/s41467-022-34546-5)
Supplement: Supplementary file 1 — Supplementary Information [file 41467_2022_34546_MOESM1_ESM.pdf]

Supplementary Information

A theory-driven synthesis of symmetric and unsymmetric 1,2-bis(diphenylphosphino)ethane analogues via radical difunctionalization of ethylene

Hideaki Takano,[1,2] Hitomi Katsuyama,[1,2] Hiroki Hayashi,[1,2] Wataru Kanna,[3] Yu Harabuchi,[1,2,3] Satoshi Maeda,[*,1,2,3,4] and Tsuyoshi Mita[*,1,2]

[1] *Institute for Chemical Reaction Design and Discovery (WPI-ICReDD), Hokkaido University, Kita 21 Nishi 10, Kita-ku, Sapporo, Hokkaido 001-0021, Japan*

[2] *JST, ERATO Maeda Artificial Intelligence in Chemical Reaction Design and Discovery Project, Kita 10 Nishi 8, Kita-ku, Sapporo, Hokkaido 060-0810, Japan*

[3] *Department of Chemistry, Faculty of Science, Hokkaido University, Kita 10 Nishi 8, Kita-ku, Sapporo, Hokkaido 060-0810, Japan*

[4] *Research and Services Division of Materials Data and Integrated System (MaDIS), National Institute for Materials Science (NIMS), Tsukuba, Ibaraki 305-0044, Japan*

smaeda@eis.hokudai.ac.jp

tmita@icredd.hokudai.ac.jp

Table of Contents

Supplementary Methods

(A) General 3

(B) Reaction Apparatus for High-Pressure Photochemical Reaction 4

(C) Synthesis of phosphine oxide **1** 5

(D) Procedure for the Synthesis of DPPE from Diphosphine 5

(E) Procedure for the Synthesis of DPPE from Diphenylphosphine and Chlorodiphenylphosphine 5

(F) General Procedures for the Synthesis of DPPE Derivatives 6

(G) Synthesis of **4aa** and **4gb** 19

(H) Gram Scale Synthesis of **3gb** 20

(I) Transformation of Oxide Sulfides **3** to Dioxides **5** 21

(J) Synthesis of $PhSiH_2$-O-SiH_2Ph 24

(K) Reduction of Dioxides **5** 24

(L) Synthesis of Unsymmetric DPPE Oxide **7gb** and Its Reduction 27

(M) Synthesis of Transition-Metal Complexes 28

(N) NMR Studies 30

(O) Crystal structures 33

(P) Measurement of Photophysical Properties 35

Supplementary References 38

Supplementary Analytical Data

Copies of ^{1}H NMR, ^{13}C NMR, ^{19}F NMR, and ^{31}P NMR Spectra 39

Supplementary Methods

(A) General

All manipulations were carried out under an atmosphere of nitrogen unless otherwise noted. Infrared (IR) spectra were recorded on a JASCO FT/IR 4600 Fourier transform infrared spectrophotometer. NMR spectra were recorded on a JEOL ESZ-400S spectrometer, operating at 400 MHz (^{1}H), 100 MHz (^{13}C), 376 MHz (^{19}F), and 162 MHz (^{31}P). Chemical shifts in $CDCl_3$ were reported in the scale relative to $CHCl_3$ (7.26 ppm) or CH_2Cl_2 (5.32 ppm) for ^{1}H NMR and to $CDCl_3$ (77.16 ppm) or CD_2Cl_2 (53.84 ppm) for ^{13}C NMR as internal references. EI mass spectra were measured on a JEOL JMS-T100GCV and ESI mass spectra were measured on a Thermo Scientific Exactive. A cylinder of ethylene was purchased from Hokkaido Air Water, Inc. A 45W PR160L-440 nm (blue LED (440 nm), Kessil Lighting), A 160WE TUNA SUN (white LED, Kessil Lighting), TH2-140X105GY57 (yellow green LED (570 nm), CCS Inc.), and LED-PBULB/24WC (green LED (520 nm), yellow LED (600 nm), and red LED (630 nm), Life Partner Corp.) were used as the light source under photochemical conditions. HPG-10 (Taiatsu Techno Corporation) was used as an autoclave which consists of a polycarbonate cylinder. Emission spectra of monochromatic LEDs and white LED were measured by USB4000 (Ocean Photonics) and the strength of LEDs were measured by T-10MA (KONICA MINOLTA, Inc.). Optical resolution of **3jb** was performed by high performance liquid chromatography (HPLC) using JASCO Extrema HPLC systems with photo diode array detector MD-4010. Recycle preparative HPLC was performed using a JAI LaboACE LC-5060. All UV-Vis absorption spectra were measured on a V-770 (JASCO Corp.). Emission spectra and lifetime at 90 K were measured on a HORIBA Fluorolog-3 spectrofluorometer and corrected for the response of the detector system using a cryostat (SB1905HA, Thermal Block Company) and a temperature controller (Model 9700, Scientific Instruments). Emission lifetimes (τ_{obs}) were measured using a pulse diode light source SpectraLED with peak wavelength at 518 nm, 520 nm, 572 nm, and 620 nm recorded on a HORIBA Fluorolog-3 spectrofluorometer. Emission lifetimes were determined from the slope of logarithmic plots of the decay profiles. Emission decay curves were analysed by multi-exponential curve fittings. All starting materials except phophine oxides and hydrosilane ($PhSiH_2$-O-SiH_2Ph) are commercially available from TCI, Aldrich, Alfa Aesar and other suppliers, and used as received.

(B) Reaction Apparatus for High-Pressure Photochemical Reaction

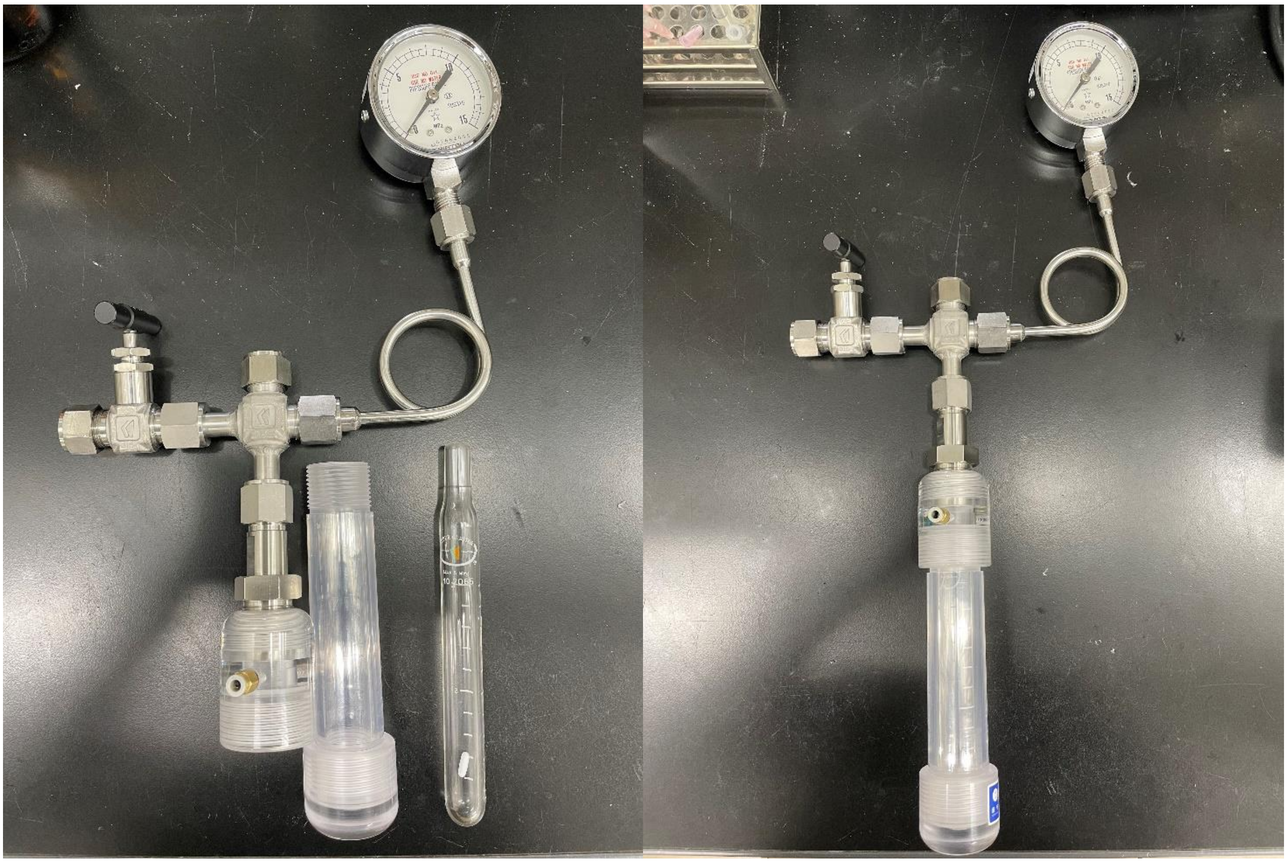

(a) **(b)**

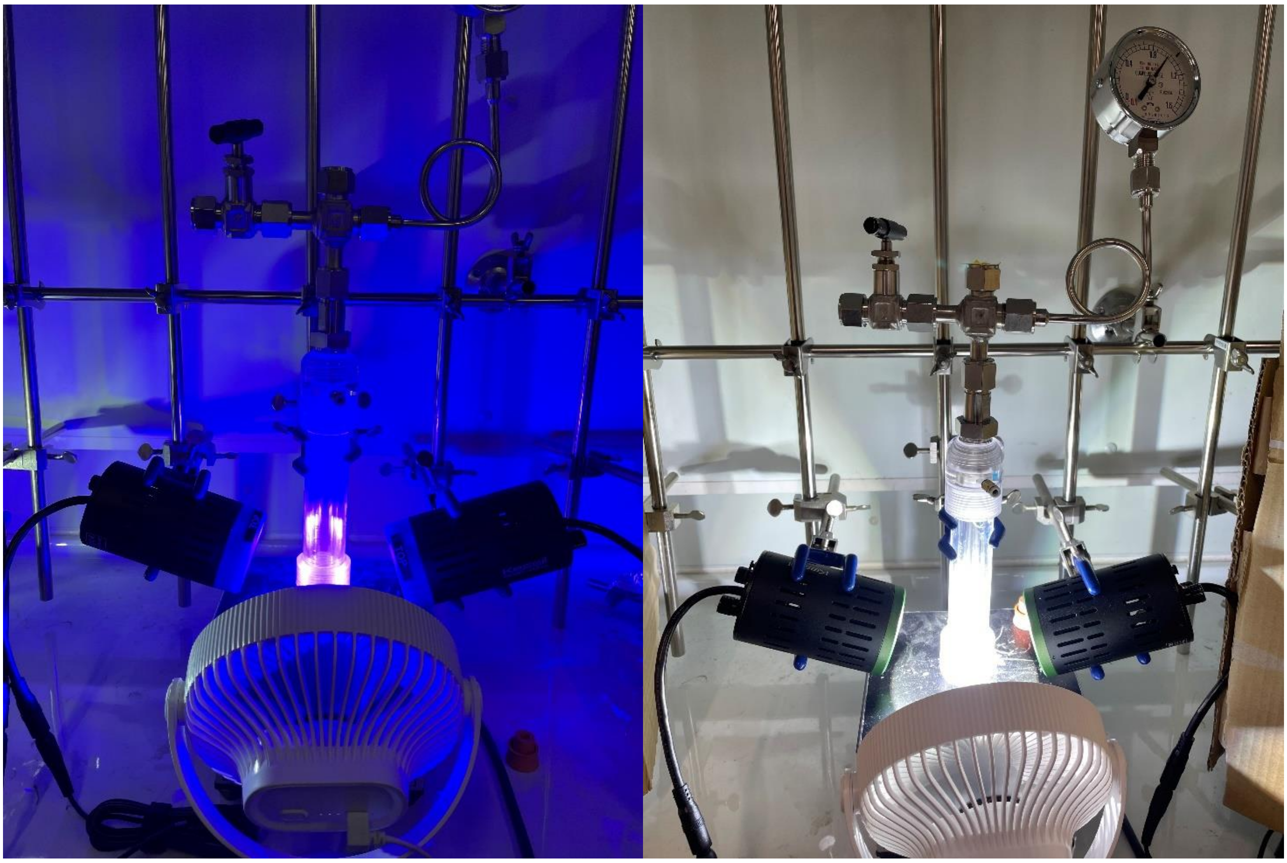

(c) **(d)**

Supplementary Figure 1. (a) Autoclave with a polycarbonate cylinder and a pressure-resistant glass tube. (b) The apparatus setting (c) A reaction under blue LED (d) A reaction under white LED

(C) Synthesis of phosphine oxide 1

Phosphine oxide **1** was synthesized according to the literature[1].

An oven dried two-neck flask with a reflux condenser was charged with magnesium turnings (72 mmol), I_2 (1 crystal) and THF (100 mL) under nitrogen atmosphere. Then aryl bromide (60 mmol) was added dropwise. If aryl bromide was solid, 50 mL solution of aryl bromide (60 mL) was added dropwise to 50 mL solution of magnesium turnings (72 mmol) and I_2 (1 crystal). After the bromide addition completed, the reaction mixture was stirred at reflux for 3 h. Next, the flask was placed in ice bath, diethyl phosphite (20 mmol) was added dropwise to the mixture via a syringe, and stirred for 4 h at room temperature. The reaction was quenched with 2 M aq. HCl (40 mL) at 0 °C, and was extracted with EtOAc three times and the combined organic layers were washed with brine, dried over Na_2SO_4 and filtered. After the solvent removed under vacuum, the resulting crude product was purified by recrystallization from the mixture of EtOAc and hexane, or silica-gel column chromatography to yield phosphine oxide **1**.

(D) Procedure for the Synthesis of DPPE from Diphosphine

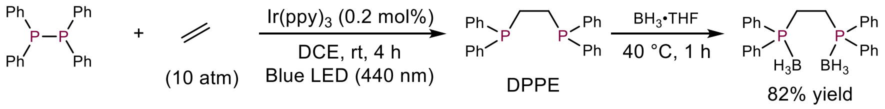

In an oven-dried 10 mL pressure-resistant glass tube were placed tetraphenyldiphosphine (185.2 mg, 0.5 mmol, 1.0 equiv) and Ir(ppy)$_3$ (0.7 mg, 0.001 mmol, 0.2 mol%) in glove box. After the addition of DCE (1.5 mL) under nitrogen, the tube was placed into autoclave which consists of a polycarbonate cylinder, and ethylene gas was pressurised to 10 atm. After the resulting mixture was stirred at room temperature for 4 h under the irradiation of blue LED (45W PR160L-440 nm Kessil light × 2), the solvent was evaporated to give the crude mixture. THF (1.0 mL) and 1M BH_3 in THF (4 mL, 4 mmol, 8 equiv) were added, and then, the reaction mixture was stirred for 1 h at 40 °C. After the evaporation, the crude product was purified by silica-gel column chromatography (eluent: hexane/ethyl acetate, 9/1 to 1/1) to afford the product (174.2 mg, 0.41mmol, 82%), of which spectra were consistent with a previous report[2].

(E) Procedure for the Synthesis of DPPE from Diphenylphosphine and Chlorodiphenylphosphine

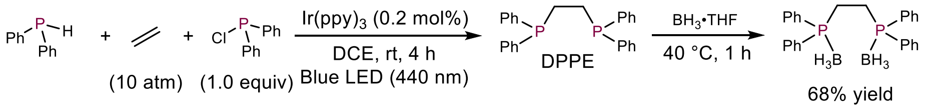

In an oven-dried 10 mL pressure-resistant glass tube were placed diphenylphosphine (86.2 µL, 0.5 mmol, 1.0 equiv) and Ir(ppy)$_3$ (0.7 mg, 0.001 mmol, 0.2 mol%) in glove box. After the addition of chlorodiphenylphosphine (91.9 µL, 0.5 mmol, 1.0 equiv) and DCE (1.5 mL) under nitrogen, the tube was placed into autoclave which consists of a polycarbonate cylinder, and ethylene gas was pressurised to 10 atm. After the resulting mixture was stirred at room temperature for 4 h under the irradiation of blue LED (45W PR160L-440 nm Kessil light × 2), the solvent was evaporated to give the crude mixture. THF (1.0 mL) and 1M BH_3 in THF (4 mL, 4 mmol, 8 equiv) were added, and then, the reaction mixture was stirred for 1 h at 40 °C. After the evaporation, the crude product was purified by silica-gel column chromatography (eluent: hexane/ethyl acetate, 9/1 to 1/1) to afford the product (145.6 mg, 0.34 mmol, 68%), of which spectra were consistent with a previous report[2].

(F) General Procedures for the Synthesis of DPPE Derivatives

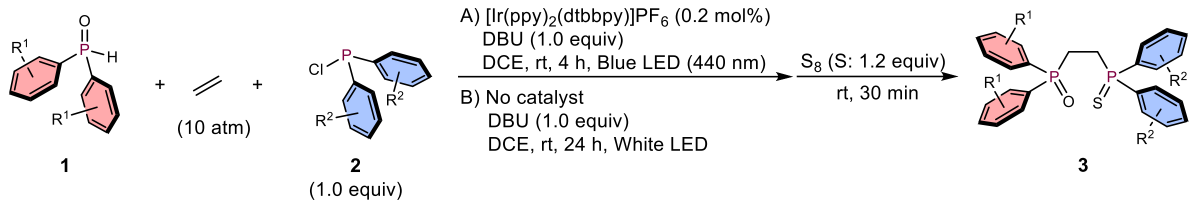

General Procedure A (for blue LED)

In an oven-dried 10 mL pressure-resistant glass tube were placed phosphine oxide **1** (0.5 mmol, 1.0 equiv) and [Ir(ppy)$_2$(dtbbpy)]PF$_6$ (0.9 mg, 0.001 mmol, 0.2 mol%). After the addition of DCE (1.5 mL) under nitrogen, chlorophosphine **2** (0.5 mmol, 1.0 equiv) and DBU (74.8 μL, 0.5 mmol, 1.0 equiv) were added. Then, the tube was placed into autoclave which consists of a polycarbonate cylinder, and ethylene gas was pressurised to 10 atm. After the resulting mixture was stirred at room temperature for 4 h under the irradiation of blue LED (45W PR160L-440 nm Kessil light × 2), sulfur (19.2 mg, 0.6 mmol, 1.2 equiv of S) was added into the reaction mixture. After the mixture was stirred for 30 min, the solvent was evaporated to give the crude mixture. The crude product was purified by silica-gel column chromatography to afford the product **3**.

General Procedure B (for white LED)

In an oven-dried 10 mL pressure-resistant glass tube was placed phosphine oxide **1** (0.5 mmol, 1.0 equiv). After the addition of DCE (1.5 mL) under nitrogen, chlorophosphine **2** (0.5 mmol, 1.0 equiv) and DBU (74.8 μL, 0.5 mmol, 1.0 equiv) were added. Then, the tube was placed into autoclave which consists of a polycarbonate cylinder, and ethylene gas was pressurised to 10 atm. After the resulting mixture was stirred at room temperature for 24 h under the irradiation of white LED (A 160WE TUNA SUN × 2), sulfur (19.2 mg, 0.6 mmol, 1.2 equiv of S) was added into the reaction mixture. After the mixture was stirred for 30 min, the solvent was evaporated to give the crude mixture. The crude product was purified by silica-gel column chromatography to afford the product **3**.

(2-(Diphenylphosphorothioyl)ethyl)diphenylphosphine oxide (**3aa**)

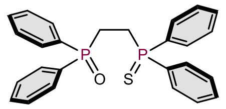

Diphenylphosphine oxide (**1a**; 101.1 mg, 0.5 mmol, 1.0 equiv) and chlorodiphenylphosphine (**2a**; 91.9 μL, 0.5 mmol, 1.0 equiv) were employed as starting materials. Purification of the crude product by silica-gel column chromatography (eluent: DCM/acetone, 9/1) afforded **3aa** (procedure A: 182.2 mg, 0.41 mmol, 82% yield, procedure B: 165.7 mg, 0.37 mmol, 74% yield). White solid; IR (ATR): 3051, 1592, 1437, 1255, 1184, 1173, 1120, 1101, 1026, 793, 730, 689, 610 cm^{-1}; ^{1}H NMR (400 MHz, CDCl$_3$) δ: 7.81-7.75 (m, 4H), 7.73-7.68 (m, 4H), 7.52-7.40 (m, 12H), 2.74-2.66 (m, 2H), 2.57-2.49 (m, 2H) ppm; ^{13}C NMR (100 MHz, CDCl$_3$) δ: 132.0 (d, J = 2.4 Hz), 131.7 (d, J = 99.1 Hz), 131.7 (d, J = 2.9 Hz), 131.5 (d, J = 80.3 Hz), 130.9 (d, J = 10.2 Hz), 130.6 (d, J = 9.2 Hz), 128.7 (d, J = 11.6 Hz), 128.7 (d, J = 12.1 Hz), 24.6 (d, J = 55.9 Hz), 22.4 (d, J = 69.6 Hz) ppm; ^{31}P NMR (162 MHz, CDCl$_3$) δ: 45.4 (d, J = 58.5 Hz), 33.6 (d, J = 58.5 Hz) ppm; HRMS (ESI) m/z calcd. for $C_{26}H_{24}OP_2SNa^+$ [M+Na]$^+$: 469.0915, found: 469.0904.

(2-(Bis(4-trifluoromethylphenyl)phosphorothioyl)ethyl)bis(4-trifluoromethylphenyl)phosphine oxide (**3bb**)

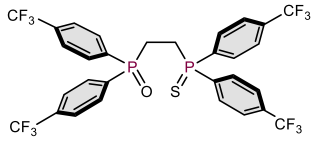

Bis(4-trifluoromethylphenyl)phosphine oxide (**1b**; 169.1 mg, 0.5 mmol, 1.0 equiv) and chlorobis(4-trifluoromethylphenyl)phosphine (**2b**; 125.6 μL, 0.5 mmol, 1.0 equiv) were employed as starting materials. Purification of the crude product by silica-gel column chromatography (eluent: hexane/ethyl acetate, 9/1 to 1/1) afforded **3bb** (procedure A: 208.1 mg, 0.29 mmol, 58% yield, procedure B: 264.6 mg, 0.37 mmol, 74% yield). White solid; IR (ATR): 2922, 1400, 1323, 1164, 1123, 1104, 1061, 1015, 835, 729, 709, 640 cm^{-1}; ^{1}H NMR (400 MHz, $CDCl_3$) δ: 7.97-7.86 (m, 8H), 7.77-7.71 (m, 8H), 2.80-2.71 (m, 2H), 2.64-2.55 (m, 2H) ppm; ^{13}C NMR (100 MHz, $CDCl_3$) δ: 135.9-133.7 (m, 4C), 131.6 (d, J = 10.8 Hz), 131.3 (d, J = 9.8 Hz), 126.2-125.9 (m, 2C), 123.3 (q, J = 271.4 Hz, two CF_3 groups were totally overlapped), 24.4 (d, J = 55.9 Hz), 22.2 (d, J = 70.6 Hz) ppm; ^{19}F NMR (376 MHz, $CDCl_3$) δ: −63.3, −63.3 ppm; ^{31}P NMR (162 MHz, $CDCl_3$) δ: 44.4 (d, J = 58.2 Hz), 31.2 (d, J = 58.2 Hz) ppm; HRMS (ESI) m/z calcd. for $C_{30}H_{20}F_{12}OP_2SNa^+$ $[M+Na]^+$: 741.0411, found: 741.0390.

(2-(Bis(4-chlorophenyl)phosphorothioyl)ethyl)bis(4-chlorophenyl)phosphine oxide (**3cc**)

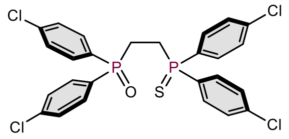

Bis(4-chlorophenyl)phosphine oxide (**1c**; 135.5 mg, 0.5 mmol, 1.0 equiv) and chlorobis(4-chlorophenyl)phosphine (**2c**; 144.8 mg, 0.5 mmol, 1.0 equiv) were employed as starting materials. Purification of the crude product by silica-gel column chromatography (eluent: hexane/ethyl acetate, 1/1) afforded **3cc** (procedure A: 192.8 mg, 0.33 mmol, 66% yield, procedure B: 224.1 mg, 0.38 mmol, 77% yield). White solid; IR (ATR): 2920, 1580, 1480, 1389, 1196, 1175, 1085, 1012, 819, 747, 729, 642 cm^{-1}; ^{1}H NMR (400 MHz, $CDCl_3$) δ: 7.73-7.60 (m, 8H), 7.46-7.41 (m, 8H), 2.67-2.58 (m, 2H), 2.50-2.41 (m, 2H) ppm; ^{13}C NMR (100 MHz, $CDCl_3$) δ: 139.2 (d, J = 3.3 Hz), 139.0 (d, J = 3.4 Hz), 132.5 (d, J = 11.4 Hz), 132.2 (d, J = 10.1 Hz), 130.4-129.3 (m, for 4C), 24.7 (d, J = 56.4 Hz), 22.5 (d, J = 70.5 Hz) ppm; ^{31}P NMR (162 MHz, $CDCl_3$) δ: 44.3 (d, J = 58.5 Hz), 32.4 (d, J = 58.5 Hz) ppm; HRMS (ESI) m/z calcd. for $C_{26}H_{20}Cl_4OP_2SNa^+$ $[M+Na]^+$: 604.9356, found: 604.9350.

(2-(Di(furan-2-yl)phosphorothioyl)ethyl)di(furan-2-yl)phosphine oxide (**3dd**)

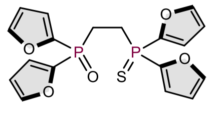

Di(furan-2-yl)phosphine oxide (**1d**; 45.5 mg, 0.25 mmol, 1.0 equiv) and chlorodi(furan-2-yl)phosphine (**2d**; 39.0 μL, 0.25 mmol, 1.0 equiv) were employed as starting materials. Purification of the crude product by silica-gel column chromatography (eluent:DCM/acetone, 20/1 to 7/1) afforded **3dd** (procedure A: 30.8 mg, 0.076 mmol, 30% yield, procedure B: 16.9 mg, 0.042 mmol, 17% yield). White solid; IR (ATR): 3090, 1553, 1458, 1368, 1206, 1125, 1002, 909, 744, 707, 655, 634 cm^{-1}; ^{1}H NMR (400 MHz, $CDCl_3$) δ: 7.69-7.66 (m, 4H), 7.18-7.16 (m, 2H), 7.14-7.13 (m, 2H), 6.52-6.48 (m, 4H), 2.76-2.67 (m, 2H), 2.57-2.47 (m, 2H) ppm; ^{13}C NMR (100 MHz, $CDCl_3$) δ: 148.9 (d, J = 7.1 Hz), 148.7 (d, J = 8.0 Hz), 146.2 (d, J = 126.8 Hz), 146.1 (d, J = 133.6 Hz), 123.2 (d, J = 20.8 Hz), 122.8 (d, J = 19.4 Hz), 111.4 (d, J = 9.1 Hz), 111.2 (J = 8.7 Hz), 24.5 (dd, J = 62.6, 2.4 Hz), 22.2 (dd, J = 79.6, 1.8 Hz) ppm; ^{31}P NMR (162 MHz, $CDCl_3$) δ: 17.1 (d, J = 71.6 Hz), 12.2 (d, J = 71.6 Hz) ppm; HRMS (ESI) m/z calcd. for $C_{18}H_{16}O_5P_2SNa^+$ $[M+Na]^+$: 429.0086, found: 429.0076.

(2-(Bis(3,5-di-*tert*-butyl-4-methoxyphenyl)phosphorothioyl)ethyl)bis(3,5-di-*tert*-butyl-4-methoxyphenyl)phosphine oxide (**3ee**)

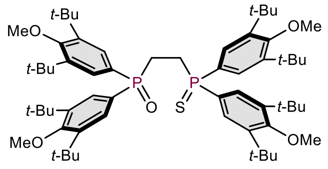

Bis(3,5-di-*tert*-butyl-4-methoxyphenyl)phosphine oxide (**1e**; 121.7 mg, 0.25 mmol, 1.0 equiv) and chlorobis(3,5-di-*tert*-butyl-4-methoxyphenyl)phosphine (**2e**; 126.3 mg, 0.25 mmol, 1.0 equiv) were employed as starting materials. Purification of the crude product by silica-gel column chromatography (eluent: hexane/ethyl acetate, 4/1) afforded **3ee** (procedure A: 20.3 mg, 0.020 mmol, 8% yield, procedure B: 37.2 mg, 0.037 mmol, 15% yield). White solid; IR (ATR): 2963, 1409, 1394, 1227, 1213, 1169, 1146, 1117, 1008, 733 cm^{-1}; ^1H NMR (400 MHz, $CDCl_3$) δ: 7.63 (d, J = 13.5 Hz, 4H), 7.52 (d, J = 12.2 Hz, 4H), 3.68 (s, 12H), 2.63-2.53 (m, 4H), 1.38 (s, 36H), 1.37 (s, 36H) ppm; ^{13}C NMR (100 MHz, $CDCl_3$) δ: 162.9 (d, J = 3.3 Hz), 162.8 (d, J = 3.3 Hz), 144.5 (d, J = 11.9 Hz), 144.3 (d, J = 12.1 Hz), 130.0 (d, J = 11.9 Hz), 129.7 (d, J = 10.9 Hz), 125.8 (d, J = 102.8 Hz), 125.4 (d, J = 83.4 Hz), 64.6, 64.6, 36.2, 36.1, 32.0, 32.0, 26.6 (dd, J = 54.6, 2.5 Hz), 23.7 (dd, J = 69.7, 2.3 Hz) ppm; ^{31}P NMR (162 MHz, $CDCl_3$) 46.7 (d, J = 56.9 Hz), 36.7 (d, J = 56.9 Hz) ppm; HRMS (ESI) *m/z* calcd. for $C_{62}H_{96}O_5P_2SNa^+$ $[M+Na]^+$: 1037.6346, found: 1037.6317.

(2-(Bis(4-methoxyphenyl)phosphorothioyl)ethyl)bis(4-methoxyphenyl)phosphine oxide (**3ff**)

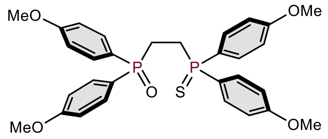

Bis(4-methoxyphenyl)phosphine oxide (**1f**; 131.1 mg, 0.5 mmol, 1.0 equiv) and chlorobis(4-methoxyphenyl)phosphine (**2f**; 140.3 mg, 0.5 mmol, 1.0 equiv) were employed as starting materials. Purification of the crude product by silica-gel column chromatography (eluent: DCM/acetone, 9/1 to 4/1) afforded **3ff** (procedure A: 102.8 mg, 0.18 mmol, 36% yield). White solid; IR (ATR): 2930, 1592, 1498, 1293, 1252, 1167, 1101, 1022, 799, 742, 665, 628 cm^{-1}; ^1H NMR (400 MHz, $CDCl_3$) δ: 7.72-7.67 (m, 4H), 7.62-7.57 (m, 4H), 6.94-6.90 (m, 8H), 3.81-3.80 (m, 12H), 2,66-2.57 (m, 2H), 2.48-2.39 (m, 2H) ppm; ^{13}C NMR (100 MHz, $CDCl_3$) δ: 162.5 (d, J = 2.9 Hz), 162.3 (d, J = 2.9 Hz), 133.0 (d, J = 11.7 Hz), 132.7 (d, J = 10.8 Hz), 123.4 (d, J = 105.7 Hz), 123.1 (d, J = 86.7 Hz), 114.5-114.3 (m, 2C), 55.5, 55.4, 25.5 (d, J = 56.6 Hz), 23.1 (d, J = 70.9 Hz) ppm; ^{31}P NMR (162 MHz, $CDCl_3$) δ: 44.0 (d, J = 58.5 Hz), 34.2 (d, J = 58.5 Hz) ppm; HRMS (ESI) *m/z* calcd. for $C_{30}H_{32}O_5P_2SNa^+$ $[M+Na]^+$: 589.1338, found: 589.1320.

(2-(Bis(4-trifluoromethylphenyl)phosphorothioyl)ethyl)bis(4-dimethylaminophenyl)phosphine oxide (**3gb**)

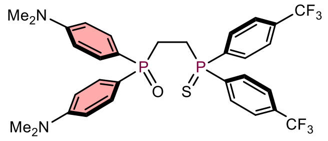

Bis(4-dimethylaminophenyl)phosphine oxide (**1g**; 144.2 mg, 0.5 mmol, 1.0 equiv) and chlorobis(4-trifluoromethylphenyl)phosphine (**2b**; 125.6 µL, 0.5 mmol, 1.0 equiv) were employed as starting materials. Purification of the crude product by silica-gel column chromatography (eluent: DCM/acetone, 9/1 to 4/1) afforded **3gb** (procedure A: 209.8 mg, 0.32 mmol, 63% yield, procedure B: 254.2 mg, 0.41 mmol, 82% yield). White solid; IR (ATR): 2912, 1598, 1515, 1361, 1315, 1165, 1114, 1061, 1016, 815, 734, 704 cm^{-1}; ^1H NMR (400 MHz, $CDCl_3$) δ: 7.93 (dd, J = 12.4, 8.2 Hz, 4H), 7.69 (dd, J = 8.2, 1.9 Hz, 4H), 7.49 (dd, J = 10.8, 8.8 Hz, 4H), 6.72-6.69 (m, 4H), 2.99 (s, 12H), 2.82-2.73 (m, 2H), 2.42-2.33 (m, 2H) ppm; ^{13}C NMR (100 MHz, $CDCl_3$) δ: 152.5 (d, J = 2.4 Hz), 136.0 (d, J = 78.0 Hz), 133.8 (qd, J = 33.0, 3.0 Hz), 132.3 (d, J = 10.6 Hz), 131.7 (d, J = 10.6 Hz), 125.9-125.8 (m), 123.5 (q, J = 271.4 Hz), 116.9 (d, J = 111.6 Hz), 111.6 (d, J = 12.5 Hz), 40.1, 24.9 (d, J = 55.9 Hz), 24.9 (dd, J = 69.9, 3.3 Hz) ppm; ^{19}F NMR (376 MHz, $CDCl_3$) δ: −63.1 ppm;

^{31}P NMR (162 MHz, $CDCl_3$) δ: 44.8 (d, J = 57.5 Hz), 34.4 (d, J = 57.5 Hz) ppm; HRMS (ESI) m/z calcd. for $C_{32}H_{32}F_6N_2OP_2SNa^+$ $[M+Na]^+$: 691.1507, found: 691.1480.

(2-(Bis(4-trifluoromethylphenyl)phosphorothioyl)ethyl)bis(4-methoxyphenyl)phosphine oxide (**3fb**)

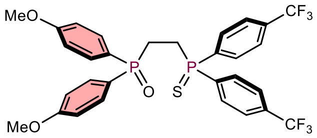

Bis(4-methoxyphenyl)phosphine oxide (**1f**; 131.1 mg, 0.5 mmol, 1.0 equiv) and chlorobis(4-trifluoromethylphenyl)phosphine (**2b**; 125.6 µL, 0.5 mmol, 1.0 equiv) were employed as starting materials. Purification of the crude product by silica-gel column chromatography (eluent: DCM/acetone, 9/1 to 7/3) afforded **3fb** (procedure A: 203.2 mg, 0.32 mmol, 63% yield, procedure B: 160.7 mg, 0.25 mmol, 50% yield). White solid; IR (ATR): 2942, 1597, 1504, 1397, 1315, 1256, 1167, 1118, 1061, 1015, 833, 741 cm^{-1}; ^{1}H NMR (400 MHz, $CDCl_3$) δ: 7.93 (dd, J = 12.6, 8.2 Hz, 4H), 7.70 (dd, J = 8.2, 2.3 Hz, 4H), 7.60 (dd, J = 11.2, 8.9 Hz, 4H), 6.95 (dd, J = 8.9, 2.3 Hz, 4H), 3.81 (s, 6H), 2.79-2.70 (m, 2H), 2.47-2.38 (m, 2H) ppm; ^{13}C NMR (100 MHz, $CDCl_3$) δ: 162.7 (d, J = 2.9 Hz), 135.8 (d, J = 78.5 Hz), 134.0 (qd, J = 32.8, 2.9 Hz), 132.7 (d, J = 10.7 Hz), 131.7 (d, J = 10.6 Hz), 126.0-125.8 (m), 123.4 (q, J = 271.5 Hz), 123.0 (d, J = 107.0 Hz), 114.6 (d, J = 12.9 Hz), 55.5, 24.7 (dd, J = 56.3, 2.8 Hz), 22.9 (dd, J = 70.1, 3.0 Hz) ppm; ^{19}F NMR (376 MHz, $CDCl_3$) δ: −63.1 ppm; ^{31}P NMR (162 MHz, $CDCl_3$) δ: 44.6 (d, J = 58.2 Hz), 33.6 (d, J = 58.2 Hz) ppm; HRMS (ESI) m/z calcd. for $C_{30}H_{26}F_6O_3P_2SNa^+$ $[M+Na]^+$: 665.0874, found: 665.0853.

(2-(Bis(4-trifluoromethylphenyl)phosphorothioyl)ethyl)bis(3-methoxyphenyl)phosphine oxide (**3hb**)

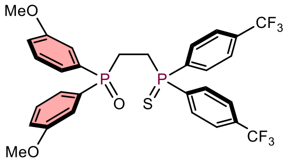

Bis(3-methoxyphenyl)phosphine oxide (**1h**; 131.1 mg, 0.5 mmol, 1.0 equiv) and chlorobis(4-trifluoromethylphenyl)phosphine (**2b**; 125.6 µL, 0.5 mmol, 1.0 equiv) were employed as starting materials. Purification of the crude product by silica-gel column chromatography (eluent: hexane/ethyl acetate, 1/1) afforded **3hb** (procedure A: 204.4 mg, 0.32 mmol, 64% yield, procedure B: 196.6 mg, 0.31 mmol, 61% yield). White solid; IR (ATR): 2937, 1575, 1419, 1315, 1167, 1129, 1062, 1045, 1016, 836, 739, 691 cm^{-1}; ^{1}H NMR (400 MHz, $CDCl_3$) δ: 7.94 (dd, J = 12.6, 8.0 Hz, 4H), 7.68 (d, J = 8.0 Hz, 4H), 7.37-7.32 (m, 2H), 7.28-7.20 (m, 4H), 7.03-7.00 (m, 2H), 3.77 (s, 6H), 2.84-2.75 (m, 2H), 2.54-2.45 (m, 2H) ppm; ^{13}C NMR (100 MHz, $CDCl_3$) δ: 159.7 (d, J = 14.6 Hz), 135.5 (d, J = 78.5 Hz), 134.2-132.2 (m, 2C), 131.6 (d, J = 10.7 Hz), 130.2 (d, J = 14.0 Hz), 125.8-125.6 (m), 123.3 (q, J = 271.6 Hz), 122.6 (d, J = 9.5 Hz), 118.1, 115.7 (d, J = 10.3 Hz), 55.3, 24.4 (d, J = 56.2 Hz), 22.3 (d, J = 69.5 Hz) ppm; ^{19}F NMR (376 MHz, $CDCl_3$) δ: −63.1 ppm; ^{31}P NMR (162 MHz, $CDCl_3$) δ: 44.6 (d, J = 58.5 Hz), 33.9 (d, J = 58.5 Hz) ppm; HRMS (ESI) m/z calcd. for $C_{30}H_{26}F_6O_3P_2SNa^+$ $[M+Na]^+$: 665.0874, found: 665.0845.

(2-(Bis(4-trifluoromethylphenyl)phosphorothioyl)ethyl)bis(2-methoxyphenyl)phosphine oxide (**3ib**)

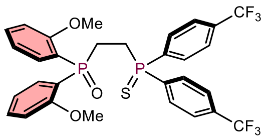

Bis(2-methoxyphenyl)phosphine oxide (**1i**; 131.1 mg, 0.5 mmol, 1.0 equiv) and chlorobis(4-trifluoromethylphenyl)phosphine (**2b**; 125.6 µL, 0.5 mmol, 1.0 equiv) were employed as starting materials. Purification of the crude product by silica-gel column chromatography (eluent: hexane/ethyl acetate, 1/1) afforded **3ib** (procedure A: 198.2 mg, 0.31 mmol, 62% yield, procedure B: 235.8 mg, 0.37 mmol, 72% yield). White solid; IR (ATR): 2952, 1591, 1476, 1433, 1399, 1317, 1180, 1121, 1061, 1015, 738, 704 cm^{-1}; ^{1}H NMR (400 MHz, $CDCl_3$) δ: 7.94 (dd, J = 12.3, 8.2 Hz, 4H), 7.69 (dd, J = 8.2, 2.3 Hz, 4H), 7.60-7.54 (m, 2H), 7.51-7.46 (m, 2H), 7.03-6.98 (m,

2H), 6.90-6.86 (m, 2H), 3.64 (s, 6H), 2.85-2.66 (m, 4H) ppm; ^{13}C NMR (100 MHz, $CDCl_3$) *δ*: 160.5 (d, *J* = 3.6 Hz), 136.1 (d, *J* = 78.0 Hz), 134.1-133.0 (m, 3C), 131.6 (d, *J* = 10.6 Hz), 125.7-125.4 (m), 123.3 (q, *J* = 271.5 Hz), 120.7 (d, *J* = 11.6 Hz), 119.2 (d, *J* = 101.5 Hz), 110.9 (d, *J* = 6.7 Hz), 55.3, 25.0 (d, *J* = 56.4 Hz), 22.0 (d, *J* = 72.8 Hz) ppm; ^{19}F NMR (376 MHz, $CDCl_3$) *δ*: −63.1 ppm; ^{31}P NMR (162 MHz, $CDCl_3$) *δ*: 45.1 (d, *J* = 67.2 Hz), 34.1 (d, *J* = 67.2 Hz) ppm; HRMS (ESI) *m*/*z* calcd. for $C_{30}H_{26}F_6O_3P_2SNa^+$ $[M+Na]^+$: 665.0874, found: 665.0845.

(2-(Bis(4-trifluoromethylphenyl)phosphorothioyl)ethyl)(2-methoxylphenyl)phenylphosphine oxide (**3jb**)

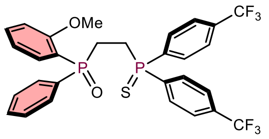

(2-Methoxyphenyl)phenylphosphine oxide (**1j**; 116.1 mg, 0.5 mmol, 1.0 equiv) and chlorobis(4-trifluoromethylphenyl)phosphine (**2b**; 125.6 μL, 0.5 mmol, 1.0 equiv) were employed as starting materials. Purification of the crude product by silica-gel column chromatography (eluent: hexane/ethyl acetate, 1/1) afforded **3jb** (procedure A: 231.0 mg, 0.38 mmol, 75% yield, procedure B: 238.4 mg, 0.39 mmol, 78% yield). White solid; IR (ATR): 3064, 1590, 1477, 1397, 1320, 1166, 1124, 1060, 1014, 835, 799, 706 cm^{-1}; ^{1}H NMR (400 MHz, $CDCl_3$) *δ*: 7.98-7.90 (m, 5H), 7.76-7.66 (m, 6H), 7.52-7.37 (m, 4H), 7.10-7.07 (m, 1H), 6.88-6.84 (m, 1H), 3.71 (s, 3H), 2.92-2.73 (m, 2H), 2.66-2.53 (m, 2H) ppm; ^{13}C NMR (100 MHz, $CDCl_3$) *δ*: 159.7 (d, *J* = 4.6 Hz), 136.2 (d, *J* = 16.2 Hz), 135.5 (d, *J* = 15.9 Hz), 134.5-133.1 (m, 3C), 132.0-131.6 (m, 3C), 130.6 (d, *J* = 10.0 Hz), 128.5 (d, *J* = 12.0 Hz), 125.8-125.6 (m), 122.4 (q, *J* = 271.4 Hz), 121.3 (d, *J* = 10.9 Hz), 118.7 (d, *J* = 98.6 Hz), 55.3, 24.7 (d, *J* = 56.4 Hz), 22.0 (d, *J* = 70.8 Hz) ppm; ^{19}F NMR (376 MHz, $CDCl_3$) *δ*: −63.1 ppm; ^{31}P NMR (162 MHz, $CDCl_3$) *δ*: 44.8 (d, *J* = 62.4 Hz), 33.0 (d, *J* = 62.4 Hz) ppm; HRMS (ESI) *m*/*z* calcd. for $C_{29}H_{24}F_6O_2P_2SNa^+$ $[M+Na]^+$: 635.0769, found: 635.0742. Optical resolution was conducted by HPLC analysis using a chiral column (Daicel Chiralpak IA: 4.6×250 mm, 254 nm PDA detector, 30 °C, eluent: 20% 2-propanol in hexane, flow rate: 1 mL/min, retention time: 9.31 min and 11.7 min). Both enantiomers in the racemic mixture were separable by recycle preparative HPLC with a chiral column (Daicel Chiralpak IA: 20.0×250 mm, eluent: 20% 2-propanol in hexane, flow rate: 19 mL/min) to obtain enantioenriched **3jb**.

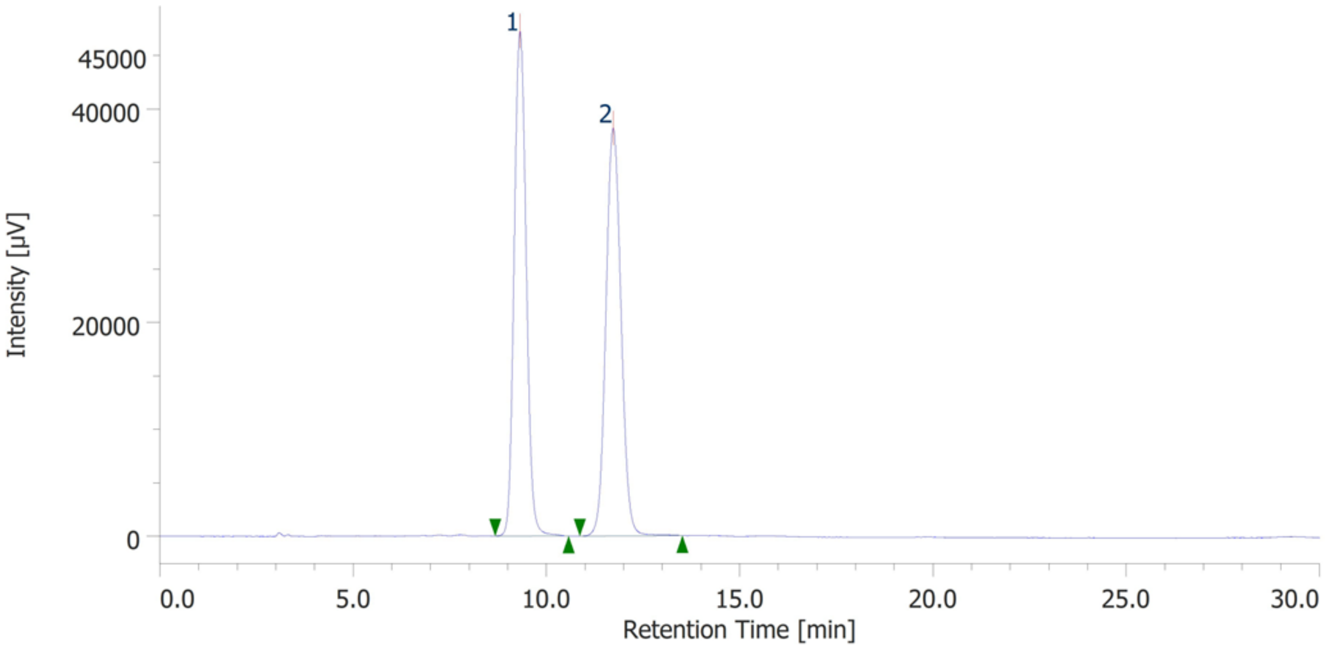

#	Peak Name	CH	tR [min]	Area [μV·sec]	Height [μV]	Area%	Height%	Quantity	NTP	Resolution	Symmetry Factor	Warning
1	Unknown	9	9.313	1025655	47220	49.711	55.313	N/A	4196	3.741	1.096	
2	Unknown	9	11.723	1037568	38148	50.289	44.687	N/A	4279	N/A	1.073	

Supplementary Figure 2. HPLC data of *rac*-**3jb**

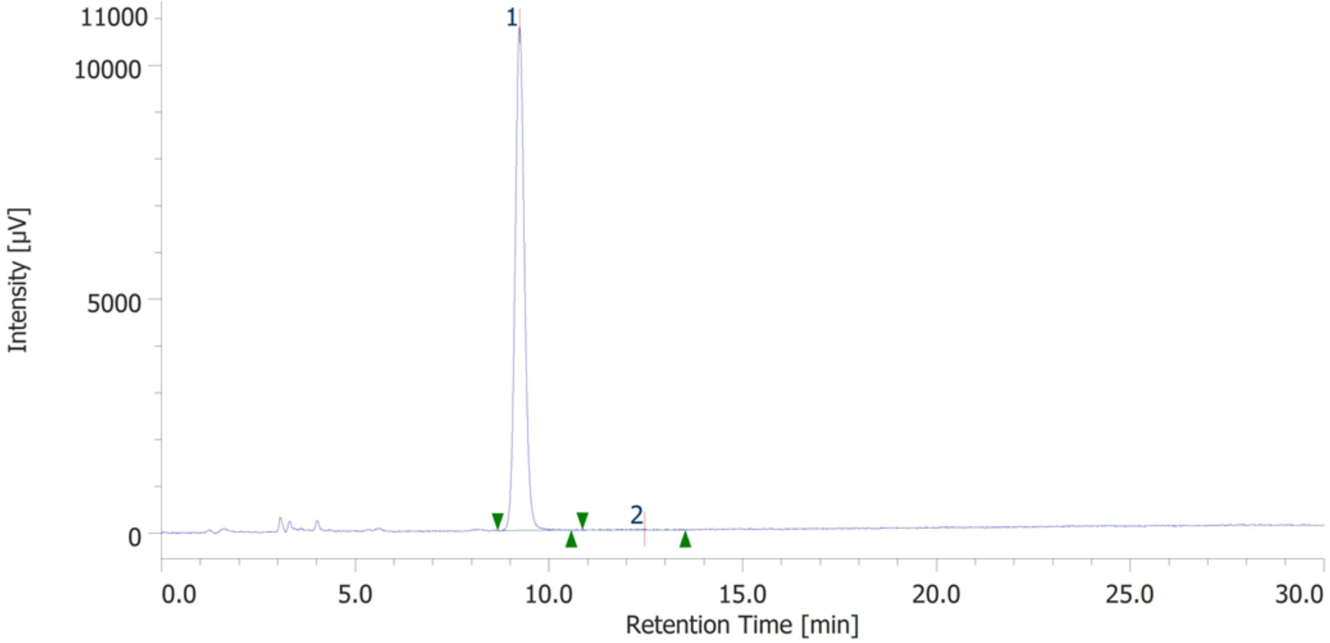

#	Peak Name	CH	tR [min]	Area [μV·sec]	Height [μV]	Area%	Height%	Quantity	NTP	Resolution	Symmetry Factor	Warning
1	Unknown	9	9.237	180888	10773	99.677	99.857	N/A	7217	1.589	1.098	
2	Unknown	9	12.460	586	15	0.323	0.143	N/A	188	N/A	0.832	

Supplementary Figure 3. HPLC data of **3jb** after the separation by recycle preparative HPLC (1st peak)

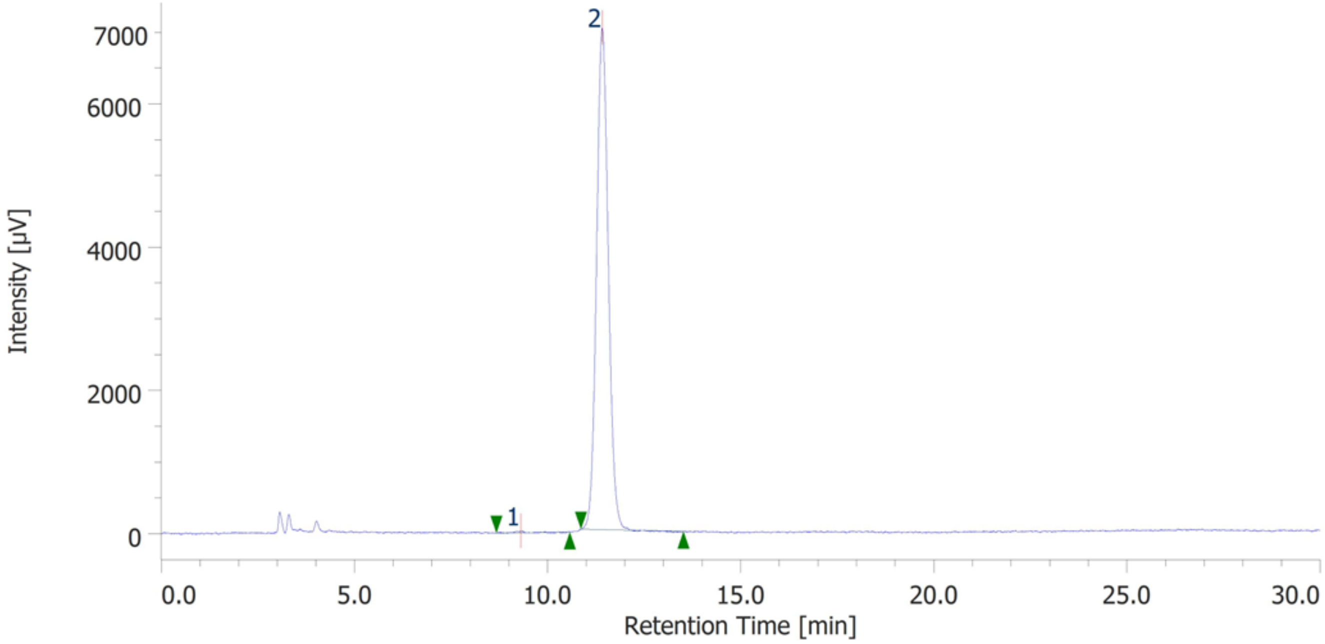

#	Peak Name	CH	tR [min]	Area [μV·sec]	Height [μV]	Area%	Height%	Quantity	NTP	Resolution	Symmetry Factor	Warning
1	Unknown	9	9.307	493	26	0.332	0.370	N/A	1191	2.588	1.504	
2	Unknown	9	11.410	147971	6999	99.668	99.630	N/A	6855	N/A	1.088	

Supplementary Figure 4. HPLC data of **3jb** after the separation by recycle preparative HPLC (2nd peak)

(2-(Bis(4-trifluoromethylphenyl)phosphorothioyl)ethyl)bis(4-*tert*-butylphenyl)phosphine oxide (**3kb**)

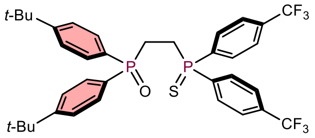

Bis(4-*tert*-butylphenyl)phosphine oxide (**1k**; 157.2 mg, 0.5 mmol, 1.0 equiv) and chlorobis(4-trifluoromethylphenyl)phosphine (**2b**; 125.6 μL, 0.5 mmol, 1.0 equiv) were employed as starting materials. Purification of the crude product by silica-gel column chromatography (eluent: hexane/ethyl acetate, 9/1 to 1/4) afforded **3kb** (procedure A: 184.9 mg, 0.27 mmol, 53% yield, procedure B: 128.6 mg, 0.19 mmol, 37% yield). White solid; IR (ATR): 2966, 1601, 1398, 1316, 1169, 1123, 1092, 1061, 1015, 831, 757, 704 cm^{-1}; ^{1}H NMR (400 MHz, $CDCl_3$) δ: 7.94 (dd, J = 12.5, 8.1 Hz, 4H), 7.70 (dd, J = 8.1, 2.2 Hz, 4H), 7.63 (dd, J = 11.3, 8.5 Hz, 4H), 7.47 (dd, J = 8.5 Hz, 2.7 Hz, 4H), 2.83-2.75 (m, 2H), 2.51-2.42 (m, 2H), 1.30 (s, 18H) ppm; ^{13}C NMR (100 MHz, $CDCl_3$) δ: 155.8 (d, J = 2.6 Hz), 135.8 (d, J = 78.4 Hz), 134.0 (qd, J = 33.0, 3.0 Hz), 131.7 (d, J = 10.7 Hz), 130.7 (d, J = 9.6 Hz), 128.6 (d, J = 102.6 Hz), 126.1-119.4 (m, 3C), 35.1, 31.2, 24.6 (dd, J = 56.1, 1.7 Hz), 22.7 (dd, J = 69.3, 2.1 Hz) ppm; ^{19}F NMR (376 MHz, $CDCl_3$) δ: −63.1 ppm; ^{31}P NMR (162 MHz, $CDCl_3$) δ: 44.6 (d, J = 58.6 Hz), 32.9 (d, J = 58.6 Hz) ppm; HRMS (ESI) *m/z* calcd. for $C_{36}H_{38}F_6OP_2SNa^+$ $[M+Na]^+$: 717.1915, found: 717.1885.

(2-(Bis(4-trifluoromethylphenyl)phosphorothioyl)ethyl)bis(3,5-di-*tert*-butyl-4-methoxyphenyl)phosphine oxide (**3eb**)

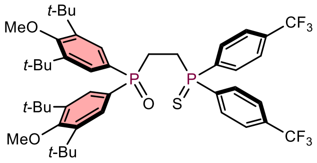

Bis(3,5-di-*tert*-butyl-4-methoxyphenyl)phosphine oxide (**1e**; 243.3 mg, 0.5 mmol, 1.0 equiv) and chlorobis(4-trifluoromethylphenyl)phosphine (**2b**; 125.6 μL, 0.5 mmol, 1.0 equiv) were employed as starting materials. Purification of the crude product by silica-gel column chromatography (eluent: hexane/ethyl acetate, 2/1) afforded **3eb** (procedure A: 260.3 mg, 0.30 mmol, 60% yield, procedure B: 335.5 mg, 0.39 mmol, 77% yield). White solid; IR (ATR): 2960, 1396, 1322, 1169, 1131, 1062, 1014, 884, 840, 793, 739,

707 cm^{-1}; ^{1}H NMR (400 MHz, $CDCl_3$) *δ*: 7.98 (dd, *J* = 12.5, 8.3 Hz, 4H), 7.72 (dd, *J* = 8.3, 1.7 Hz, 4H), 7.49 (d, *J* = 12.3Hz, 4H), 3.68 (s, 6H), 2.86-2.77 (m, 2H), 2.48-2.39 (m, 2H), 1.37 (s, 36H) ppm; ^{13}C NMR (100 MHz, $CDCl_3$) *δ*: 163.2 (d, *J* = 3.3 Hz), 144.8 (d, *J* = 12.0 Hz), 136.0 (d, *J* = 78.2 Hz), 134.0 (qd, *J* = 32.6, 2.6 Hz), 131.8 (d, *J* = 10.7 Hz), 129.6 (d, *J* = 11.0 Hz), 126.0-125.7 (m), 125.2 (d, *J* = 103.5 Hz), 123.5 (q, *J* = 271.4 Hz), 64.6, 36.1, 32.0, 25.2 (dd, *J* = 55.5, 1.4 Hz), 22.9 (dd, *J* = 68.8, 2.2 Hz) ppm; ^{19}F NMR (376 MHz, $CDCl_3$) *δ*: −63.1 ppm; ^{31}P NMR (162 MHz, $CDCl_3$) *δ*: 44.8 (d, *J* = 58.5 Hz), 36.5 (d, *J* = 58.5 Hz) ppm; HRMS (ESI) *m/z* calcd. for $C_{46}H_{58}F_6O_3P_2SNa^+$ $[M+Na]^+$: 889.3378, found: 889.3339.

(2-(Bis(4-trifluoromethylphenyl)phosphorothioyl)ethyl)bis(4-fluorophenyl)phosphine oxide (**3lb**)

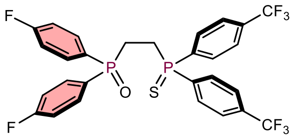

Bis(4-fluorophenyl)phosphine oxide (**1l**; 119.1 mg, 0.5 mmol, 1.0 equiv) and chlorobis(4-trifluoromethylphenyl)phosphine (**2b**; 125.6 μL, 0.5 mmol, 1.0 equiv) were employed as starting materials. Purification of the crude product by silica-gel column chromatography (eluent: hexane/ethyl acetate, 9/1 to 0/100) afforded **3lb** (procedure A: 199.1 mg, 0.32 mmol, 64% yield, procedure B: 195.8 mg, 0.32 mmol, 63% yield). White solid; IR (ATR): 3042, 1592, 1499, 1397, 1321, 1237, 1162, 1119, 1060, 1014, 829, 705 cm^{-1}; ^{1}H NMR (400 MHz, $CDCl_3$) *δ*: 7.93 (dd, *J* = 12.6, 8.0 Hz, 4H), 7.74-7.67 (m, 8H), 7.18-7.14 (m, 4H), 2.77-2.68 (m, 2H), 2.53-2.44 (m, 2H) ppm; ^{13}C NMR (100 MHz, $CDCl_3$) *δ*: 165.3 (dd, *J* = 254.4, 3.3 Hz), 135.5 (d, *J* = 78.8 Hz), 134.1 (qd, *J* = 32.9, 3.0 Hz), 133.3 (dd, *J* = 10.9, 8.9 Hz), 131.7 (d, *J* = 10.7 Hz), 127.4 (dd, *J* = 103.1, 3.6 Hz), 126.1-125.9 (m), 123.4 (q, *J* = 271.6 Hz), 116.6 (dd, *J* = 21.6, 13.0 Hz), 24.5 (dd, *J* = 56.3, 2.1 Hz), 22.7 (dd, *J* = 70.7, 1.8 Hz) ppm; ^{19}F NMR (376 MHz, $CDCl_3$) *δ*: −63.1, −105.3-−105.4 (m) ppm; ^{31}P NMR (162 MHz, $CDCl_3$) *δ*: 44.5 (d, *J* = 58.3 Hz), 32.1 (d, *J* = 58.3 Hz) ppm; HRMS (ESI) *m/z* calcd. for $C_{28}H_{20}F_8OP_2SNa^+$ $[M+Na]^+$: 641.0475, found: 641.0449.

Bis(4-chlorophenyl)(2-(Bis(4-trifluoromethylphenyl)phosphorothioyl)ethyl)phosphine oxide (**3cb**)

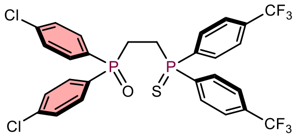

Bis(4-chlorophenyl)phosphine oxide (**1c**; 135.5 mg, 0.5 mmol, 1.0 equiv) and chlorobis(4-trifluoromethylphenyl)phosphine (**2b**; 125.6 μL, 0.5 mmol, 1.0 equiv) were employed as starting materials. Purification of the crude product by silica-gel column chromatography (eluent: hexane/ethyl acetate, 2/1) afforded **3cb** (procedure A: 255.2 mg, 0.39 mmol, 78% yield, procedure B: 173.0 mg, 0.27 mmol, 53% yield). White solid; IR (ATR): 3043, 1582, 1397, 1320, 1169, 1126, 1088, 1061, 1013, 822, 754, 705 cm^{-1}; ^{1}H NMR (400 MHz, $CDCl_3$) *δ*: 7.93 (dd, *J* = 12.6, 8.1 Hz, 4H), 7.72 (dd, *J* = 8.1, 1.8 Hz, 4H), 7.63 (dd, *J* = 11.3, 8.5 Hz, 4H), 7.45 (dd, *J* = 8.5 Hz, 2.4 Hz, 4H), 2.77-2.68 (m, 2H), 2.53-2.44 (m, 2H) ppm; ^{13}C NMR (100 MHz, $CDCl_3$) *δ*: 139.3 (d, *J* = 2.9 Hz), 135.4 (d, *J* = 78.8 Hz), 134.1 (q, *J* = 32.8 Hz), 132.2 (d, *J* = 10.5 Hz), 131.6 (d, *J* = 10.6 Hz), 129.6 (d, *J* = 11.9 Hz), 129.6 (d, *J* = 101.1 Hz), 126.1-125.9 (m), 123.3 (q, *J* = 271.8 Hz), 24.4 (d, *J* = 55.9 Hz), 22.4 (d, *J* = 70.3 Hz) ppm; ^{19}F NMR (376 MHz, $CDCl_3$) *δ*: −63.1 ppm; ^{31}P NMR (162 MHz, $CDCl_3$) *δ*: 44.5 (d, *J* = 58.0 Hz), 32.2 (d, *J* = 58.0 Hz) ppm; HRMS (ESI) *m/z* calcd. for $C_{28}H_{20}Cl_2F_6OP_2SNa^+$ $[M+Na]^+$: 672.9884, found: 672.9864.

(2-(Bis(4-trifluoromethylphenyl)phosphorothioyl)ethyl)diphenylphosphine oxide (**3ab**)

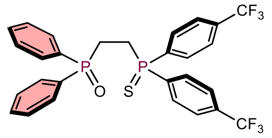

Diphenylphosphine oxide (**1a**; 101.1 mg, 0.5 mmol, 1.0 equiv) and chlorobis(4-trifluoromethylphenyl)phosphine (**2b**; 125.6 μL, 0.5 mmol, 1.0 equiv) were employed as starting materials. Purification of the crude product by silica-gel column chromatography (eluent: hexane/ethyl acetate, 5/1 to 0/100) afforded **3ab** (procedure A: 243.0 mg, 0.42 mmol, 83% yield, procedure B: 203.4 mg, 0.35 mmol, 70% yield). White solid; IR (ATR): 2942, 1436, 1399, 1321, 1188, 1175, 1120, 1061, 1015, 741, 717, 696 cm^{-1}; ^{1}H NMR (400 MHz, $CDCl_3$) δ: 7.93 (dd, J = 12.6, 8.1 Hz, 4H), 7.74-7.69 (m, 8H), 7.56-7.45 (m, 6H), 2.81-2.73 (m, 2H), 2.56-2.47 (m, 2H) ppm; ^{13}C NMR (100 MHz, $CDCl_3$) δ: 135.6 (d, J = 78.6 Hz), 134.0 (qd, J = 33.0, 3.0 Hz), 132.4, 131.7 (d, J = 10.8 Hz), 131.6 (d, J = 100.0 Hz), 130.9 (d, J = 9.0 Hz), 129.1 (d, J = 11.4 Hz), 126.0-125.9 (m), 123.4 (q, J = 271.5 Hz), 24.5 (d, J = 56.4 Hz), 22.5 (d, J = 69.1 Hz) ppm; ^{19}F NMR (376 MHz, $CDCl_3$) δ: −63.1 ppm; ^{31}P NMR (162 MHz, $CDCl_3$) δ: 44.6 (d, J = 58.5 Hz), 33.4 (d, J = 58.5 Hz) ppm; HRMS (ESI) m/z calcd. for $C_{28}H_{22}F_6OP_2SNa^+$ $[M+Na]^+$: 605.0663, found: 605.0642.

(2-(Bis(4-trifluoromethylphenyl)phosphorothioyl)ethyl)di(naphthalen-1-yl)phosphine oxide (**3mb**)

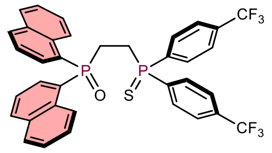

Di(naphthalen-1-yl)phosphine oxide (**1m**; 151.2 mg, 0.5 mmol, 1.0 equiv) and chlorobis(4-trifluoromethylphenyl)phosphine (**2b**; 125.6 μL, 0.5 mmol, 1.0 equiv) were employed as starting materials. Purification of the crude product by silica-gel column chromatography (eluent: hexane/ethyl acetate, 9/1 to 1/2) afforded **3mb** (procedure A: 230.4 mg, 0.34 mmol, 68% yield, procedure B: 226.3 mg, 0.33 mmol, 66% yield). White solid; IR (ATR): 3044, 1506, 1397, 1320, 1166, 1125, 1061, 1015, 800, 772, 705, 624 cm^{-1}; ^{1}H NMR (400 MHz, $CDCl_3$) δ: 8.48 (d, J = 8.5 Hz, 2H), 8.02 (d, J = 8.2 Hz, 2H), 7.95-7.83 (m, 8H), 7.61-7.59 (m, 4H), 7.49-7.45 (m, 4H), 7.36-7.32 (m, 2H), 3.00-2.80 (m, 4H) ppm; ^{13}C NMR (100 MHz, $CDCl_3$) δ: 135.5 (d, J = 78.5 Hz), 134.1-131.2 (m, 6C), 129.2, 127.5-119.2 (m, 7C), 24.7 (d, J = 56.1 Hz), 23.0 (d, J = 70.9 Hz) ppm; ^{19}F NMR (376 MHz, $CDCl_3$) δ: −63.1 ppm; ^{31}P NMR (162 MHz, $CDCl_3$) δ: 44.4 (d, J = 56.4 Hz), 37.7 (d, J = 56.4 Hz) ppm; HRMS (ESI) m/z calcd. for $C_{36}H_{26}F_6OP_2SNa^+$ $[M+Na]^+$: 705.0976, found: 705.0955.

(2-(Bis(4-trifluoromethylphenyl)phosphorothioyl)ethyl)di(naphthalen-2-yl)phosphine oxide (**3nb**)

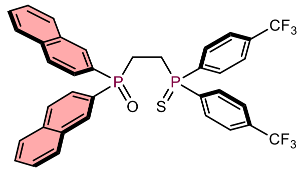

Bis(4-dimethylaminophenyl)phosphine oxide (**1n**; 151.2 mg, 0.5 mmol, 1.0 equiv) and chlorobis(4-trifluoromethylphenyl)phosphine (**2b**; 125.6 μL, 0.5 mmol, 1.0 equiv) were employed as starting materials. Purification of the crude product by silica-gel column chromatography (eluent: hexane/ethyl acetate, 9/1 to 1/2) afforded **3nb** (procedure A: 273.7 mg, 0.40 mmol, 80% yield, procedure B: 276.1 mg, 0.40 mmol, 81% yield). White solid; IR (ATR): 2938, 1326, 1314, 1165, 1123, 1093, 1061, 817, 741, 707 cm^{-1}; ^{1}H NMR (400 MHz, $CDCl_3$) δ: 8.43 (d, J = 13.4 Hz, 2H), 7.96-7.84 (m, 10H), 7.71-7.66 (m, 6H), 7.61-7.53 (m, 4H), 2.91-2.81 (m, 2H), 2.78-2.68 (m, 2H) ppm; ^{13}C NMR (100 MHz, $CDCl_3$) δ: 135.6 (d, J = 78.6 Hz), 134.9 (d, J = 2.3 Hz), 134.0 (qd, J = 33.0, 3.0 Hz), 133.1 (d, J = 8.6 Hz), 132.6 (d, J = 12.9 Hz), 131.7 (d, J = 10.6 Hz), 129.1 (d, J = 11.6 Hz), 129.0, 128.6 (d, J = 100.2 Hz), 128.6 128.0, 127.3, 126.0-125.8 (m), 125.4 (d, J = 10.6 Hz), 123.4 (q, J = 271.5 Hz), 24.7 (d, J = 56.2 Hz), 22.4 (d, J = 69.6 Hz) ppm; ^{19}F NMR (376 MHz, $CDCl_3$) δ: −63.1 ppm; ^{31}P NMR (162 MHz, $CDCl_3$) δ: 44.6 (d, J = 58.0 Hz), 33.6 (d, J =

58.0 Hz) ppm; HRMS (ESI) *m*/*z* calcd. for $C_{36}H_{26}F_6OP_2SNa^+$ $[M+Na]^+$: 705.0976, found: 705.0968.

Bis(4-dimethylaminophenyl)(2-(diphenylphosphorothioyl)ethyl)phosphine oxide (**3ga**)

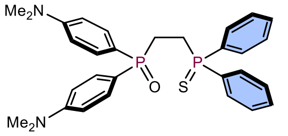

Bis(4-dimethylaminophenyl)phosphine oxide (**1g**; 144.2 mg, 0.5 mmol, 1.0 equiv) and chlorodiphenylphosphine (**2a**; 91.9 µL, 0.5 mmol, 1.0 equiv) were employed as starting materials. Purification of the crude product by silica-gel column chromatography (eluent: ethyl acetate/MeOH, 20/1) afforded **3ga** (procedure A: 126.2 mg, 0.24 mmol, 47% yield, procedure B: 216.2 mg, 0.41 mmol, 81% yield). White solid; IR (ATR): 2899, 1596, 1518, 1438, 1365, 1182, 1167, 1108, 810, 730, 693, 604 cm^{-1}; ^1H NMR (400 MHz, $CDCl_3$) δ: 7.81-7.75 (m, 4H), 7.52-7.26 (m, 10H), 6.65 (dd, J = 9.0, 2.3 Hz, 4H), 2.96 (s, 12H), 2.74-2.65 (m, 2H), 2.44-2.35 (m, 2H) ppm; ^{13}C NMR (100 MHz, $CDCl_3$) δ: 152.3 (d, J = 2.3 Hz), 132.5-131.6 (m, 3C), 131.2 (d, J = 10.2 Hz), 128.8 (d, J = 12.0 Hz), 117.3 (d, J = 110.9 Hz), 111.5 (d, J = 12.3 Hz), 40.0, 25.2 (dd, J = 55.9, 2.2 Hz), 23.1 (dd, J = 70.6, 2.7 Hz) ppm; ^{31}P NMR (162 MHz, $CDCl_3$) δ: 45.6 (d, J = 58.5 Hz), 34.8 (d, J = 58.5 Hz) ppm; HRMS (ESI) *m*/*z* calcd. for $C_{30}H_{35}N_2OP_2S^+$ $[M+H]^+$: 533.1940, found: 533.1931.

(2-(Bis(4-fluorophenyl)phosphorothioyl)ethyl)bis(4-dimethylaminophenyl)phosphine oxide (**3gg**)

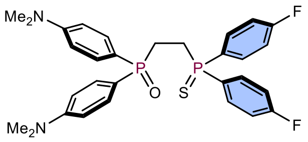

Bis(4-dimethylaminophenyl)phosphine oxide (**1g**; 144.2 mg, 0.5 mmol, 1.0 equiv) and chlorobis(4-fluorophenyl)phosphine (**2g**; 128.3 mg, 0.5 mmol, 1.0 equiv) were employed as starting materials. Purification of the crude product by silica-gel column chromatography (eluent: DCM/MeOH, 99/1 to 92/8) afforded **3gg** (procedure A: 194.4 mg, 0.34 mmol, 68% yield, procedure B: 188.0 mg, 0.33 mmol, 66% yield). White solid; IR (ATR): 2896, 1592, 1515, 1495, 1361, 1227, 1158, 1113, 1102, 811, 734, 661 cm^{-1}; ^1H NMR (400 MHz, $CDCl_3$) δ: 7.81-7.75 (m, 4H), 7.48 (dd, J = 11.0, 9.0 Hz, 4H), 7.13-7.08 (m, 4H), 6.67 (dd, J = 9.0, 2.4 Hz, 4H), 2.98 (s, 12H), 2.72-2.63 (m, 2H), 2.41-2.31 (m, 2H) ppm; ^{13}C NMR (100 MHz, $CDCl_3$) δ: 164.8 (dd, J = 254.0, 3.3 Hz), 152.2 (d, J = 2.4 Hz), 133.6 (dd, J = 11.9, 8.8 Hz), 132.1 (d, J = 10.7 Hz), 127.8 (dd, J = 83.4, 3.3 Hz), 117.0 (d, J = 111.3 Hz), 116.0 (dd, J = 21.6, 13.4 Hz), 111.4 (d, J = 12.5 Hz), 39.9, 25.4 (d, J = 56.3 Hz), 23.0 (dd, J = 70.0, 2.4 Hz) ppm; ^{19}F NMR (376 MHz, $CDCl_3$) δ: −107.0-−107.1 (m) ppm; ^{31}P NMR (162 MHz, $CDCl_3$) δ: 44.3 (d, J = 58.6 Hz), 34.6 (d, J = 58.6 Hz) ppm; HRMS (ESI) *m*/*z* calcd. for $C_{30}H_{33}F_2N_2OP_2S^+$ $[M+H]^+$: 569.1751, found: 569.1737.

(2-(Bis(4-chlorophenyl)phosphorothioyl)ethyl)bis(4-dimethylaminophenyl)phosphine oxide (**3gc**)

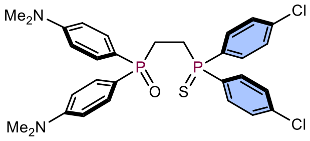

Bis(4-dimethylaminophenyl)phosphine oxide (**1g**; 144.2 mg, 0.5 mmol, 1.0 equiv) and chlorobis(4-chlorophenyl)phosphine (**2c**; 144.8 mg, 0.5 mmol, 1.0 equiv) were employed as starting materials. Purification of the crude product by silica-gel column chromatography (eluent: ethyl acetate/MeOH, 100/0 to 95/5) afforded **3gc** (procedure A: 163.6 mg, 0.27 mmol, 54% yield, procedure B: 212.8 mg, 0.37 mmol, 74% yield). White solid; IR (ATR): 2801, 1597, 1514, 1356, 1164, 1116, 1083, 1012, 815, 747, 737, 644 cm^{-1}; ^1H NMR (400 MHz, $CDCl_3$) δ: 7.69 (dd, J = 12.4, 8.5 Hz, 4H), 7.46 (dd, J = 11.1, 8.9 Hz, 4H), 7.37 (dd, J = 8.5, 2.1 Hz, 4H), 6.64 (dd, J = 8.9, 2.2 Hz, 4H), 2.95 (s, 12H), 2.71-2.62 (m, 2H), 2.38-2.29 (m, 2H) ppm; ^{13}C NMR (100 MHz, $CDCl_3$) δ: 151.7 (d, J = 2.2 Hz), 137.8 (d, J = 3.4 Hz), 132.0 (d, J = 11.2 Hz), 131.5 (d, J = 10.6 Hz), 129.8 (d, J = 81.3 Hz), 128.5 (d, J = 12.8 Hz), 116.4 (d, J = 111.0 Hz), 110.9 (d, J = 12.4 Hz), 39.4, 24.6 (d, J = 56.1 Hz),

22.6 (d, J = 70.1 Hz) ppm; ^{31}P NMR (162 MHz, $CDCl_3$) δ: 44.6 (d, J = 58.5 Hz), 34.6 (d, J = 58.5 Hz) ppm; HRMS (ESI) m/z calcd. for $C_{30}H_{33}Cl_2N_2OP_2S^+$ $[M+H]^+$: 601.1160, found: 601.1148.

(2-(Bis(3,5-bis(trifluoromethyl)phenyl)phosphorothioyl)ethyl)bis(4-dimethylaminophenyl)phosphine oxide (**3gh**)

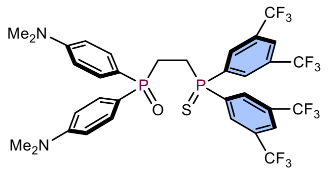

Bis(4-dimethylaminophenyl)phosphine oxide (**1g**; 144.2 mg, 0.5 mmol, 1.0 equiv) and chlorobis(3,5-bis(trifluoromethyl)phenyl)phosphine (**2h**; 246.3 mg, 0.5 mmol, 1.0 equiv) were employed as starting materials. Purification of the crude product by silica-gel column chromatography (eluent: hexane/ethyl acetate, 1/1) afforded **3gh** (procedure A: 204.3 mg, 0.25 mmol, 51% yield, procedure B: 281.2 mg, 0.35 mmol, 70% yield). White solid; IR (ATR): 2907, 1598, 1518, 1358, 1277, 1179, 1114, 1096, 909, 701, 681, 644 cm^{-1}; ^{1}H NMR (400 MHz, $CDCl_3$) δ: 8.28 (d, J = 12.4 Hz, 4H), 8.03 (s, 2H), 7.48 (dd, J = 11.1, 9.0 Hz, 4H), 6.68 (dd, J = 9.0, 2.3 Hz, 4H), 2.99 (s, 12 H), 2.90-2.81 (m, 2H), 2.40-2.31 (m, 2H) ppm; ^{13}C NMR (100 MHz, $CDCl_3$) δ: 152.5 (d, J = 2.4 Hz), 134.8 (d, J = 77.9 Hz), 133.3-132.1 (m, 2C), 131.2-131.1 (m), 126.2 (br), 122.6 (q, J = 272.1 Hz), 116.4 (d, J = 112.3 Hz), 111.6 (d, J = 12.5 Hz), 40.0, 25.0 (d, J = 56.5 Hz), 23.0 (d, J = 68.1 Hz) ppm; ^{19}F NMR (376 MHz, $CDCl_3$) δ: −62.7 ppm; ^{31}P NMR (162 MHz, $CDCl_3$) δ: 44.8 (d, J = 58.5 Hz), 34.0 (d, J = 58.5 Hz) ppm; HRMS (ESI) m/z calcd. for $C_{34}H_{30}F_{12}N_2OP_2SNa^+$ $[M+Na]^+$: 827.1255, found: 827.1223.

Bis(4-dimethylaminophenyl) (2-(Bis(4-methylphenyl)phosphorothioyl)ethyl)phosphine oxide (**3gi**)

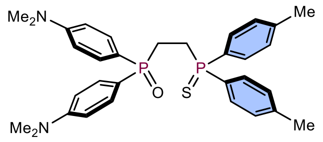

Bis(4-dimethylaminophenyl)phosphine oxide (**1g**; 144.2 mg, 0.5 mmol, 1.0 equiv) and chlorobis(4-methylphenyl)phosphine (**2i**; 107.3 μL, 0.5 mmol, 1.0 equiv) were employed as starting materials. Purification of the crude product by silica-gel column chromatography (eluent: DCM/acetone, 95/5 to 3/2) afforded **3gi** (procedure A: 118.9 mg, 0.21 mmol, 42% yield, procedure B: 122.0 mg, 0.22 mmol, 44% yield). White solid; IR (ATR): 2909, 1596, 1517, 1363, 1164, 1116, 1101, 809, 768, 733, 654, 631 cm^{-1}; ^{1}H NMR (400 MHz, $CDCl_3$) δ: 7.66 (dd, J = 12.7, 8.2 Hz, 4H), 7.49 (dd, J = 11.0, 8.8 Hz, 4H), 7.20 (dd, J = 8.2, 2.7 Hz, 4H), 6.66 (dd, J = 8.8, 2.2 Hz, 4H), 2.97 (s, 12H), 2.70-2.56 (m, 2H), 2.43-2.35 (m, 8H) ppm; ^{13}C NMR (100 MHz, $CDCl_3$) δ: 152.1 (d, J = 2.3 Hz), 141.9 (d, J = 2.9 Hz), 132.1 (d, J = 10.6 Hz), 131.1 (d, J = 10.6 Hz), 129.4-128.4 (m, 2C), 117.3 (d, J = 110.7 Hz), 111.4 (d, J = 12.4 Hz), 39.9, 25.2 (d, J = 56.1 Hz), 23.0 (d, J = 70.7 Hz), 21.4 ppm; ^{31}P NMR (162 MHz, $CDCl_3$) δ: 45.1 (d, J = 58.5 Hz), 34.7 (d, J = 58.5 Hz) ppm; HRMS (ESI) m/z calcd. for $C_{32}H_{39}N_2OP_2S^+$ $[M+H]^+$: 561.2226, found: 561.2241.

Bis(4-dimethylaminophenyl)(2-(Bis(3,5-dimethylphenyl)phosphorothioyl)ethyl)phosphine oxide (**3gj**)

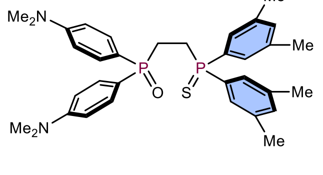

Bis(4-dimethylaminophenyl)phosphine oxide (**1g**; 144.2 mg, 0.5 mmol, 1.0 equiv) and chlorobis(3,5-dimethylphenyl)phosphine (**2j**; 125.6 μL, 0.5 mmol, 1.0 equiv) were employed as starting materials. Purification of the crude product by silica-gel column chromatography (eluent: ethyl acetate/MeOH, 20/1) afforded **3gj** (procedure A: 99.6 mg, 0.17 mmol, 34% yield, procedure B: 224.9 mg, 0.38 mmol, 76% yield). White solid; IR (ATR): 2911, 1595, 1515, 1361, 1161, 1114, 812, 768, 734, 691, 642 cm^{-1}; ^{1}H NMR (400 MHz, $CDCl_3$) δ: 7.49 (dd, J = 11.0, 8.6 Hz, 4H), 7.37 (d, J = 13.1 Hz, 4H), 7.06 (s, 2H), 6.65 (dd, J = 8.6,

1.8 Hz, 4H), 2.95 (s, 12H), 2.73-2.64 (m, 2H), 2.44-2.33 (m, 2H), 2.29 (s, 12H) ppm; ^{13}C NMR (100 MHz, $CDCl_3$) δ: 152.2, 138.4 (d, J = 12.7 Hz), 133.4 (d, J = 3.4 Hz), 132.2 (d, J = 10.6 Hz), 131.9 (d, J = 79.1 Hz), 128.7 (d, J = 10.1 Hz), 117.5 (d, J = 110.7 Hz), 111.5 (d, J = 12.4 Hz), 40.0, 24.9 (d, J = 55.6 Hz), 22.9 (d, J = 70.6 Hz), 21.3 ppm; ^{31}P NMR (162 MHz, $CDCl_3$) δ: 45.5 (d, J = 58.5 Hz), 34.9 (d, J = 58.5 Hz) ppm; HRMS (ESI) *m/z* calcd. for $C_{34}H_{43}N_2OP_2S^+$ $[M+H]^+$: 589.2566, found: 589.2550.

(2-(Di(furan-2-yl)phosphorothioyl)ethyl)bis(4-dimethylaminophenyl)phosphine oxide (**3gd**)

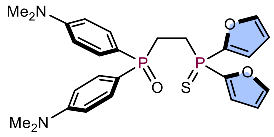

Bis(4-dimethylaminophenyl)phosphine oxide (**1g**; 144.2 mg, 0.5 mmol, 1.0 equiv) and chlorodi(furan-2-yl)phosphine (**2d**; 77.8 μL, 0.5 mmol, 1.0 equiv) were employed as starting materials. Purification of the crude product by silica-gel column chromatography (eluent: DCM/acetone, 9/1 to 1/1 and DCM/MeOH, 97/3) afforded **3gd** (procedure A: 92.0 mg, 0.18 mmol, 36% yield, procedure). White solid; IR (ATR): 2910, 1595, 1516, 1363, 1162, 1114, 1003, 813, 736, 654 cm^{-1}; ^{1}H NMR (400 MHz, $CDCl_3$) δ: 7.63 (ddd, J = 2.4, 1.7, 0.8 Hz, 2H), 7.49 (dd, J = 11.0, 8.8 Hz, 4H), 7.12 (ddd, J = 3.4, 2.2, 0.8 Hz, 2H), 6.67 (dd, J = 8.8, 2.1 Hz, 4H), 6.44 (ddd, J = 3.4, 1.6, 1.6 Hz, 2H), 2.96 (s, 12H), 2.71-2.62 (m, 2H), 2.42-2.30 (m, 2H) ppm; ^{13}C NMR (100 MHz, $CDCl_3$) δ: 152.3, 148.7 (d, J = 7.1 Hz), 146.4 (d, J = 117.7 Hz), 132.2 (d, J = 10.6 Hz), 122.8 (d, J = 20.5 Hz), 117.2 (d, J = 111.0 Hz), 111.5 (d, J = 12.4 Hz), 111.2 (d, J = 9.0 Hz), 40.0, 25.3 (d, J = 62.0 Hz), 22.5 (d, J = 70.9 Hz) ppm; ^{31}P NMR (162 MHz, $CDCl_3$) δ: 34.3 (d, J = 63.6 Hz), 18.1 (d, J = 63.6 Hz) ppm; HRMS (ESI) *m/z* calcd. for $C_{26}H_{30}N_2O_3P_2SNa^+$ $[M+Na]^+$: 535.1345, found: 535.1332.

(2-(Bis(pentafluorophenyl)phosphanyl)ethyl)bis(4-dimethylaminophenyl)phosphine oxide (**3gk**)

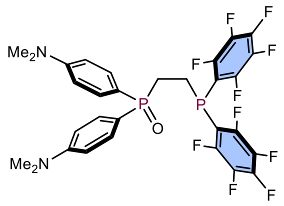

Bis(4-dimethylaminophenyl)phosphine oxide (**1g**; 144.2 mg, 0.5 mmol, 1.0 equiv) and chlorobispentafluorophenylphosphine (**2k**; 200.3 mg, 0.5 mmol, 1.0 equiv) were employed as starting materials. The reactions were conducted without the treatment of sulfur. Purification of the crude product by silica-gel column chromatography (eluent: ethyl acetate/MeOH, 99/1) afforded **3gk** (procedure A: 112.4 mg, 0.17 mmol, 33% yield, procedure B: 15.5 mg, 0.023 mmol, 5% yield). White solid; IR (ATR): 2911, 1597, 1469, 1362, 1116, 1082, 974, 813, 734, 528 cm^{-1}; ^{1}H NMR (400 MHz, $CDCl_3$) δ: 7.45 (dd, J = 10.9, 8.9 Hz, 4H), 6.67 (dd, J = 8.9, 2.1 Hz, 4H), 2.98 (s, 12H), 2.73-2.66 (m, 2H), 2.21-2.12 (m, 2H) ppm; ^{13}C NMR (100 MHz, $CDCl_3$) δ: 152.4 (d, J = 1.9 Hz), 149.0-146.3 (m), 143.9-141.0 (m), 139.0-136.1 (m), 132.2 (d, J = 10.5 Hz), 116.9 (d, J = 111.1 Hz), 111.4 (d, J = 12.5 Hz), 108.7-107.9 (m), 40.0, 27.4 (dd, J = 69.7, 21.3 Hz), 16.4-16.1 (m) ppm; ^{19}F NMR (376 MHz, $CDCl_3$) δ: −129.3-−129.5 (m), −149.3-−149.5 (m), −159.7-−159.8 (m) ppm; ^{31}P NMR (162 MHz, $CDCl_3$) δ: 33.6 (d, J = 58.6 Hz), −41.9-−42.9 (m) ppm; HRMS (ESI) *m/z* calcd. for $C_{30}H_{24}F_{10}N_2OP_2Na^+$ $[M+Na]^+$: 703.1096, found: 703.1067.

(2-(Bis(4-chlorophenyl)phosphorothioyl)ethyl)(2-methylphenyl)phenylphosphine oxide (**3jc**)

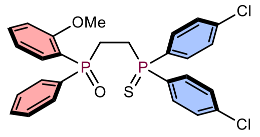

(2-Methoxyphenyl)phenylphosphine oxide (**1j**; 116.1 mg, 0.5 mmol, 1.0 equiv) and chlorobis(4-chlorophenyl)phosphine (**2c**; 144.8 mg, 0.5 mmol, 1.0 equiv) were employed as starting materials. Purification of the crude product by silica-gel column chromatography (eluent: hexane/ethyl acetate, 4/1 to 1/3) afforded **3jc** (procedure A: 128.3 mg, 0.24 mmol, 47% yield, procedure B: 195.5 mg, 0.36 mmol, 71% yield). White solid; IR (ATR): 2935, 1577, 1476, 1271,

1177, 1113, 1085, 1011, 745, 692, 635, 620 cm^{-1}; ^{1}H NMR (400 MHz, $CDCl_3$) δ: 7.97-7.92 (m, 1H), 7.76-7.67 (m, 6H), 7.53-7.45 (m, 2H), 7.43-7.37 (m, 6H), 7.12-7.07 (m, 1H), 6.87 (dd, J = 8.3, 5.3 Hz, 1H), 3.73 (s, 3H), 2.84-2.70 (m, 2H), 2.61-2.42 (m, 2H) ppm; ^{13}C NMR (100 MHz, $CDCl_3$) δ: 159.8 (d, J = 4.7 Hz), 138.7-138.6 (m), 134.6 (d, J = 5.5 Hz), 134.5 (d, J = 1.5 Hz), 133.3-132.3 (m, 2C), 131.9 (d, J = 2.8 Hz), 130.8-129.8 (m, 2C), 129.3-129.1 (m), 128.5 (d, J = 12.1 Hz), 121.3 (d, J = 10.9 Hz), 119.0 (d, J = 98.2 Hz), 110.9 (d, J = 6.8 Hz), 55.5, 25.1 (dd, J = 57.0, 3.1 Hz), 22.1 (dd, J = 71.1, 1.8 Hz) ppm; ^{31}P NMR (162 MHz, $CDCl_3$) δ: 44.6 (d, J = 62.9 Hz), 33.3 (d, J = 62.9 Hz) ppm; HRMS (ESI) m/z calcd. for $C_{27}H_{24}Cl_2O_2P_2SNa^+$ [M+Na]$^+$: 567.0242, found: 567.0232.

(2-(Bis(4-chlorophenyl)phosphorothioyl)ethyl)bis(3,5-di-*tert*-butyl-4-methoxyphenyl)phosphine oxide (**3ec**)

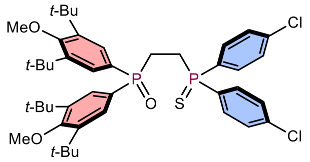

Bis(3,5-di-*tert*-butyl-4-methoxyphenyl) oxide (**1e**; 243.3 mg, 0.5 mmol, 1.0 equiv) and chlorobis(4-chlorophenyl)phosphine (**2c**; 144.8 mg, 0.5 mmol, 1.0 equiv) were employed as starting materials. Purification of the crude product by silica-gel column chromatography (eluent: hexane/ethyl acetate, 4/1 to 2/3) afforded **3ec** (procedure A: 240.8 mg, 0.30 mmol, 60% yield, procedure B: 178.6 mg, 0.22 mmol, 45% yield). White solid; IR (ATR): 2958, 1577, 1480, 1390, 1169, 1147, 1115, 1086, 1011, 884, 748, 639, 623 cm^{-1}; ^{1}H NMR (400 MHz, $CDCl_3$) δ: 7.73 (dd, J = 12.4, 8.5 Hz, 4H), 7.47 (d, J = 12.3 Hz, 4H), 7.38 (dd, J = 8.5, 2.2 Hz, 4H), 3.65 (s, 6H), 2.75-2.66 (m, 2H), 2.46-2.37 (m, 2H), 1.35 (s, 36H) ppm; ^{13}C NMR (100 MHz, $CDCl_3$) δ: 162.9 (d, J = 3.3 Hz), 144.5 (d, J = 11.8 Hz), 138.6 (d, J = 3.4 Hz), 132.5 (d, J = 11.2 Hz), 130.2 (d, J = 81.8 Hz), 129.4 (d, J = 11.1 Hz), 129.1 (d, J = 12.8 Hz), 125.2 (d, J = 103.7 Hz), 64.4, 36.0, 31.9, 25.3 (d, J = 55.2 Hz), 22.9 (d, J = 69.0 Hz) ppm; ^{31}P NMR (162 MHz, $CDCl_3$) δ: 44.6 (d, J = 58.5 Hz), 35.8 (d, J = 58.5 Hz) ppm; HRMS (ESI) m/z calcd. for $C_{44}H_{58}Cl_2O_3P_2SNa^+$ [M+Na]$^+$: 821.2851, found: 821.2835.

(2-(Bis(3,5-bis(trifluoromethyl)phenyl)phosphorothioyl)ethyl)bis(3,5-di-*tert*-butyl-4-methoxyphenyl)phosphine oxide (**3eh**)

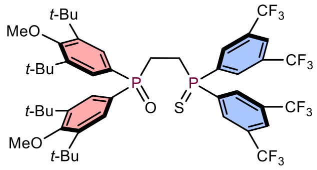

Bis(3,5-di-*tert*-butyl-4-methoxyphenyl) oxide (**1e**; 243.3 mg, 0.5 mmol, 1.0 equiv) and chlorobis(3,5-bis(trifluoromethyl)phenyl)phosphine (**2h**; 246.3 mg, 0.5 mmol, 1.0 equiv) were employed as starting materials. Purification of the crude product by silica-gel column chromatography (eluent: hexane/acetone, 7/1) afforded **3eh** (procedure A: 301.7 mg, 0.30 mmol, 60% yield, procedure B: 282.1 mg, 0.28 mmol, 56% yield). White solid; IR (ATR): 2964, 1360, 1280, 1170, 1134, 1124, 1100, 1009, 902, 733, 682, 637 cm^{-1}; ^{1}H NMR (400 MHz, $CDCl_3$) δ: 8.31 (d, J = 12.5 Hz, 4H), 8.04 (s, 2H), 7.51 (d, J = 12.4 Hz, 4H), 3.68 (s, 6H), 2.92-2.84 (m, 2H), 2.51-2.41 (m, 2H), 1.37 (s, 36H) ppm; ^{13}C NMR (100 MHz, $CDCl_3$) δ: 163.3 (d, J = 3.3 Hz), 144.9 (d, J = 12.0 Hz), 134.7 (d, J = 78.2 Hz), 132.9 (qd, J = 34.2, 12.2 Hz), 131.2-131.1 (m), 129.6 (d, J = 11.0 Hz), 126.4-126.3 (m), 124.9 (d, J = 104.5 Hz), 122.6 (q, J = 272.0 Hz), 64.5, 36.1, 31.9, 25.4 (d, J = 56.5 Hz), 22.8 (d, J = 67.5 Hz) ppm; ^{19}F NMR (376 MHz, $CDCl_3$) δ: −62.7 ppm; ^{31}P NMR (162 MHz, $CDCl_3$) δ: 44.8 (d, J = 58.4 Hz), 35.3 (d, J = 58.4 Hz) ppm; HRMS (ESI) m/z calcd. for $C_{48}H_{56}F_{12}O_3P_2SNa^+$ [M+Na]$^+$: 1025.3126, found: 1025.3104

(2-(Bis(pentafluorophenyl)phosphanyl)ethyl)bis(3,5-di-*tert*-butyl-4-methoxyphenyl)phosphine oxide (**3ek**)

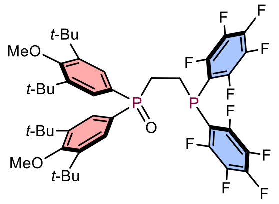

Bis(3,5-di-*tert*-butyl-4-methoxyphenyl) oxide (**1e**; 243.3 mg, 0.5 mmol, 1.0 equiv) and chlorobispentafluorophenylphosphine (**2k**; 200.3 mg, 0.5 mmol, 1.0 equiv) were employed as starting materials. The reactions were conducted without the treatment of sulfur. Purification of the crude product by silica-gel column chromatography (eluent: DCM/acetone, 50/1) afforded **3ek** (procedure A: 130.6 mg, 0.15 mmol, 30% yield, procedure B: 55.8 mg, 0.064 mmol, 13% yield). White solid; IR (ATR): 2964, 1516, 1469, 1148, 1116, 1086, 1008, 974, 881, 476 cm^{-1}; ^{1}H NMR (400 MHz, $CDCl_3$) δ: 7.51 (d, J = 12.3 Hz, 4H), 3.69 (s, 6H), 2.74-2.67 (m, 2H), 2.33-2.24 (m, 2H), 1.39 (s, 36H) ppm; ^{13}C NMR (100 MHz, $CDCl_3$) δ: 163.1 (d, J = 3.3 Hz), 149.1-146.3 (m), 144.7 (d, J = 11.9 Hz), 144.0-141.2 (m), 139.1-136.2 (m), 129.6 (d, J = 10.9 Hz), 125.3 (d, J = 103.4 Hz), 108.9-107.7 (m), 64.6, 36.1, 31.9, 27.7 (dd, J = 67.9, 22.5 Hz), 17.0-16.8 (m) ppm; ^{19}F NMR (376 MHz, $CDCl_3$) −129.7-−129.9 (m), −148.9-−149.0 (m), −159.4-−159.5 (m) ppm; ^{31}P NMR (162 MHz, $CDCl_3$) 34.5 (d, J = 58.2 Hz), −41.4-−42.4 (m) ppm; HRMS (ESI) *m*/*z* calcd. for $C_{44}H_{50}F_{10}O_3P_2Na^+$ $[M+Na]^+$: 901.2968, found: 901.2937.

(G) Synthesis of 4aa and 4gb

Diphosphine oxide **4aa** was synthesized according to the literature[3].

To a 50 mL round bottom flask, methoxydiphenylphosphine (3.76 mL, 20 mmol) and chlorodiphenylphosphine (3.59 mL, 20 mmol) dissolved in degassed benzene (10 mL) was added under nitrogen atmosphere, and the mixture was refluxed for 3 h. The solvent was removed by decantation and washed with benzene under nitrogen atmosphere by using cannula to afford the product as a white solid.

1,1,2,2-Tetraphenyldiphosphine 1-oxide (**4aa**)

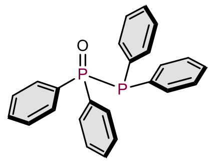

White solid; IR (ATR): 1434, 1175, 1092, 739, 690, 556, 513, 496, 463, 435 cm^{-1}; ^{1}H NMR (400 MHz, $CDCl_3$) δ: 7.80-7.75 (m, 4H), 7.61-7.57 (m, 4H), 7.44-7.21 (m, 12H) ppm; ^{13}C NMR (100 MHz, $CDCl_3$) δ: 135.3 (dd, J = 19.5, 7.1 Hz), 133.8 (dd, J = 85.4, 14.0 Hz), 131.5-129.8 (m, 3C), 129.6, 128.5-128.2 (m, 2C) ppm; ^{31}P NMR (162 MHz, $CDCl_3$) 36.6 (d, J = 227.6 Hz), −22.0 (d, J = 227.6 Hz) ppm; HRMS (ESI) *m*/*z* calcd. for $C_{24}H_{20}OP_2Na^+$ $[M+Na]^+$: 409.0882, found: 409.0884.

Diphosphine oxide **4gb** was synthesized by following procedure.

In an oven-dried test tube were placed phosphine oxide **1g** (1.44g, 5 mmol, 1.0 equiv). After the addition of DCM (15 mL) under nitrogen, chlorophosphine **2b** (1.26 mL, 5 mmol, 1.0 equiv) and DBU (0.75 mL, 5 mmol, 1.0 equiv) were added. After the resulting mixture was stirred at room temperature for 30 min, the solvent was evaporated, and crude product was purified by silica-gel column chromatography (eluent: DCM/Acetone, 9/1) under nitrogen to afford **4gb** in 70%, of which isolated yield was lower than NMR yield because only high-purity fractions of chromatography were collected.

1,1-Bis(4-dimethylaminophenyl)-2,2-bis(4-trifluoromethylphenyl)diphosphine 1-oxide (**4gb**)

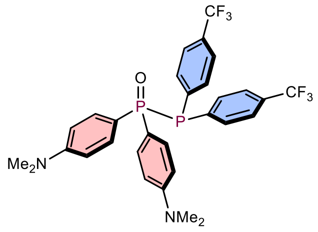

White solid; IR (ATR): 1594, 1320, 1163, 1106, 1058, 1011, 811, 605, 549, 517 cm^{-1}; ^{1}H NMR (400 MHz, $CDCl_3$) δ: 7.67-7.62 (m, 4H), 7.53-7.47 (m, 8H), 6.62 (dd, J = 9.0, 2.6 Hz, 4H), 2.98 (s, 12H) ppm; ^{13}C NMR (100 MHz, $CDCl_3$) δ: 152.3, 136.6-135.3 (m, 2C), 132.7 (dd, J = 10.5, 5.8 Hz), 131.2 (q, J = 32.2 Hz), 125.2-125.1 (m), 124.1 (q, J = 270.9 Hz), 118.1 (dd, J = 100.0, 15.9 Hz), 111.5 (d, J = 12.6 Hz), 40.1 ppm; ^{19}F NMR (376 MHz, $CDCl_3$) −62.8 ppm; ^{31}P NMR (162 MHz, $CDCl_3$) 37.9 (d, J = 206.1 Hz), −23.6 (d, J = 206.1 Hz) ppm; HRMS (ESI) m/z calcd. for $C_{30}H_{28}F_6N_2OP_2Na^+$ $[M+Na]^+$: 631.1473, found: 631.1477.

(H) Gram Scale Synthesis of 3gb

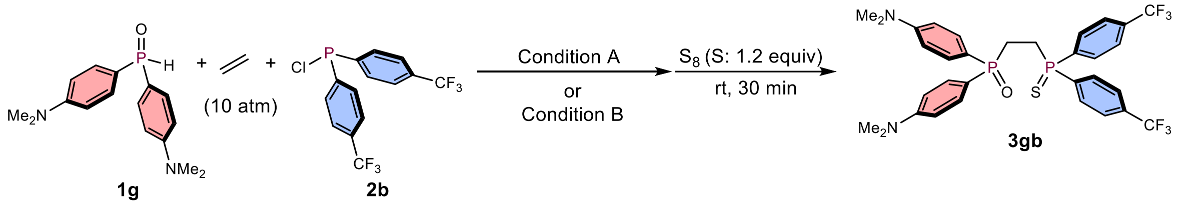

General Procedure A (for blue LED)

In an oven-dried 10 mL pressure-resistant glass tube were placed phosphine oxide **1g** (1.44 g, 5.0 mmol, 1.0 equiv) and $[Ir(ppy)_2(dtbbpy)]PF_6$ (9.1 mg, 0.01 mmol, 0.2 mol%). After the addition of DCE (15 mL) under nitrogen, chlorophosphine **2b** (1.26 mL, 5.0 mmol, 1.0 equiv) and DBU (0.75 mL, 5.0 mmol, 1.0 equiv) were added. Then, the tube was placed into autoclave which consists of a polycarbonate cylinder, and ethylene gas was pressurised to 10 atm. After the resulting mixture was stirred at room temperature for 4h under the irradiation of blue LED (45W PR160L-440 nm Kessil light × 2), sulfur (192 mg, 6 mmol, 1.2 equiv of S) was added into the reaction mixture. After the mixture was stirred for 30 min, the solvent was evaporated to give the crude mixture. The crude product was purified by silica-gel column chromatography (DCM/acetone = 9/1 to 4/1) to afford the product **3gb** (2.19 g, 3.3 mmol, 65%).

General Procedure B (for white LED)

In an oven-dried 10 mL pressure-resistant glass tube was placed phosphine oxide **1g** (1.44 g, 5.0 mmol, 1.0 equiv). After the addition of DCE (15 mL) under nitrogen, chlorophosphine **2b** (1.26 mL, 5.0 mmol, 1.0 equiv) and DBU (0.75 mL, 5.0 mmol, 1.0 equiv) were added. Then, the tube was placed into autoclave which consists of a polycarbonate cylinder, and ethylene gas was pressurised to 10 atm. After the resulting mixture was stirred at room temperature for 24h under the irradiation of white LED (A 160WE TUNA SUN × 4), sulfur (192 mg, 6 mmol, 1.2 equiv of S) was added into the reaction mixture. After the mixture was stirred for 30 min, the solvent was evaporated to give the crude mixture. The crude product was purified by silica-gel column chromatography (DCM/acetone = 9/1 to 4/1) to afford the product **3gb** (2.20 g, 3.3 mmol, 66%).

(I) Transformation of Oxide Sulfide 3 to Dioxide 5[4]

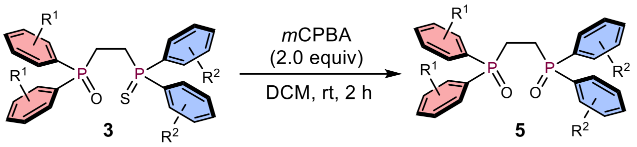

DPPE derivatives **3** (1.0 equiv) was placed in a round bottom flask and dissolved into DCM (0.05 M). After the solution was cooled to 0 °C, *m*CPBA (2.0 equiv) was slowly added, and then, the mixture was stirred for 2h. The reaction mixture was then quenched with 5% aq. $NaHCO_3$ and extracted with DCM. The organic layer was dried with Na_2SO_4, filtered, and concentrated under reduced pressure. The product was purified by silica-gel column chromatography to afford the product **5**.

(2-(Bis(4-trifluoromethylphenyl)phosphinyl)ethyl)bis(4-dimethylaminophenyl)phosphine oxide (**5gb**)

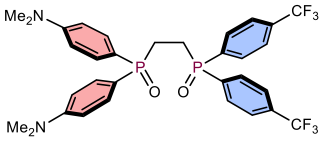

3gb (334.3 mg, 0.5 mmol, 1.0 equiv) was employed as starting materials. Purification of the crude product by silica-gel column chromatography (eluent: DCM/MeOH, 9/1) afforded **5gb** (295.0 mg, 0.45 mmol, 90% yield). White solid; IR (ATR): 2910, 1599, 1517, 1317, 1165, 1117, 1061, 1017, 816, 781, 743, 709 cm^{-1}; ^{1}H NMR (400 MHz, $CDCl_3$) *δ*: 7.84 (dd, *J* = 10.9, 8.2 Hz, 4H), 7.69 (dd, *J* = 8.2, 1.8 Hz, 4H), 7.46 (dd, *J* = 11.0, 9.0 Hz, 4H), 6.65 (dd, *J* = 9.0, 2.2 Hz, 4H), 2.96 (s, 12H), 2.62-2.53 (m, 2H), 2.39-2.31 (m, 2H) ppm; ^{13}C NMR (100 MHz, $CDCl_3$) *δ*: 152.4 (d, *J* = 2.4 Hz), 136.1 (d, *J* = 96.2 Hz), 134.1 (qd, *J* = 33.0, 2.9 Hz), 132.2 (d, *J* = 10.6 Hz), 131.4 (d, *J* = 9.6 Hz), 125.9-125.8 (m), 128.0 (q, *J* = 271.5 Hz), 116.8 (d, *J* = 110.9 Hz), 111.5 (d, *J* = 12.3 Hz), 40.0, 22.0 (dd, *J* = 69.5, 3.9 Hz), 21.8 (dd, *J* = 70.8, 2.9 Hz) ppm; ^{19}F NMR (376 MHz, $CDCl_3$) *δ*: −63.2 ppm; ^{31}P NMR (162 MHz, $CDCl_3$) *δ*: 34.0 (d, *J* = 52.0 Hz), 32.0 (d, *J* = 52.0 Hz) ppm; HRMS (ESI) *m/z* calcd. for $C_{32}H_{32}F_6N_2O_2P_2Na^+$ $[M+Na]^+$: 675.1735, found: 675.1727.

(2-(Bis(4-trifluoromethylphenyl)phosphinyl)ethyl)bis(4-methoxyphenyl)phosphine oxide (**5fb**)

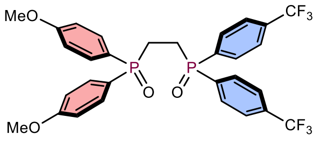

3fb (125.5 mg, 0.2 mmol, 1.0 equiv) was employed as starting materials. Purification of the crude product by silica-gel column chromatography (eluent: DCM/acetone, 1/1) afforded **5fb** (114.4 mg, 0.18 mmol, 91% yield). White solid; IR (ATR): 2914, 1598, 1504, 1324, 1171, 1121, 1102, 1062, 1018, 830, 749, 710 cm^{-1}; ^{1}H NMR (400 MHz, $CDCl_3$) *δ*: 7.86 (dd, *J* = 10.9, 8.2 Hz, 4H), 7.73 (dd, *J* = 8.2, 1.9 Hz, 4H), 7.60 (dd, *J* = 11.0, 8.8 Hz, 4H), 6.95 (dd, *J* = 8.8, 2.2 Hz, 4H), 3.83 (s, 6H), 2.62-2.52 (m, 2H), 2.49-2.39 (m, 2H) ppm; ^{13}C NMR (100 MHz, $CDCl_3$) *δ*: 162.7 (d, *J* = 2.8 Hz), 135.8 (d, *J* = 95.6 Hz), 134.3 (qd, *J* = 32.8, 2.4 Hz), 132.7 (d, *J* = 10.6 Hz), 131.4 (d, *J* = 9.6 Hz), 126.1-125.9 (m), 123.4 (q, *J* = 271.5 Hz), 122.9 (d, *J* = 105.6 Hz), 114.6 (d, *J* = 12.6 Hz), 55.4, 22.1 (dd, *J* = 28.3, 2.9 Hz), 21.4 (dd, *J* = 29.2, 2.9 Hz) ppm; ^{19}F NMR (376 MHz, $CDCl_3$) *δ*: −63.2 ppm; ^{31}P NMR (162 MHz, $CDCl_3$) *δ*: 34.5 (d, *J* = 53.0 Hz), 32.2 (d, *J* = 53.0 Hz) ppm; HRMS (ESI) *m/z* calcd. for $C_{30}H_{26}F_6O_4P_2Na^+$ $[M+Na]^+$: 649.1103, found: 649.1085.

(2-(Bis(4-trifluoromethylphenyl)phosphinyl)ethyl)bis(2-methoxyphenyl)phosphine oxide (**5jb**)

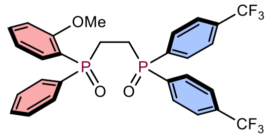

3jb (122.5 mg, 0.20 mmol, 1.0 equiv) was employed as starting materials. Purification of the crude product by silica-gel column chromatography (eluent: DCM/MeOH, 100/0 to 15/1) afforded **5jb** (116.3 mg, 0.195 mmol, 97% yield). White solid; IR (ATR): 2940, 1590, 1478, 1435, 1399, 1319, 1167, 1124, 1060, 1015, 800, 745 cm^{-1}; ^{1}H NMR (400 MHz, $CDCl_3$) *δ*: 7.58-7.55 (m, 4H), 7.46-7.28 (m, 10H), 6.92-6.81 (m, 3H), 3.67 (s, 3H), 2.28-1.95 (m, 4H) ppm; ^{13}C NMR (100 MHz, $CDCl_3$) *δ*: 159.8 (d, *J* = 4.2 Hz), 136.5-131.9 (m, 6C), 131.4 (d, *J* = 9.2 Hz), 130.7 (d, *J* = 9.4 Hz), 128.5 (d, *J* = 11.5 Hz), 126.0-125.8 (m), 123.5 (q, *J* = 271.5 Hz), 121.4 (d, *J* = 10.4 Hz), 119.0 (dd, *J* = 92.1, 6.9 Hz), 111.0 (d, *J* = 6.5 Hz), 55.4, 21.6 (d, *J* = 67.2 Hz), 21.0 (d, *J* = 66.4 Hz) ppm; ^{19}F NMR (376 MHz, $CDCl_3$) *δ*: −63.2 ppm; ^{31}P NMR (162 MHz, $CDCl_3$) *δ*: 32.5 (d, *J* = 54.9 Hz), 31.7 (d, *J* = 54.9 Hz) ppm; HRMS (ESI) *m*/*z* calcd. for $C_{29}H_{24}F_6O_3P_2Na^+$ $[M+Na]^+$: 619.0997, found: 619.0982.

(2-(Bis(4-trifluoromethylphenyl)phosphinyl)ethyl)bis(3,5-di-*tert*-butyl-4-methoxyphenyl)phosphine oxide (**5eb**)

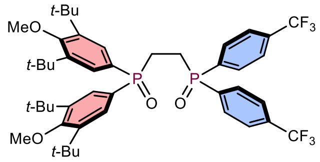

3eb (216.7 mg, 0.25 mmol, 1.0 equiv) was employed as starting materials. Analytically pure product **5eb** was obtained without silica-gel column chromatography (212.3 mg, 0.25 mmol, quantitative yield). White solid; IR (ATR): 2960, 1700, 1397, 1321, 1170, 1131, 1062, 1009, 885, 838, 794, 746 cm^{-1}; ^{1}H NMR (400 MHz, $CDCl_3$) *δ*: 7.93 (dd, *J* = 11.2, 8.0 Hz, 4H), 7.74 (dd, *J* = 8.0, 2.0 Hz, 4H), 7.50 (d, *J* = 12.3 Hz, 4H), 3.68 (s, 6H), 2.75-2.67 (m, 2H), 2.47-2.39 (m, 2H), 1.36 (s, 36H) ppm; ^{13}C NMR (100 MHz, $CDCl_3$) *δ*: 163.2 (d, *J* = 3.3 Hz), 144.8 (d, *J* = 12.0 Hz), 136.0 (d, *J* = 96.5 Hz), 134.3 (qd, *J* = 33.1, 2.6 Hz), 131.5 (d, *J* = 9.7 Hz), 129.5 (d, *J* = 11.0 Hz), 126.1-125.9 (m), 125.1 (d, *J* = 103.1 Hz), 123.5 (q, *J* = 271.7 Hz), 64.6, 36.1, 31.9, 22.4-21.6 (m, 2C) ppm; ^{19}F NMR (376 MHz, $CDCl_3$) *δ*: −63.2 ppm; ^{31}P NMR (162 MHz, $CDCl_3$) *δ*: 35.3 (d, *J* = 52.5 Hz), 31.9 (d, *J* = 52.5 Hz) ppm; HRMS (ESI) *m*/*z* calcd. for $C_{46}H_{58}F_6O_4P_2Na^+$ $[M+Na]^+$: 873.3607, found: 873.3592.

(2-(Bis(4-chlorophenyl)phosphinyl)ethyl)bis(3,5-di-*tert*-butyl-4-methoxyphenyl)phosphine oxide (**5ec**)

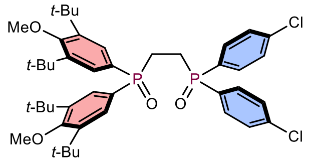

3ec (240.0 mg, 0.3 mmol, 1.0 equiv) was employed as starting materials. Purification of the crude product by silica-gel column chromatography (eluent: DCM/acetone, 98/2 to 7/3) afforded **5ec** (249.4 mg, 0.32 mmol, quantitative yield). White solid; IR (ATR): 2961, 1582, 1408, 1391, 1198, 1172, 1148, 1116, 1090, 1011, 749, 728 cm^{-1}; ^{1}H NMR (400 MHz, $CDCl_3$) *δ*: 7.64 (dd, *J* = 11.0, 8.4 Hz, 4H), 7.48-7.26 (m, 8H), 3.67 (s, 6H), 2.63-2.51 (m, 2H), 2.42-2.33 (m, 2H), 1.36 (s, 36H) ppm; ^{13}C NMR (100 MHz, $CDCl_3$) *δ*: 162.9 (d, *J* = 3.3 Hz), 144.5 (d, *J* = 11.8 Hz), 138.8 (d, *J* = 3.3 Hz), 132.1 (d, *J* = 10.2 Hz), 130.3 (d, *J* = 100.1 Hz), 129.4-129.2 (m, 2C), 125.3 (d, *J* = 102.9 Hz), 64.4, 35.9, 31.8, 22.4-21.7 (m, 2C) ppm; ^{31}P NMR (162 MHz, $CDCl_3$) *δ*: 34.8 (d, *J* = 52.0 Hz), 32.5 (d, *J* = 52.0 Hz) ppm; HRMS (ESI) *m*/*z* calcd. for $C_{44}H_{58}Cl_2O_4P_2Na^+$ $[M+Na]^+$: 805.3080, found: 805.3082.

Bis(4-chlorophenyl)(2-(Bis(4-trifluoromethylphenyl)phosphinyl)ethyl)phosphine oxide (**5cb**)

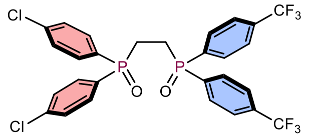

3cb (130.3 mg, 0.2 mmol, 1.0 equiv) was employed as starting materials. Purification of the crude product by silica-gel column chromatography (eluent: DCM/MeOH, 100/0 to 5/1) afforded **5cb** (127.1 mg, 0.20 mmol, quantitative yield). White solid; IR (ATR): 2914, 1583, 1483, 1400, 1327, 1183, 1164, 1123, 1063, 1015, 754, 711 cm^{-1}; ^{1}H NMR (400 MHz, $CDCl_3$) δ: 7.87-7.82 (m, 4H), 7.74-7.72 (m, 4H), 7.61 (dd, J = 10.9, 8.4 Hz, 4H), 7.44 (dd, J = 8.4, 2.1 Hz, 4H), 2.58-2.42 (m, 4H) ppm; ^{13}C NMR (100 MHz, $CDCl_3$) δ: 139.4 (d, J = 2.9 Hz), 136.0-134.0 (m, 2C), 132.2 (d, J = 9.9 Hz), 131.3 (d, J = 9.2 Hz), 130.2-129.2 (m, 2C), 126.2-126.0 (m), 123.4 (q, J = 271.4 Hz), 21.8 (d, J = 2.9 Hz), 21.2 (d, J = 2.9 Hz) ppm; ^{19}F NMR (376 MHz, $CDCl_3$) δ: −63.2 ppm; ^{31}P NMR (162 MHz, $CDCl_3$) δ: 31.8 (d, J = 52.0 Hz), 30.9 (d, J = 52.0 Hz) ppm; HRMS (ESI) *m/z* calcd. for $C_{28}H_{20}Cl_2F_6O_2P_2Na^+$ $[M+Na]^+$: 657.0112, found: 657.0093.

(2-(Bis(4-trifluoromethylphenyl)phosphinyl)ethyl)di(naphthalen-2-yl)phosphine oxide (**5nb**)

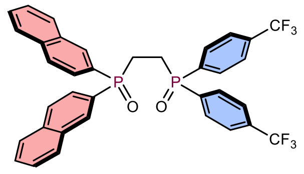

3na (136.5 mg, 0.2 mmol, 1.0 equiv) was employed as starting materials. Purification of the crude product by silica-gel column chromatography (eluent: DCM/MeOH, 100/0 to 5/1) afforded **5nb** (127.7 mg, 0.19 mmol, 96% yield). White solid; IR (ATR): 3053, 1401, 1321, 1193, 1164, 1132, 1103, 1062, 1017, 833, 745, 654 cm^{-1}; ^{1}H NMR (400 MHz, $CDCl_3$) δ: 8.41 (dd, J = 13.3, 1.5 Hz, 2H), 7.91-7.83 (m, 10H), 7.70-7.64 (m, 6H), 7.61-7.52 (m, 4H), 2.77-2.60 (m, 4H) ppm; ^{13}C NMR (100 MHz, $CDCl_3$) δ: 135.7 (d, J = 96.8 Hz), 134.9-133.8 (m, 2C), 133.0 (d, J = 8.7 Hz), 132.6 (d, J = 12.8 Hz), 131.4 (d, J = 9.7 Hz), 129.2-128.1 (m, 3C), 127.5, 127.4, 126.1-125.9 (m, 2C), 125.3 (d, J = 10.6 Hz), 123.4 (d, J = 271.5 Hz), 21.9-21.1 (m, 2C) ppm; ^{19}F NMR (376 MHz, $CDCl_3$) δ: −63.2 ppm; ^{31}P NMR (162 MHz, $CDCl_3$) δ: 33.3 (d, J = 52.2 Hz), 31.5 (d, J = 52.2 Hz) ppm; HRMS (ESI) *m/z* calcd. for $C_{36}H_{26}F_6O_2P_2Na^+$ $[M+Na]^+$: 689.1204, found: 689.1190.

(2-(Bis(4-methylphenyl)phosphinyl)ethyl)bis(4-dimethylaminophenyl)phosphine oxide (**5gi**)

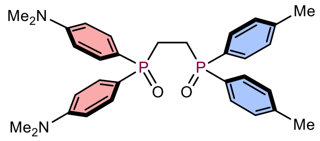

3gi (140.2 mg, 0.25 mmol, 1.0 equiv) was employed as starting materials. Purification of the crude product by silica-gel column chromatography (eluent: DCM/MeOH, 99/1 to 9/1) afforded **5gi** (129.6 mg, 0.24 mmol, 95% yield). White solid; IR (ATR): 2910, 1598, 1517, 1362, 1183, 1166, 1116, 809, 739, 653 cm^{-1}; ^{1}H NMR (400 MHz, $CDCl_3$) δ: 7.58-7.54 (m, 4H), 7.50-7.45 (m, 4H), 7.21 (d, J = 7.7 Hz, 4H), 6.65 (d, J = 8.4 Hz, 4H), 2.96 (s, 12H), 2.47-2.35 (m, 10H) ppm; ^{13}C NMR (100 MHz, $CDCl_3$) δ: 152.2 (d, J = 1.3 Hz), 142.2 (d, J = 2.3 Hz), 132.1 (d, J = 9.2 Hz), 130.8 (d, J = 8.2 Hz), 130.0-128.3 (m, 2C), 118.3-116.6 (m), 111.4 (d, J = 10.7 Hz), 40.0, 22.5-21.8 (m, 2C), 21.5 ppm; ^{31}P NMR (162 MHz, $CDCl_3$) δ: 34.4 (d, J = 52.0 Hz), 34.0 (d, J = 52.0 Hz) ppm; HRMS (ESI) *m/z* calcd. for $C_{32}H_{38}N_2O_2P_2Na^+$ $[M+Na]^+$: 567.2301, found: 567.2296.

1,2-Ethanediylbis(bis(3,5-di-*tert*-butyl-4-methoxyphenyl)phosphine) dioxide (**5ee**)

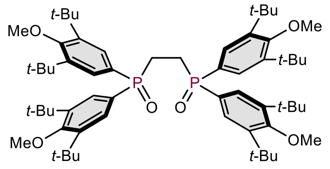

3ee (32.0 mg, 0.032 mmol, 1.0 equiv) was employed as starting materials. Purification of the crude product by silica-gel column chromatography (eluent: hexane/ethyl acetate, 2/1 to 1/1) afforded **5ee** (25.4 mg, 0.025 mmol, 81% yield). White solid; IR (ATR): 2960, 1445, 1406, 1225, 1213, 1149, 1117, 1003, 880, 741 cm^{-1}; ^{1}H NMR (400 MHz, $CDCl_3$) δ: 7.54-7.51 (m, 8H), 3.67 (s, 12H), 2.49 (d, J = 2.1 Hz, 4H), 1.37 (s, 72H) ppm; ^{13}C NMR (100 MHz, $CDCl_3$) δ: 162.9, 144.5-144.4 (m), 129.7-129.6 (m), 126.6-125.2 (m), 64.6, 36.1, 32.0, 23.4-22.2 (m) ppm; ^{31}P NMR (162 MHz, $CDCl_3$) δ: 35.6 ppm; HRMS (ESI) *m*/*z* calcd. for $C_{62}H_{96}O_6P_2Na^+$ $[M+Na]^+$: 1021.6574, found: 1021.6551.

(J) Synthesis of $PhSiH_2$-O-SiH_2Ph

$PhSiH_2$-O-SiH_2Ph was synthesized according to the literature[5].

To a 200 mL round bottom flask was added $CuCl_2$ (5.4 g, 40 mmol), CuI (381 mg, 2 mmol), and Et_2O (27 mL). The reaction mixture was stirred at room temperature for 30 min (750 rpm). Phenylsilane (5.54 mL, 45 mmol, 1.0 equiv) was added dropwise, and after 20 h, the reaction was filtered directly onto ice and washed with Et_2O. The ether and ice were swirled every 5 min for 15 min, until the ice was melted. The organic layers were separated and washed with cold deionized water and then dried with $MgSO_4$. The solvent was evaporated to afford $PhSiH_2$-O-SiH_2Ph as pale yellow oil in 76% yield (1.76g, 7.62 mmol).

(K) Reduction of Dioxide 5[6]

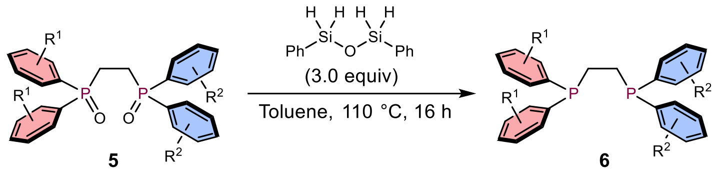

In a Teflon-cock sealed tube were placed 1,3-diphenyl-disiloxane (DPDS, 3.0 equiv) and **5** (1.0 equiv). The sealed tube was evacuated and backfilled with N_2 (3 times) followed by the addition of toluene. The mixture was stirred at 110 °C for 16 h, and then, after cooling to room temperature, the volatiles were removed under reduced pressure. Hexane was added to the crude mixture and the insoluble solid product **6** was collected by filtration with washing with hexane. If the product has high solubility for hexane, the filtrate was condensed to dryness and the mixture was purified by silica-gel column chromatography to afford the product **6**.

(2-(Bis(4-trifluoromethylphenyl)phosphanyl)ethyl)bis(4-dimethylaminophenyl)phosphine (**6gb**)

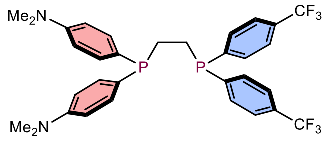

5gb (326.2 mg, 0.5 mmol, 1.0 equiv) was employed as starting materials and 1 mL of toluene was used as a solvent. After the addition of hexane, solid **6gb** was collected (262.8 mg, 0.42 mmol, 85% yield). White solid; IR (ATR): 2887, 1595, 1506, 1318, 1160, 1120, 1100, 1058, 1013, 826, 805, 696 cm^{-1}; ^{1}H NMR (400 MHz, $CDCl_3$) δ: 7.54-7.52 (m, 4H), 7.41-7.38 (m, 4H), 7.20 (dd, J = 8.8, 7.1 Hz, 4H), 6.66- 6.64 (m, 4H), 2.95 (s, 12H), 2.18-2.10 (m, 2H), 2.00-1.93 (m, 2H) ppm; ^{13}C NMR (100 MHz, $CDCl_3$) δ: 150.8, 142.9 (d, J = 17.3 Hz), 133.8 (d, J = 19.7 Hz), 133.2 (d, J = 18.4 Hz), 130.9 (q, J = 32.5 Hz), 128.2-120.0 (m, 3C), 112.4 (d, J = 7.5 Hz), 40.3, 24.6 (dd, J = 13.9, 13.9 Hz), 23.8 (dd, J = 18.0, 14.2 Hz) ppm; ^{19}F NMR

(376 MHz, $CDCl_3$) δ: −62.7 ppm; ^{31}P NMR (162 MHz, $CDCl_3$) δ: −11.8 (d, J = 34.7 Hz), −16.7 (d, J = 34.7 Hz) ppm; HRMS (ESI) m/z calcd. for $C_{32}H_{33}F_6N_2P_2^+$ $[M+H]^+$: 621.2018, found: 621.2006.

(2-(Bis(4-trifluoromethylphenyl)phosphanyl)ethyl)bis(4-methoxyphenyl)phosphine (**6fb**)

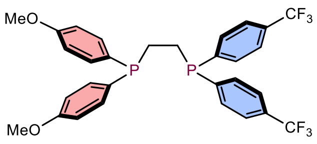

5fb (62.6 mg, 0.1 mmol, 1.0 equiv) was employed as starting materials and 0.2 mL of toluene was used as a solvent. Solidified product **6fb** was obtained and additional purification of the filtrate by silica-gel column chromatography (eluent: hexane/ethyl acetate, 100/0 to 10/1) afforded **6fb** (Total 46.8 mg, 0.079 mmol, 79% yield). White solid; IR (ATR): 2936, 1594, 1497, 1325, 1244, 1165, 1122, 1104, 1059, 1014, 830, 698 cm^{-1}; ^1H NMR (400 MHz, $CDCl_3$) δ: 7.57-7.28 (m, 4H), 7.43-7.39 (m, 4H), 7.28-7.24 (m, 4H), 6.87-6.84 (m, 4H), 3.80 (s, 6H), 2.15-2.06 (m, 2H), 2.04-1.97 (m, 2H) ppm; ^{13}C NMR (100 MHz, $CDCl_3$) δ: 160.4, 142.6 (d, J = 17.0 Hz), 134.2 (d, J = 19.8 Hz), 133.1 (d, J = 18.4 Hz), 131.1 (q, J = 32.4 Hz), 128.8 (d, J = 10.8 Hz), 128.1-120.0 (m, 2C), 114.3 (d, J = 7.6 Hz), 55.3, 24.4 (dd, J = 14.4, 14.4 Hz), 23.7 (dd, J = 17.3, 14.6 Hz) ppm; ^{19}F NMR (376 MHz, $CDCl_3$) δ: −62.7 ppm; ^{31}P NMR (162 MHz, $CDCl_3$) δ: −12.0 (d, J = 35.8 Hz), −15.8 (d, J = 35.8 Hz) ppm; HRMS (ESI) m/z calcd. for $C_{30}H_{27}F_6O_2P_2^+$ $[M+H]^+$: 595.1385, found: 595.1373.

(2-(Bis(4-trifluoromethylphenyl)phosphanyl)ethyl)bis(2-methoxyphenyl)phosphine (**6jb**)

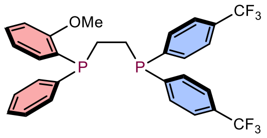

5jb (59.6 mg, 0.1 mmol, 1.0 equiv) was employed as starting materials and 1 mL of toluene was used as a solvent. Purification of the crude product by silica-gel column chromatography (eluent: hexane/ethyl acetate, 15/1) afforded **6jb** (50.0 mg, 0.089 mmol, 89% yield). Colorless liquid; IR (ATR): 2933, 1606, 1431, 1395, 1320, 1239, 1163, 1120, 1058, 1014, 828, 696 cm^{-1}; ^1H NMR (400 MHz, $CDCl_3$) δ: 7.58-7.55 (m, 4H), 7.46-7.26 (m, 10H), 6.92-6.81 (m, 3H), 3.70 (s, 3H), 2.67-1.95 (m, 4H) ppm; ^{13}C NMR (100 MHz, $CDCl_3$) δ: 161.1 (d, J = 12.9 Hz), 142.7 (dd, J = 41.6, 17.1 Hz), 136.5 (d, J = 12.6 Hz), 133.6-132.9 (m, 2C), 132.2 (d, J = 4.4 Hz), 131.3-130.8 (m), 130.4, 129.2, 128.6 (d, J = 7.2 Hz), 128.1-120.0 (m, 2C), 126.1 (d, J = 15.8 Hz), 121.0, 110.4, 55.5, 24.0 (dd, J = 18.8, 14.8 Hz), 21.8 (dd, J = 14.8, 14.8 Hz) ppm; ^{19}F NMR (376 MHz, $CDCl_3$) δ: −62.7 ppm; ^{31}P NMR (162 MHz, $CDCl_3$) δ: −11. 8 (d, J = 34.7 Hz), −21.1 (d, J = 34.7 Hz) ppm; HRMS (ESI) m/z calcd. for $C_{29}H_{25}F_6OP_2^+$ $[M+H]^+$: 565.1279, found: 565.1269.

(2-(Bis(4-trifluoromethylphenyl)phosphanyl)ethyl)bis(3,5-di-*tert*-butyl-4-methoxyphenyl)phosphine (**6eb**)

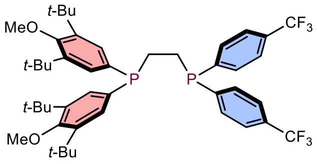

5eb (212.7 mg, 0.25 mmol, 1.0 equiv) was employed as starting materials and 0.5 ml of toluene was used as a solvent. Purification of the crude product by silica-gel column chromatography (eluent: hexane/diethyl ether, 20/1) afforded **6eb** (176.1 mg, 0.22 mmol, 86% yield). White solid; IR (ATR): 2957, 1606, 1409, 1393, 1321, 1224, 1166, 1126, 1059, 1013, 828, 698 cm^{-1}; ^1H NMR (400 MHz, $CDCl_3$) δ: 7.57-7.55 (m, 4H), 7.46-7.42 (m, 4H), 7.18 (d, J = 7.3 Hz, 4H), 3.67 (s, 6H), 2.23-2.17 (m, 2H), 2.03-1.97 (m, 2H), 1.35 (s, 36H) ppm; ^{13}C NMR (100 MHz, $CDCl_3$) δ: 160.4, 143.9 (d, J = 6.2 Hz), 142.8 (d, J = 15.9 Hz), 133.4-133.0 (m, 2C), 131.6-120.0 (m, 4C), 64.4, 36.0, 32.1, 24.8 (dd, J = 13.1, 13.1 Hz), 24.3 (dd, J = 15.0, 12.4 Hz) ppm; ^{19}F NMR (376 MHz, $CDCl_3$) δ: −62.7 ppm; ^{31}P NMR (162 MHz, $CDCl_3$) δ: −11.1 (d, J =

34.7 Hz), −11.5 (d, $J = 34.7$ Hz) ppm; HRMS (ESI) *m/z* calcd. for $C_{46}H_{59}F_6O_2P_2^+$ $[M+H]^+$: 819.3889, found: 819.3874.

(2-(Bis(4-chlorophenyl)phosphanyl)ethyl)bis(3,5-di-*tert*-butyl-4-methoxyphenyl)phosphine (**6ec**)

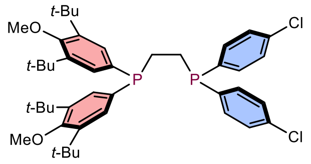

5ec (78.2 mg, 0.1 mmol, 1.0 equiv) was employed as starting materials and 0.2 mL of toluene was used as a solvent. Purification of the crude product by silica-gel column chromatography (eluent: hexane/ethyl acetate, 100/0 to 9/1) afforded **6ec** (66.7 mg, 0.089 mmol, 89% yield). White solid; IR (ATR): 2956, 1480, 1404, 1390, 1223, 1086, 1011, 812, 739, 479 cm^{-1}; ^1H NMR (400 MHz, $CDCl_3$) δ: 7.28-7.22 (m, 8H), 7.17 (d, $J = 7.7$ Hz, 4H), 3.66 (s, 6H), 2.13-2.07 (m, 2H), 2.00-1.94 (m, 2H), 1.34 (s, 36H) ppm; ^{13}C NMR (100 MHz, $CDCl_3$) δ: 160.3, 143.7 (d, $J = 6.8$ Hz), 136.6 (d, $J = 15.2$ Hz), 135.2, 134.1 (d, $J = 18.9$ Hz), 131.2-130.9 (m, 2C), 128.9 (d, $J = 6.8$ Hz), 64.4, 36.0, 32.1, 24.9-24.6 (m, 2C) ppm; ^{31}P NMR (162 MHz, $CDCl_3$) δ: −11.3 (d, $J = 34.8$ Hz), −13.2 (d, $J = 34.8$ Hz) ppm; HRMS (ESI) *m/z* calcd. for $C_{44}H_{59}Cl_2O_2P_2^+$ $[M+H]^+$: 715.3362, found: 715.3361.

Bis(4-chlorophenyl)(2-(Bis(4-trifluoromethylphenyl)phosphanyl)ethyl)phosphine (**6cb**)

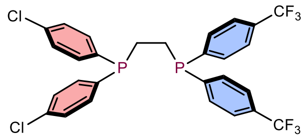

5cb (63.5 mg, 0.1 mmol, 1.0 equiv) was employed as starting materials and 0.2 mL of toluene was used as a solvent. After the addition of hexane, solid **6cb** was collected (46.9 mg, 0.078 mmol, 78% yield). White solid; IR (ATR): 3051, 1607, 1478, 1396, 1324, 1164, 1118, 1103, 1059, 1012, 816, 698 cm^{-1}; ^1H NMR (400 MHz, $CDCl_3$) δ: 7.58-7.56 (m, 4H), 7.42-7.38 (m, 4H), 7.30-7.27 (m, 4H), 7.24-7.20 (m, 4H), 2.13-1.98 (m, 4H) ppm; ^{13}C NMR (100 MHz, $CDCl_3$) δ: 142.2 (d, $J = 17.1$ Hz), 135.9 (d, $J = 14.7$ Hz), 135.6, 134.1 (d, $J = 19.2$ Hz), 133.1 (d, $J = 18.6$ Hz), 131.3 (q, $J = 32.5$ Hz), 129.1 (d, $J = 6.8$ Hz), 125.6-125.5 (m), 124.0 (q, $J = 270.9$ Hz), 24.1-23.4 (m, 2C) ppm; ^{19}F NMR (376 MHz, $CDCl_3$) δ: −62.7 ppm; ^{31}P NMR (162 MHz, $CDCl_3$) δ: −12.4 (d, $J = 36.8$ Hz), −14.4 (d, $J = 36.8$ Hz) ppm; HRMS (ESI) *m/z* calcd. for $C_{28}H_{21}Cl_2F_6P_2^+$ $[M+H]^+$: 603.0394, found: 603.0386.

(2-(Bis(4-trifluoromethylphenyl)phosphanyl)ethyl)di(naphthalen-2-yl)phosphine (**6nb**)

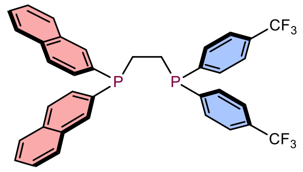

5nb (66.6 mg, 0.1 mmol, 1.0 equiv) was employed as starting materials and 1 mL of toluene was used as a solvent. After the addition of hexane, solid **6nb** was collected (35.8 mg, 0.056 mmol, 56% yield). White solid; 3048, 1607, 1396, 1325, 1164, 1122, 1102, 1060, 1015, 815, 743, 698 cm^{-1}; ^1H NMR (400 MHz, $CDCl_3$) δ: 7.96-7.93 (m, 2H), 7.84-7.75 (m, 6H), 7.54-7.49 (m, 8H), 7.40-7.32 (m, 6H), 2.33-2.17 (m, 4H) ppm; ^{13}C NMR (100 MHz, $CDCl_3$) δ: 142.4 (d, $J = 16.9$ Hz), 135.1 (d, $J = 13.2$ Hz), 133.6-133.0 (m, 4C), 131.1 (q, $J = 32.6$ Hz), 128.9 (d, $J = 14.0$ Hz), 128.3 (d, $J = 6.0$ Hz), 128.1, 127.9-120.0 (m, 5C), 24.0-23.6 (m, 2C) ppm; ^{19}F NMR (376 MHz, $CDCl_3$) δ: −62.7 ppm; ^{31}P NMR (162 MHz, $CDCl_3$) δ: −11.2 (d, $J = 34.7$ Hz), −12.0 (d, $J = 34.7$ Hz) ppm; HRMS (ESI) *m/z* calcd. for $C_{36}H_{27}F_6P_2^+$ $[M+H]^+$: 635.1487, found: 635.1476.

(2-(Bis(4-methylphenyl)phosphanyl)ethyl)bis(4-dimethylaminophenyl)phosphine (**6gi**)

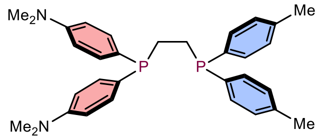

5gi (54.4 mg, 0.1 mmol, 1.0 equiv) was employed as starting materials and 1 mL of toluene was used as a solvent. Purification of the crude product by silica-gel column chromatography (eluent: DCM/acetone, 50/2) afforded **6ee** (44.3 mg, 0.0864 mmol, 86% yield). White solid; IR (ATR): 2916, 1594, 1507, 1351, 1197, 1095, 803, 684, 511, 498 cm^{-1}; ^{1}H NMR (400 MHz, $CDCl_3$) δ: 7.26-7.22 (m, 8H), 7.10 (d, J = 7.8 Hz, 4H), 6.66 (d, J = 7.9 Hz, 4H), 2.94 (s, 12H), 2.33 (s, 6H), 2.10-1.96 (m, 4H) ppm; ^{13}C NMR (100 MHz, $CDCl_3$) δ: 150.7, 138.4, 135.2 (d, J = 12.2 Hz), 133.9 (d, J = 19.4 Hz), 132.8 (d, J = 18.5 Hz), 129.3 (d, J = 6.8 Hz), 124.1 (d, J = 7.9 Hz), 112.4 (d, J = 7.5 Hz), 40.4, 24.7-24.0 (m, 2C), 21.4 ppm; ^{31}P NMR (162 MHz, $CDCl_3$) δ: −13.7 (d, J = 32.7 Hz), −16.4 (d, J = 32.7 Hz) ppm; HRMS (ESI) m/z calcd. for $C_{32}H_{39}N_2P_2^+$ $[M+H]^+$: 513.2583, found: 513.2579.

1,2-Bis(bis(3,5-di-*tert*-butyl-4-methoxyphenyl)phosphino)ethane (**6ee**)

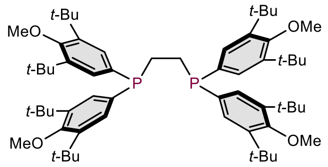

5ee (44.8 mg, 0.0448 mmol, 1.0 equiv) was employed as starting materials and 0.1 mL of toluene was used as a solvent. Purification of the crude product by silica-gel column chromatography (eluent: DCM) afforded **6ee** (41.1 mg, 0.0425 mmol, 95% yield). White solid; 2956, 1408, 1391, 1224, 1114, 1008, 835, 737, 696, 604 cm^{-1}; ^{1}H NMR (400 MHz, $CDCl_3$) δ: 7.25 (d, J = 3.9 Hz, 8H), 3.67 (s, 12H), 2.12-2.10 (m, 4H), 1.36 (s, 72H) ppm; ^{13}C NMR (100 MHz, $CDCl_3$) δ: 160.2, 143.6-143.5 (m), 133.4-131.1 (m, 2C), 64.3, 36.0, 32.2, 25.5 (d, J = 2.5 Hz) ppm; ^{31}P NMR (162 MHz, $CDCl_3$) δ: −11.6 ppm; HRMS (ESI) m/z calcd. for $C_{62}H_{97}O_4P_2^+$ $[M+H]^+$: 967.6857, found: 967.6847.

(L) Synthesis of Unsymmetric DPPE Oxide 7gb and Its Reduction

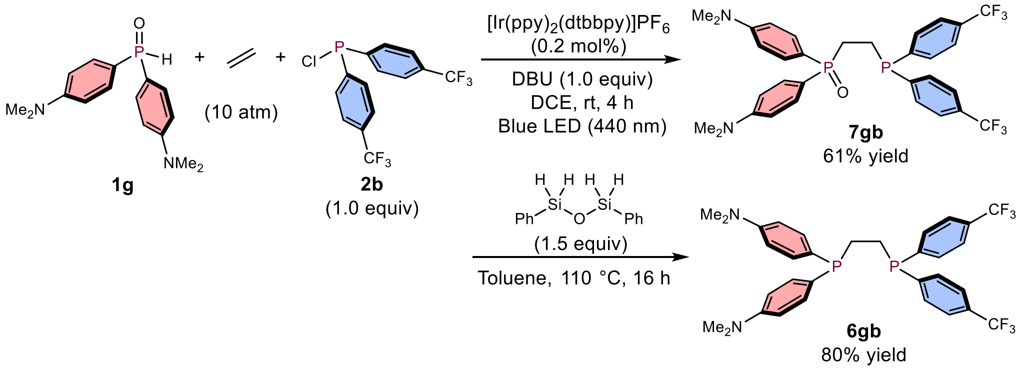

In an oven-dried 10 mL pressure-resistant glass tube were placed phosphine oxide **1g** (0.5 mmol, 1.0 equiv) and $[Ir(ppy)_2(dtbbpy)]PF_6$ (0.9 mg, 0.001 mmol, 0.2 mol%). After the addition of DCE (1.5 mL) under nitrogen, chlorophosphine **2b** (0.5 mmol, 1.0 equiv) and DBU (74.8 µL,0.5 mmol, 1.0 equiv) were added. Then, the tube was placed into autoclave which consists of a polycarbonate cylinder, and ethylene gas was pressurised to 10 atm. After the resulting mixture was stirred at room temperature for 4h under the irradiation of blue LED (45W PR160L-440 nm Kessil light × 2), the solvent was evaporated to give the crude mixture. The crude product was purified by silica-gel column chromatography to afford the product **7gb** (193.9 mg, 0.30 mmol, 61%).

In a Teflon-cock sealed tube were placed 1,3-diphenyl-disiloxane (DPDS, 34,6 mg, 0.15 mmol, 1.5 equiv) and **7gb** (63.7 mg, 0.1 mmol, 1.0 equiv). The sealed tube was evacuated and backfilled with N_2 (3 times)

followed by the addition of toluene (0.2 mL). The mixture was stirred at 110 °C for 16 h, and then, after cooling to room temperature, the volatiles were removed under reduced pressure. Hexane was added to the crude mixture and the insoluble solid product **6gb** was collected by filtration with washing with hexane (49.4mg, 0.08 mmol, 80%).

(2-(Bis(4-trifluoromethylphenyl)phosphanyl)ethyl)bis(4-dimethylaminophenyl)phosphine oxide (**7gb**)

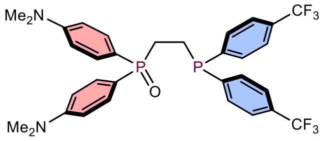

White solid; IR (ATR): 2889, 1598, 1322, 1161, 1117, 1060, 1015, 814, 723, 502 cm^{-1}; ^{1}H NMR (400 MHz, $CDCl_3$) δ: 7.54 (d, J = 7.9 Hz, 4H), 7.45-7.38 (m, 8H), 6.65 (dd, J = 8.9, 2.3 Hz, 4H), 2.98 (s, 12H), 2.35-2.29 (m, 2H), 2.17-2.08 (m, 2H) ppm; ^{13}C NMR (100 MHz, $CDCl_3$) δ: 152.4 (d, J = 2.4 Hz), 142.2 (d, J = 16.9 Hz), 133.2 (d, J = 18.8 Hz), 132.3 (d, J = 6.7 Hz), 131.1 (q, J = 32.6 Hz), 125.5-125.4 (m), 124.0 (d, J = 270.9 Hz), 117.4 (d, J = 110.1 Hz), 111.5 (d, J = 12.4 Hz), 40.2-40.0 (m), 26.3 (dd, J = 70.3, 15.4 Hz), 19.3 (d, J = 14.7 Hz) ppm; ^{19}F NMR (376 MHz, $CDCl_3$) δ: −62.7 ppm; ^{31}P NMR (162 MHz, $CDCl_3$) δ: 34.3 (d, J = 47.7 Hz), −11.3 (d, J = 47.7 Hz) ppm; HRMS (ESI) *m/z* calcd. for $C_{32}H_{32}F_6N_2OP_2Na^+$ $[M+Na]^+$: 659.1786, found: 659.1764.

(M) Synthesis of Transition-Metal Complexes

Synthesis of $NiCl_2$(6gb)

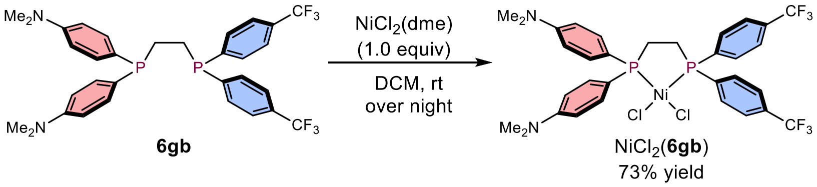

In an oven-dried test tube were placed **6gb** (62.1mg, 0.1 mmol, 1.0 equiv) and $NiCl_2$(dme) (22.0 mg, 0.1 mmol, 1.0 equiv) in DCM (2 mL). The resulting mixture was stirred over night at room temperature. The solvent was removed under reduced pressure and small amount of hexane was added. The resulting slurry was filtrated to obtain the product $NiCl_2$(**6gb**) (54.7 mg, 0.073 mmol, 73%).

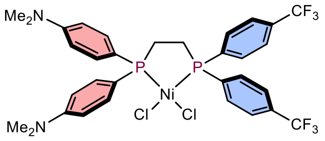

Red solid; IR (ATR): 1594, 1320, 1168, 1124, 1103, 1060, 1014, 810, 706, 543 cm^{-1}; ^{1}H NMR (400 MHz, $CDCl_3$) δ: 8.14-8.09 (m, 4H), 7.79-7.70 (m, 8H), 6.72-6.70 (m, 4H), 3.02 (s, 12H), 2.14-2.05 (m, 4H) ppm; ^{13}C NMR (100 MHz, $CDCl_3$) δ: 152.4, 134.8 (d, J = 9.7 Hz), 134.3-131.4 (m, 3C), 125.9-125.8 (m), 123.6 (q, J = 271.5 Hz), 112.5-111.5 (m, 2C), 40.1, 28.3 (dd, J = 32.4, 18.4 Hz), 26.4 (dd, J = 29.4, 15.4 Hz) ppm; ^{19}F NMR (376 MHz, $CDCl_3$) δ: −63.1 ppm; ^{31}P NMR (162 MHz, $CDCl_3$) δ: 56.6 (d, J = 69.4 Hz), 55.9 (d, J = 69.4 Hz) ppm; HRMS (ESI) *m/z* calcd. for $C_{32}H_{32}ClF_6N_2NiP_2^+$ $[M-Cl]^+$: 713.0981, found: 713.0997.

Synthesis of $PdCl_2$(6gb)

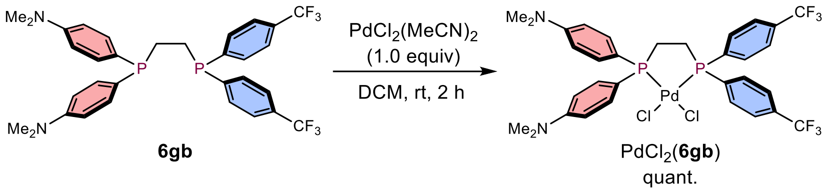

In an oven-dried test tube were placed **6gb** (31.1mg, 0.05 mmol, 1.0 equiv) and $PdCl_2(MeCN)_2$ (13 mg, 0.05 mmol, 1.0 equiv) in DCM (1 mL). The resulting mixture was stirred for 2 h at room temperature. The solvent was removed under reduced pressure and small amount of hexane was added. The resulting slurry was filtrated to obtain the product $PdCl_2$(**6gb**) (39.8 mg, 0.05 mmol, quantitative yield).

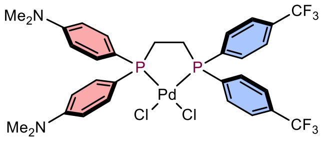

Yellow solid; IR (ATR): 1592, 1320, 1127, 1104, 1061, 1014, 813, 708, 698, 545 cm^{-1}; ^{1}H NMR (400 MHz, CD_2Cl_2) δ: 8.04 (dd, J = 11.8, 8.0 Hz, 4H), 7.76 (dd, J = 8.0, 1.6 Hz, 4H), 7.61 (dd, J = 12.0, 8.8 Hz, 4H), 6.73 (dd, J = 8.8, 2.2 Hz, 4H), 3.02 (s, 12H), 2.51-2.47 (m, 2H), 2.42-2.34 (m, 2H) ppm; ^{13}C NMR (100 MHz, CD_2Cl_2) δ: 152.9, 135.0 (d, J = 12.1 Hz), 134.7-132.9 (m, 3C), 126.2-126.1 (m), 123.9 (q, J = 271.4 Hz), 112.2-111.6 (m, 2C), 40.2, 30.0 (dd, J = 36.7, 16.8 Hz), 27.3 (dd, J = 33.4, 12.6 Hz) ppm; ^{19}F NMR (376 MHz, CD_2Cl_2) δ: −65.4 ppm; ^{31}P NMR (162 MHz, CD_2Cl_2) δ: 62.3 (d, J = 10.8 Hz), 61.1 (d, J = 10.8 Hz) ppm; HRMS (ESI) *m/z* calcd. for $C_{32}H_{32}ClF_6N_2P_2Pd^+$ $[M-Cl]^+$: 757.0684, found: 757.0690.

Synthesis of $PtCl_2$(6gb)

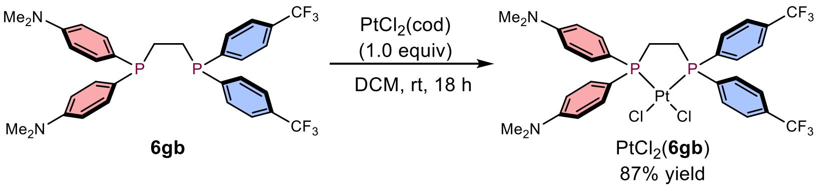

In an oven-dried test tube were placed **6gb** (62.1mg, 0.1 mmol, 1.0 equiv) and $PtCl_2$(cod) (37.4 mg, 0.1 mmol, 1.0 equiv) in DCM (1 mL). The resulting mixture was stirred for 18 h at room temperature. The solvent was removed under reduced pressure and diethyl ether was added. The resulting slurry was filtrated and washed with ethanol to obtain the product $PtCl_2$(**6gb**) (77.4 mg, 0.087 mmol, 87%).

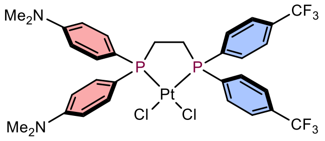

White solid; IR (ATR): 2906, 1594, 1319, 1130, 1106, 1061, 1014, 811, 699, 500 cm^{-1}; ^{1}H NMR (400 MHz, $CDCl_3$) δ: 8.03 (dd, J = 11.9, 8.0 Hz, 4H), 7.66-7.58 (m, 8H), 6.70 (br, 4H), 2.97 (s, 12H), 2.46-2.22 (m, 4H) ppm; ^{13}C NMR (100 MHz, $CDCl_3$) δ: 152.1-151.8 (m), 134.7 (d, J = 12.1 Hz), 134.2-133.1 (m, 3C), 132.0 (d, J = 60.2 Hz), 127.9-127.4 (m), 123.5 (q, J = 271.7 Hz), 112.5-112.1 (m), 40.4, 29.7-29.2 (m), 27.8-27.3 (m) ppm; ^{19}F NMR (376 MHz, $CDCl_3$) δ: −63.1 ppm; ^{31}P NMR (162 MHz, $CDCl_3$) δ: 40.9 (s, J_{Pt-P} = 3773 Hz), 38.8 (s, J_{Pt-P} = 3489 Hz) ppm; HRMS (ESI) *m/z* calcd. for $C_{32}H_{32}ClF_6N_2P_2Pt^+$ $[M-Cl]^+$: 851.1276, found: 851.1268.

Synthesis of Au_2Cl_2(6gb)

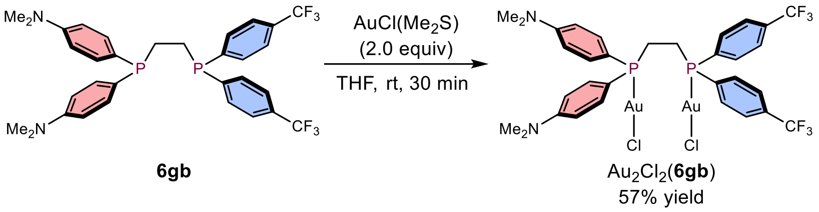

In an oven-dried test tube were placed **6gb** (62.1mg, 0.1 mmol, 1.0 equiv) and AuCl(Me_2S) (58.9 mg, 0.2 mmol, 2.0 equiv) in THF (3 mL). The resulting mixture was stirred for 30 min at room temperature. The solvent was removed under reduced pressure and purification of the crude product by silica-gel column chromatography (eluent: DCM) afforded Au_2Cl_2(**6gb**) (61.7 mg, 0.057 mmol, 57%).

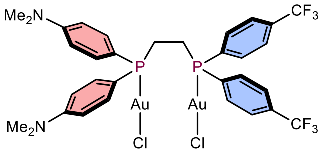

White solid; IR (ATR): 2895, 1593, 1513, 1322, 1104, 1061, 813, 712, 702, 535 cm^{-1}; ^{1}H NMR (400 MHz, $CDCl_3$) δ: 7.80-7.72 (m, 8H), 7.44 (dd, J = 12.5, 8.9 Hz, 4H), 6.66 (dd, J = 9.0, 2.1 Hz, 4H), 3.00 (s, 12H), 2.76-2.67 (m, 2H), 2.54-2.45 (m, 2H) ppm; ^{13}C NMR (100 MHz, $CDCl_3$) δ: 152.6, 135.1-131.7 (m, 3C), 132.0 (d, J = 57.4 Hz), 126.7-126.5 (m), 123.2 (q, J = 271.6 Hz), 112.4-111.5 (m, 2C), 40.1, 25.3 (dd, J = 37.2, 4.3 Hz), 23.7 (dd, J = 35.7, 5.7 Hz) ppm; ^{19}F NMR (376 MHz, $CDCl_3$) δ: −63.2 ppm; ^{31}P NMR (162 MHz, $CDCl_3$) δ: 32.4 (d, J = 60.7 Hz), 28.4 (d, J = 60.7 Hz) ppm; HRMS (ESI) m/z calcd. for $C_{32}H_{32}Au_2Cl_2F_6N_2P_2Na^+$ $[M+Na]^+$: 1107.0554, found: 1107.0545.

(N) NMR studies

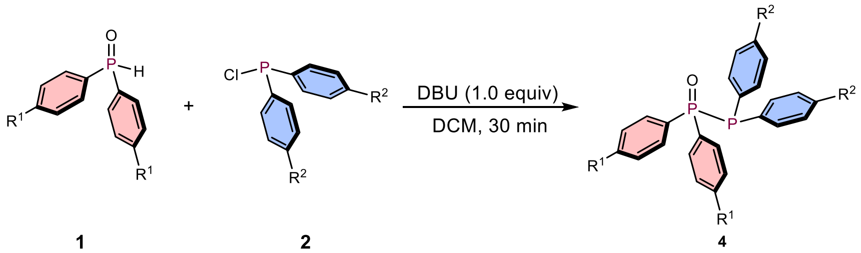

In an oven-dried test tube were placed phosphine oxide **1** (**1a**: 20.2 mg or **1g**: 28.8 mg, 0.1 mmol, 1.0 equiv). After the addition of DCM (0.3 mL) under nitrogen, chlorophosphine **2** (**2a**: 18.0 µL or **2b**: 25.1 µL, 0.1 mmol, 1.0 equiv) and DBU (14.9 µL, 0.1 mmol, 1.0 equiv) were added. After the resulting mixture was stirred at room temperature for 30 min, the solvent was evaporated, and $CDCl_3$ was then added to measure ^{31}P NMR with $P(OPh)_3$ as an internal standard.

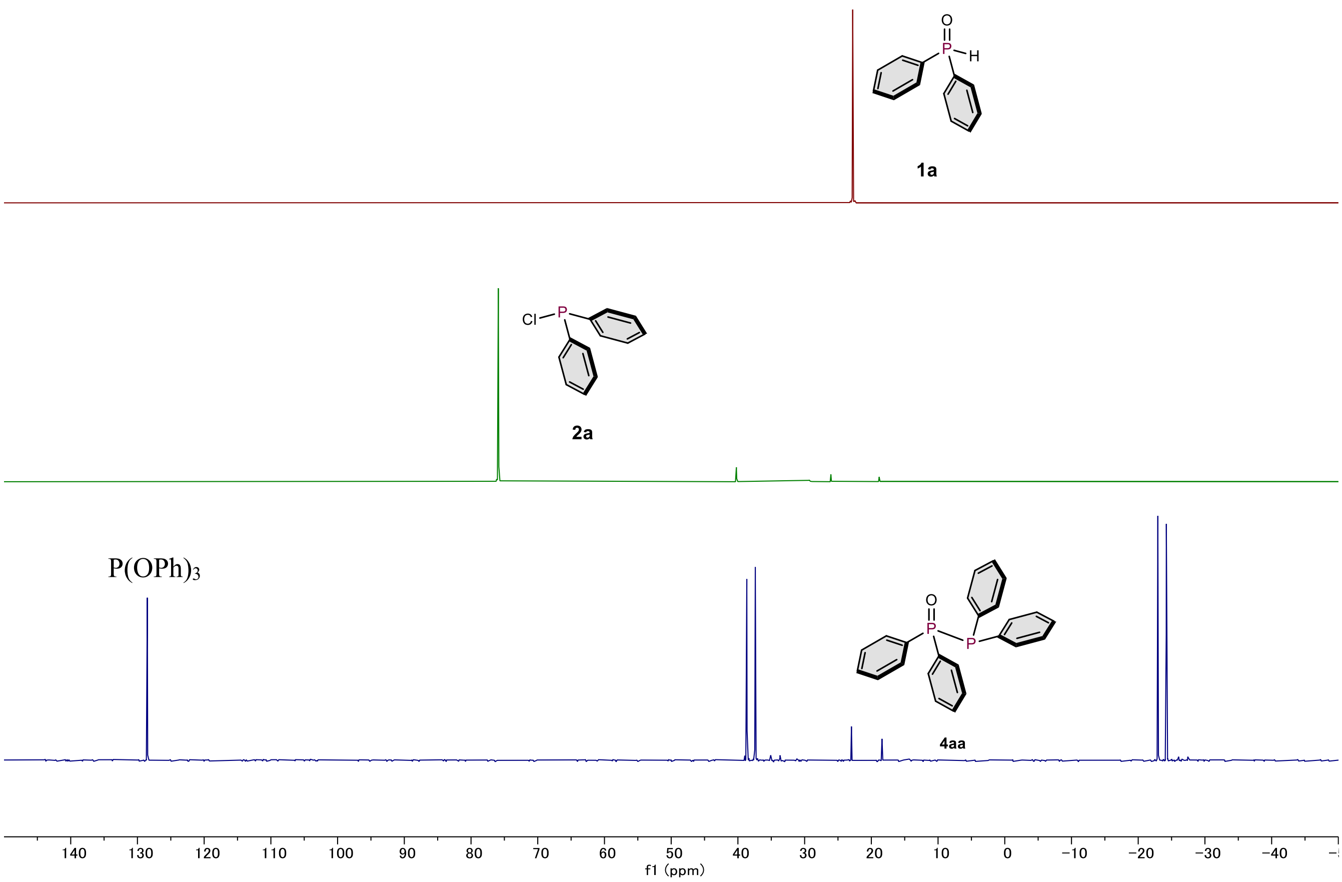

Supplementary Figure 5. ^{31}P NMR spectra (162 MHz, Red: **1a**, Green: **2a**, Blue: **4aa**) in $CDCl_3$

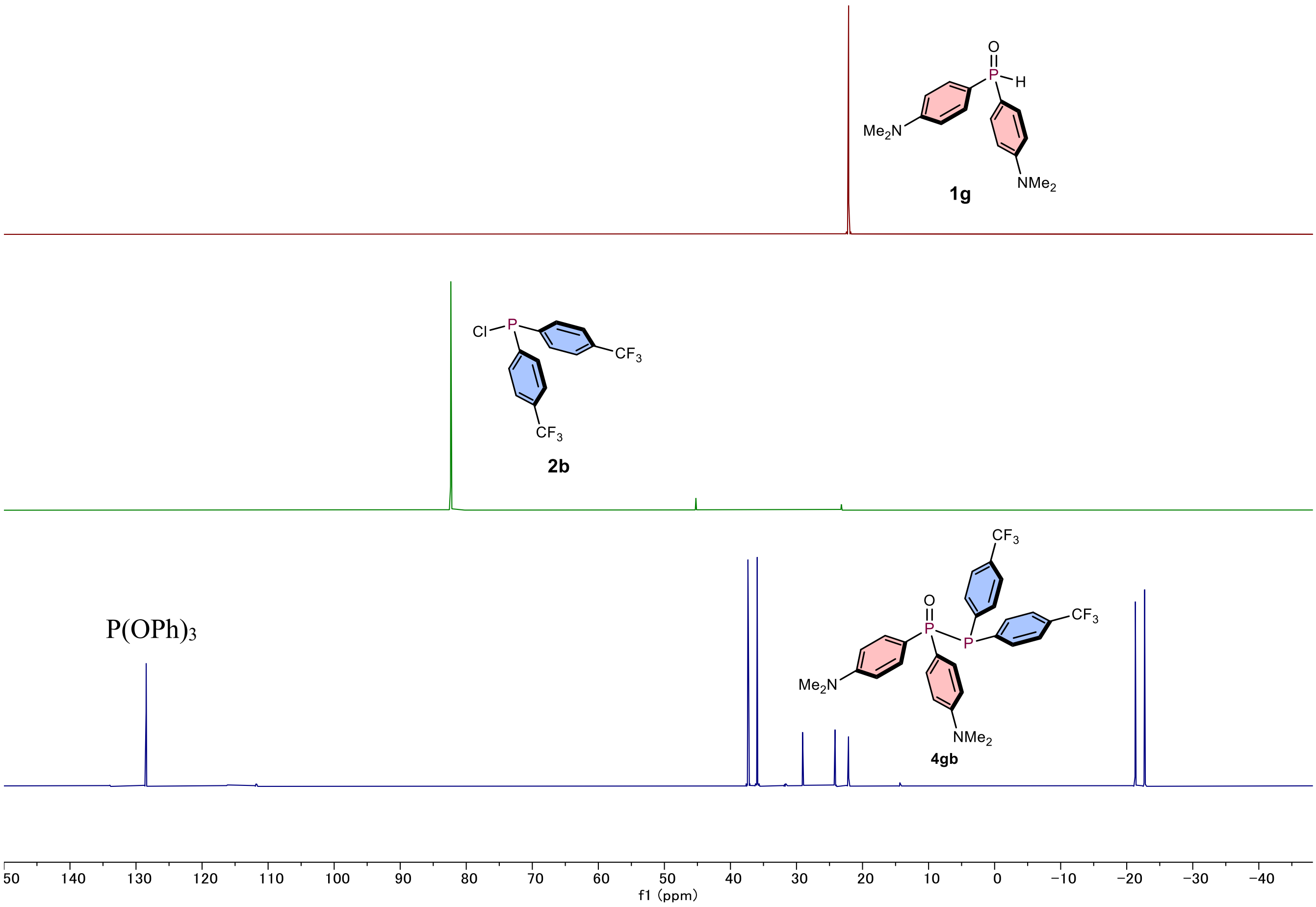

Supplementary Figure 6. ^{31}P NMR spectra (162 MHz, Red: **1g**, Green: **2b**, Blue: **4ga**) in $CDCl_3$

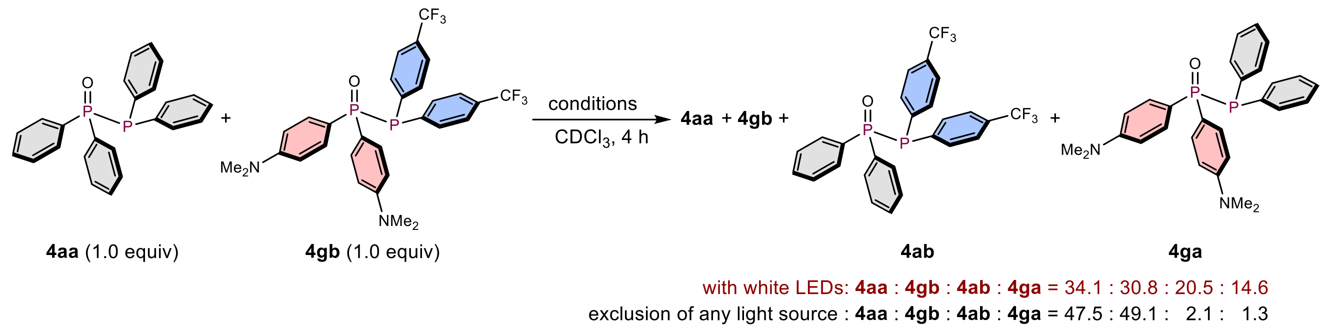

Supplementary Figure 7. Scrambling experiments.

(O) Crystal structures

Supplementary Table 1. The detail of crystal structure of $NiCl_2$(**6gb**)

Compound	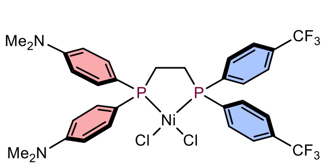 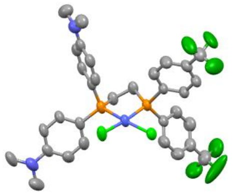
CCDC Name	CCDC 2152051
Empirical Formula	$C_{32}H_{32}Cl_2F_6N_2NiP_2$
Formula Weight	750.14
Crystal System	orthorhombic
Crystal Size / mm	0.2 × 0.15 × 0.04
a / Å	8.87800(10)
b / Å	25.6005(4)
c / Å	29.3762(3)
α / °	90
β / °	90
γ / °	90
V / Å^3	6676.66(15)
Space Group	P b c a
Z value	8
D_{calc} / g cm^{-3}	1.493
Temperature / K	123(2)
$2\theta_{max}$ / °	74.5410
μ (CuK_α) / mm^{-1}	3.742
No. of Reflections Measured	6118
No. of Observations (All reflections)	96902
Residuals: R_1 (I > 2.00σ(I))	0.0834
Residuals: wR_2 (All reflections)	0.2189
Goodness of Fit Indicator (*GOF*)	1.208
Maximum peak in Final Diff. Map / Å^3	1.220
Minimum peak in Final Diff. Map / Å^3	-0.535

Supplementary Table 2. The detail of crystal structure of $PdCl_2$(**6gb**)

Compound	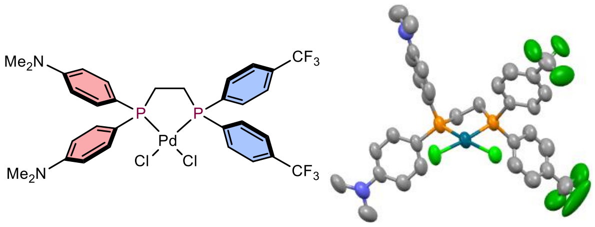
CCDC Name	CCDC 2152052
Empirical Formula	$C_{32}H_{32}Cl_2F_6N_2P_2Pd$
Formula Weight	797.83
Crystal System	orthorhombic
Crystal Size / mm	0.2 × 0.15 × 0.04
a / Å	8.96760(10)
b / Å	25.3443(5)
c / Å	29.6064(3)
α / °	90
β / °	90
γ / °	90
V / Å^3	6728.87(17)
Space Group	P b c a
Z value	8
D_{calc} / g cm^{-3}	1.575
Temperature / K	123(2)
$2\theta_{max}$ / °	68.500
μ (CuK$_\alpha$) / mm^{-1}	7.332
No. of Reflections Measured	6041
No. of Observations (All reflections)	52916
Residuals: R_1 (I > 2.00σ(I))	0.0913
Residuals: wR_2 (All reflections)	0.2636
Goodness of Fit Indicator (*GOF*)	1.030
Maximum peak in Final Diff. Map / Å^3	2.362
Minimum peak in Final Diff. Map / Å^3	-1.148

(P) Measurement of Photophysical Properties

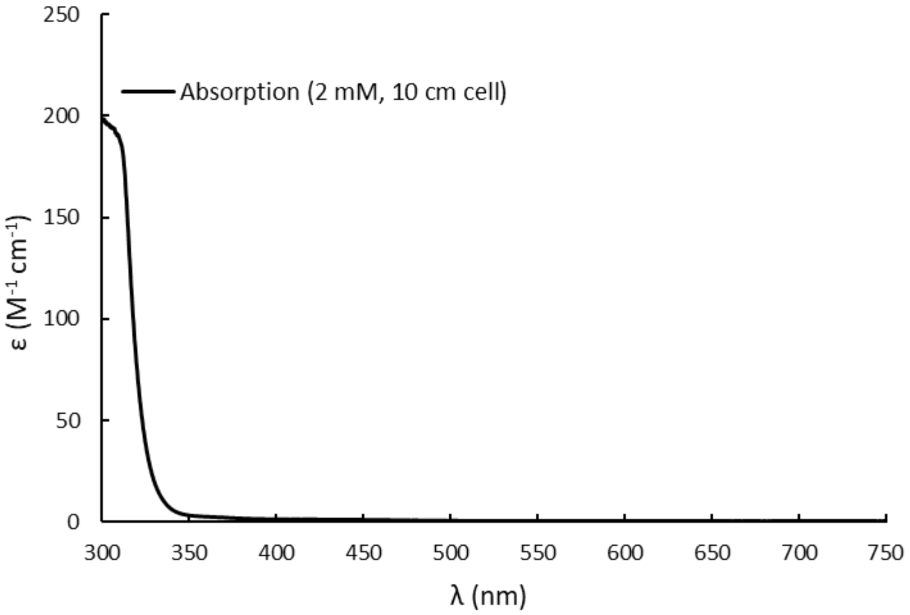

Supplementary Figure 8. Absorption spectrum of diphosphine **4aa** in DCM (2.0 mM) by 10 cm length quartz cell.

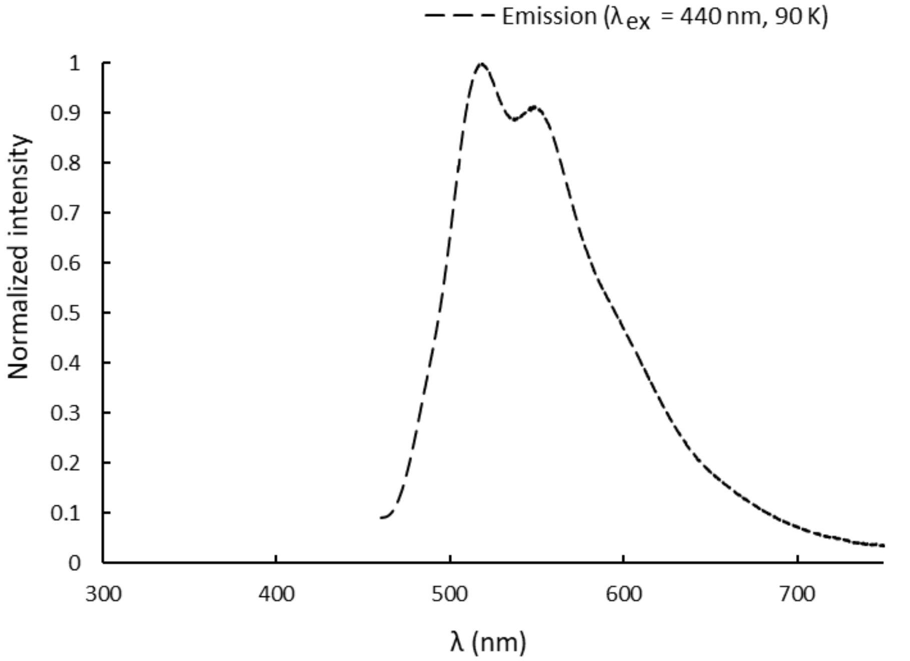

Supplementary Figure 9. Emission spectrum of diphosphine **4aa** in DCM (0.33 M) by 1 cm length quartz cell at 90 K

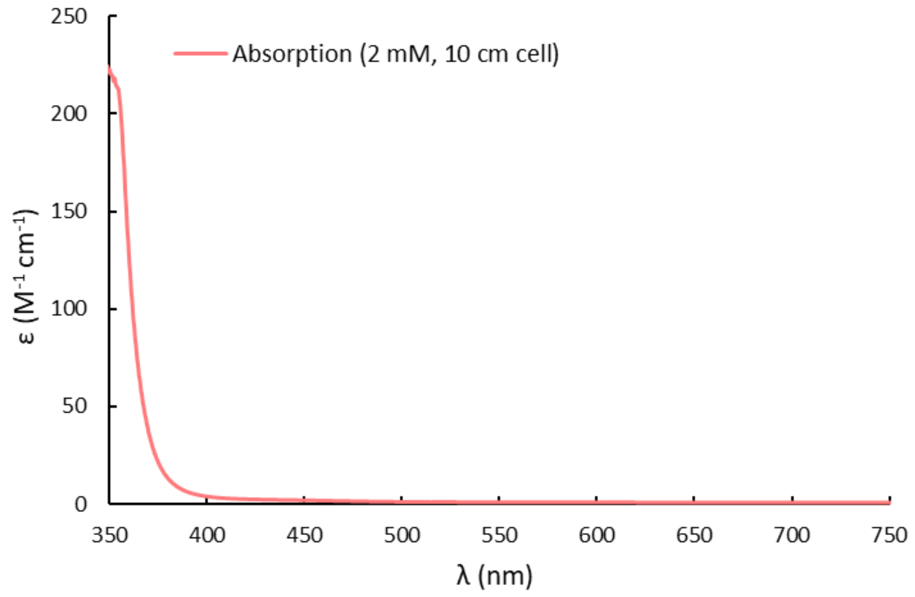

Supplementary Figure 10. Absorption spectrum of diphosphine **4gb** in DCM (2.0 mM) by 10 cm length quartz cell

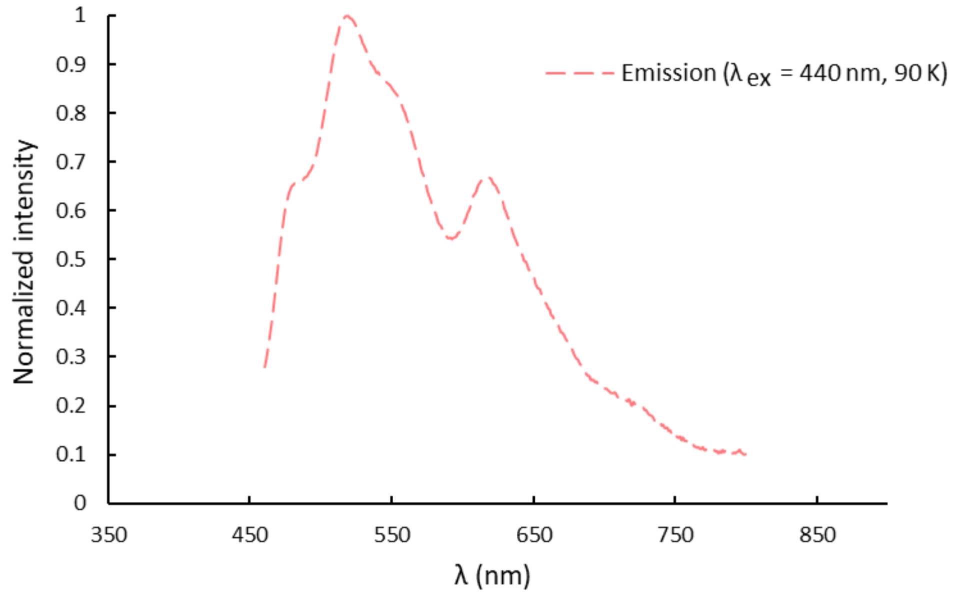

Supplementary Figure 11. Emission spectrum of diphosphine **4gb** in DCM (0.33 M) by 1 cm length quartz cell at 90 K

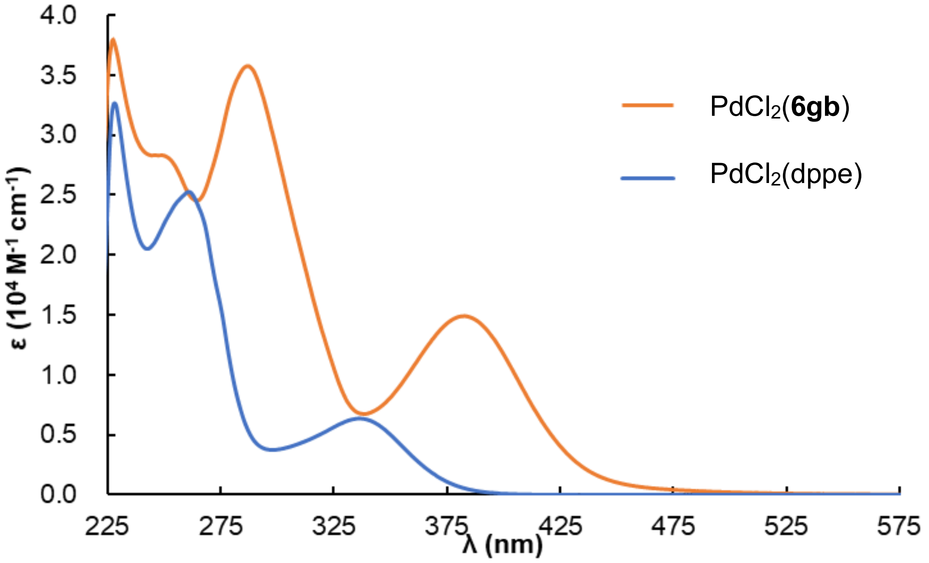

Supplementary Figure 12. Absorption spectra of Pd complexes in DCM by 1 cm length quartz cell. ($PdCl_2$(**6gb**): 5 $\times 10^{-5}$ M, $PdCl_2$(dppe): 6 $\times 10^{-5}$ M)

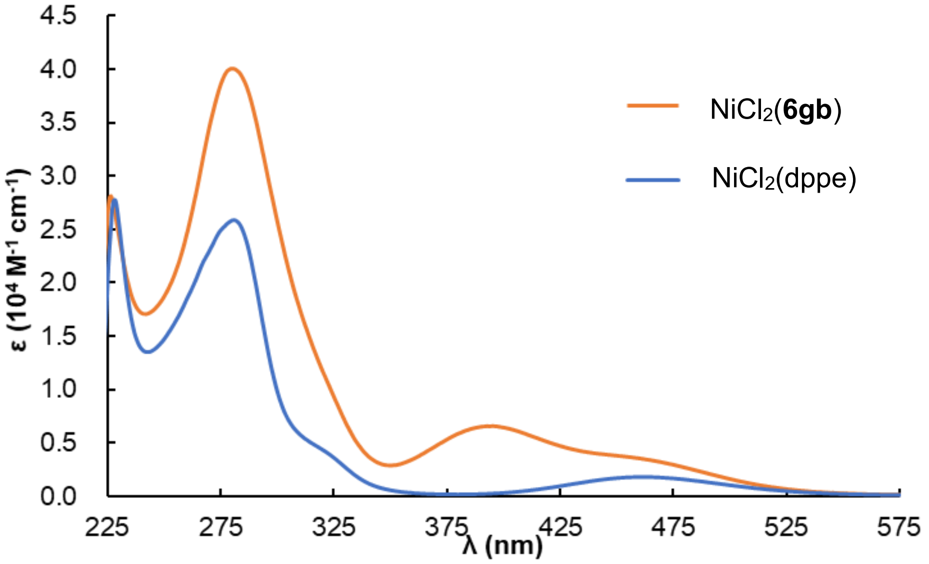

Supplementary Figure 13. Absorption spectra of Ni complexes in DCM by 1 cm length quartz cell. ($NiCl_2$(**6gb**): 3 $\times 10^{-5}$ M, $NiCl_2$(dppe): 8 $\times 10^{-5}$ M)

Supplementary References

1. Liu, W., Sahoo, B., Spannenberg, A., Junge, K. & Beller, M. Tailored cobalt-catalysts for reductive alkylation of anilines with carboxylic acids under mild conditions. *Angew. Chem. Int. Ed.* **57**, 11673–11677 (2018).
2. Imamoto, T., Kikuchi, S. I., Miura, T. & Wada, Y. Stereospecific reduction of phosphine oxides to phosphines by the use of a methylation reagent and lithium aluminum hydride. *Org. Lett.* **3**, 87–90 (2001).
3. Sato, Y., Kawaguchi, S.-i., Nomoto, A. & Ogawa, A. Highly selective phosphinylphosphination of alkenes with tetraphenyldiphosphine monoxide. *Angew. Chem. Int. Ed.* **55**, 9700−9703 (2016).
4. Yue, W.–J., Xiao, J.-Z., Zhang, S. & Yin, L. Rapid synthesis of chiral 1,2-bisphosphine derivatives through copper(I)-catalyzed asymmetric conjugate hydrophosphination. *Angew. Chem. Int. Ed.* **59**, 7057−7062 (2020).
5. Buonomo, J. A., Eiden, C. G. & Aldrich, C. C. Scalable synthesis of hydrido-disiloxanes from silanes: A one-pot preparation of 1,3-diphenyldisiloxane from phenylsilane. *Synthesis* **50**, 278–281 (2018).
6. Buonomo, J. A., Eiden, C. G. & Aldrich, C. C. Chemoselective reduction of phosphine oxides by 1,3-diphenyl-disiloxane. *Chem. Eur. J.* **23**, 14434−14438 (2017).

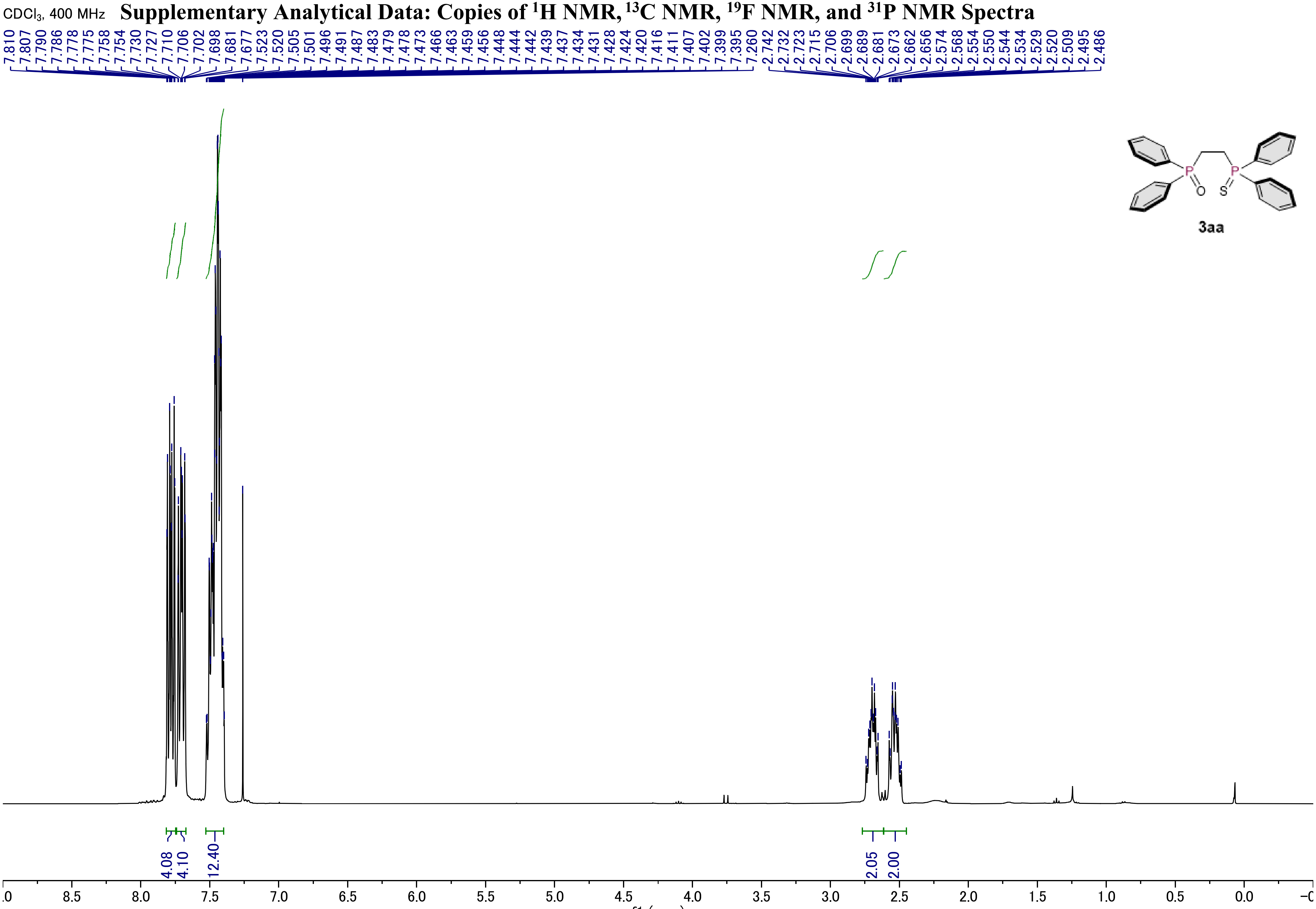

Supplementary Figure 14. ^{1}H NMR spectrum (400 MHz, $CDCl_3$) of **3aa**

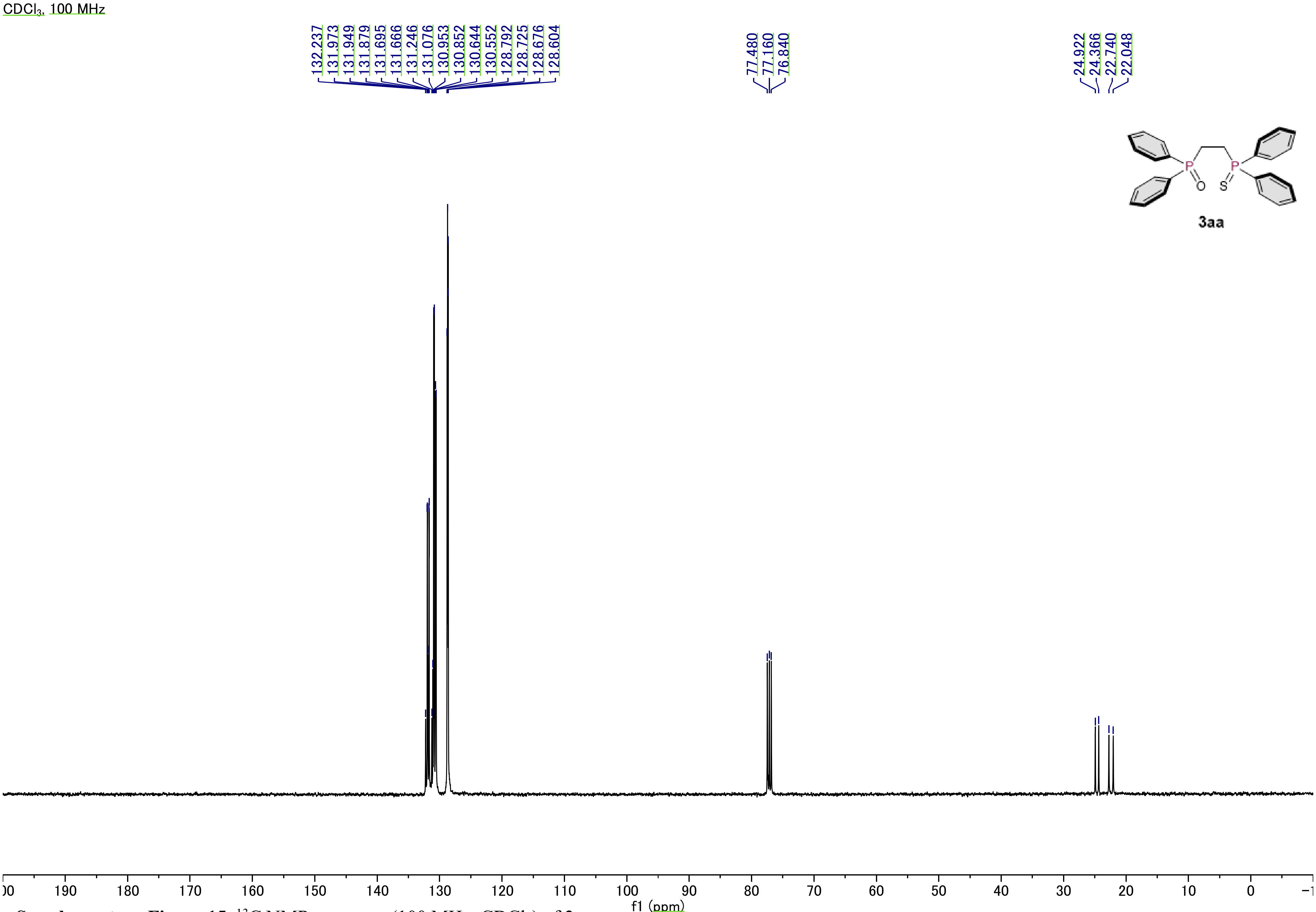

Supplementary Figure 15. ^{13}C NMR spectrum (100 MHz, $CDCl_3$) of **3aa**

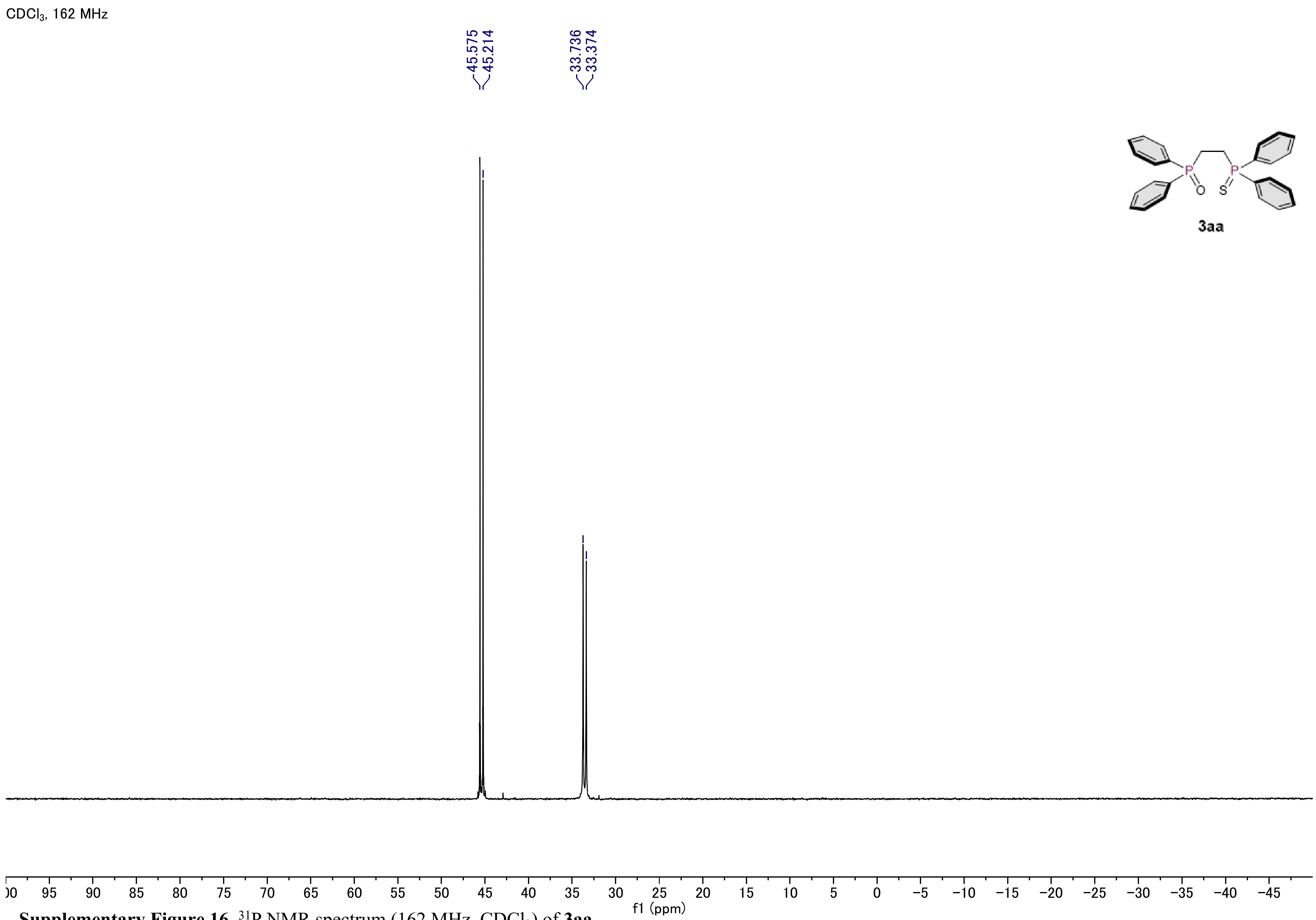

Supplementary Figure 16. ^{31}P NMR spectrum (162 MHz, $CDCl_3$) of **3aa**

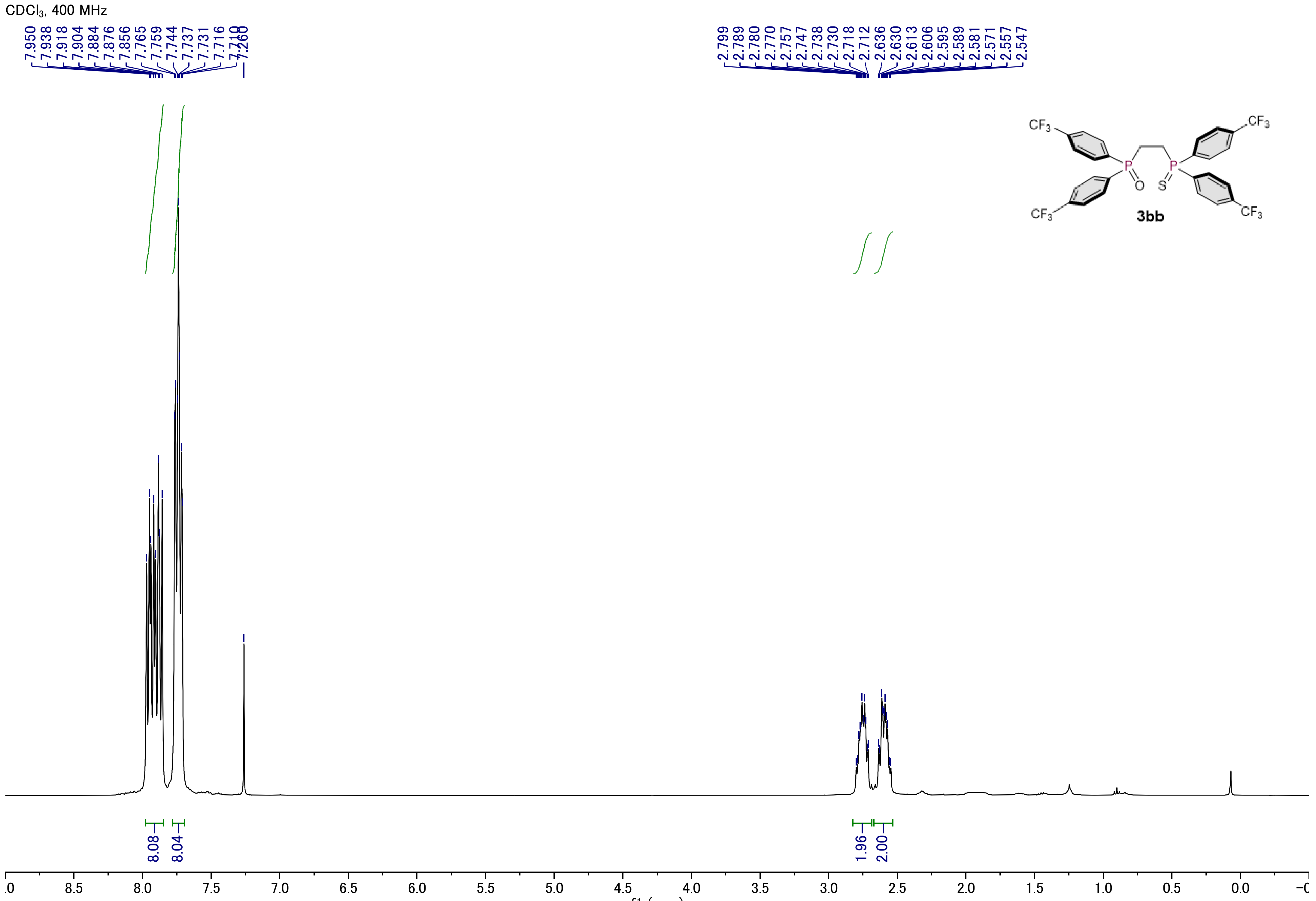

Supplementary Figure 17. ^{1}H NMR spectrum (400 MHz, $CDCl_3$) of **3bb**

CDCl$_3$, 100 MHz

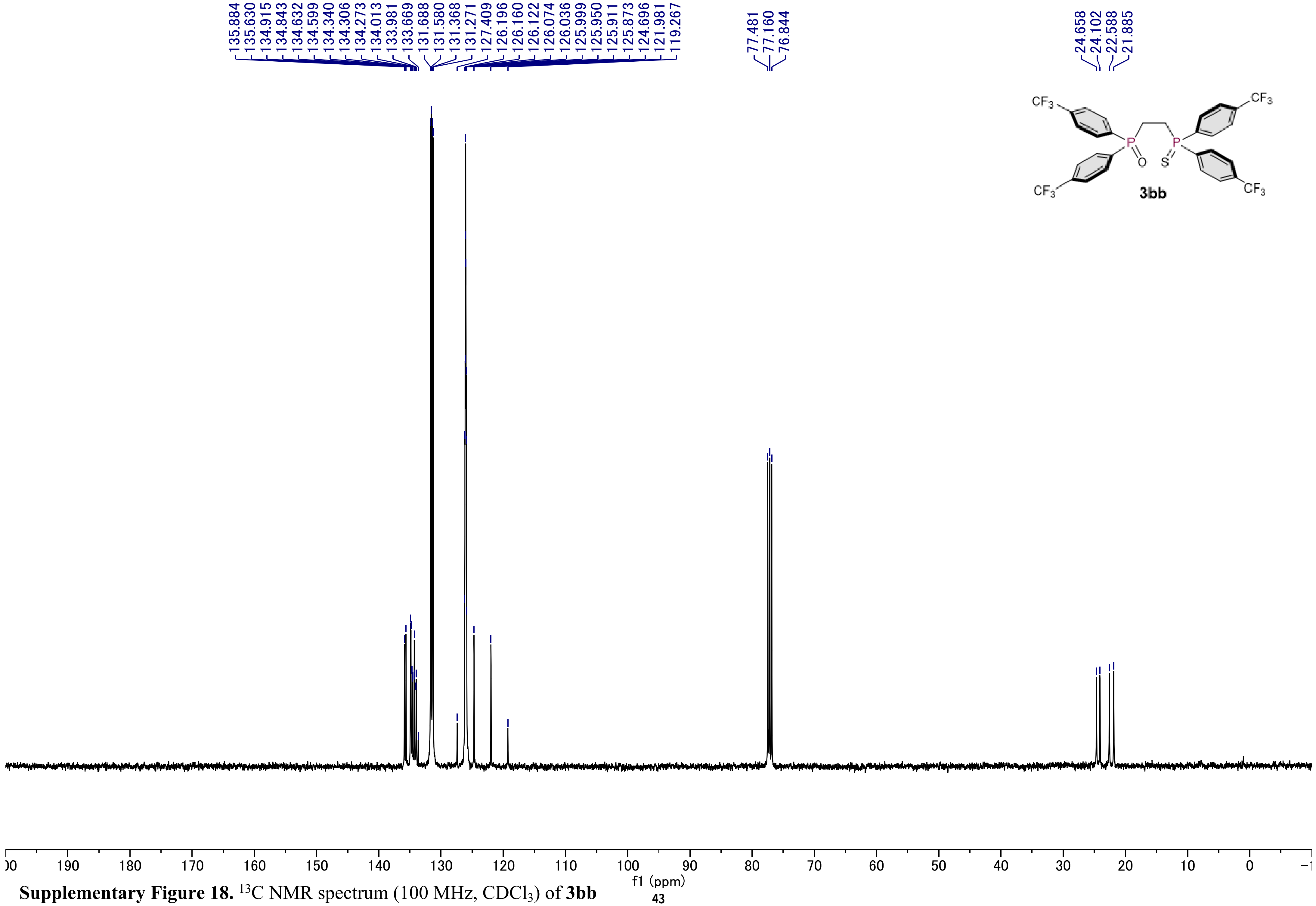

Supplementary Figure 18. ^{13}C NMR spectrum (100 MHz, CDCl$_3$) of **3bb**

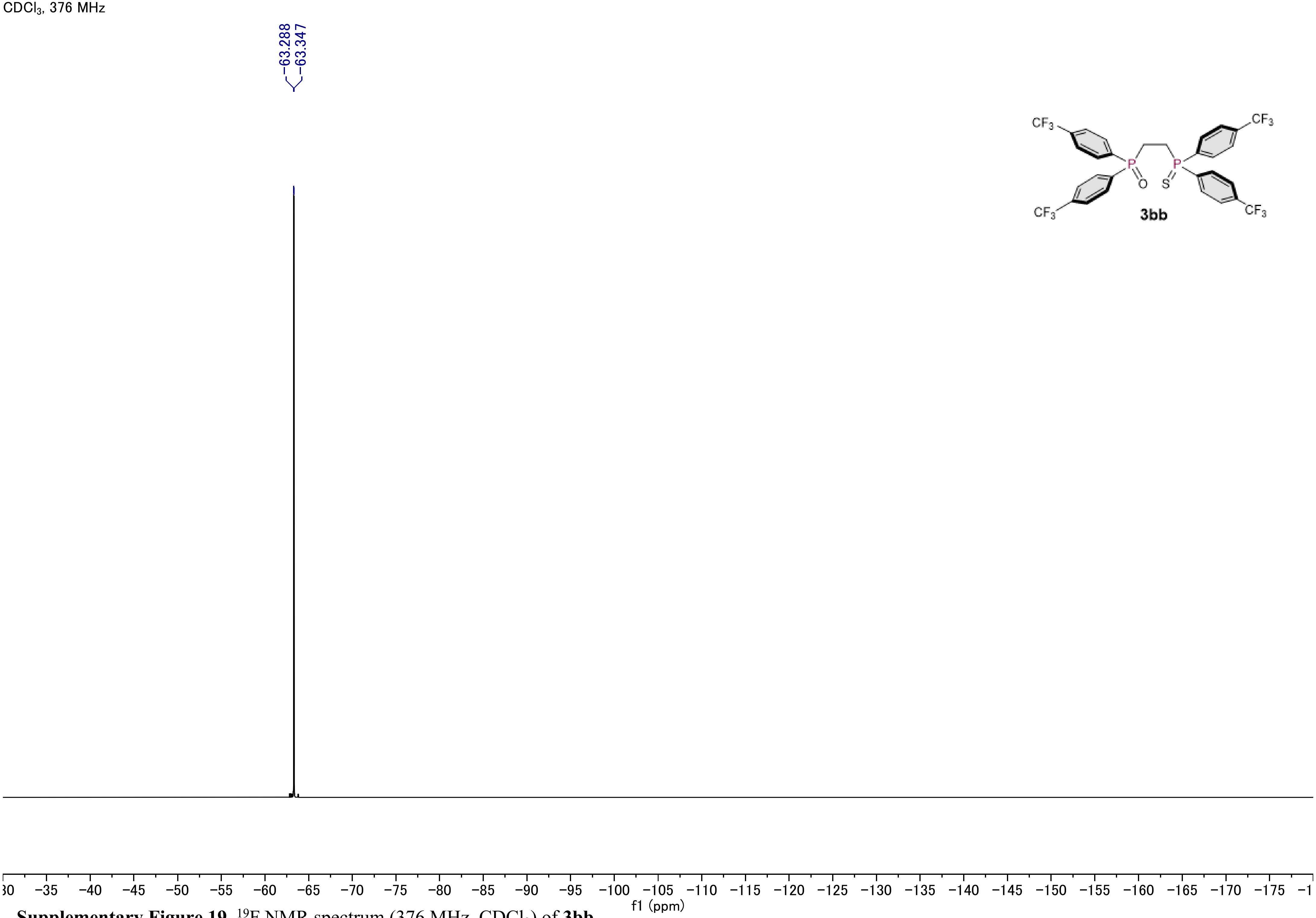

Supplementary Figure 19. ^{19}F NMR spectrum (376 MHz, $CDCl_3$) of **3bb**

CDCl3, 162 MHz

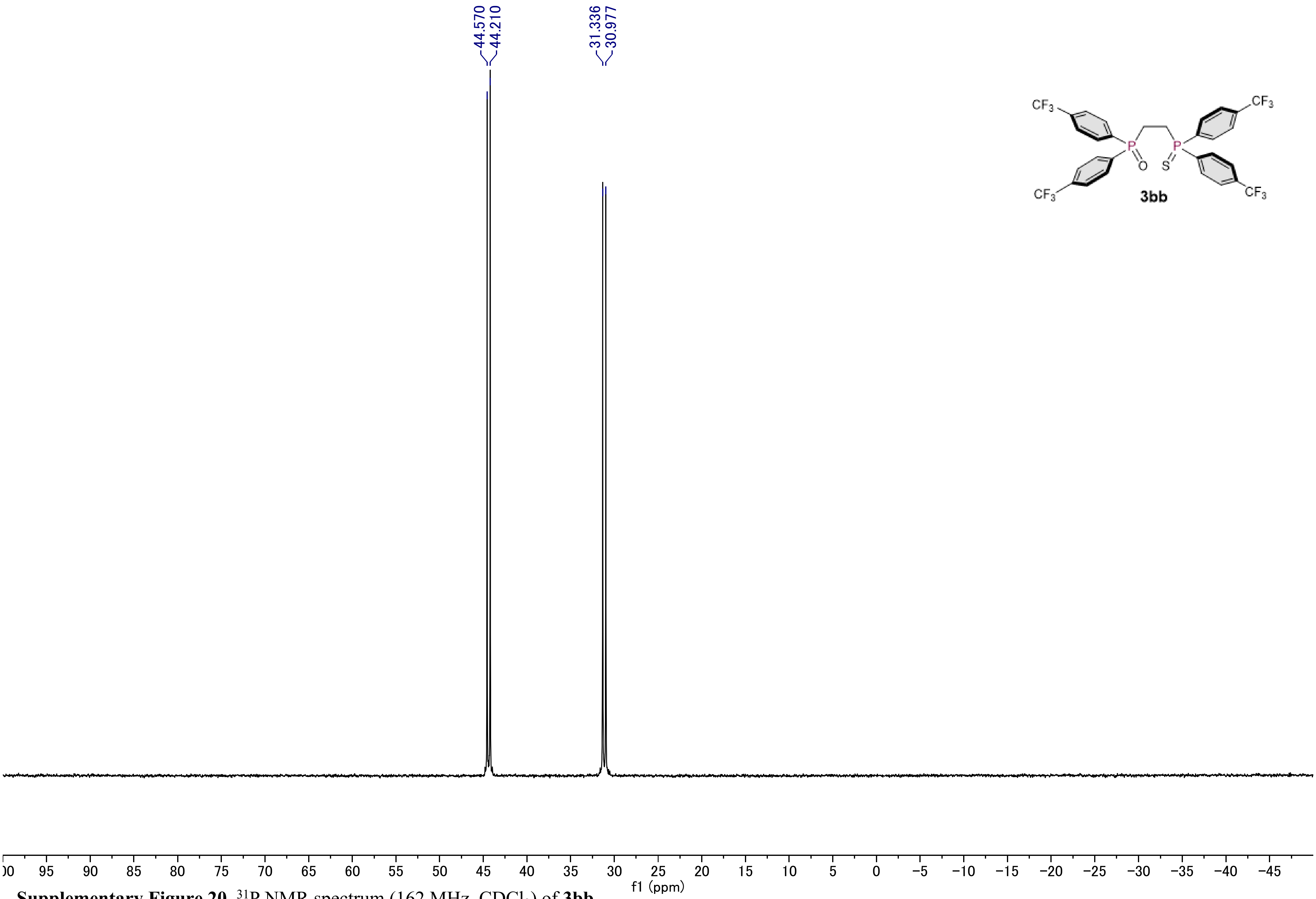

Supplementary Figure 20. ^{31}P NMR spectrum (162 MHz, $CDCl_3$) of **3bb**

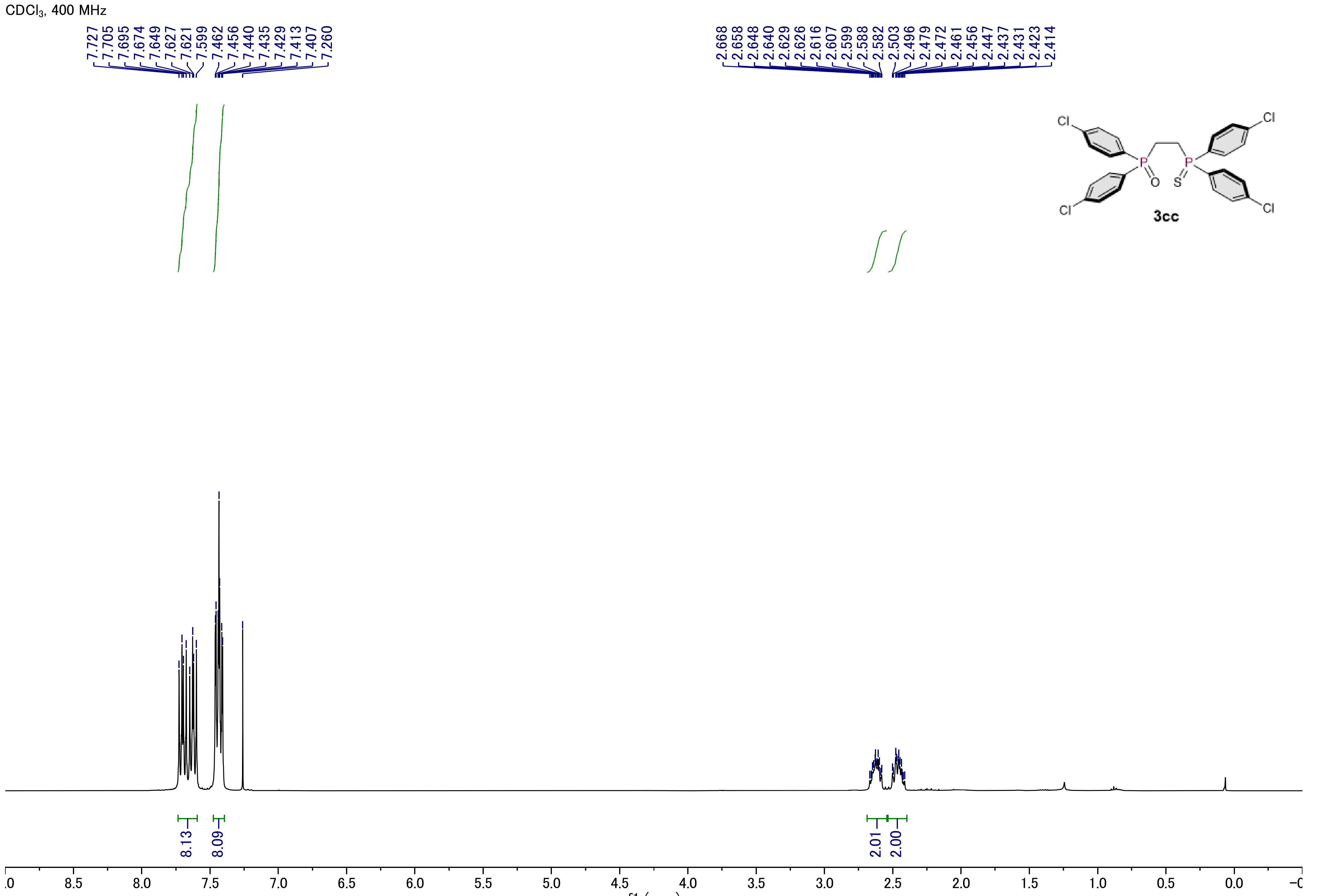

Supplementary Figure 21. ^{1}H NMR spectrum (400 MHz, $CDCl_3$) of **3cc**

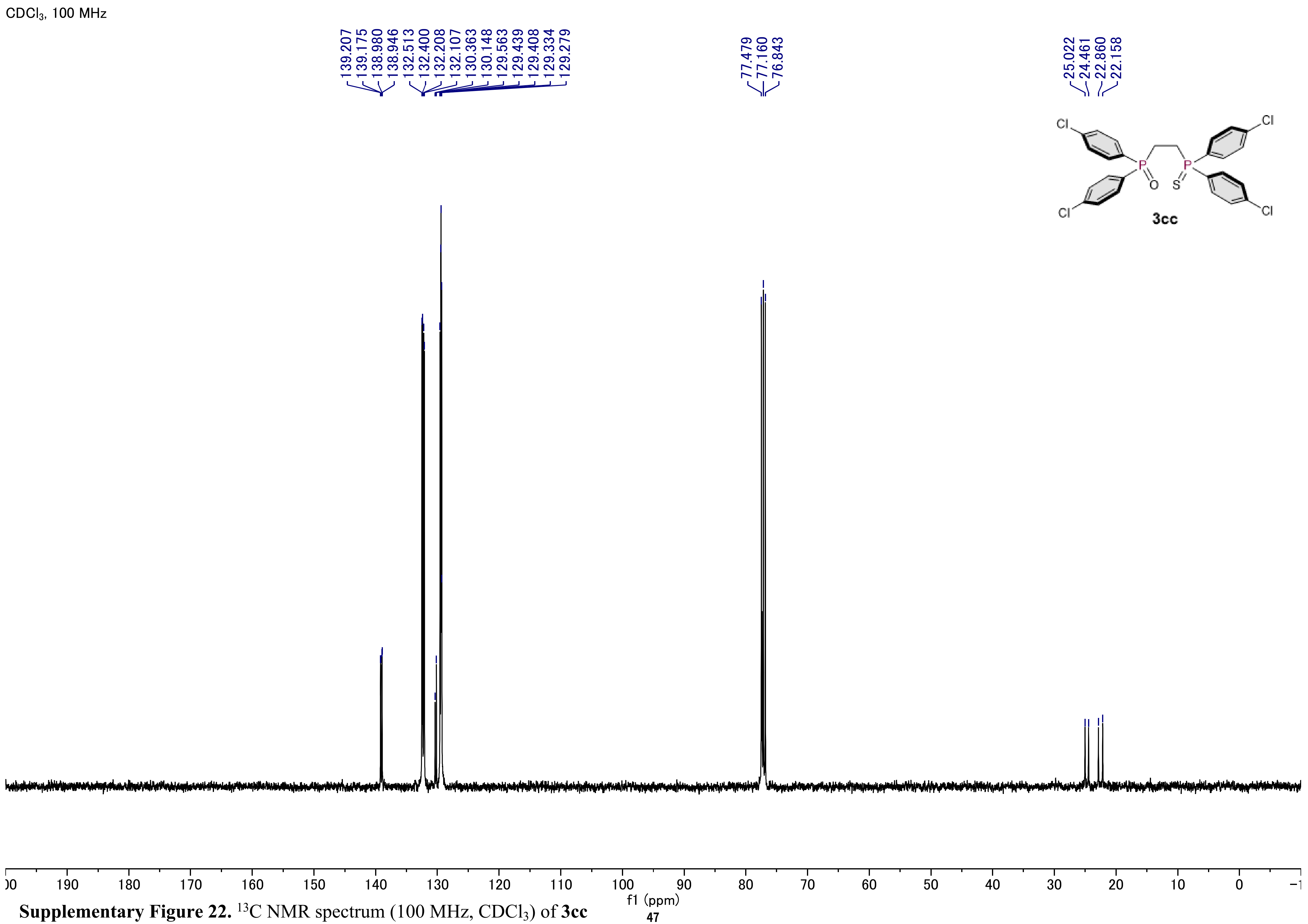

Supplementary Figure 22. ^{13}C NMR spectrum (100 MHz, $CDCl_3$) of **3cc**

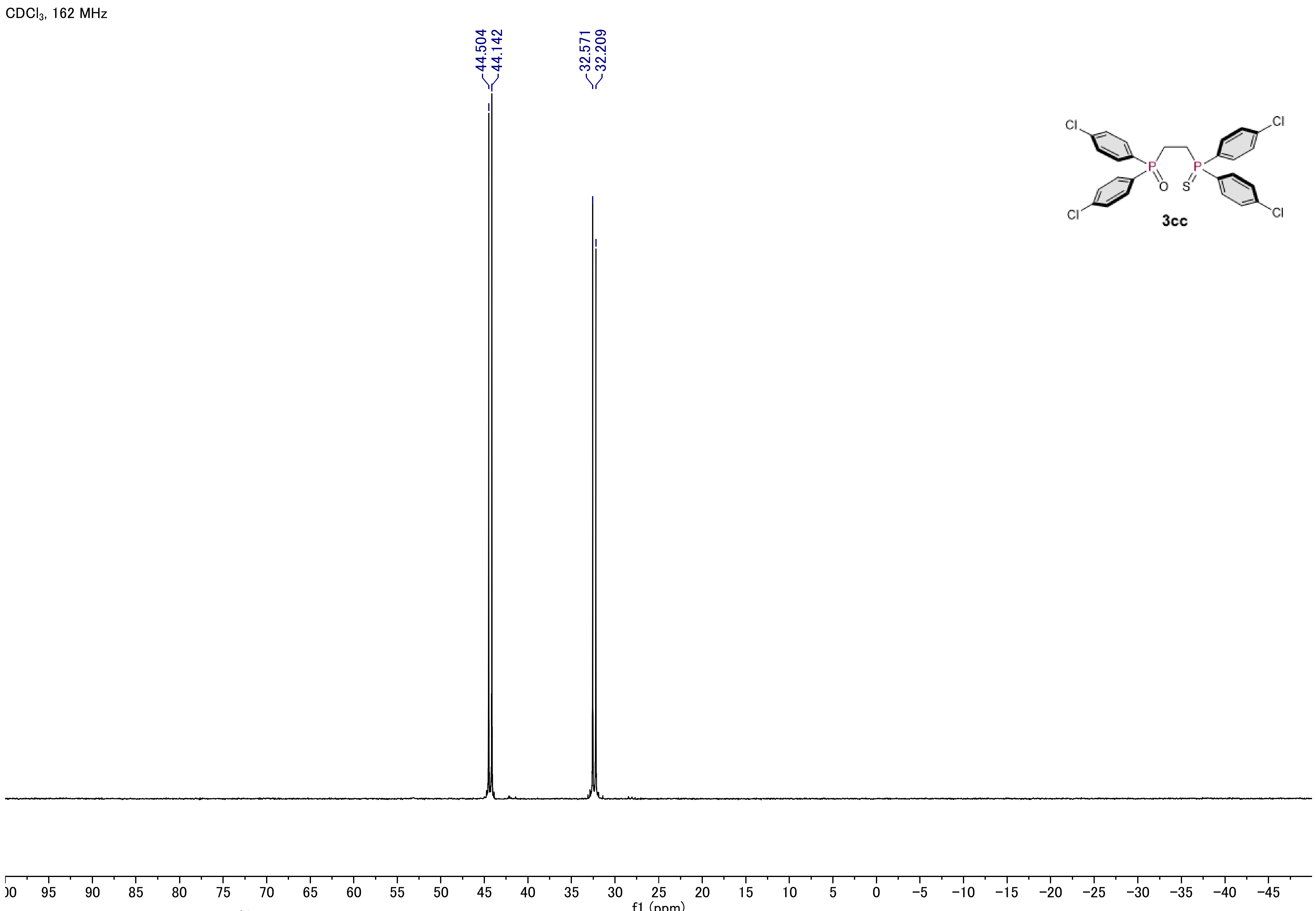

Supplementary Figure 23. ^{31}P NMR spectrum (162 MHz, $CDCl_3$) of **3cc**

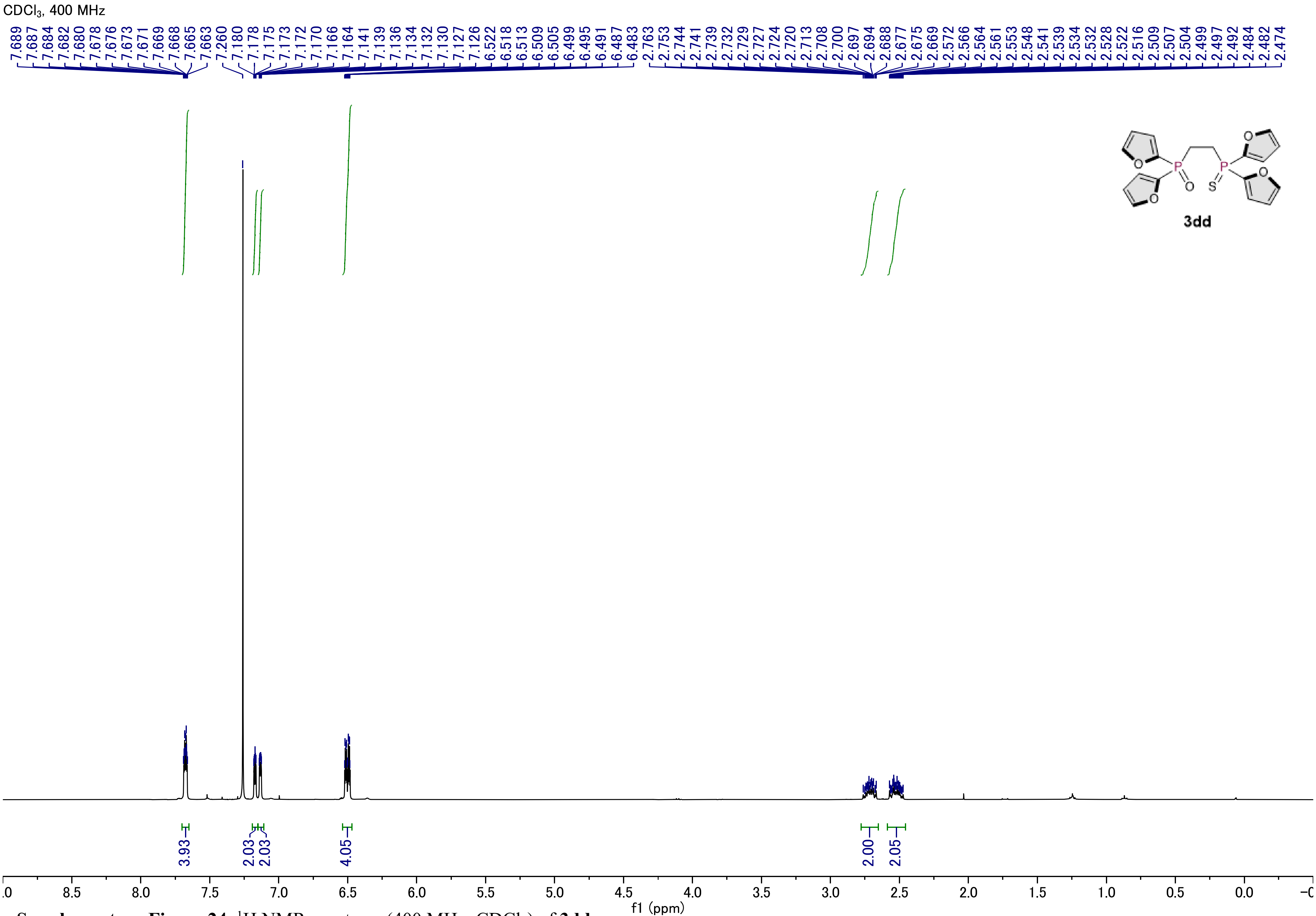

Supplementary Figure 24. ^{1}H NMR spectrum (400 MHz, $CDCl_3$) of **3dd**

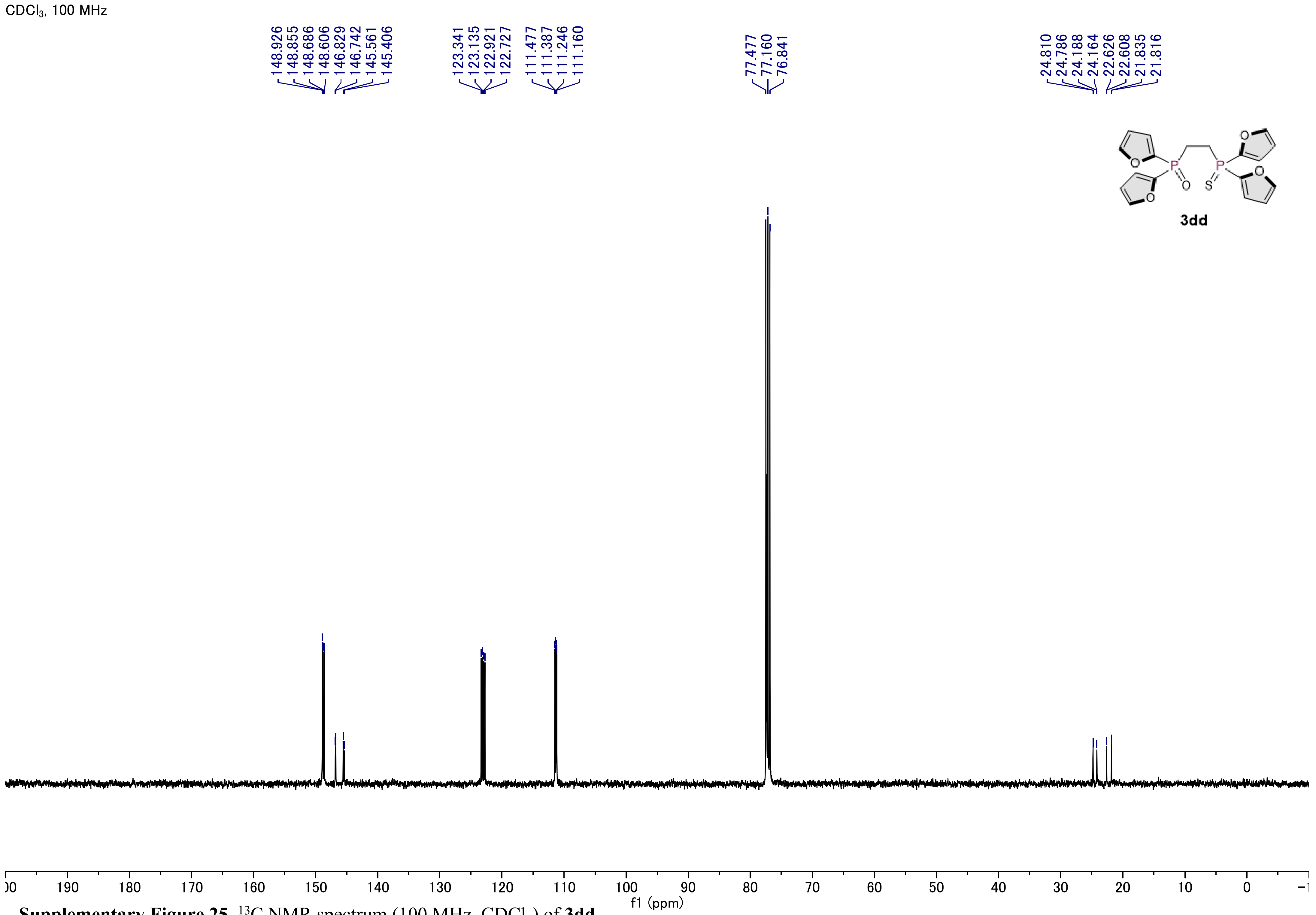

Supplementary Figure 25. ^{13}C NMR spectrum (100 MHz, $CDCl_3$) of **3dd**

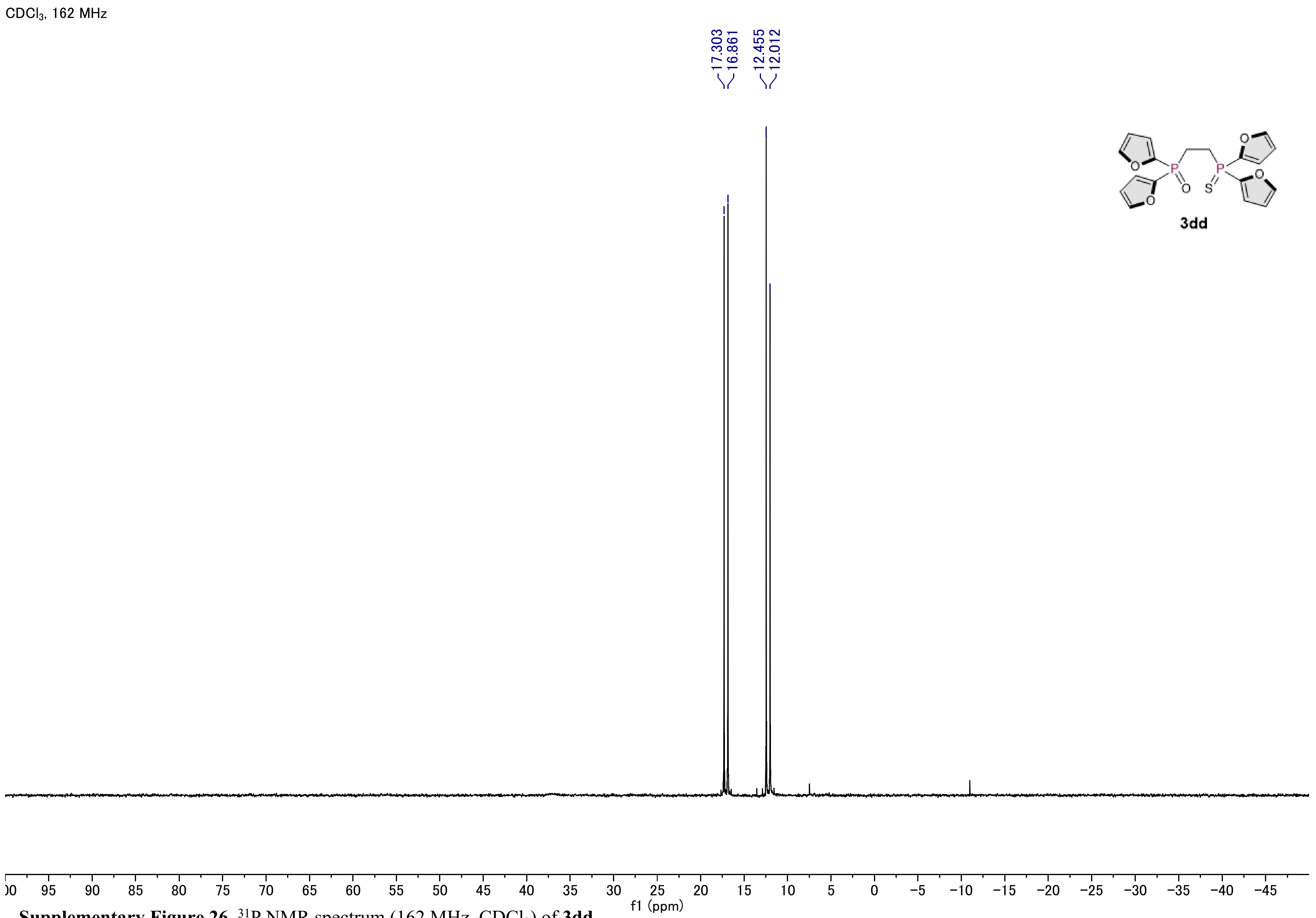

Supplementary Figure 26. ^{31}P NMR spectrum (162 MHz, $CDCl_3$) of **3dd**

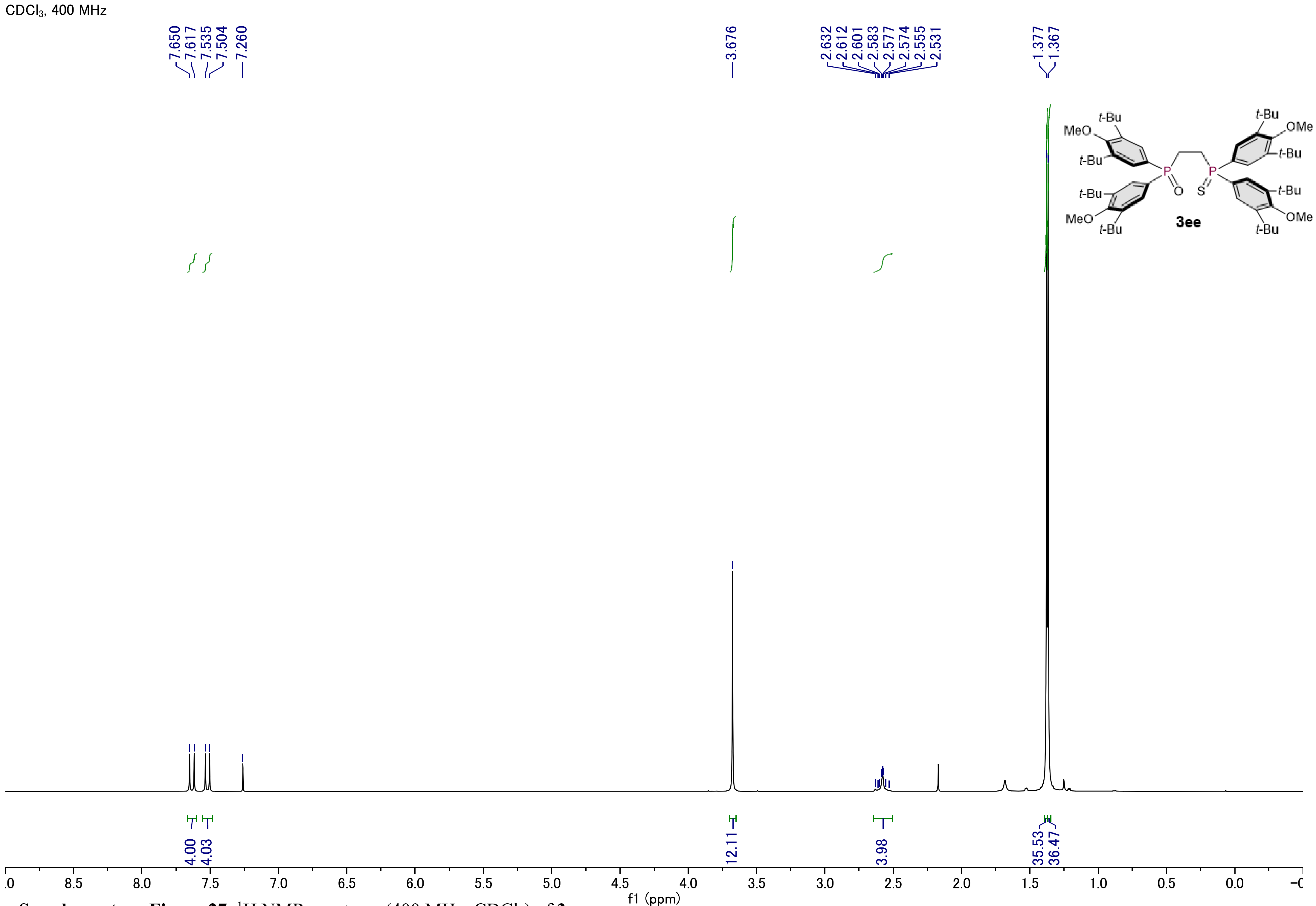

Supplementary Figure 27. ^{1}H NMR spectrum (400 MHz, $CDCl_3$) of **3ee**

CDCl$_3$, 100 MHz

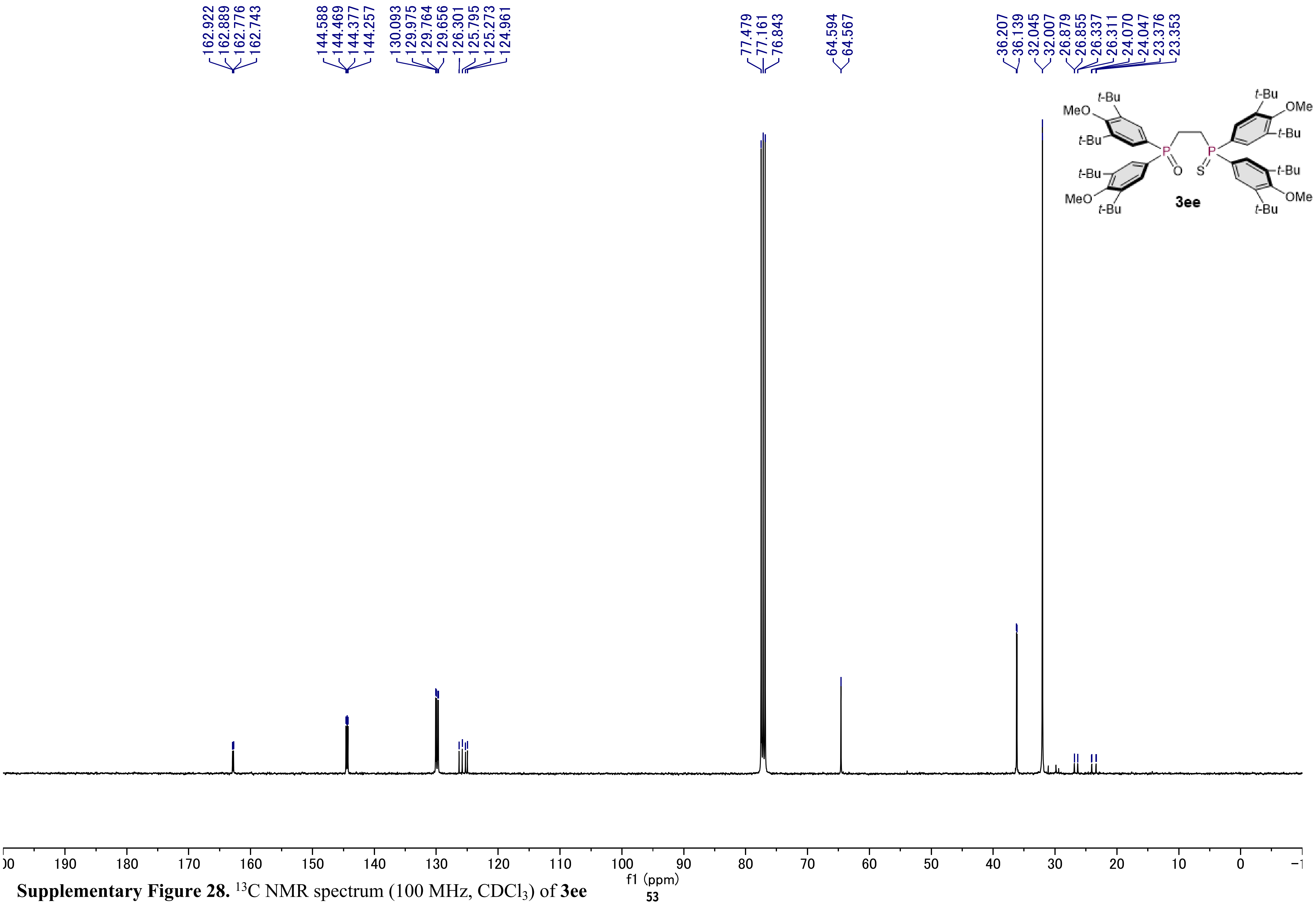

Supplementary Figure 28. ^{13}C NMR spectrum (100 MHz, CDCl$_3$) of **3ee**

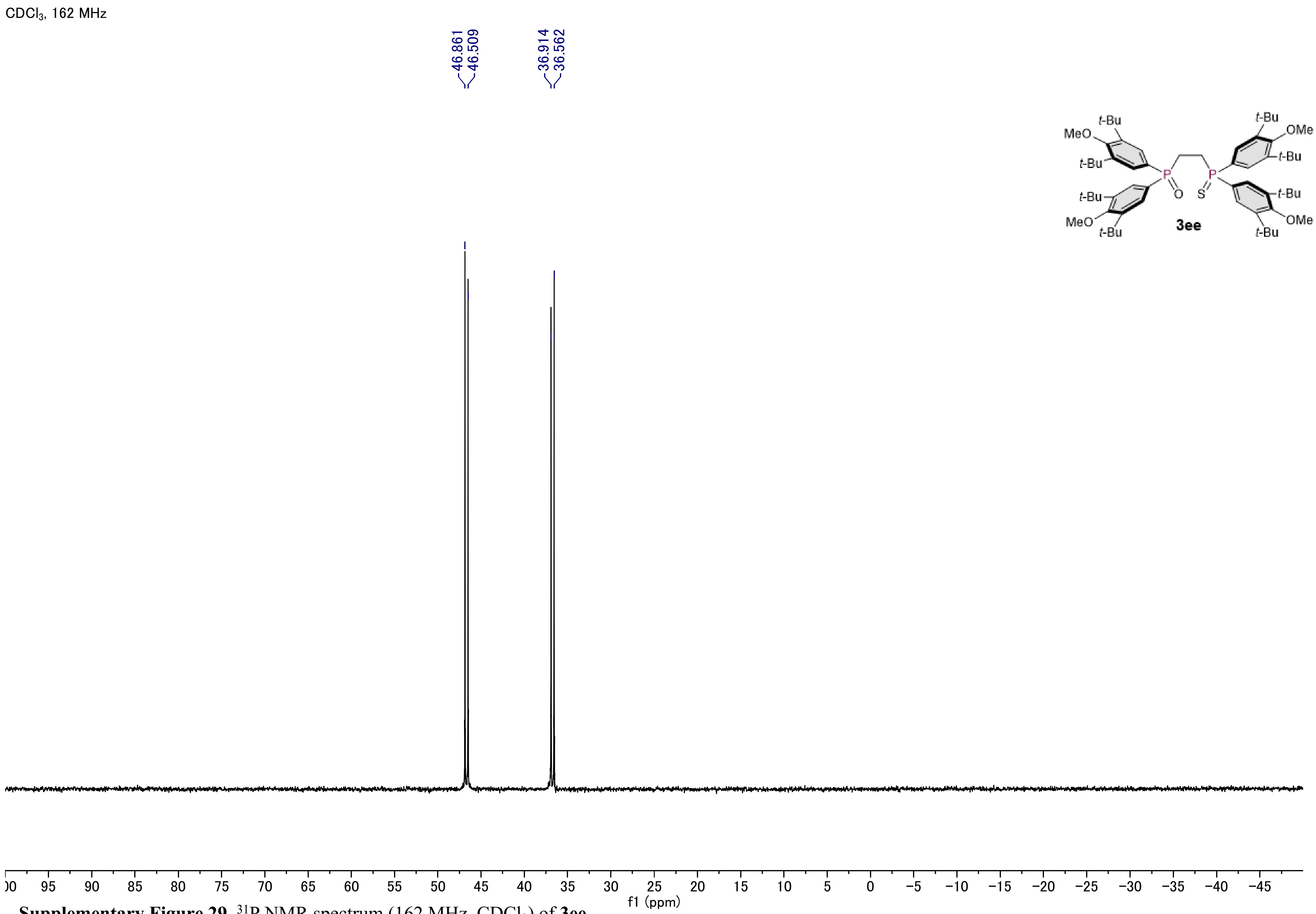

Supplementary Figure 29. ^{31}P NMR spectrum (162 MHz, CDCl$_3$) of **3ee**

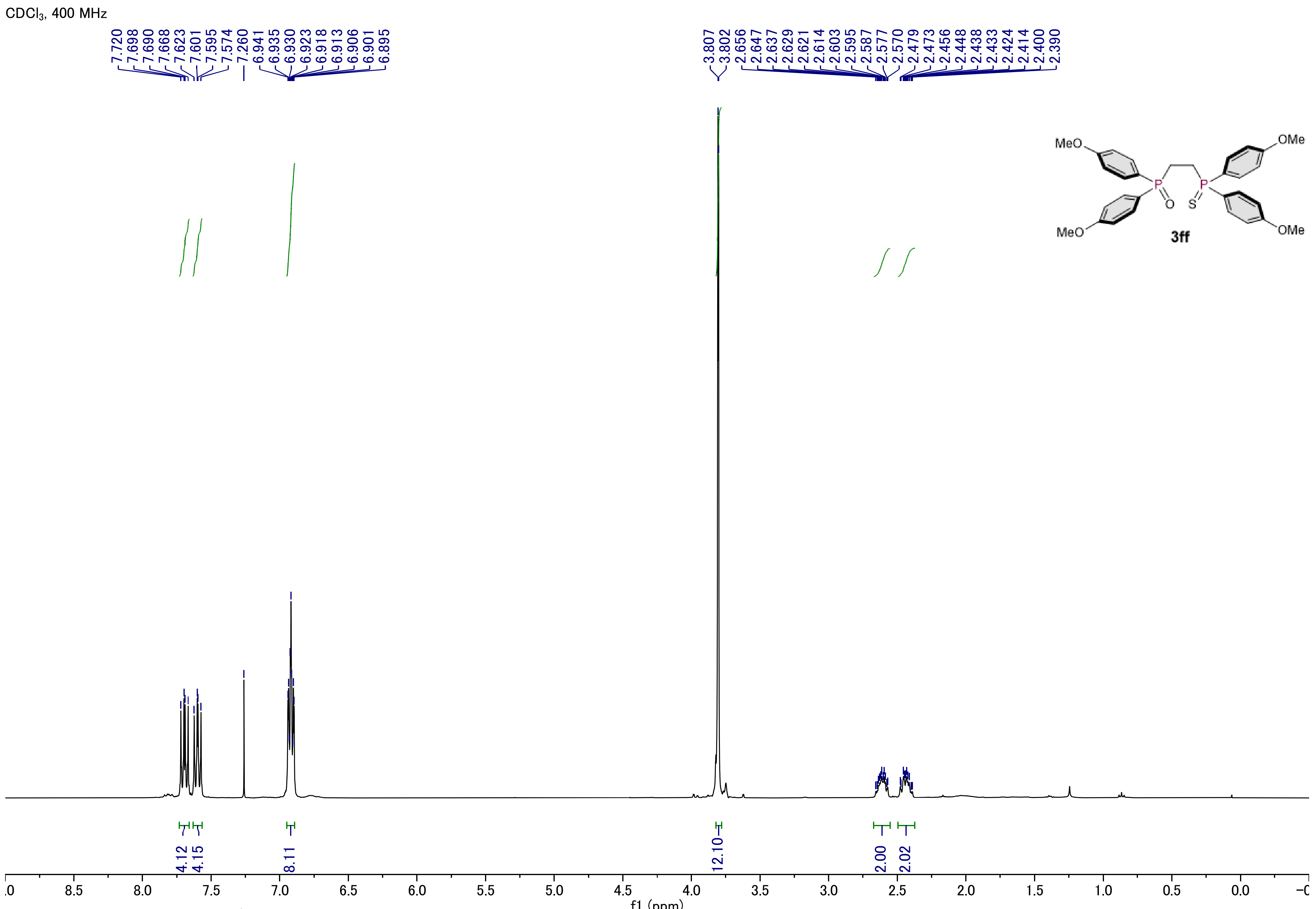

Supplementary Figure 30. ^{1}H NMR spectrum (400 MHz, $CDCl_3$) of **3ff**

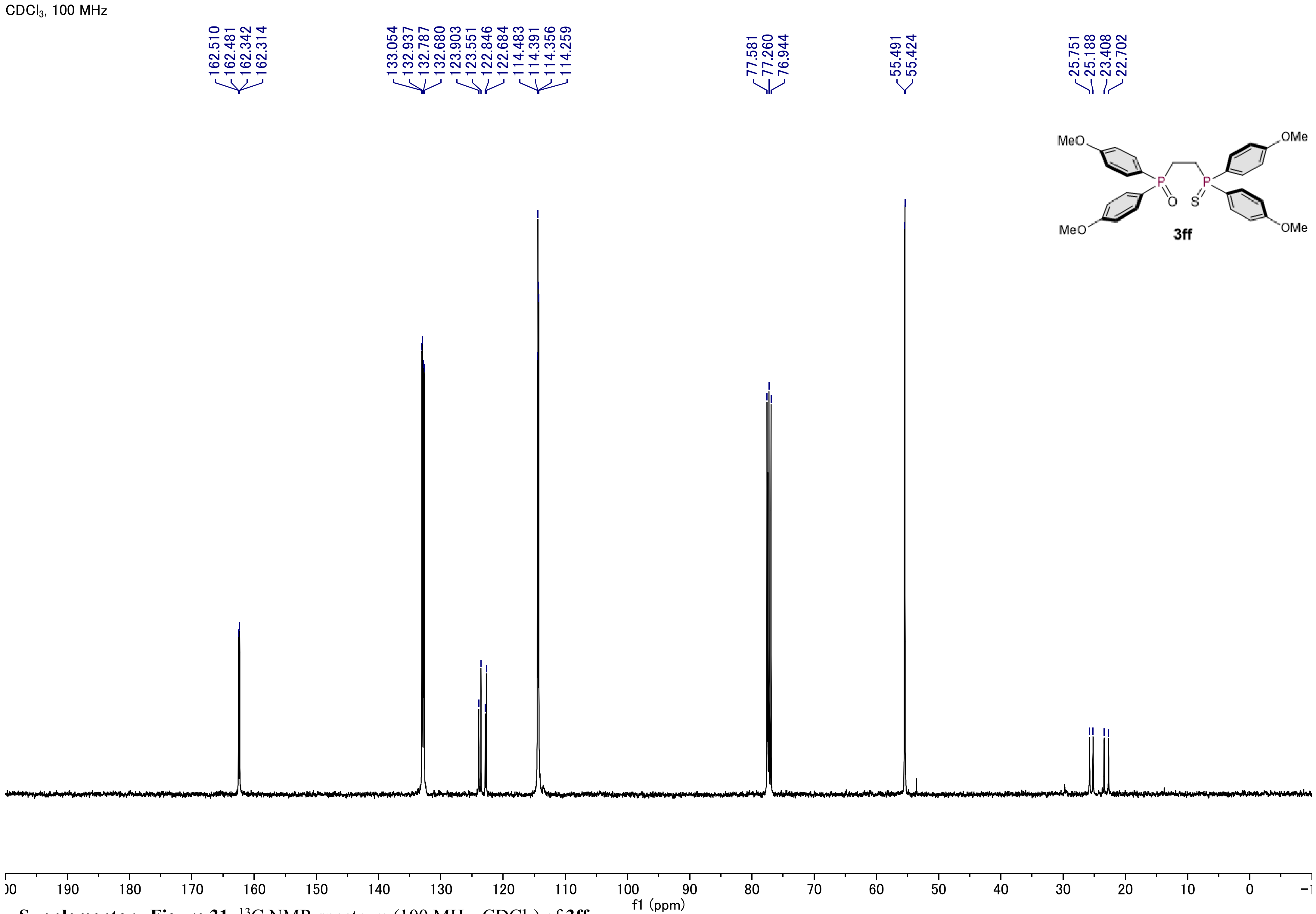

Supplementary Figure 31. ^{13}C NMR spectrum (100 MHz, $CDCl_3$) of **3ff**

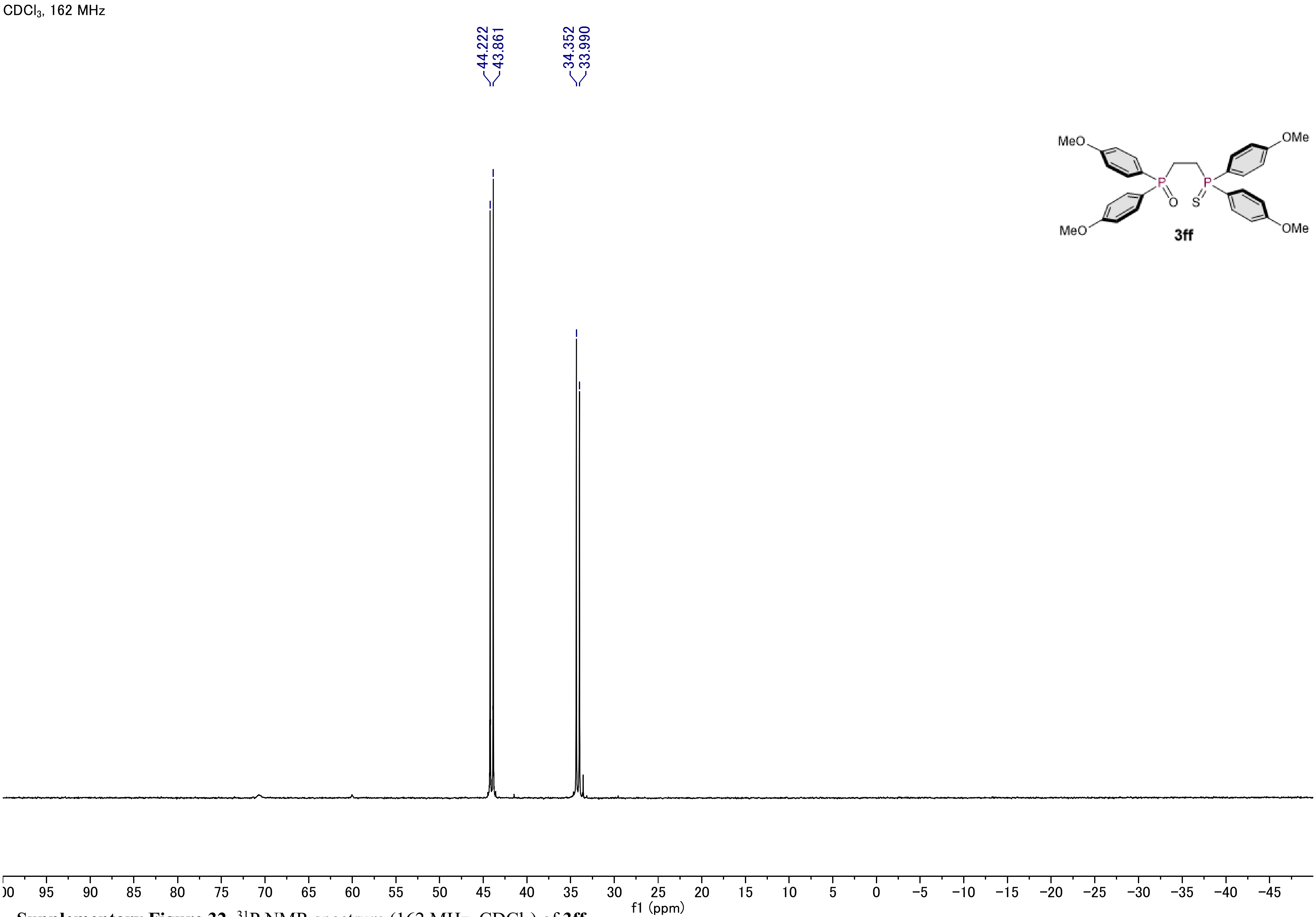

Supplementary Figure 32. ^{31}P NMR spectrum (162 MHz, $CDCl_3$) of **3ff**

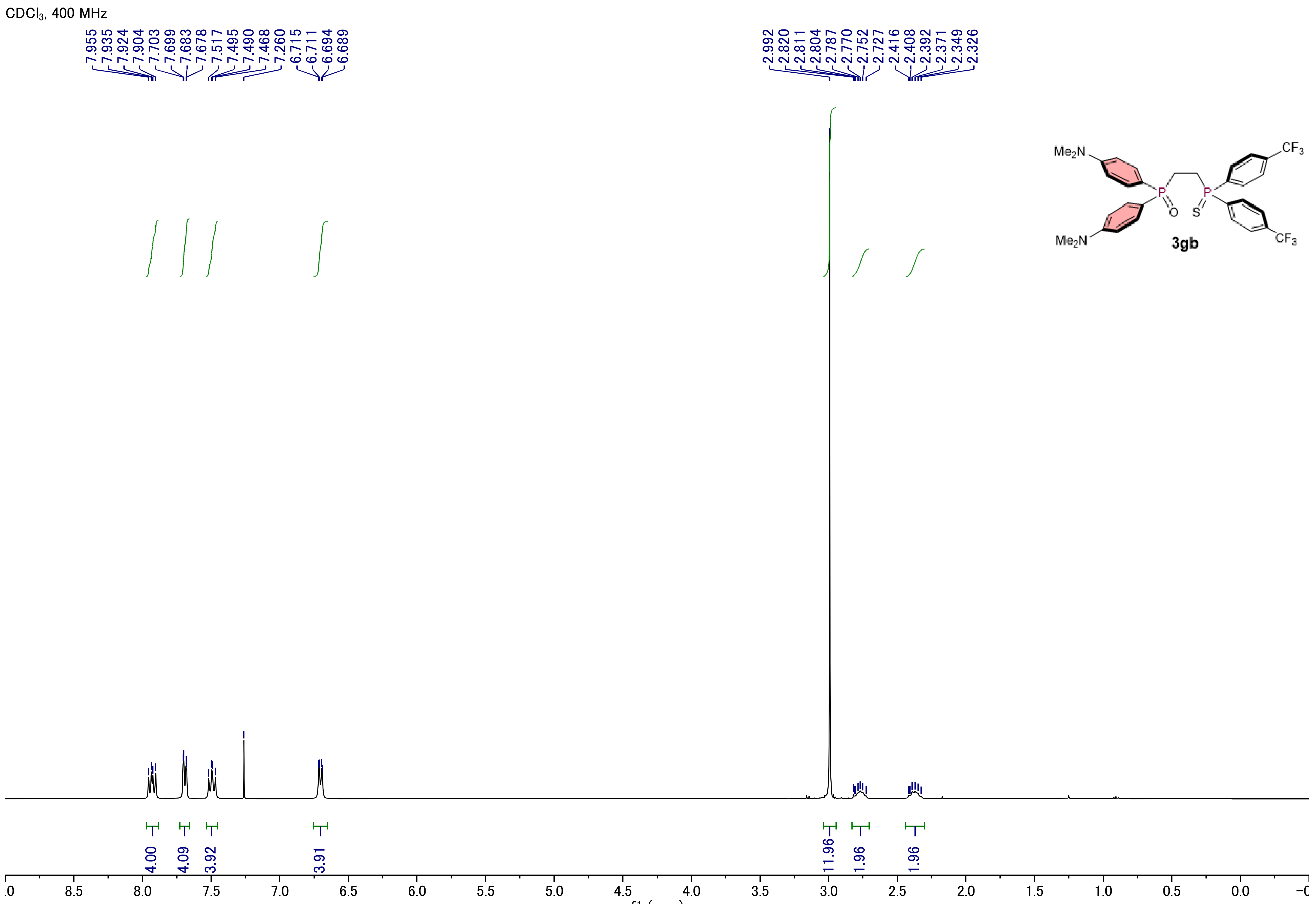

Supplementary Figure 33. ^{1}H NMR spectrum (400 MHz, $CDCl_3$) of **3gb**

CDCl3, 100 MHz

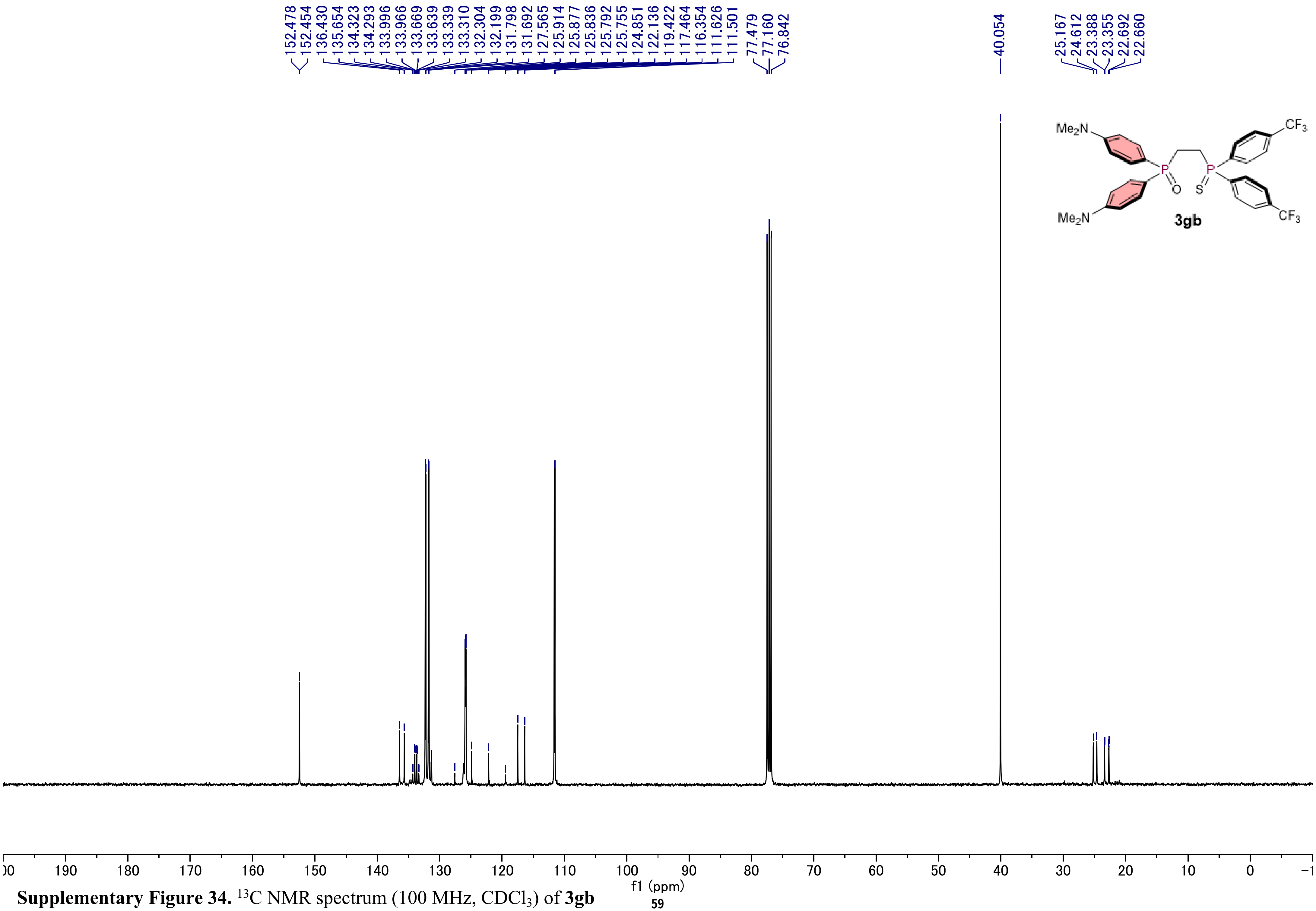

Supplementary Figure 34. ^{13}C NMR spectrum (100 MHz, $CDCl_3$) of **3gb**

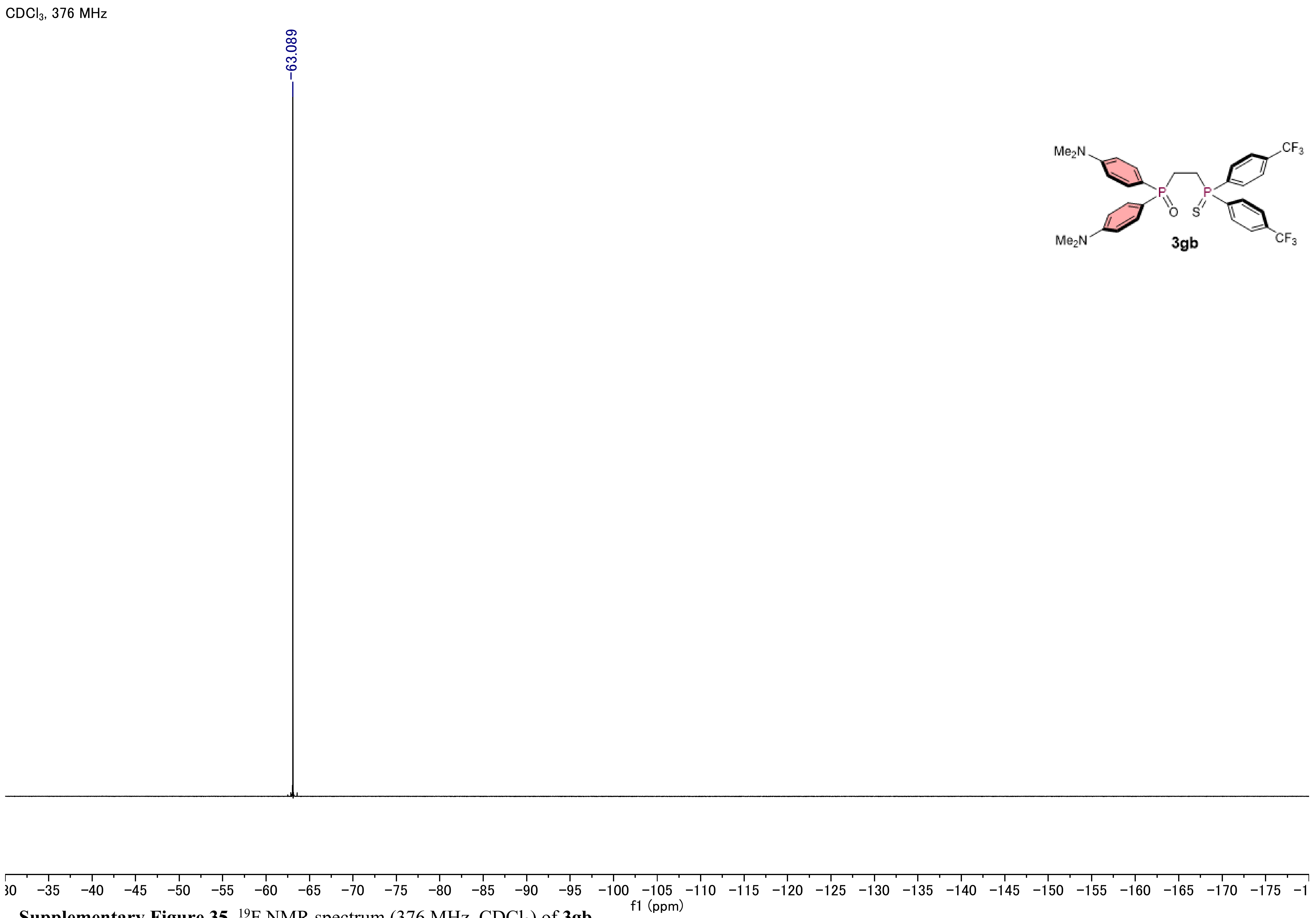

Supplementary Figure 35. ^{19}F NMR spectrum (376 MHz, $CDCl_3$) of **3gb**

CDCl$_3$, 162 MHz

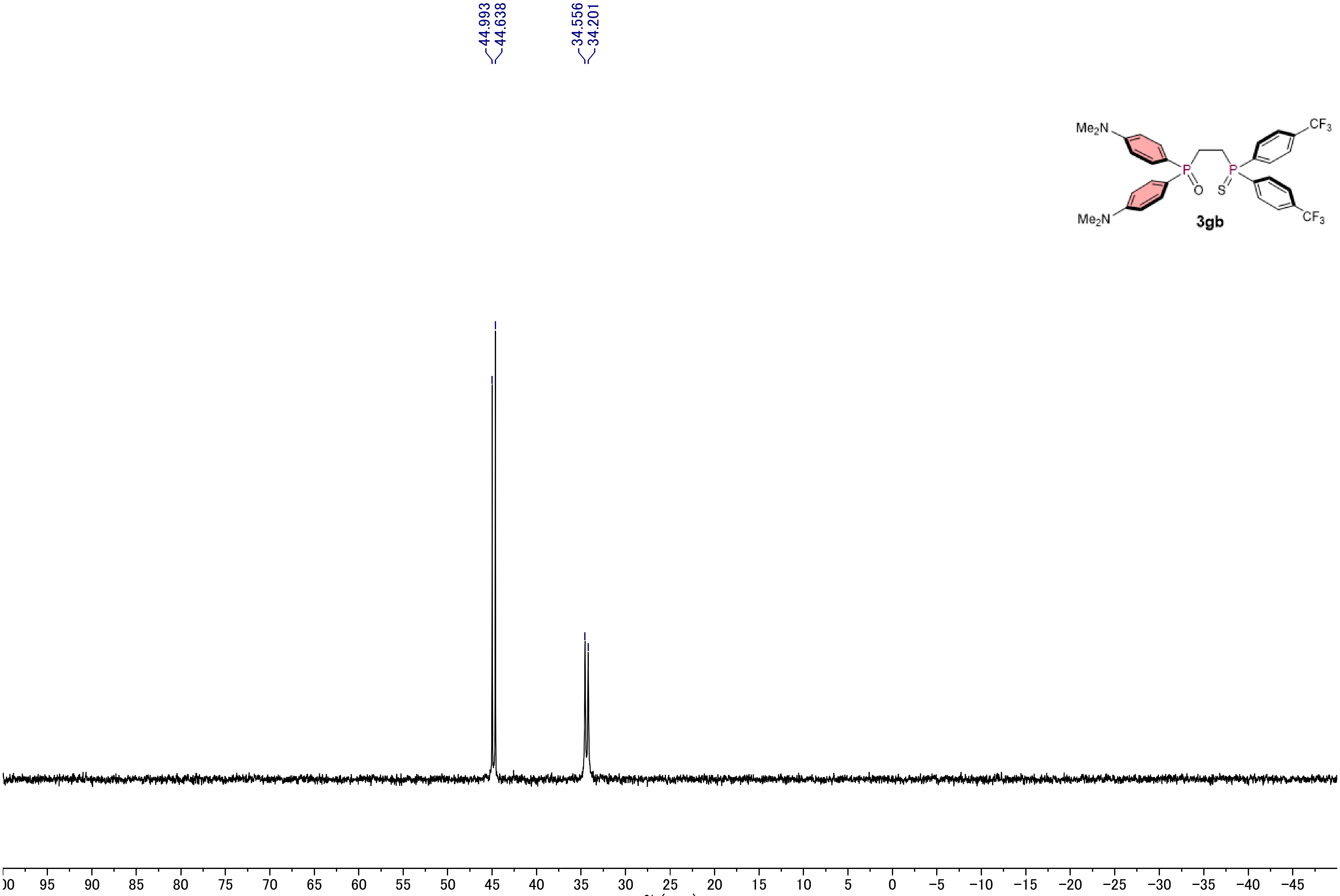

Supplementary Figure 36. ^{31}P NMR spectrum (162 MHz, CDCl$_3$) of **3gb**

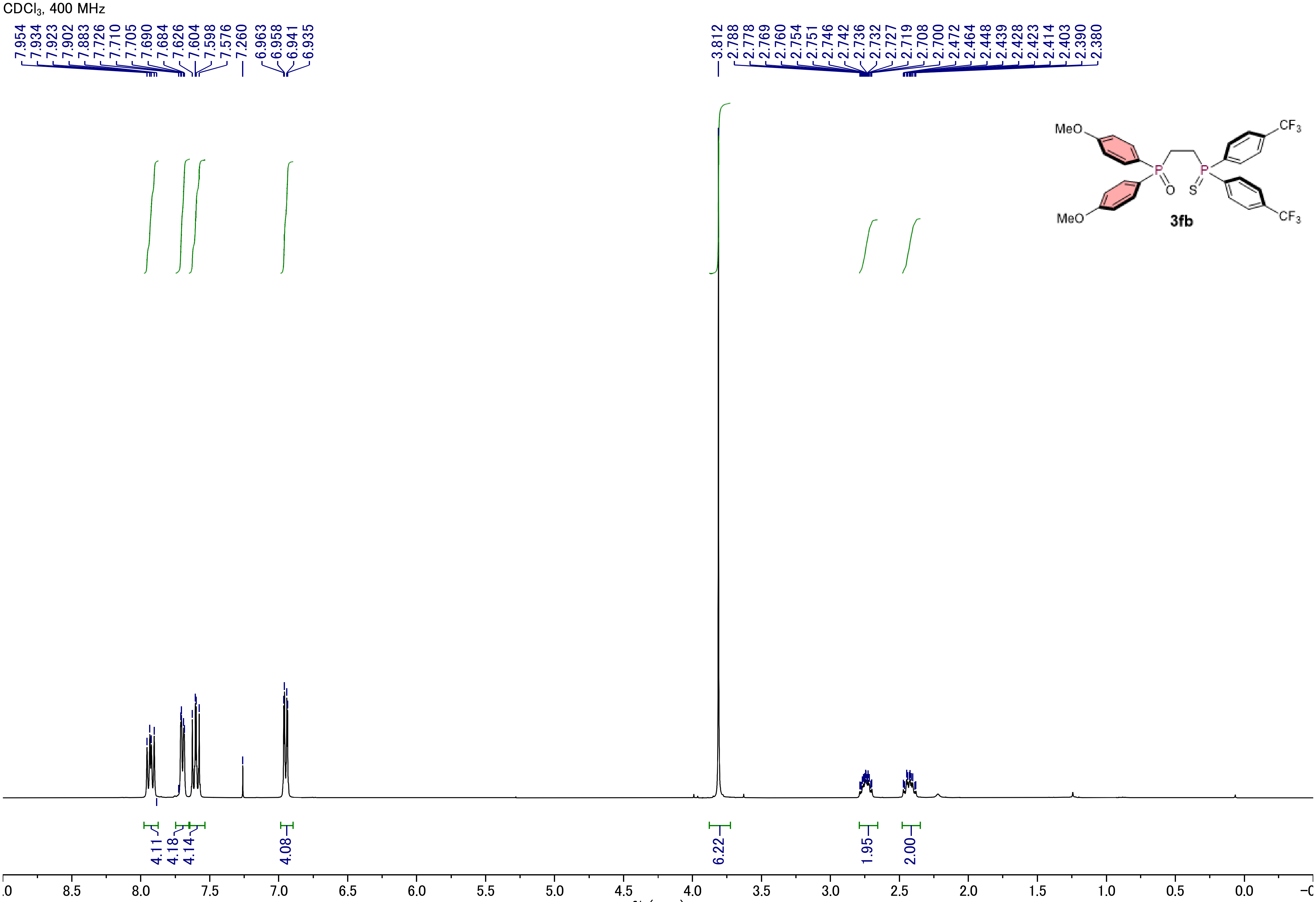

Supplementary Figure 37. ^{1}H NMR spectrum (400 MHz, $CDCl_3$) of **3fb**

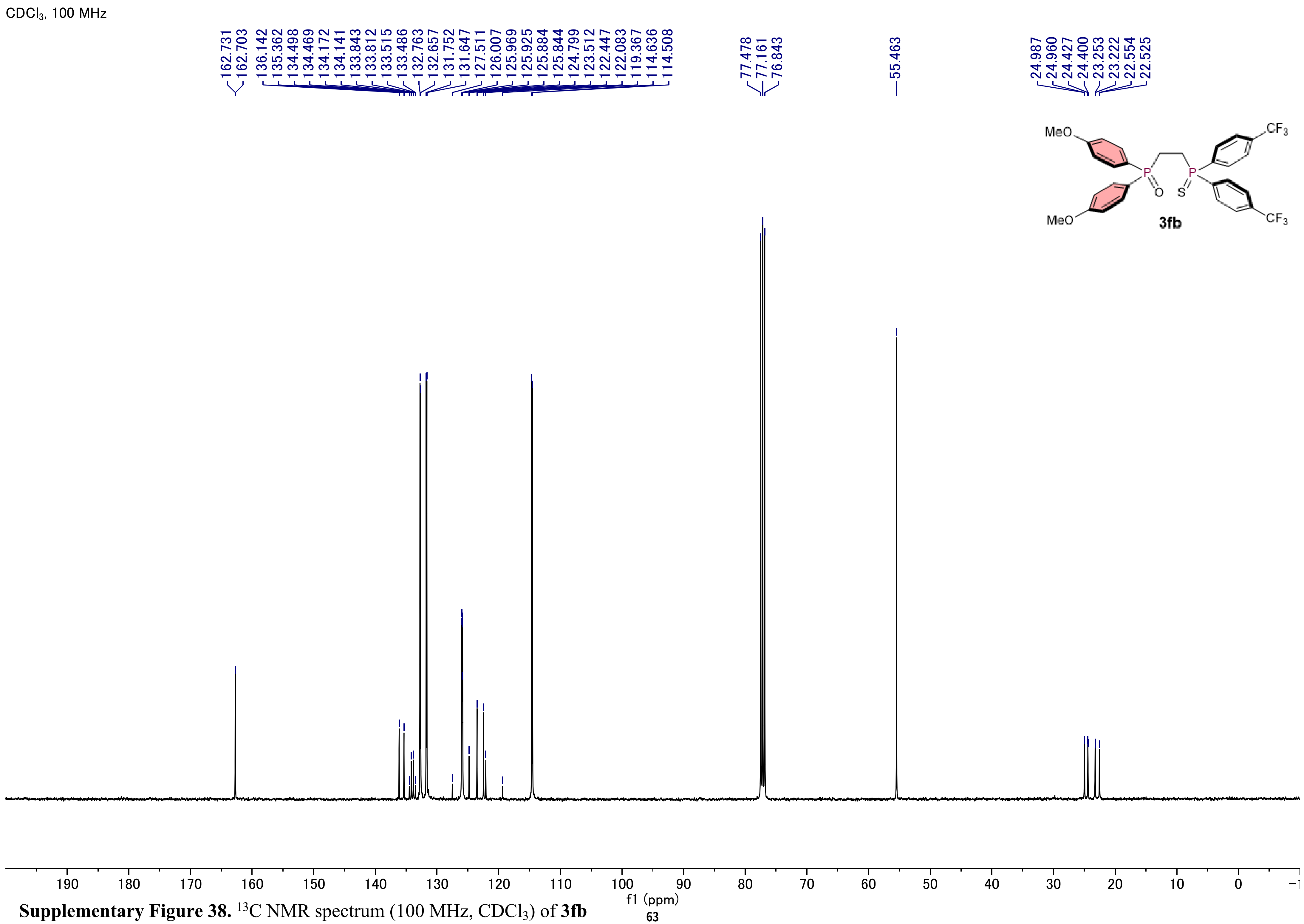

Supplementary Figure 38. ^{13}C NMR spectrum (100 MHz, $CDCl_3$) of **3fb**

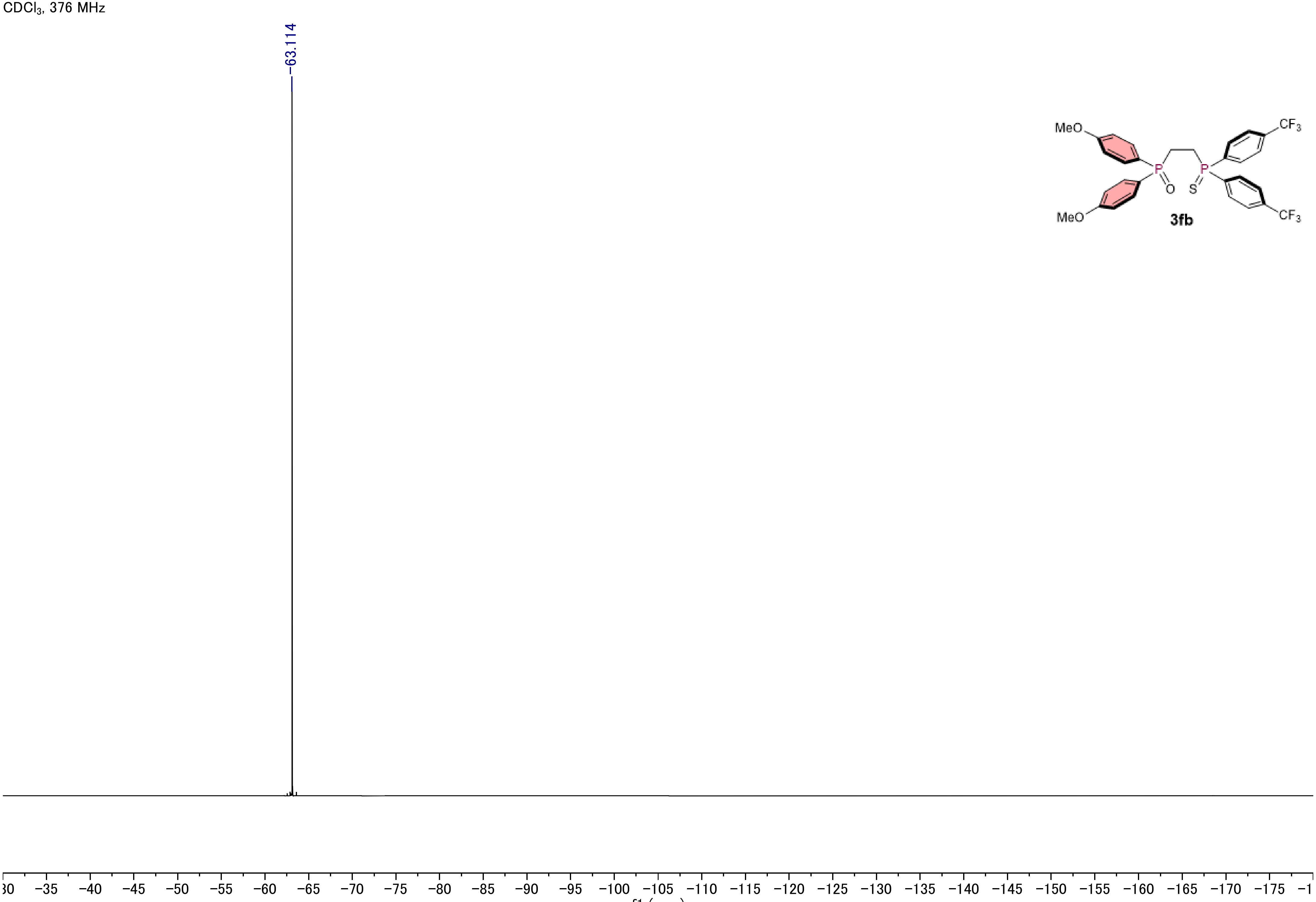

Supplementary Figure 39. ^{19}F NMR spectrum (376 MHz, $CDCl_3$) of **3fb**

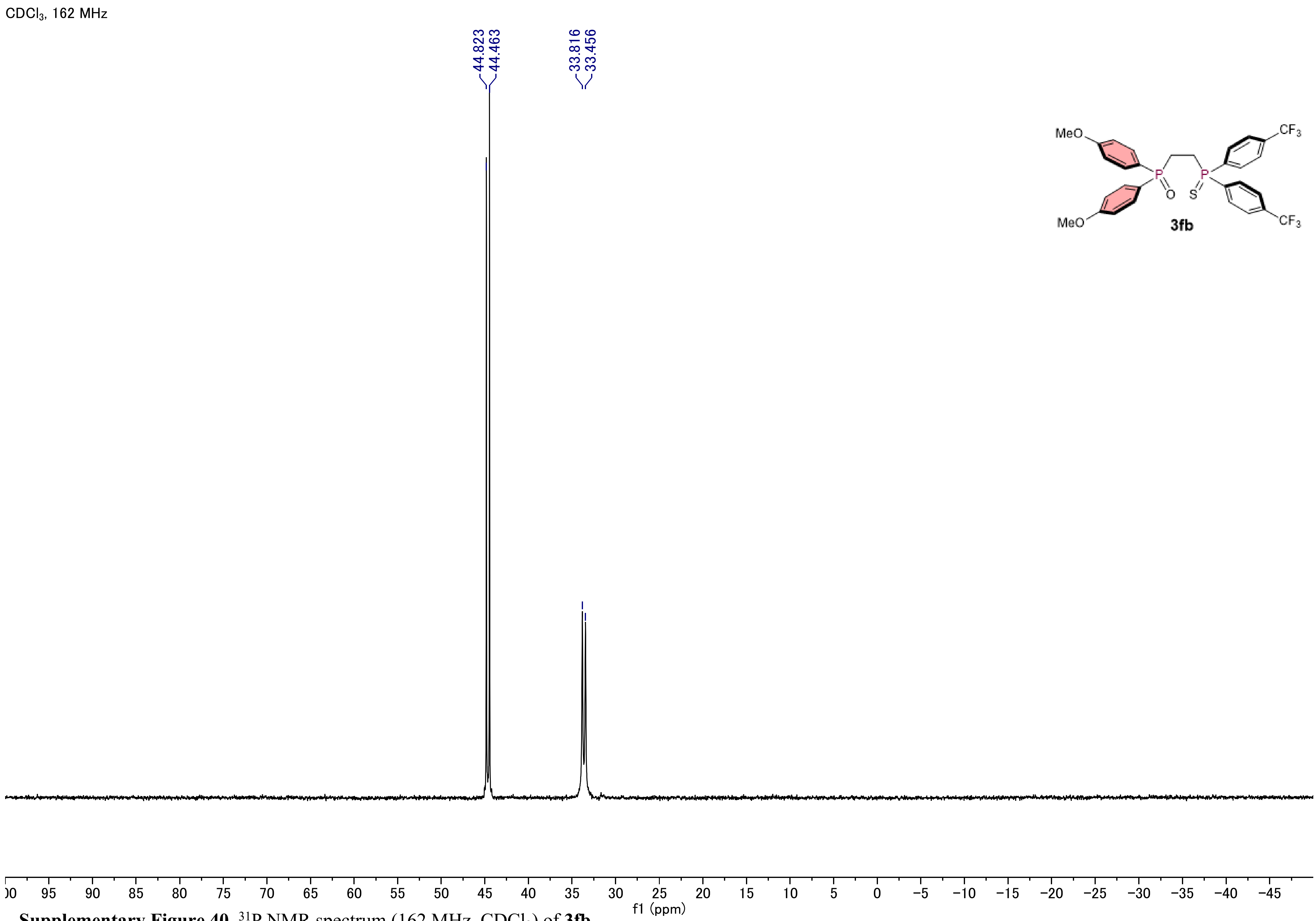

3fb

Supplementary Figure 40. ^{31}P NMR spectrum (162 MHz, $CDCl_3$) of **3fb**

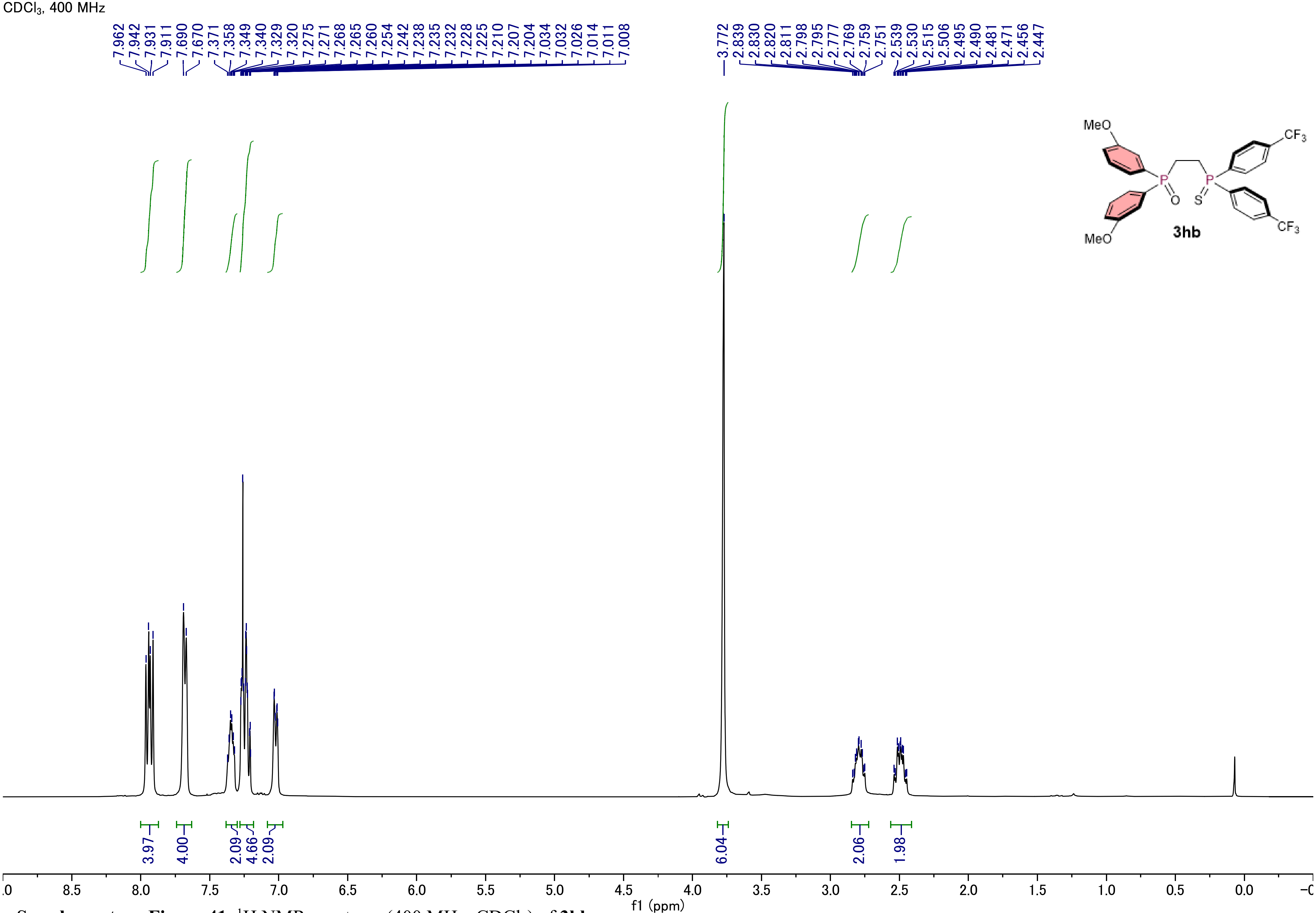

Supplementary Figure 41. ^{1}H NMR spectrum (400 MHz, $CDCl_3$) of **3hb**

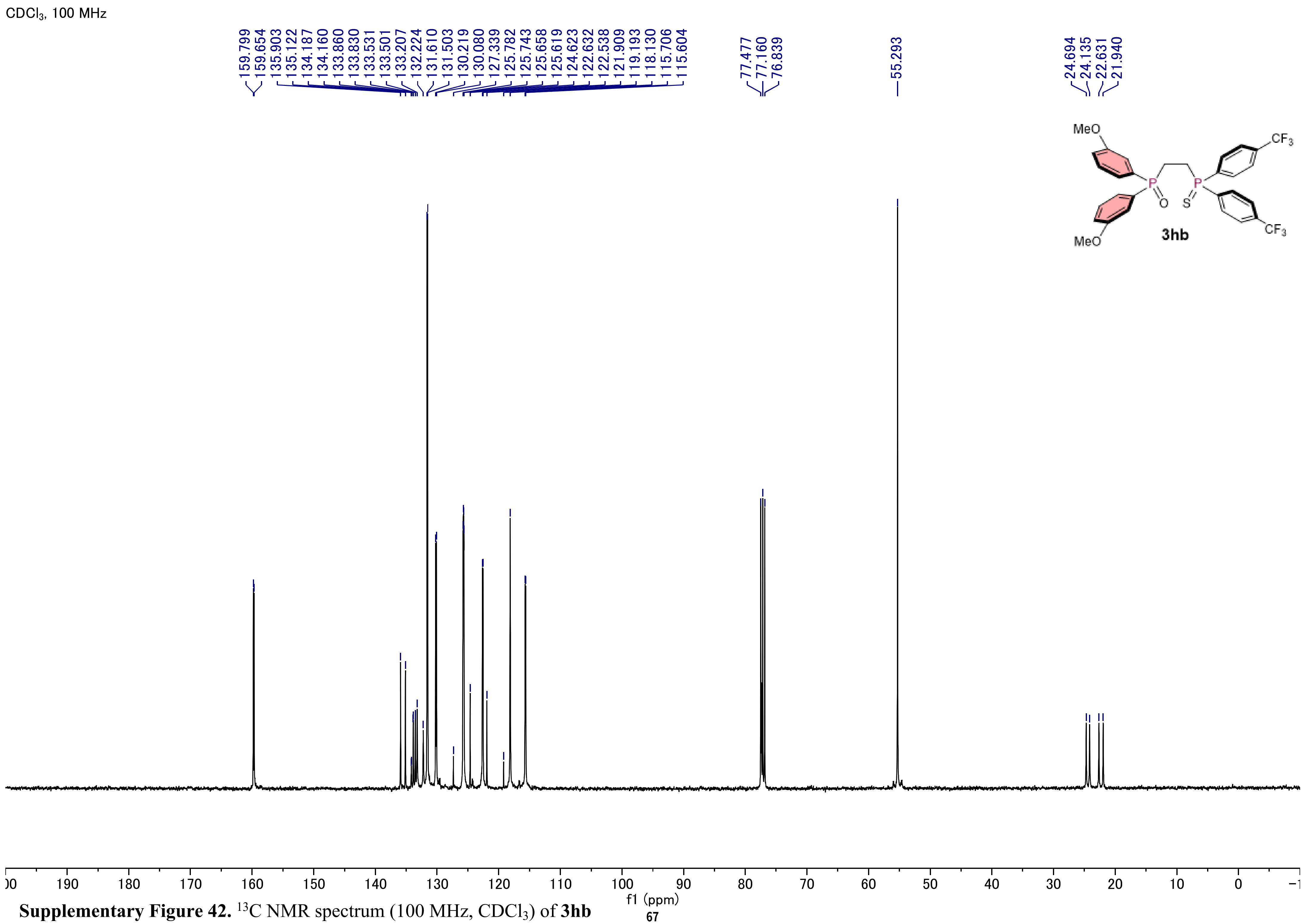

Supplementary Figure 42. ^{13}C NMR spectrum (100 MHz, $CDCl_3$) of **3hb**

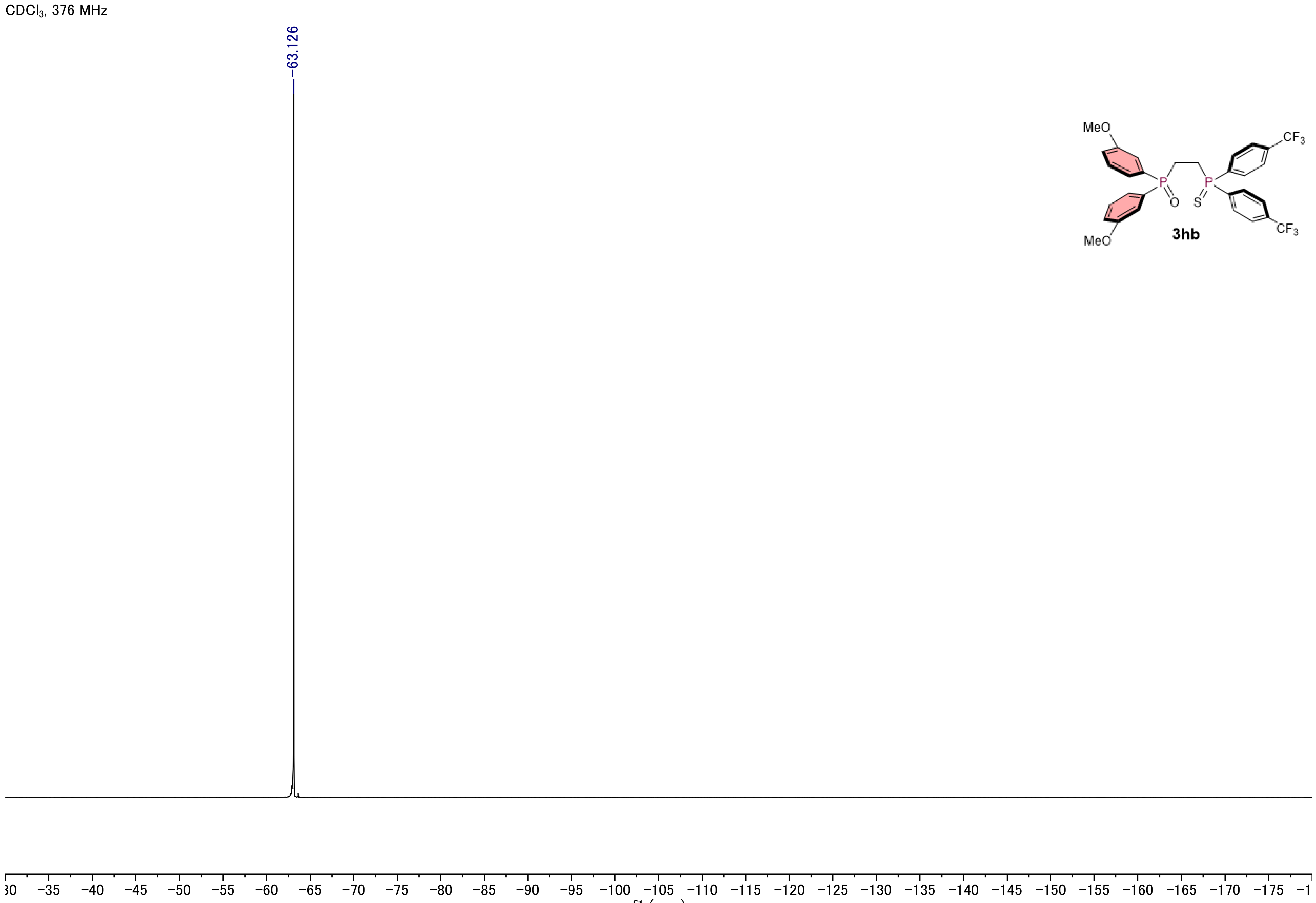

Supplementary Figure 43. ^{19}F NMR spectrum (376 MHz, $CDCl_3$) of **3hb**

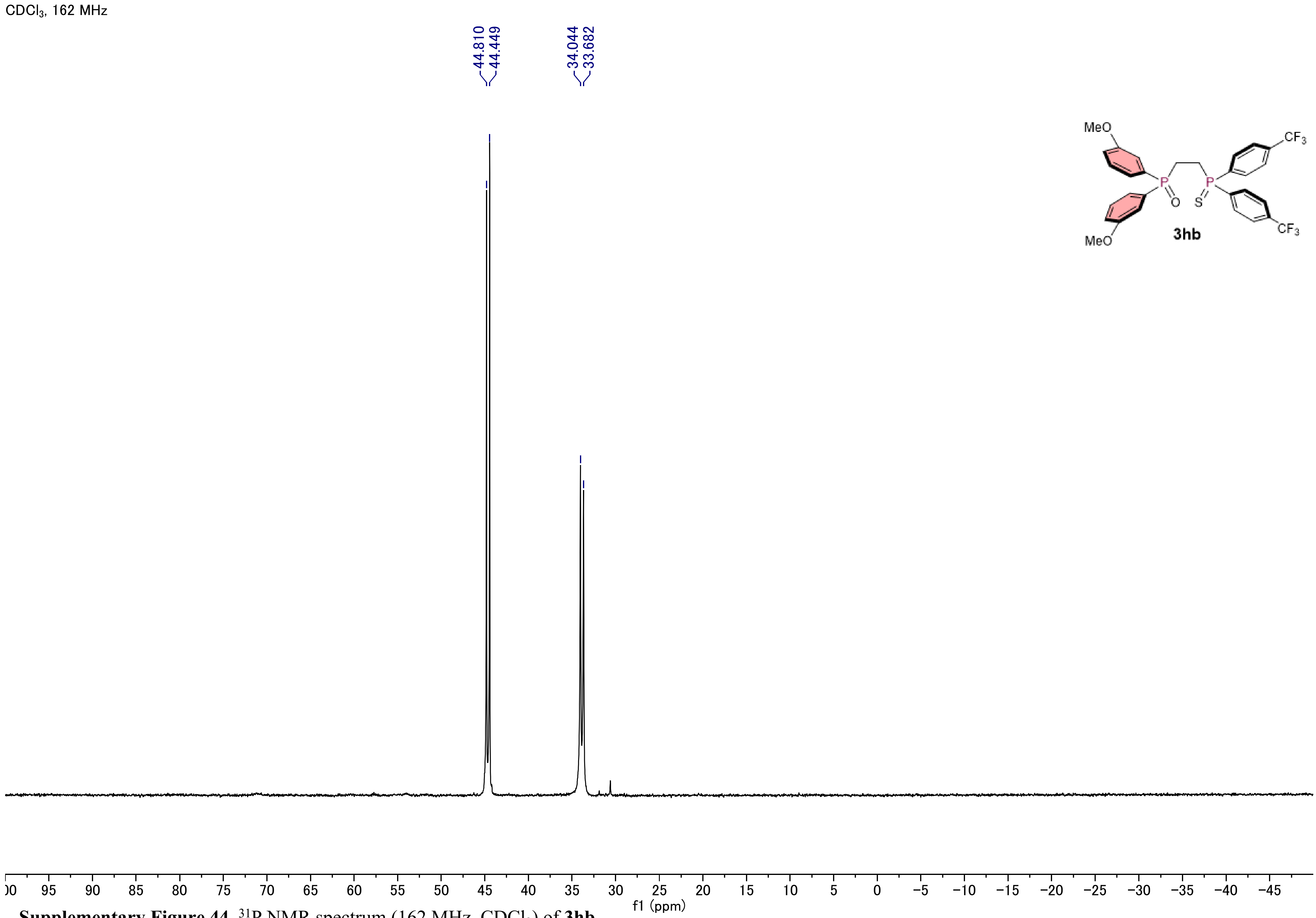

Supplementary Figure 44. ^{31}P NMR spectrum (162 MHz, $CDCl_3$) of **3hb**

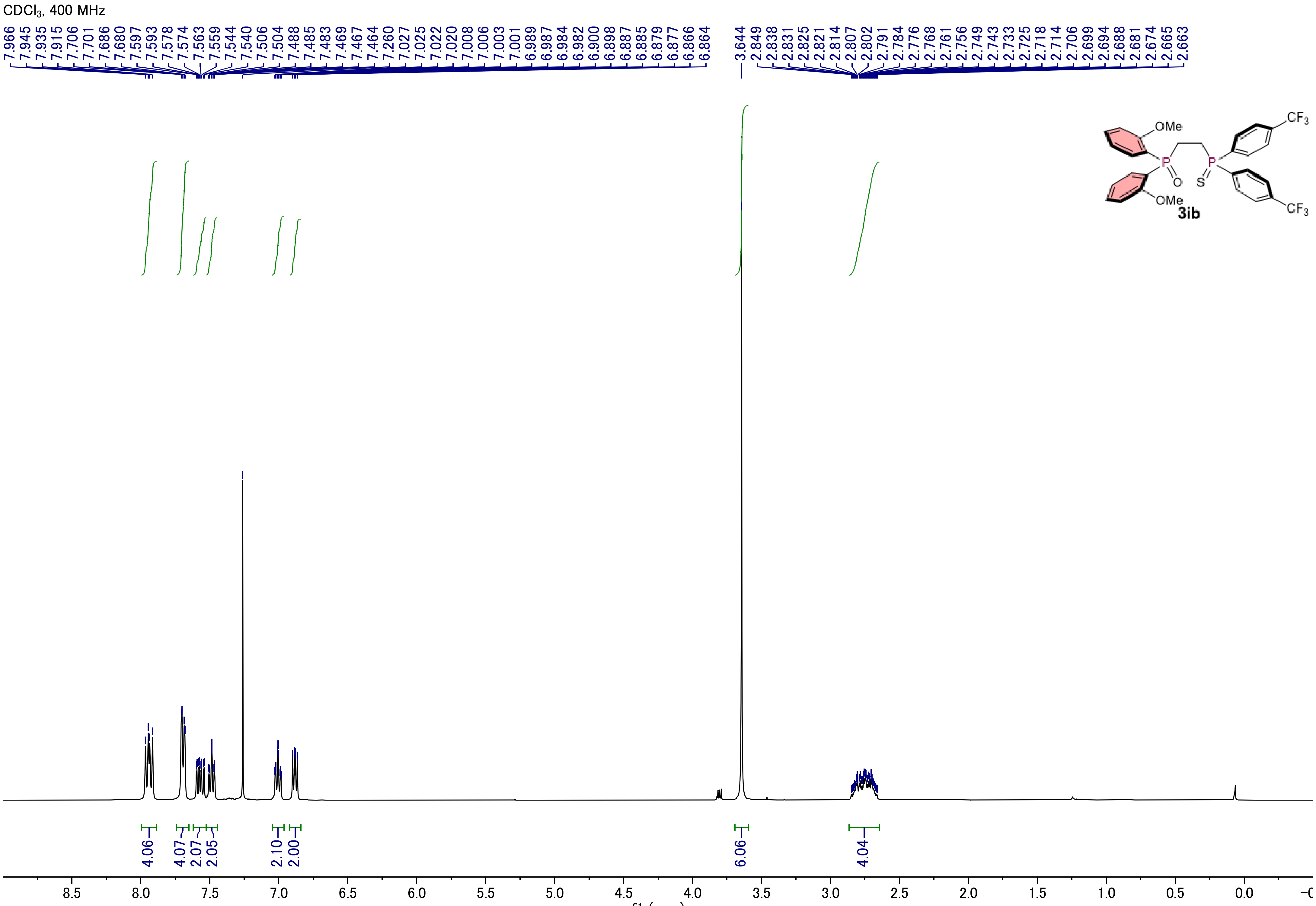

Supplementary Figure 45. ^{1}H NMR spectrum (400 MHz, $CDCl_3$) of **3ib**

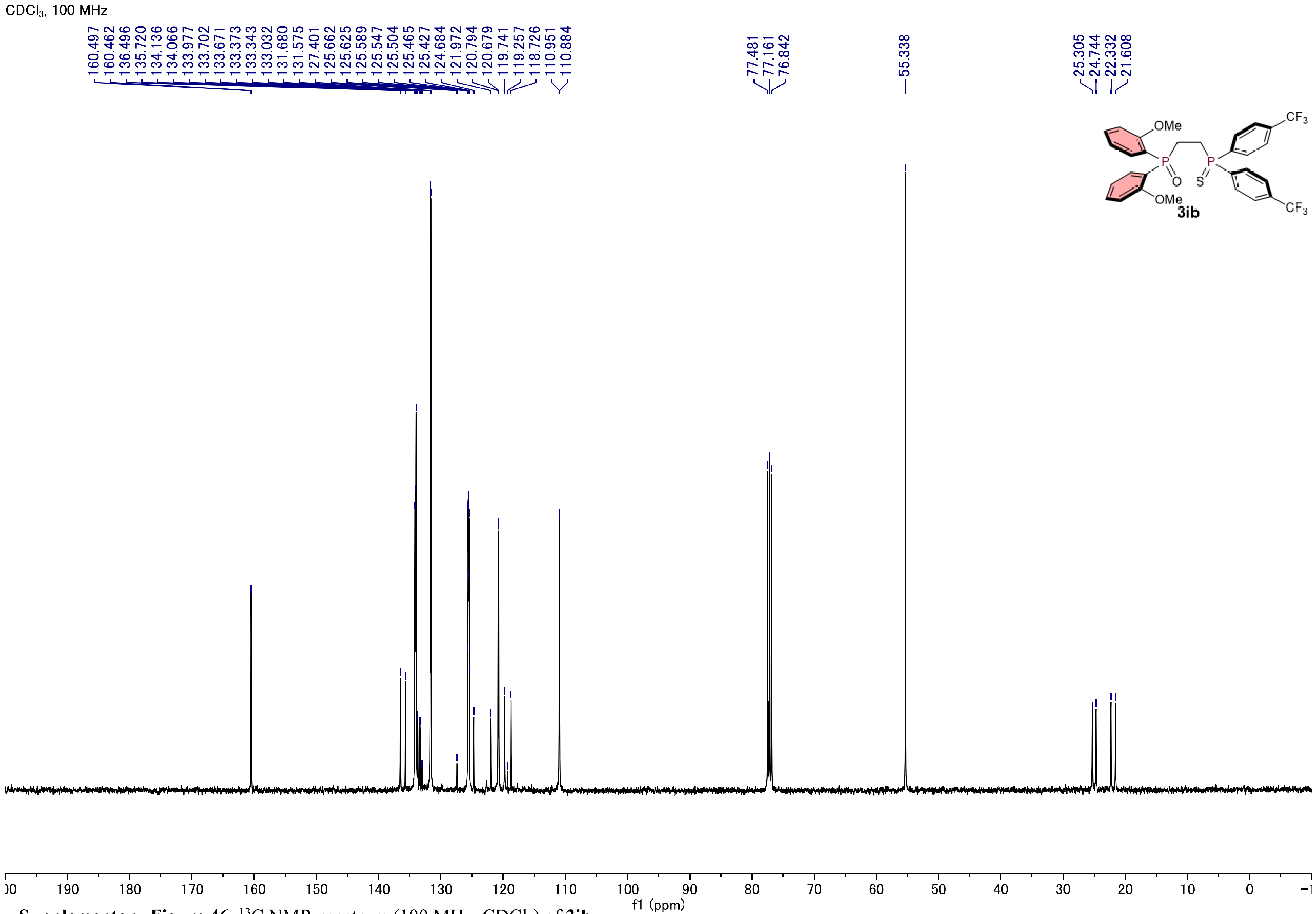

Supplementary Figure 46. ^{13}C NMR spectrum (100 MHz, $CDCl_3$) of **3ib**

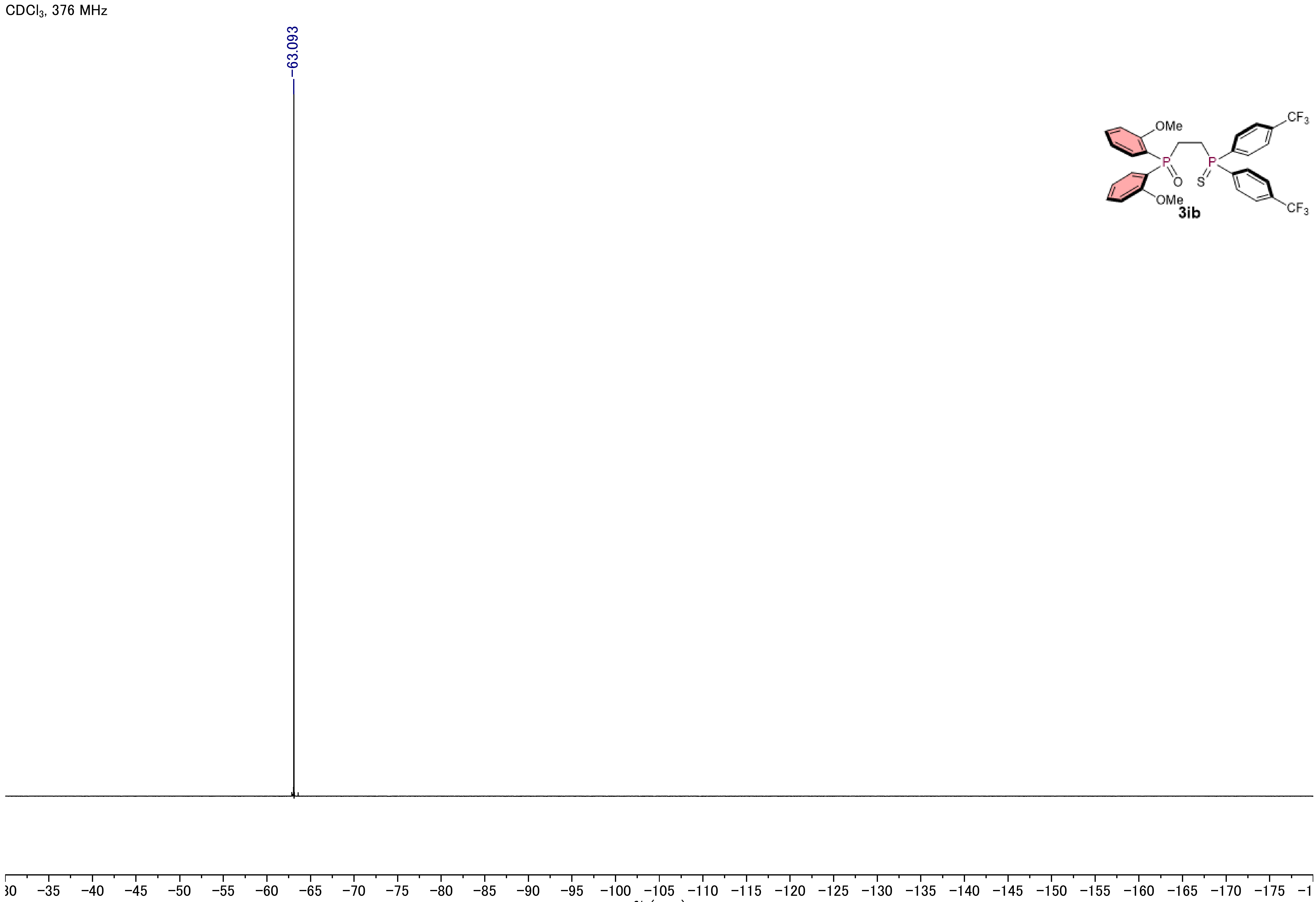

Supplementary Figure 47. ^{19}F NMR spectrum (376 MHz, $CDCl_3$) of **3ib**

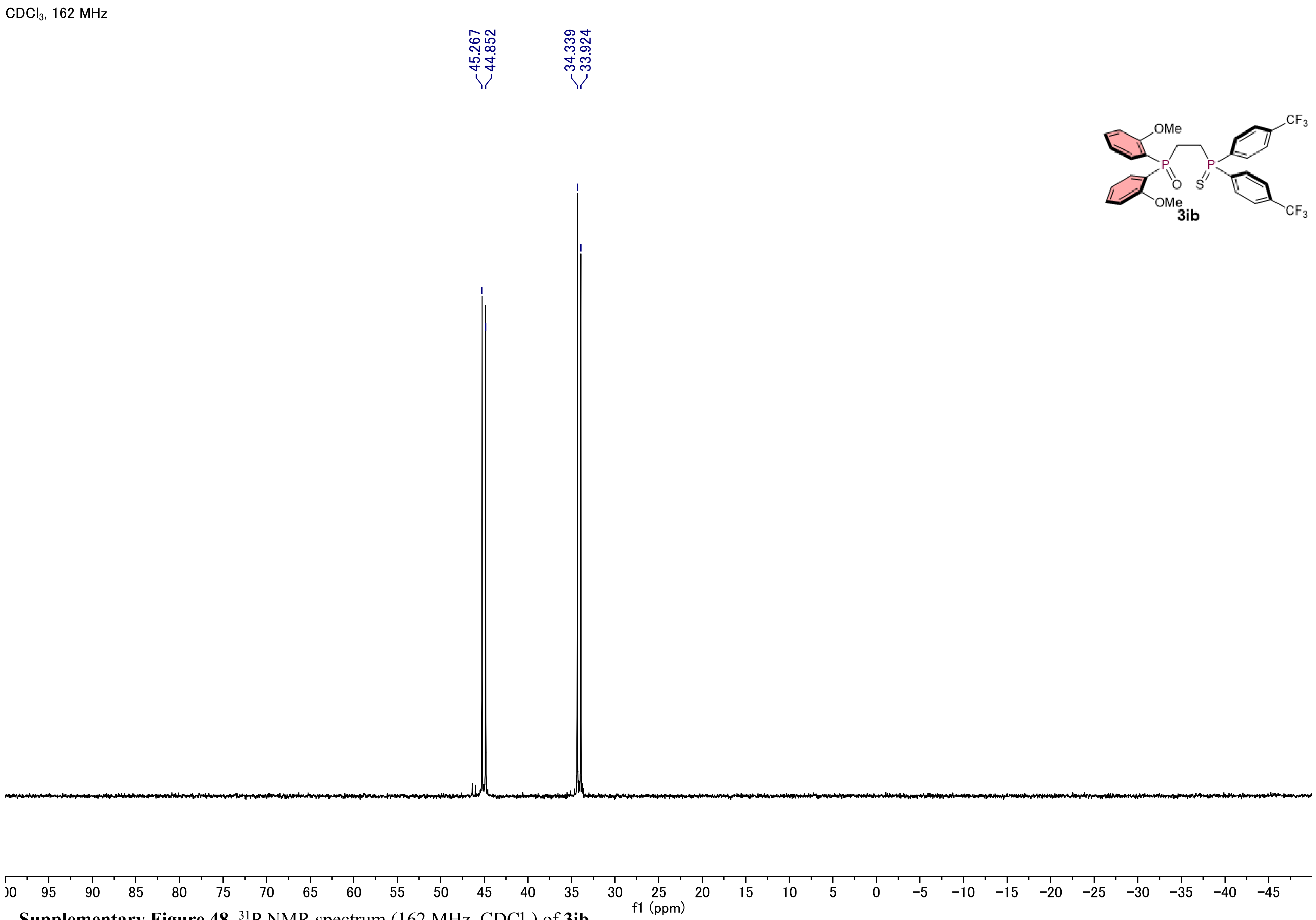

Supplementary Figure 48. ^{31}P NMR spectrum (162 MHz, $CDCl_3$) of **3ib**

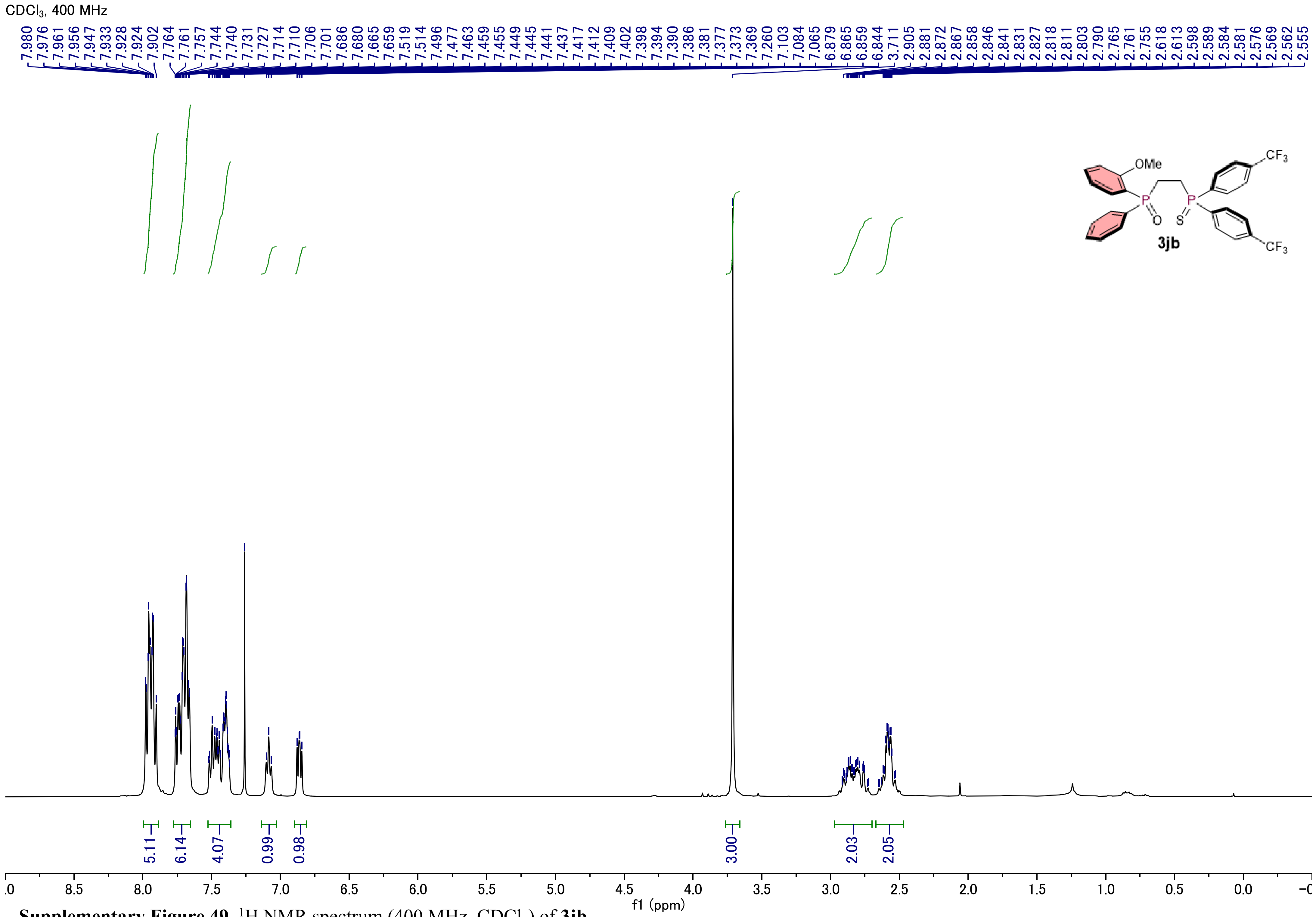

Supplementary Figure 49. ^{1}H NMR spectrum (400 MHz, $CDCl_3$) of **3jb**

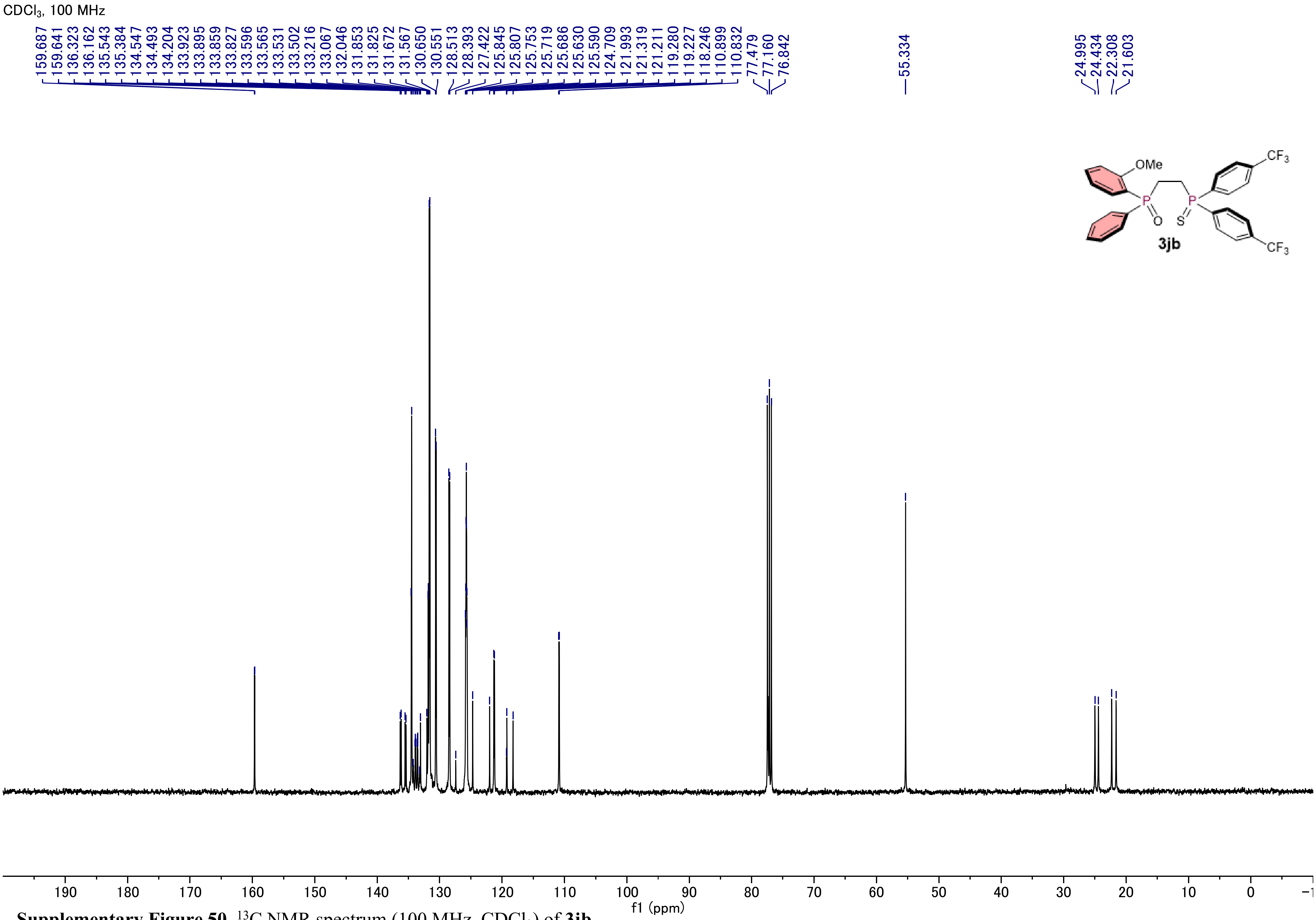

Supplementary Figure 50. ^{13}C NMR spectrum (100 MHz, $CDCl_3$) of **3jb**

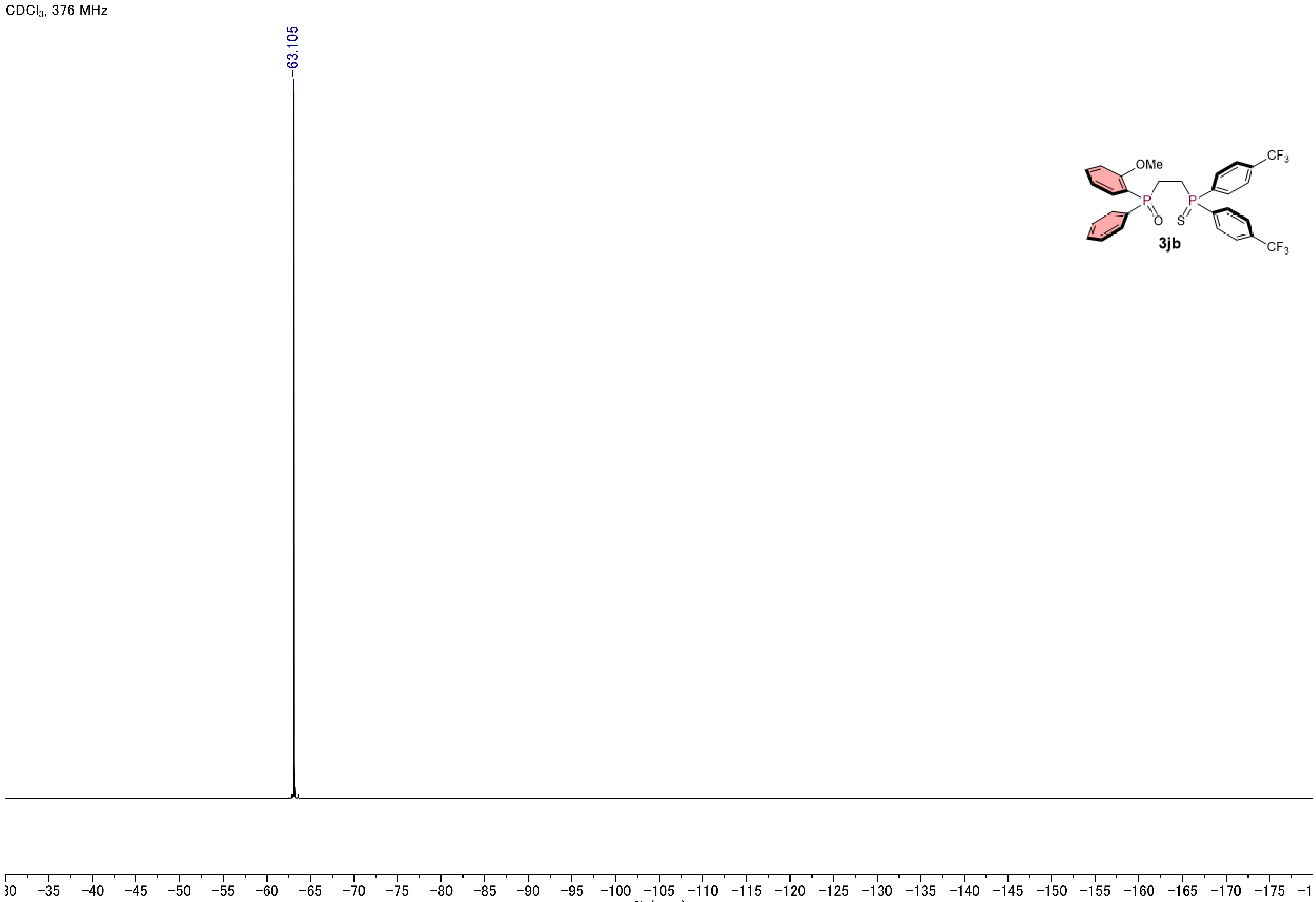

Supplementary Figure 51. ^{19}F NMR spectrum (376 MHz, $CDCl_3$) of **3jb**

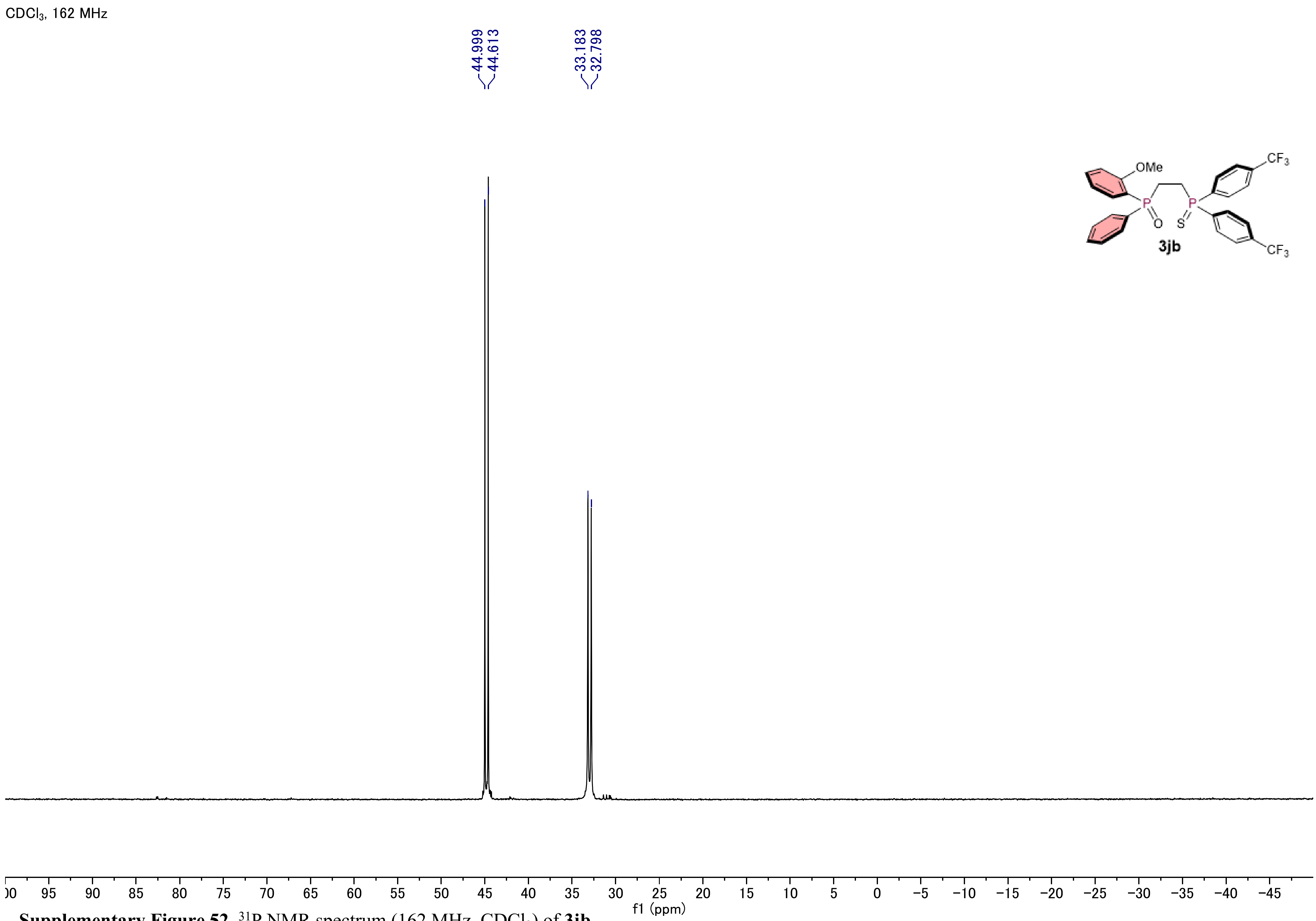

Supplementary Figure 52. ^{31}P NMR spectrum (162 MHz, $CDCl_3$) of **3jb**

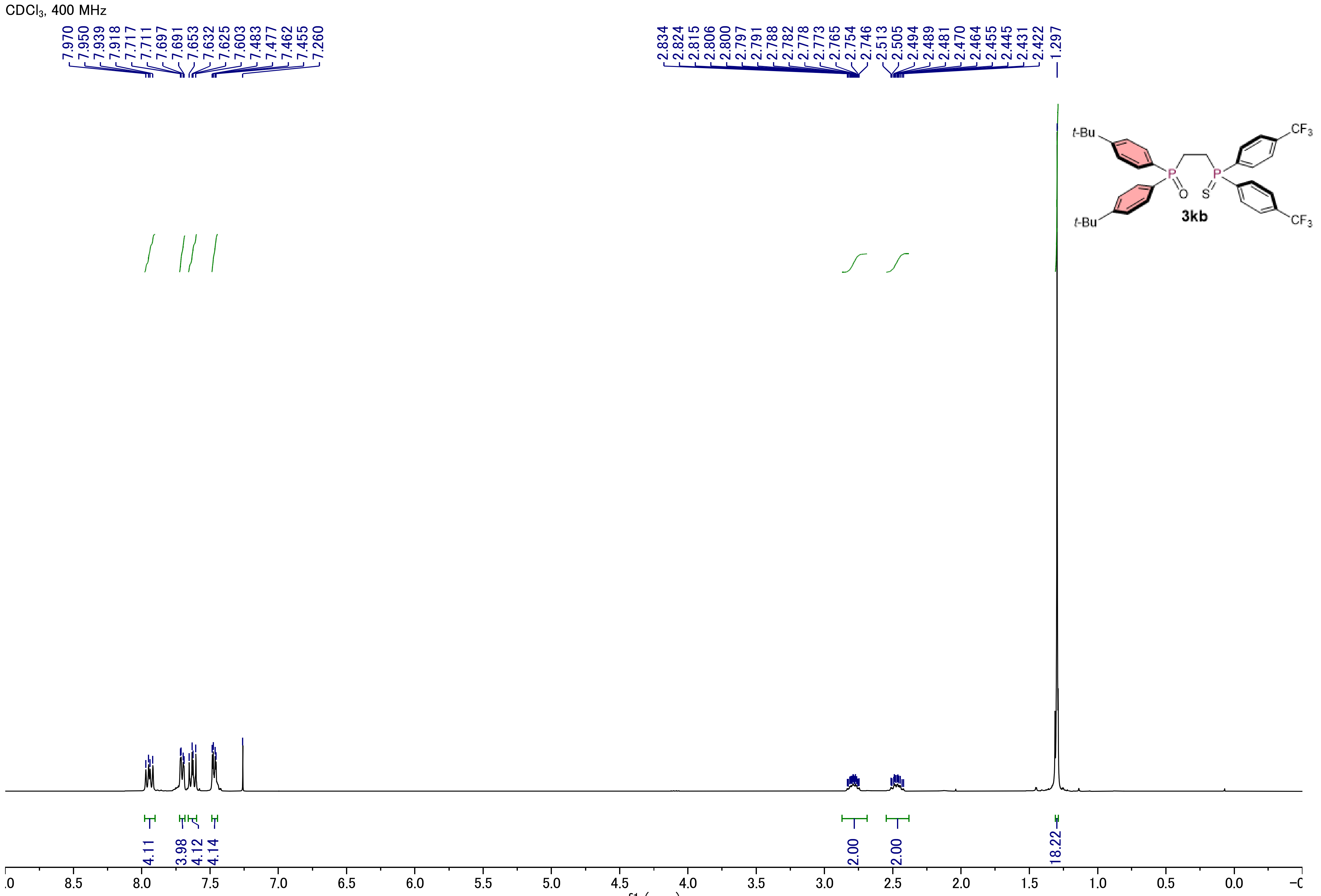

Supplementary Figure 53. ^{1}H NMR spectrum (400 MHz, $CDCl_3$) of **3kb**

CDCl$_3$, 100 MHz

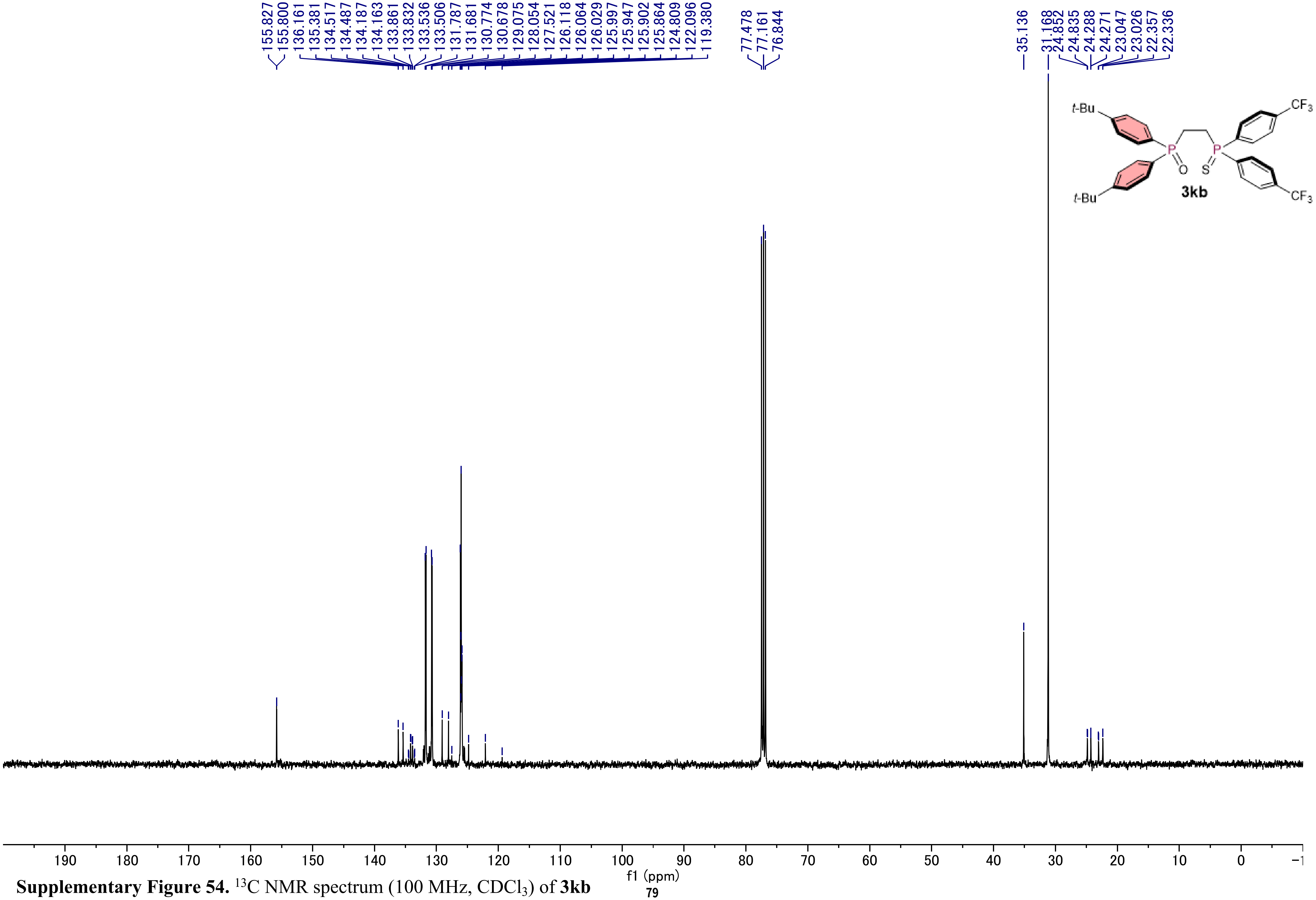

Supplementary Figure 54. ^{13}C NMR spectrum (100 MHz, CDCl$_3$) of **3kb**

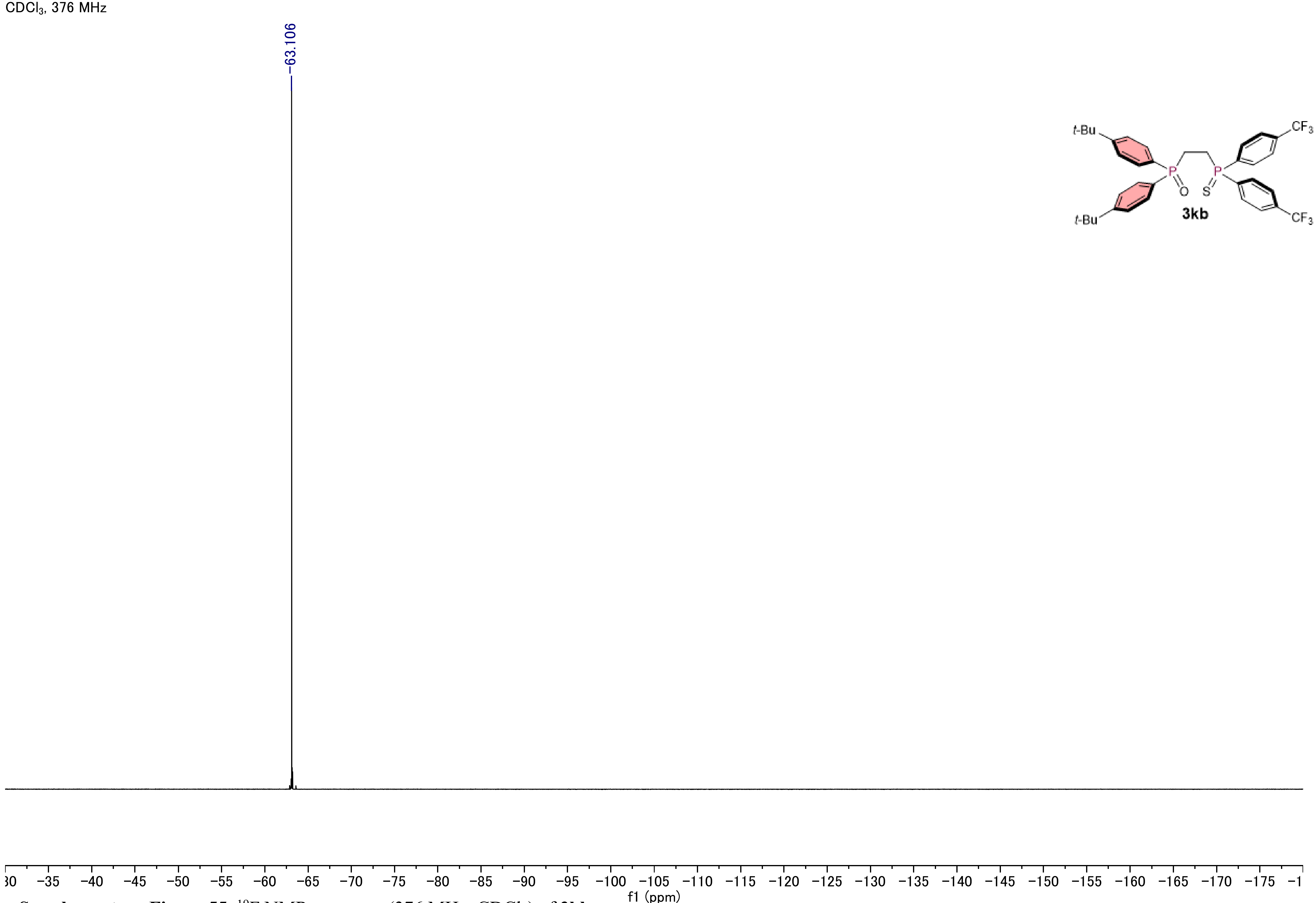

Supplementary Figure 55. ^{19}F NMR spectrum (376 MHz, $CDCl_3$) of **3kb**

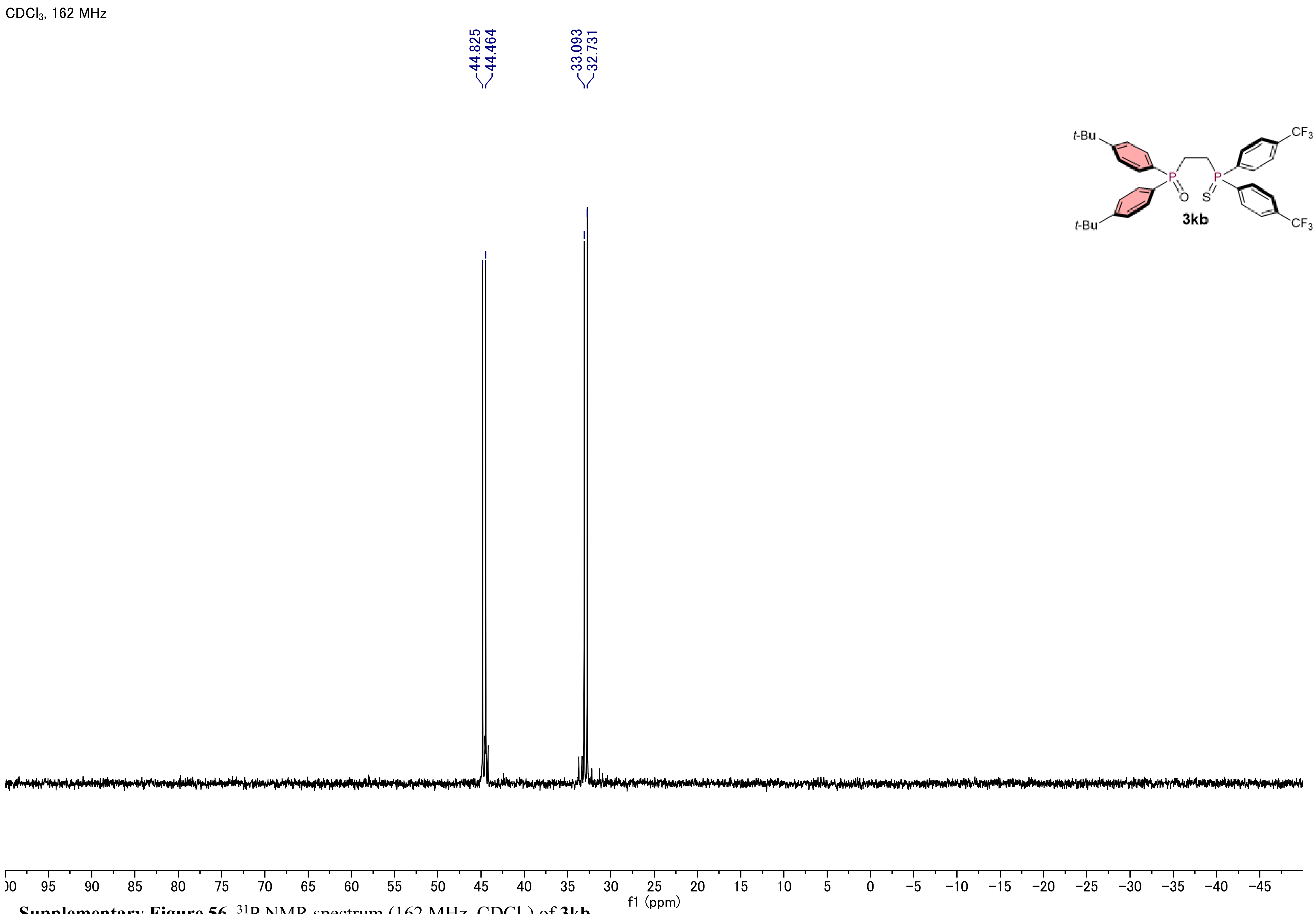

Supplementary Figure 56. ^{31}P NMR spectrum (162 MHz, $CDCl_3$) of **3kb**

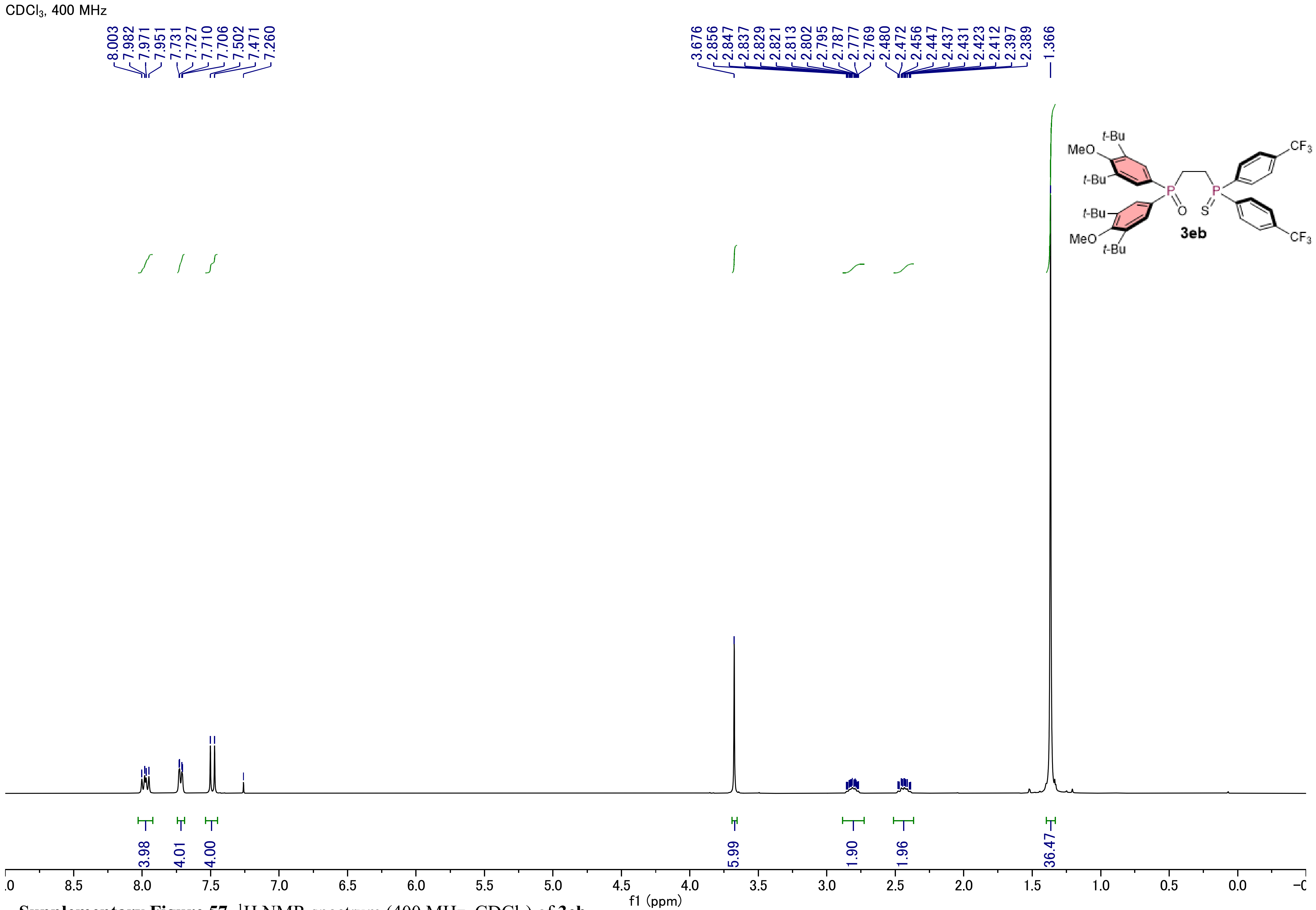

Supplementary Figure 57. ^{1}H NMR spectrum (400 MHz, $CDCl_3$) of **3eb**

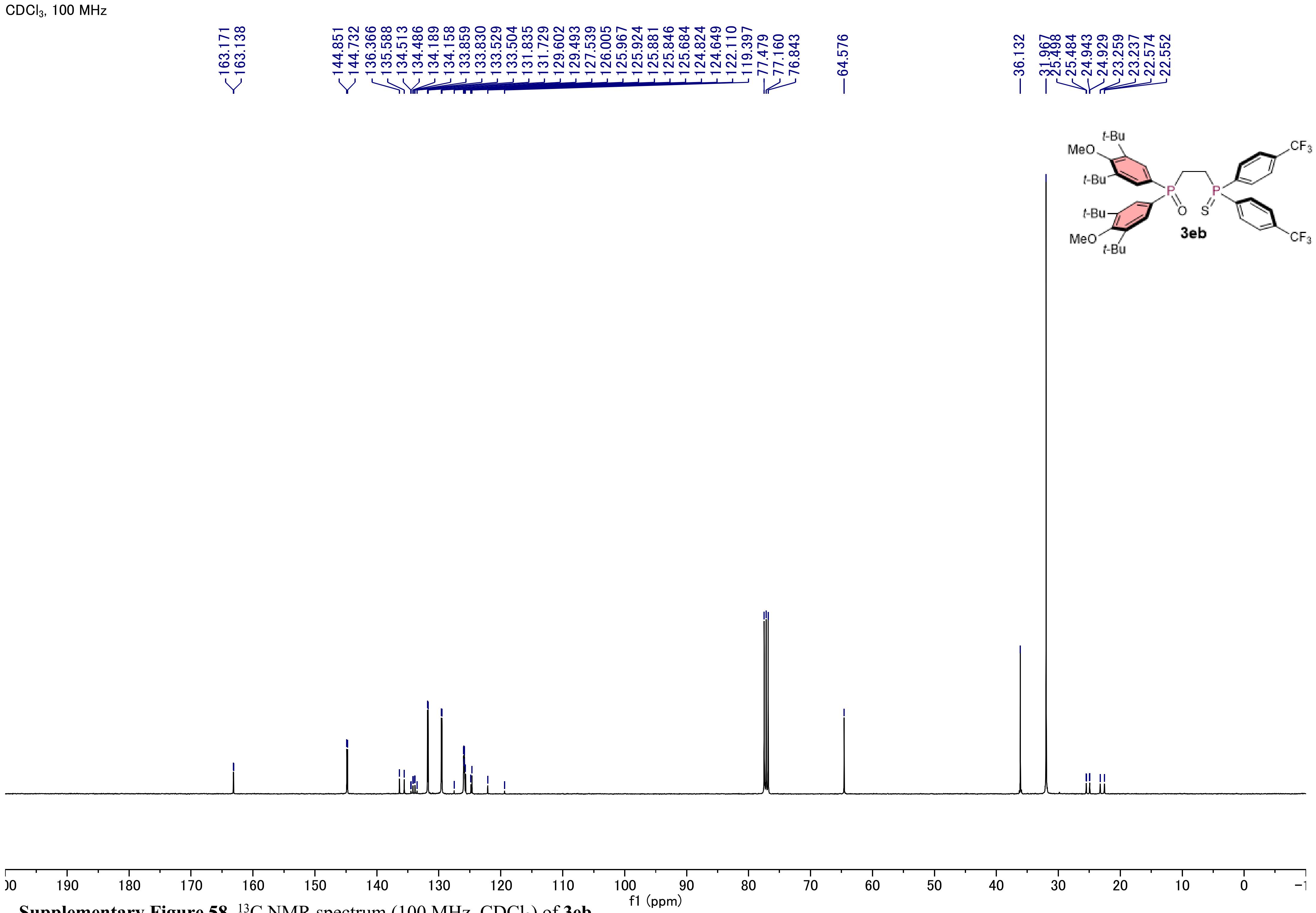

Supplementary Figure 58. ^{13}C NMR spectrum (100 MHz, $CDCl_3$) of **3eb**

CDCl$_3$, 376 MHz

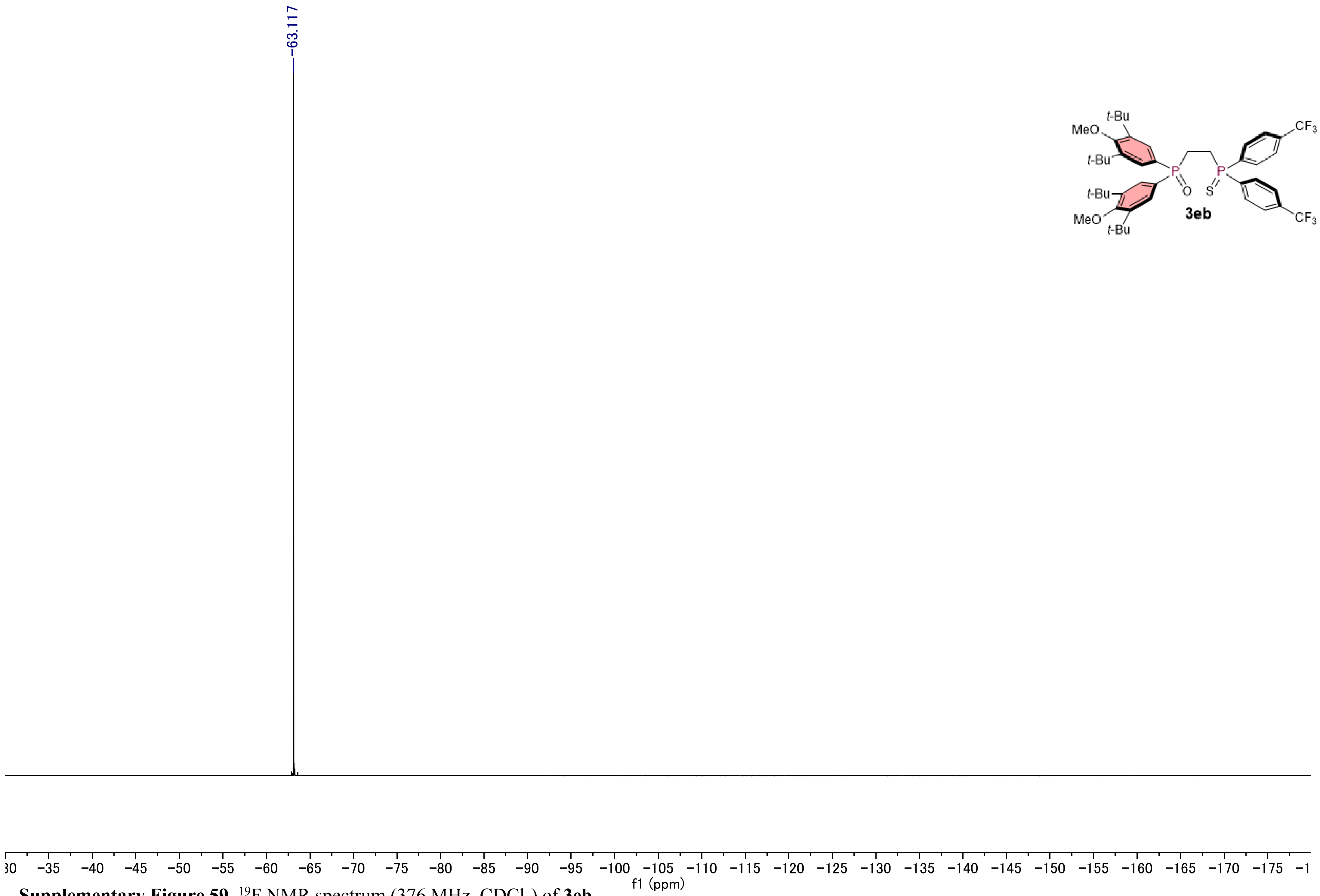

3eb

Supplementary Figure 59. ^{19}F NMR spectrum (376 MHz, CDCl$_3$) of **3eb**

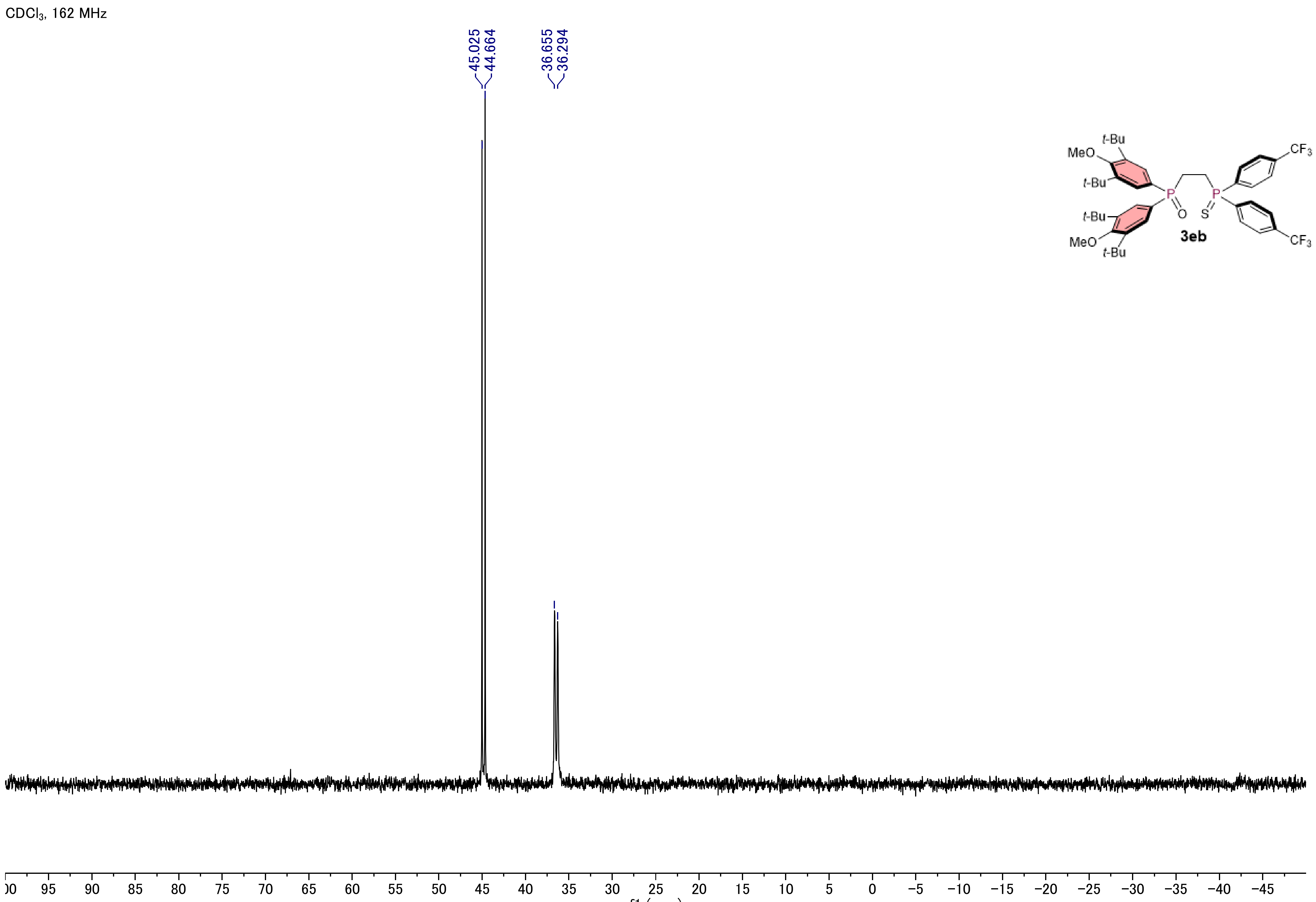

Supplementary Figure 60. ^{31}P NMR spectrum (162 MHz, $CDCl_3$) of **3eb**

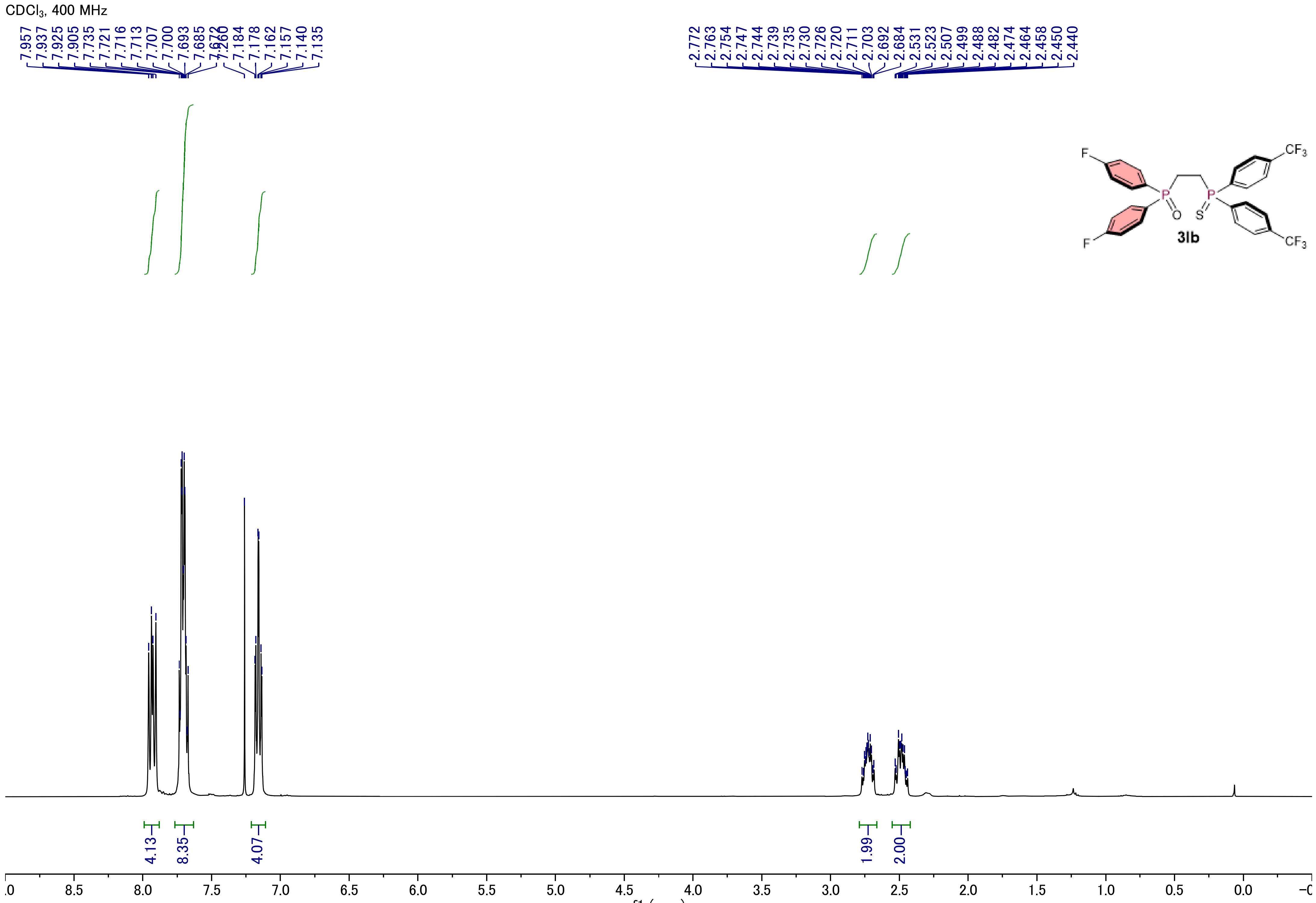

Supplementary Figure 61. ^{1}H NMR spectrum (400 MHz, $CDCl_3$) of **3lb**

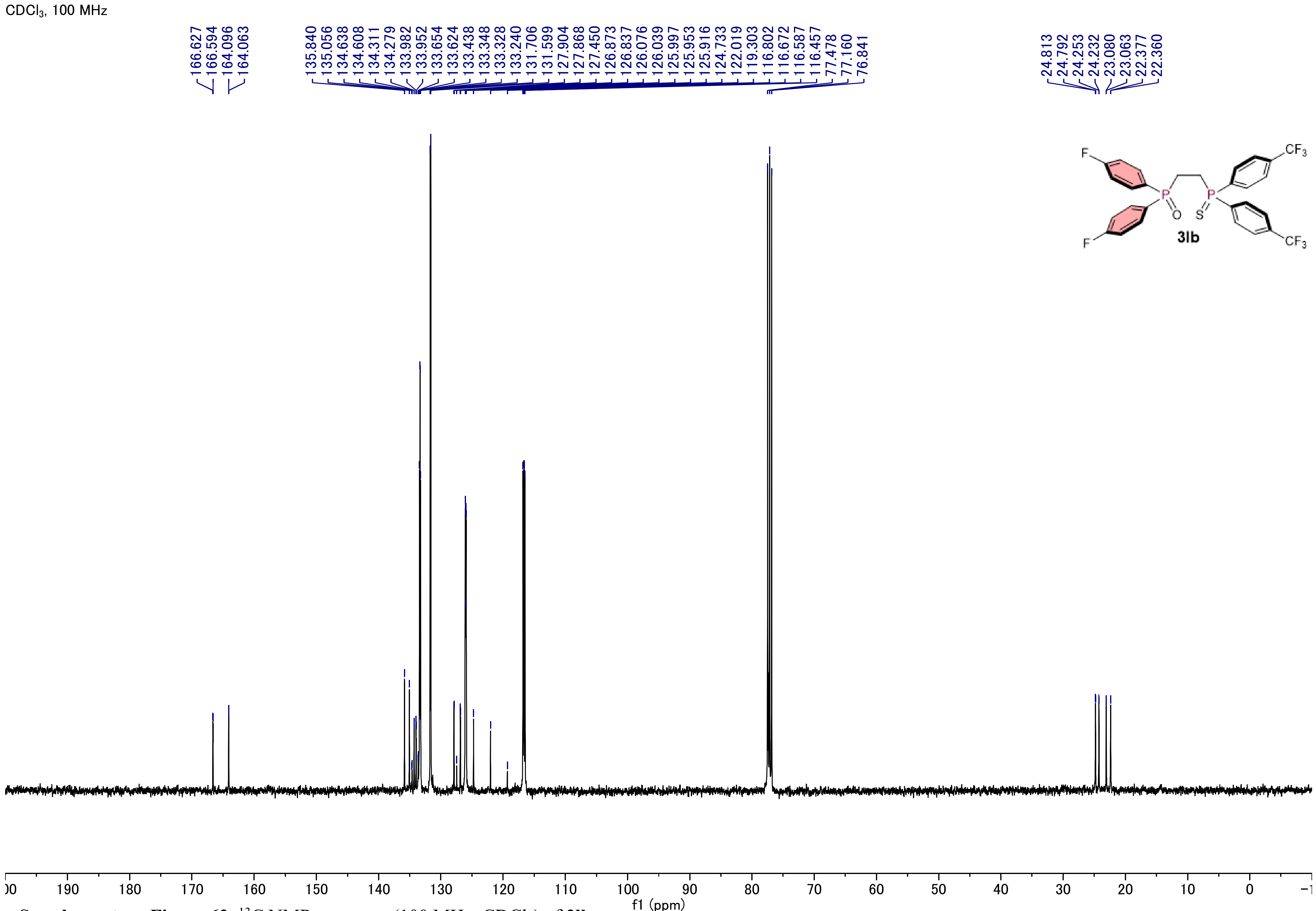

Supplementary Figure 62. ^{13}C NMR spectrum (100 MHz, CDCl$_3$) of **3lb**

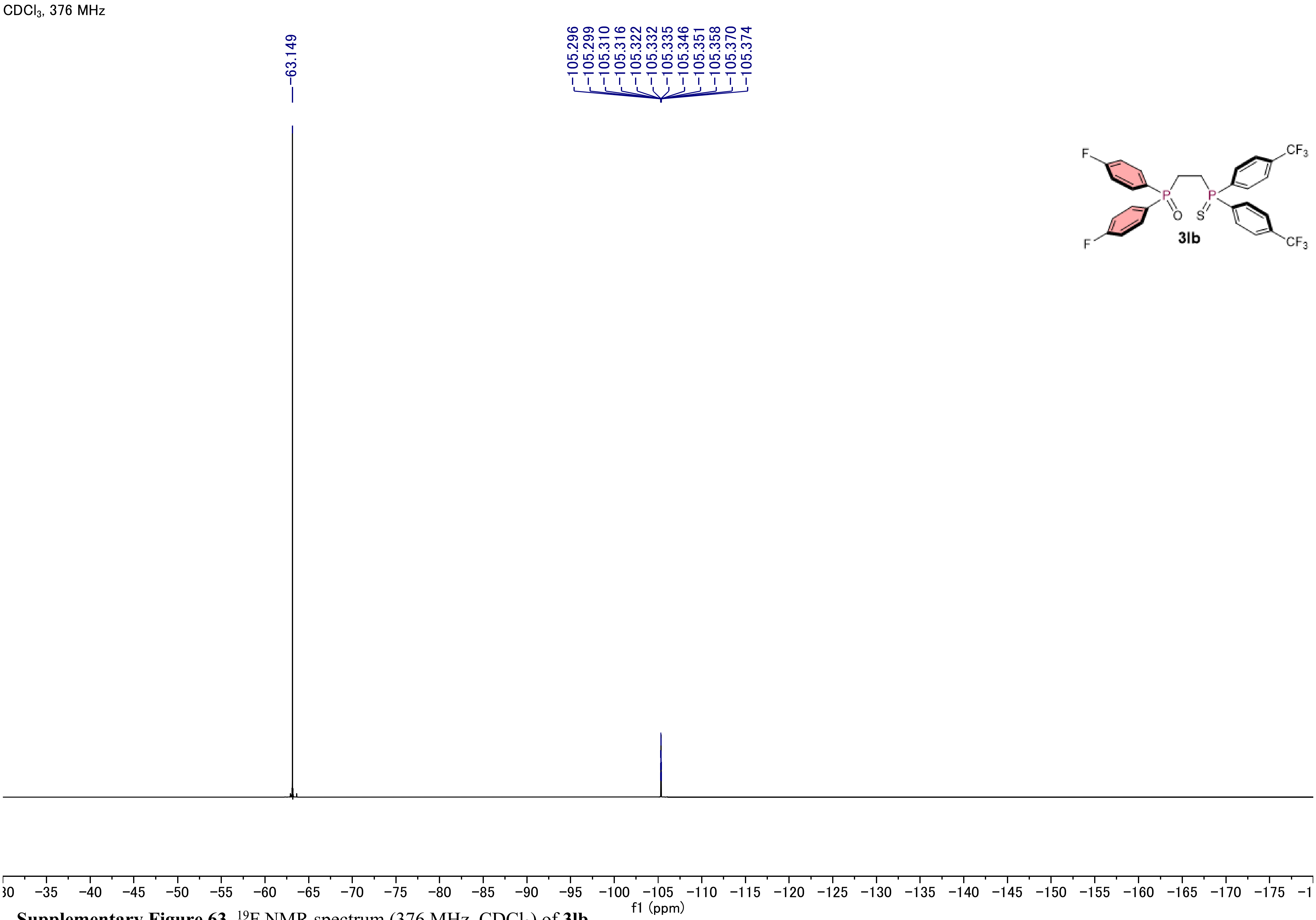

Supplementary Figure 63. ^{19}F NMR spectrum (376 MHz, $CDCl_3$) of **3lb**

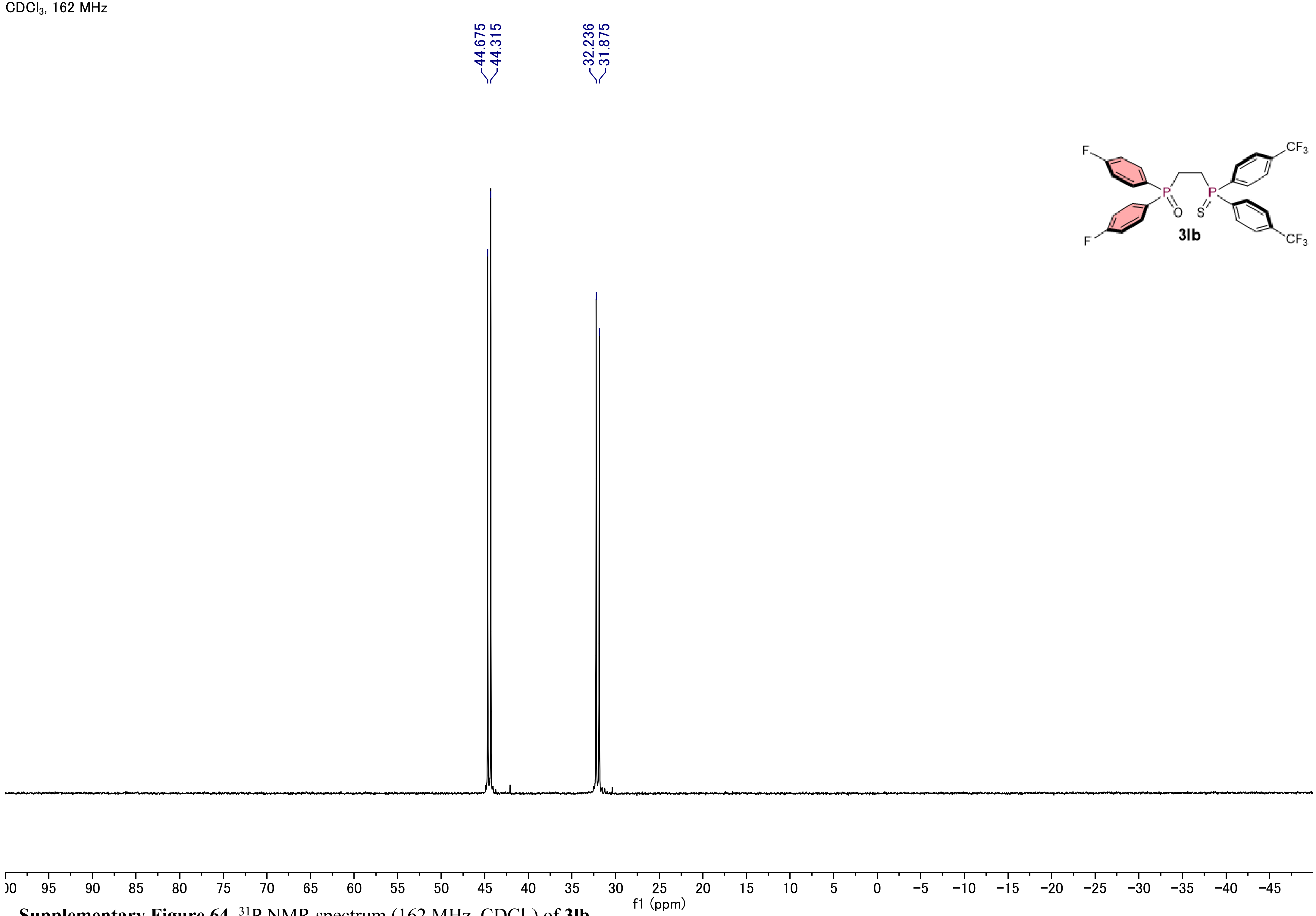

Supplementary Figure 64. ^{31}P NMR spectrum (162 MHz, $CDCl_3$) of **3lb**

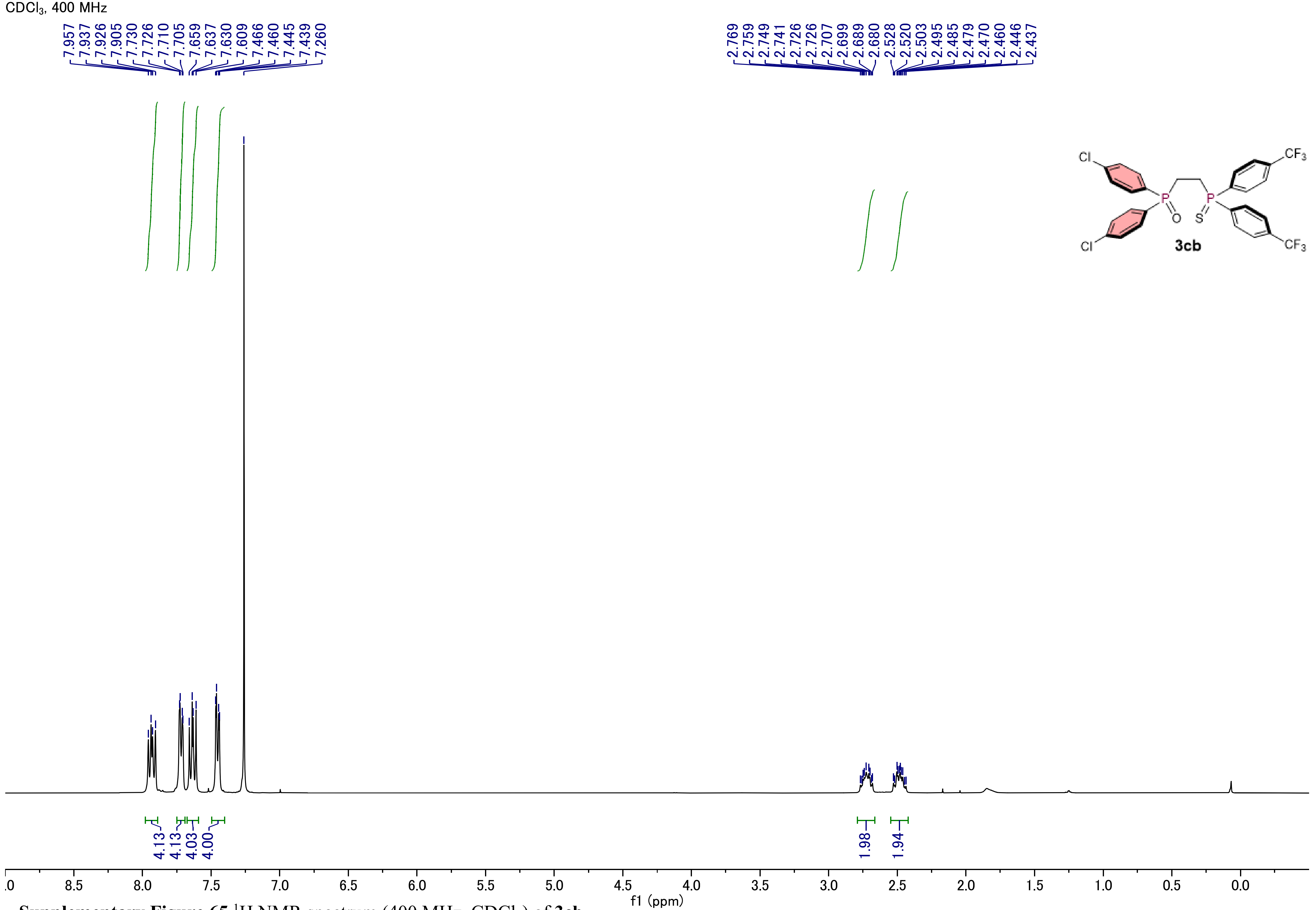

Supplementary Figure 65 ^{1}H NMR spectrum (400 MHz, $CDCl_3$) of **3cb**

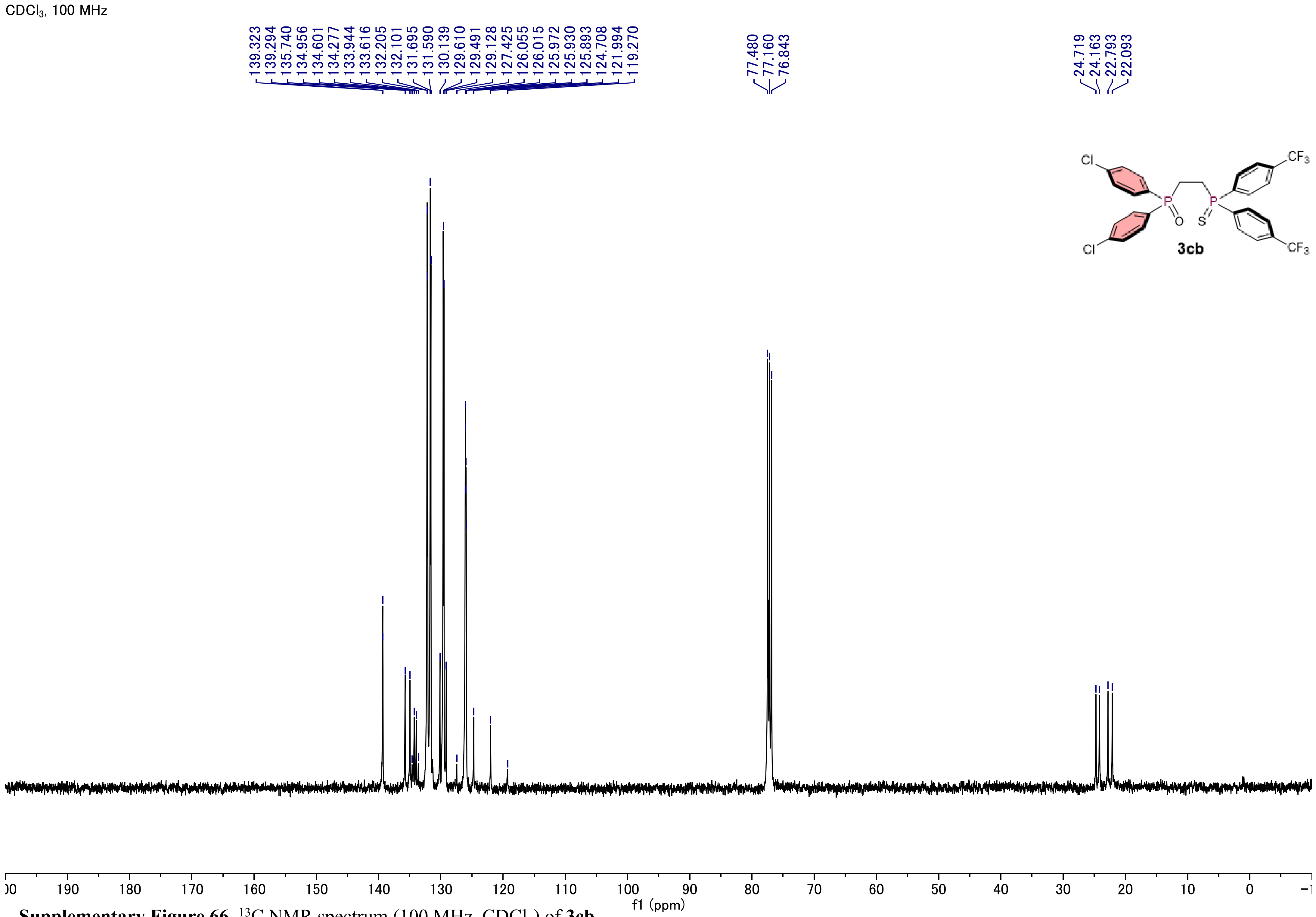

Supplementary Figure 66. ^{13}C NMR spectrum (100 MHz, CDCl$_3$) of **3cb**

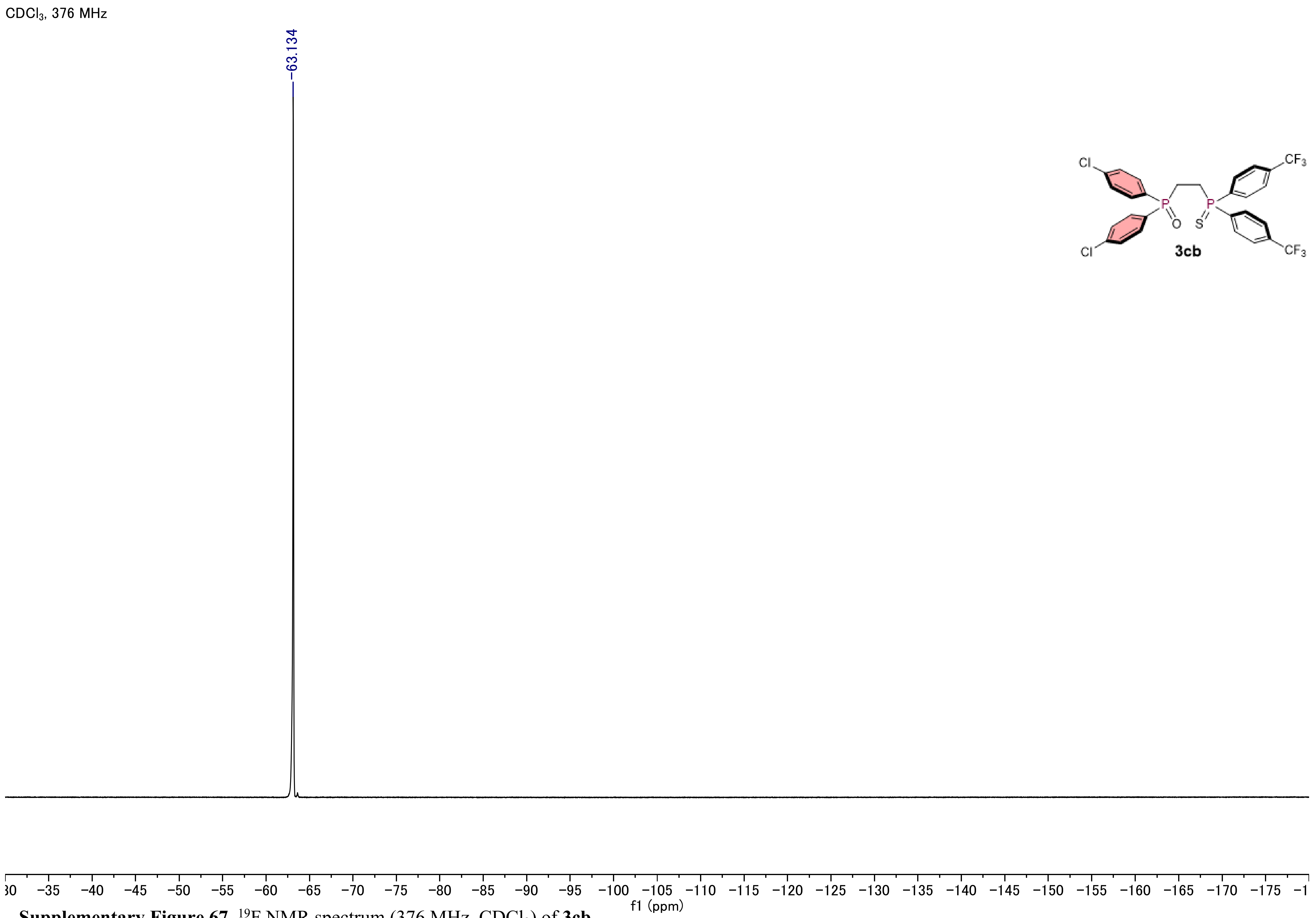

Supplementary Figure 67. ^{19}F NMR spectrum (376 MHz, $CDCl_3$) of **3cb**

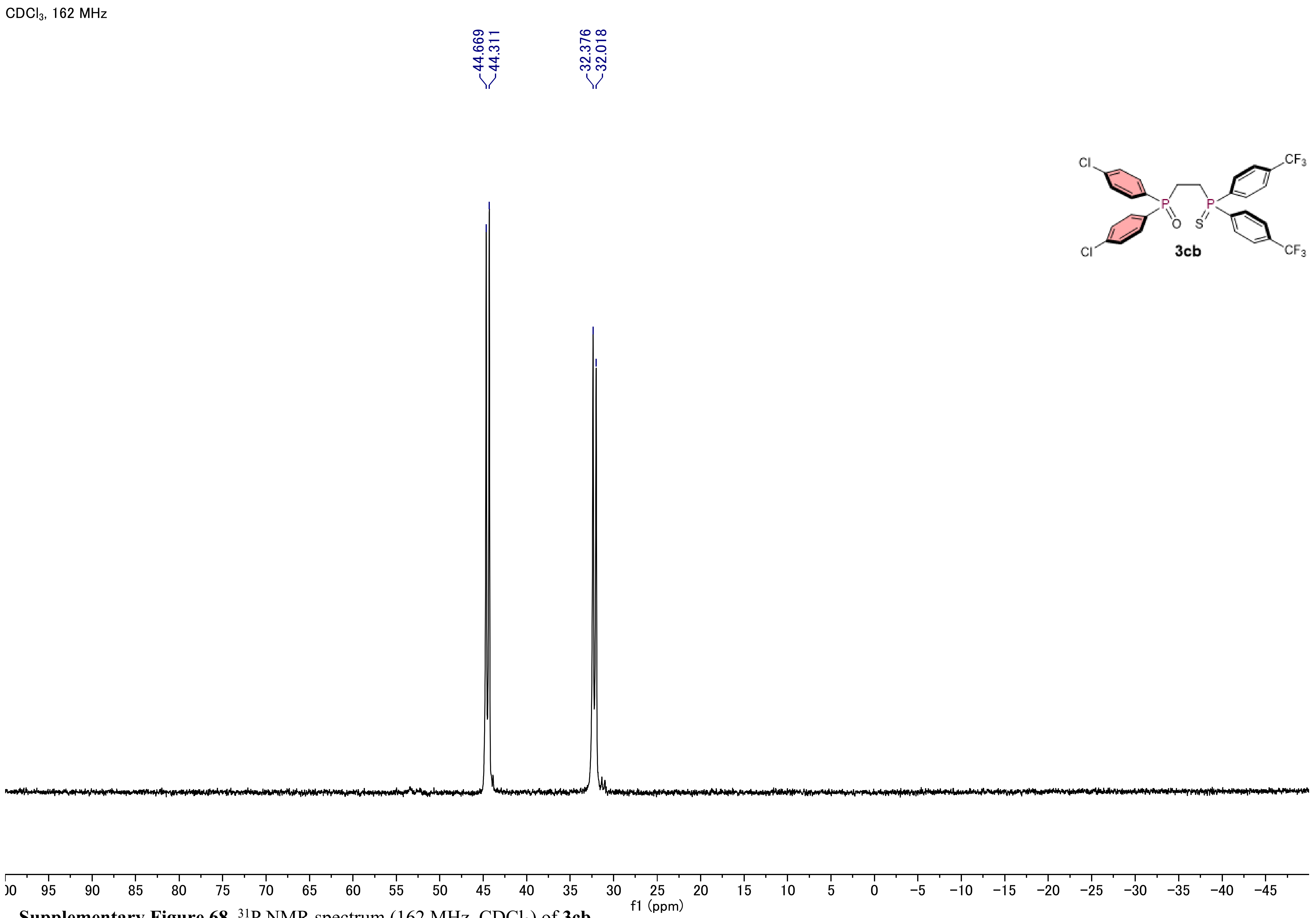

Supplementary Figure 68. ^{31}P NMR spectrum (162 MHz, $CDCl_3$) of **3cb**

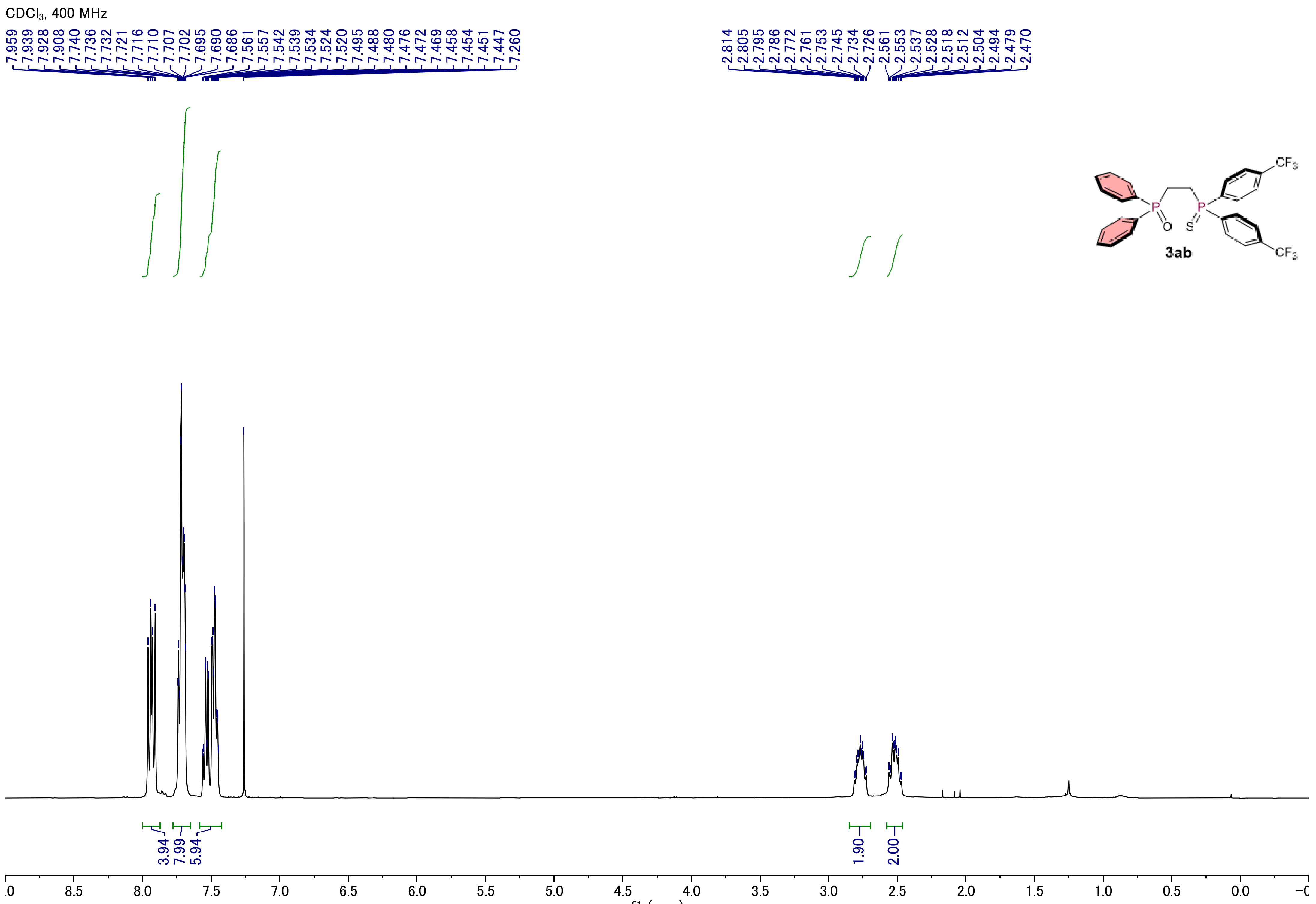

Supplementary Figure 69. ^{1}H NMR spectrum (400 MHz, $CDCl_3$) of **3ab**

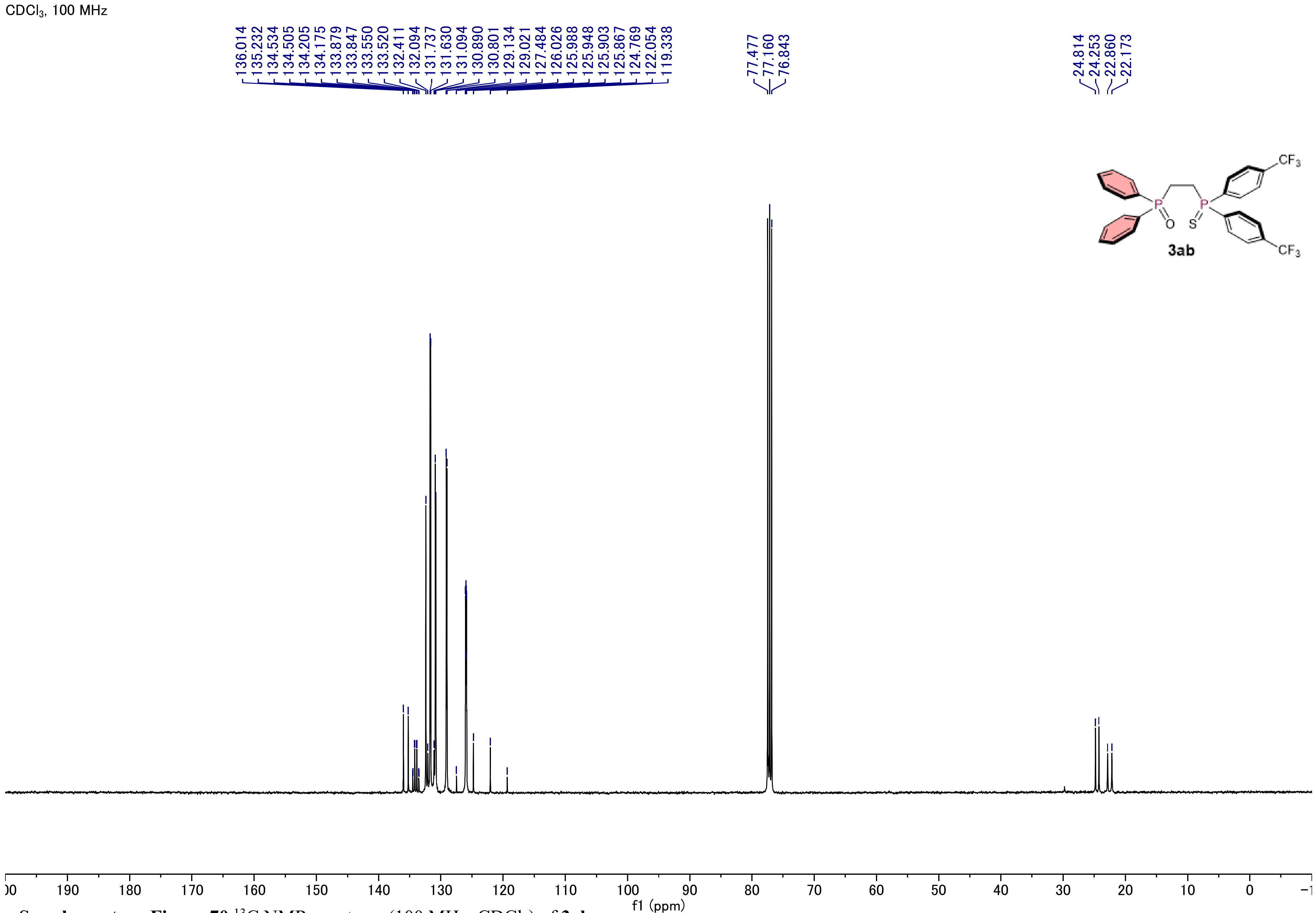

Supplementary Figure 70 ^{13}C NMR spectrum (100 MHz, $CDCl_3$) of **3ab**

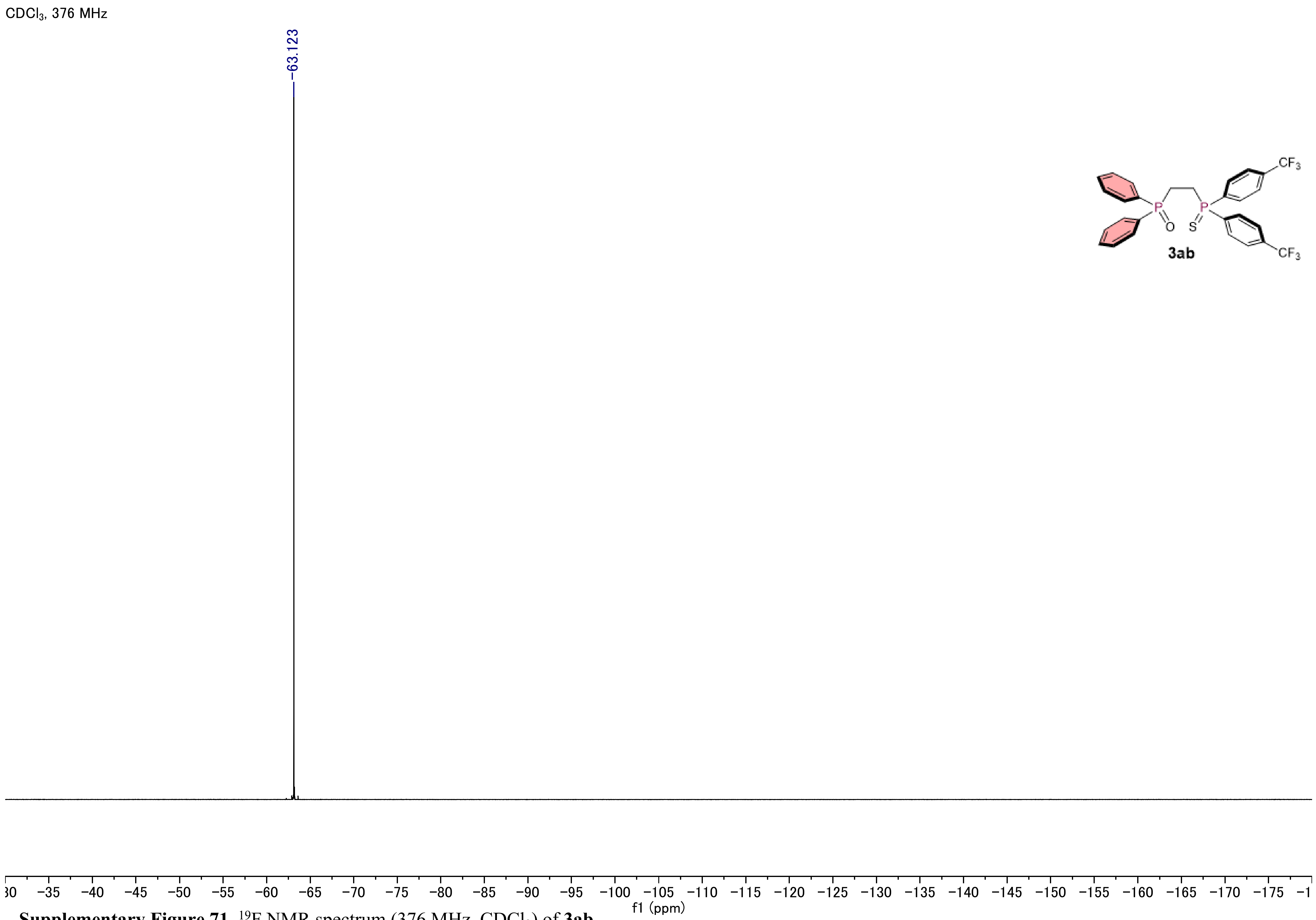

Supplementary Figure 71. ^{19}F NMR spectrum (376 MHz, $CDCl_3$) of **3ab**

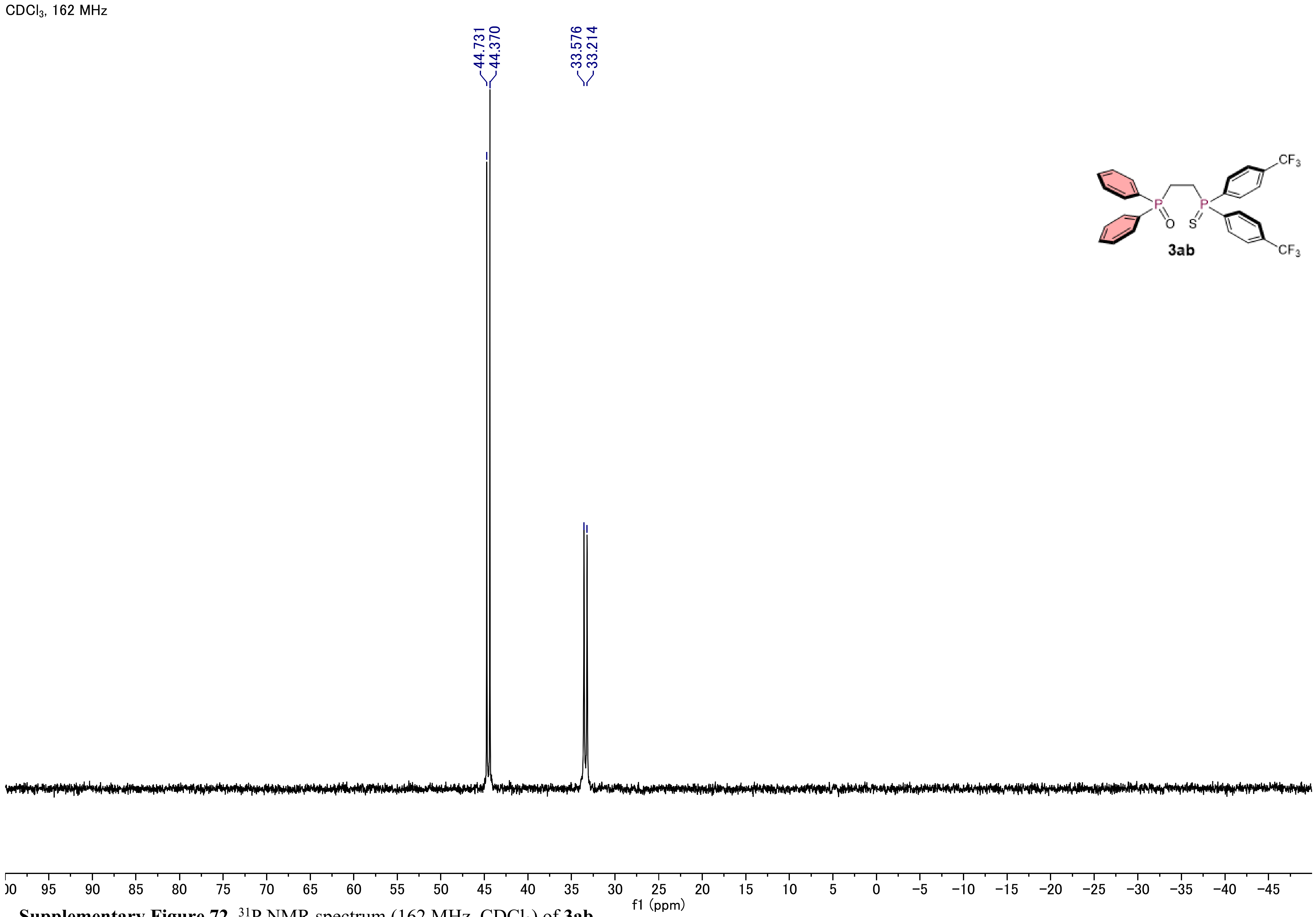

Supplementary Figure 72. ^{31}P NMR spectrum (162 MHz, $CDCl_3$) of **3ab**

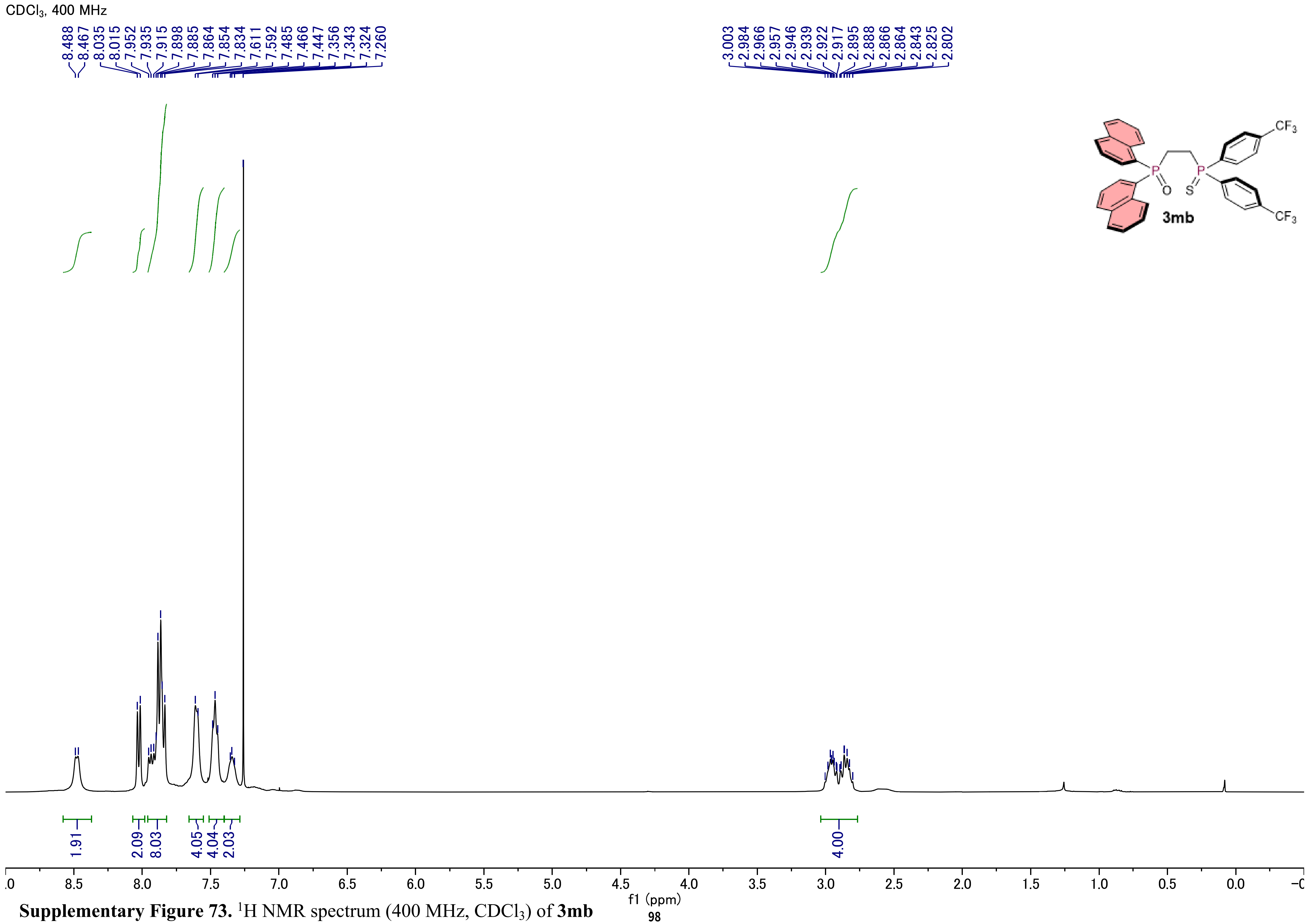

Supplementary Figure 73. ^{1}H NMR spectrum (400 MHz, CDCl$_3$) of **3mb**

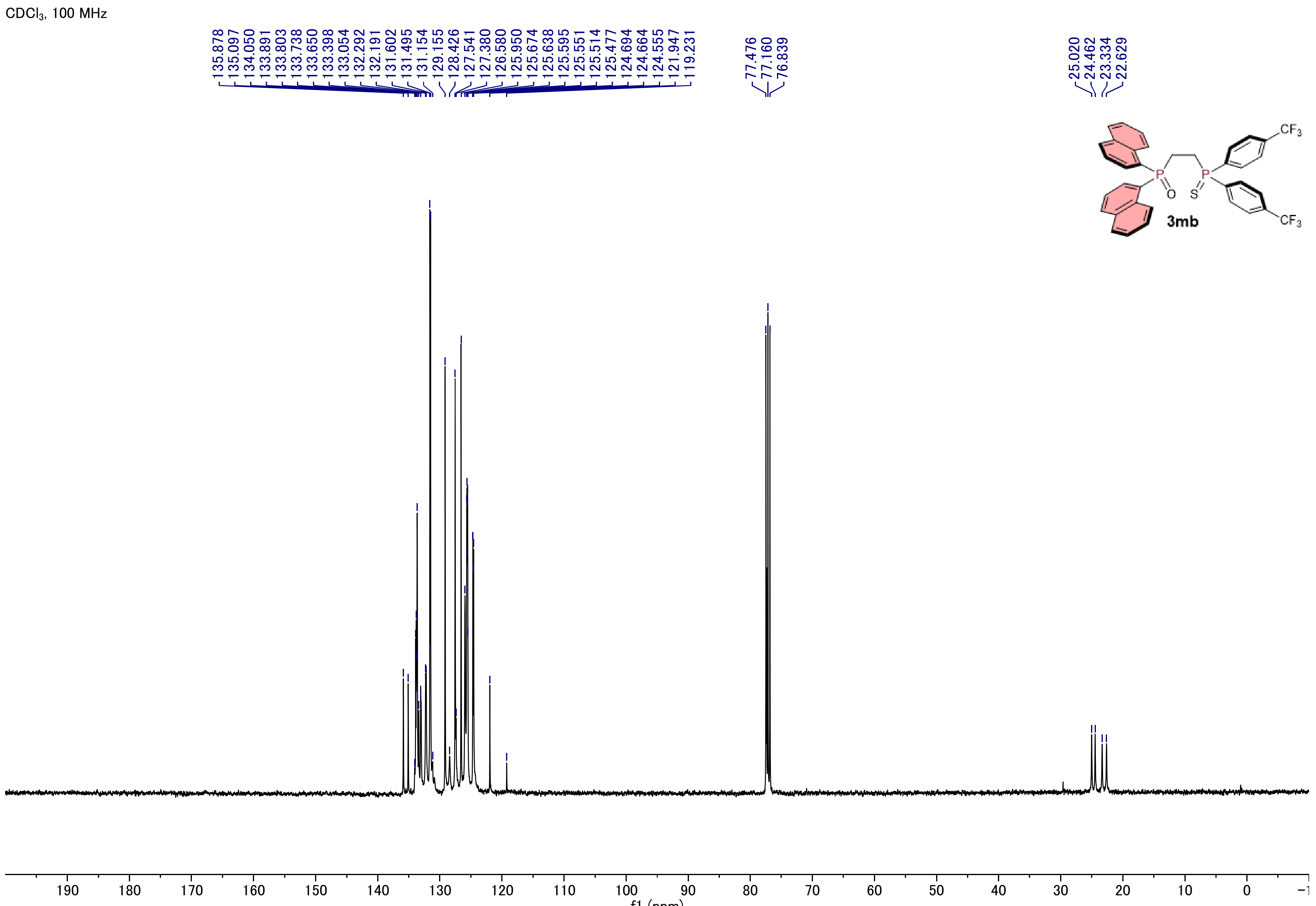

Supplementary Figure 74. ^{13}C NMR spectrum (100 MHz, $CDCl_3$) of **3mb**

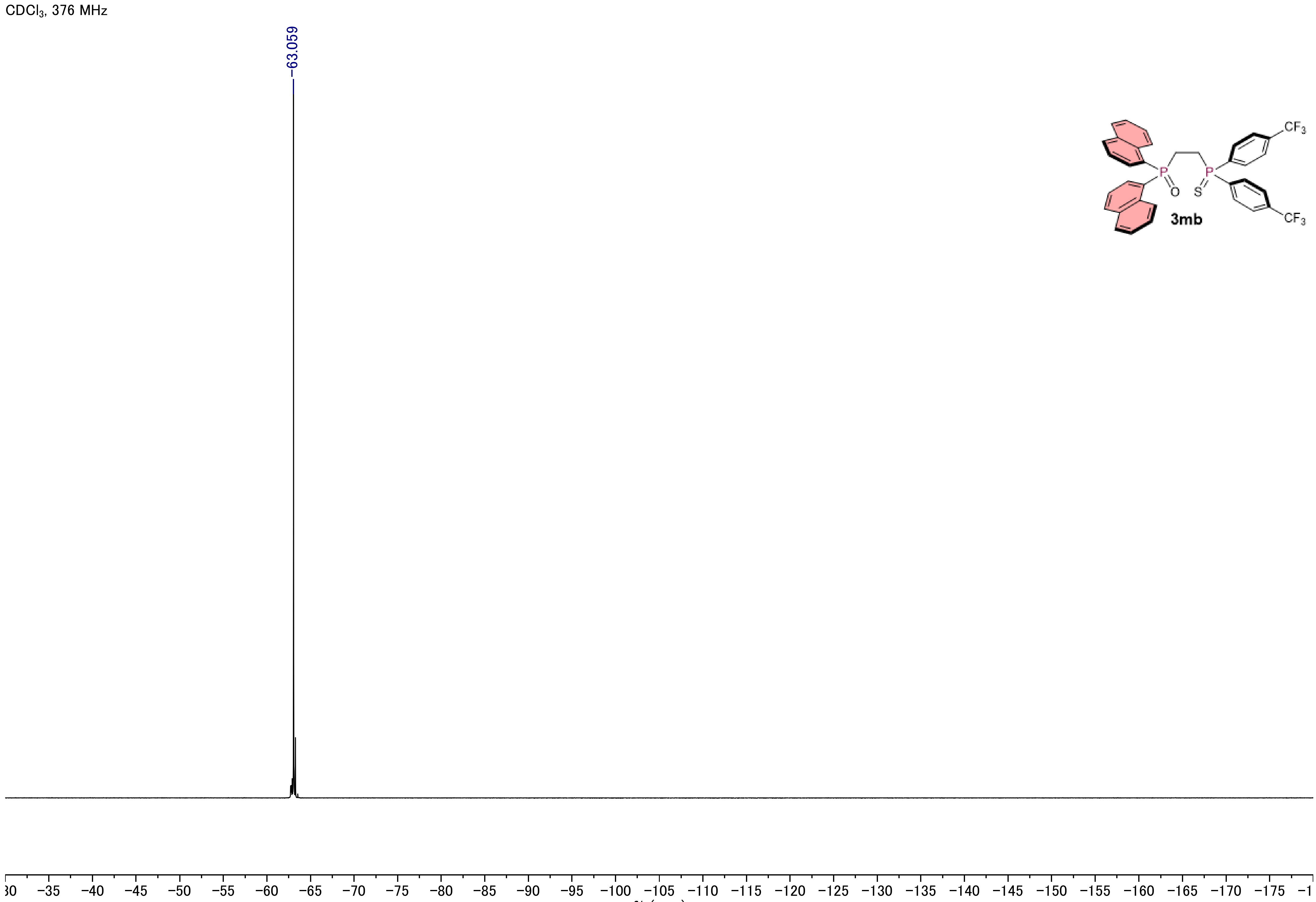

Supplementary Figure 75. ^{19}F NMR spectrum (376 MHz, $CDCl_3$) of **3mb**

CDCl$_3$, 162 MHz

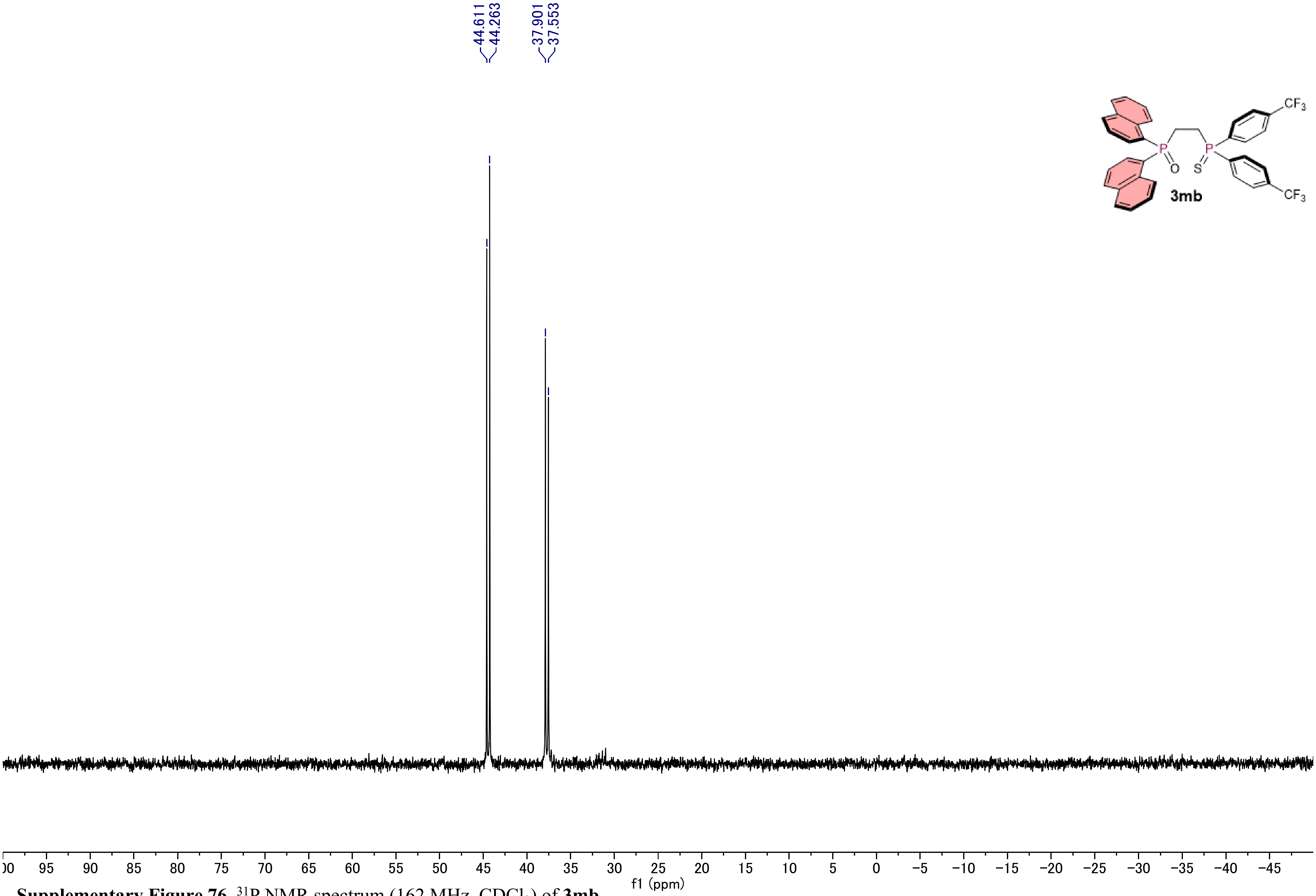

3mb

Supplementary Figure 76. ^{31}P NMR spectrum (162 MHz, CDCl$_3$) of **3mb**

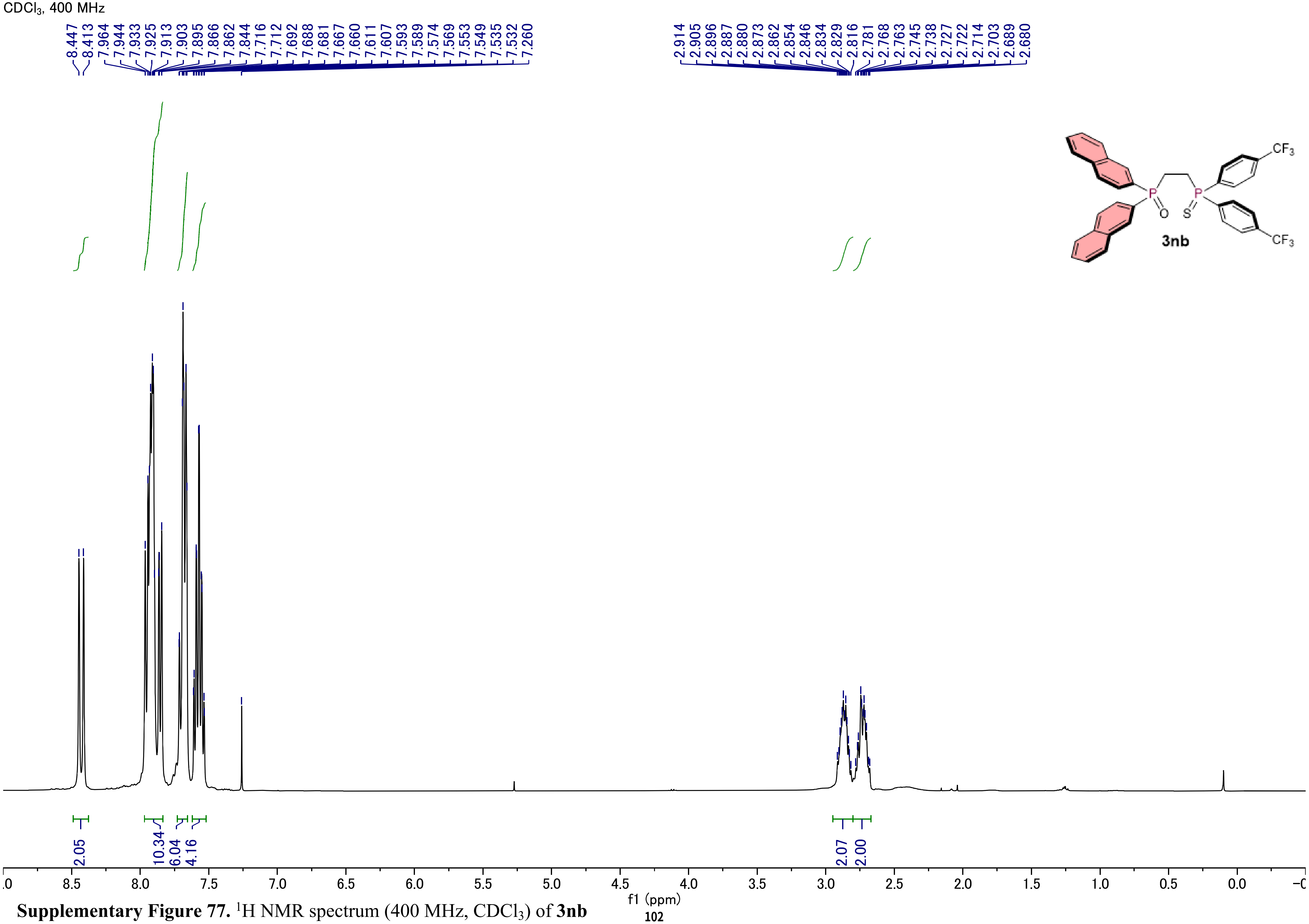

Supplementary Figure 77. ^{1}H NMR spectrum (400 MHz, $CDCl_3$) of **3nb**

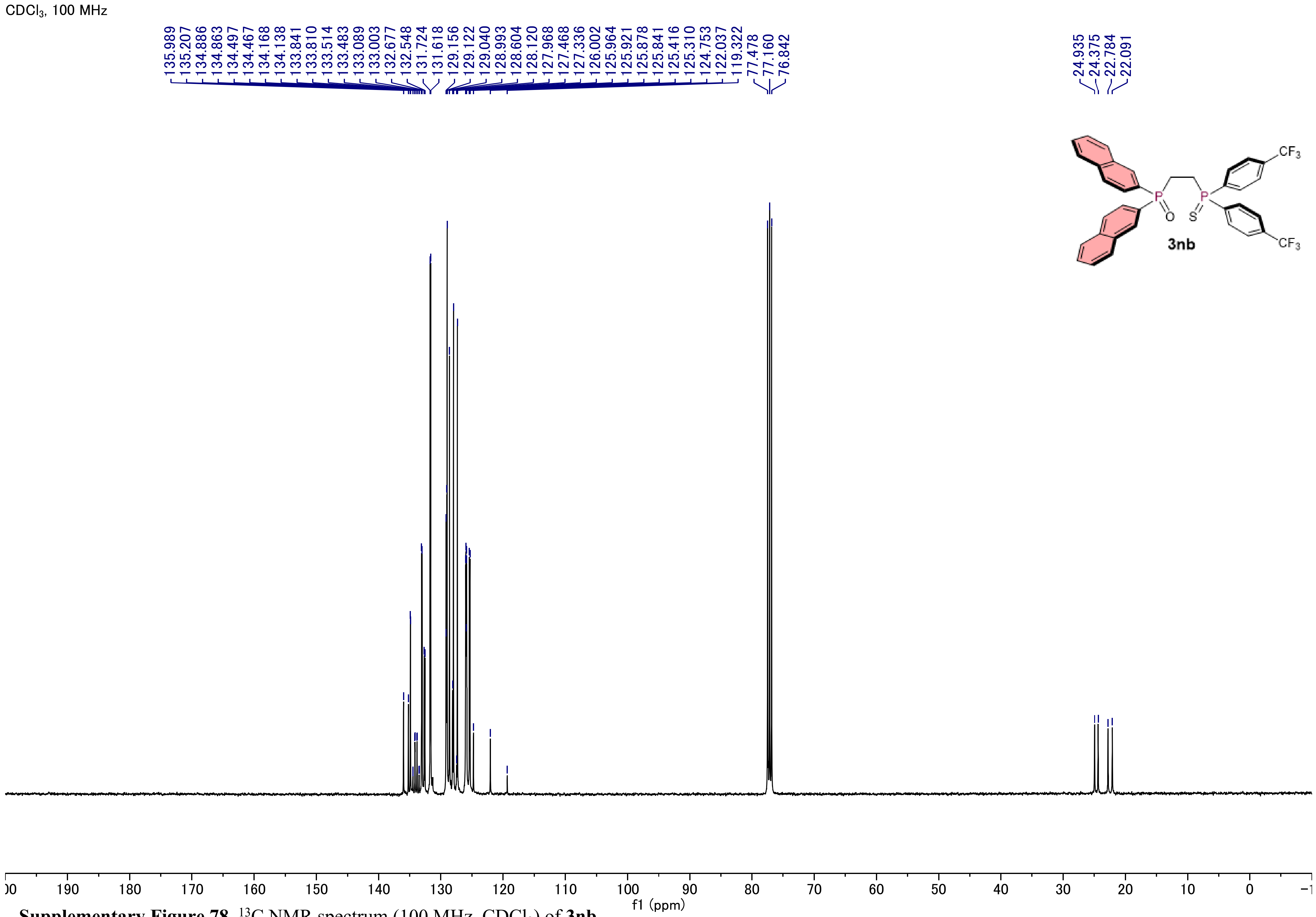

Supplementary Figure 78. ^{13}C NMR spectrum (100 MHz, $CDCl_3$) of **3nb**

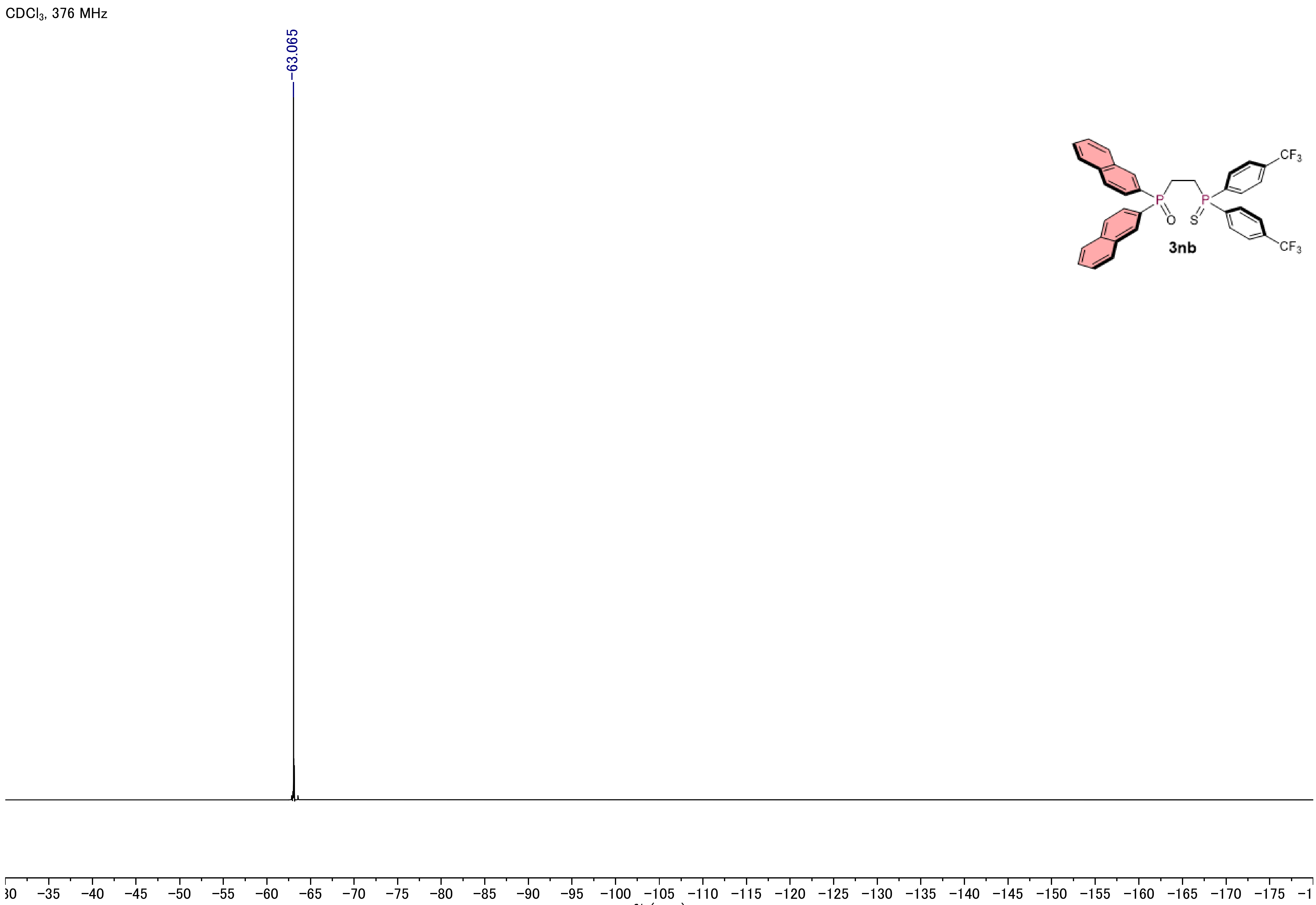

Supplementary Figure 79. ^{19}F NMR spectrum (376 MHz, $CDCl_3$) of **3nb**

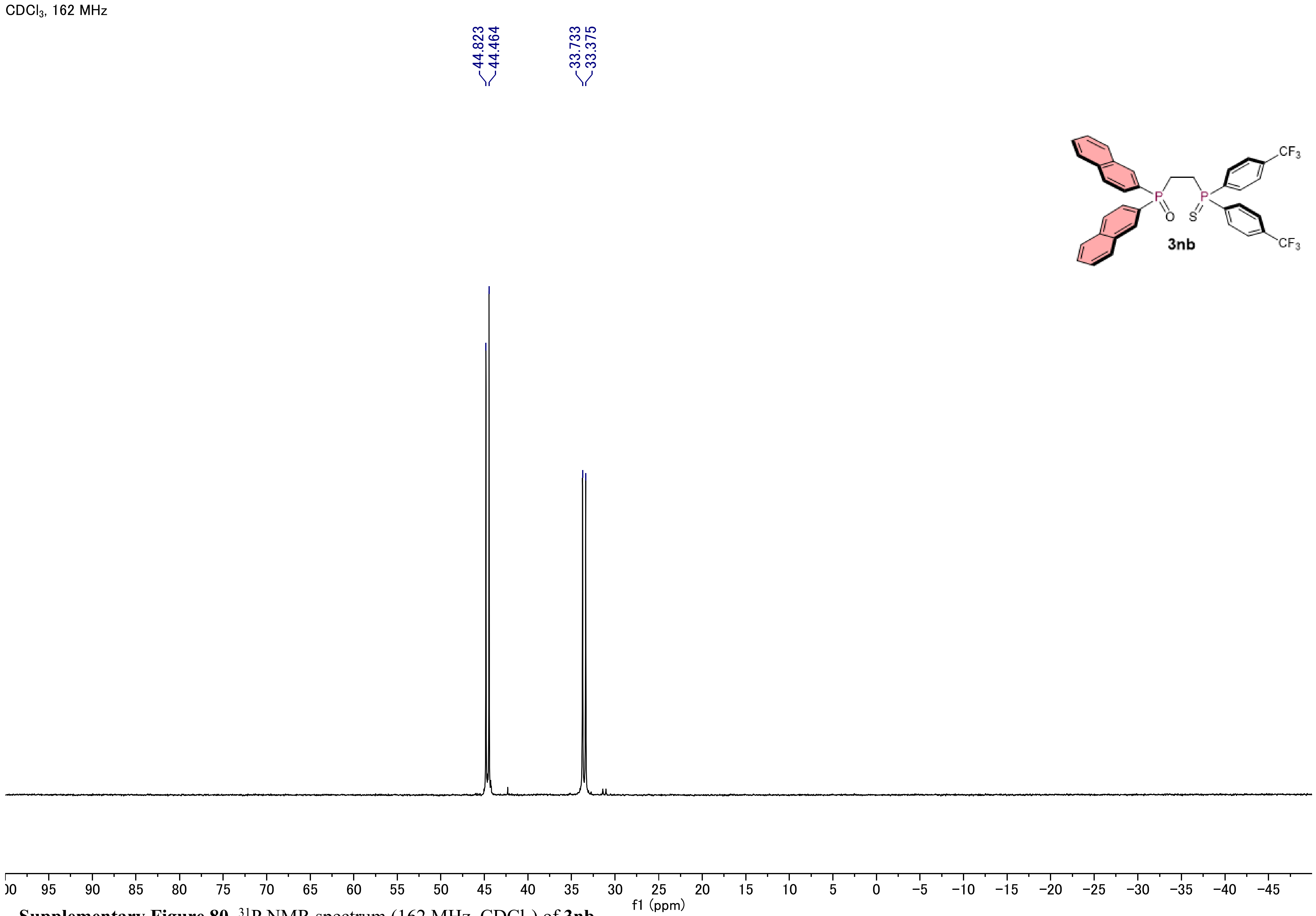

Supplementary Figure 80. ^{31}P NMR spectrum (162 MHz, $CDCl_3$) of **3nb**

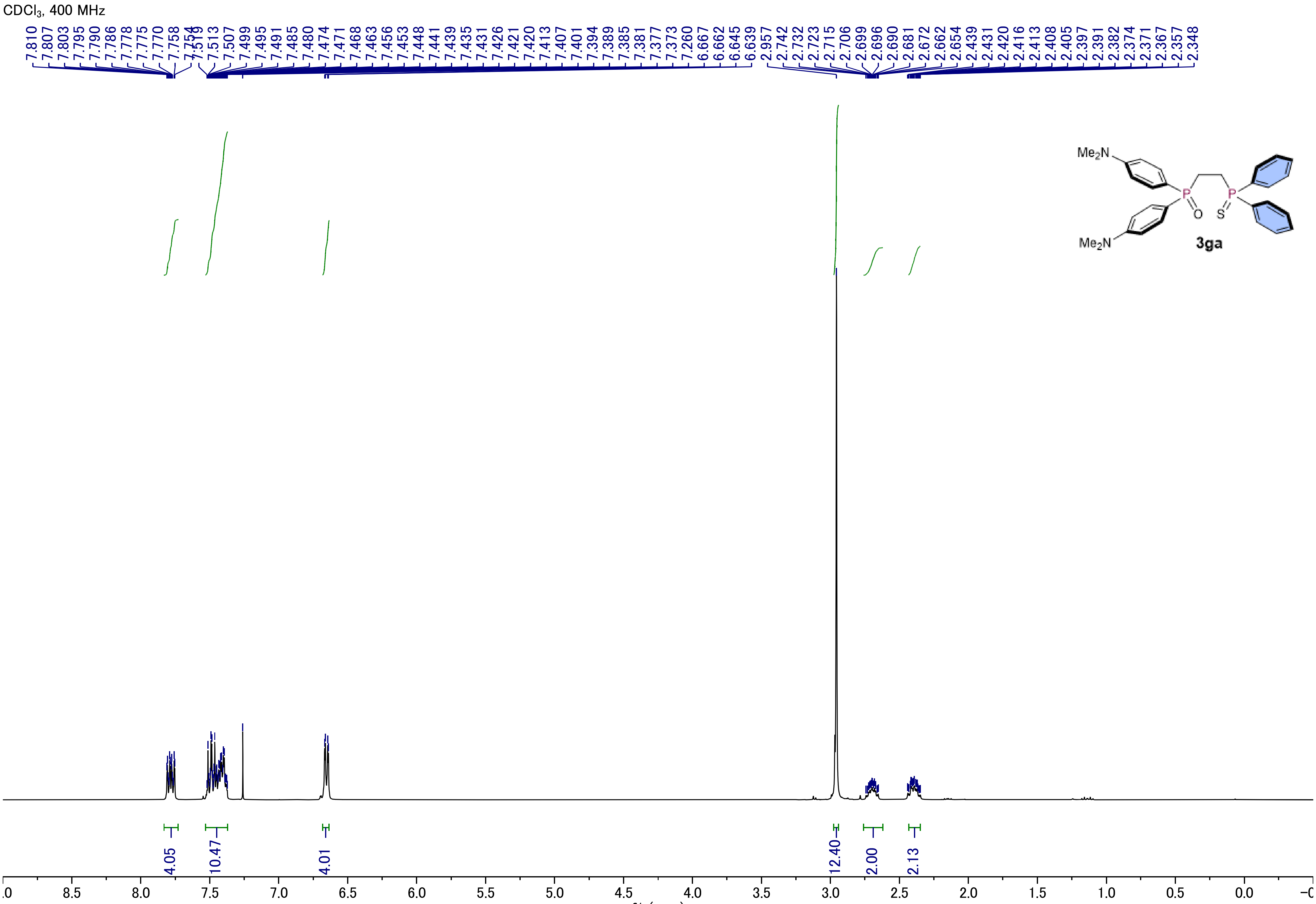

Supplementary Figure 81. ^{1}H NMR spectrum (400 MHz, $CDCl_3$) of **3ga**

CDCl$_3$, 100 MHz

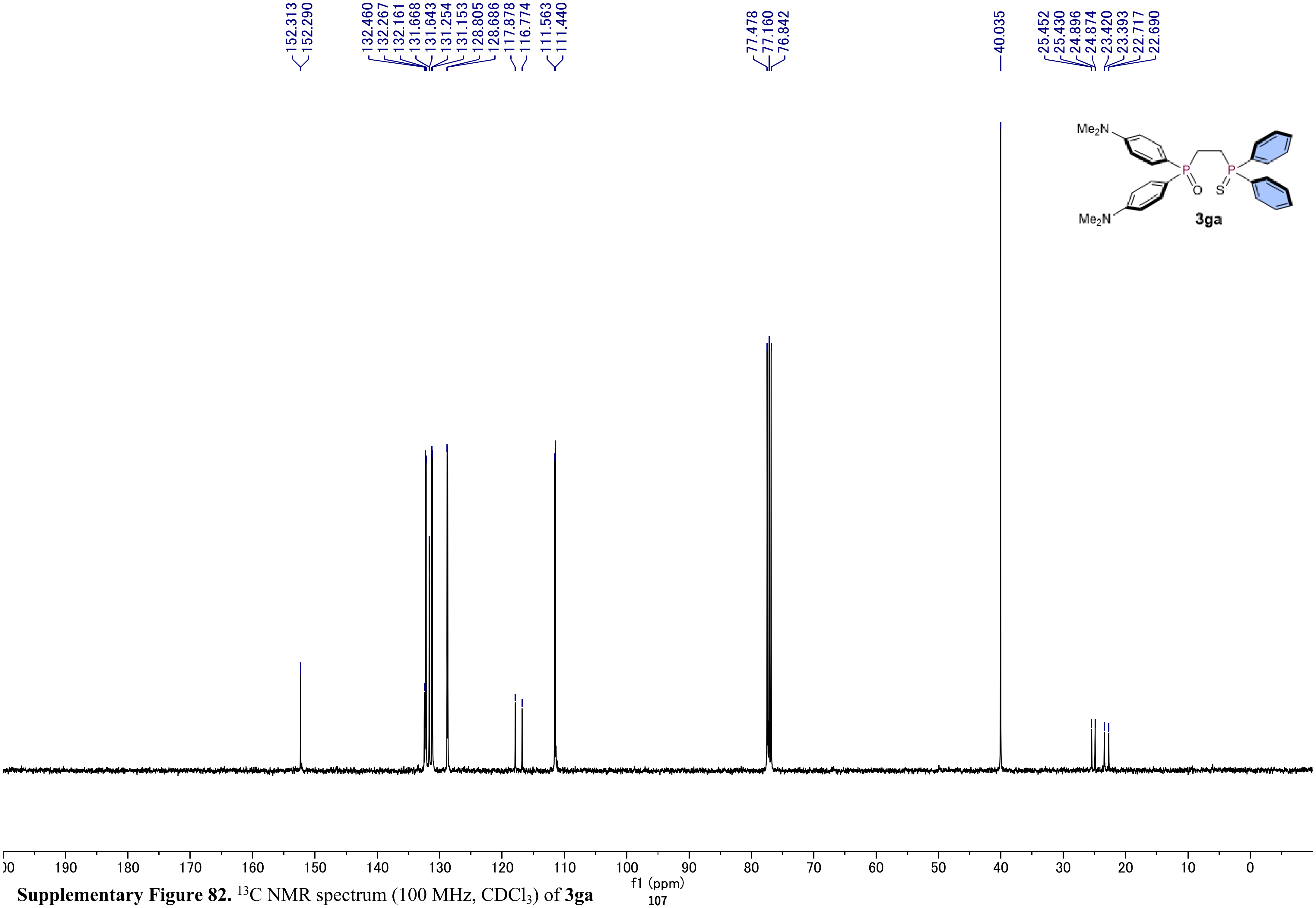

Supplementary Figure 82. ^{13}C NMR spectrum (100 MHz, CDCl$_3$) of **3ga**

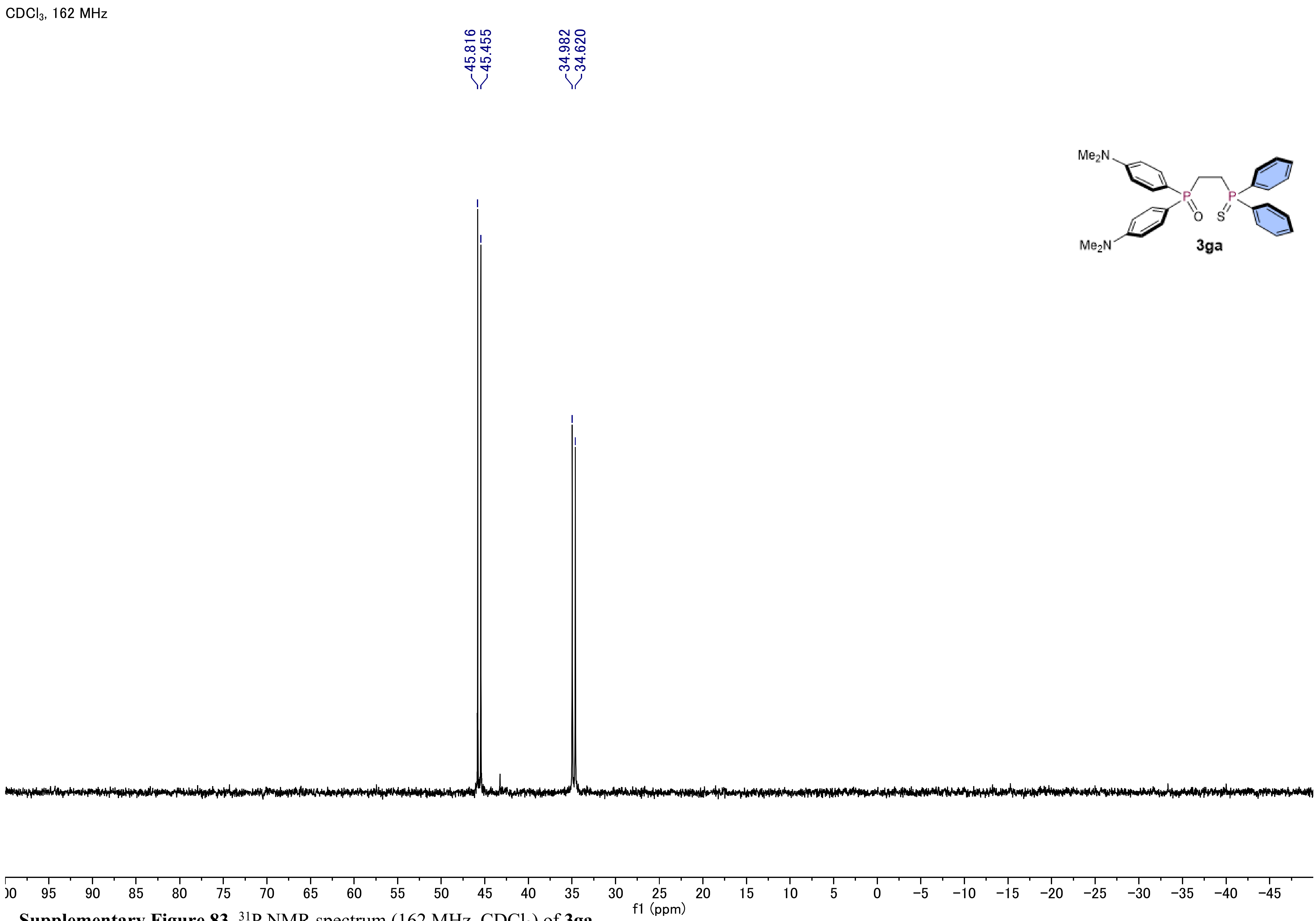

Supplementary Figure 83. ^{31}P NMR spectrum (162 MHz, $CDCl_3$) of **3ga**

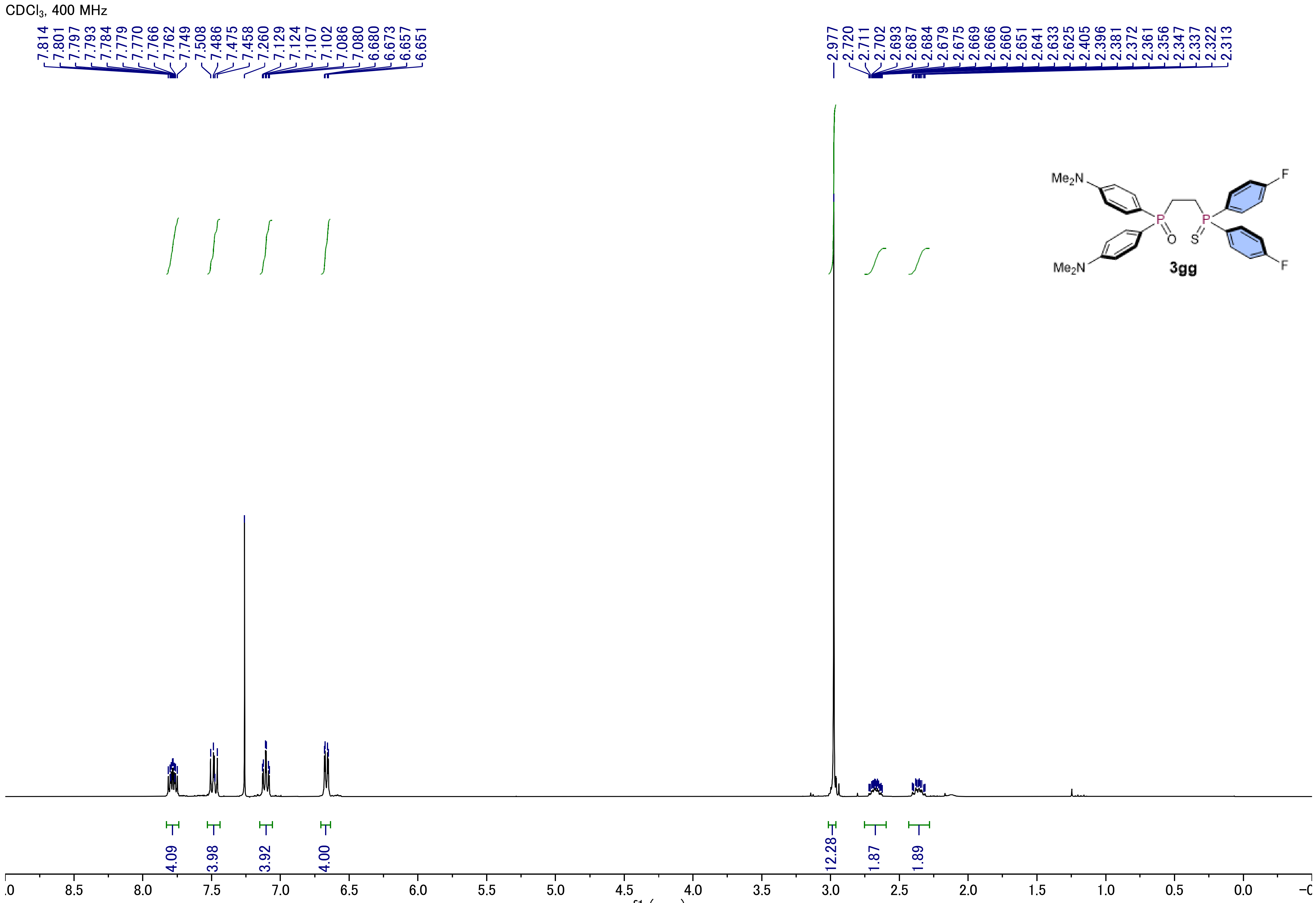

Supplementary Figure 84. ^{1}H NMR spectrum (400 MHz, $CDCl_3$) of **3gg**

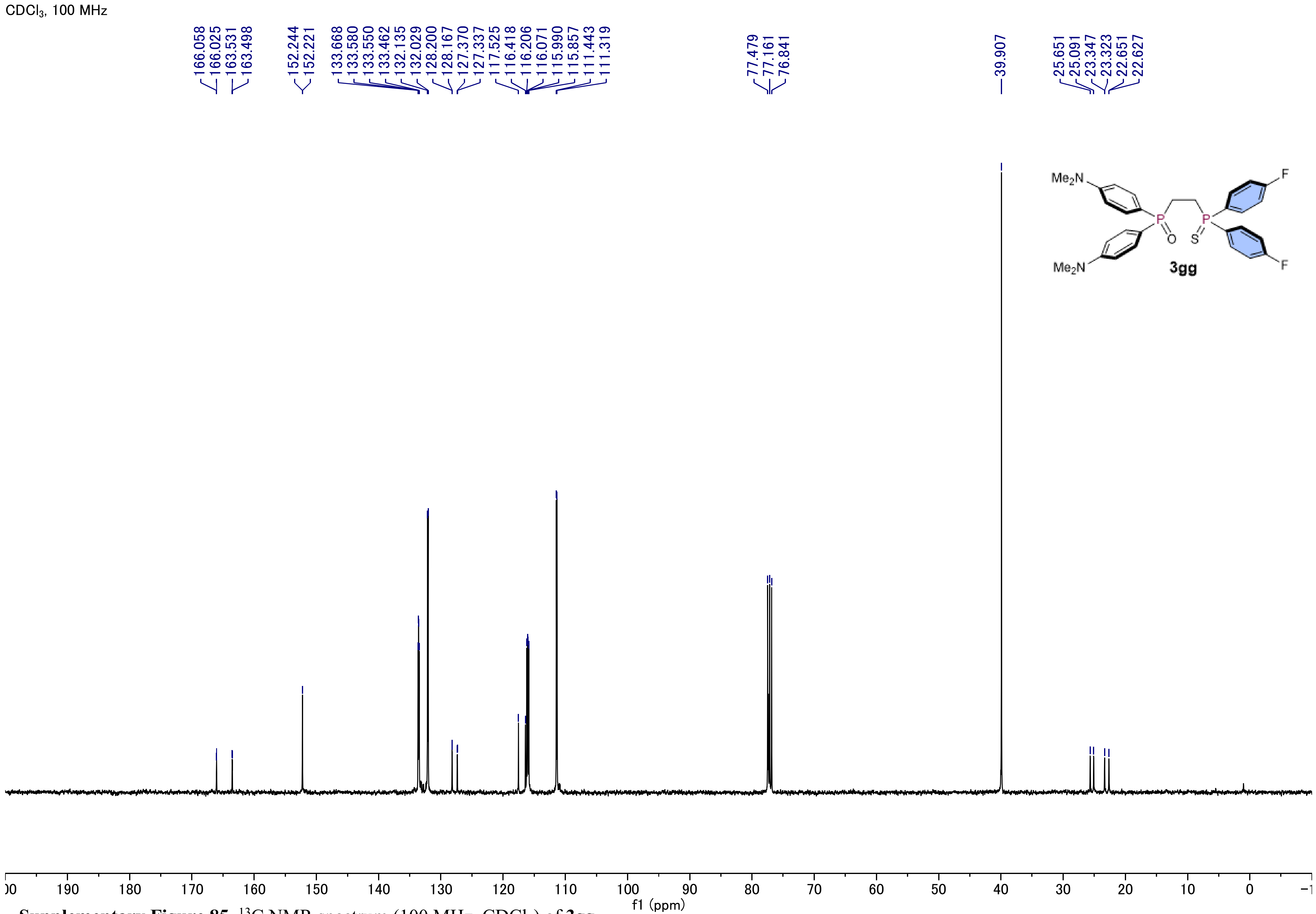

Supplementary Figure 85. ^{13}C NMR spectrum (100 MHz, $CDCl_3$) of **3gg**

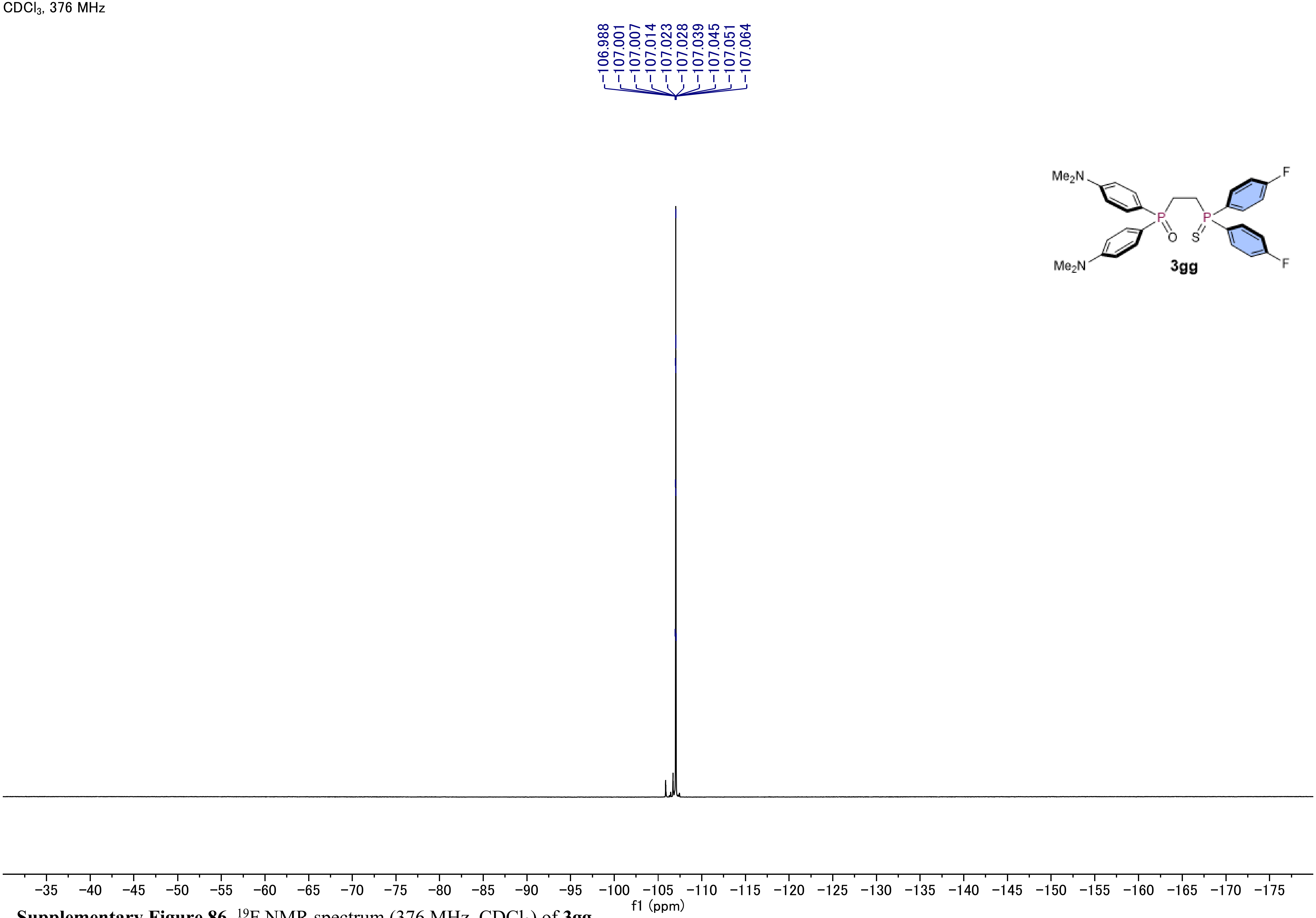

Supplementary Figure 86. ^{19}F NMR spectrum (376 MHz, $CDCl_3$) of **3gg**

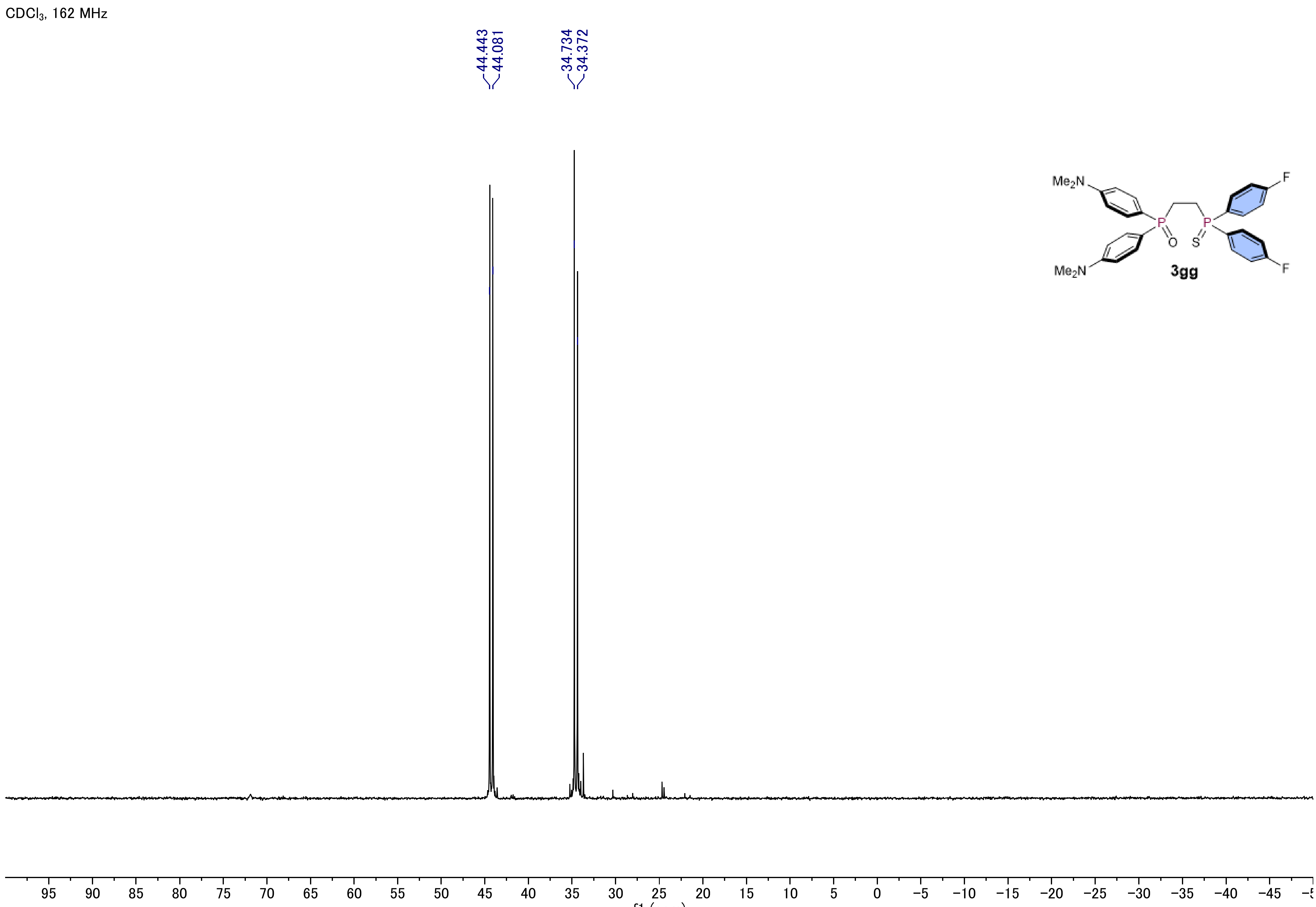

Supplementary Figure 87. ^{31}P NMR spectrum (162 MHz, $CDCl_3$) of **3gg**

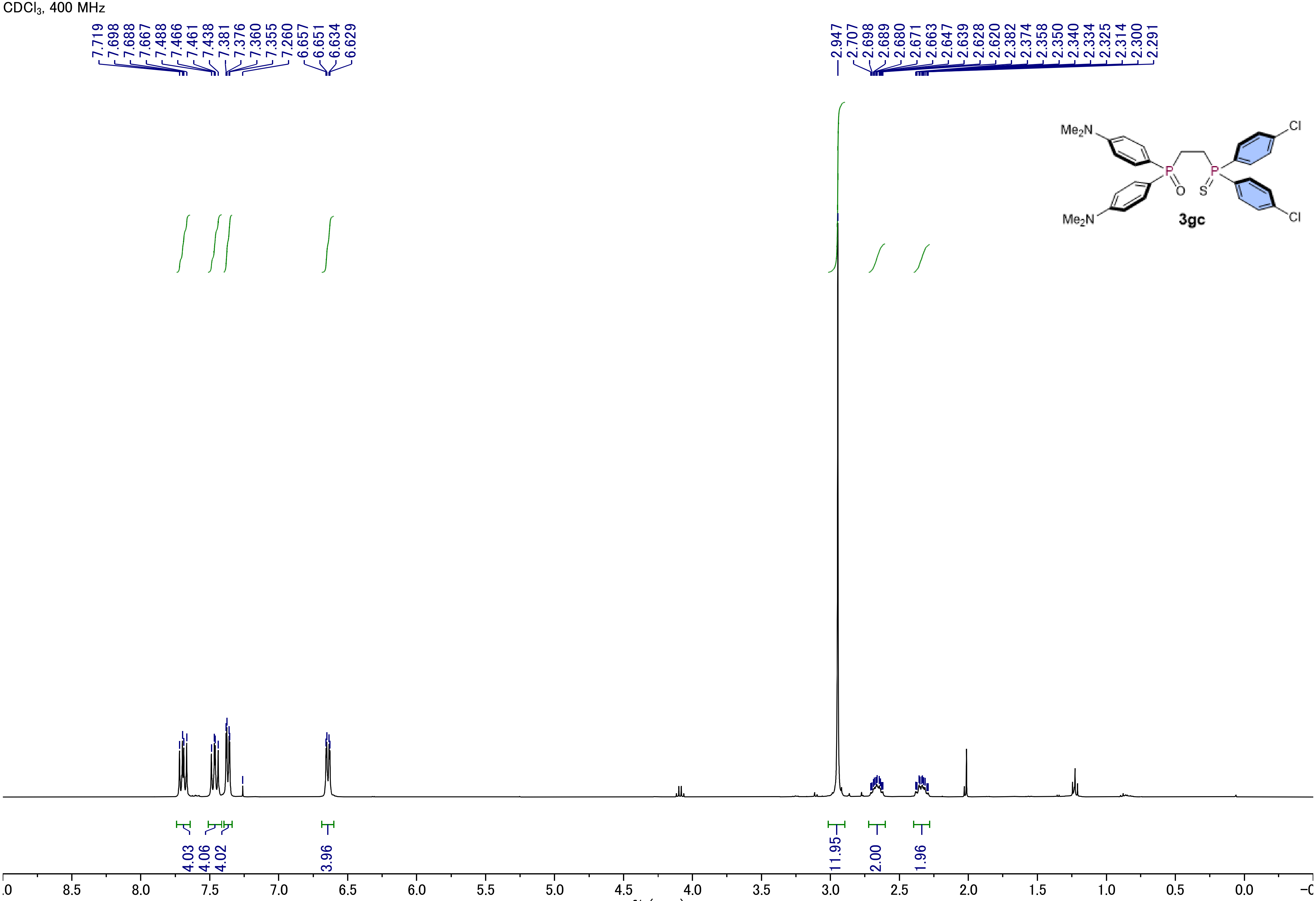

Supplementary Figure 88. ^{1}H NMR spectrum (400 MHz, $CDCl_3$) of **3gc**

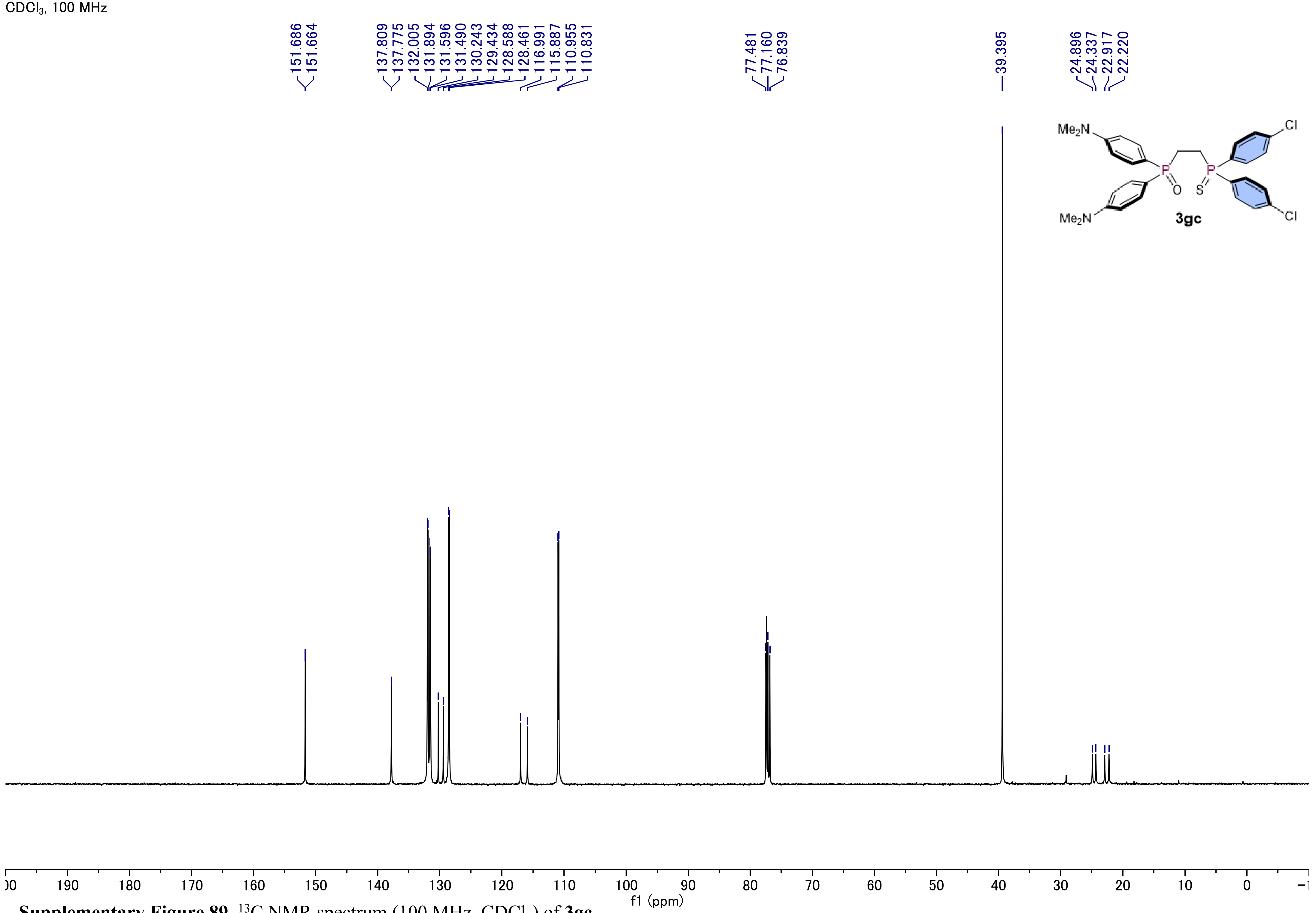

Supplementary Figure 89. ^{13}C NMR spectrum (100 MHz, $CDCl_3$) of **3gc**

CDCl$_3$, 162 MHz

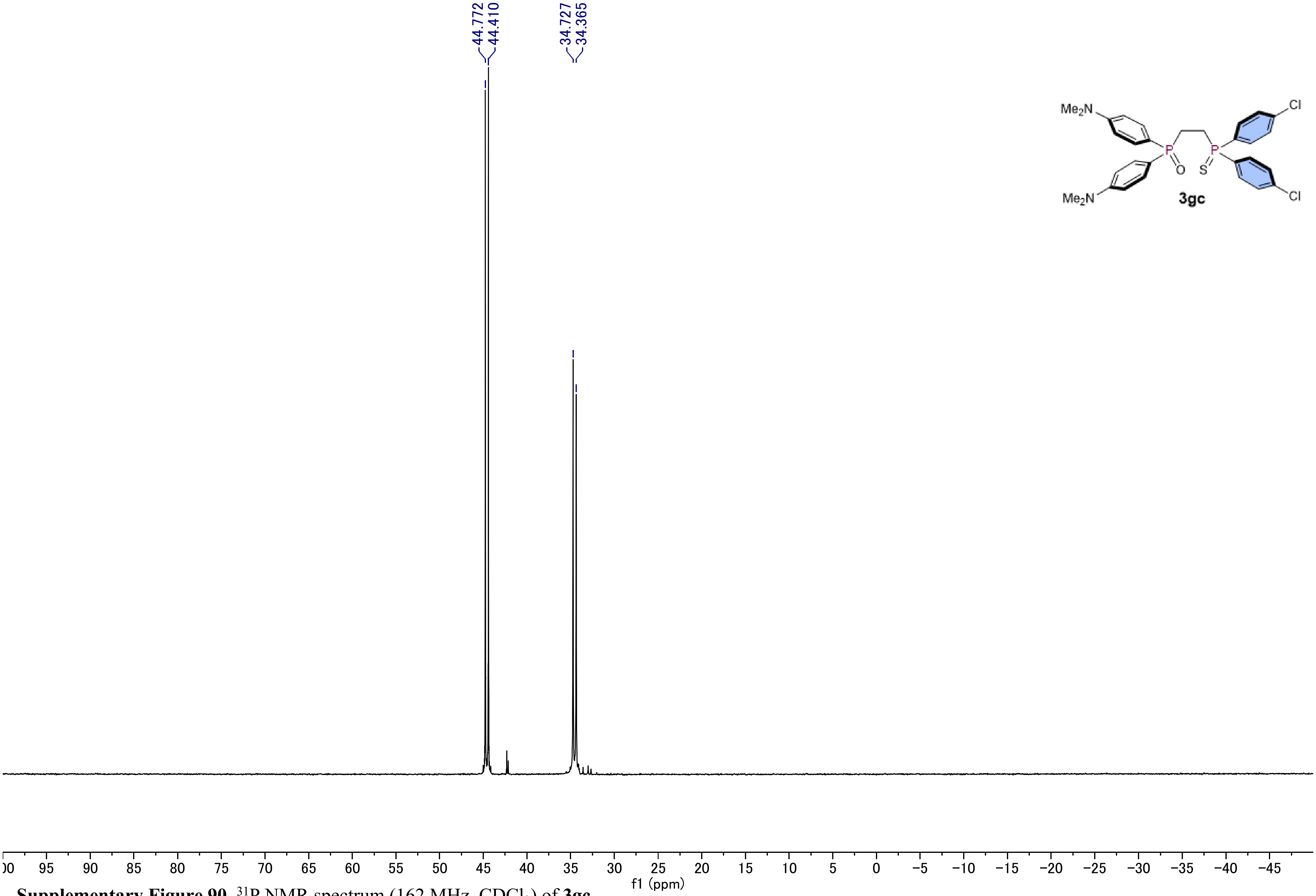

Supplementary Figure 90. ^{31}P NMR spectrum (162 MHz, CDCl$_3$) of **3gc**

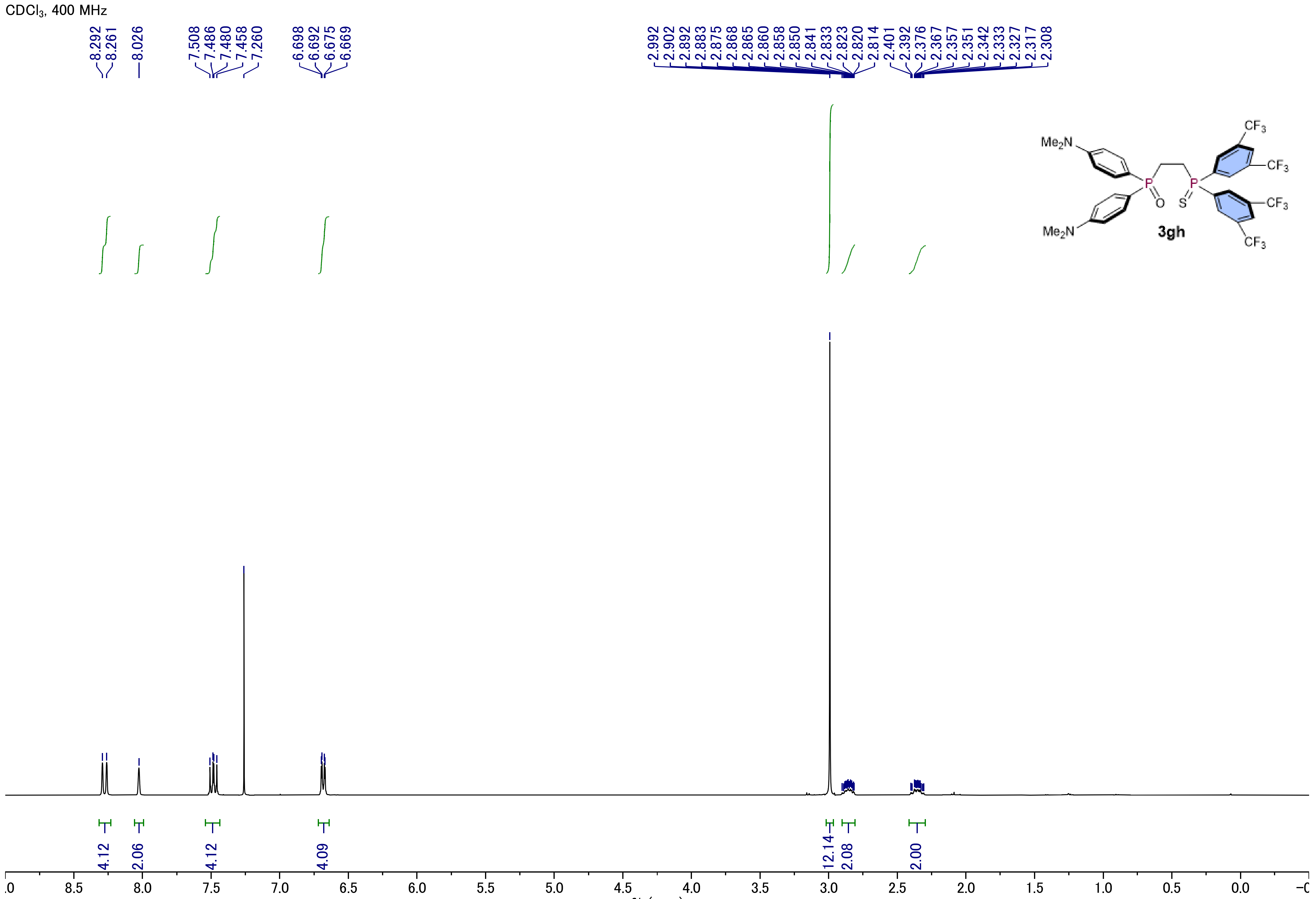

Supplementary Figure 91. ^{1}H NMR spectrum (400 MHz, $CDCl_3$) of **3gh**

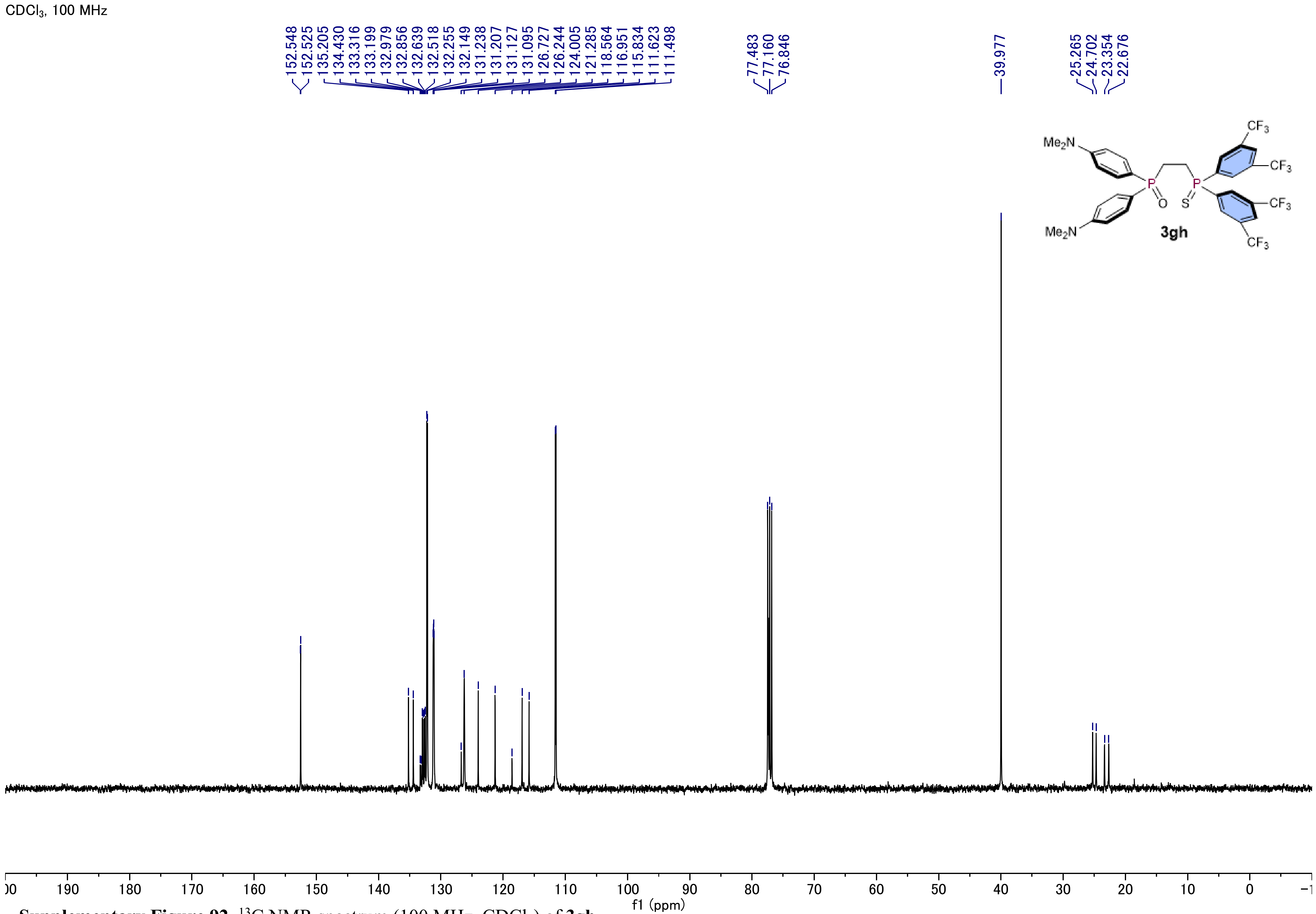

Supplementary Figure 92. ^{13}C NMR spectrum (100 MHz, $CDCl_3$) of **3gh**

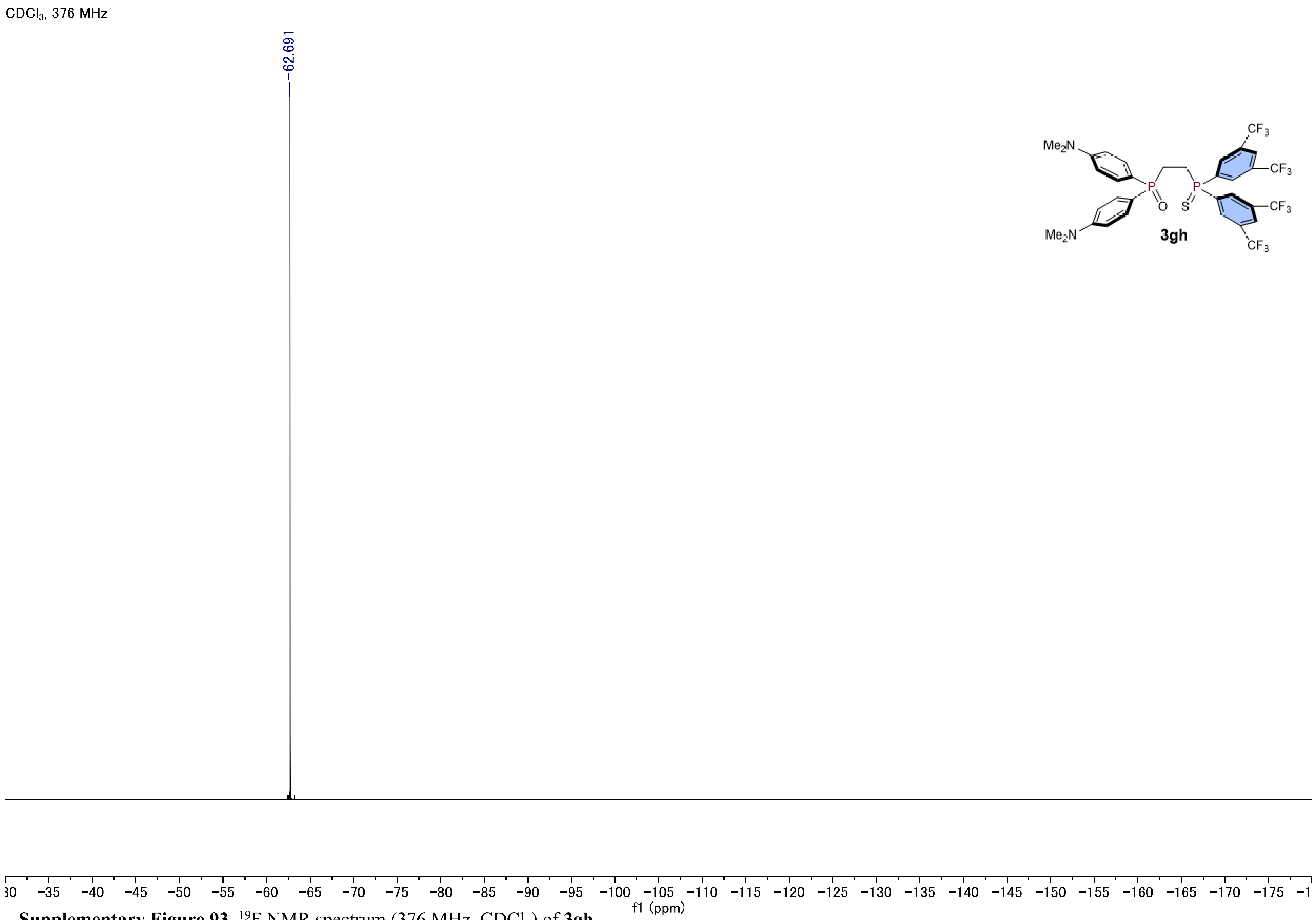

Supplementary Figure 93. ^{19}F NMR spectrum (376 MHz, $CDCl_3$) of **3gh**

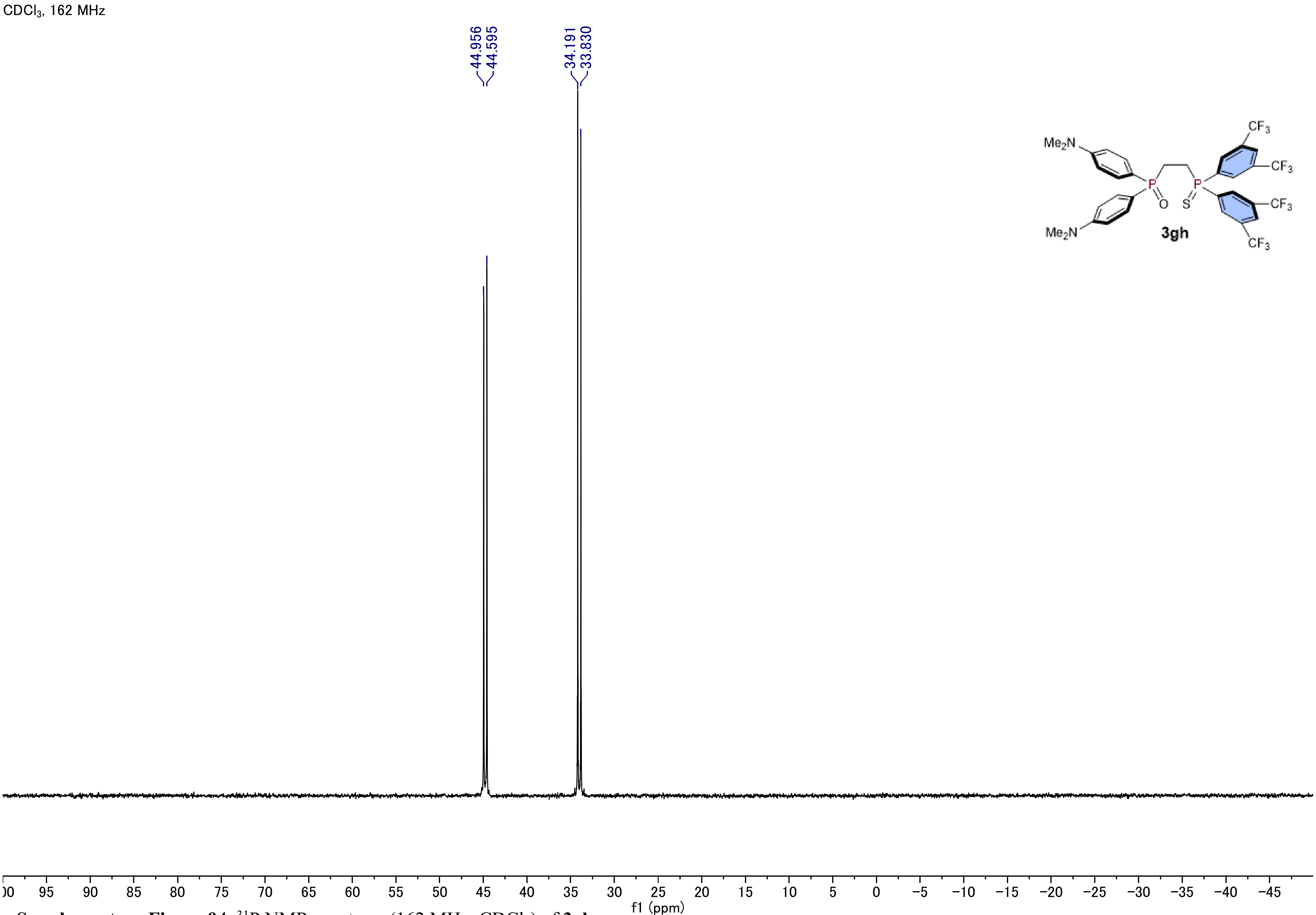

Supplementary Figure 94. ^{31}P NMR spectrum (162 MHz, $CDCl_3$) of **3gh**

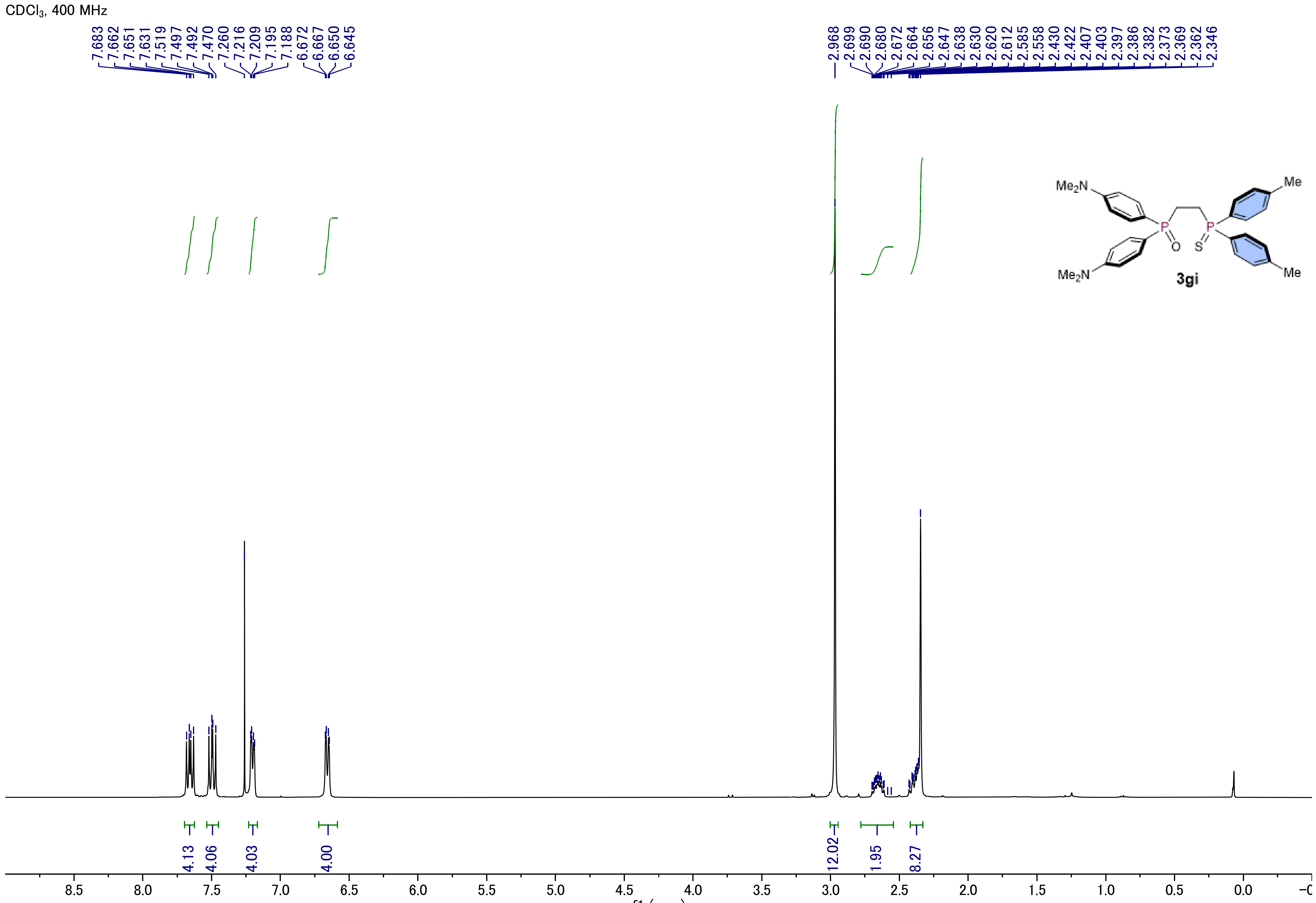

Supplementary Figure 95. ^{1}H NMR spectrum (400 MHz, $CDCl_3$) of **3gi**

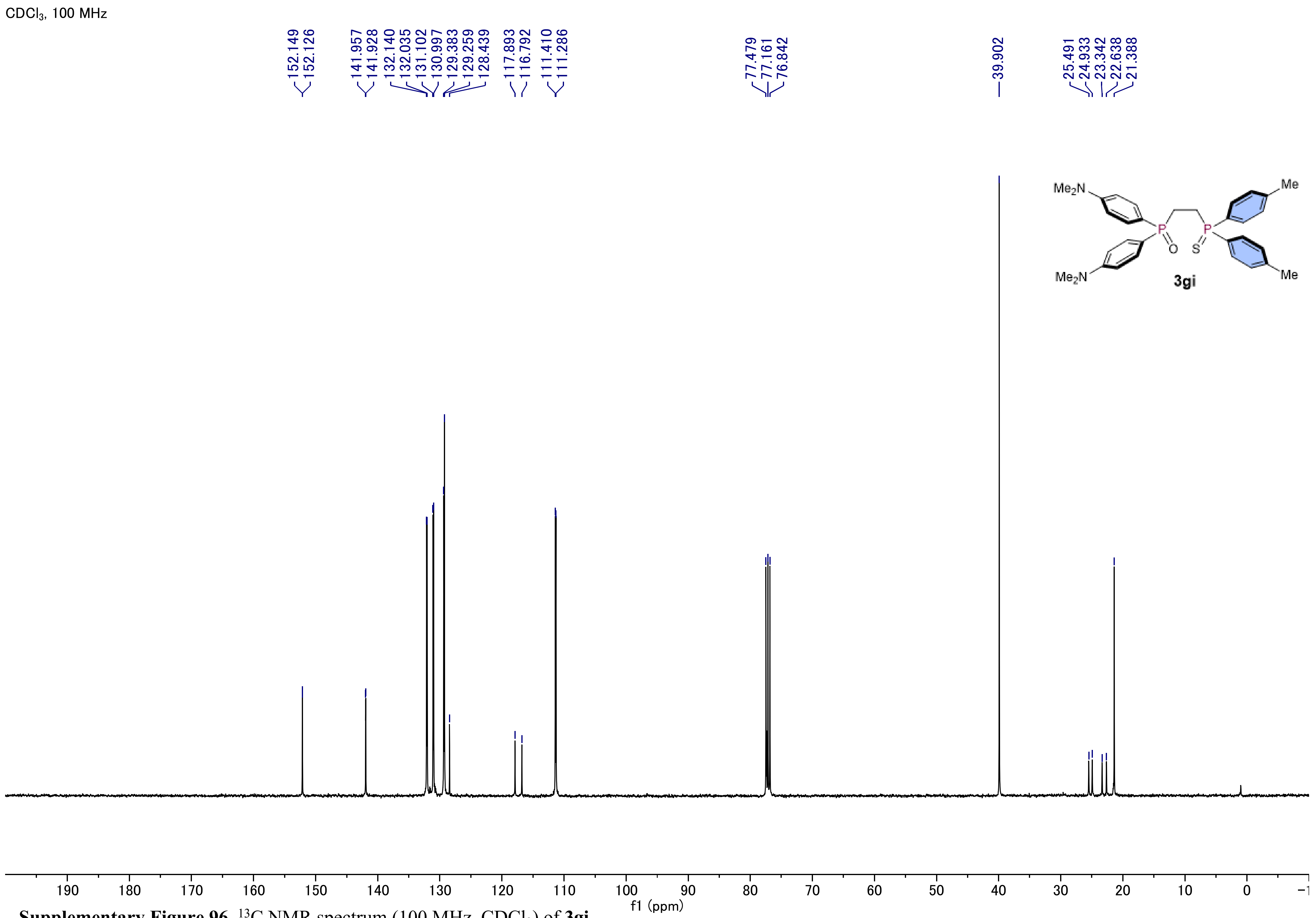

Supplementary Figure 96. ^{13}C NMR spectrum (100 MHz, $CDCl_3$) of **3gi**

CDCl$_3$, 162 MHz

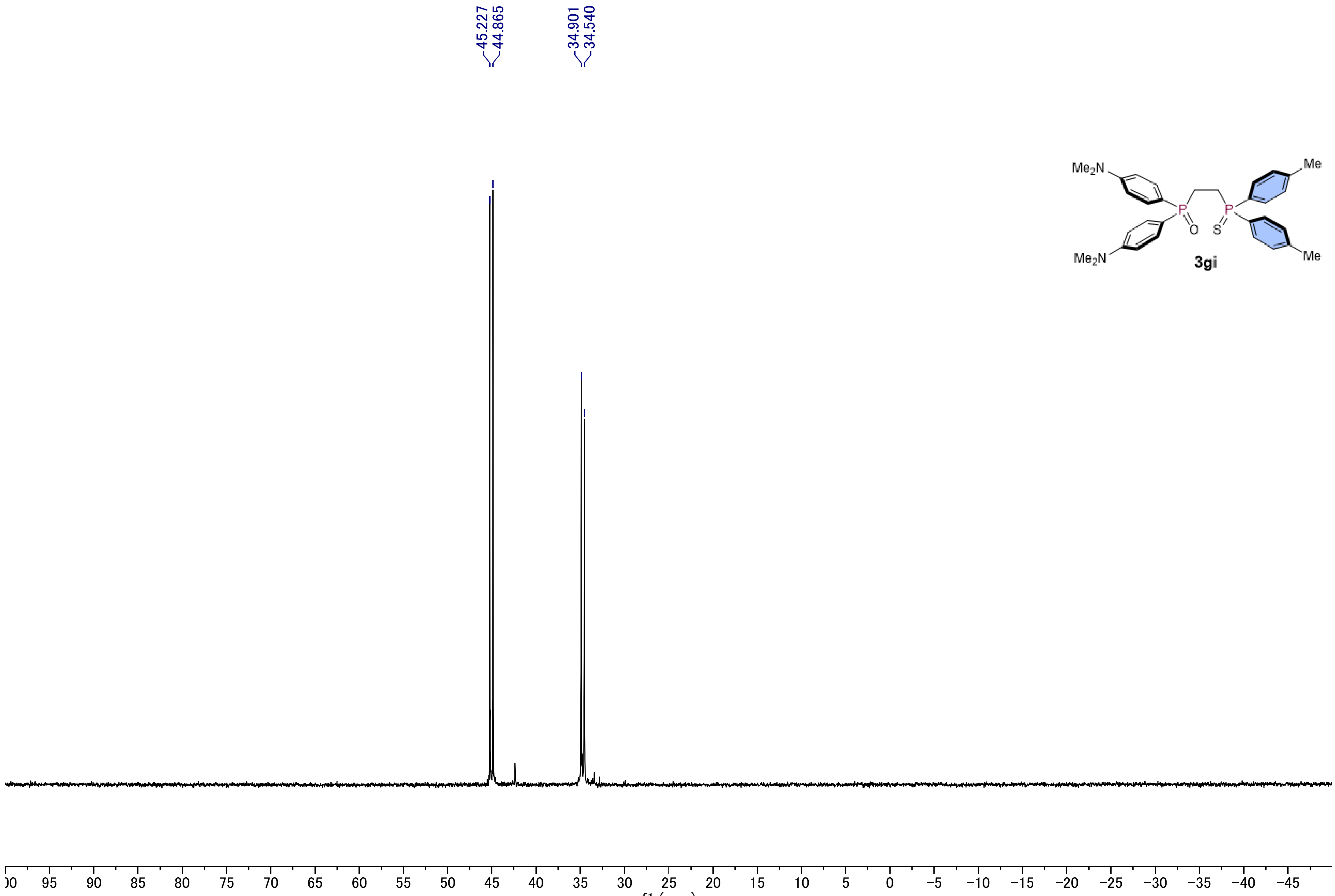

3gi

Supplementary Figure 97. ^{31}P NMR spectrum (162 MHz, CDCl$_3$) of **3gi**

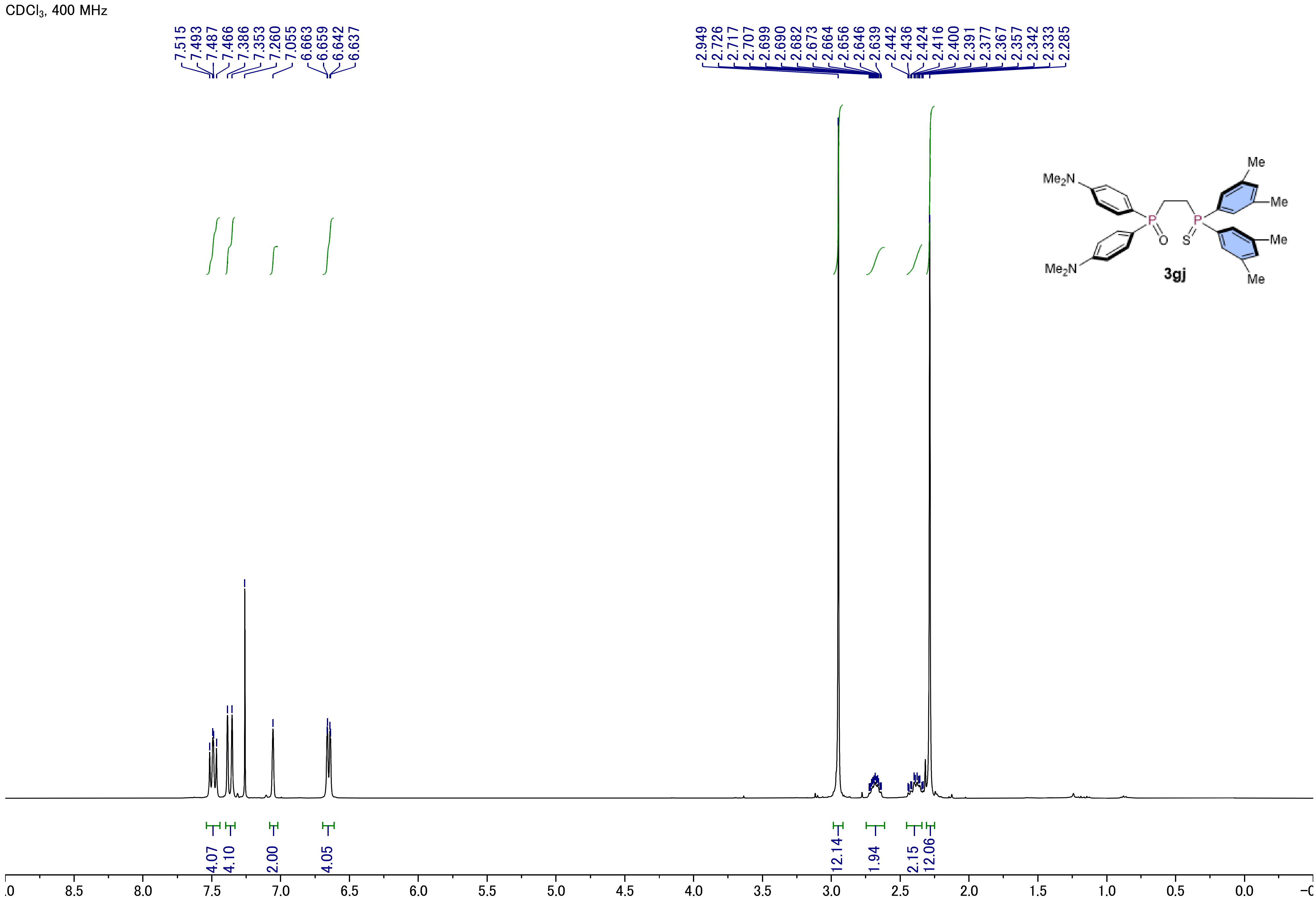

Supplementary Figure 98. ^{1}H NMR spectrum (400 MHz, $CDCl_3$) of **3gj**

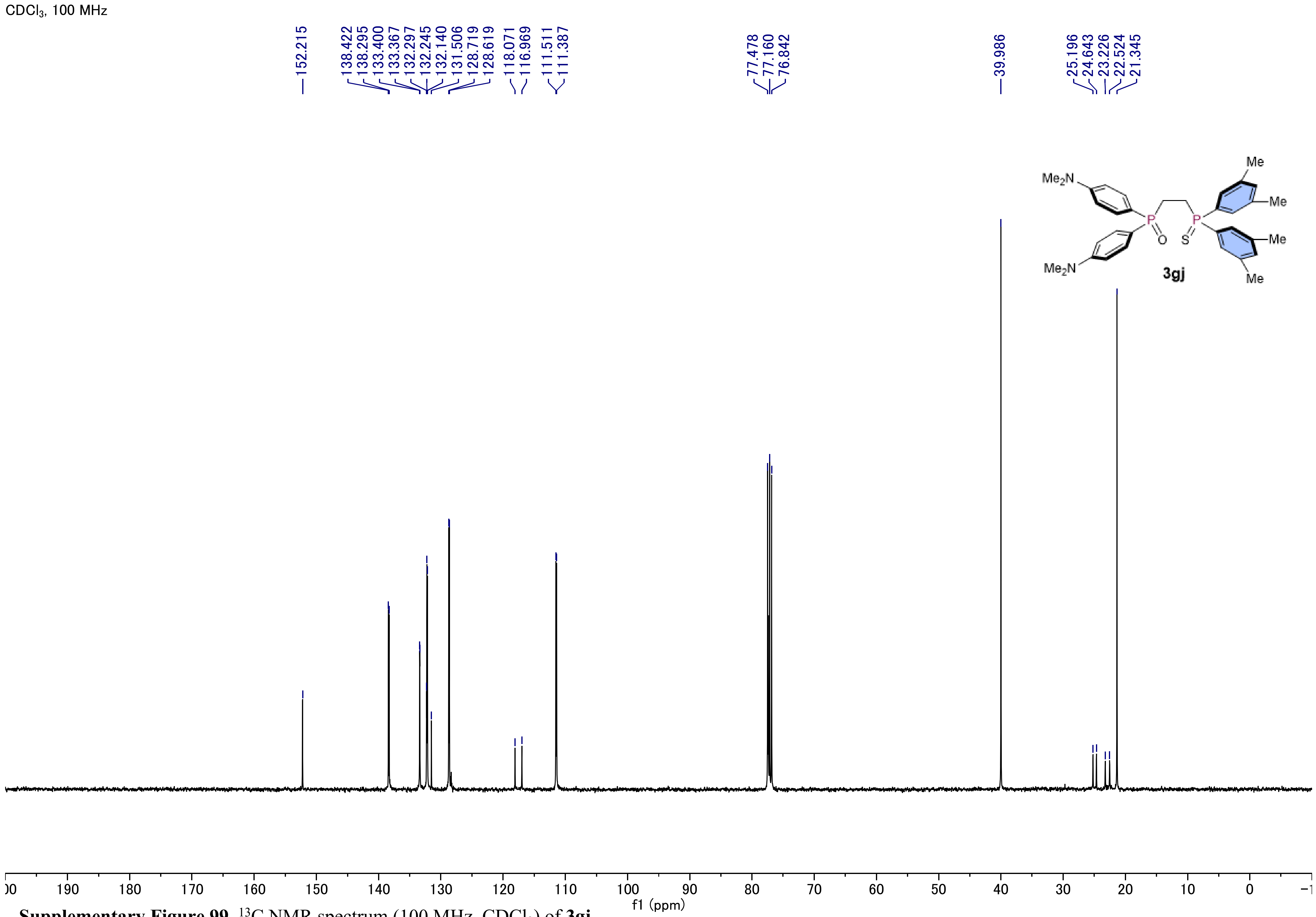

Supplementary Figure 99. ^{13}C NMR spectrum (100 MHz, $CDCl_3$) of **3gj**

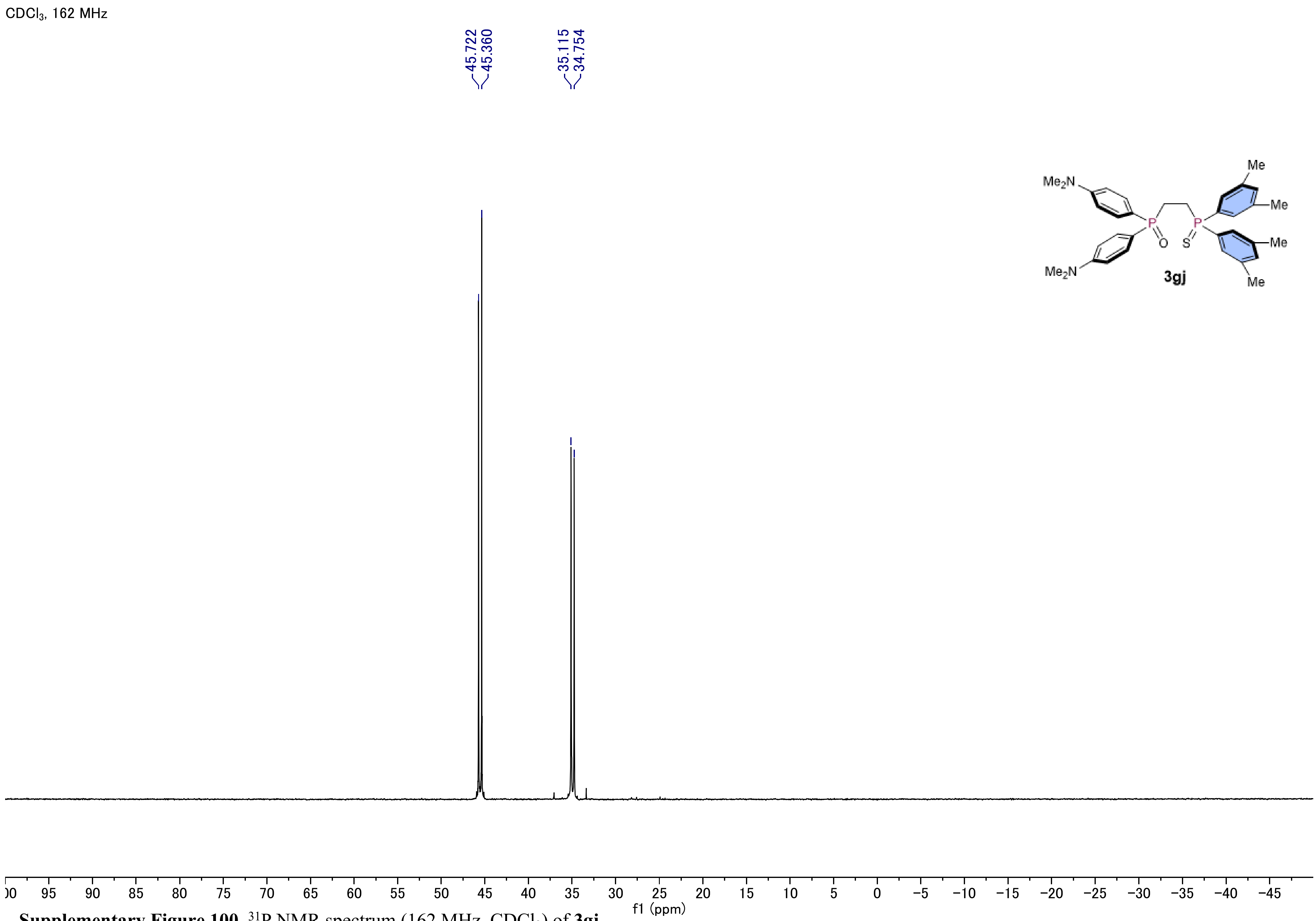

Supplementary Figure 100. ^{31}P NMR spectrum (162 MHz, $CDCl_3$) of **3gj**

CDCl$_3$, 400 MHz

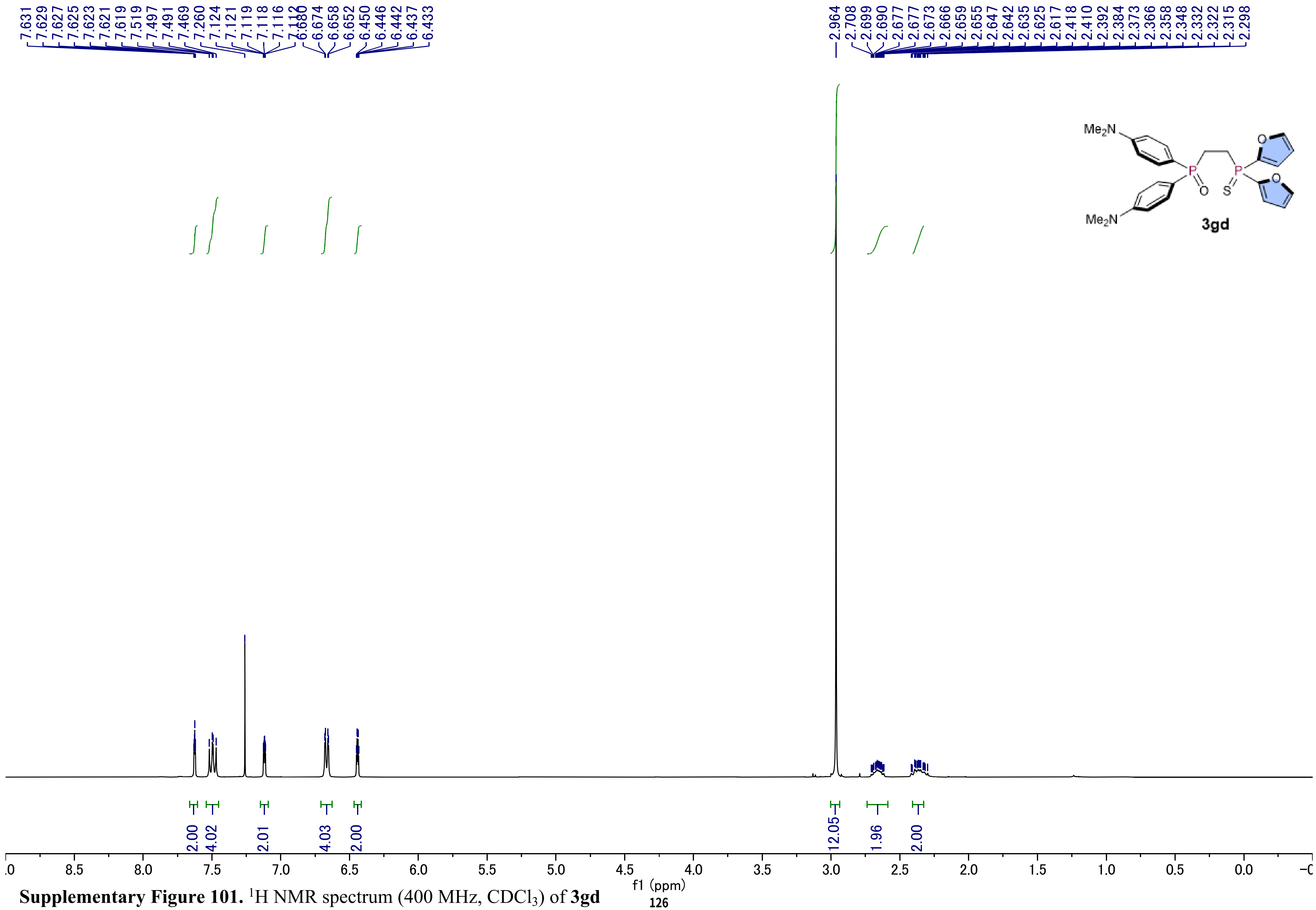

3gd

Supplementary Figure 101. ^{1}H NMR spectrum (400 MHz, CDCl$_3$) of **3gd**

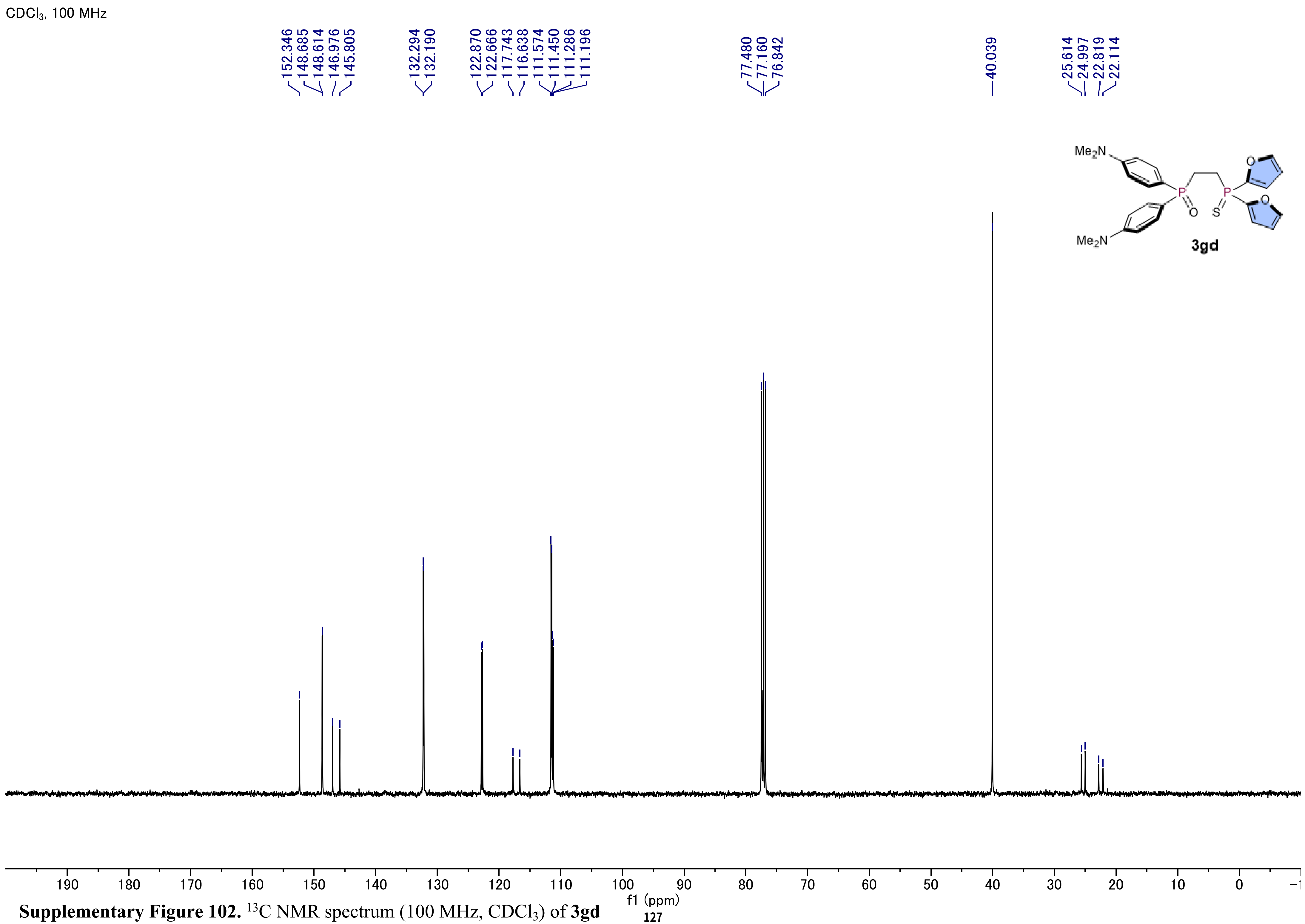

Supplementary Figure 102. ^{13}C NMR spectrum (100 MHz, $CDCl_3$) of **3gd**

CDCl$_3$, 162 MHz

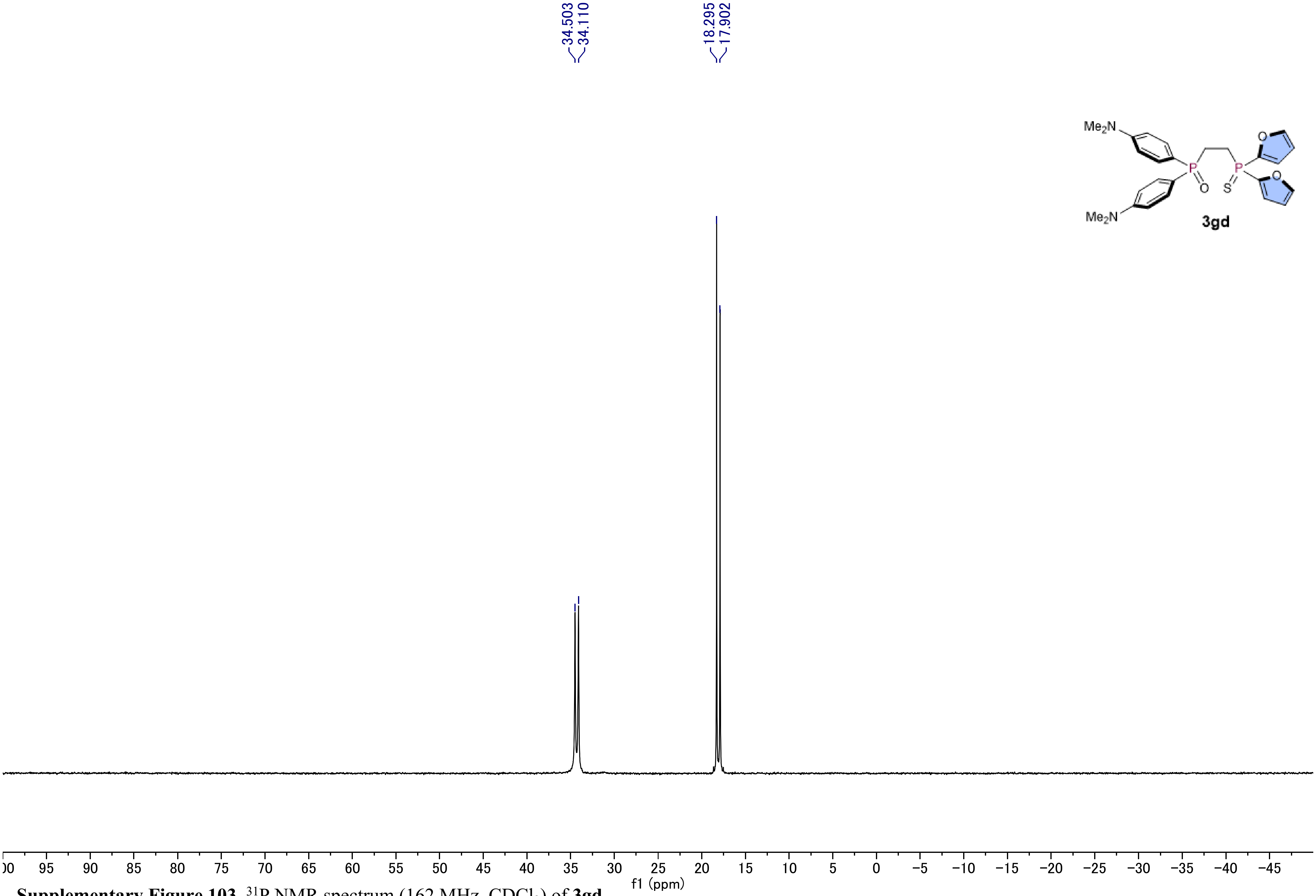

3gd

Supplementary Figure 103. ^{31}P NMR spectrum (162 MHz, CDCl$_3$) of **3gd**

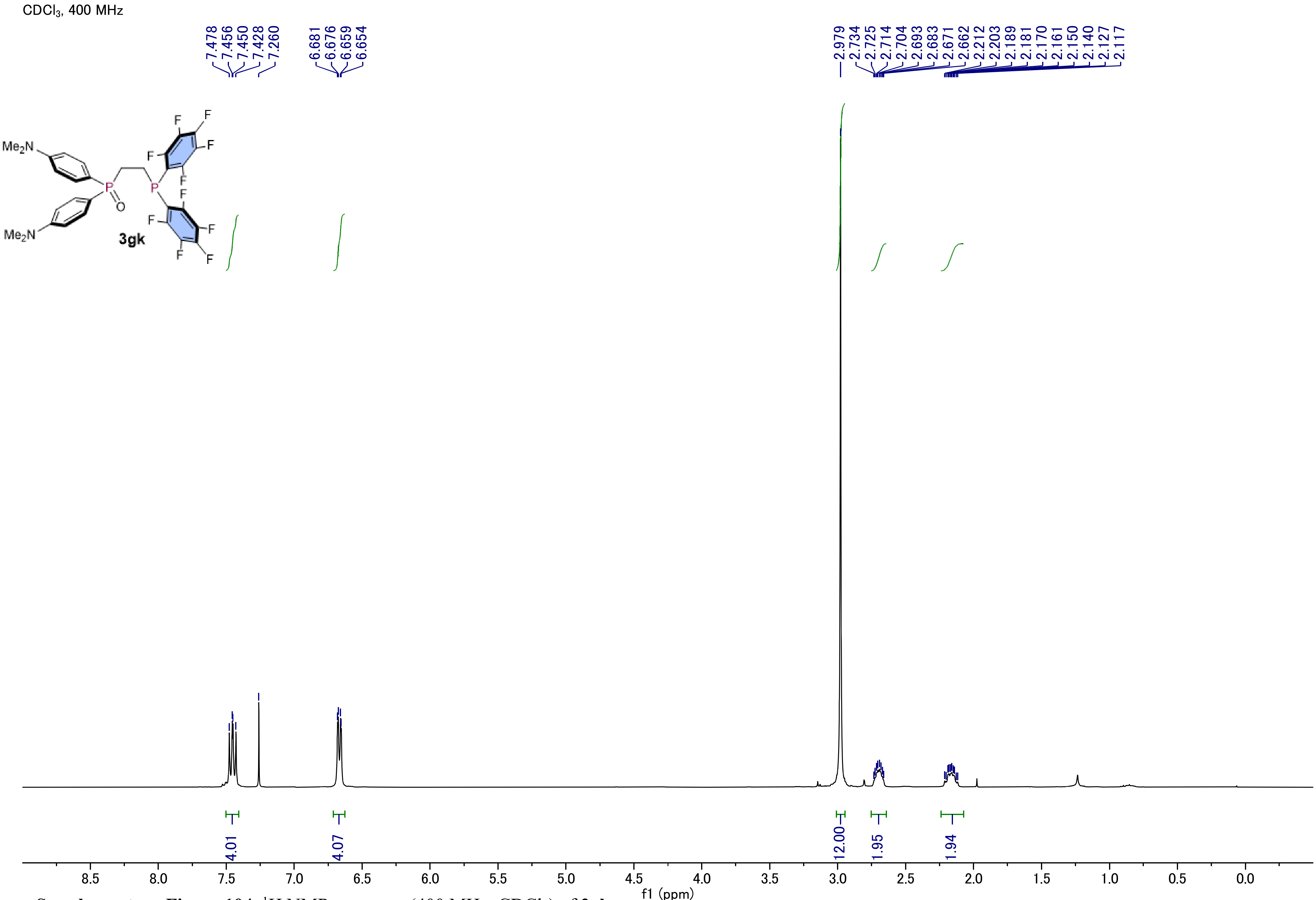

Supplementary Figure 104. ^{1}H NMR spectrum (400 MHz, $CDCl_3$) of **3gk**

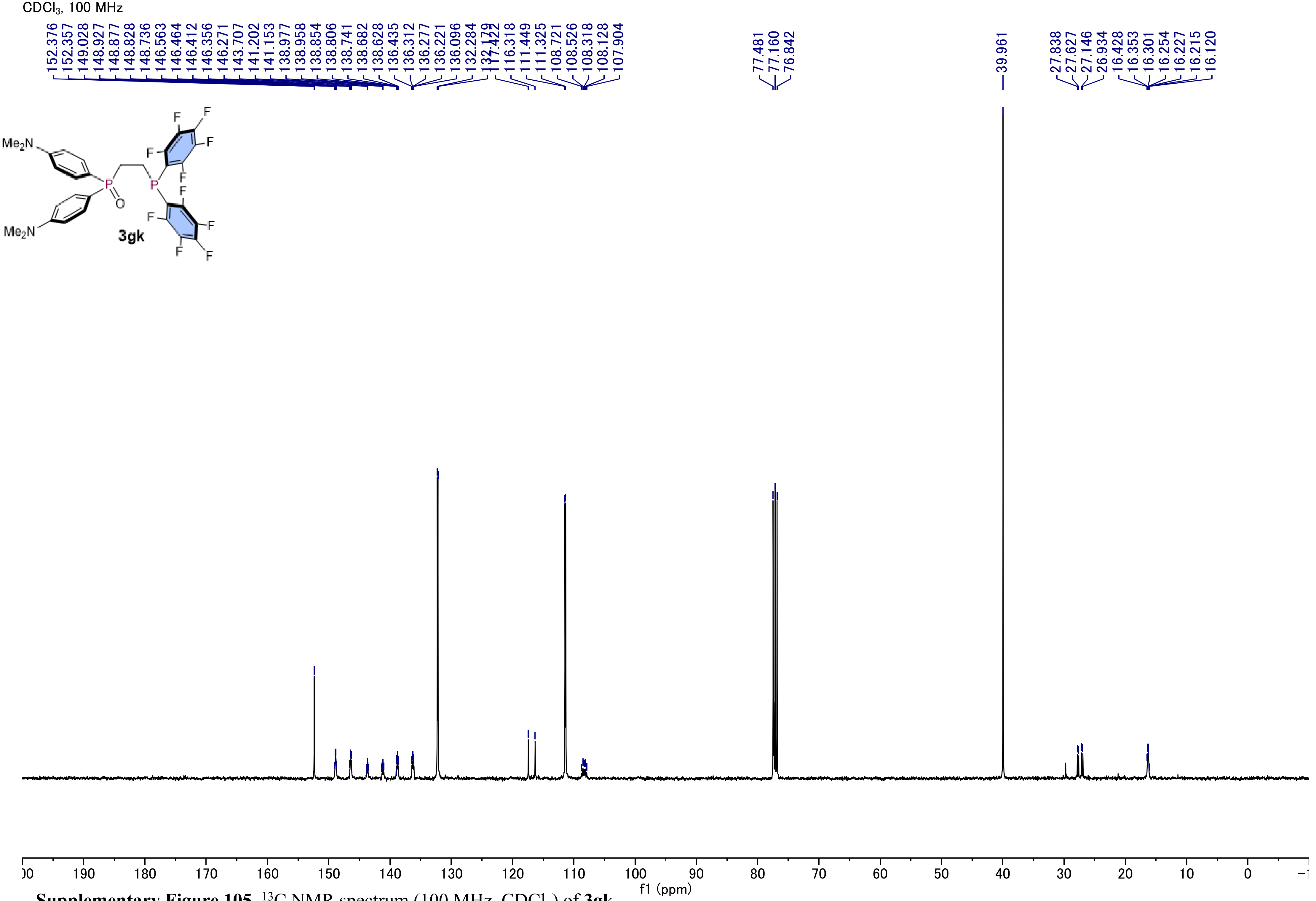

Supplementary Figure 105. ^{13}C NMR spectrum (100 MHz, $CDCl_3$) of **3gk**

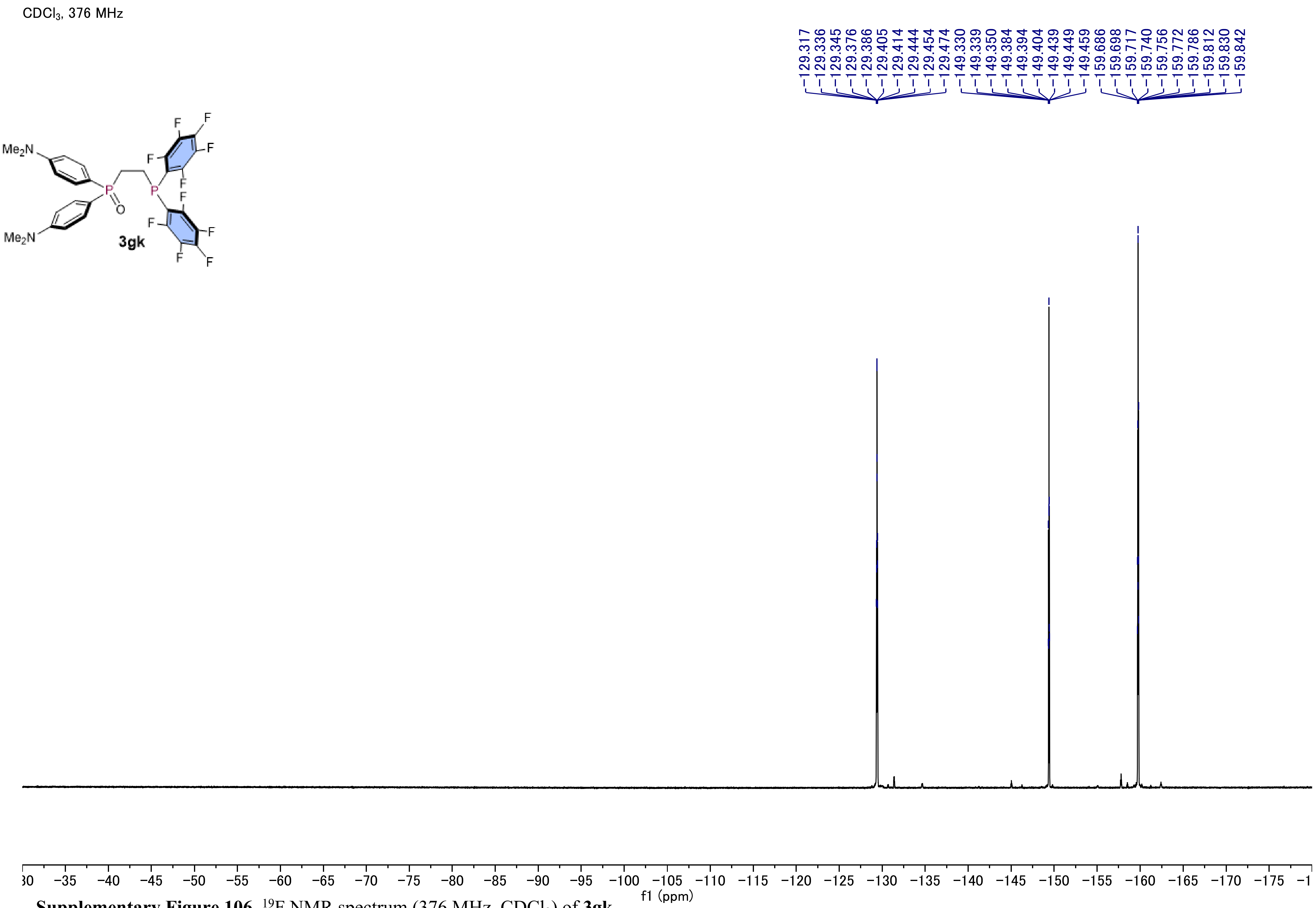

Supplementary Figure 106. ^{19}F NMR spectrum (376 MHz, $CDCl_3$) of **3gk**

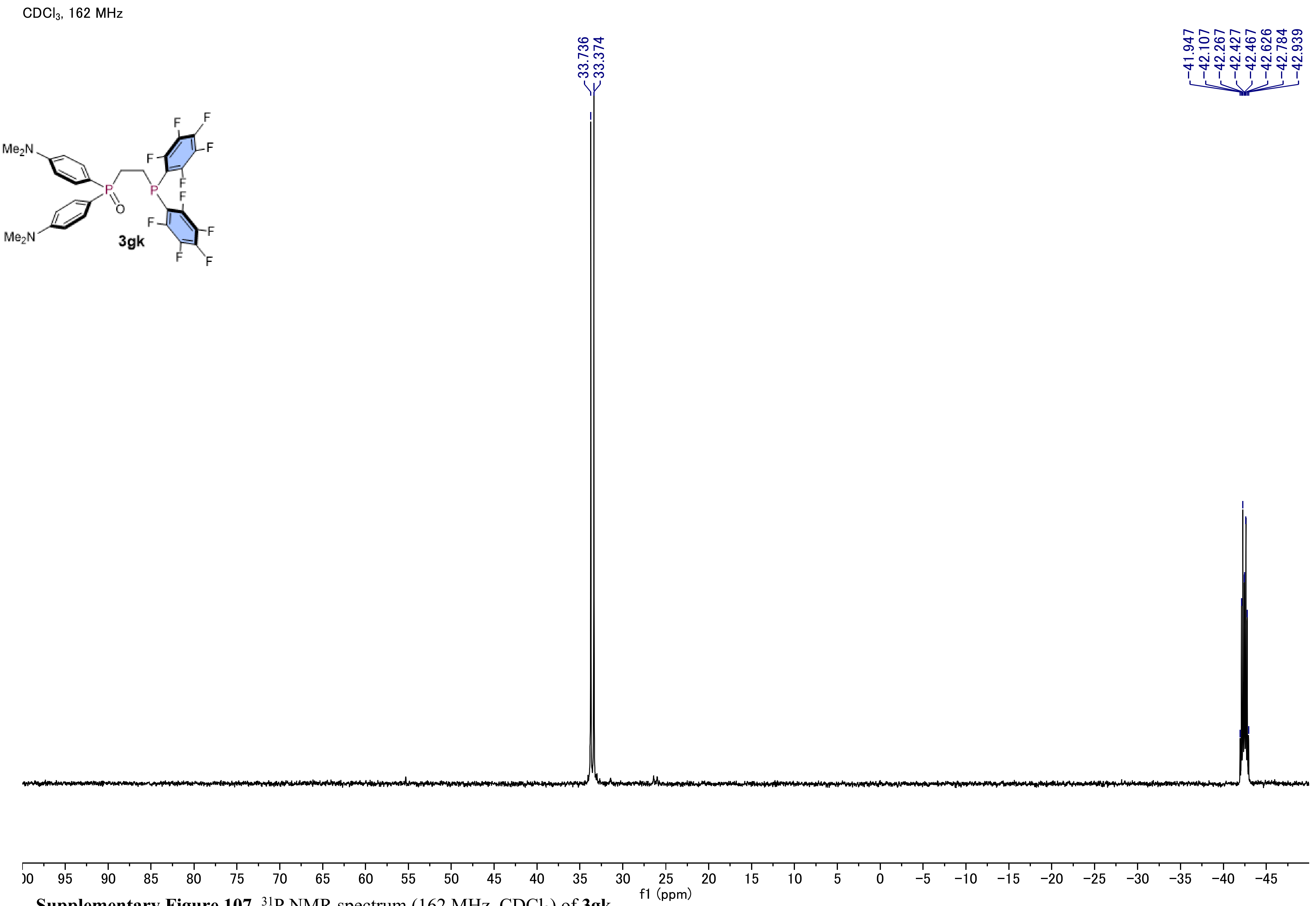

Supplementary Figure 107. ^{31}P NMR spectrum (162 MHz, $CDCl_3$) of **3gk**

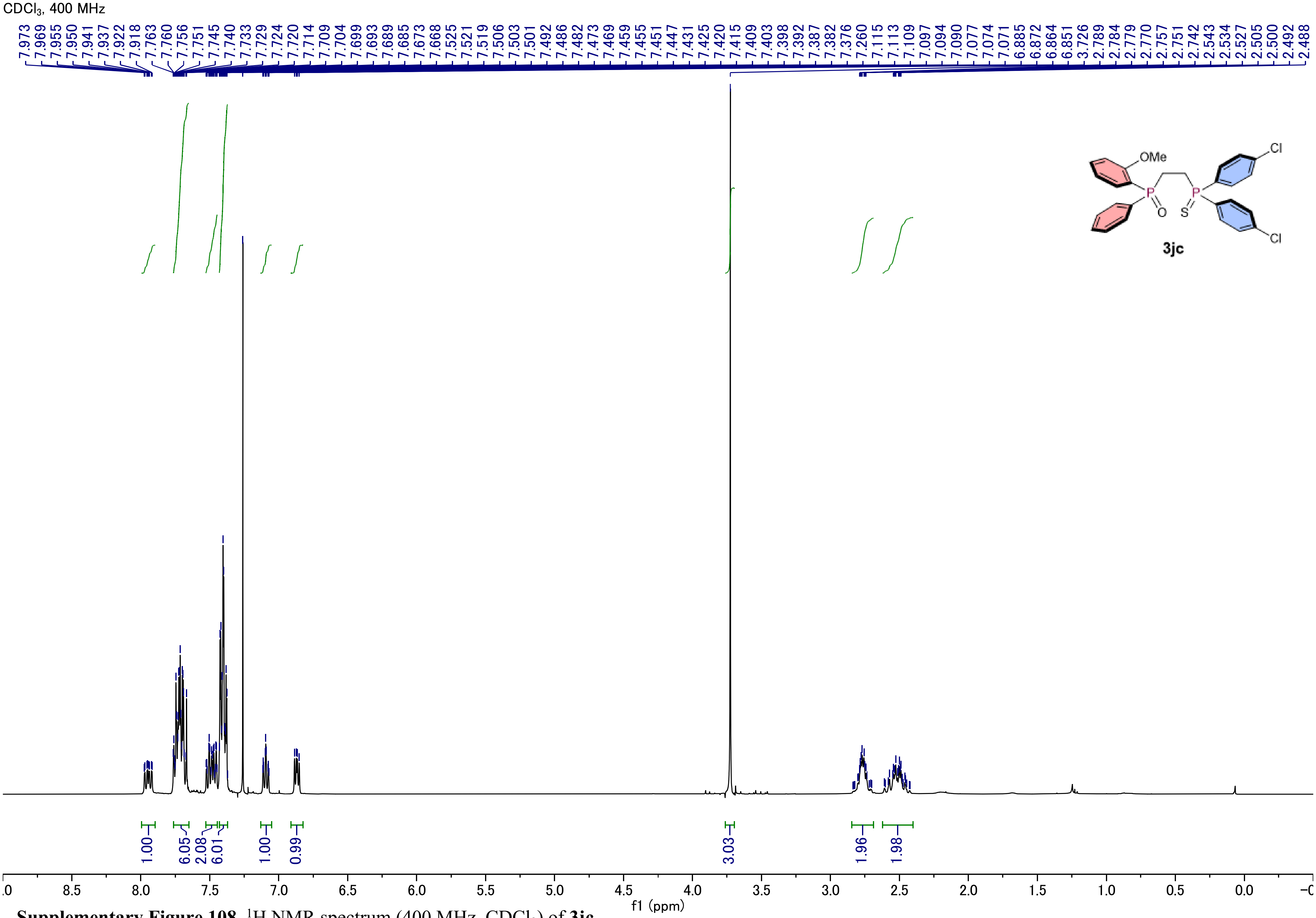

Supplementary Figure 108. ^{1}H NMR spectrum (400 MHz, $CDCl_3$) of **3jc**

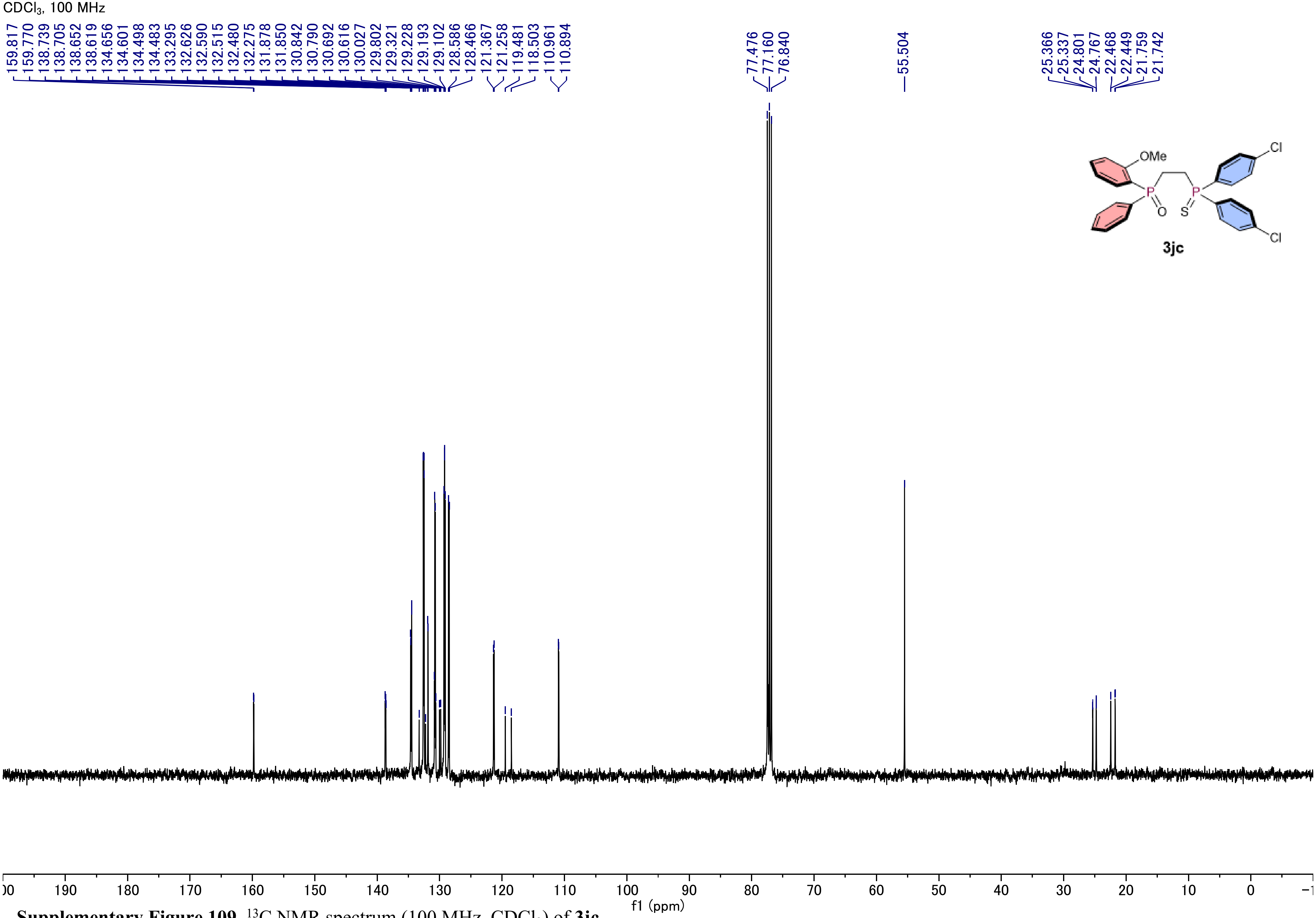

Supplementary Figure 109. ^{13}C NMR spectrum (100 MHz, $CDCl_3$) of **3jc**

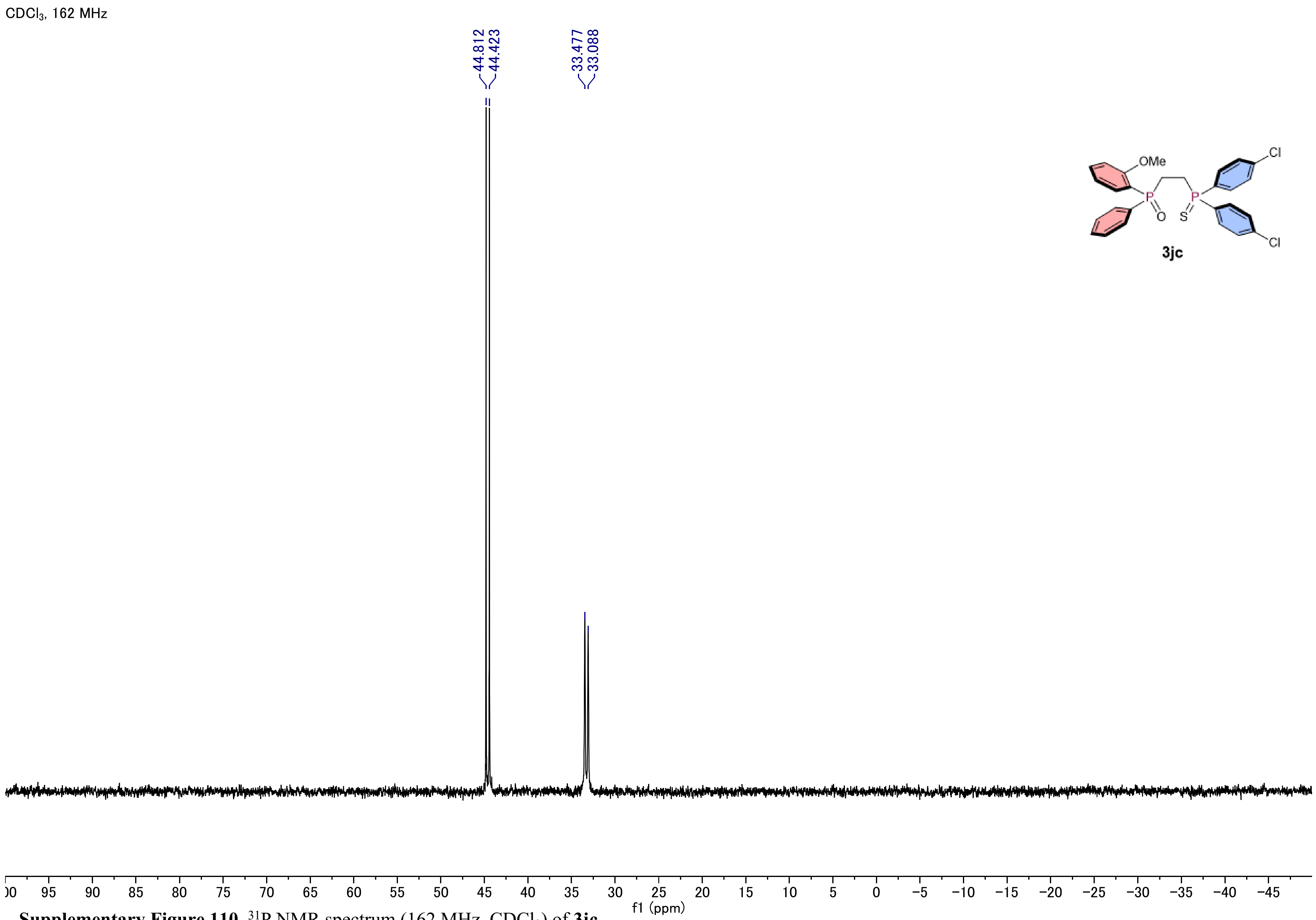

3jc

Supplementary Figure 110. ^{31}P NMR spectrum (162 MHz, $CDCl_3$) of **3jc**

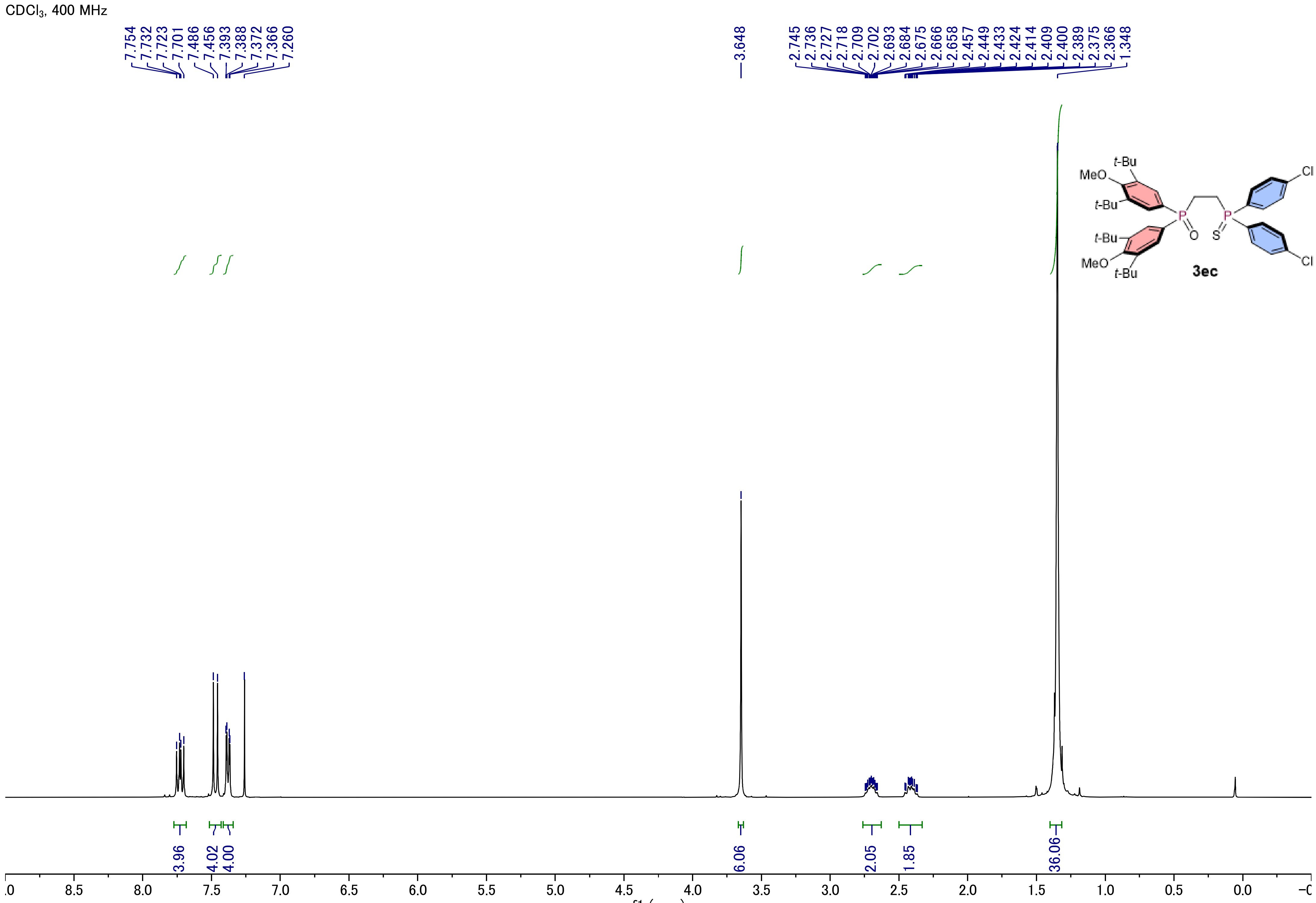

Supplementary Figure 111. ^{1}H NMR spectrum (400 MHz, $CDCl_3$) of **3ec**

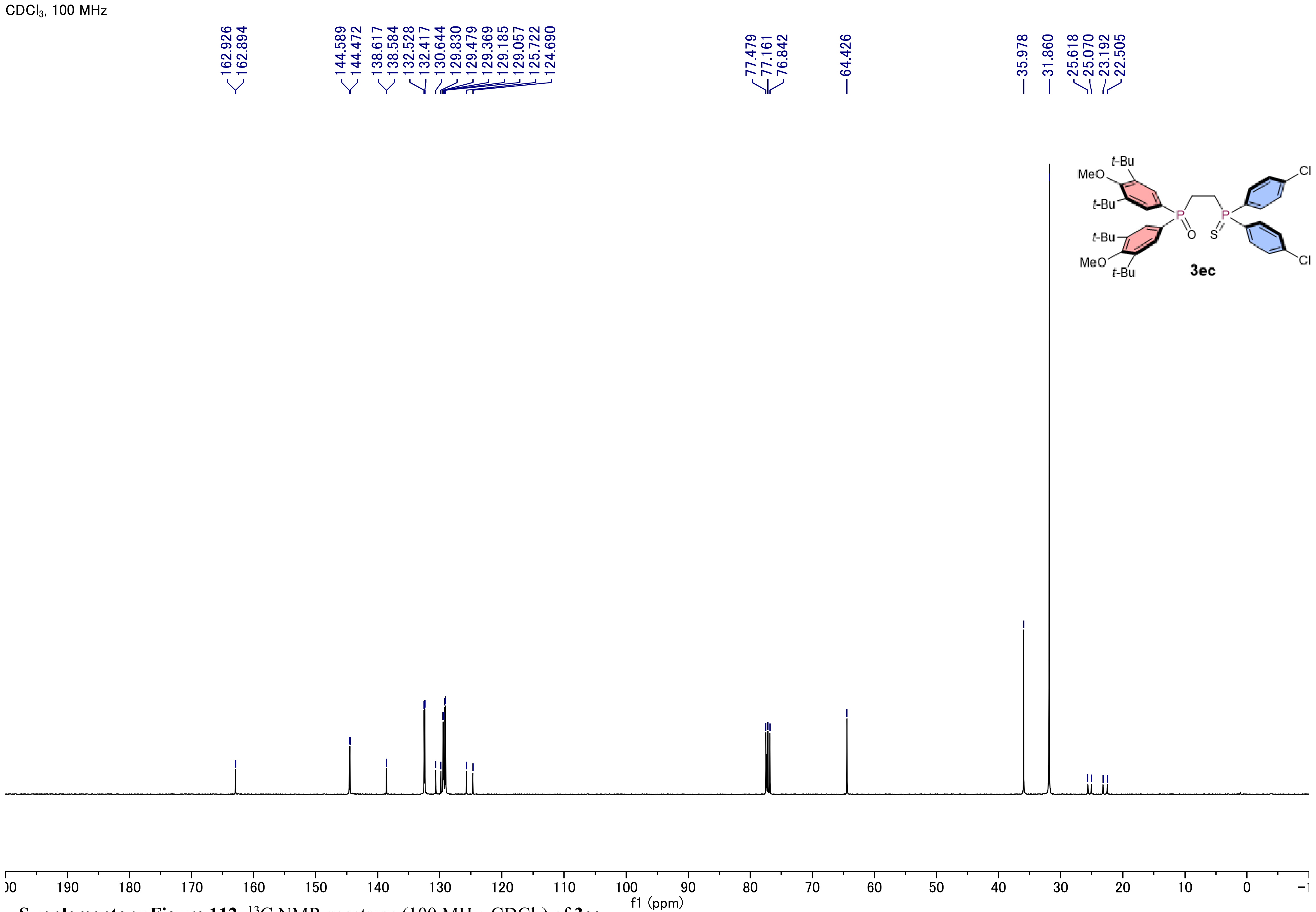

Supplementary Figure 112. ^{13}C NMR spectrum (100 MHz, $CDCl_3$) of **3ec**

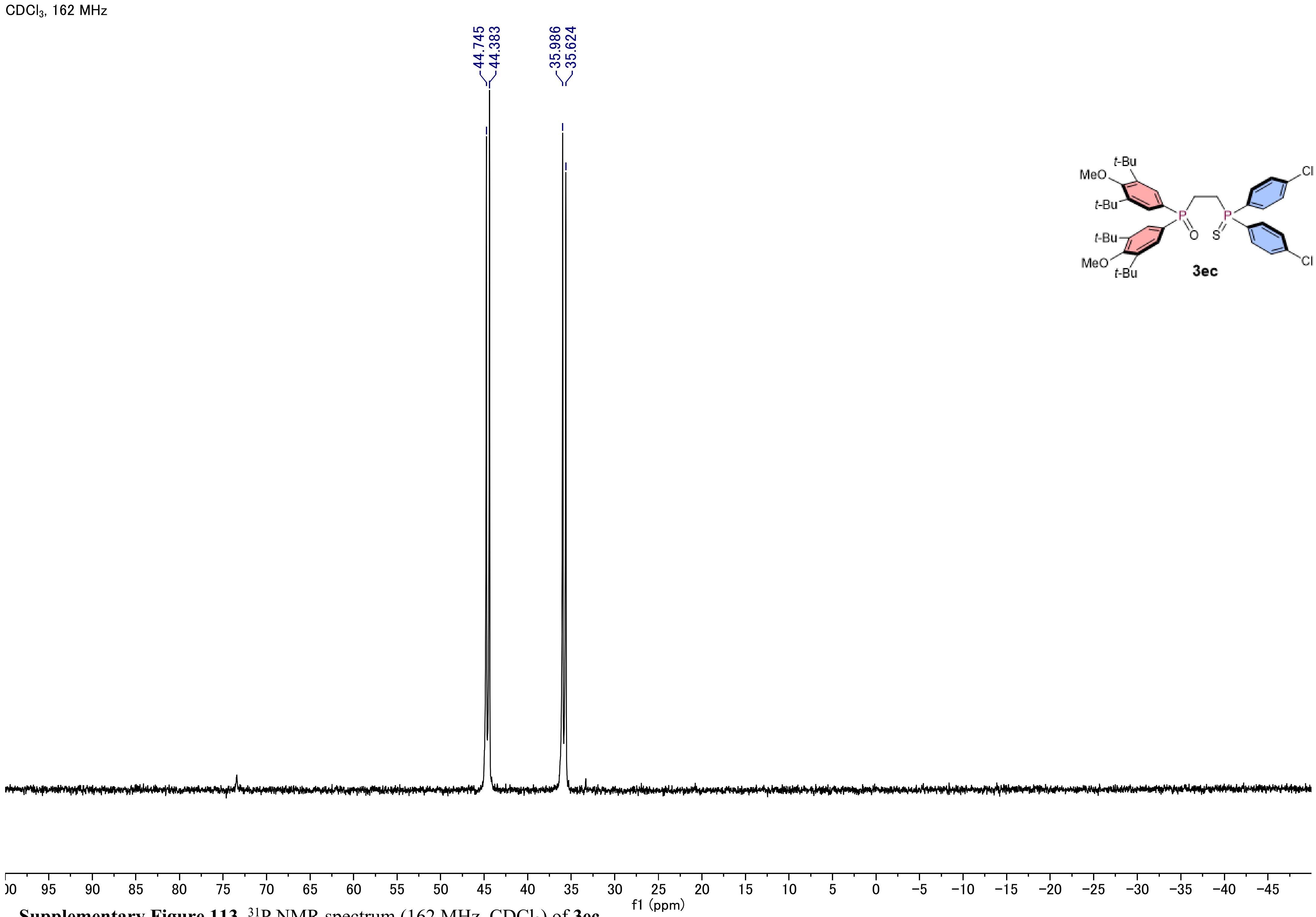

Supplementary Figure 113. ^{31}P NMR spectrum (162 MHz, $CDCl_3$) of **3ec**

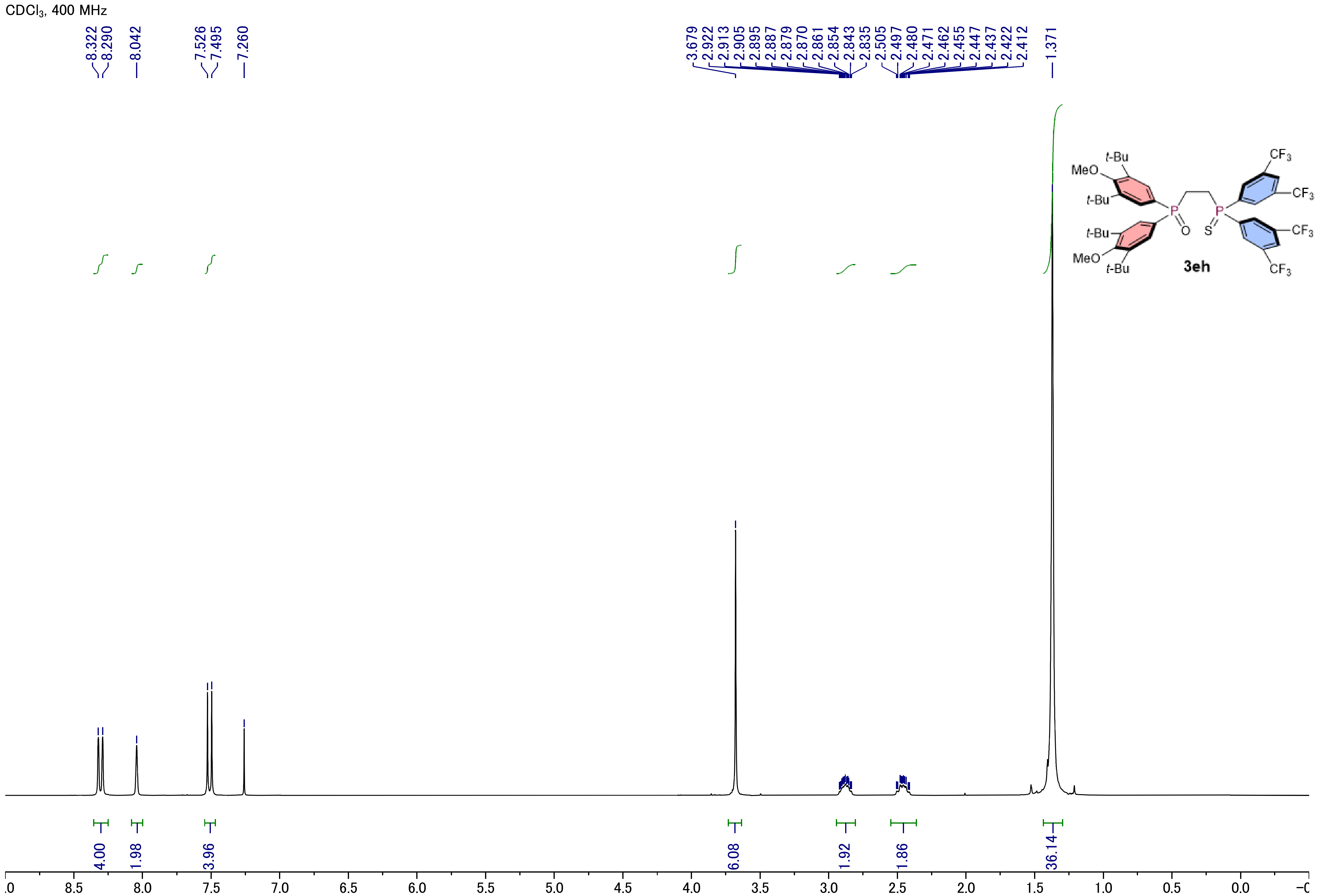

Supplementary Figure 114. ^1H NMR spectrum (400 MHz, $CDCl_3$) of **3eh**

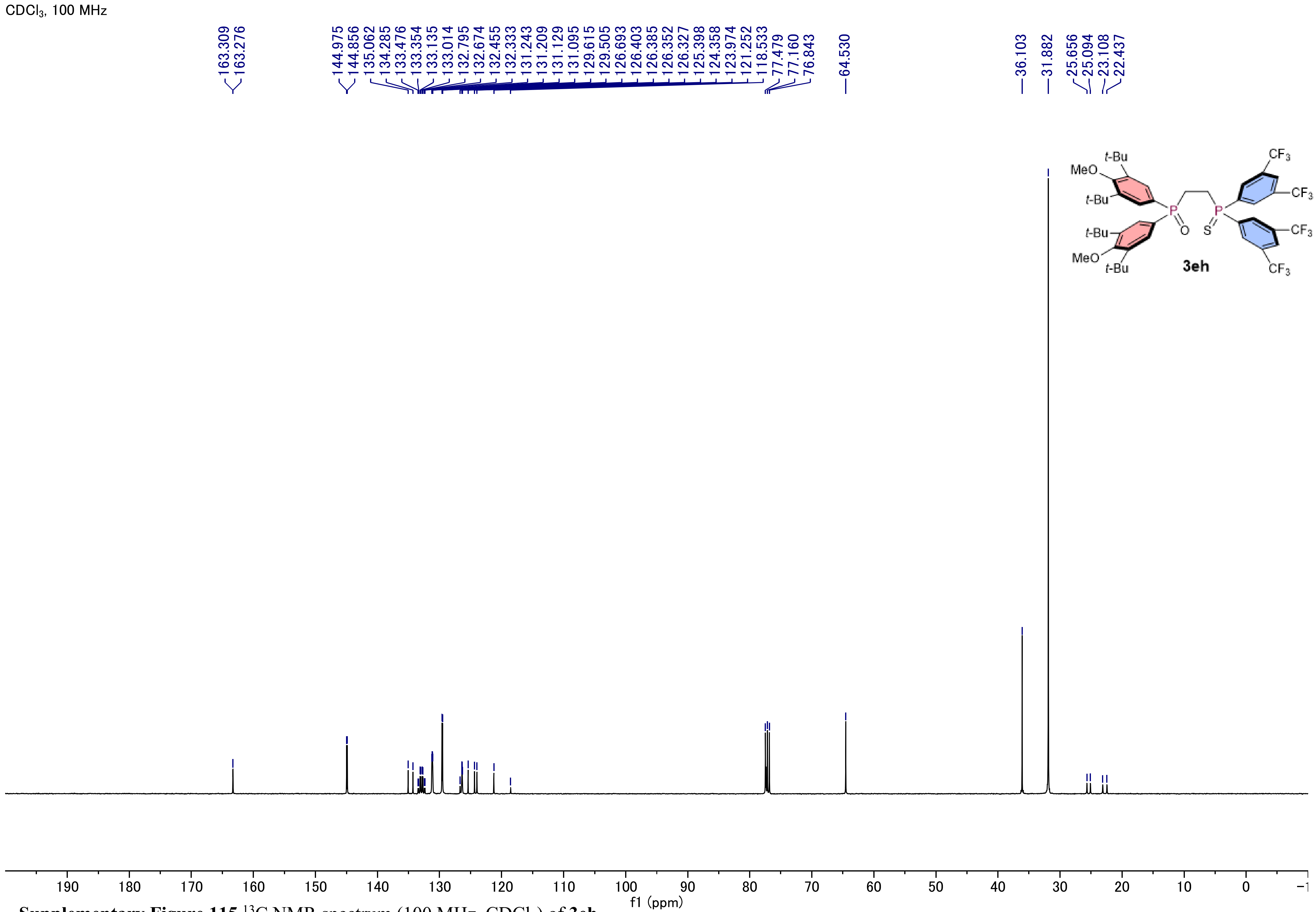

Supplementary Figure 115 ^{13}C NMR spectrum (100 MHz, $CDCl_3$) of **3eh**

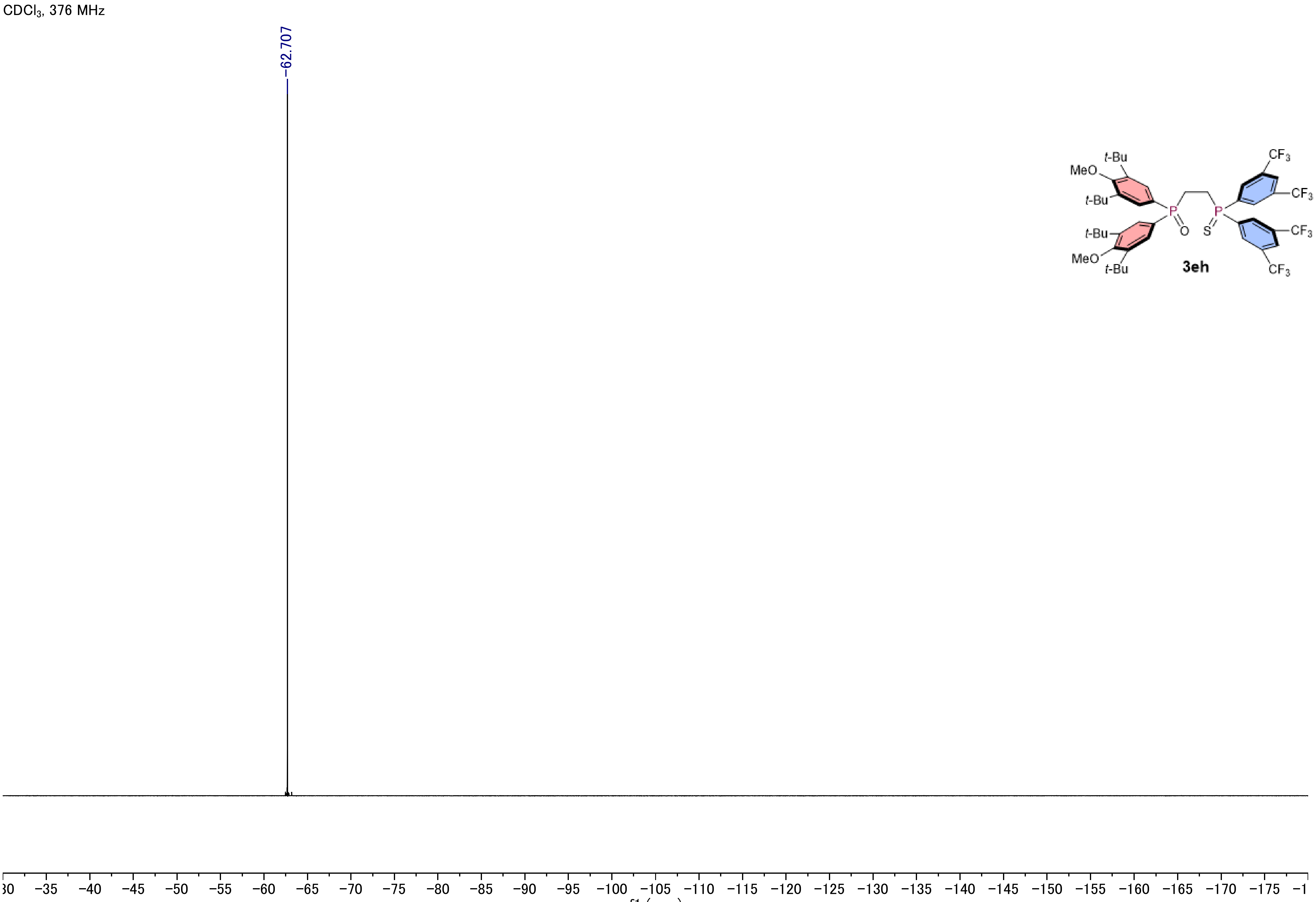

Supplementary Figure 116. ^{19}F NMR spectrum (376 MHz, $CDCl_3$) of **3eh**

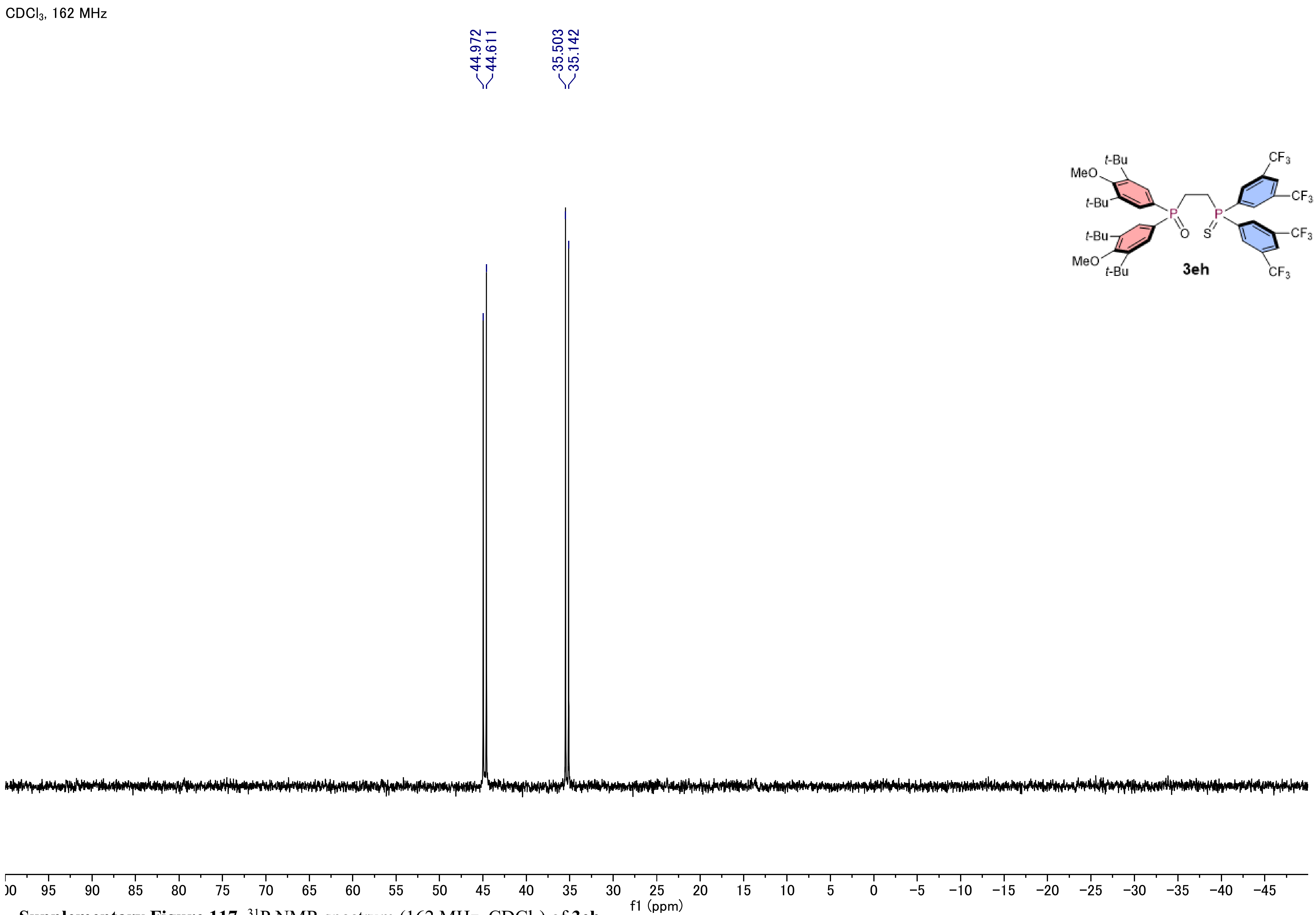

Supplementary Figure 117. ^{31}P NMR spectrum (162 MHz, $CDCl_3$) of **3eh**

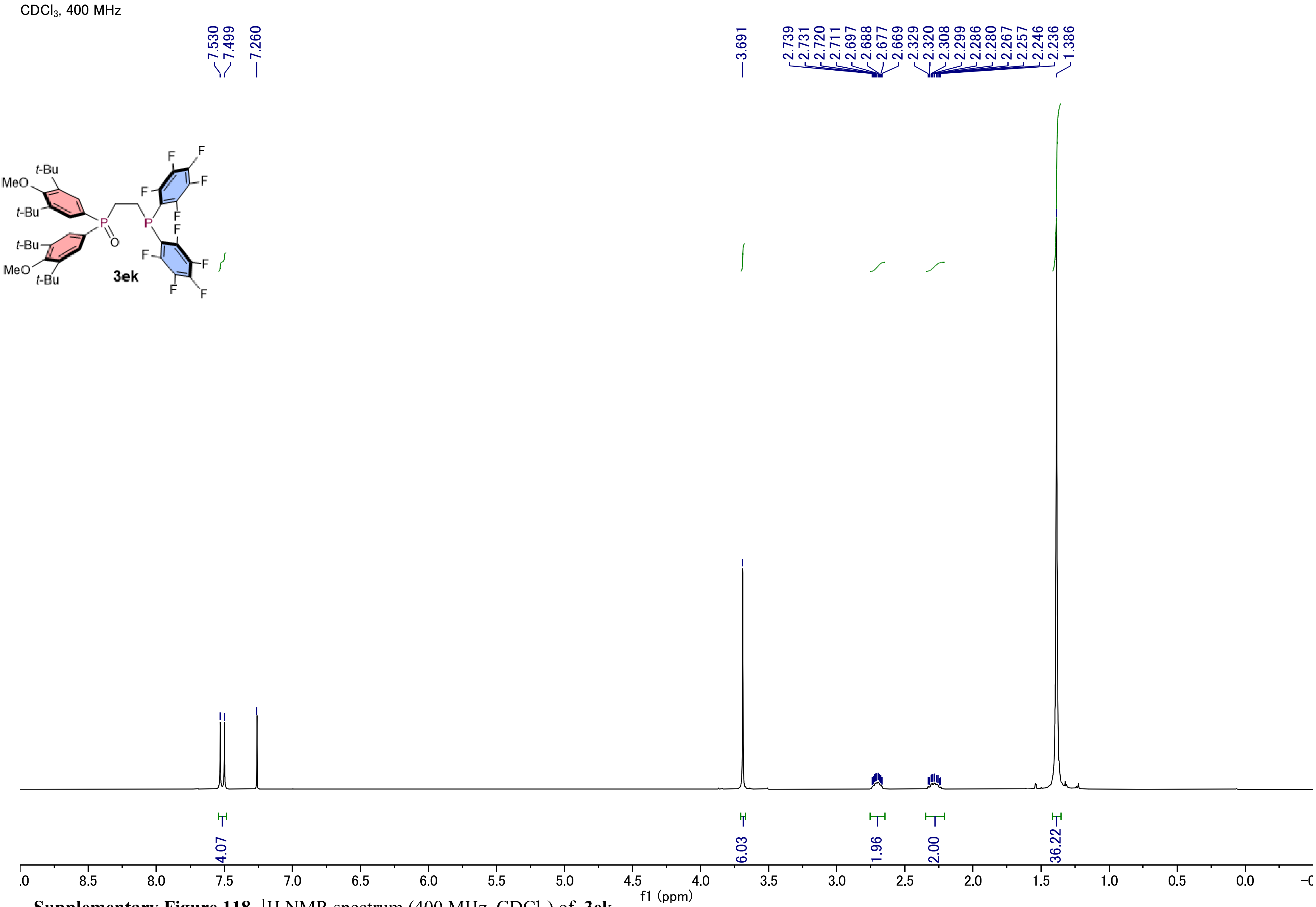

Supplementary Figure 118. ^{1}H NMR spectrum (400 MHz, $CDCl_3$) of **3ek**

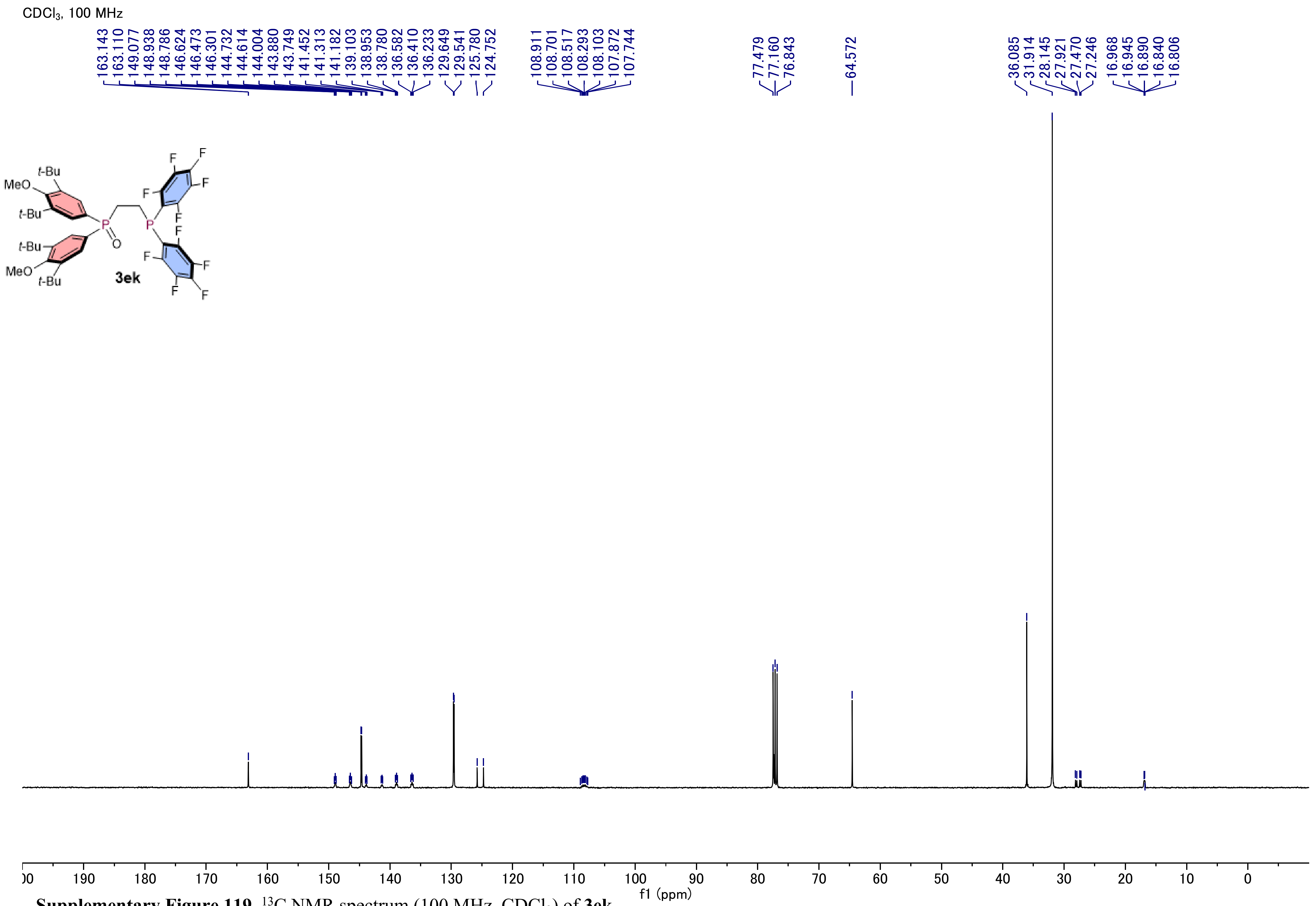

Supplementary Figure 119. ^{13}C NMR spectrum (100 MHz, $CDCl_3$) of **3ek**

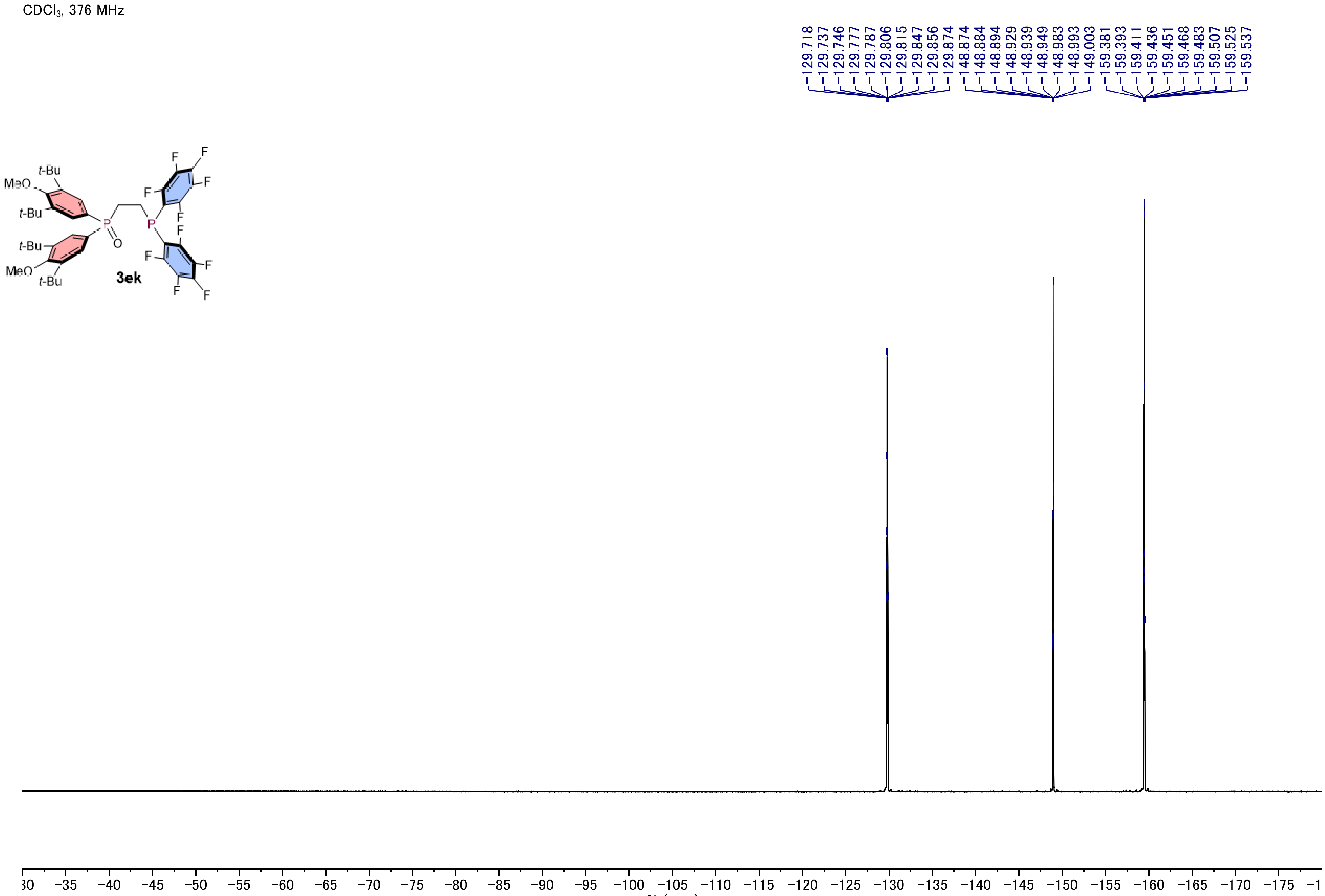

Supplementary Figure 120. ^{19}F NMR spectrum (376 MHz, $CDCl_3$) of **3ek**

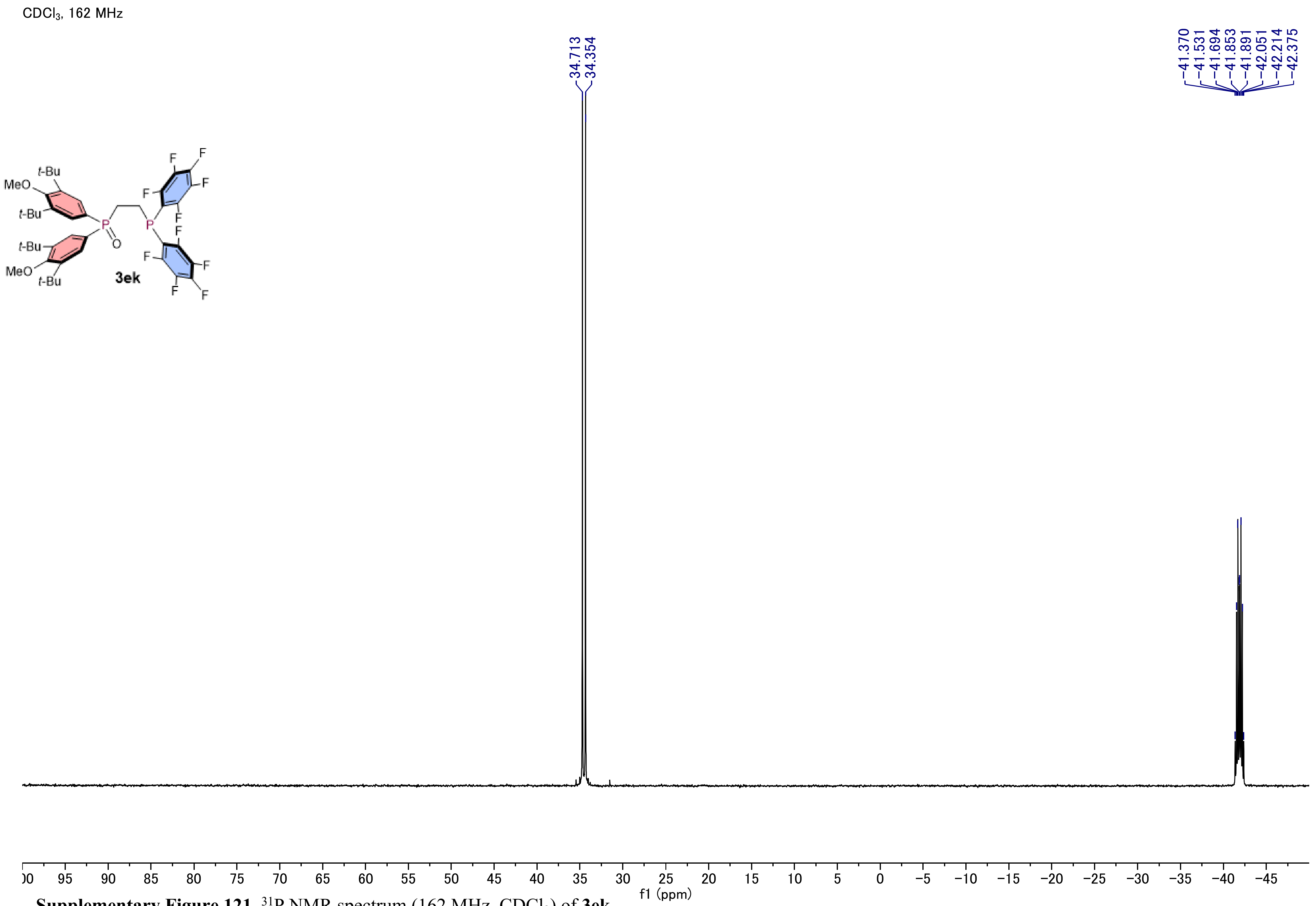

Supplementary Figure 121. ^{31}P NMR spectrum (162 MHz, $CDCl_3$) of **3ek**

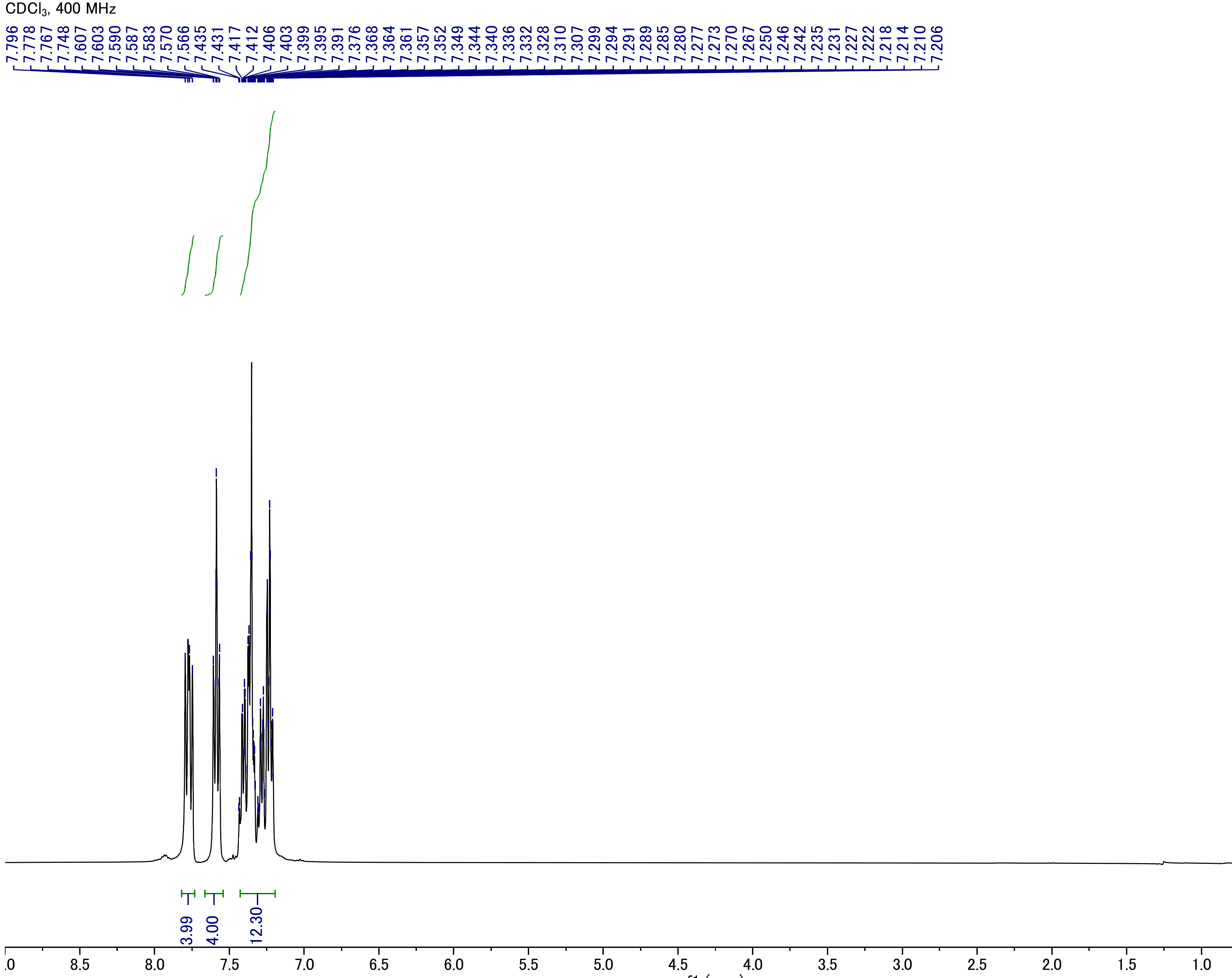

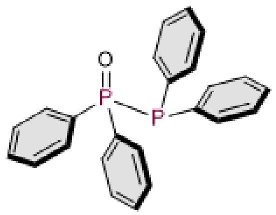

4aa

Supplementary Figure 122. ^{1}H NMR spectrum (400 MHz, $CDCl_3$) of **4aa**

CDCl$_3$, 100 MHz

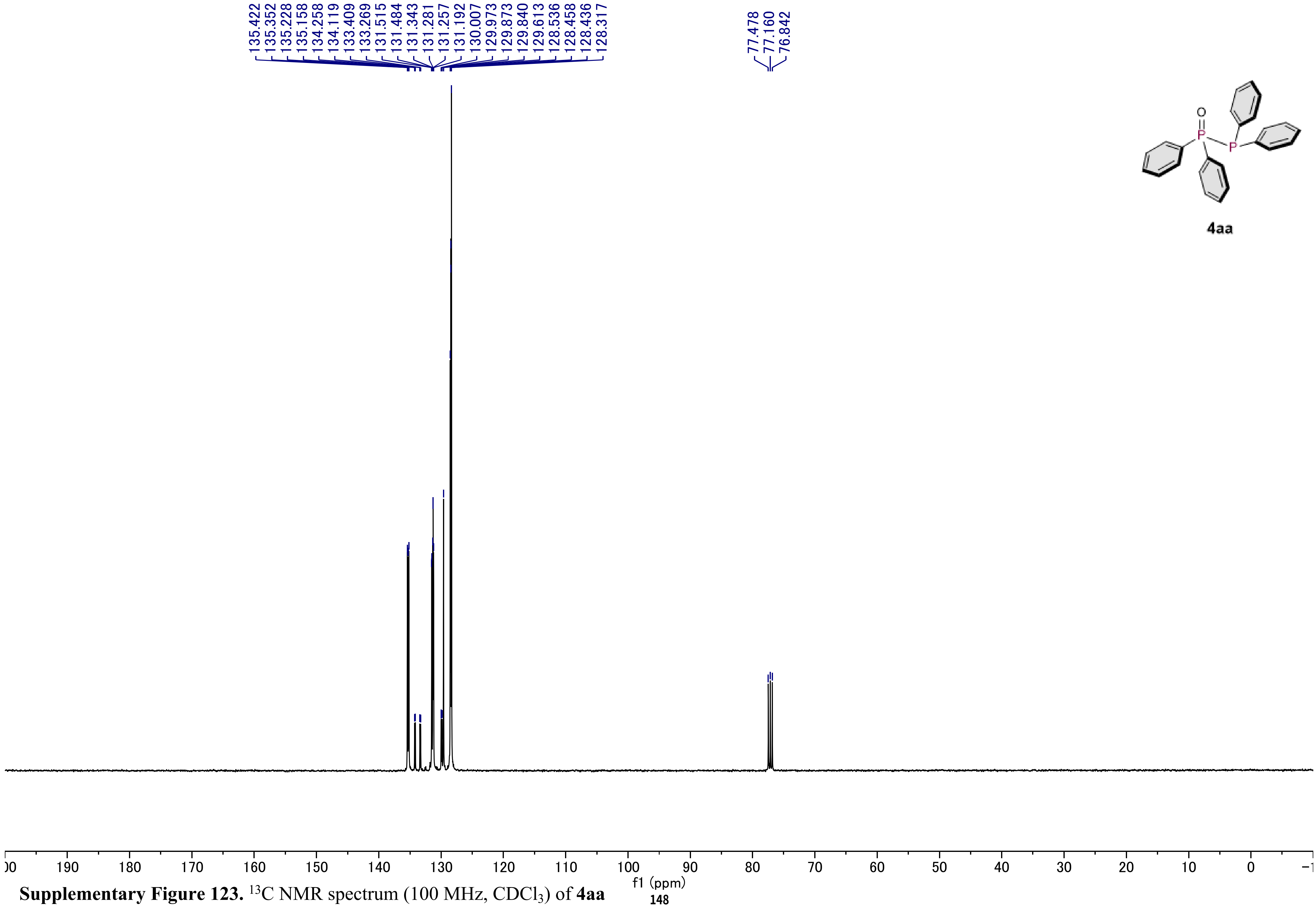

4aa

Supplementary Figure 123. ^{13}C NMR spectrum (100 MHz, CDCl$_3$) of **4aa**

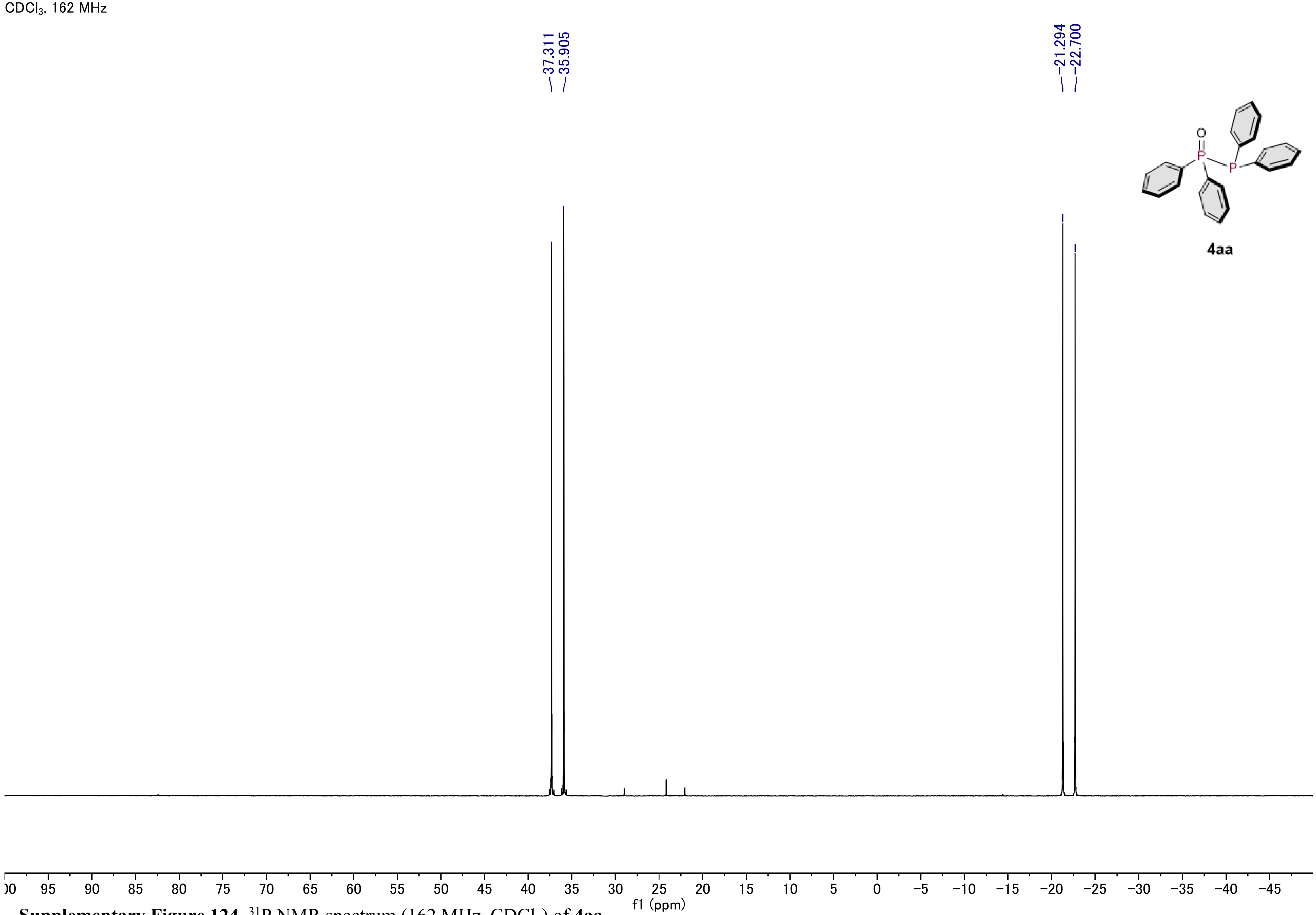

Supplementary Figure 124. ^{31}P NMR spectrum (162 MHz, $CDCl_3$) of **4aa**

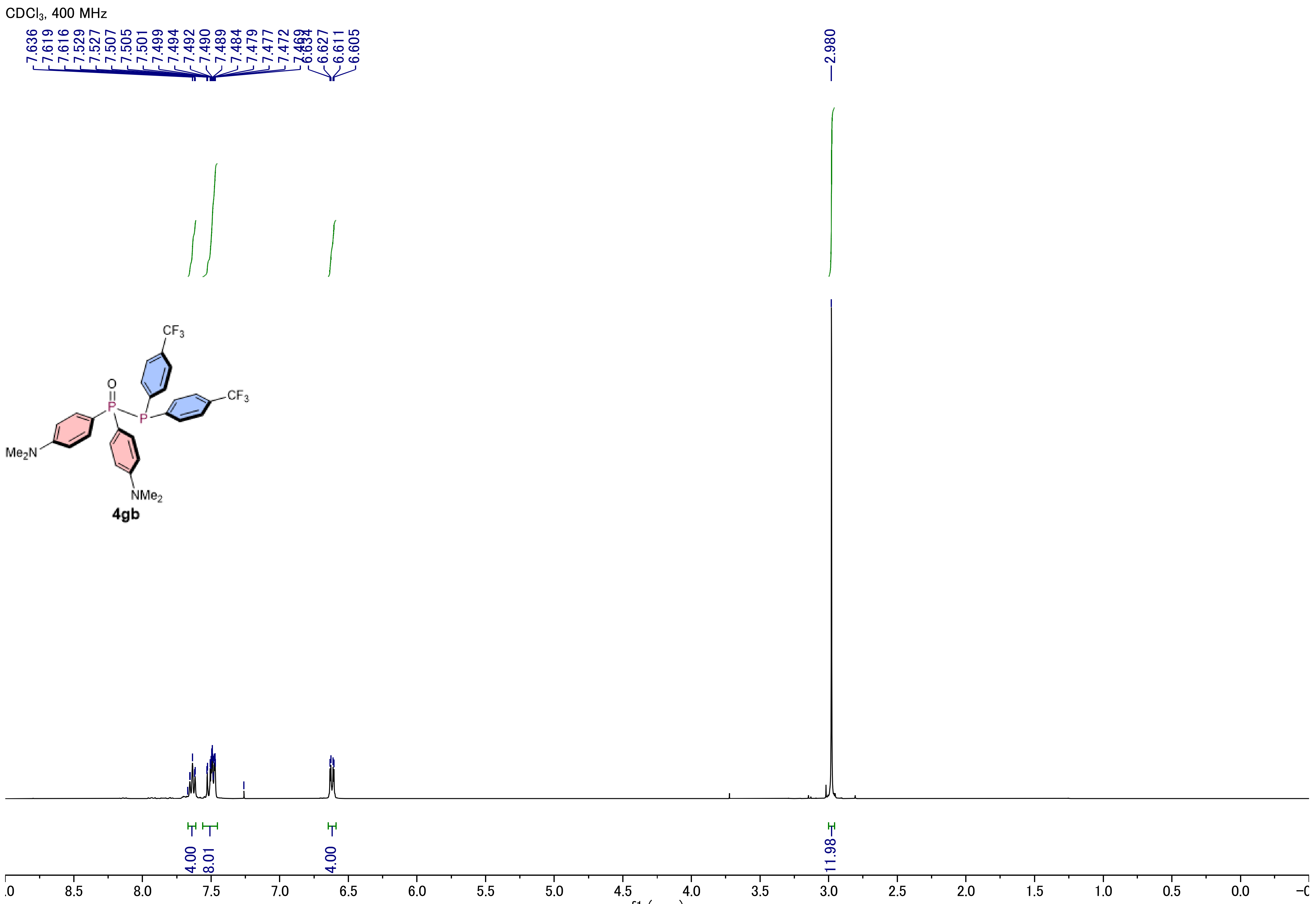

Supplementary Figure 125. ^{1}H NMR spectrum (400 MHz, $CDCl_3$) of **4gb**

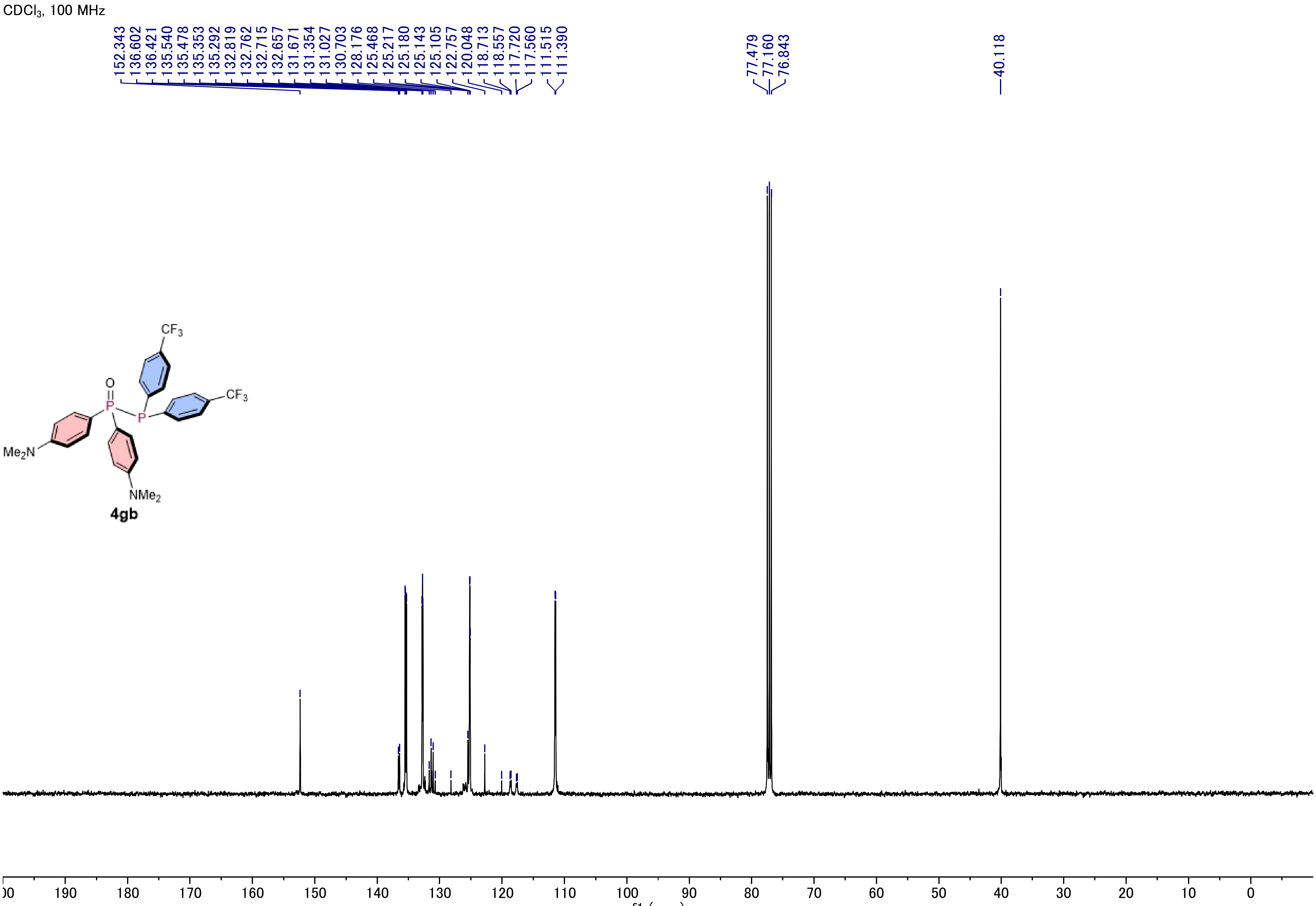

Supplementary Figure 126. ^{13}C NMR spectrum (100 MHz, $CDCl_3$) of **4gb**

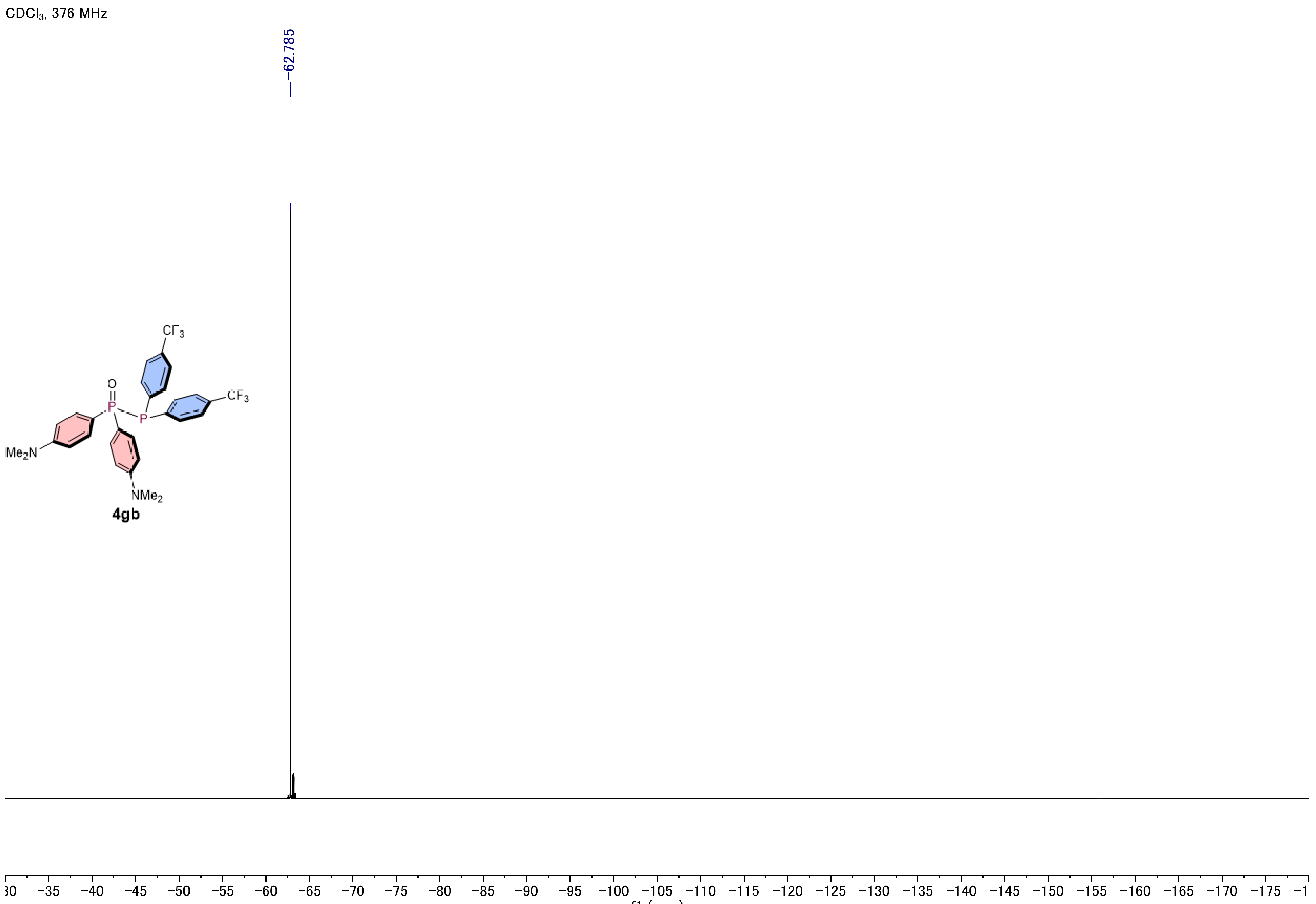

Supplementary Figure 127. ^{19}F NMR spectrum (376 MHz, $CDCl_3$) of **4gb**

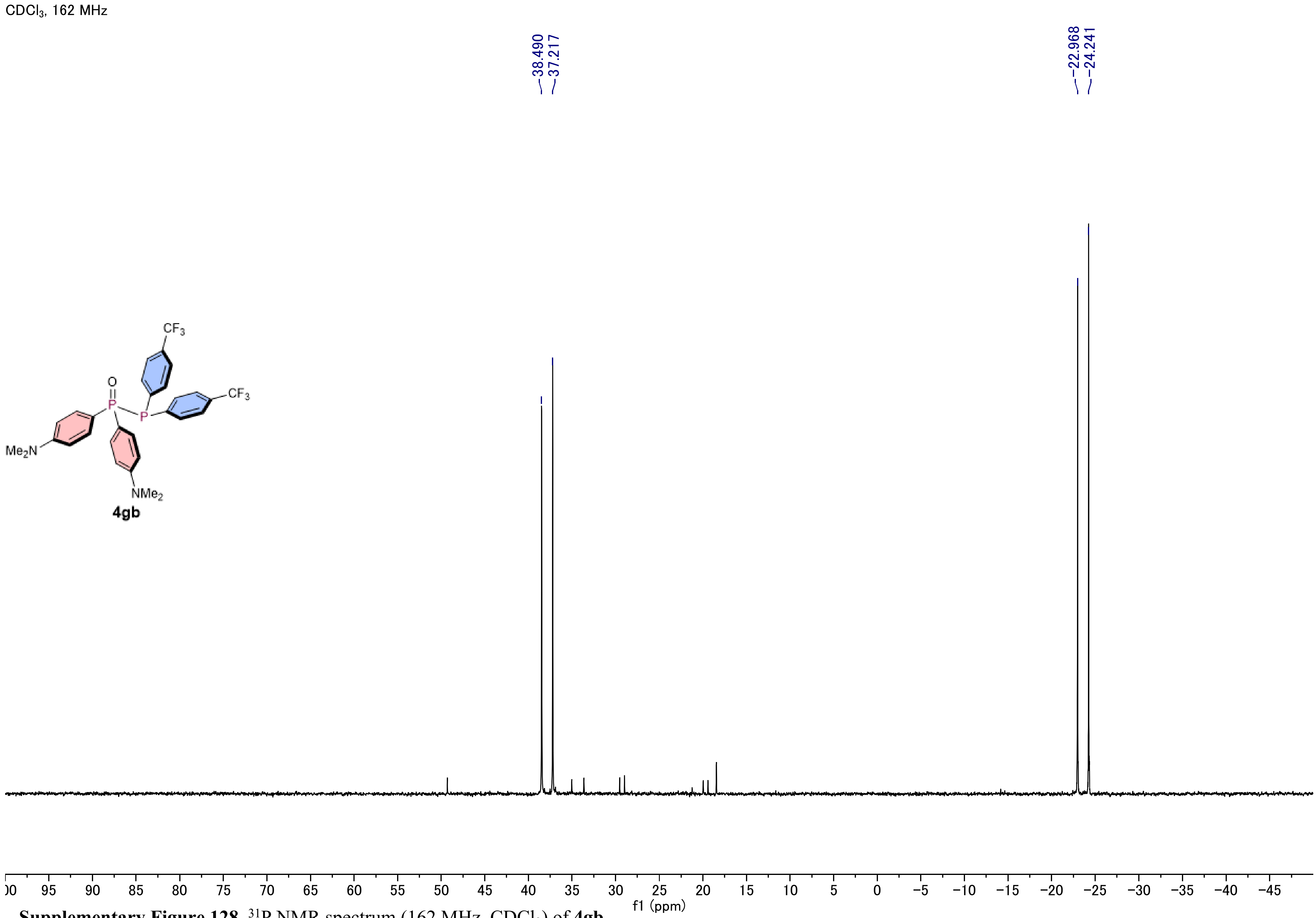

Supplementary Figure 128. ^{31}P NMR spectrum (162 MHz, $CDCl_3$) of **4gb**

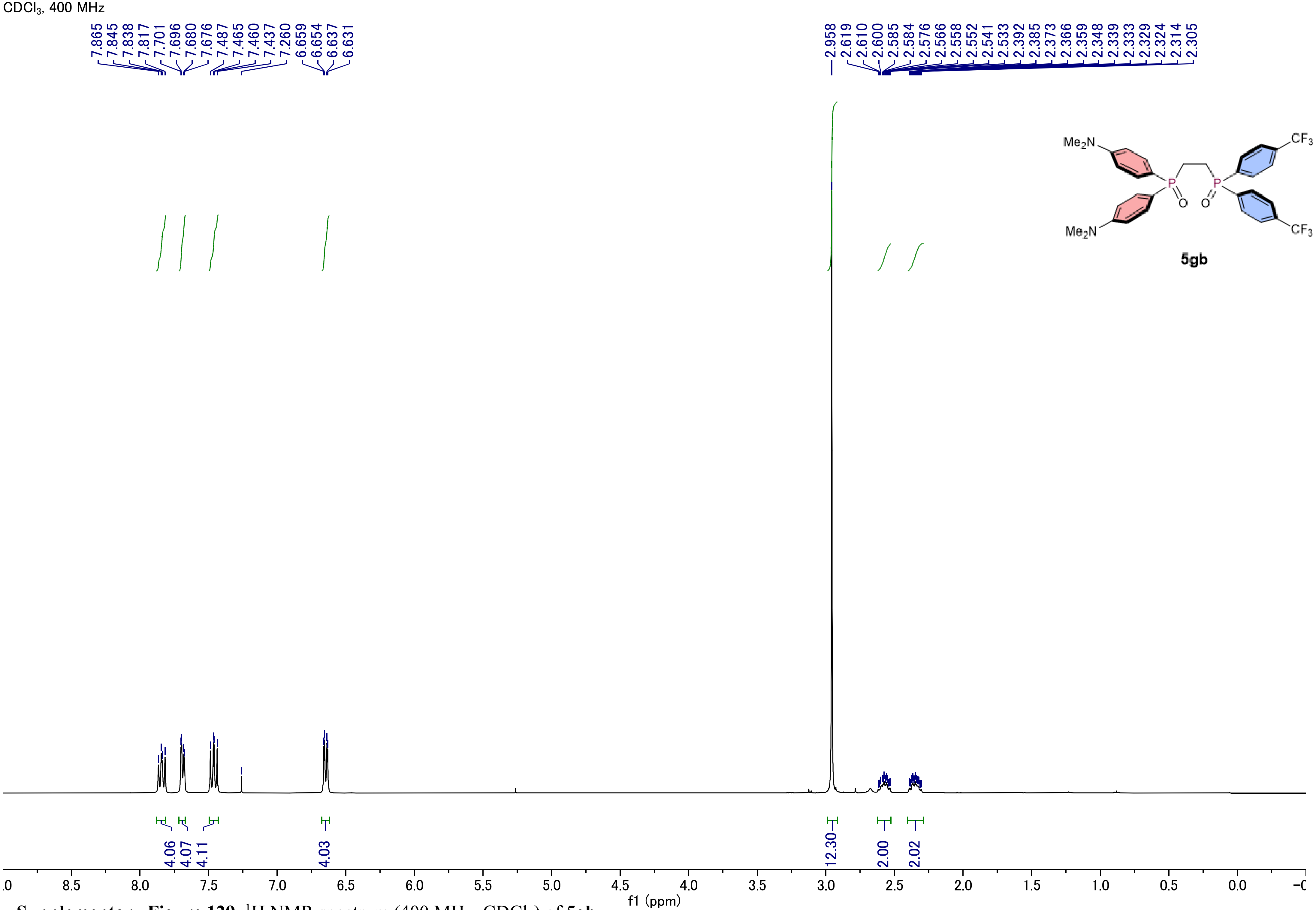

Supplementary Figure 129. ^{1}H NMR spectrum (400 MHz, $CDCl_3$) of **5gb**

CDCl3, 100 MHz

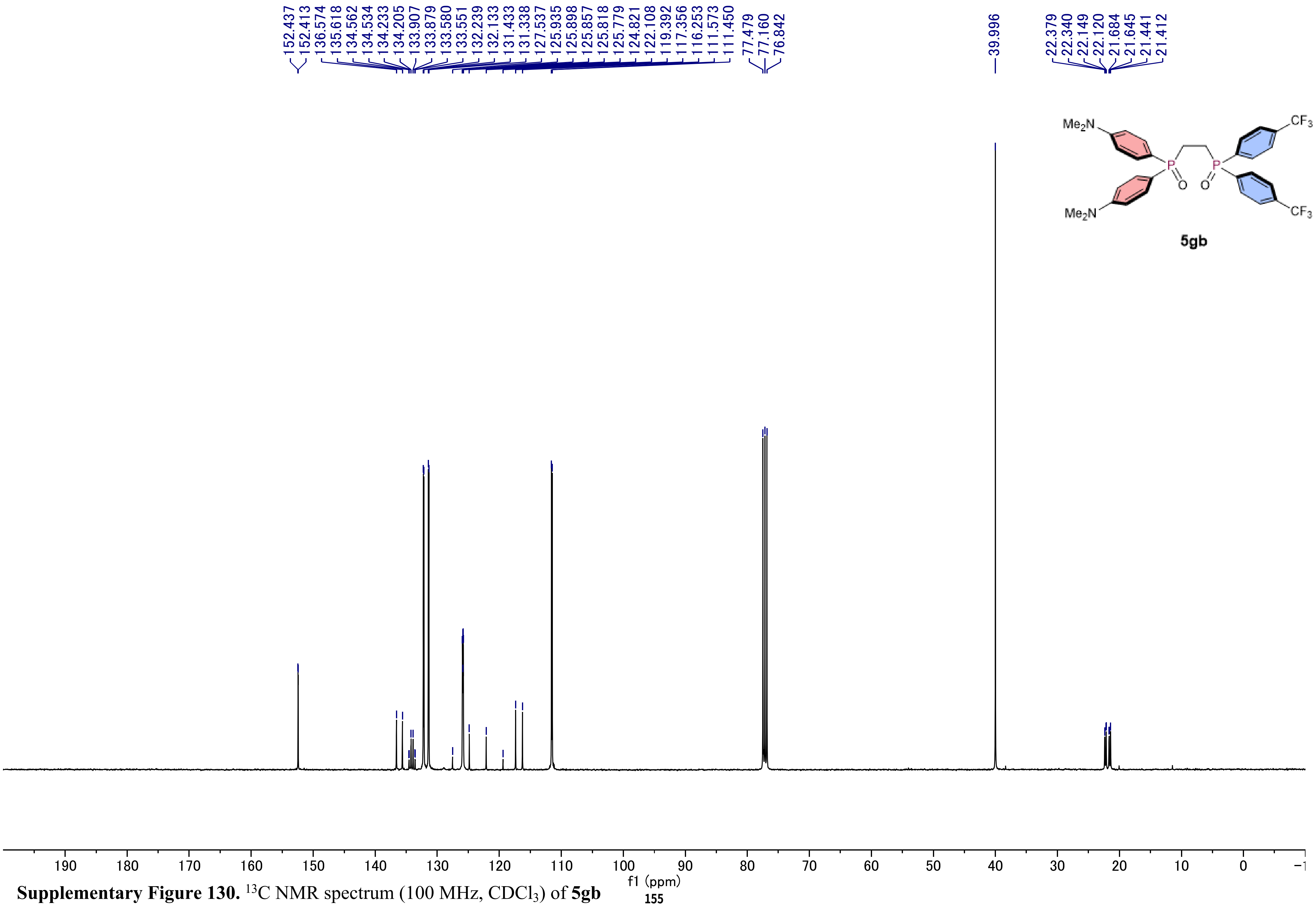

Supplementary Figure 130. ^{13}C NMR spectrum (100 MHz, $CDCl_3$) of **5gb**

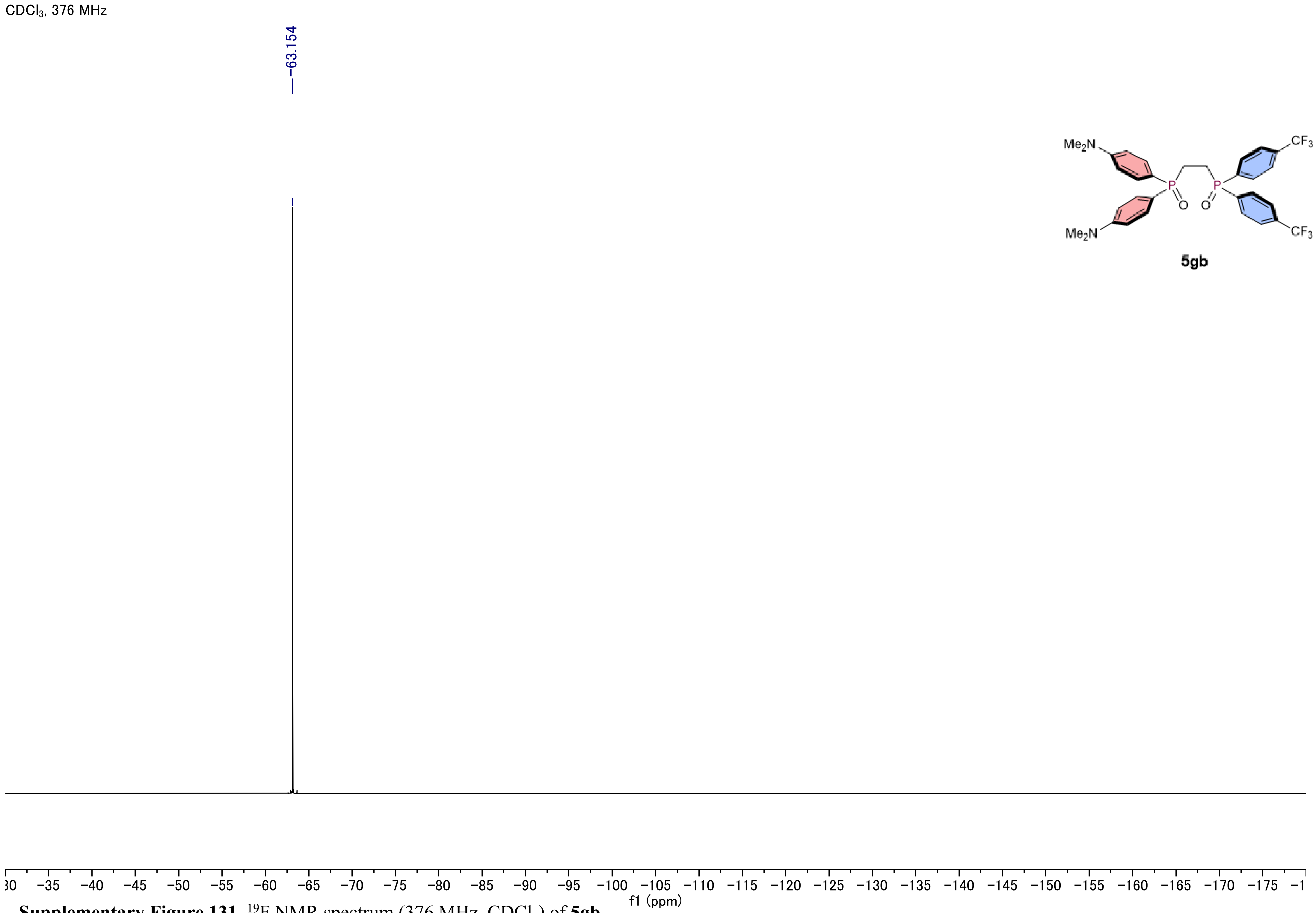

Supplementary Figure 131. ^{19}F NMR spectrum (376 MHz, $CDCl_3$) of **5gb**

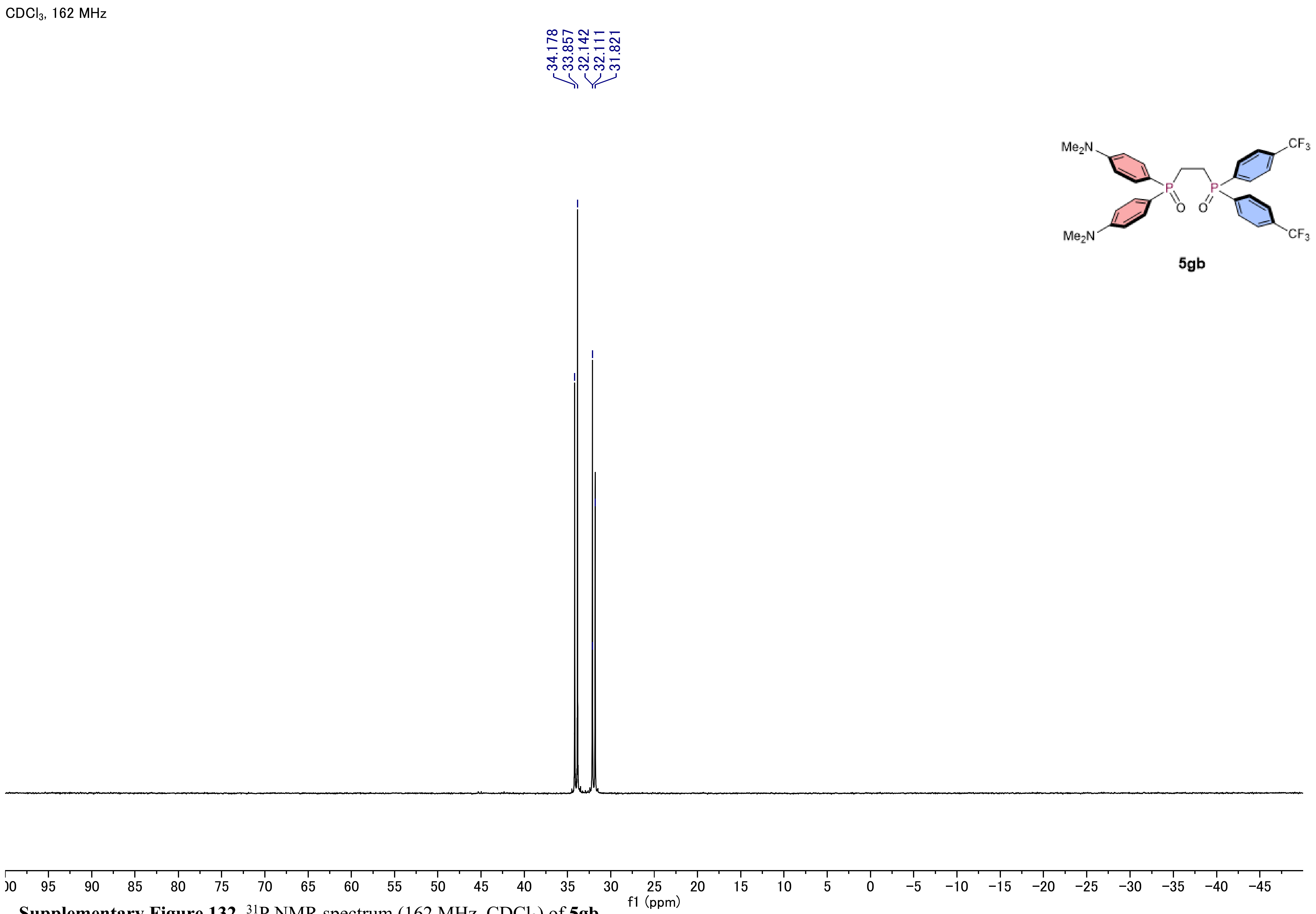

Supplementary Figure 132. ^{31}P NMR spectrum (162 MHz, $CDCl_3$) of **5gb**

CDCl3, 400 MHz

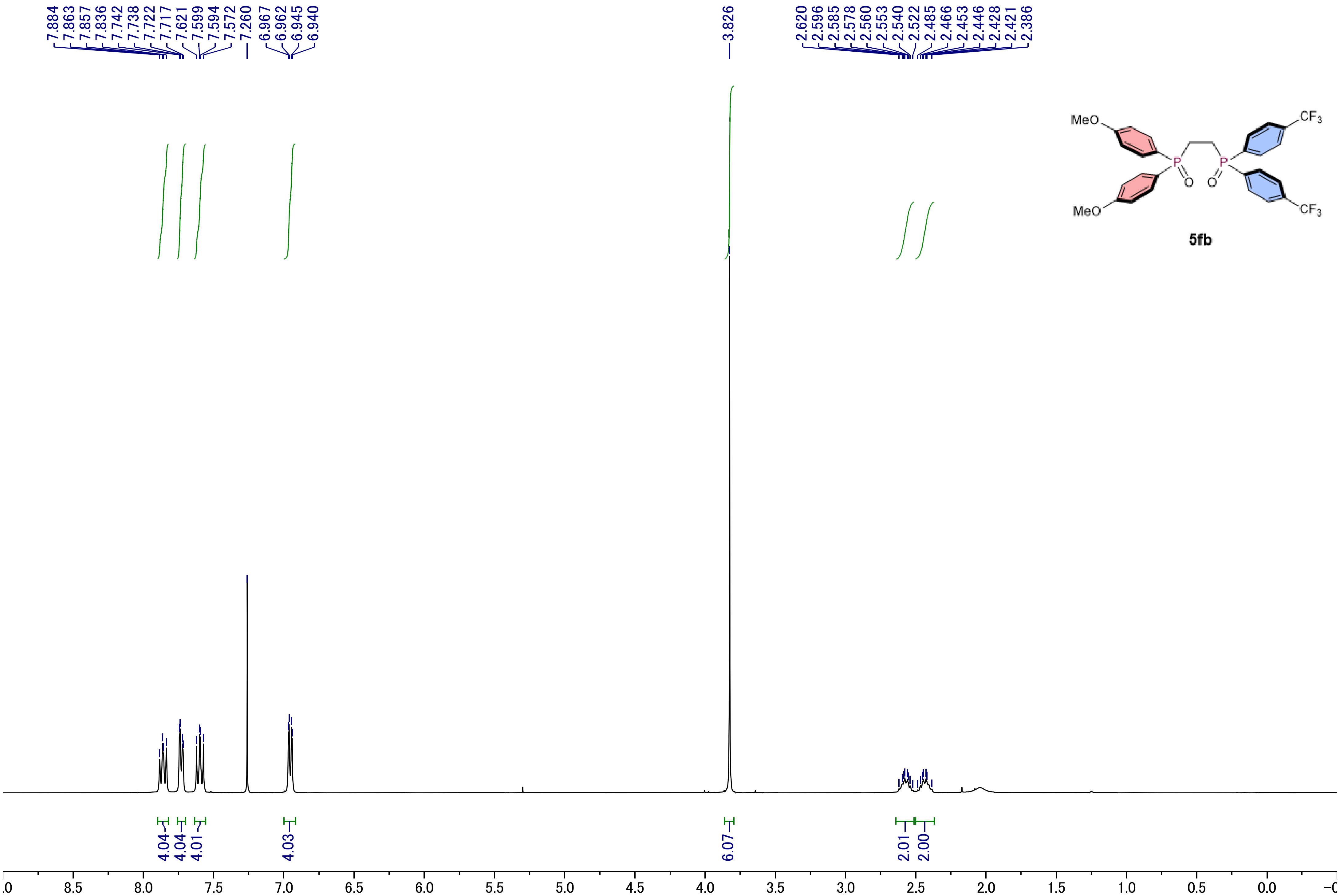

Supplementary Figure 133. ^{1}H NMR spectrum (400 MHz, $CDCl_3$) of **5fb**

CDCl$_3$, 100 MHz

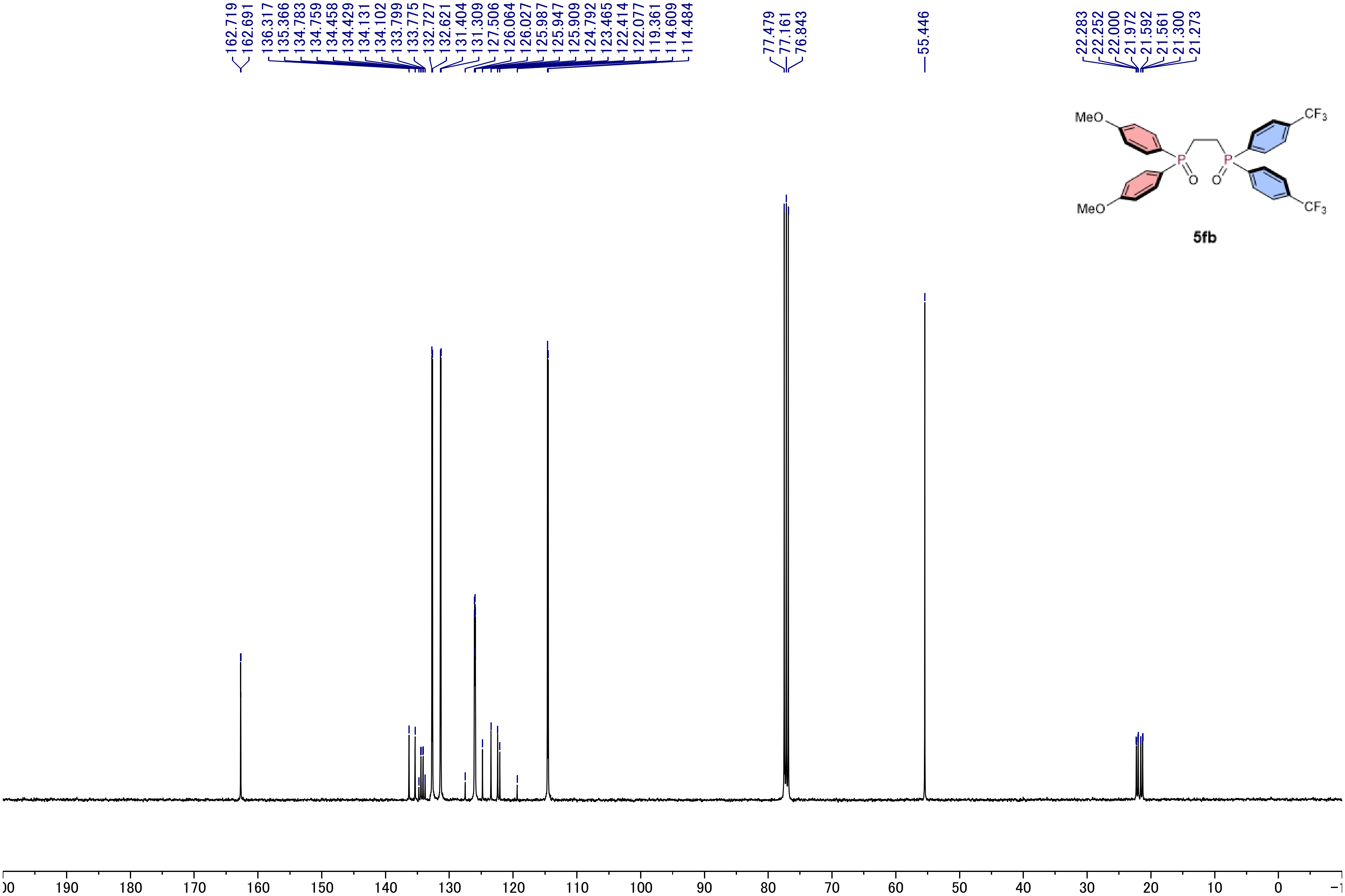

Supplementary Figure 134. ^{13}C NMR spectrum (100 MHz, CDCl$_3$) of **5fb**

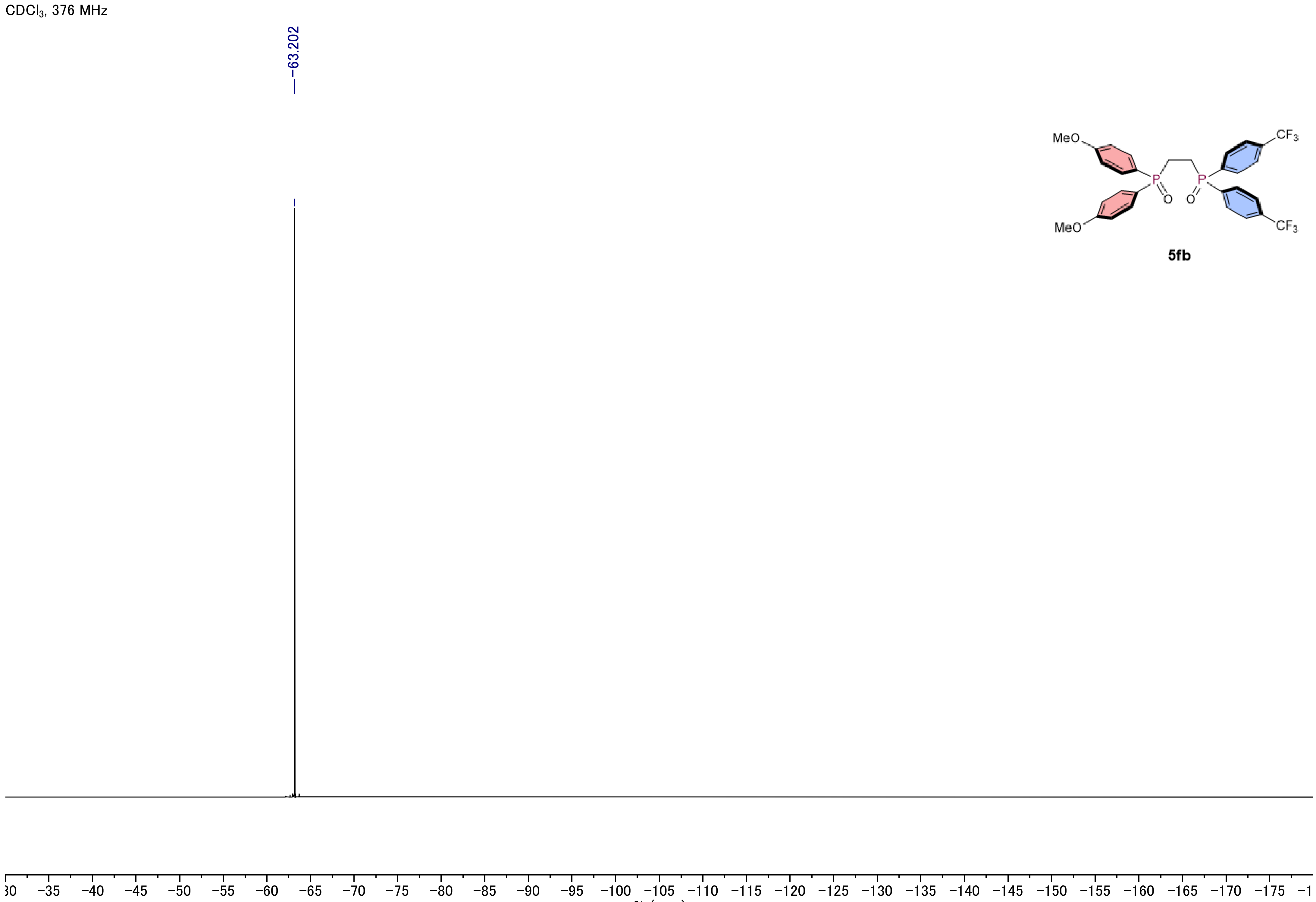

Supplementary Figure 135. ^{19}F NMR spectrum (376 MHz, $CDCl_3$) of **5fb**

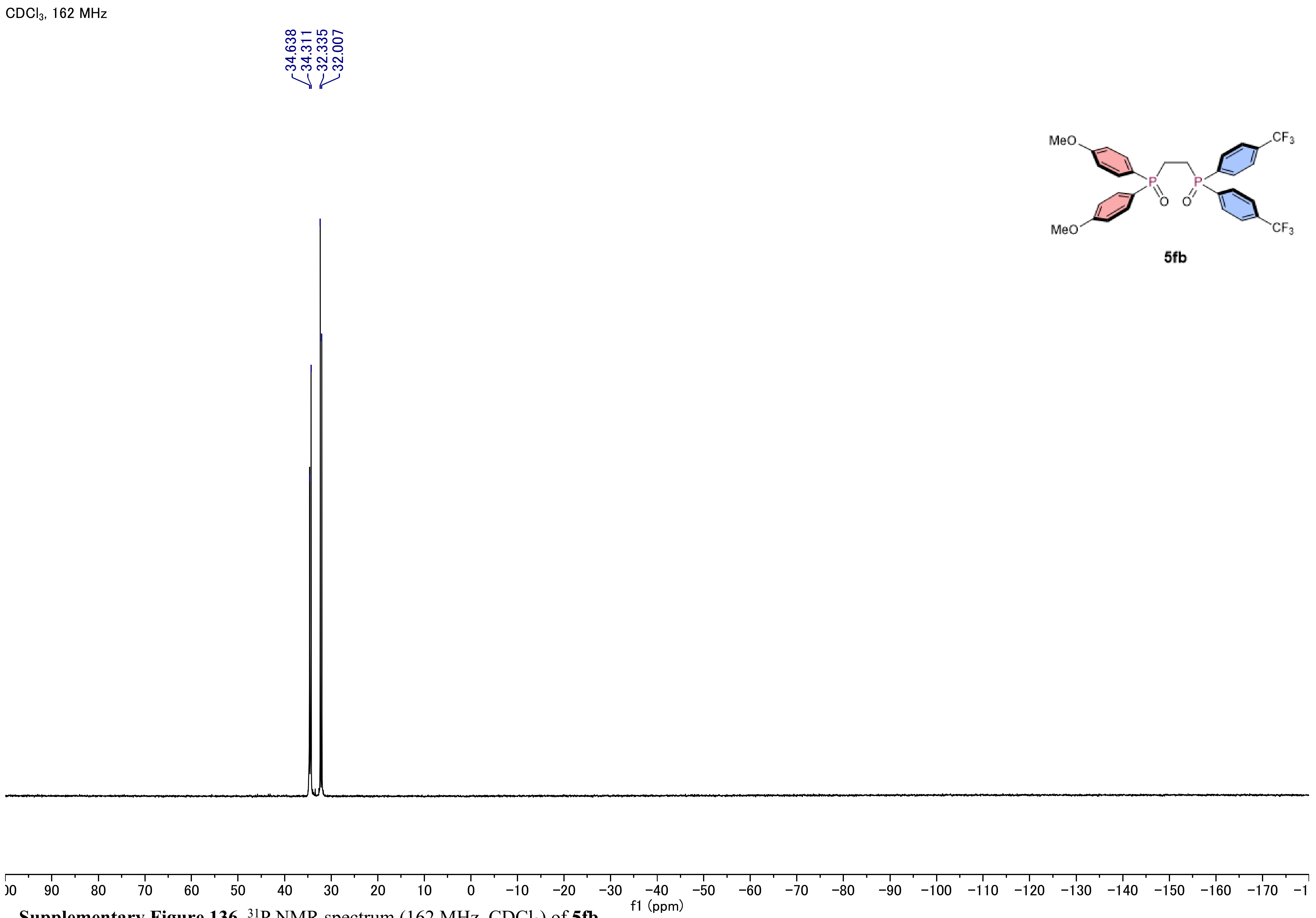

Supplementary Figure 136. ^{31}P NMR spectrum (162 MHz, $CDCl_3$) of **5fb**

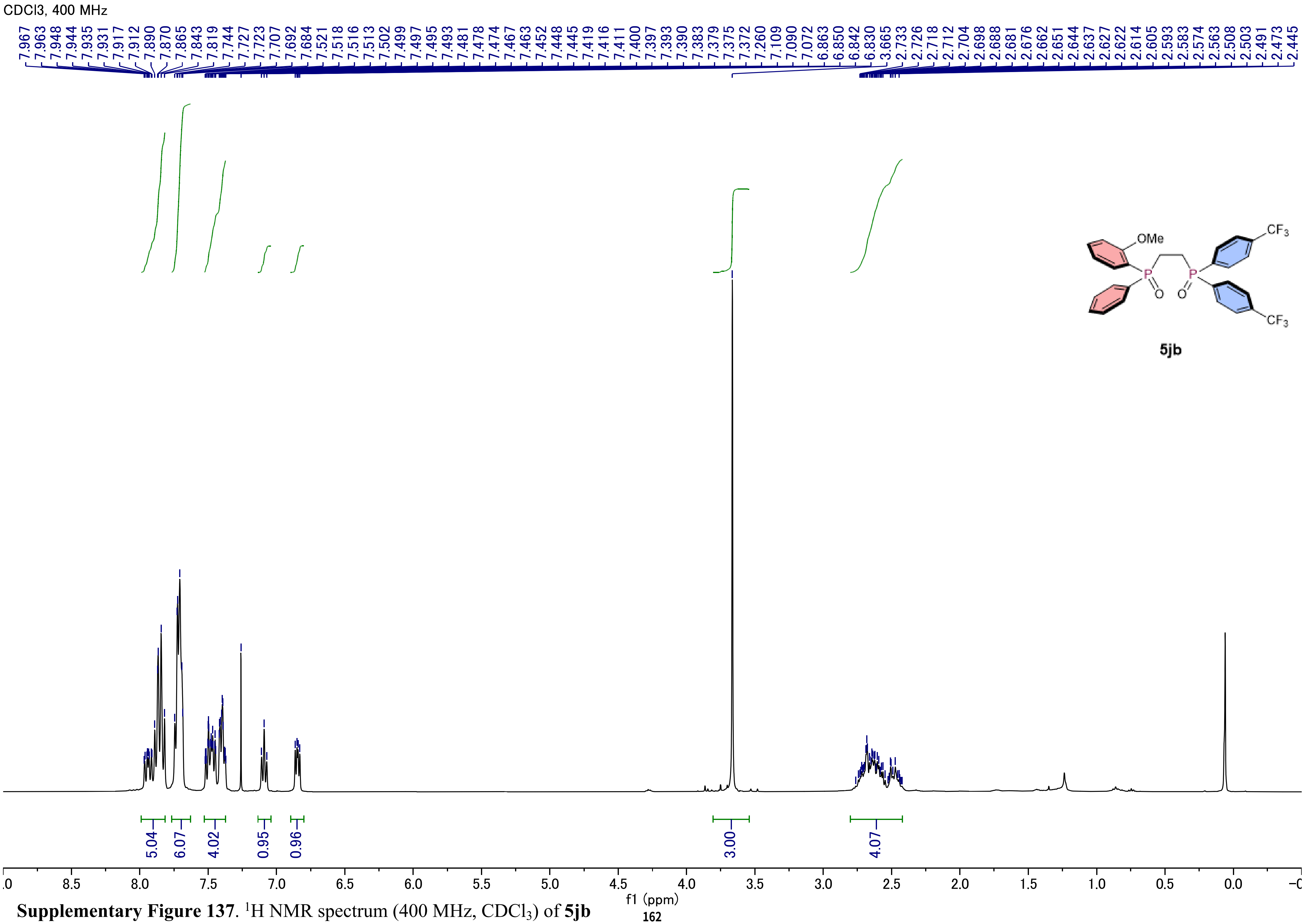

Supplementary Figure 137. ^{1}H NMR spectrum (400 MHz, $CDCl_3$) of **5jb**

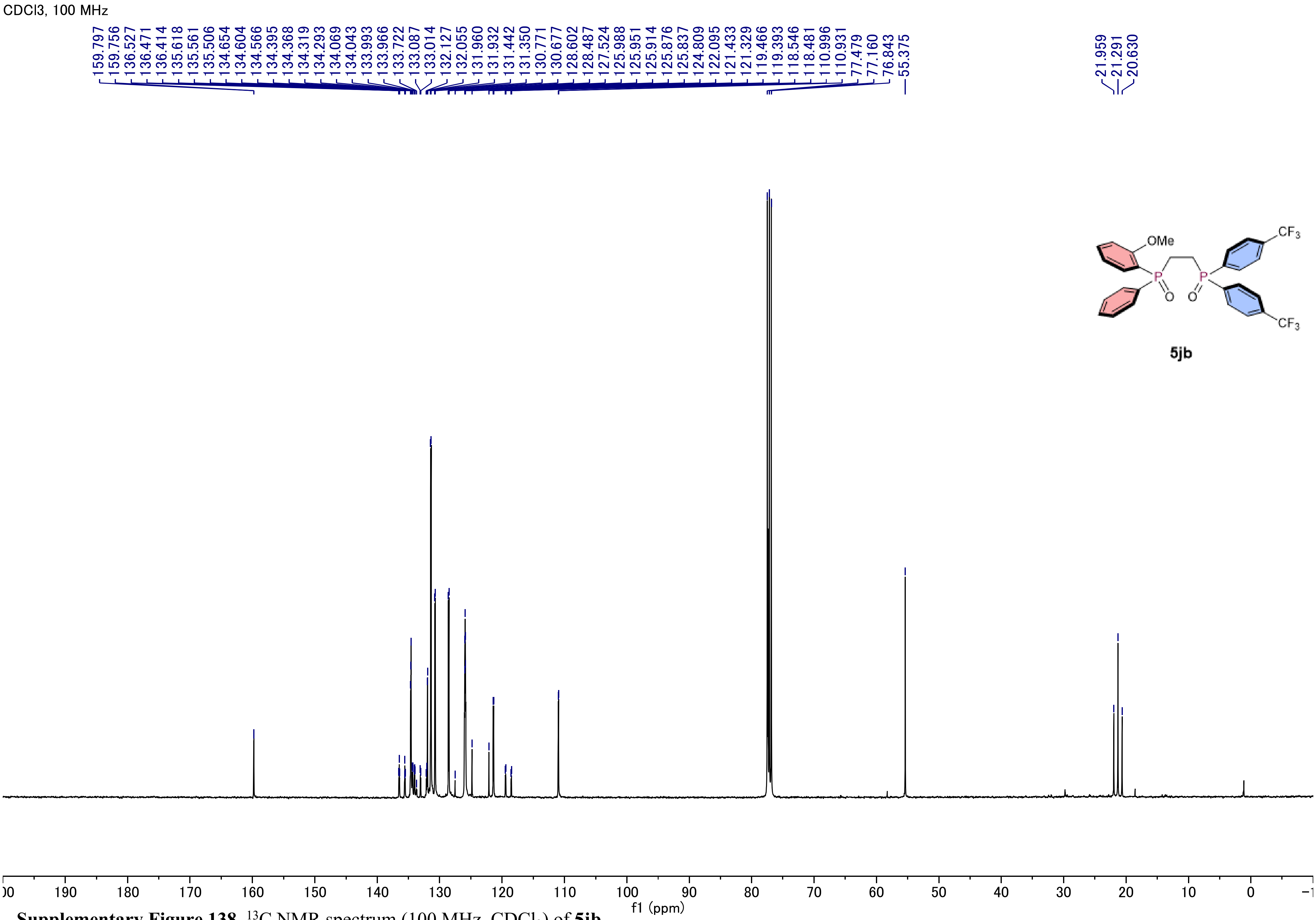

Supplementary Figure 138. ^{13}C NMR spectrum (100 MHz, $CDCl_3$) of **5jb**

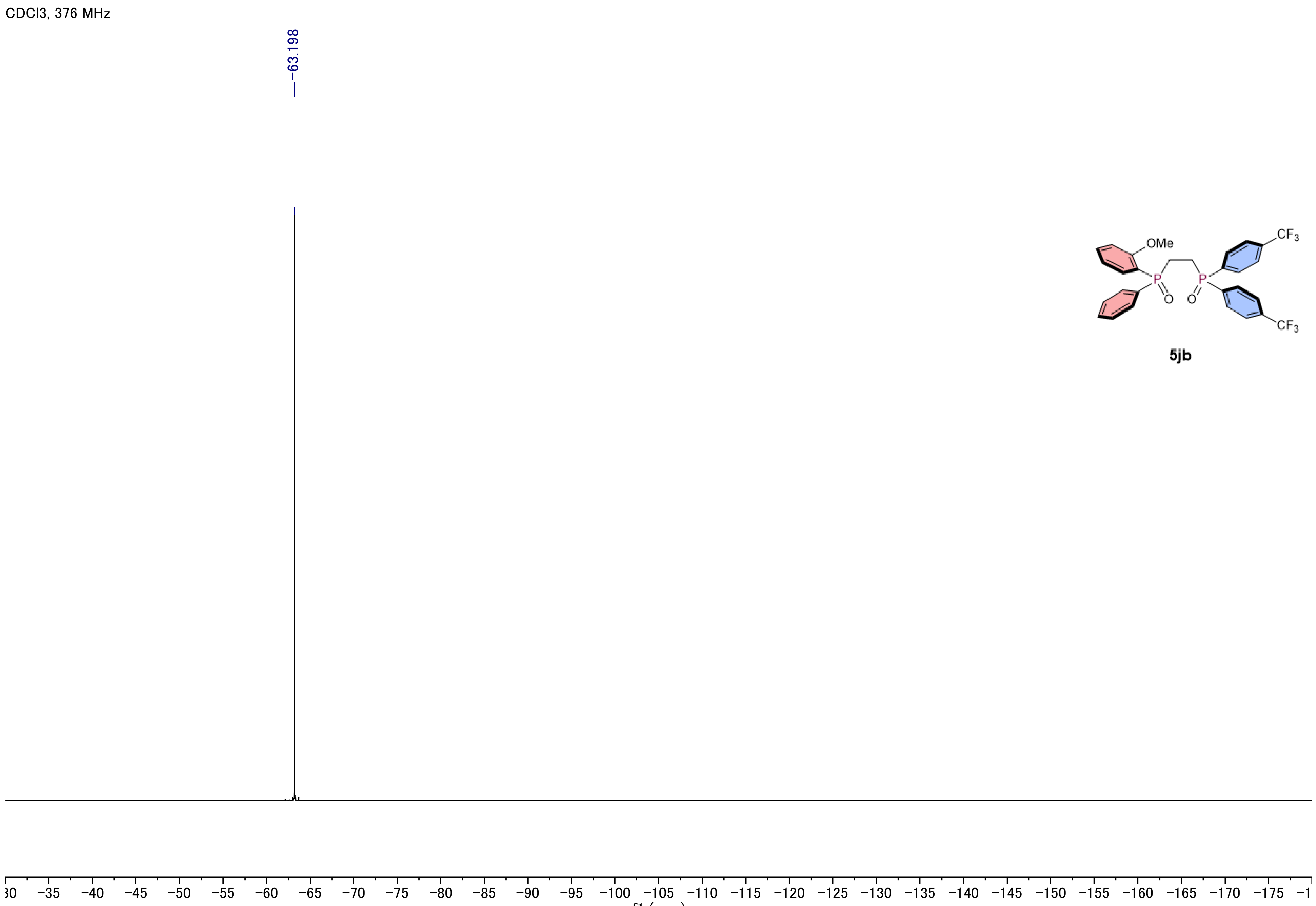

5jb

Supplementary Figure 139. ^{19}F NMR spectrum (376 MHz, $CDCl_3$) of **5jb**

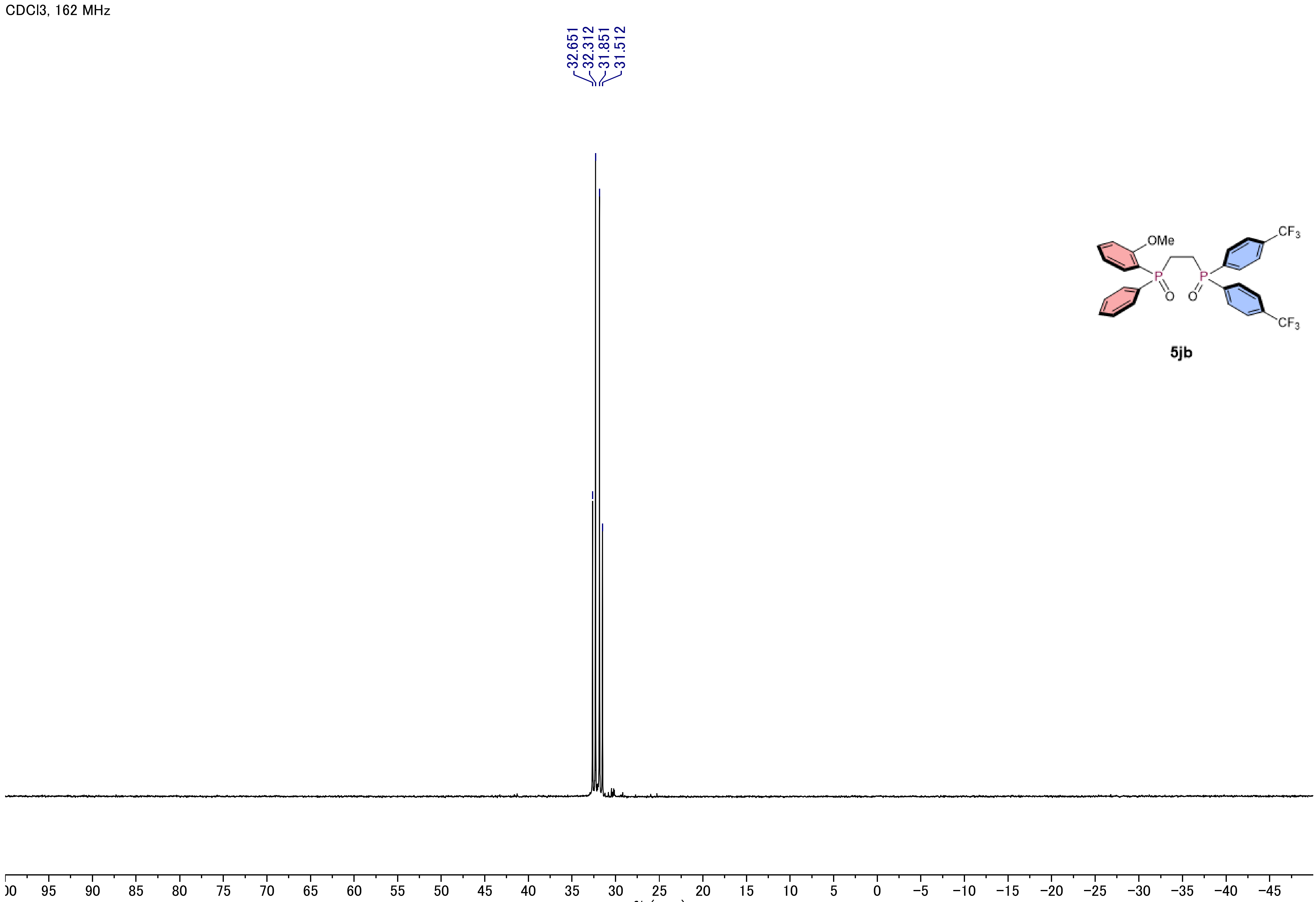

5jb

Supplementary Figure 140. ^{31}P NMR spectrum (162 MHz, $CDCl_3$) of **5jb**

CDCl3, 400 MHz

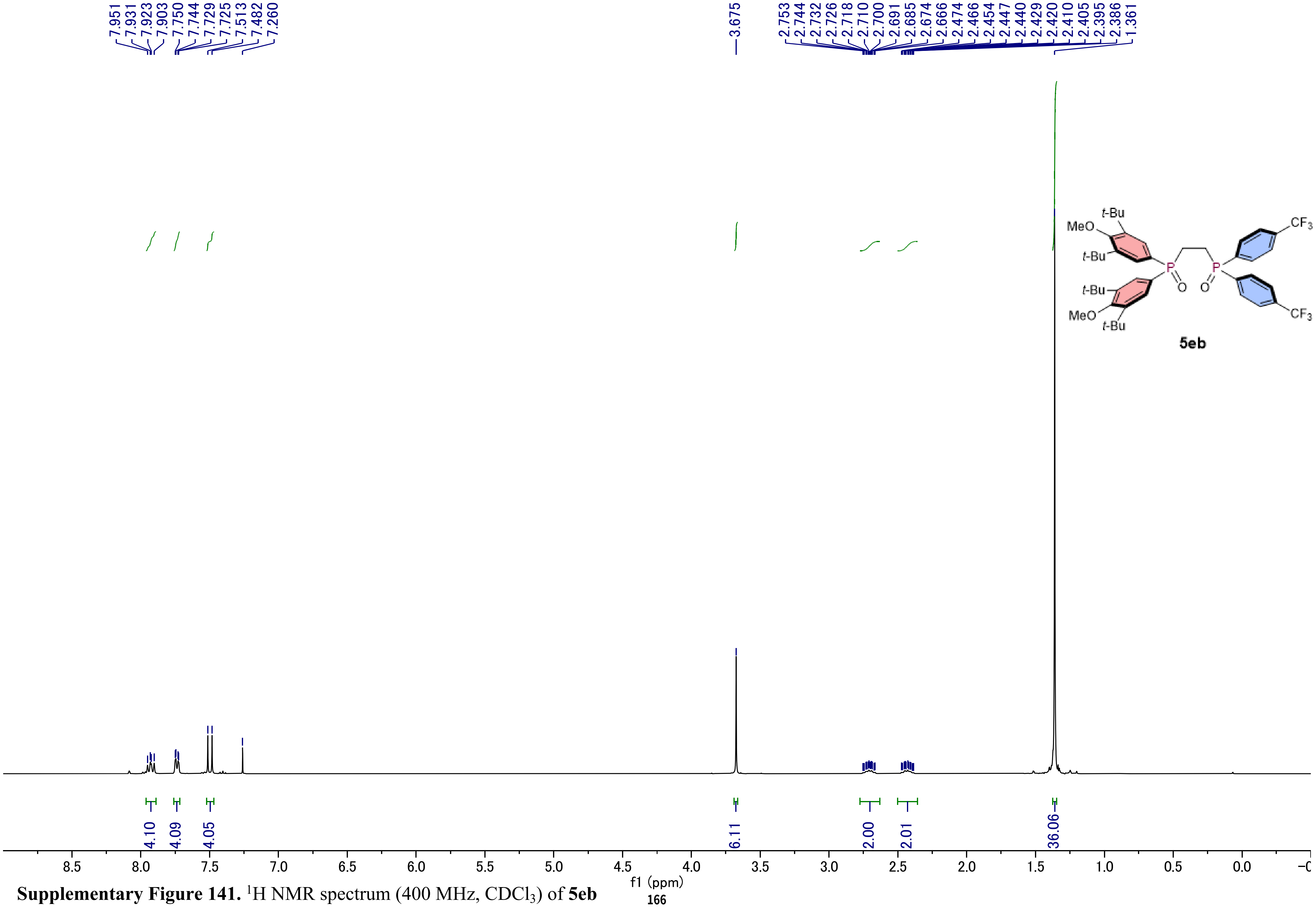

Supplementary Figure 141. ^{1}H NMR spectrum (400 MHz, $CDCl_3$) of **5eb**

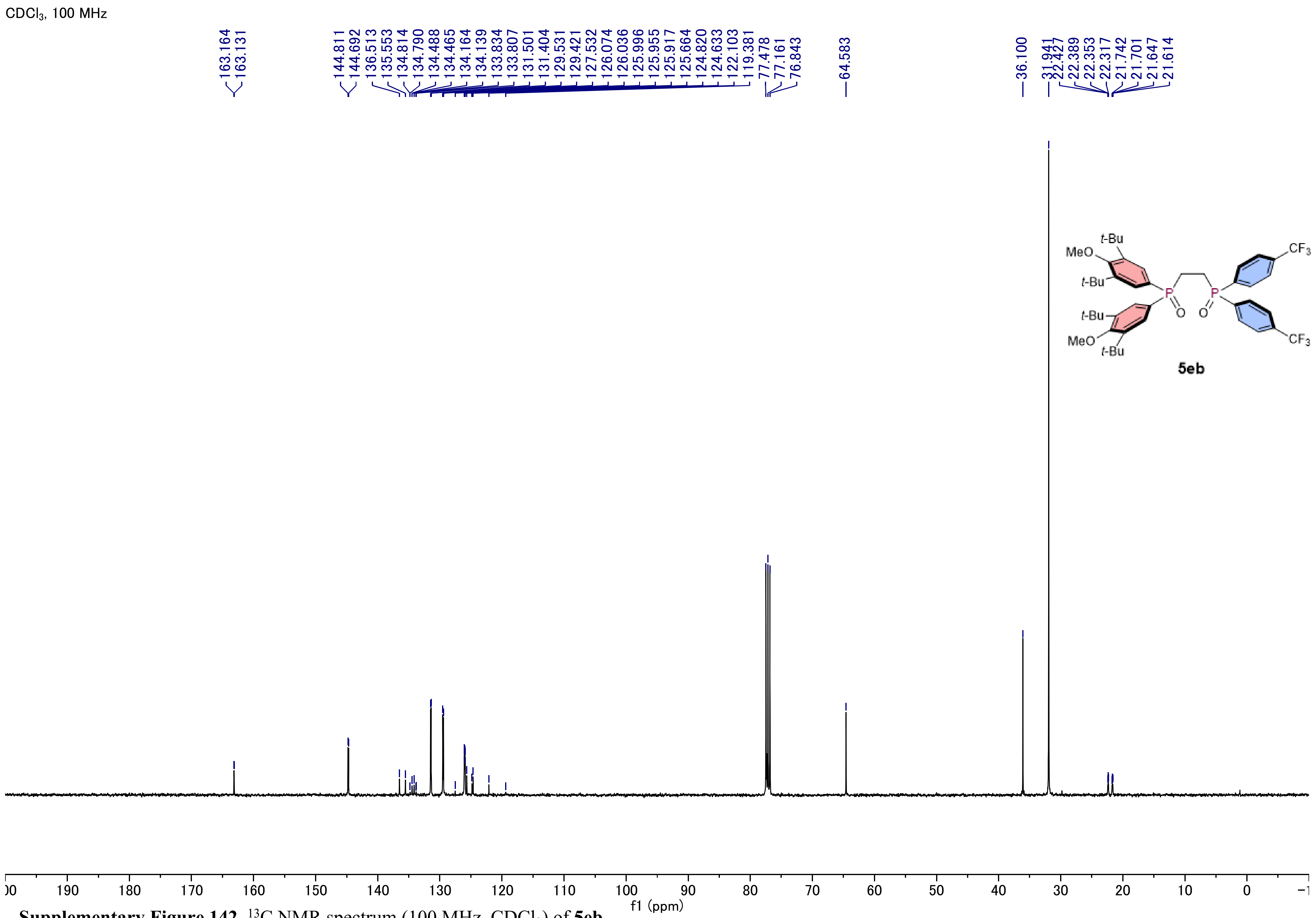

Supplementary Figure 142. ^{13}C NMR spectrum (100 MHz, CDCl$_3$) of **5eb**

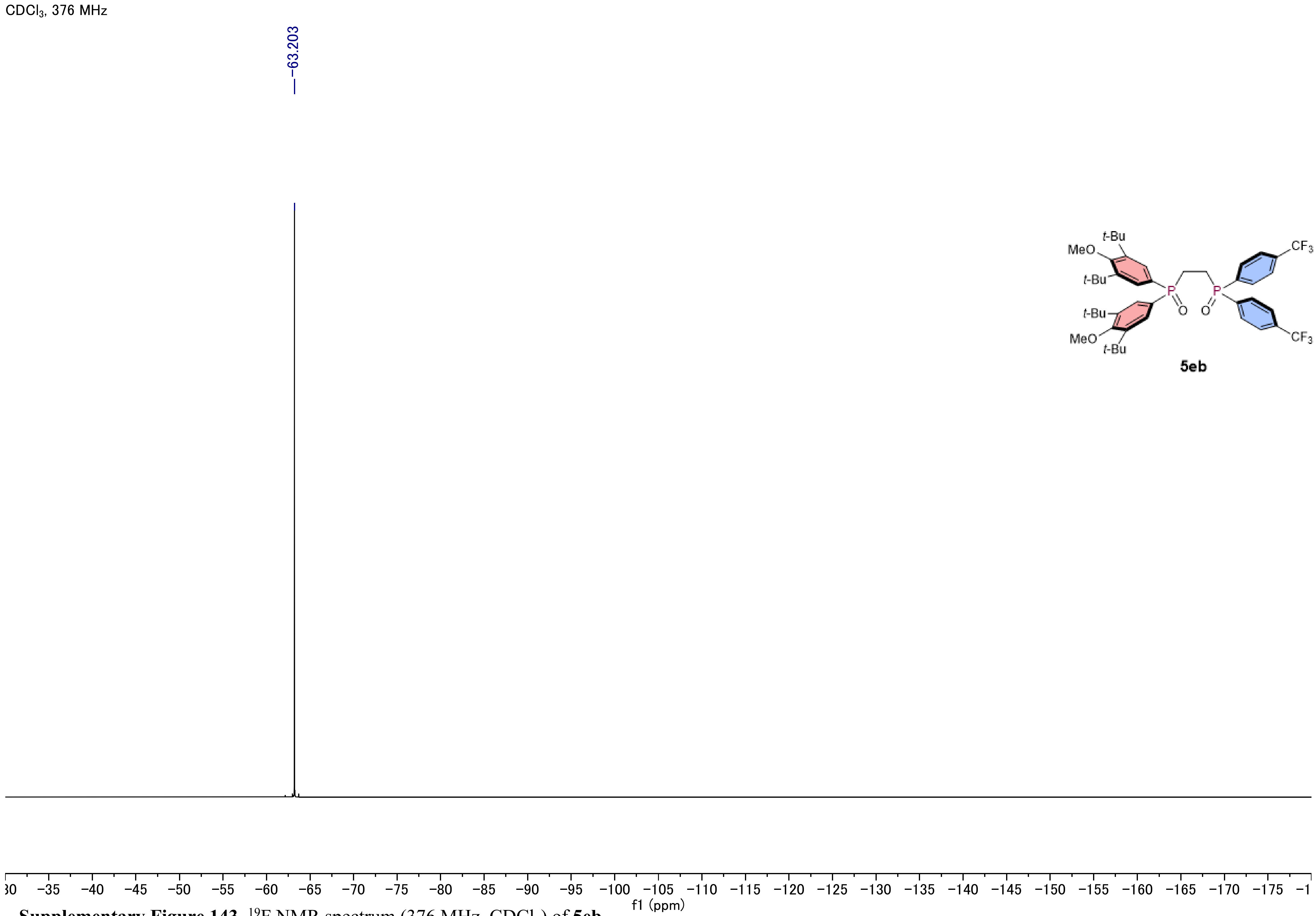

Supplementary Figure 143. ^{19}F NMR spectrum (376 MHz, $CDCl_3$) of **5eb**

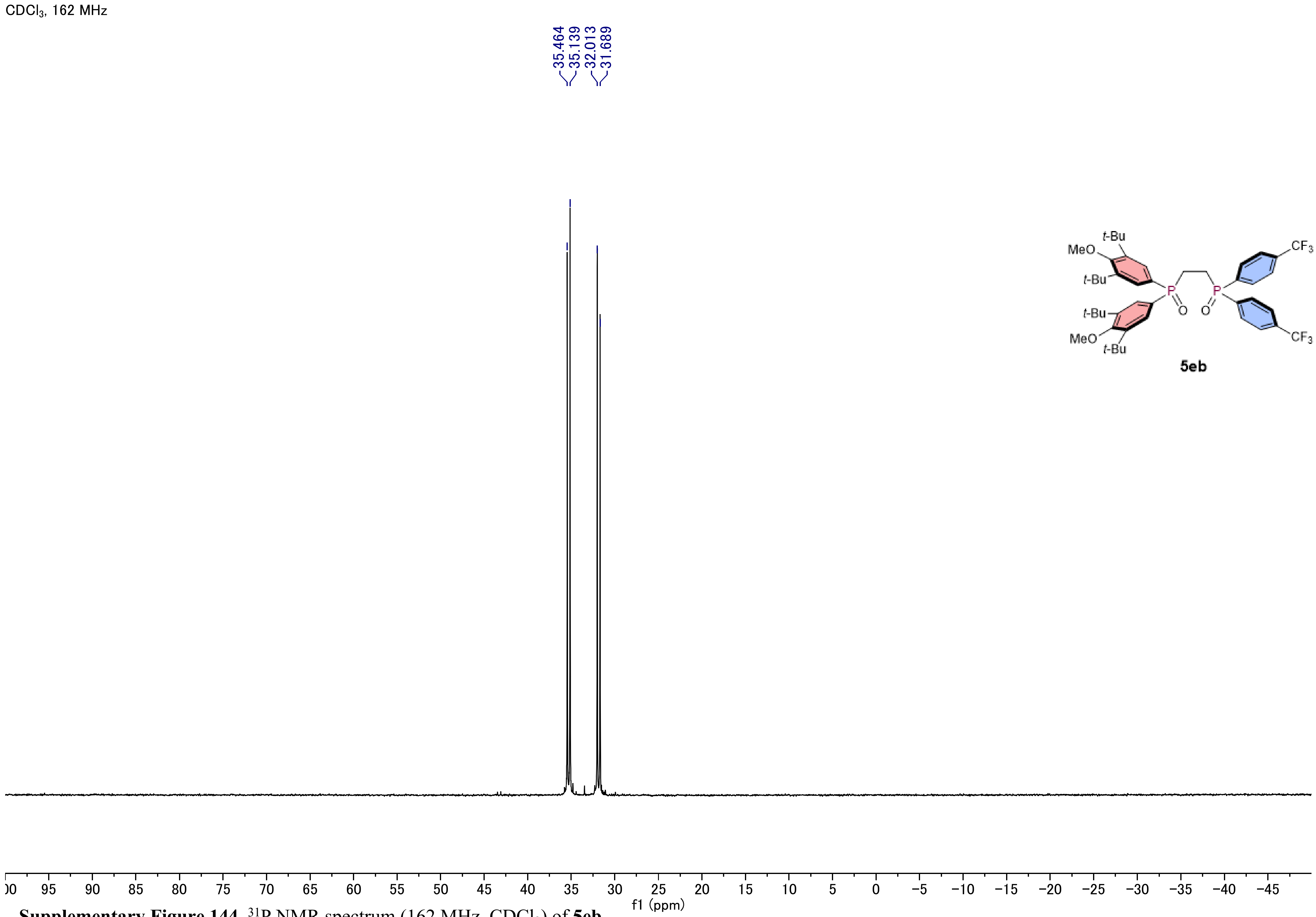

5eb

Supplementary Figure 144. ^{31}P NMR spectrum (162 MHz, $CDCl_3$) of **5eb**

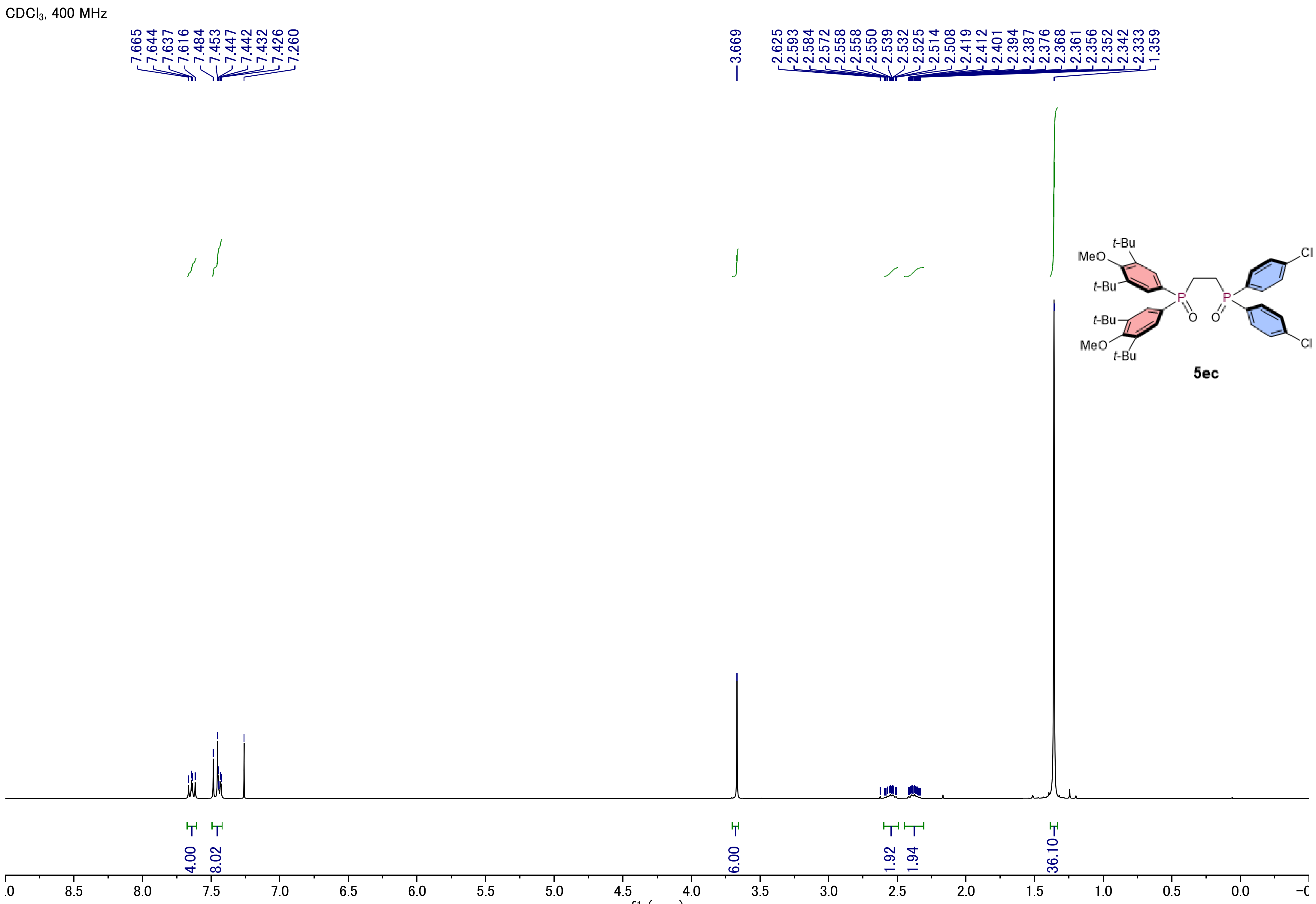

Supplementary Figure 145 ^{1}H NMR spectrum (400 MHz, $CDCl_3$) of **5ec**

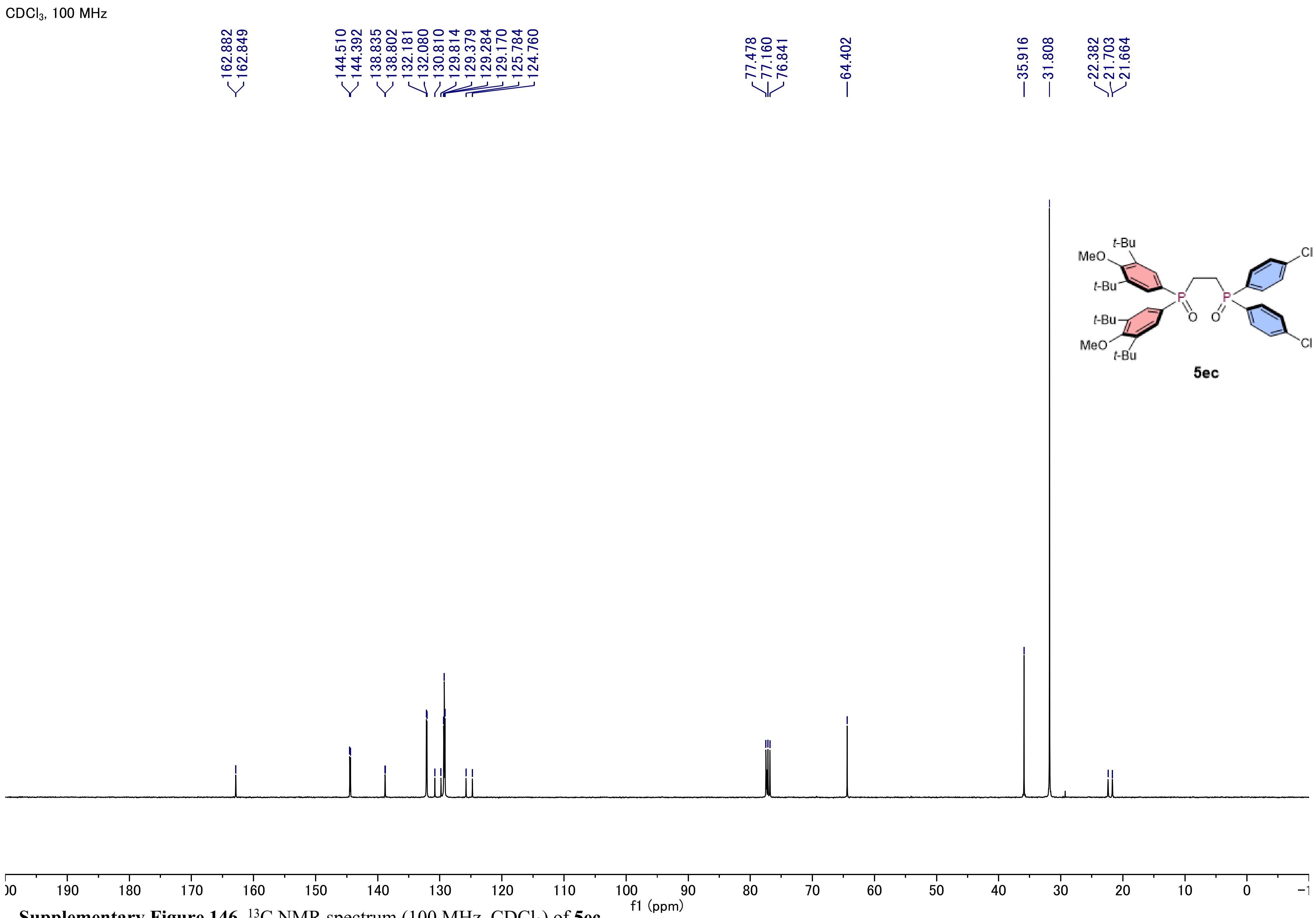

Supplementary Figure 146. ^{13}C NMR spectrum (100 MHz, $CDCl_3$) of **5ec**

CDCl$_3$, 162 MHz

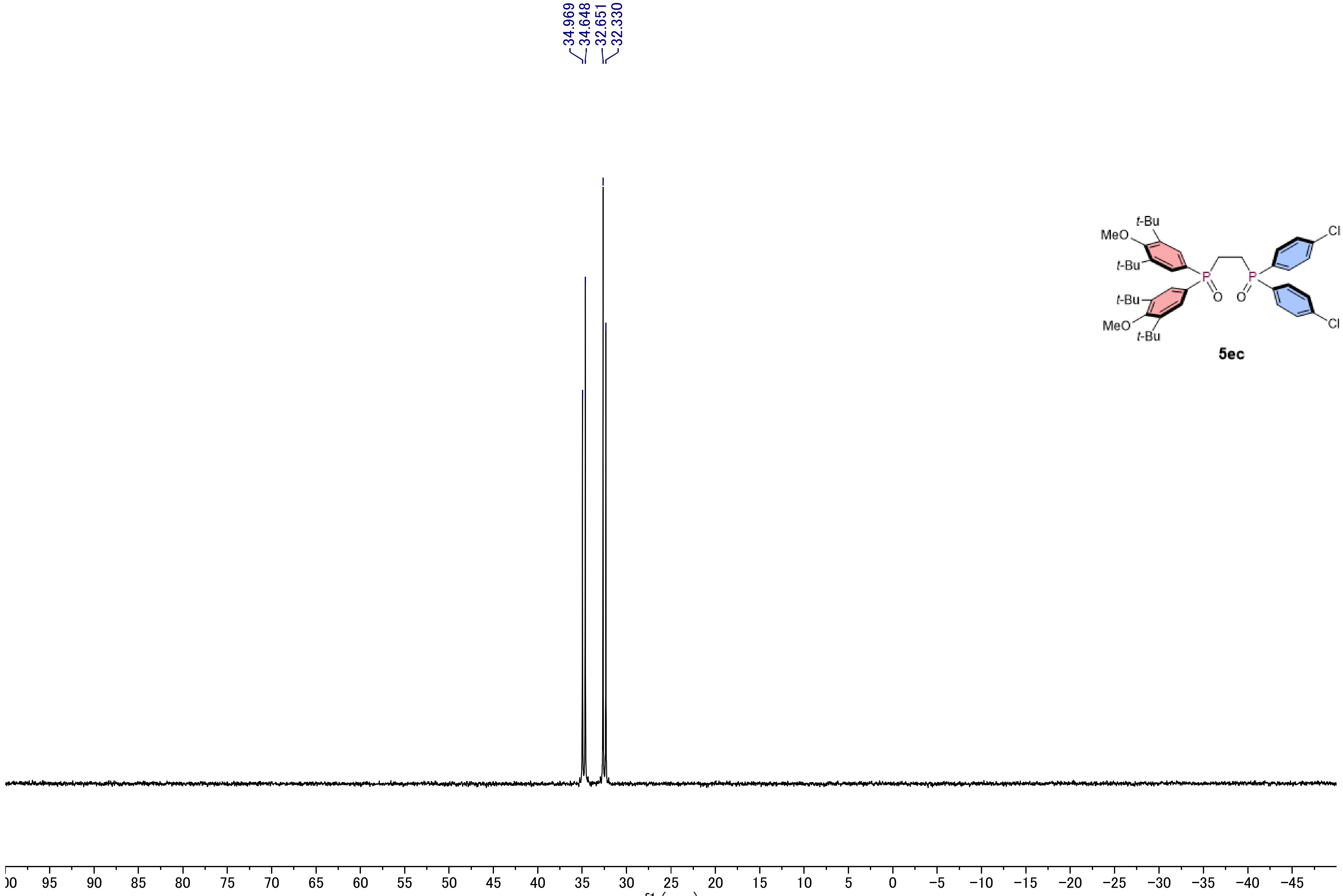

5ec

Supplementary Figure 147. ^{31}P NMR spectrum (162 MHz, CDCl$_3$) of **5ec**

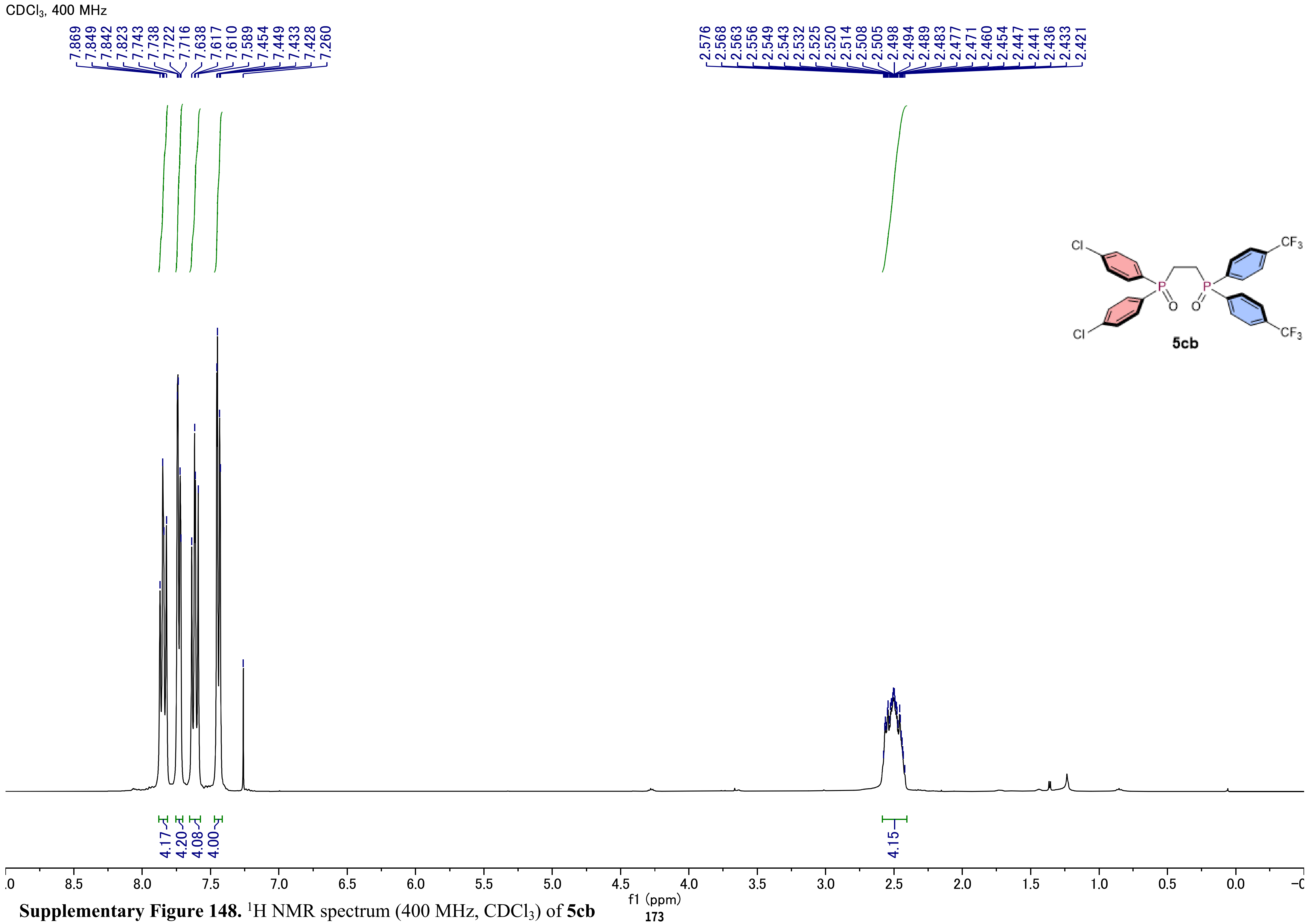

Supplementary Figure 148. ^{1}H NMR spectrum (400 MHz, $CDCl_3$) of **5cb**

CDCl$_3$, 100 MHz

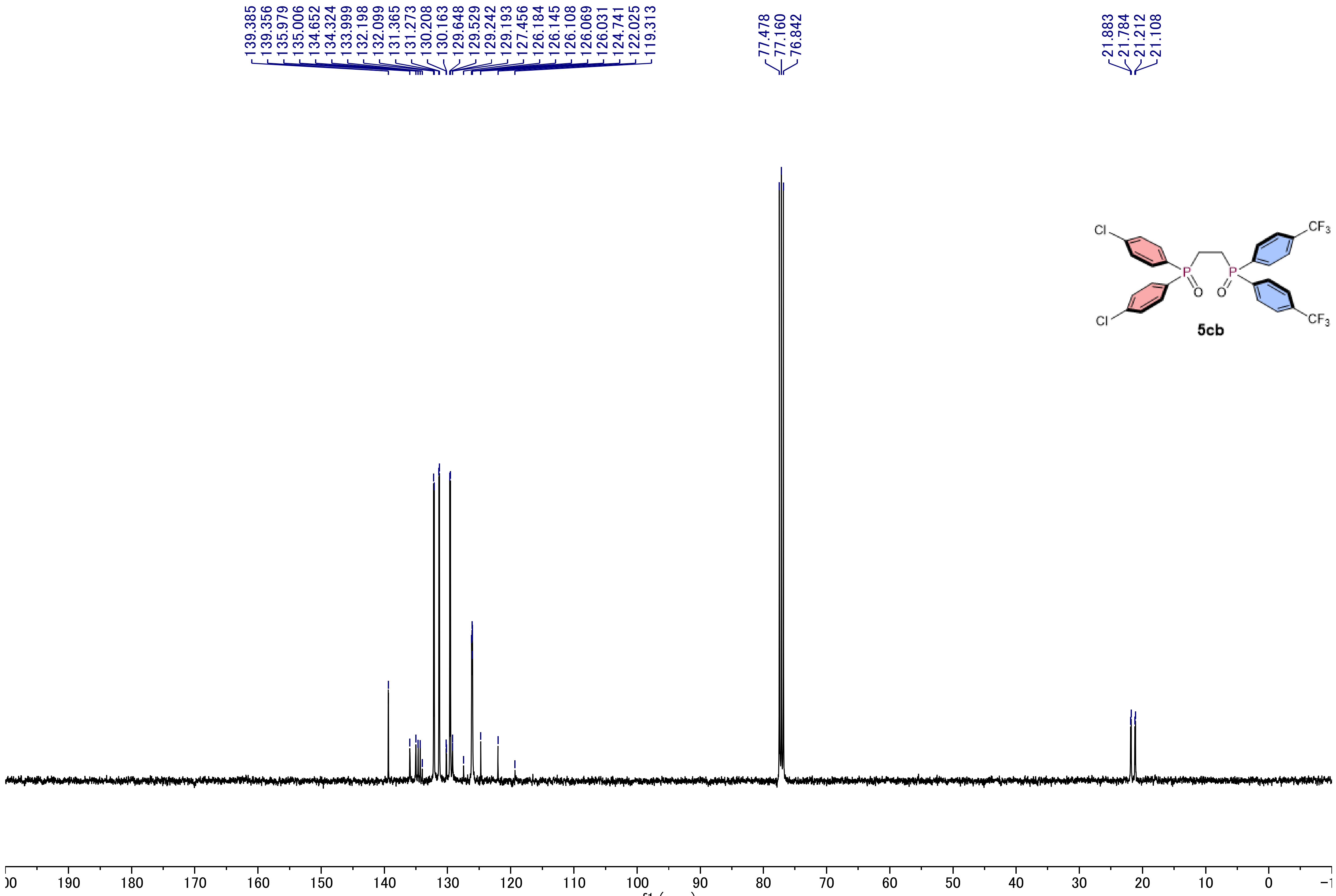

Supplementary Figure 149. ^{13}C NMR spectrum (100 MHz, CDCl$_3$) of **5cb**

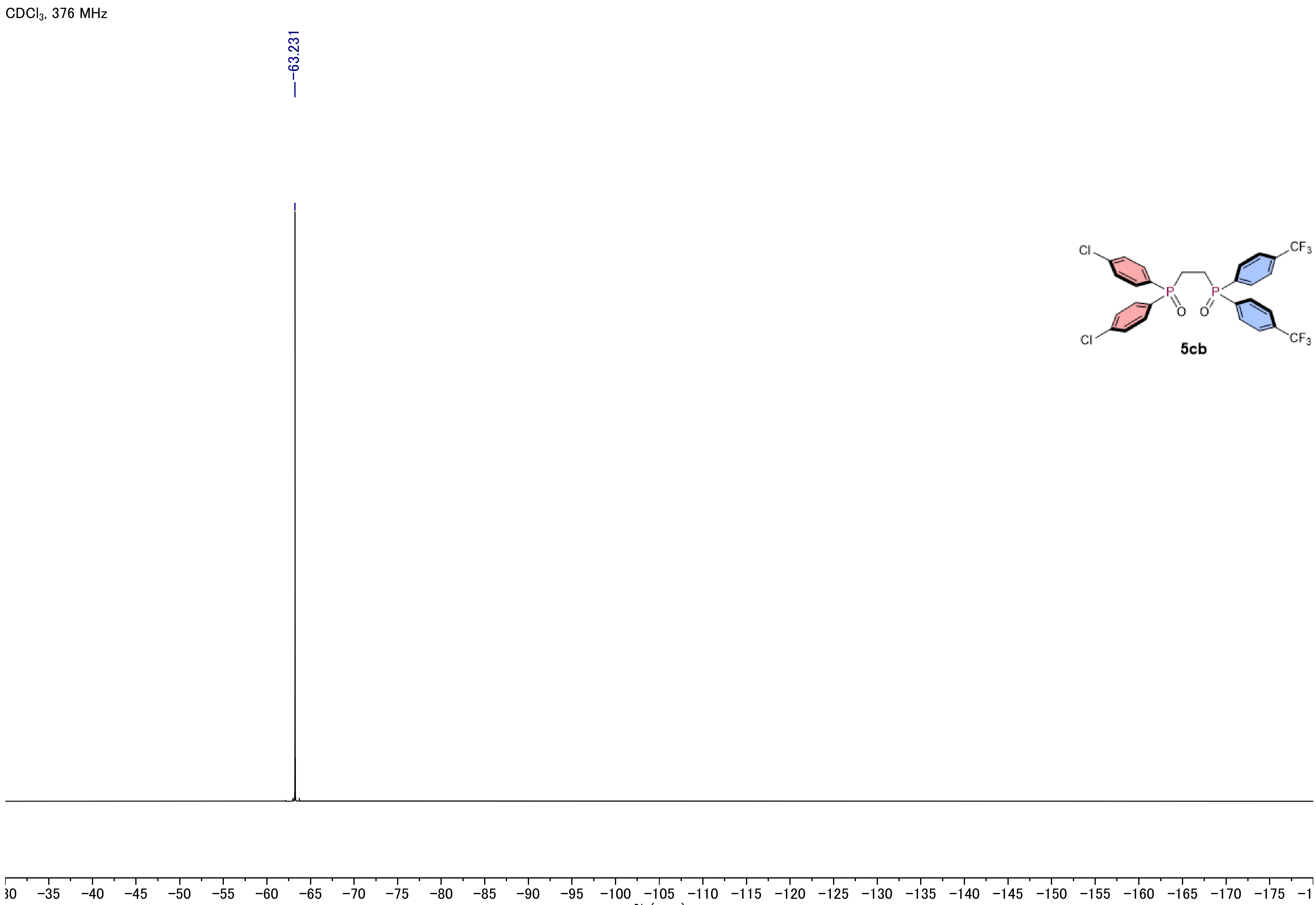

Supplementary Figure 150. ^{19}F NMR spectrum (376 MHz, $CDCl_3$) of **5cb**

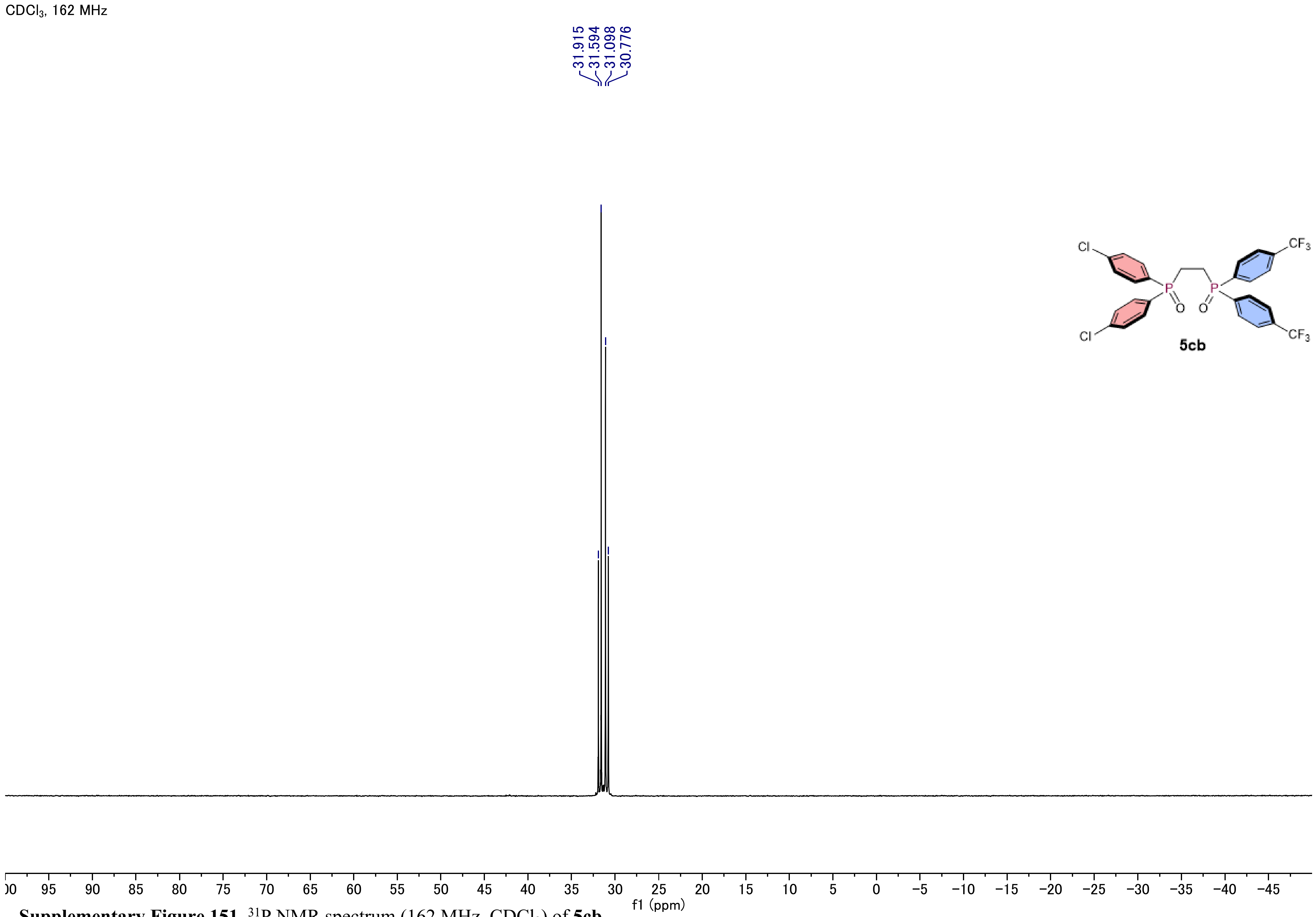

Supplementary Figure 151. ^{31}P NMR spectrum (162 MHz, $CDCl_3$) of **5cb**

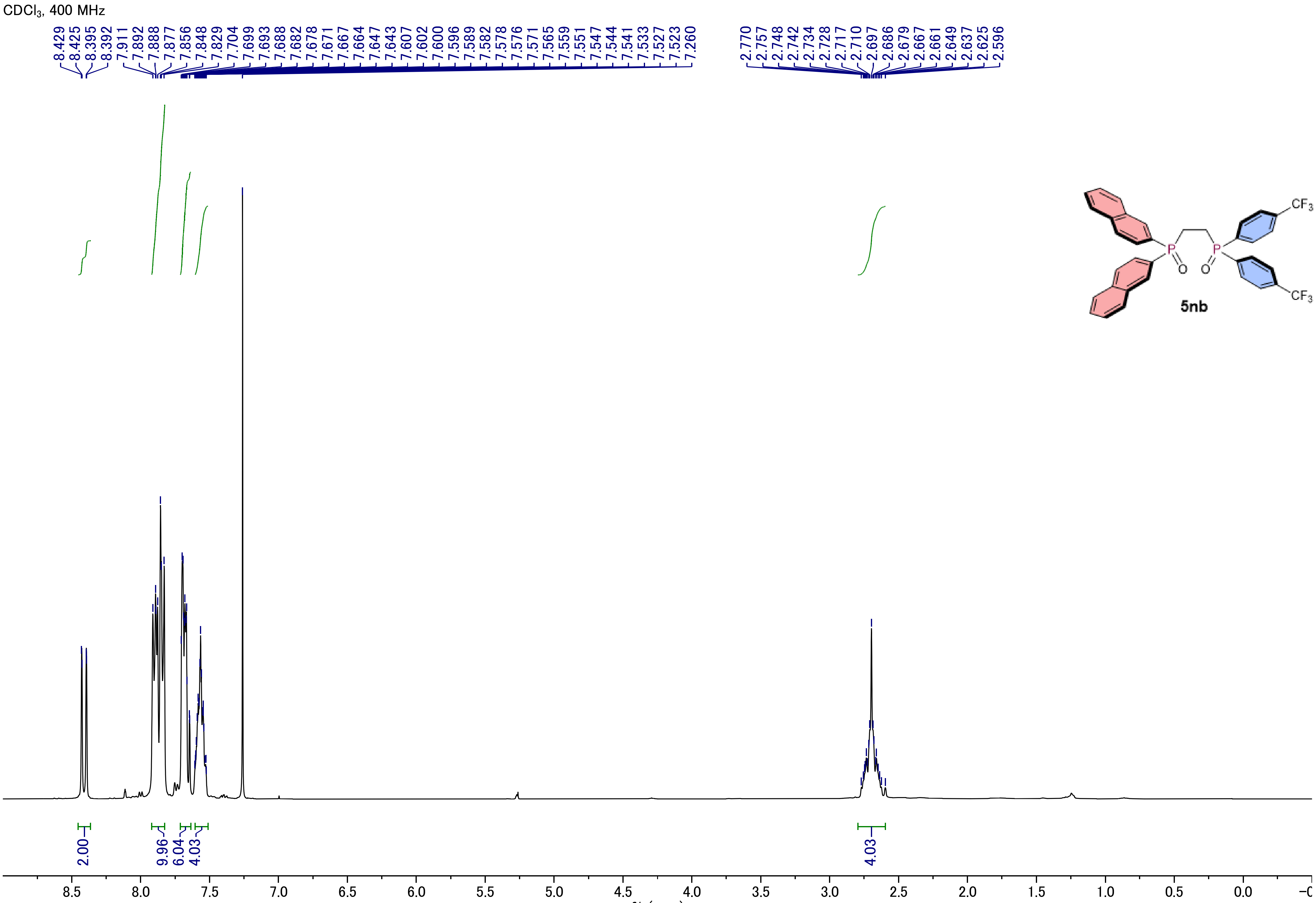

Supplementary Figure 152. ^{1}H NMR spectrum (400 MHz, $CDCl_3$) of **5nb**

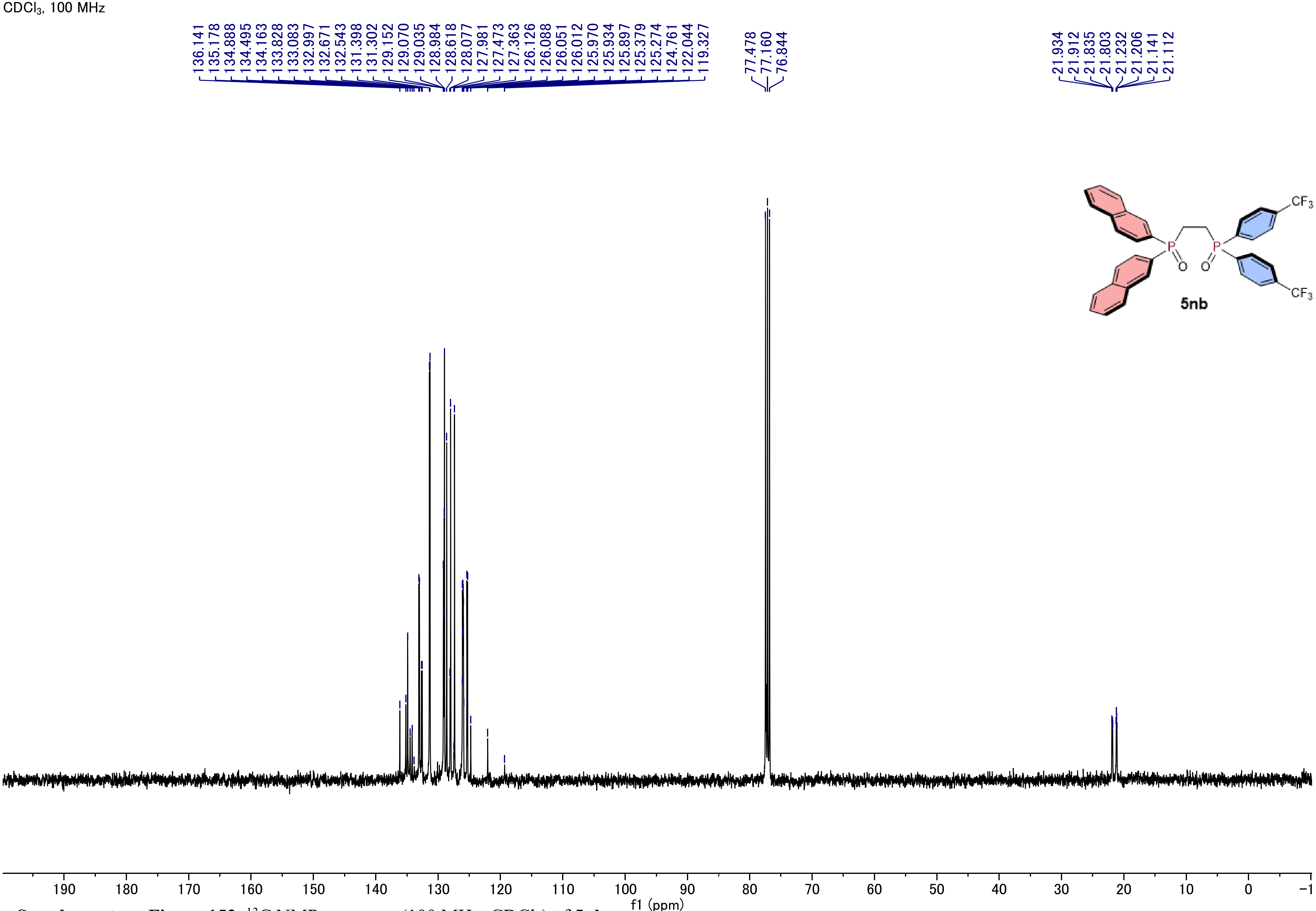

Supplementary Figure 153. ^{13}C NMR spectrum (100 MHz, CDCl$_3$) of **5nb**

CDCl$_3$, 376 MHz

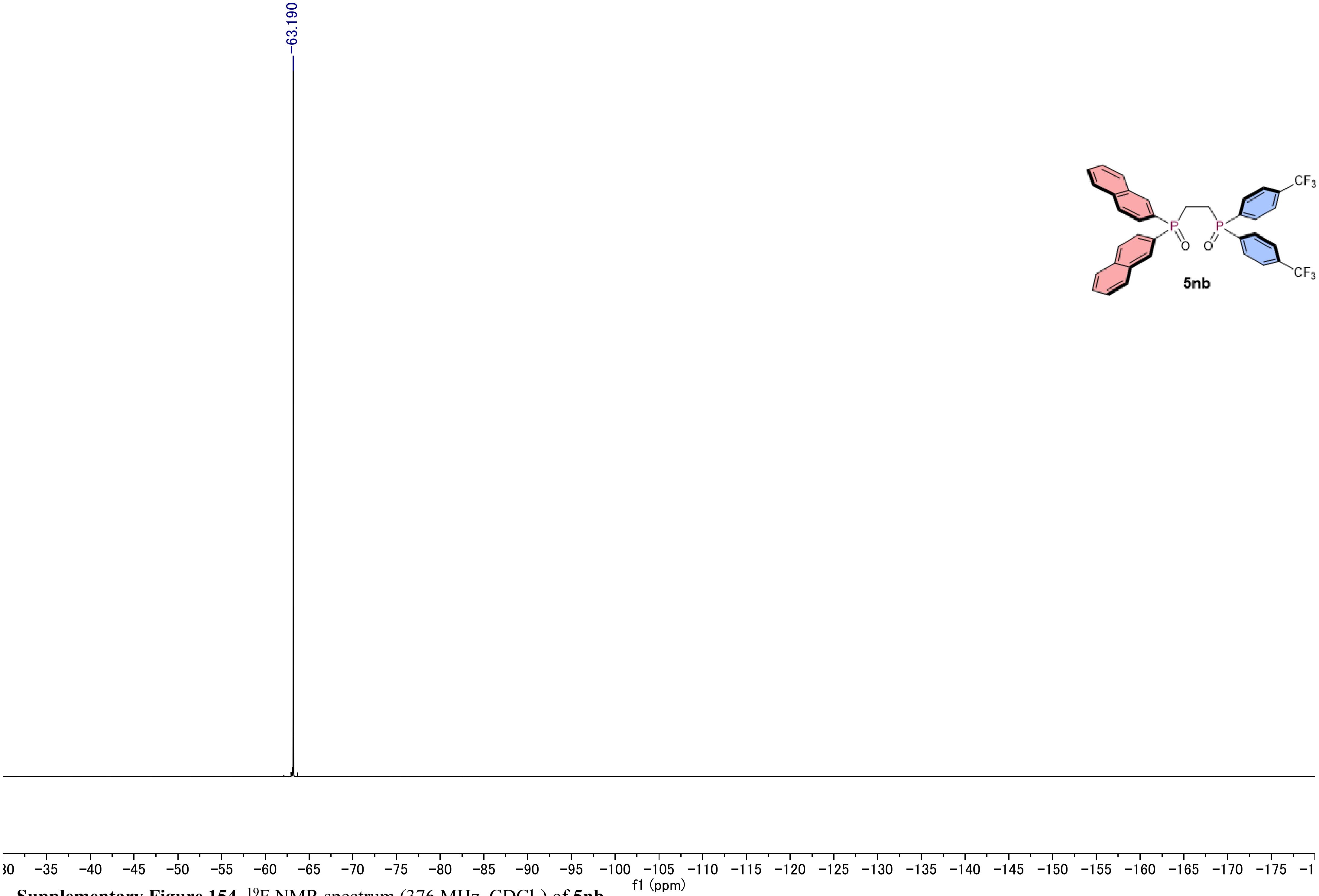

5nb

Supplementary Figure 154. ^{19}F NMR spectrum (376 MHz, CDCl$_3$) of **5nb**

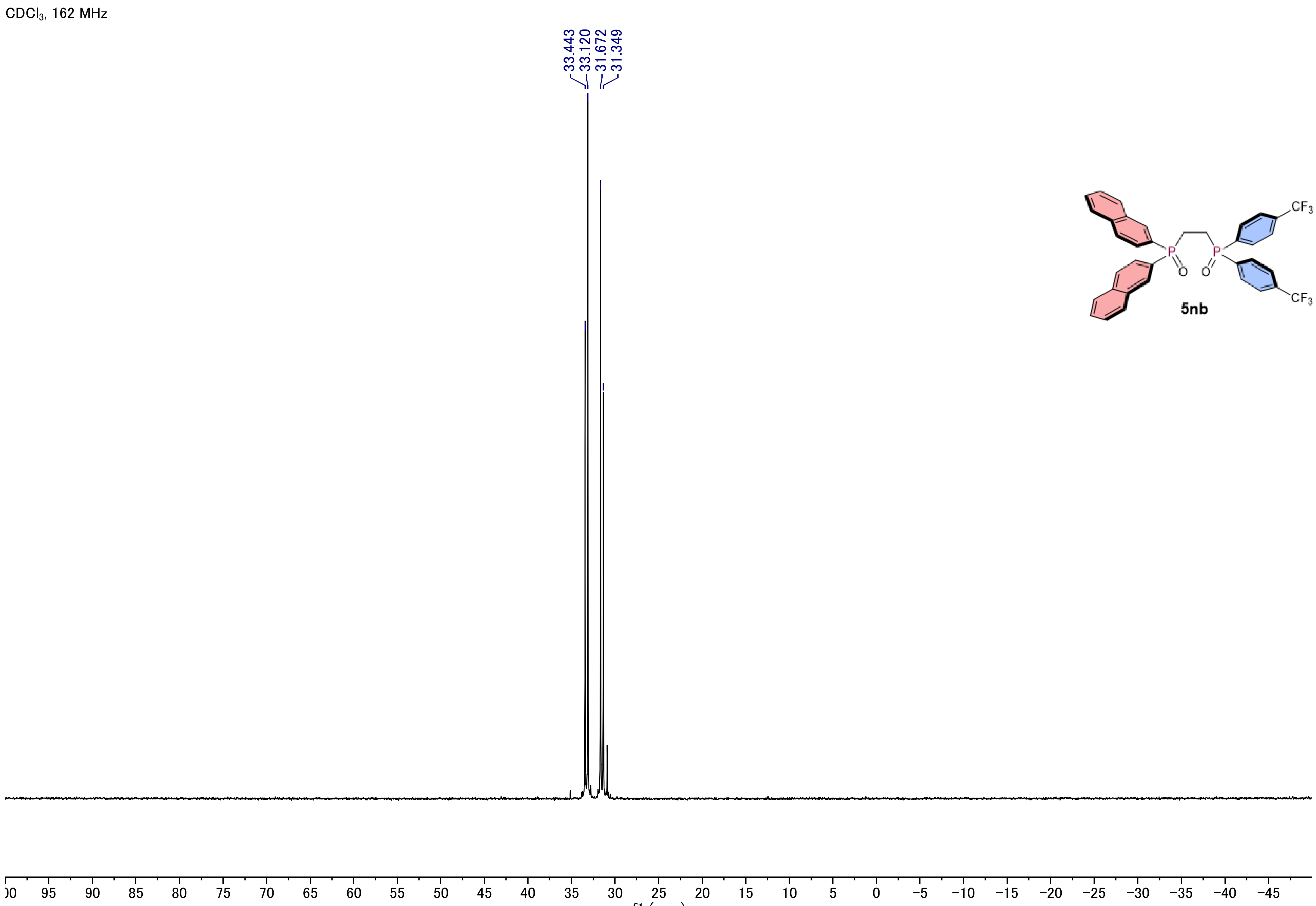

Supplementary Figure 155. ^{31}P NMR spectrum (162 MHz, $CDCl_3$) of **5nb**

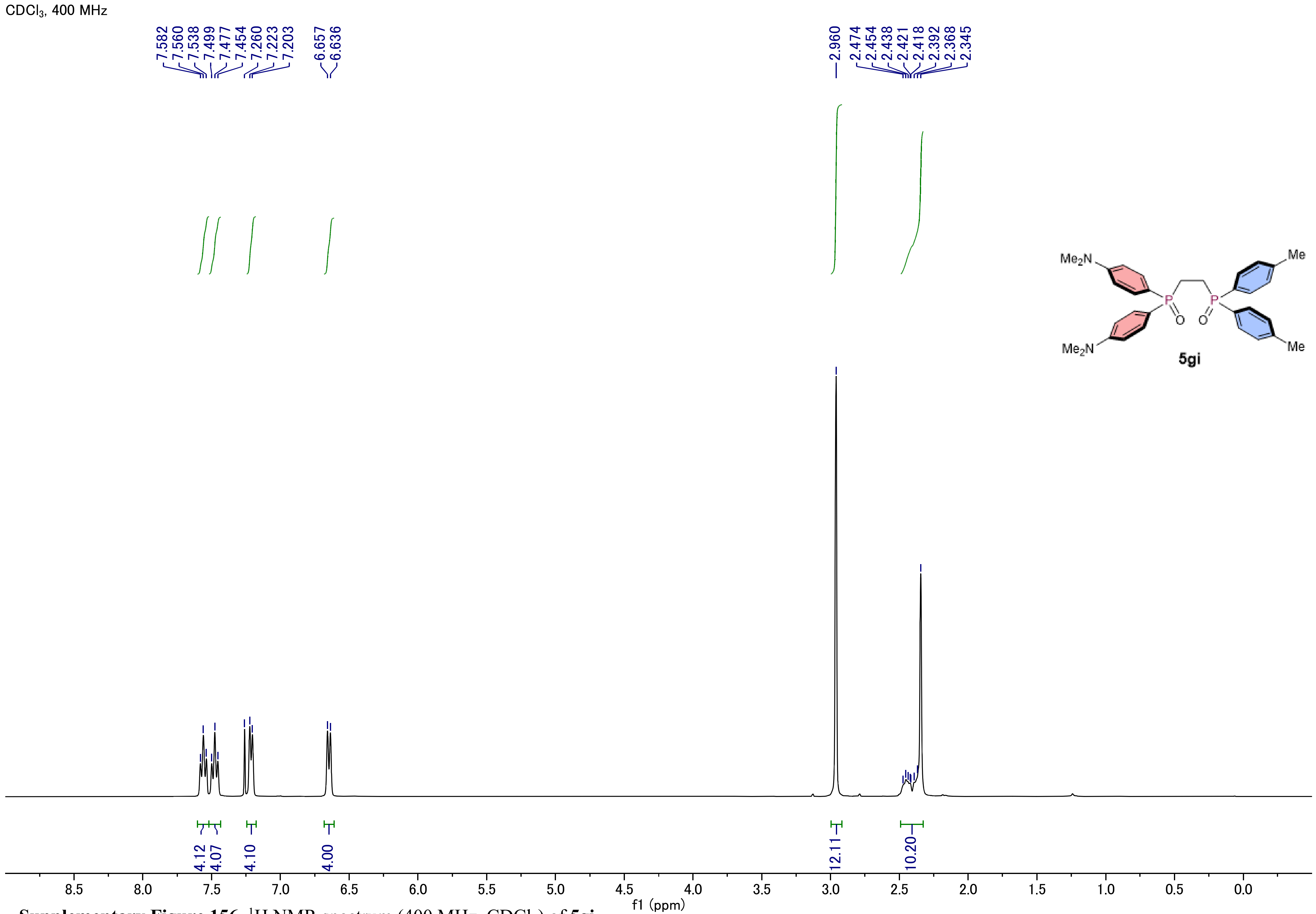

Supplementary Figure 156. ^{1}H NMR spectrum (400 MHz, $CDCl_3$) of **5gi**

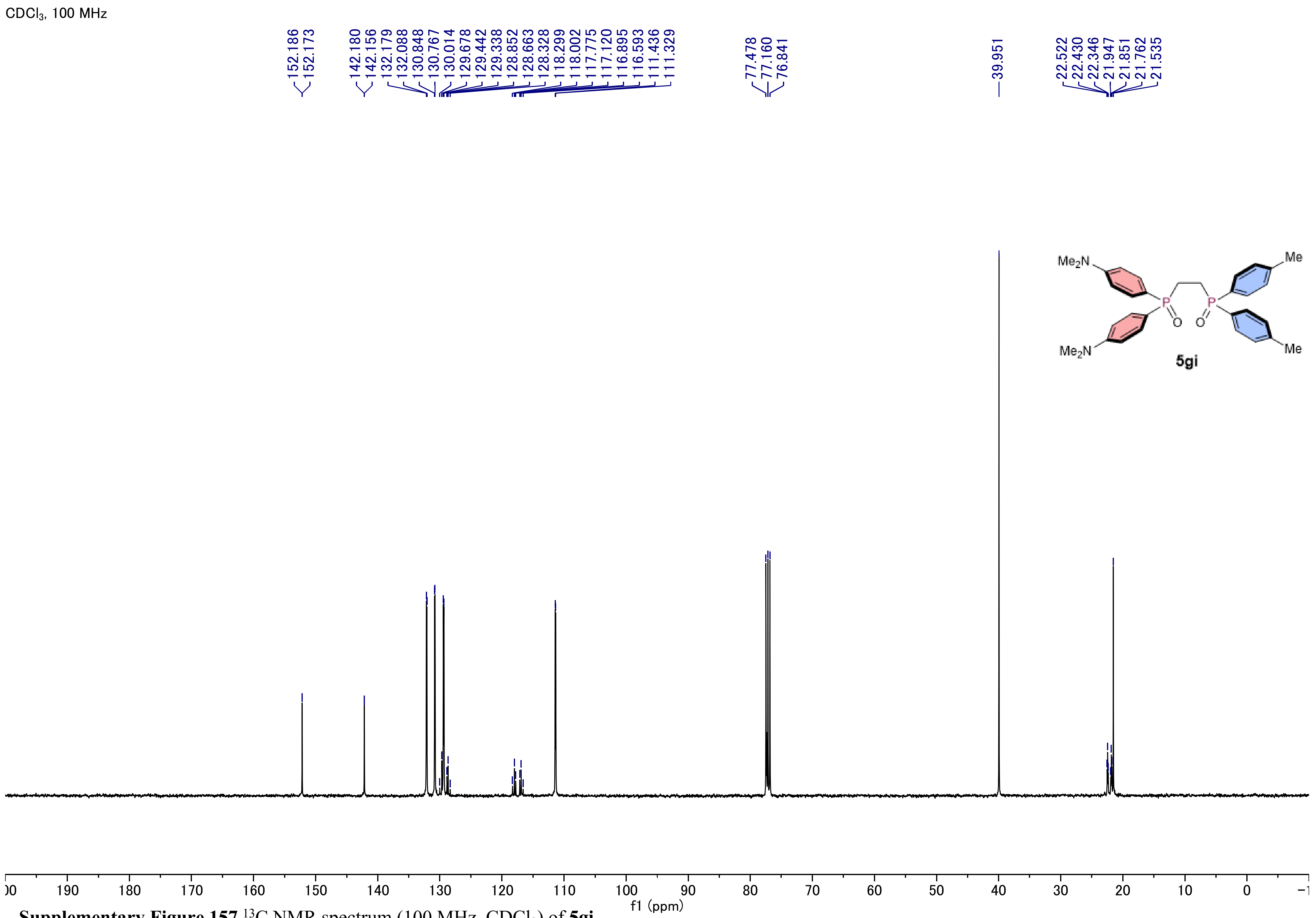

Supplementary Figure 157 ^{13}C NMR spectrum (100 MHz, $CDCl_3$) of **5gi**

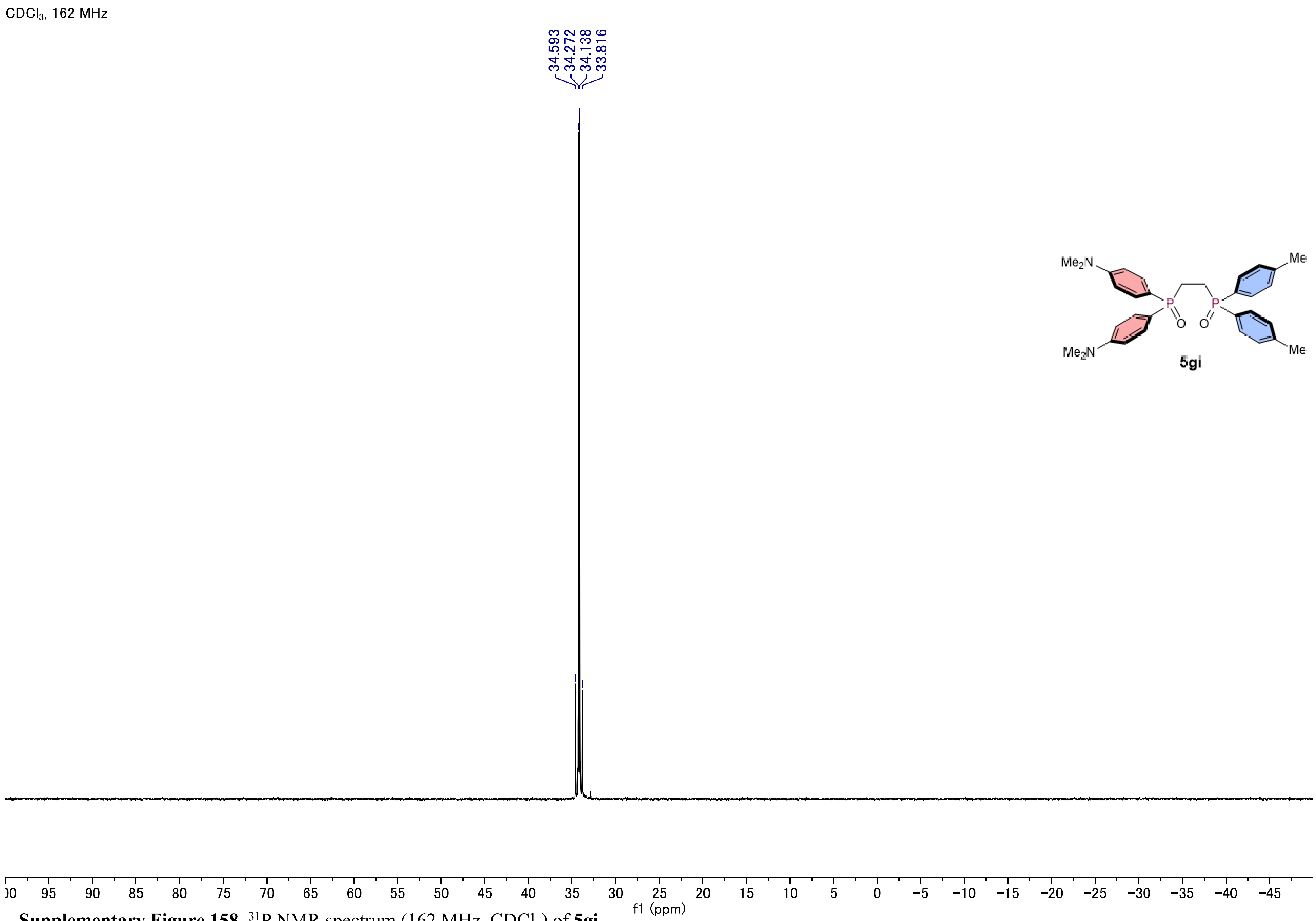

Supplementary Figure 158. ^{31}P NMR spectrum (162 MHz, $CDCl_3$) of **5gi**

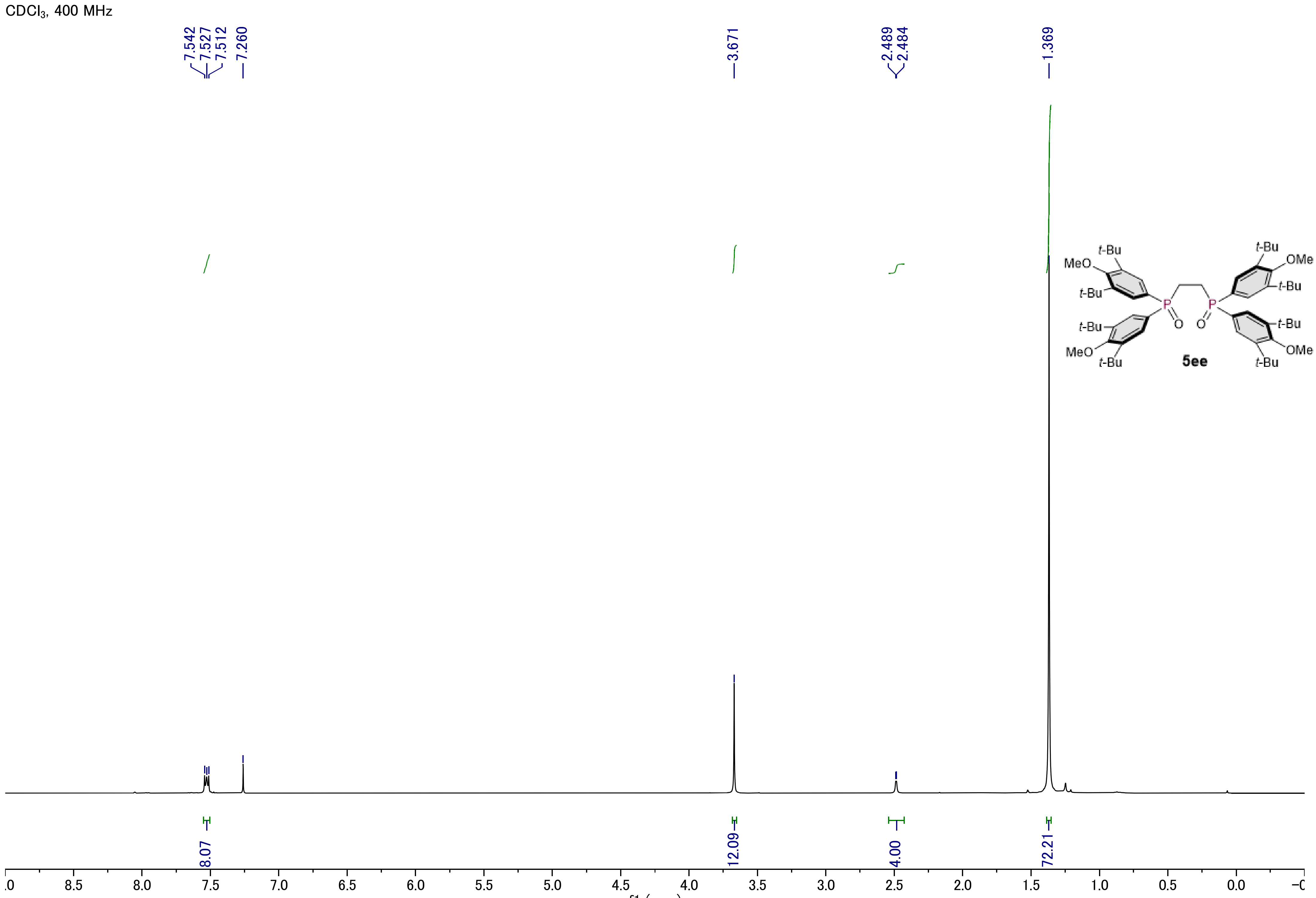

Supplementary Figure 159. ^{1}H NMR spectrum (400 MHz, $CDCl_3$) of **5ee**

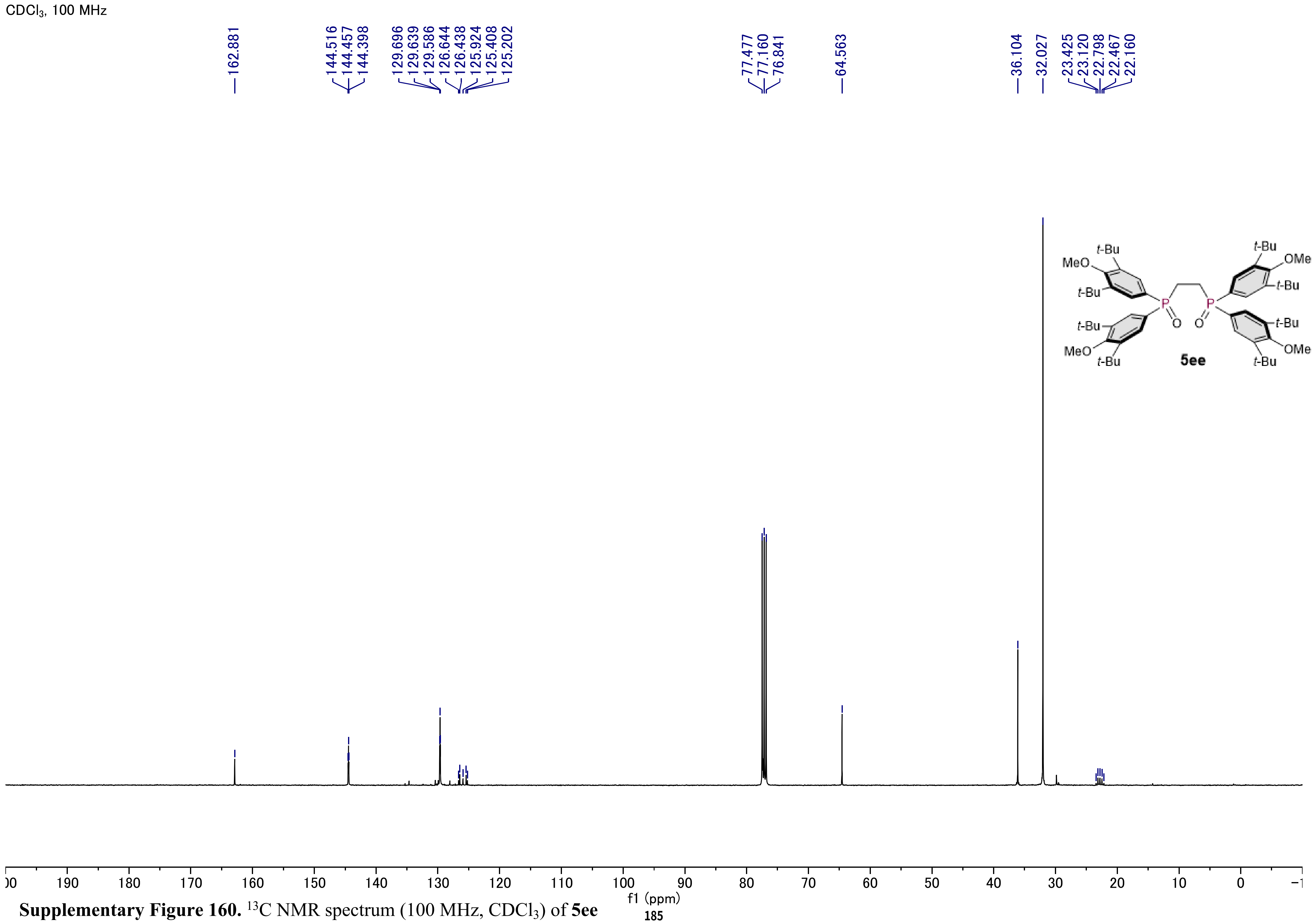

Supplementary Figure 160. ^{13}C NMR spectrum (100 MHz, $CDCl_3$) of **5ee**

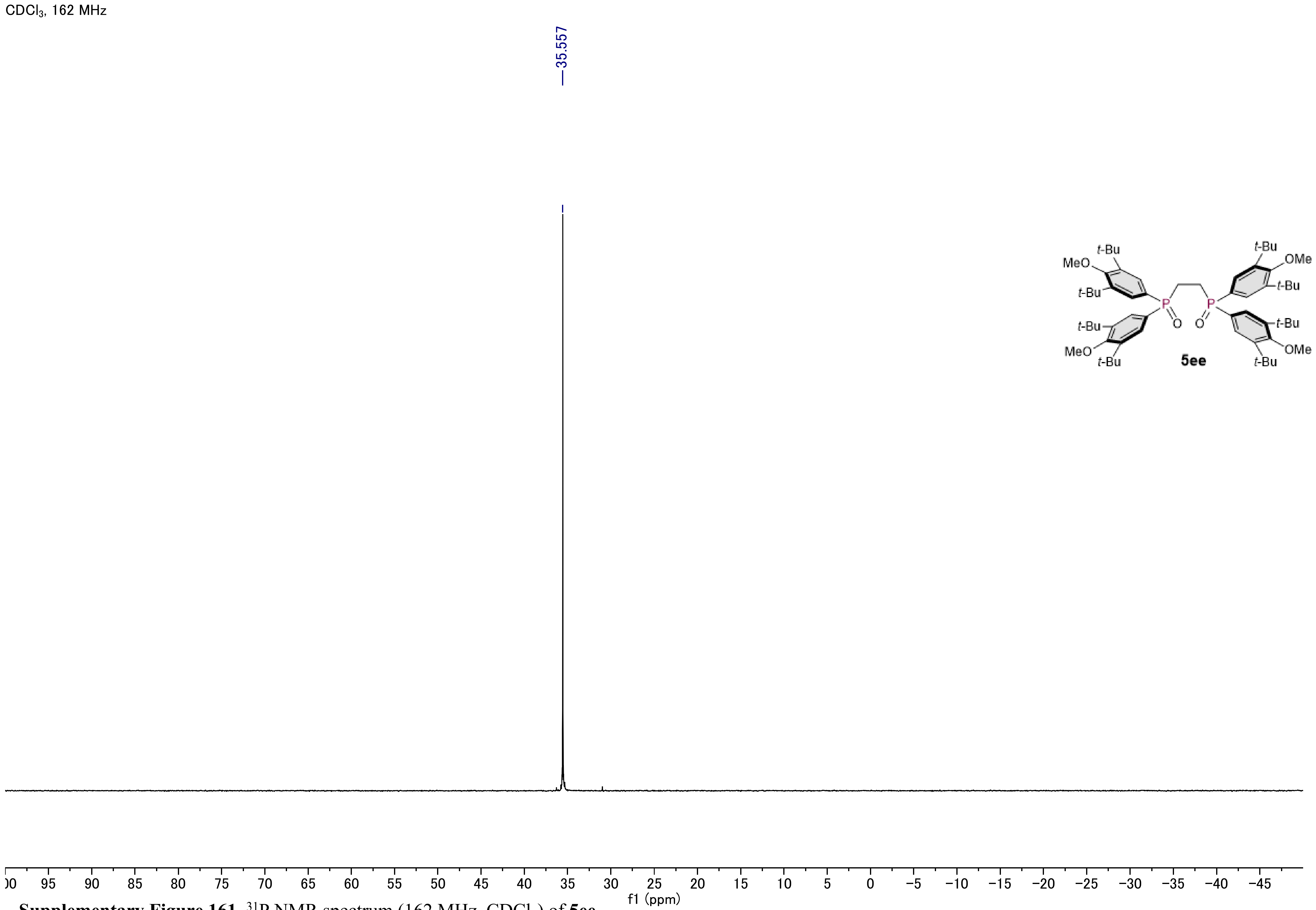

Supplementary Figure 161. ^{31}P NMR spectrum (162 MHz, CDCl$_3$) of **5ee**

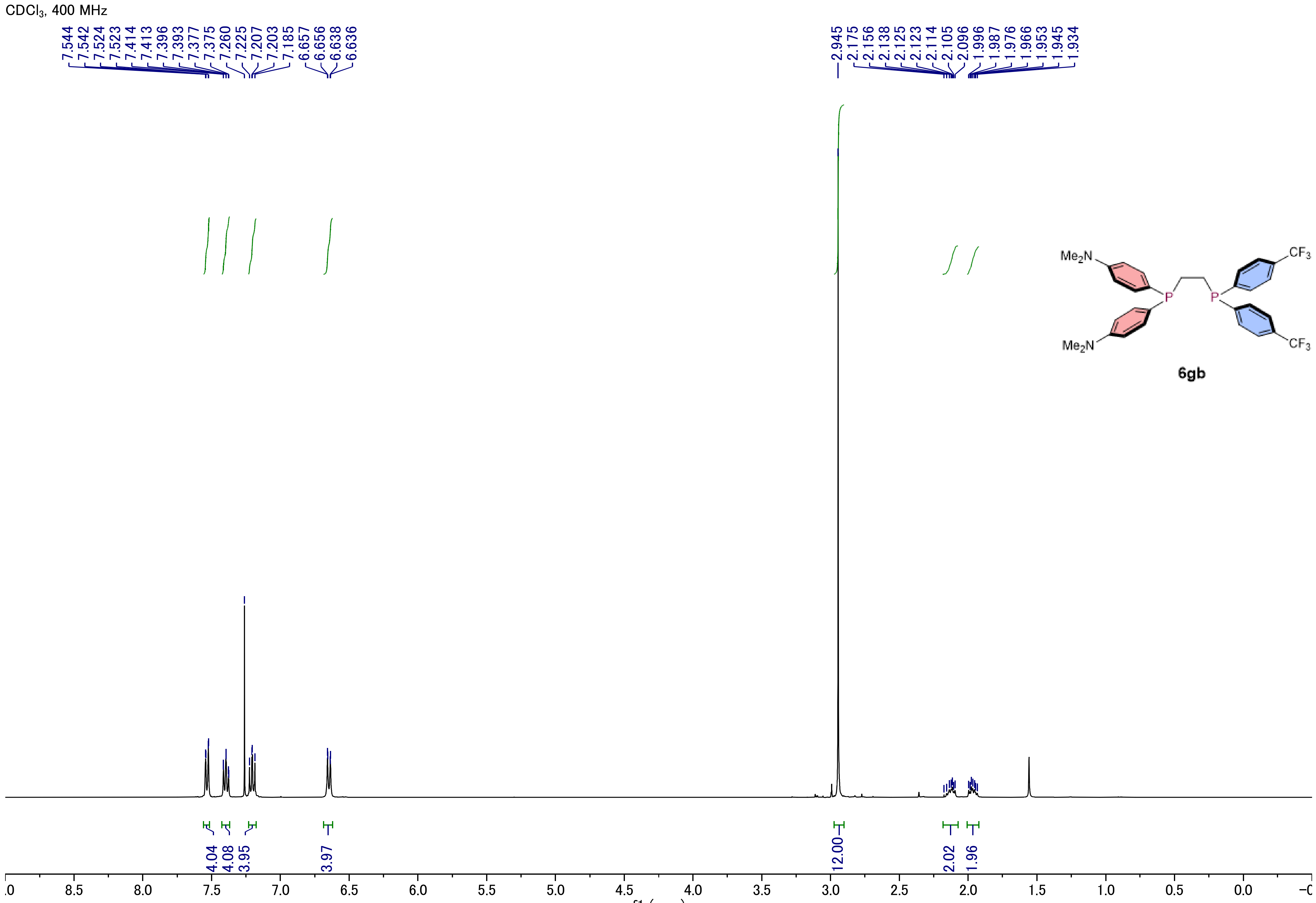

Supplementary Figure 162. ^{1}H NMR spectrum (400 MHz, $CDCl_3$) of **6gb**

CDCl$_3$, 100 MHz

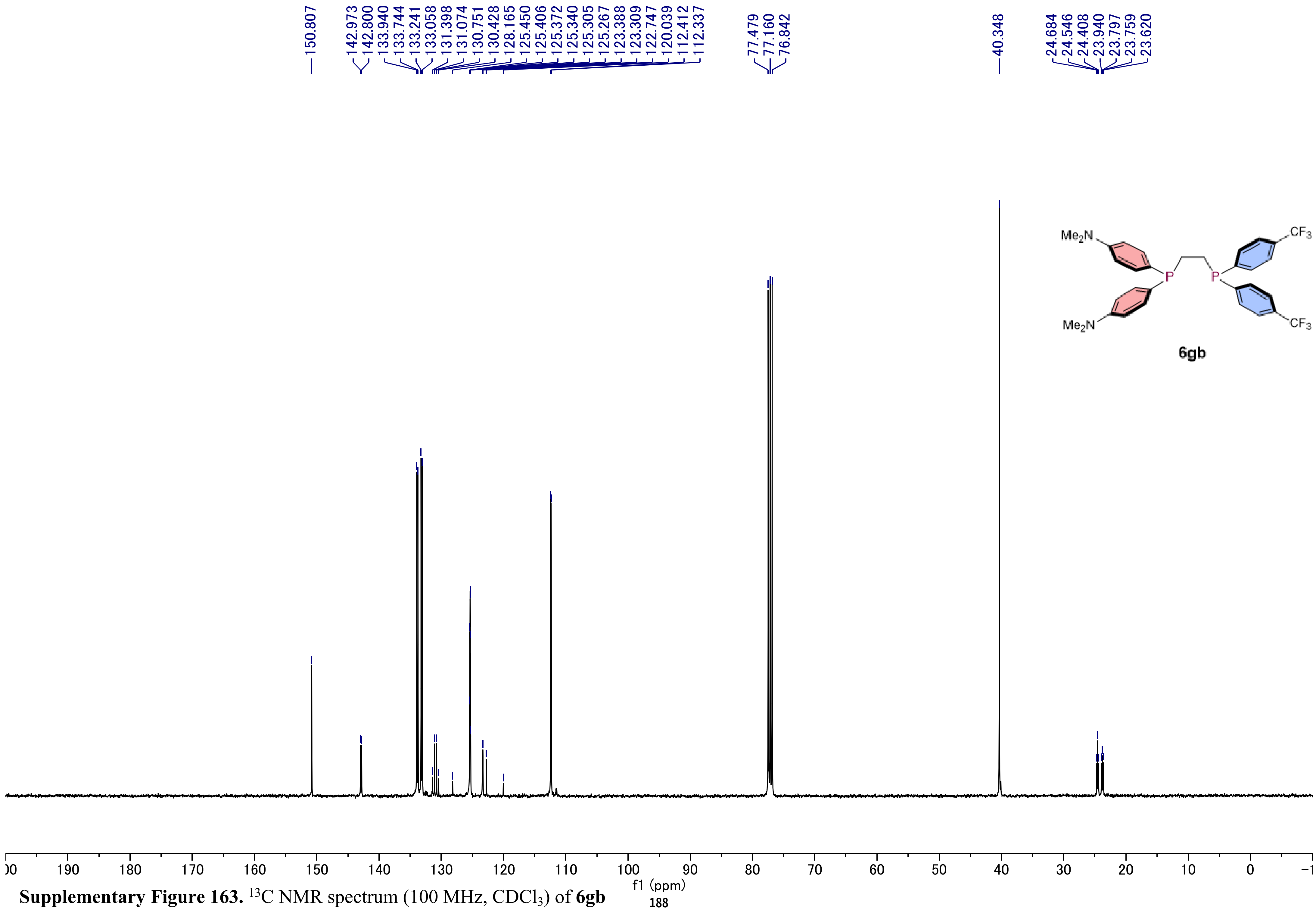

Supplementary Figure 163. ^{13}C NMR spectrum (100 MHz, CDCl$_3$) of **6gb**

CDCl$_3$, 376 MHz

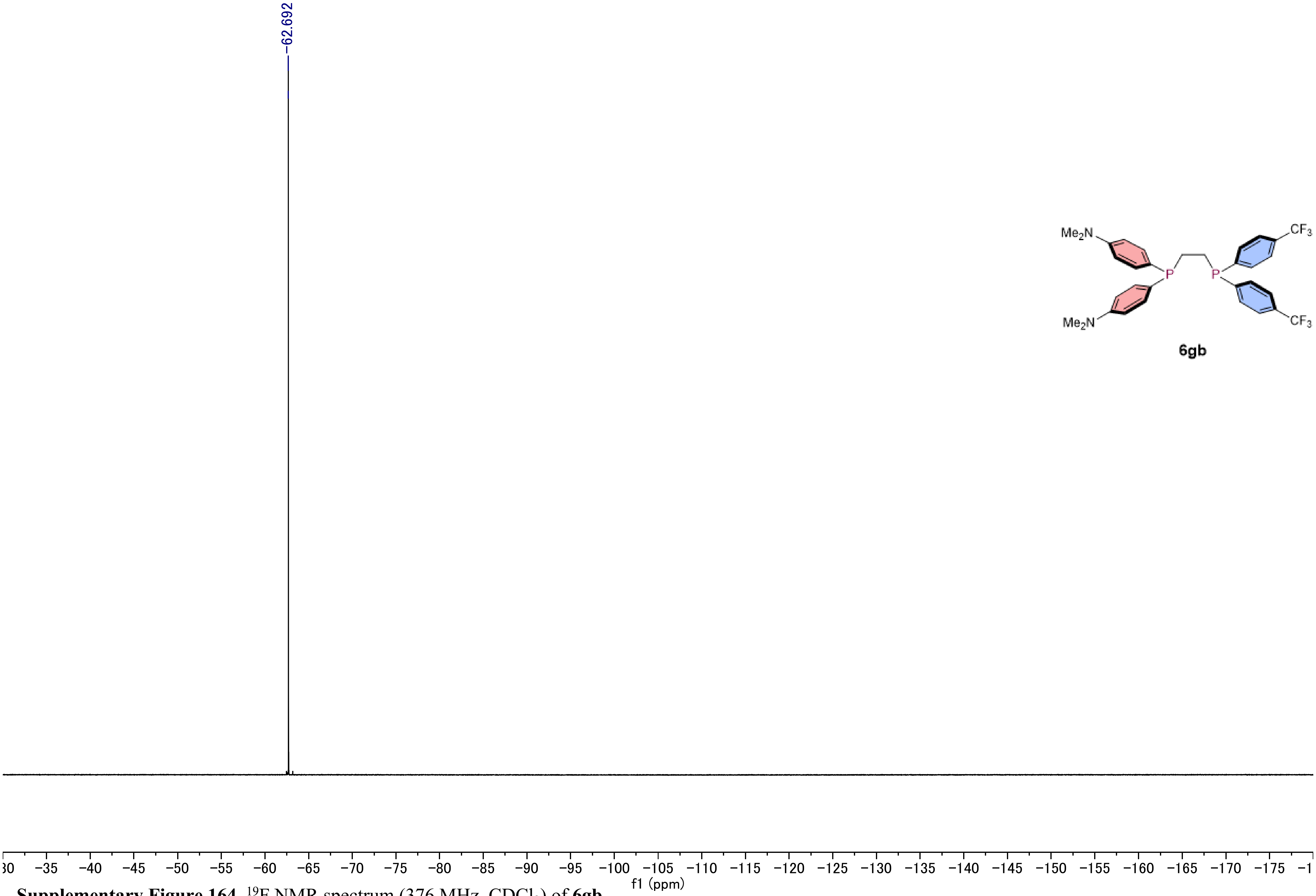

6gb

Supplementary Figure 164. ^{19}F NMR spectrum (376 MHz, CDCl$_3$) of **6gb**

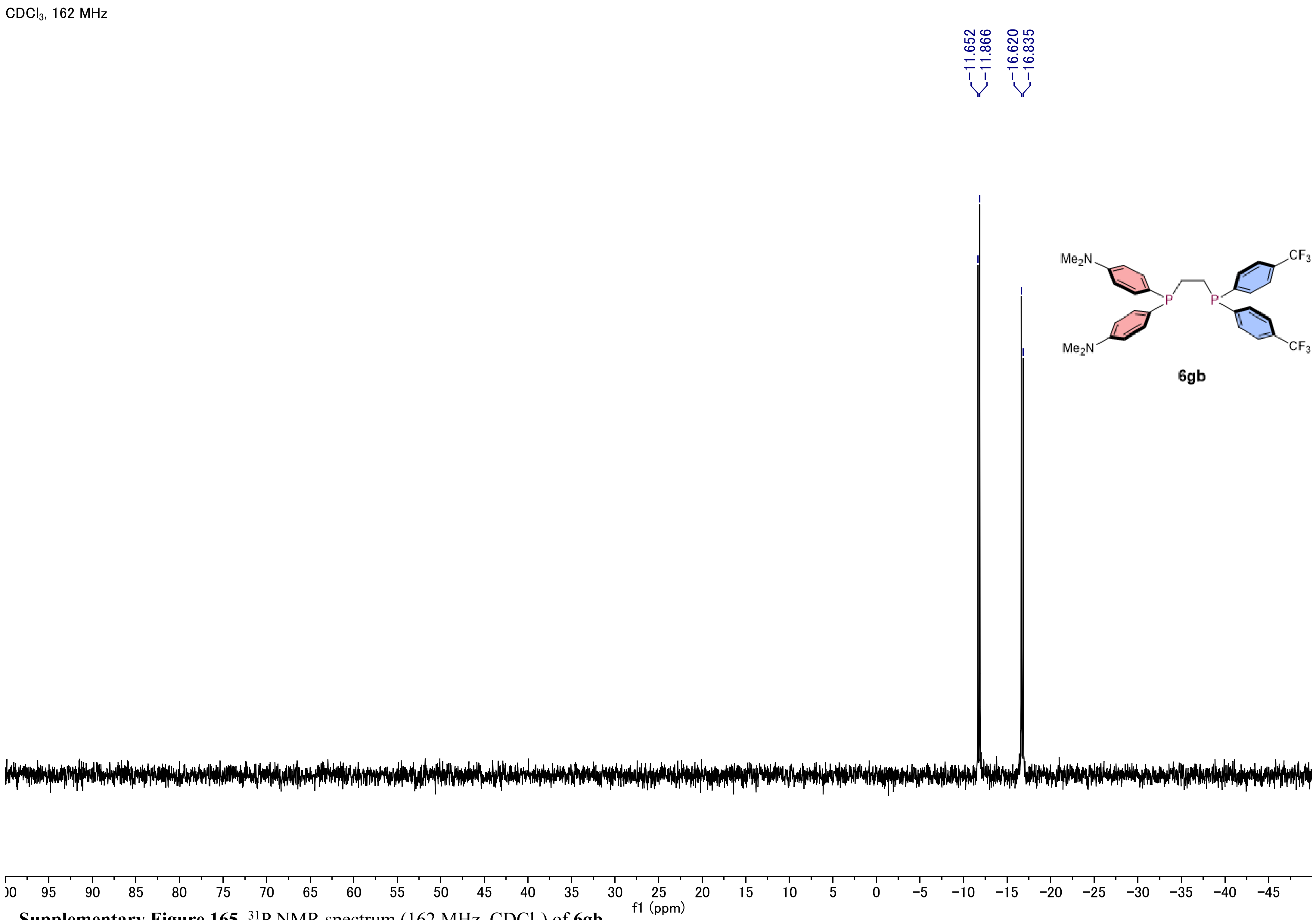

Supplementary Figure 165. ^{31}P NMR spectrum (162 MHz, $CDCl_3$) of **6gb**

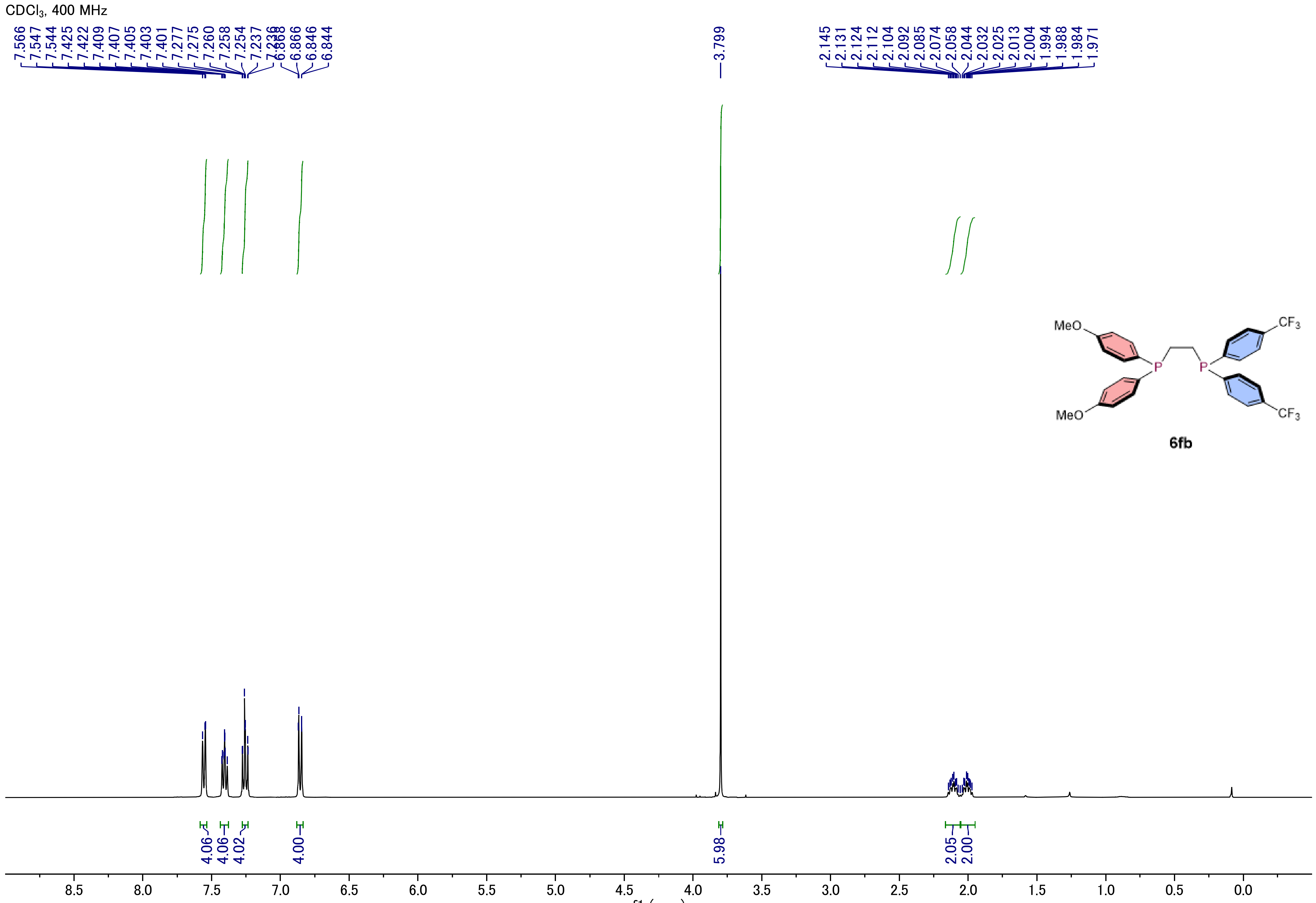

Supplementary Figure 166. ^{1}H NMR spectrum (400 MHz, $CDCl_3$) of **6fb**

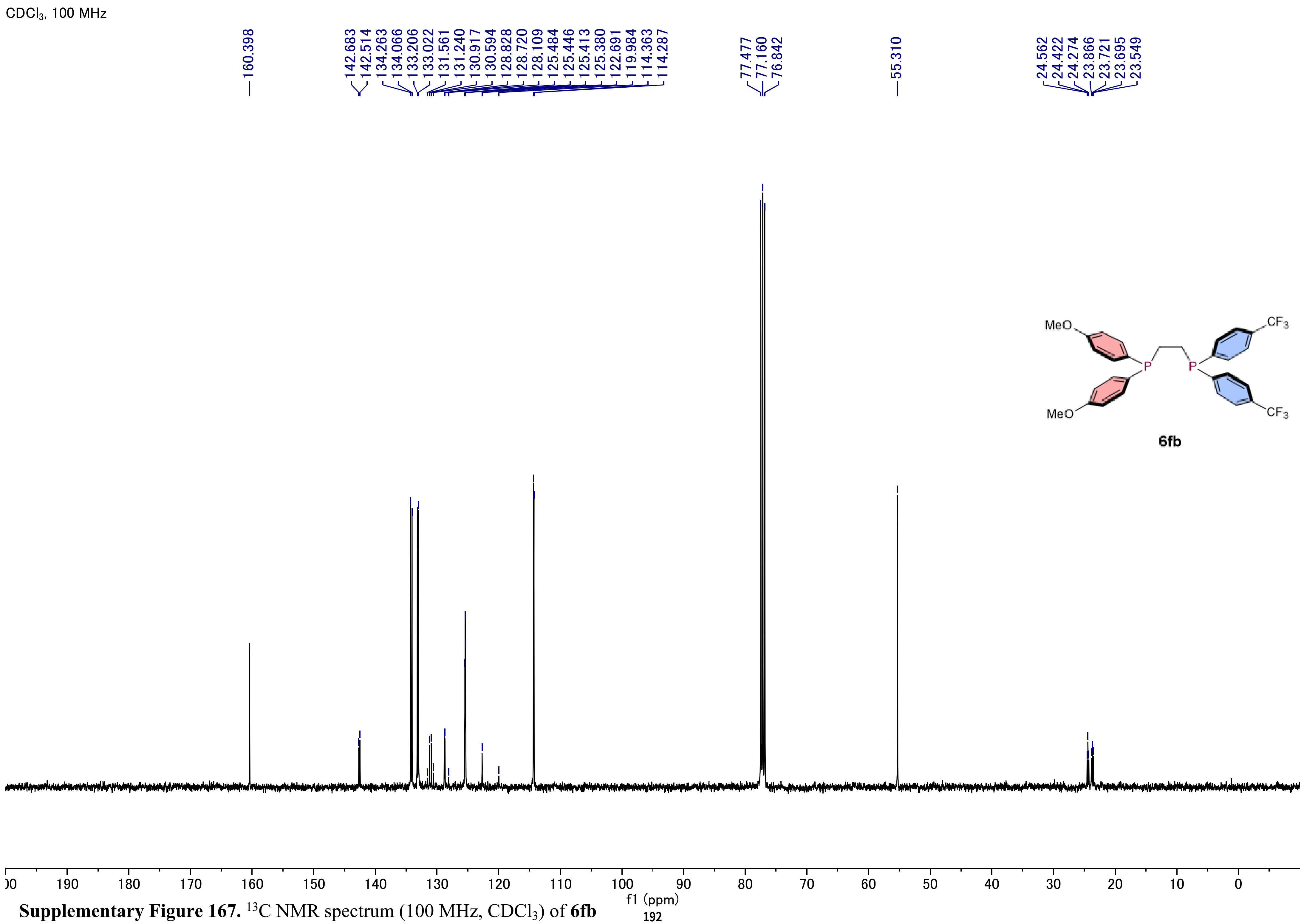

Supplementary Figure 167. ^{13}C NMR spectrum (100 MHz, $CDCl_3$) of **6fb**

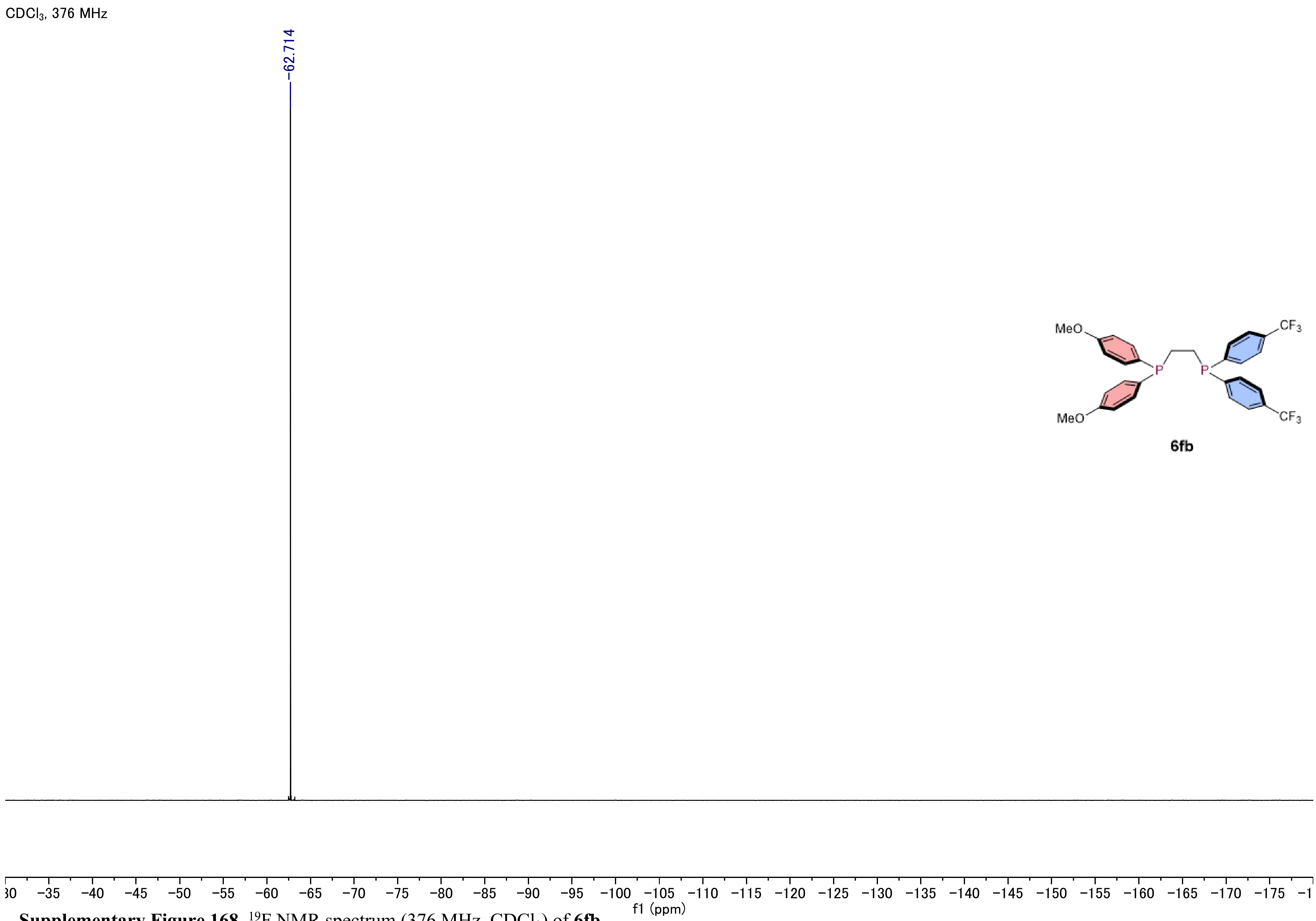

Supplementary Figure 168. ^{19}F NMR spectrum (376 MHz, $CDCl_3$) of **6fb**

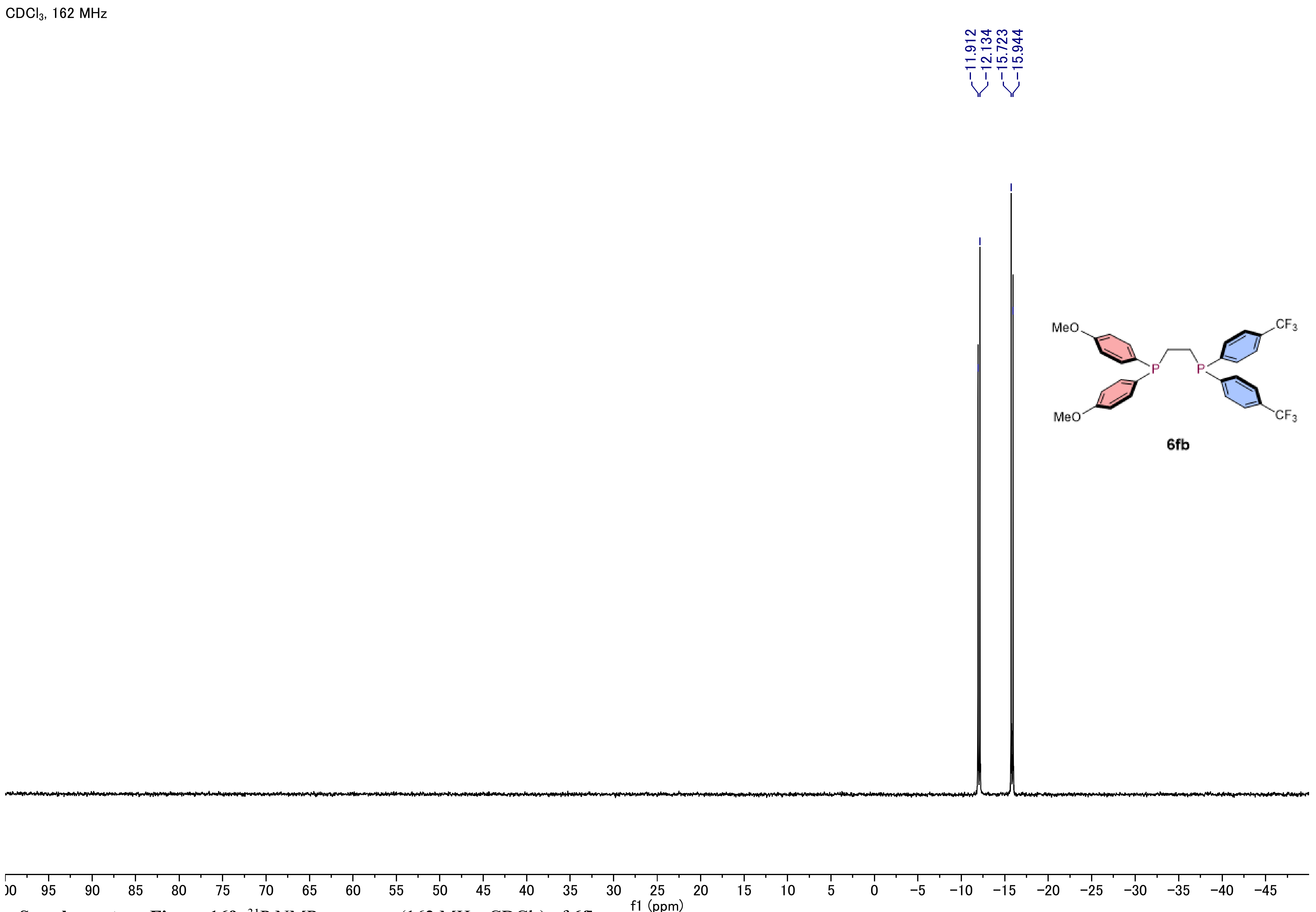

Supplementary Figure 169. ^{31}P NMR spectrum (162 MHz, $CDCl_3$) of **6fb**

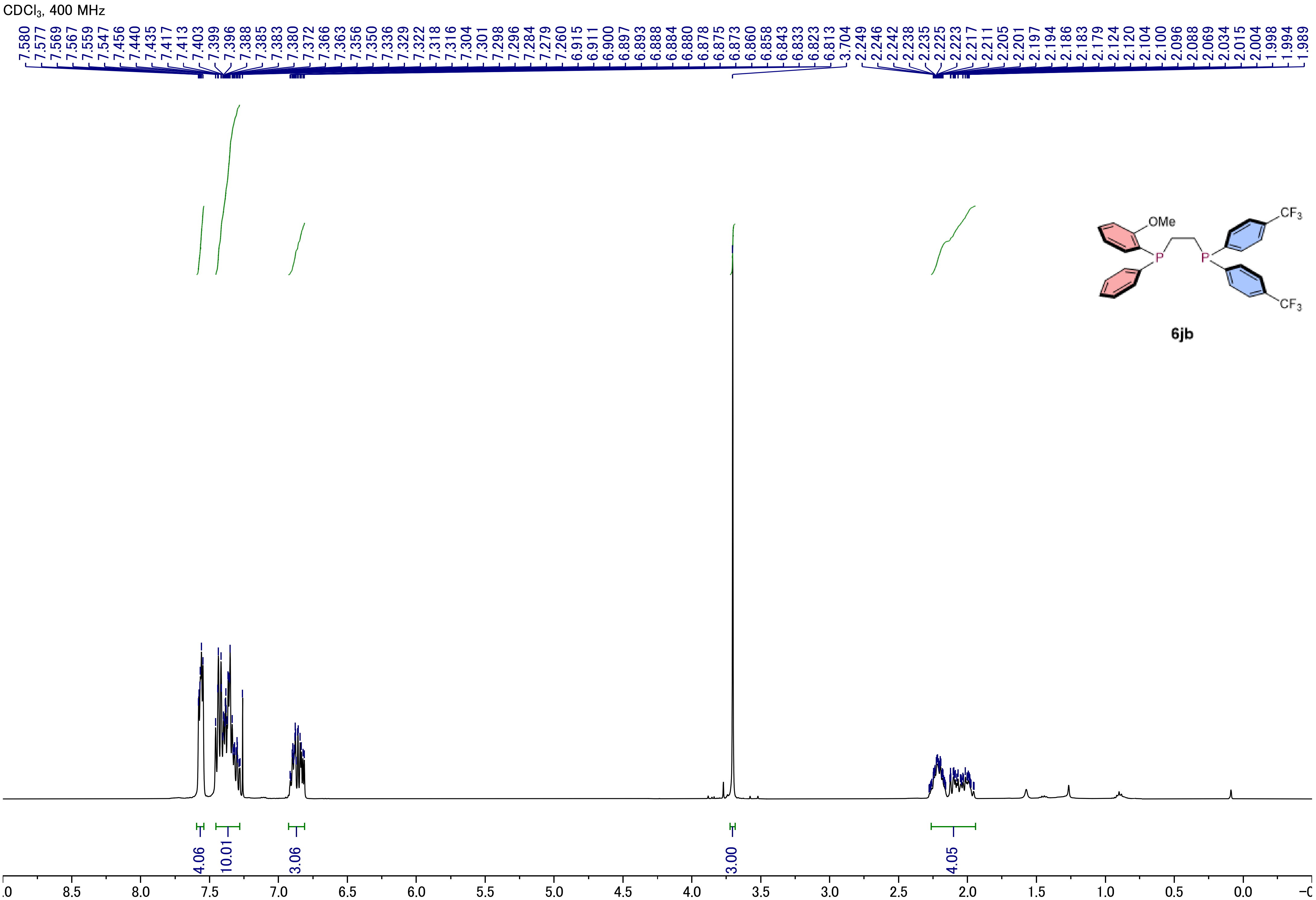

Supplementary Figure 170. ^{1}H NMR spectrum (400 MHz, $CDCl_3$) of **6jb**

CDCl$_3$, 100 MHz

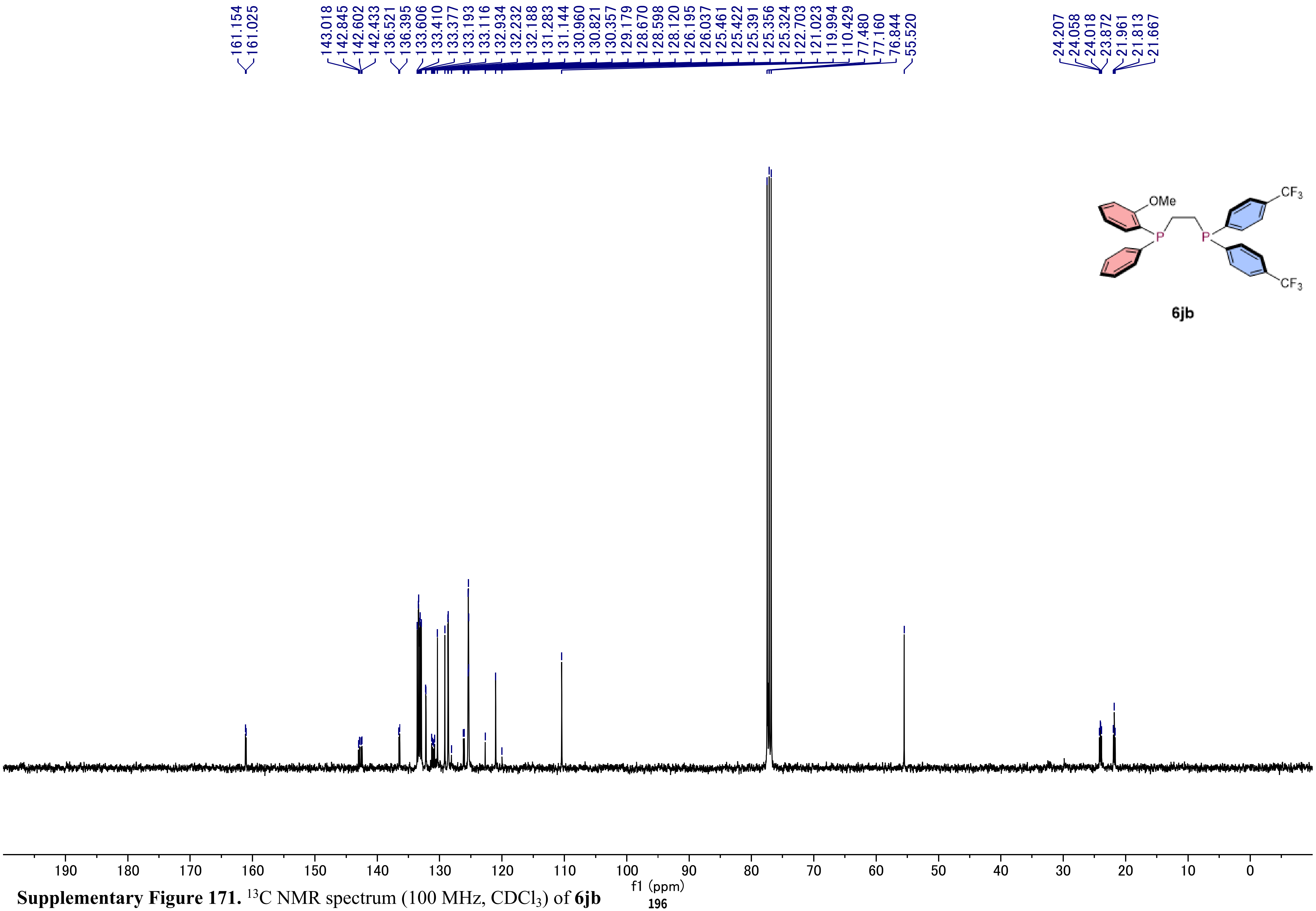

Supplementary Figure 171. ^{13}C NMR spectrum (100 MHz, CDCl$_3$) of **6jb**

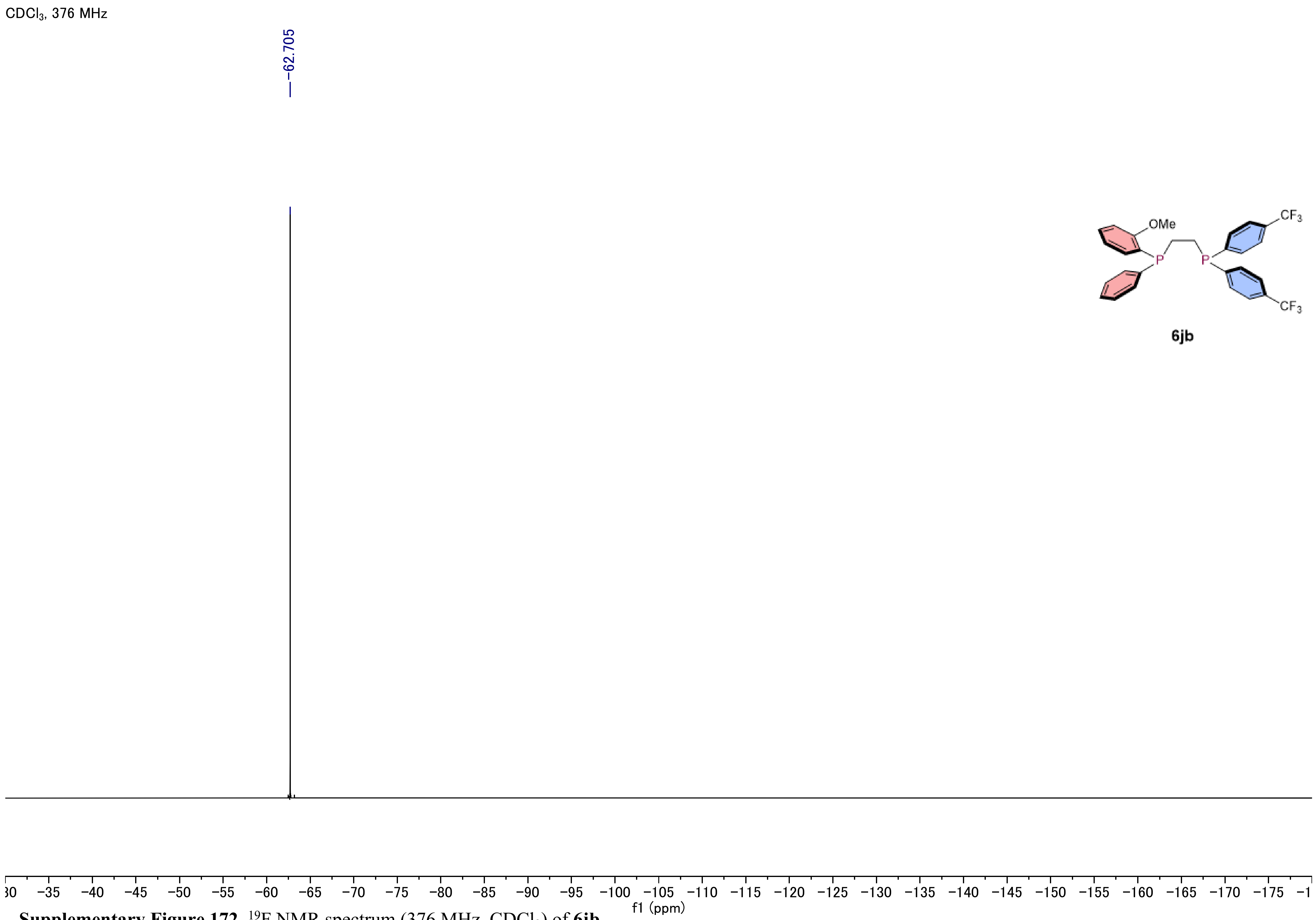

Supplementary Figure 172. ^{19}F NMR spectrum (376 MHz, $CDCl_3$) of **6jb**

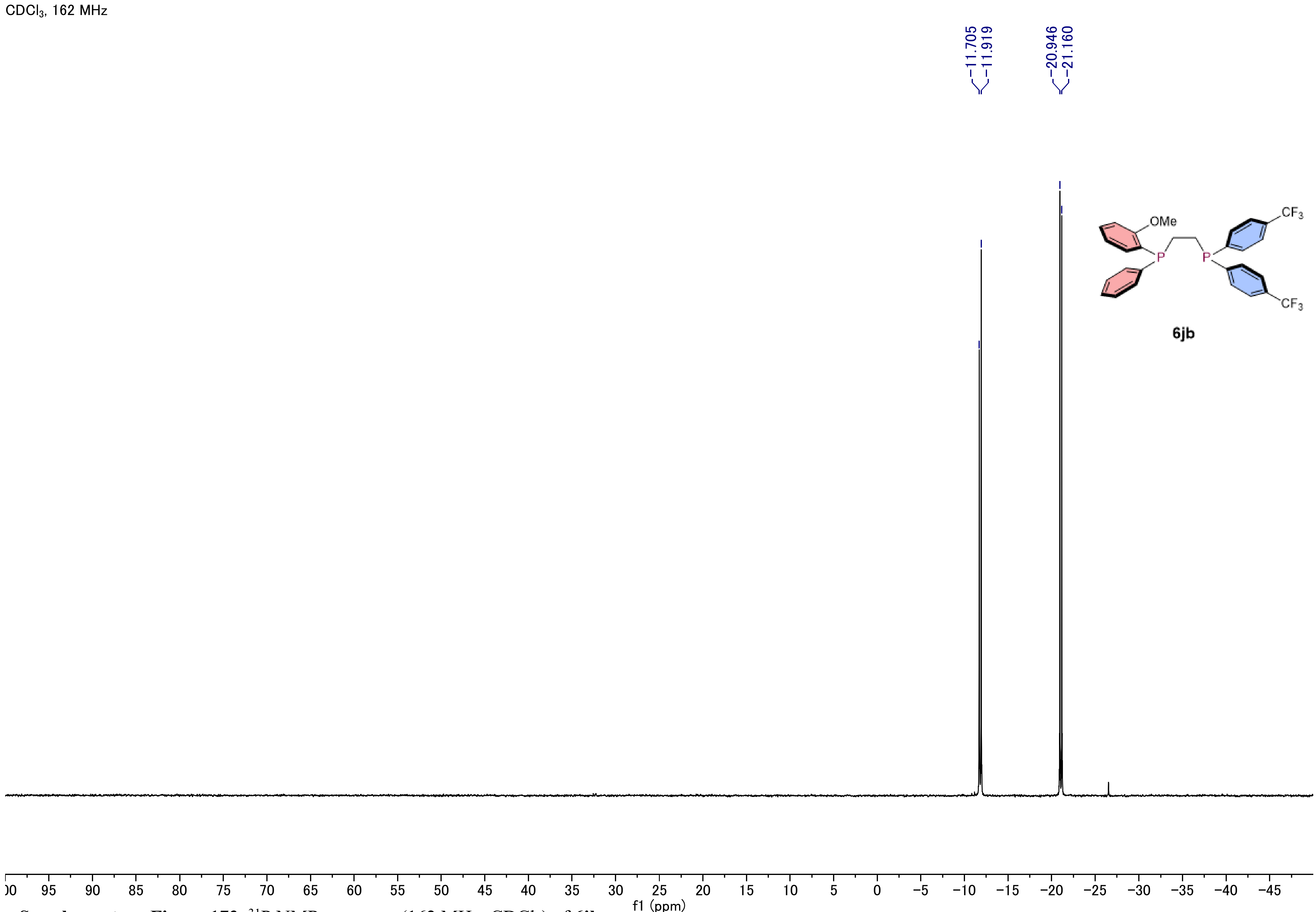

Supplementary Figure 173. ^{31}P NMR spectrum (162 MHz, $CDCl_3$) of **6jb**

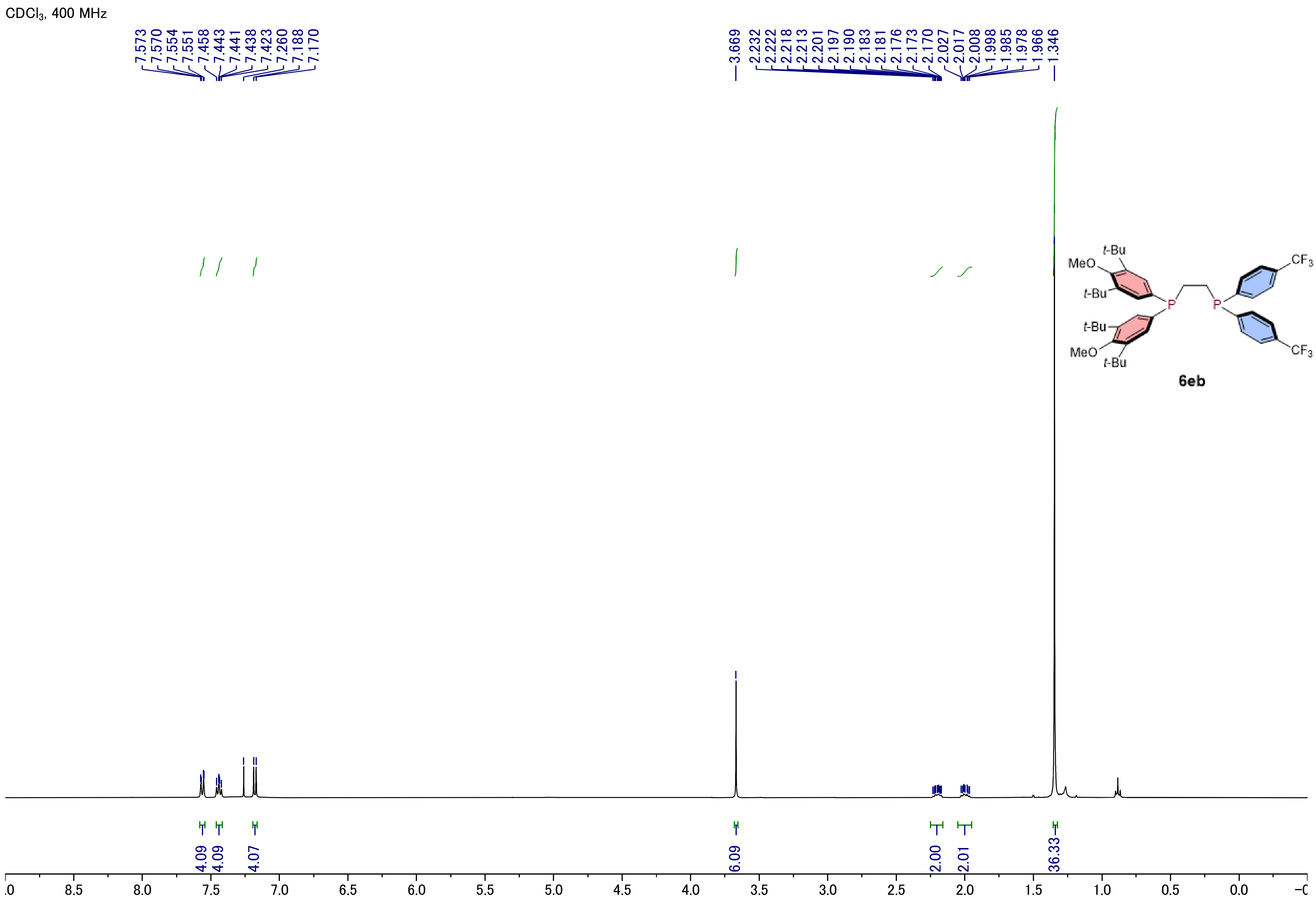

Supplementary Figure 174. ^{1}H NMR spectrum (400 MHz, $CDCl_3$) of **6eb**

CDCl3, 100 MHz

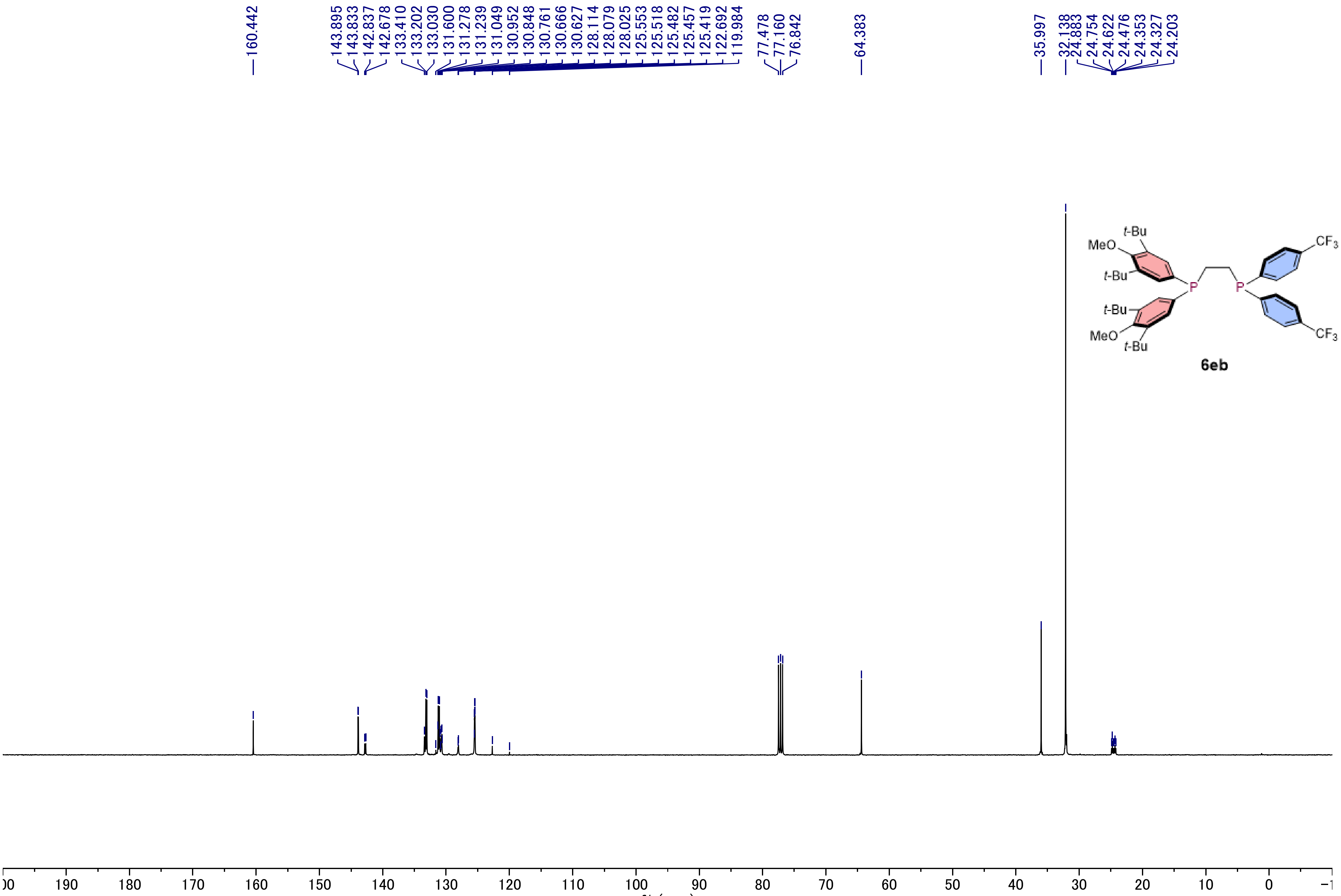

Supplementary Figure 175. ^{13}C NMR spectrum (100 MHz, $CDCl_3$) of **6eb**

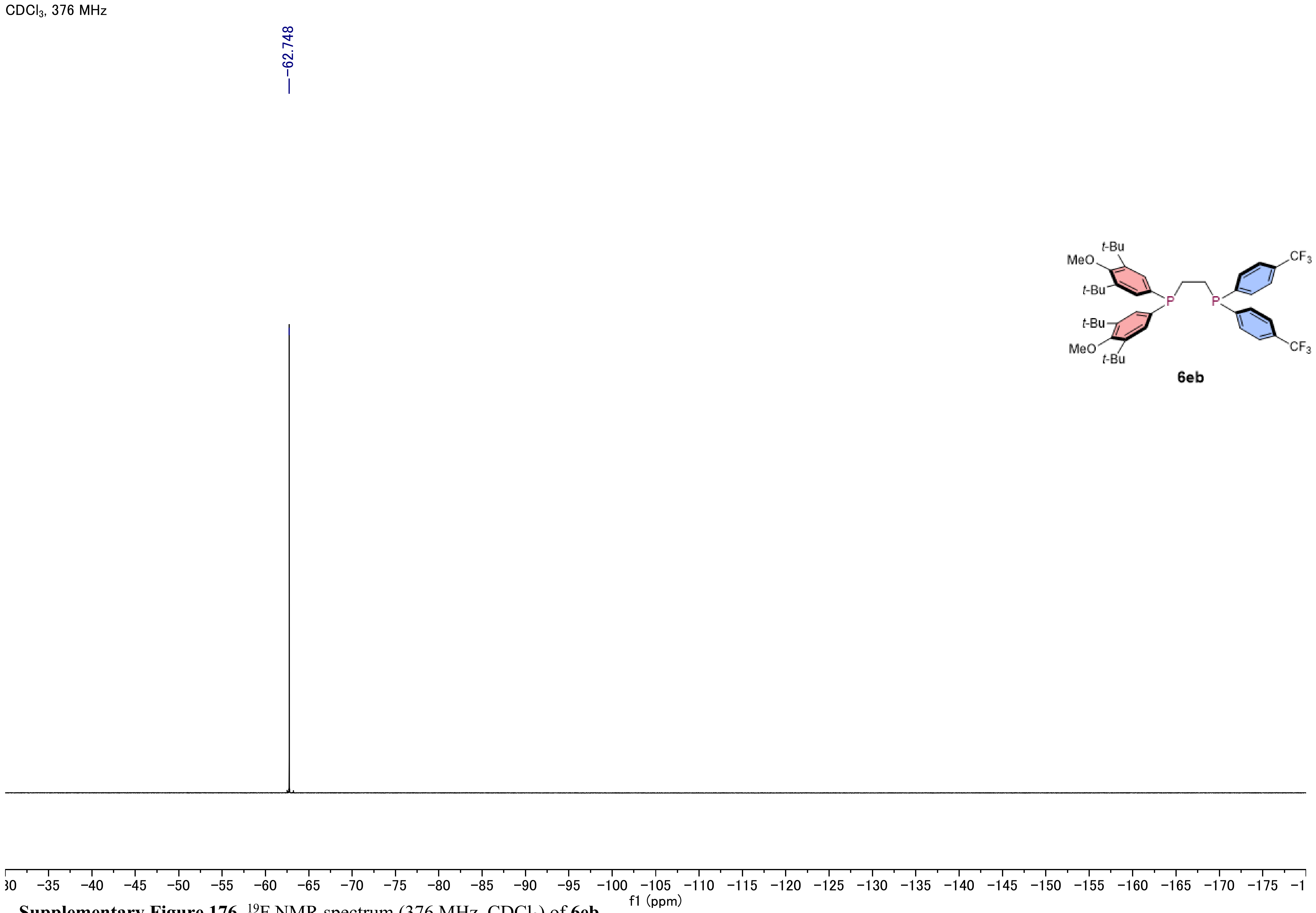

6eb

Supplementary Figure 176. ^{19}F NMR spectrum (376 MHz, $CDCl_3$) of **6eb**

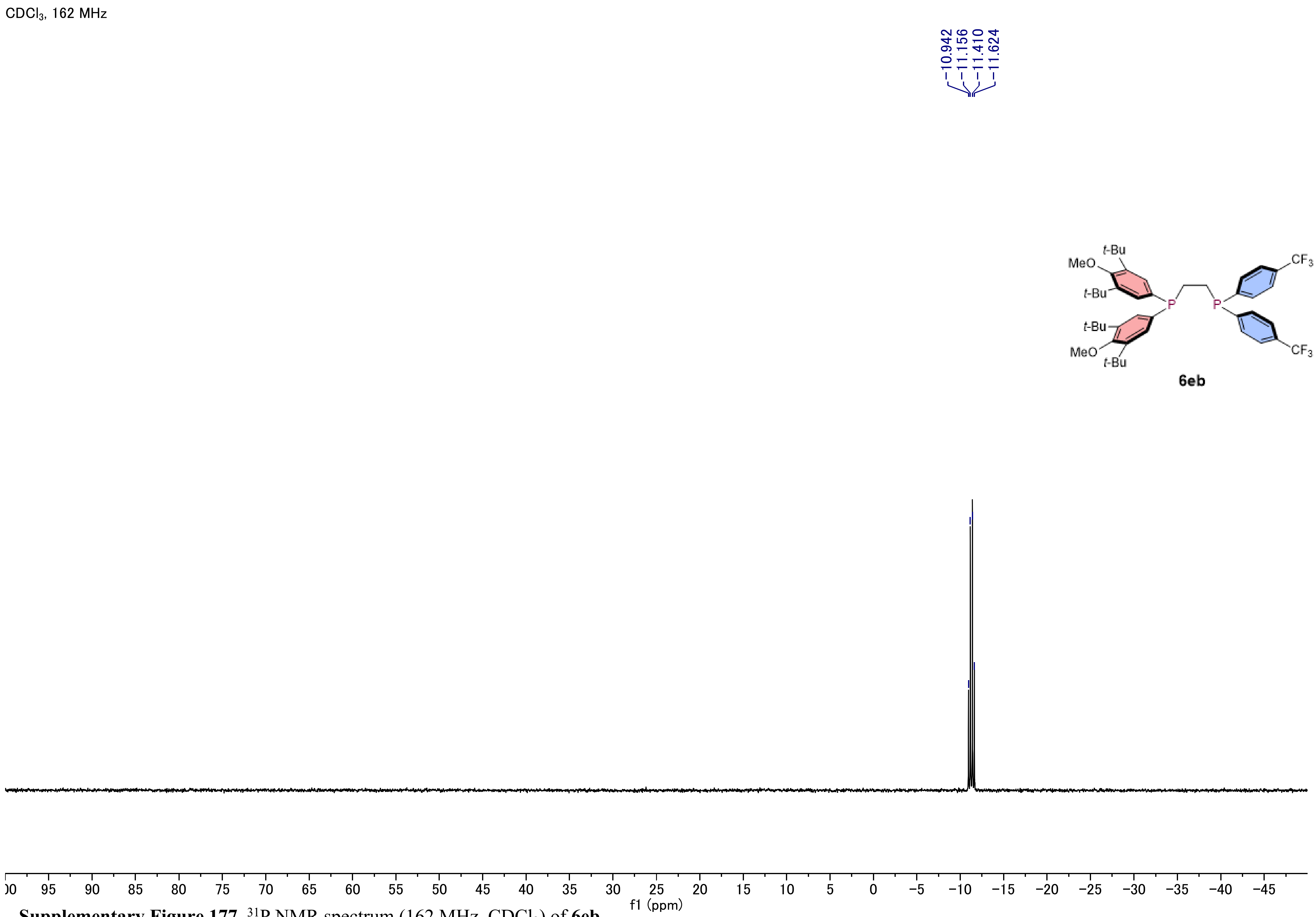

6eb

Supplementary Figure 177. ^{31}P NMR spectrum (162 MHz, $CDCl_3$) of **6eb**

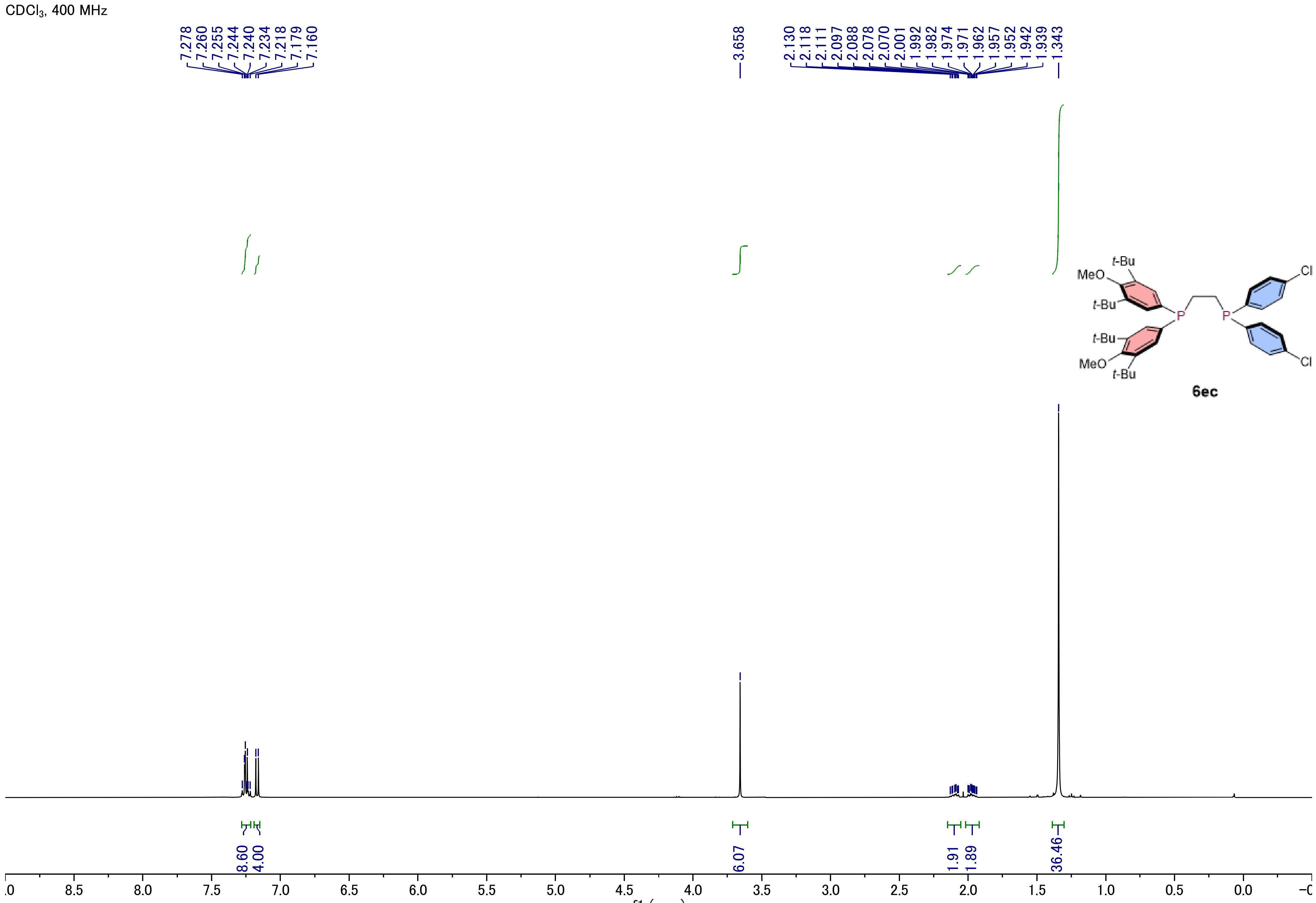

Supplementary Figure 178. ^{1}H NMR spectrum (400 MHz, $CDCl_3$) of **6ec**

CDCl$_3$, 100 MHz

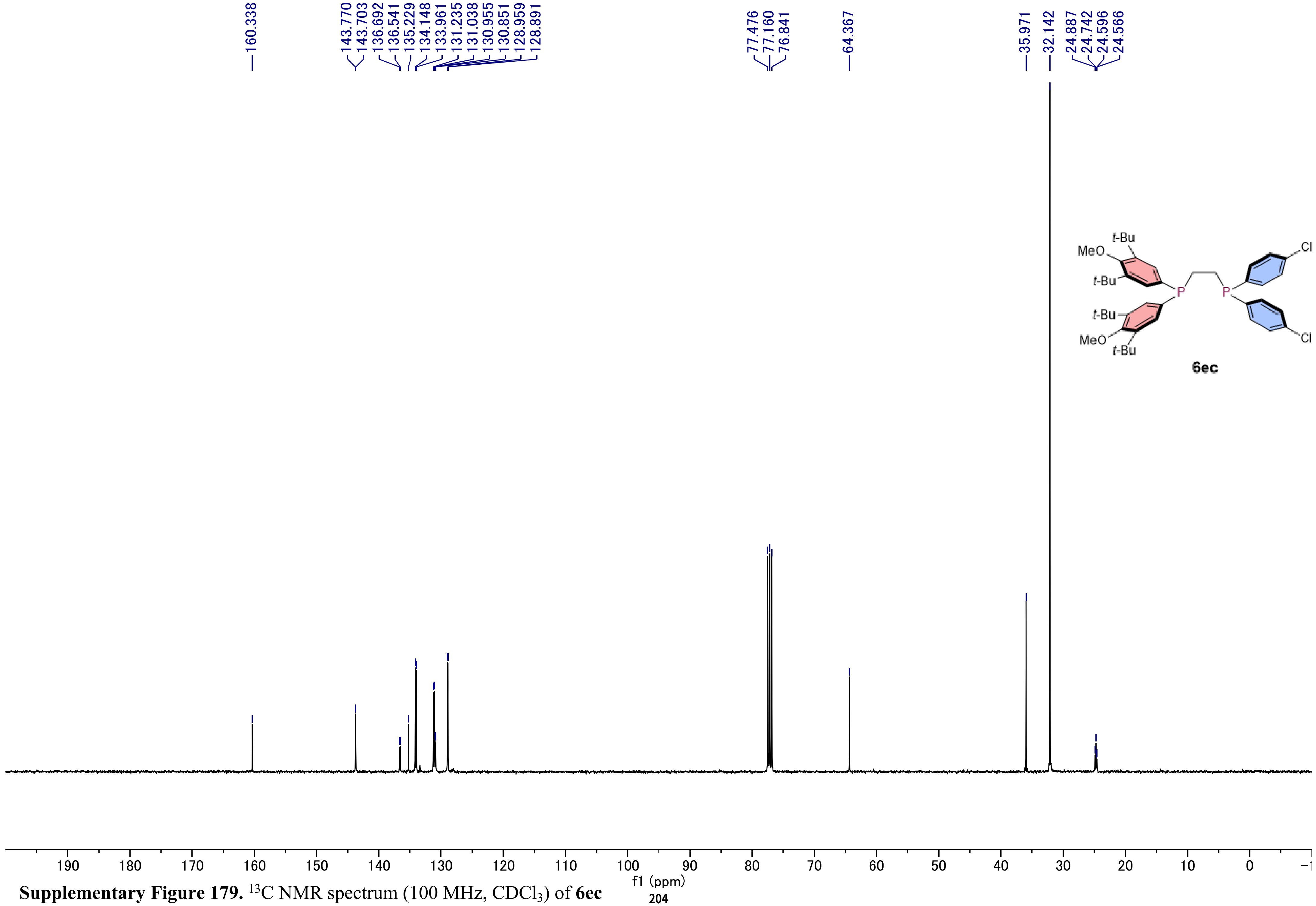

6ec

Supplementary Figure 179. ^{13}C NMR spectrum (100 MHz, CDCl$_3$) of **6ec**

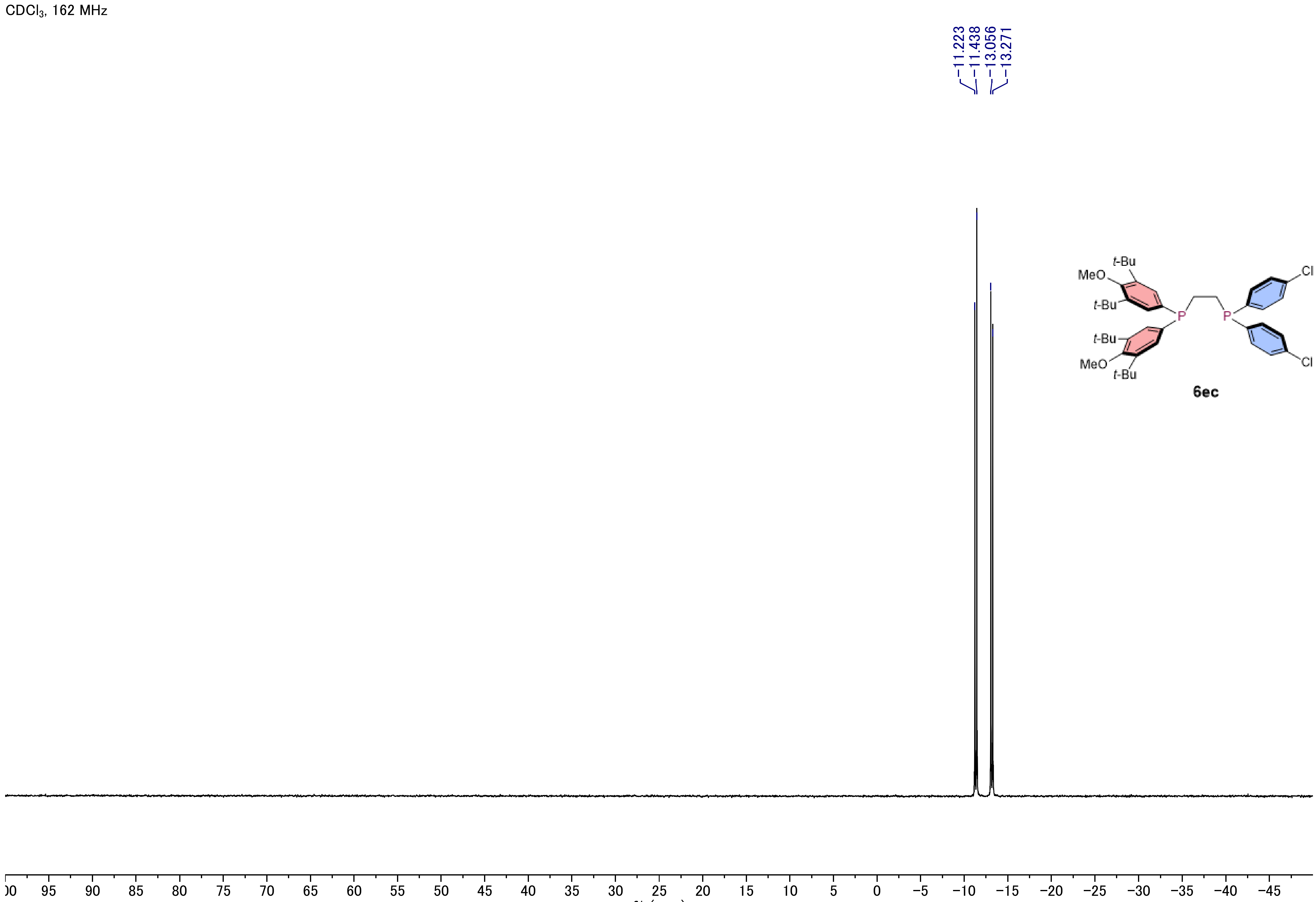

Supplementary Figure 180. ^{31}P NMR spectrum (162 MHz, $CDCl_3$) of **6ec**

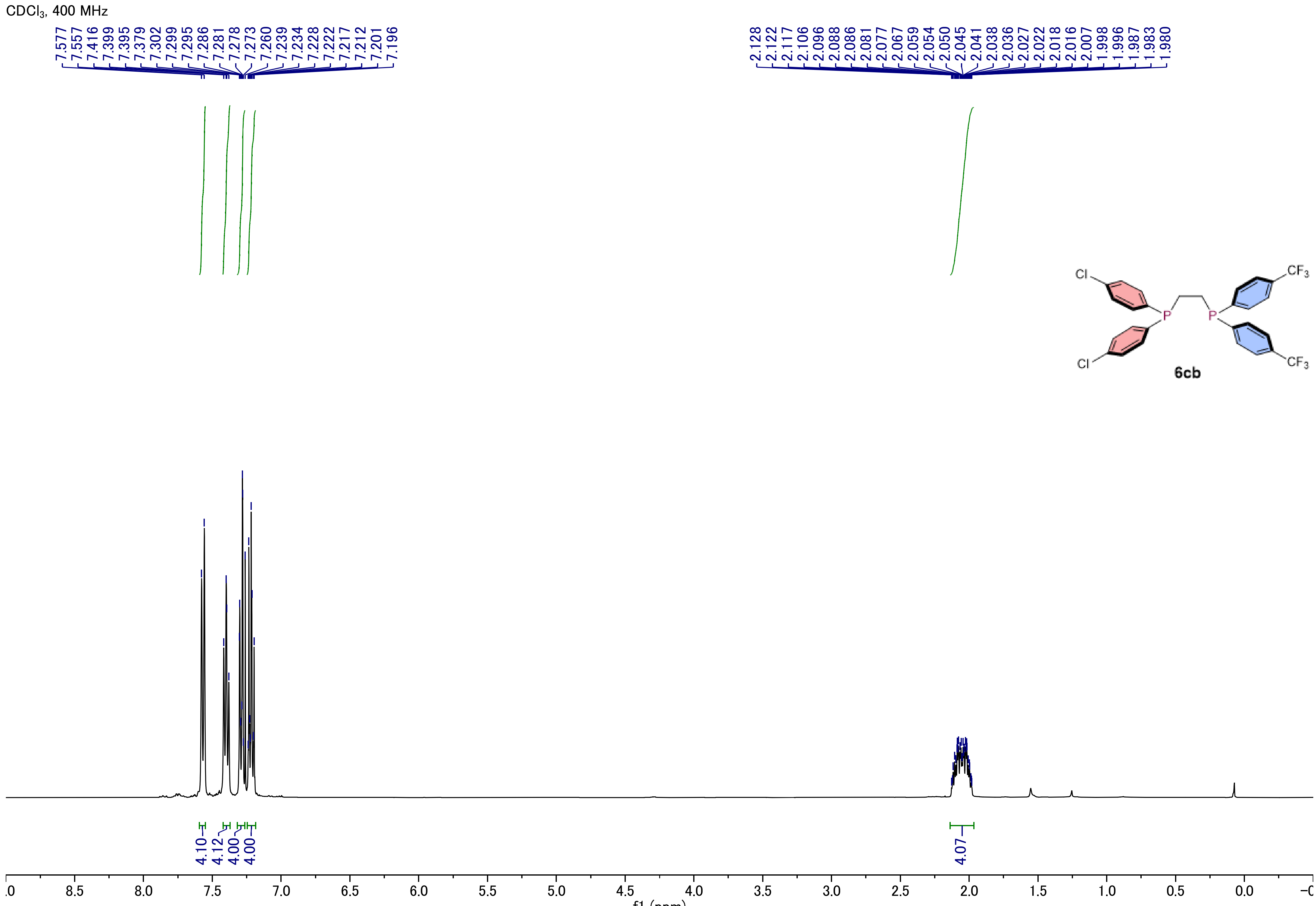

Supplementary Figure 181. ^{1}H NMR spectrum (400 MHz, $CDCl_3$) of **6cb**

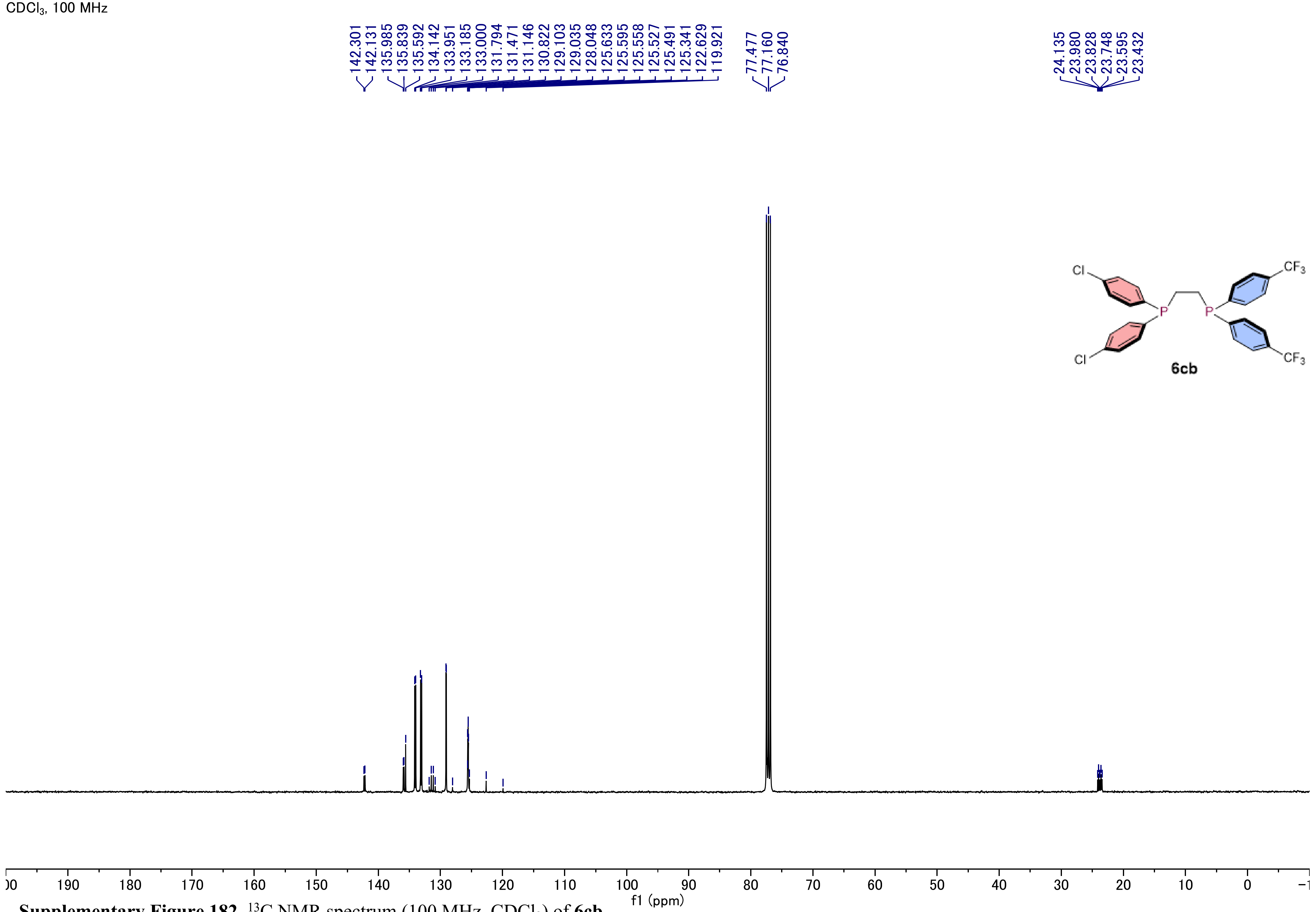

Supplementary Figure 182. ^{13}C NMR spectrum (100 MHz, $CDCl_3$) of **6cb**

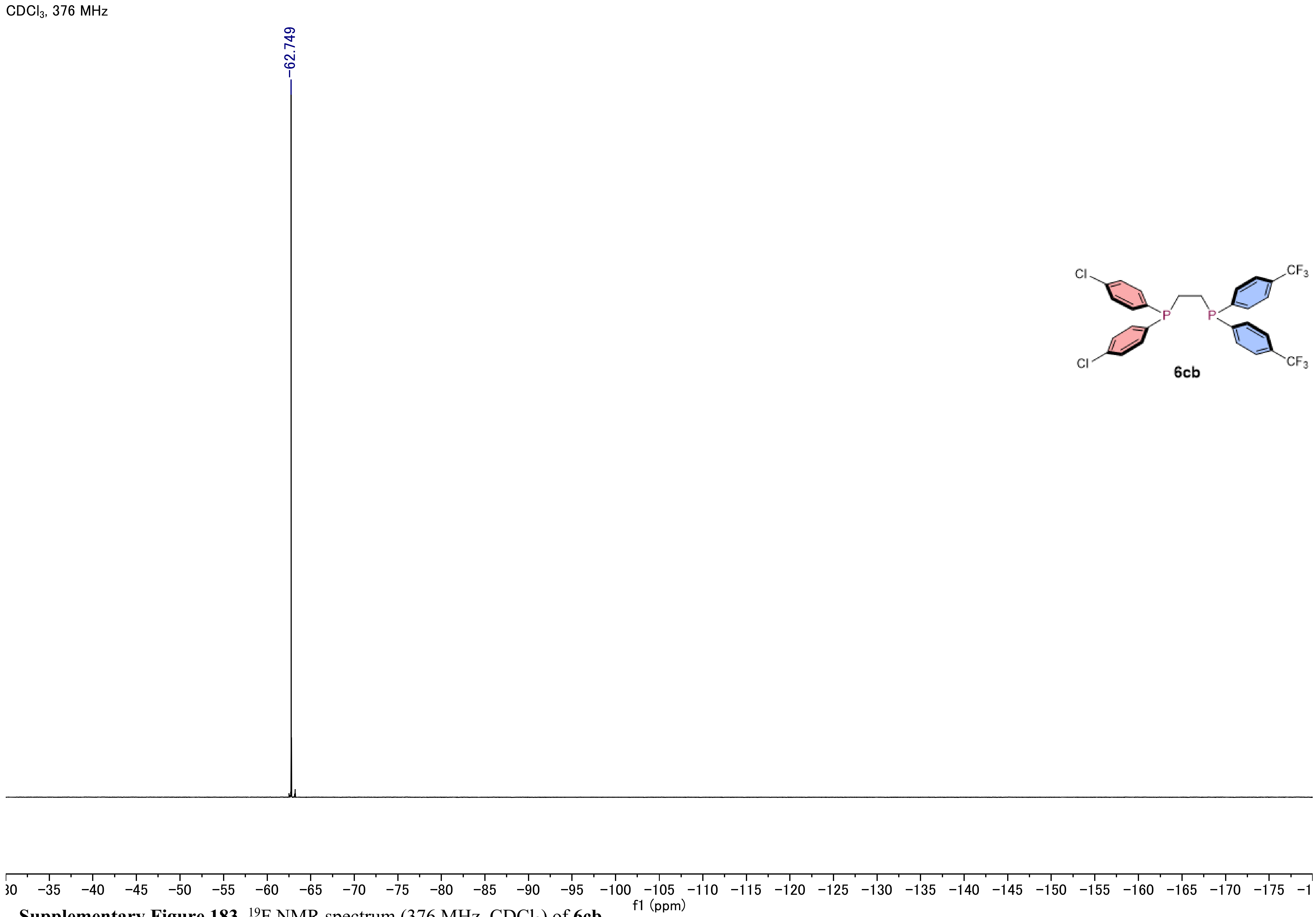

Supplementary Figure 183. ^{19}F NMR spectrum (376 MHz, $CDCl_3$) of **6cb**

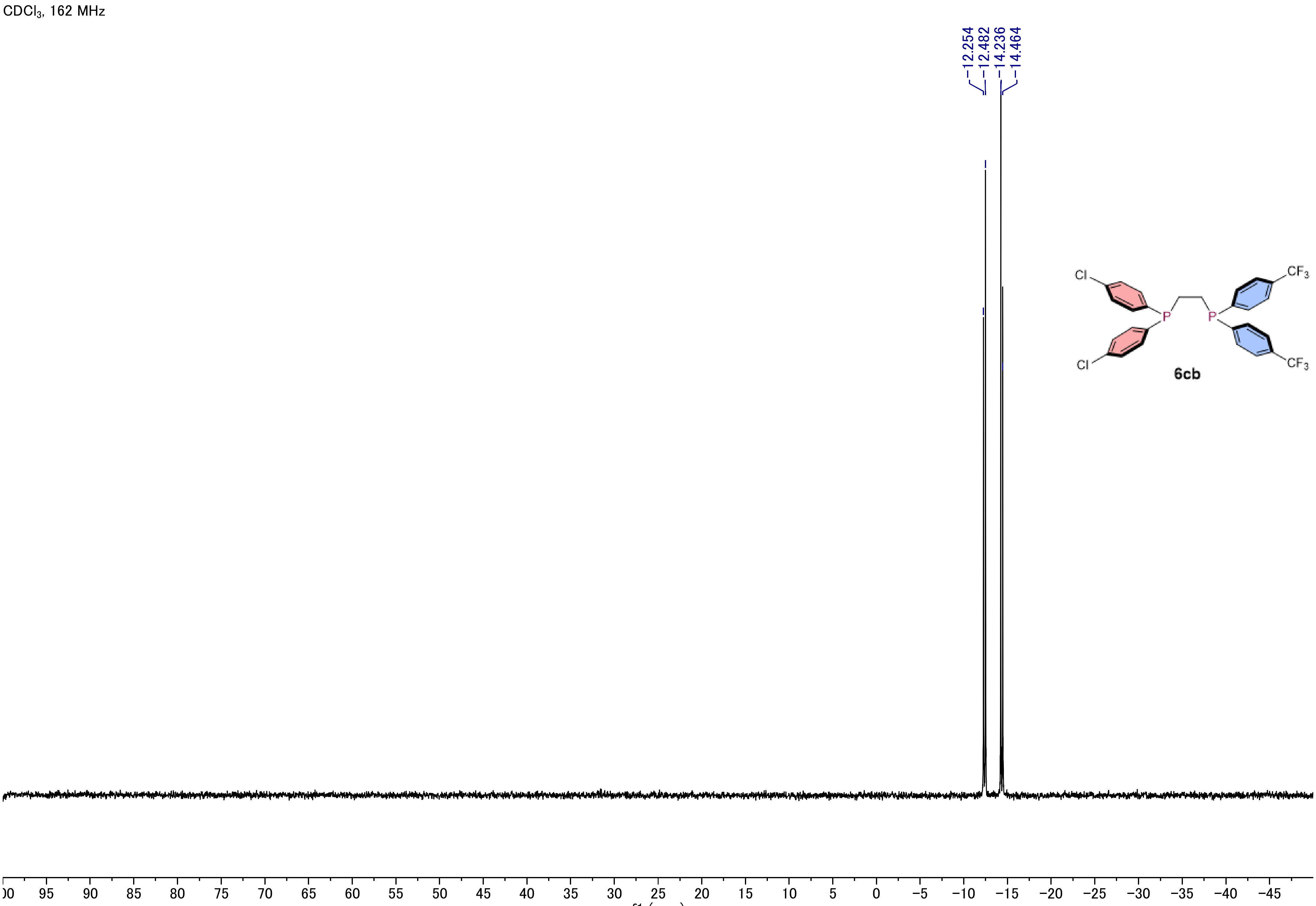

Supplementary Figure 184. ^{31}P NMR spectrum (162 MHz, $CDCl_3$) of **6bc**

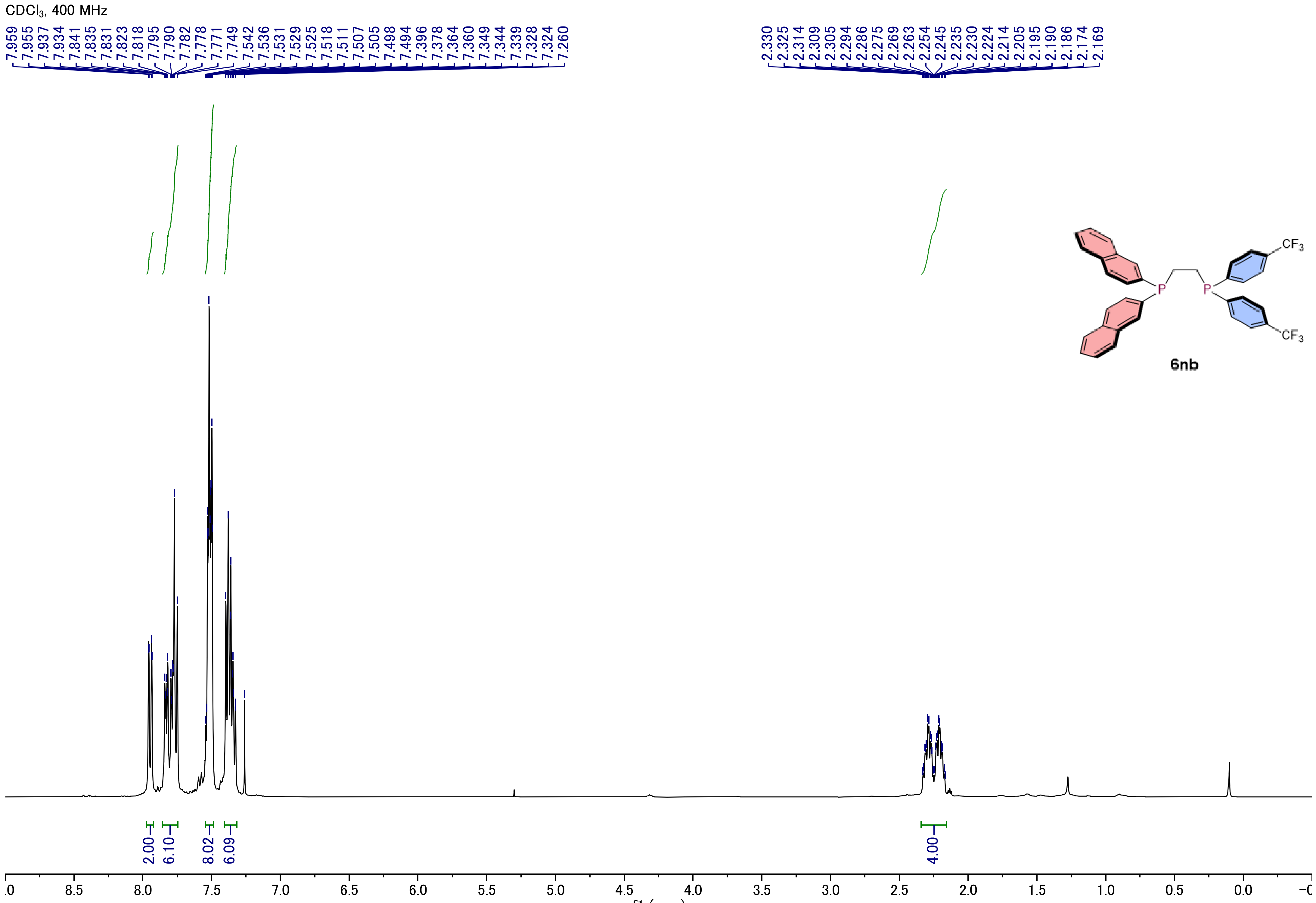

Supplementary Figure 185. ^{1}H NMR spectrum (400 MHz, $CDCl_3$) of **6nb**

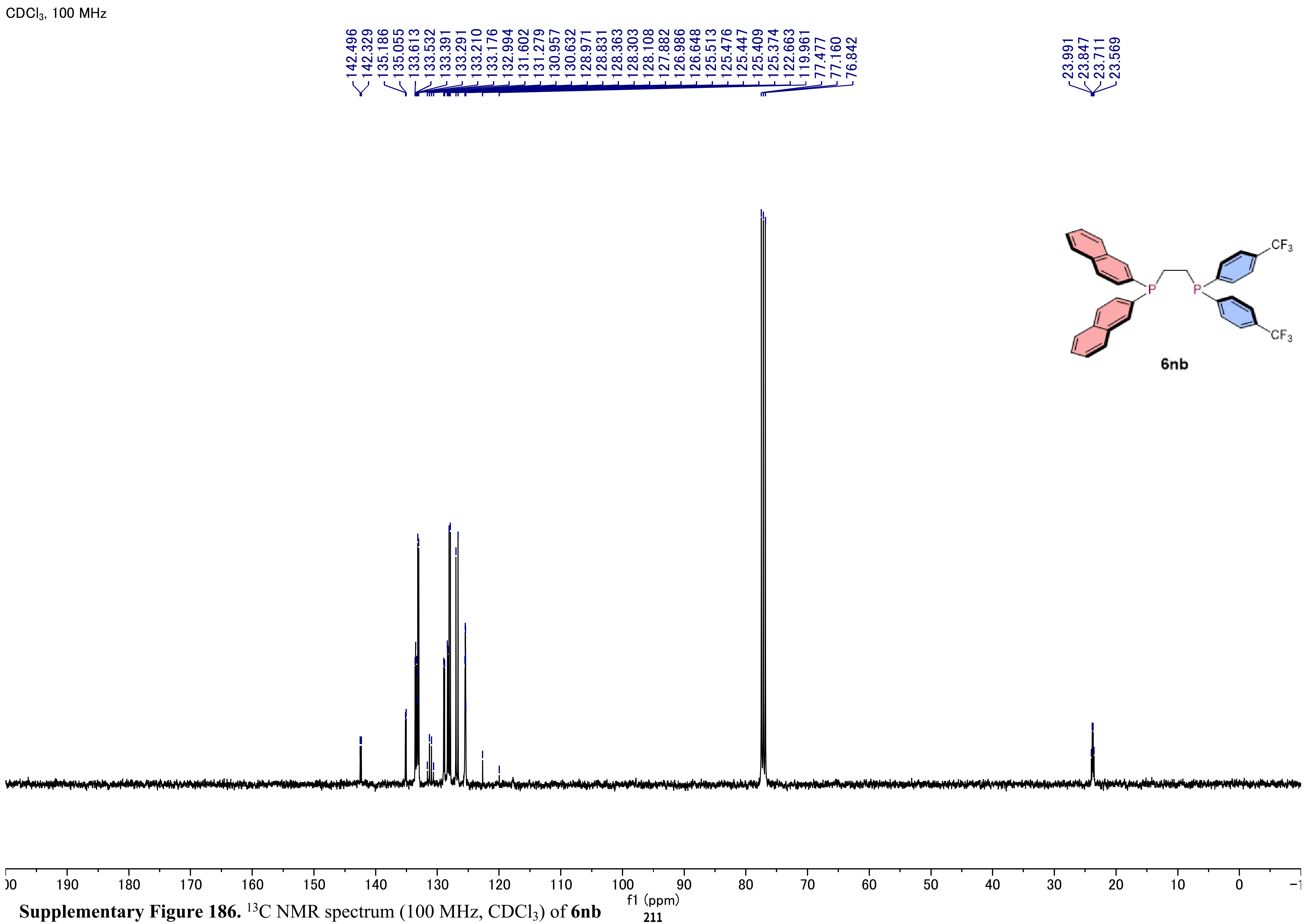

Supplementary Figure 186. ^{13}C NMR spectrum (100 MHz, $CDCl_3$) of **6nb**

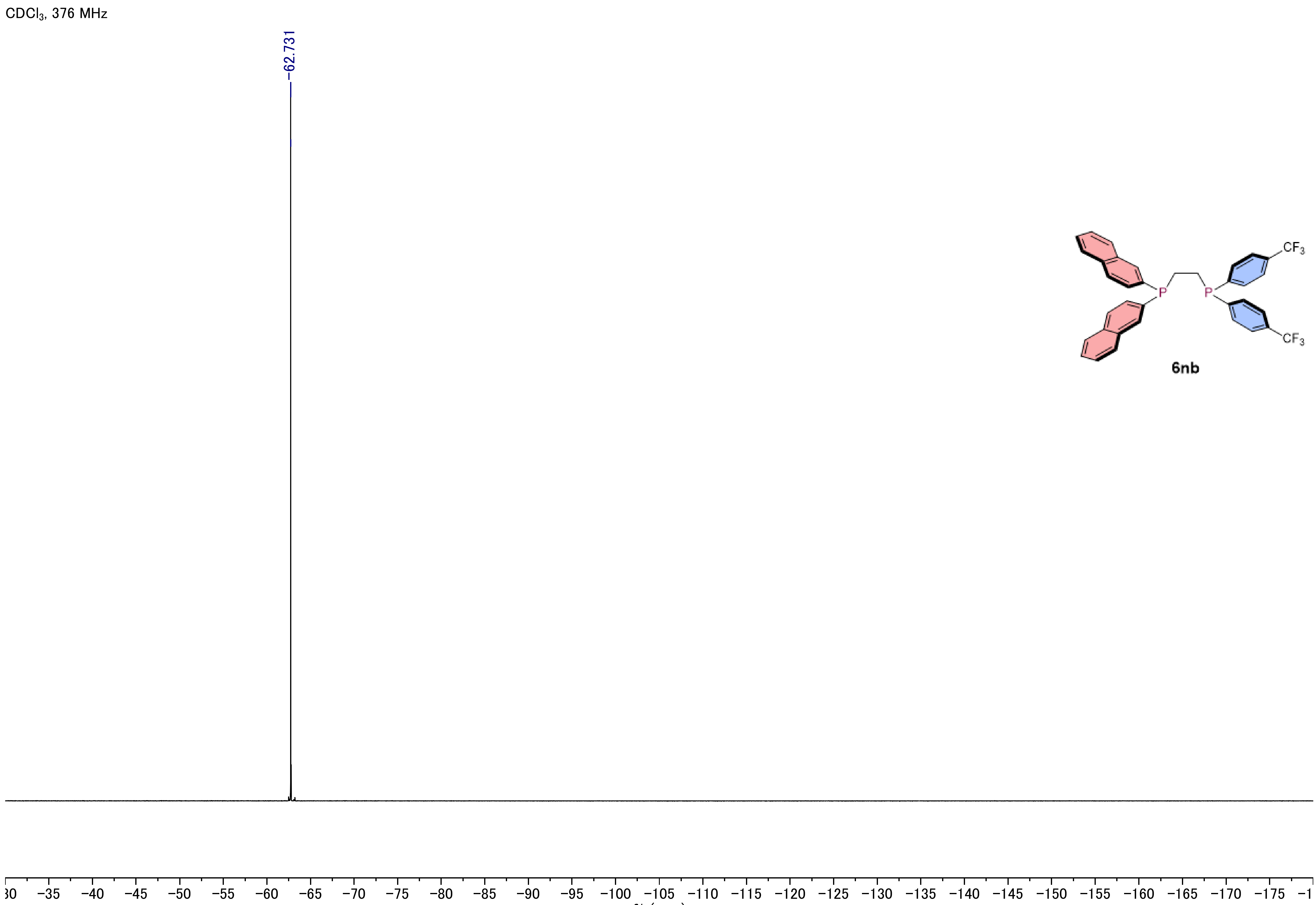

Supplementary Figure 187. ^{19}F NMR spectrum (376 MHz, $CDCl_3$) of **6nb**

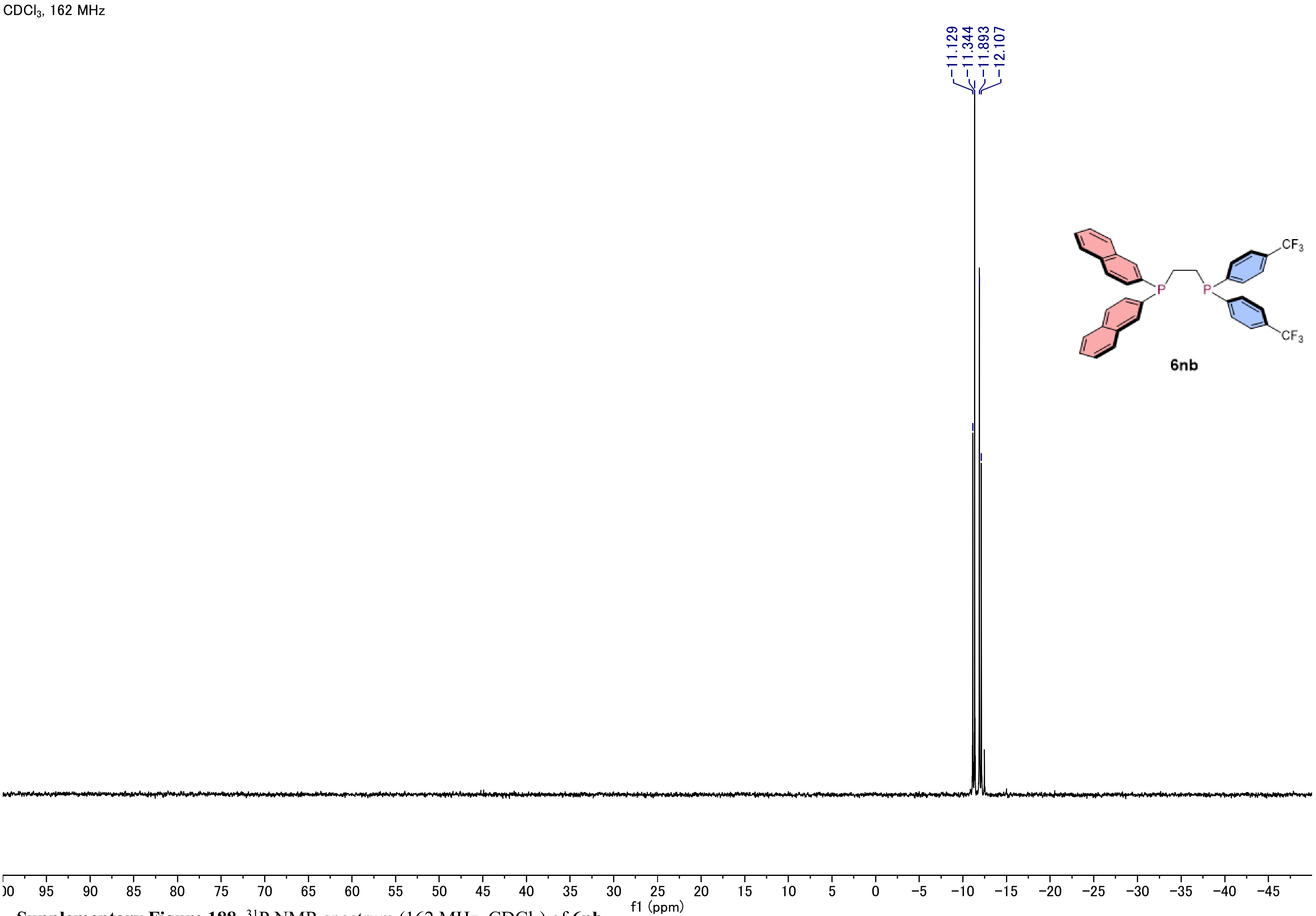

Supplementary Figure 188. ^{31}P NMR spectrum (162 MHz, $CDCl_3$) of **6nb**

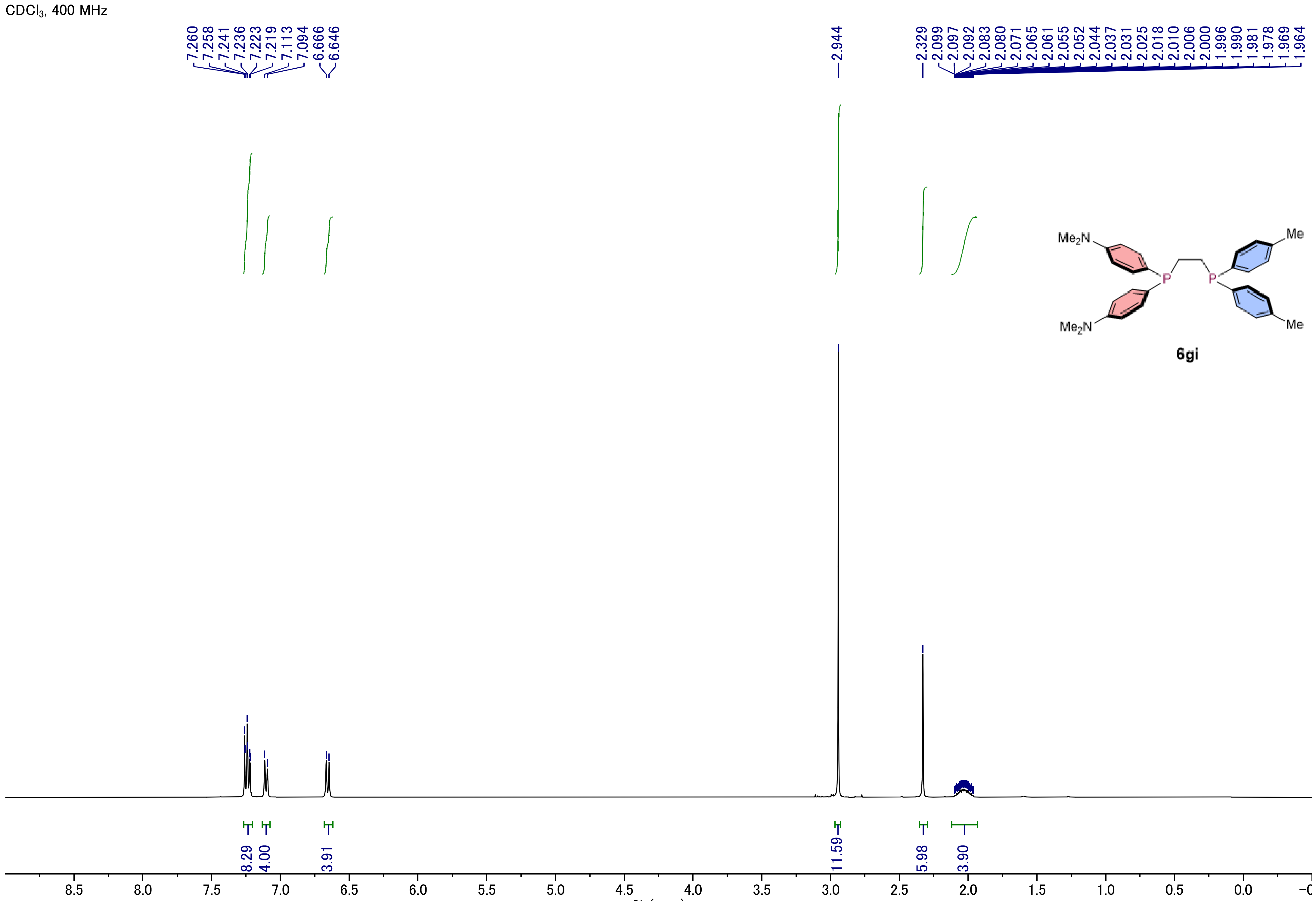

Supplementary Figure 189. ^{1}H NMR spectrum (400 MHz, $CDCl_3$) of **6gi**

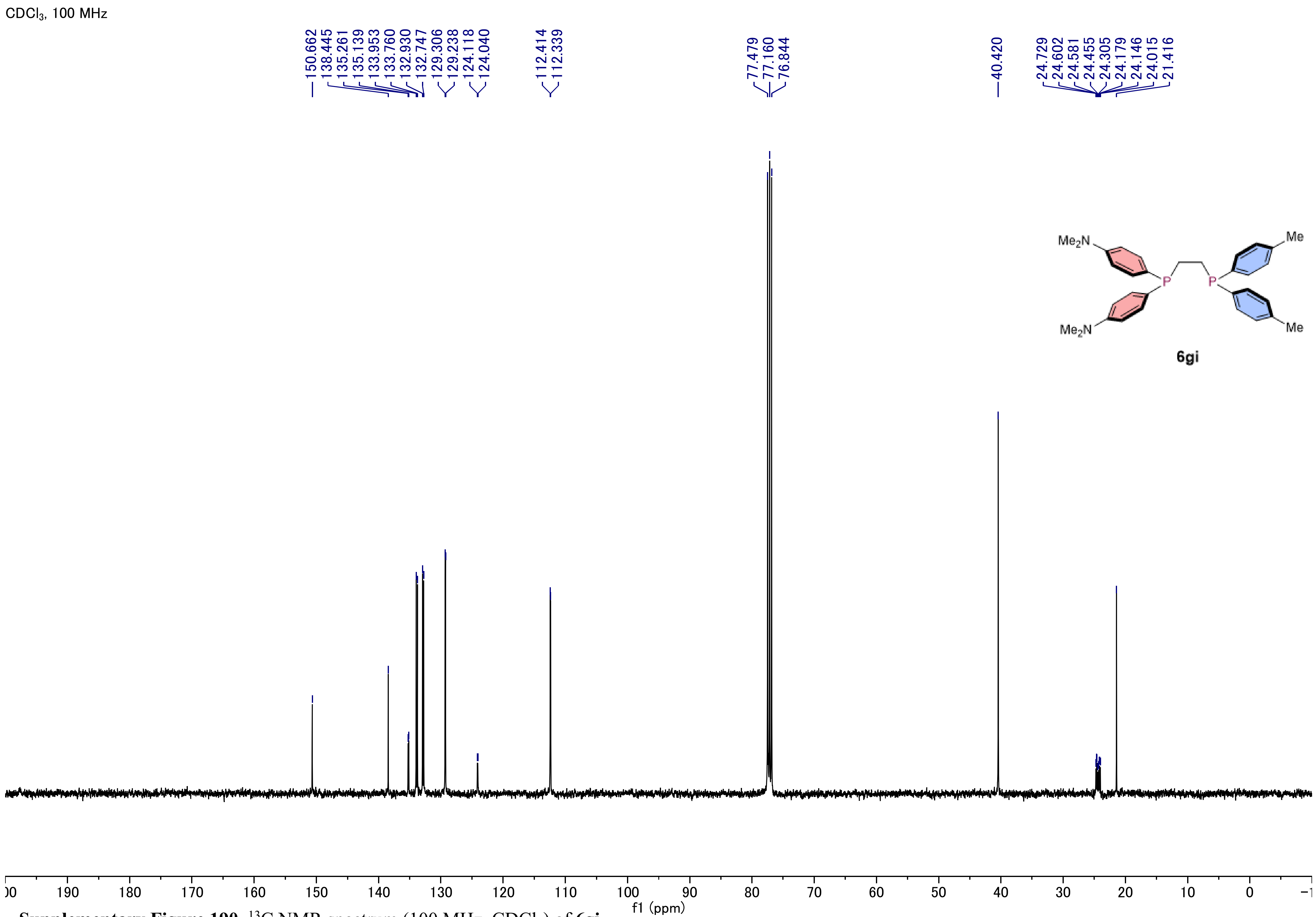

Supplementary Figure 190. ^{13}C NMR spectrum (100 MHz, CDCl$_3$) of **6gi**

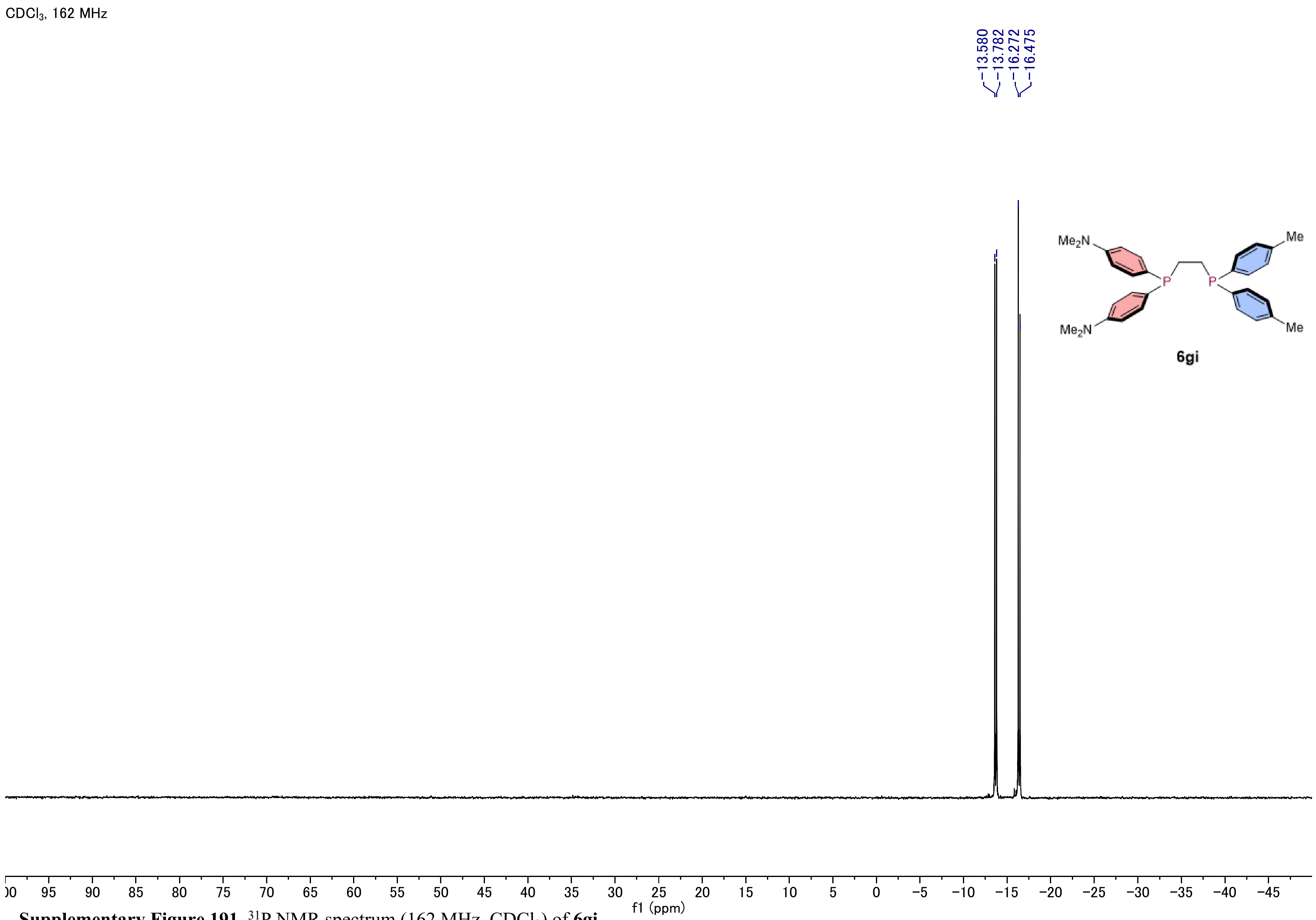

Supplementary Figure 191. ^{31}P NMR spectrum (162 MHz, $CDCl_3$) of **6gi**

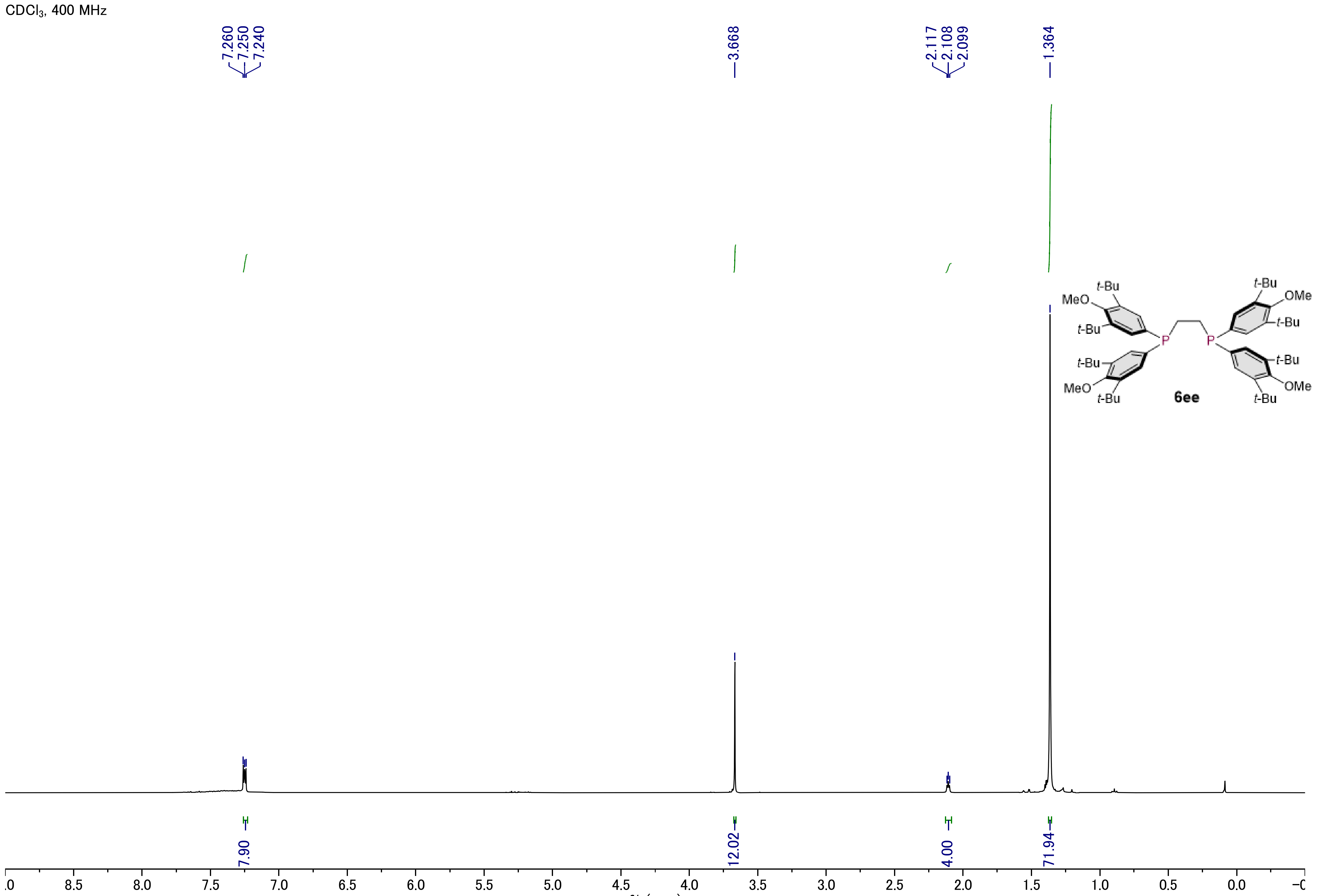

Supplementary Figure 192. ^{1}H NMR spectrum (400 MHz, $CDCl_3$) of **6ee**

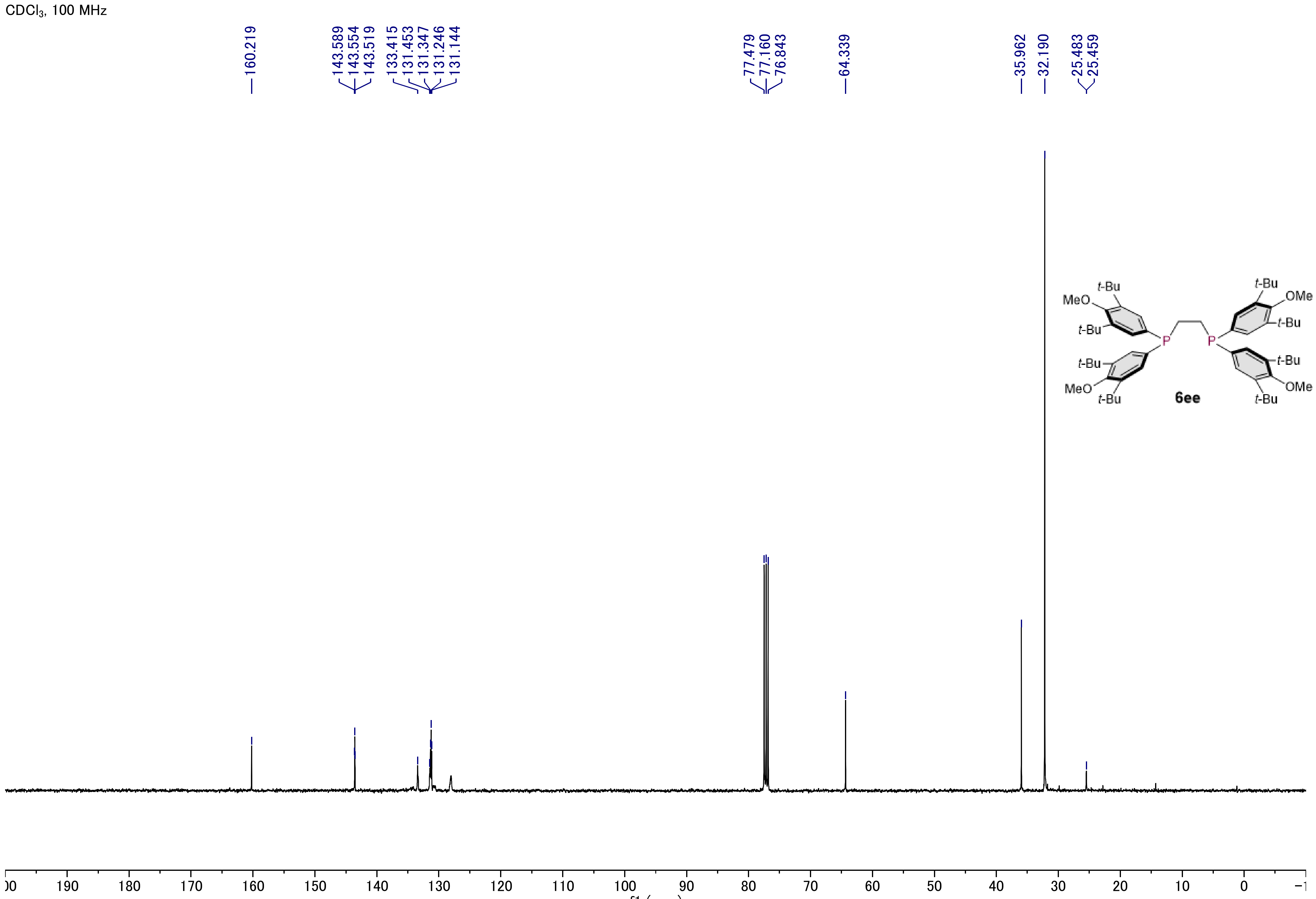

Supplementary Figure 193. ^{13}C NMR spectrum (100 MHz, $CDCl_3$) of **6ee**

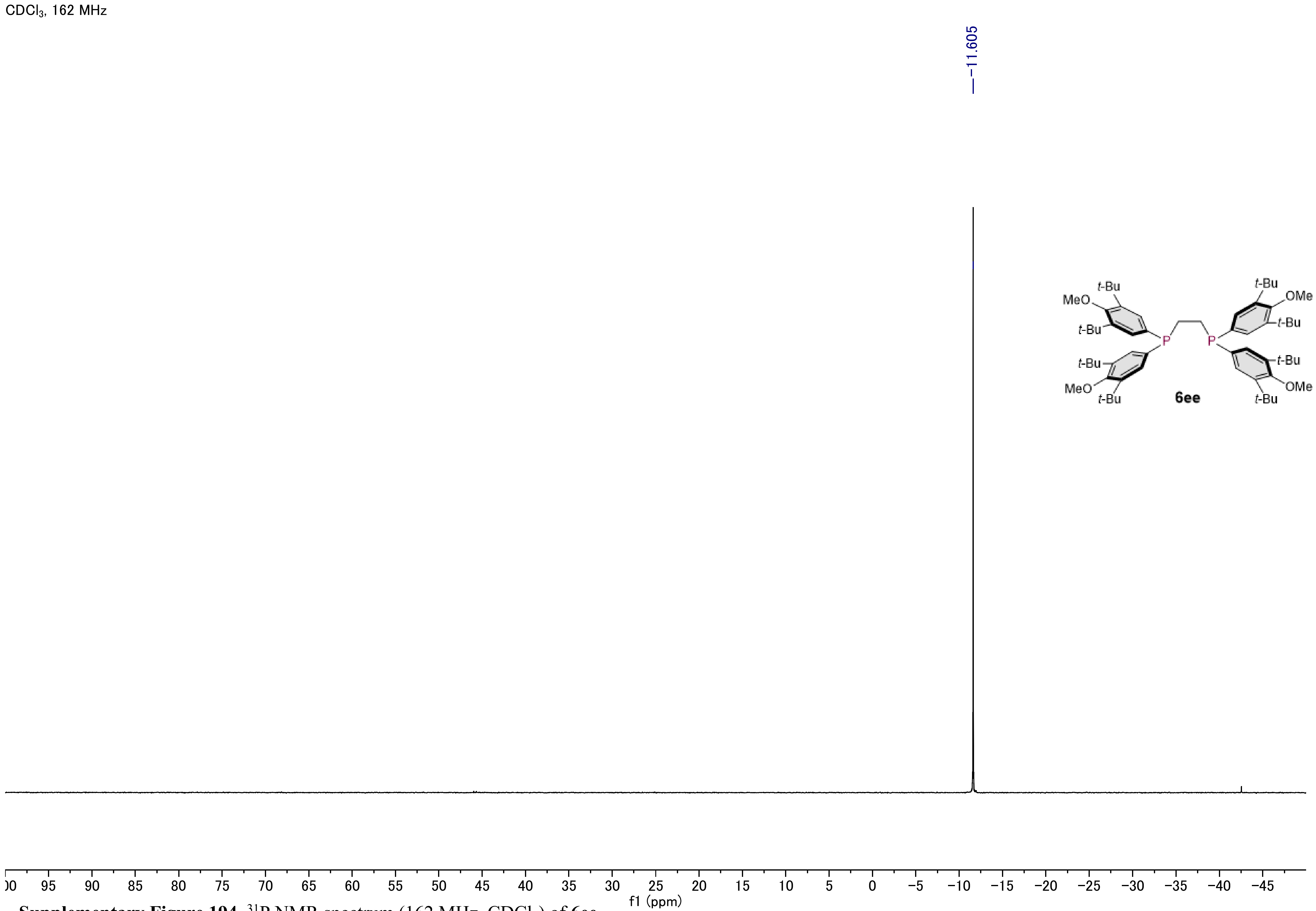

Supplementary Figure 194. ^{31}P NMR spectrum (162 MHz, $CDCl_3$) of **6ee**

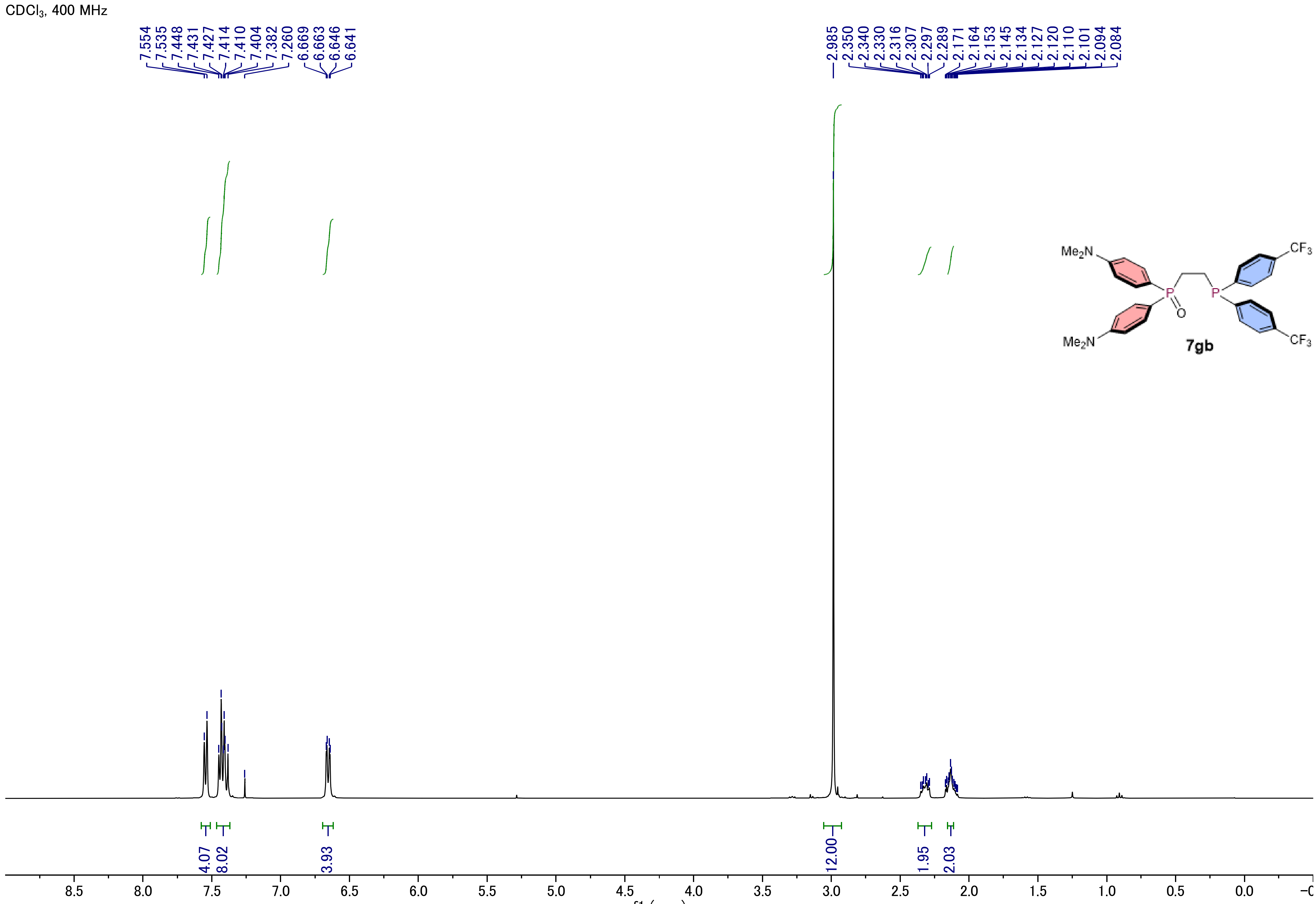

Supplementary Figure 195. ^{1}H NMR spectrum (400 MHz, $CDCl_3$) of **7gb**

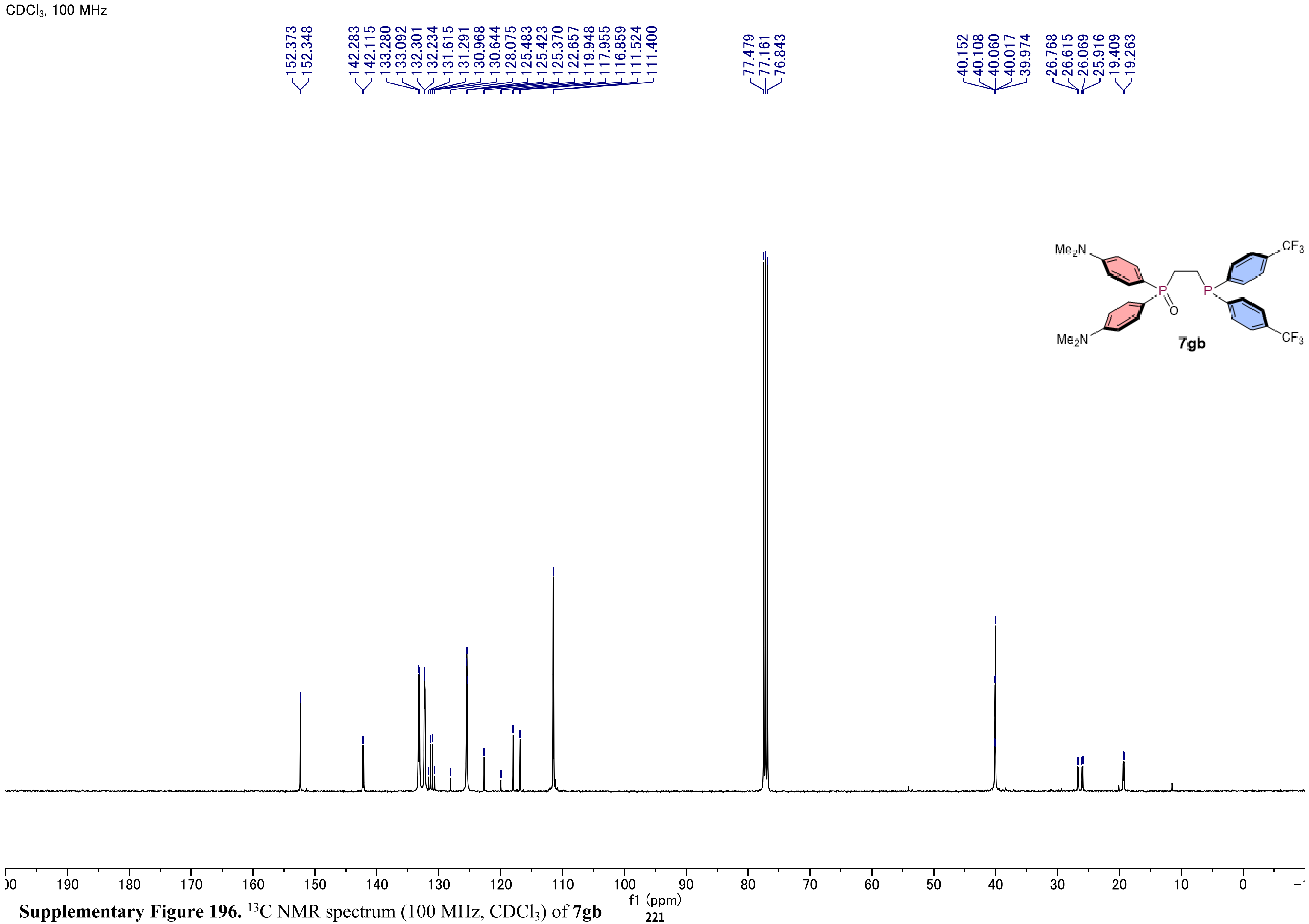

Supplementary Figure 196. ^{13}C NMR spectrum (100 MHz, $CDCl_3$) of **7gb**

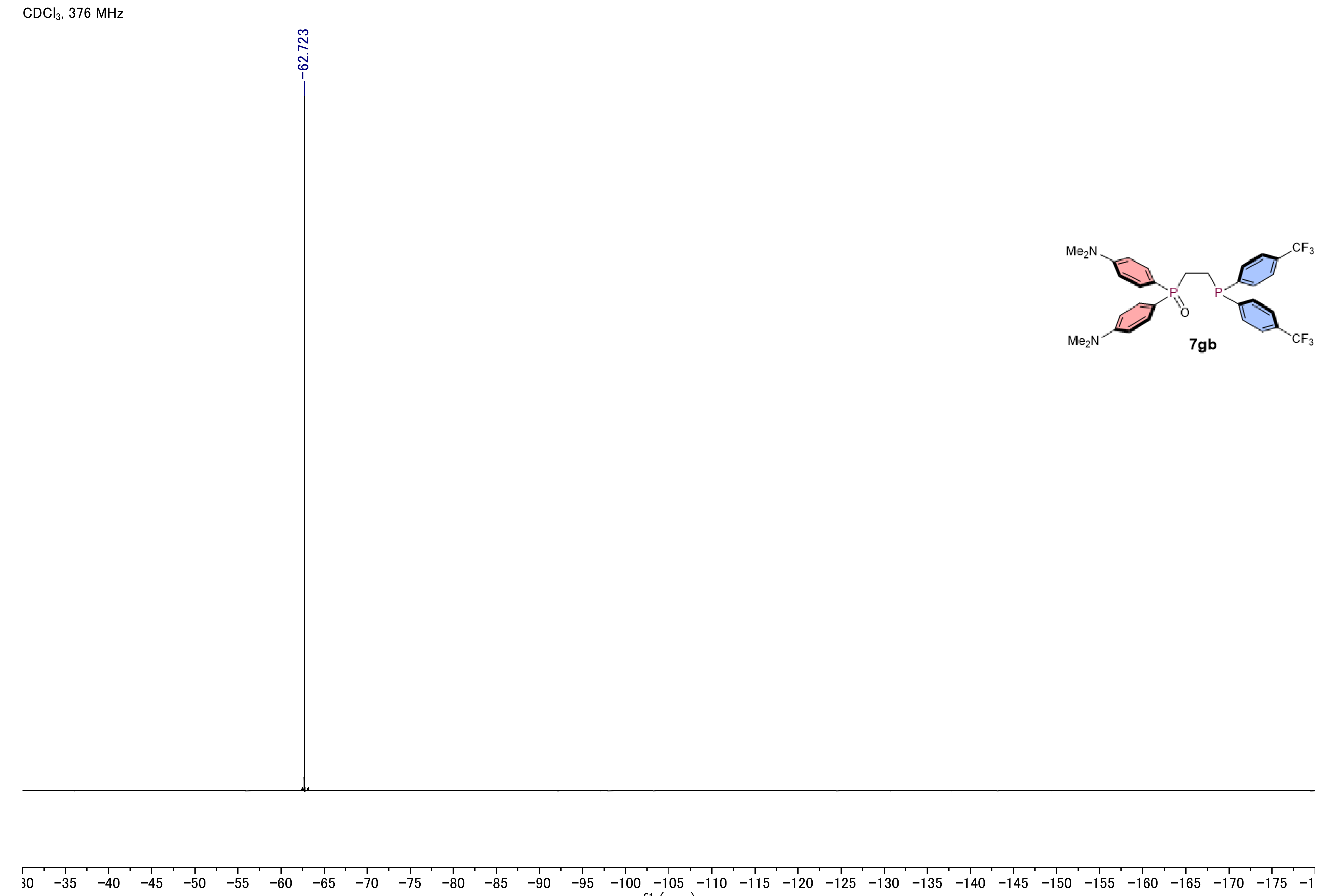

Supplementary Figure 197. ^{19}F NMR spectrum (376 MHz, $CDCl_3$) of **7gb**

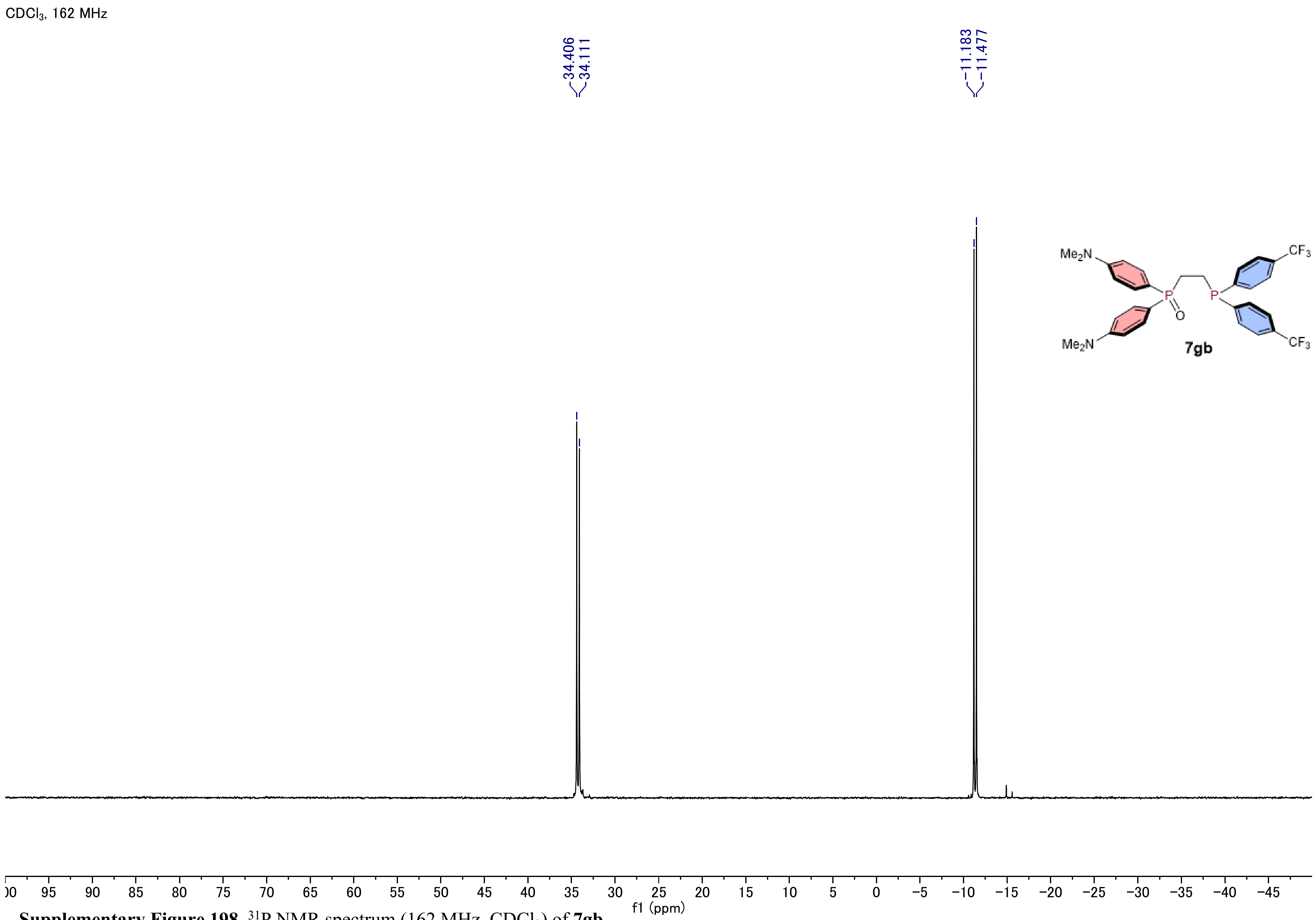

Supplementary Figure 198. ^{31}P NMR spectrum (162 MHz, $CDCl_3$) of **7gb**

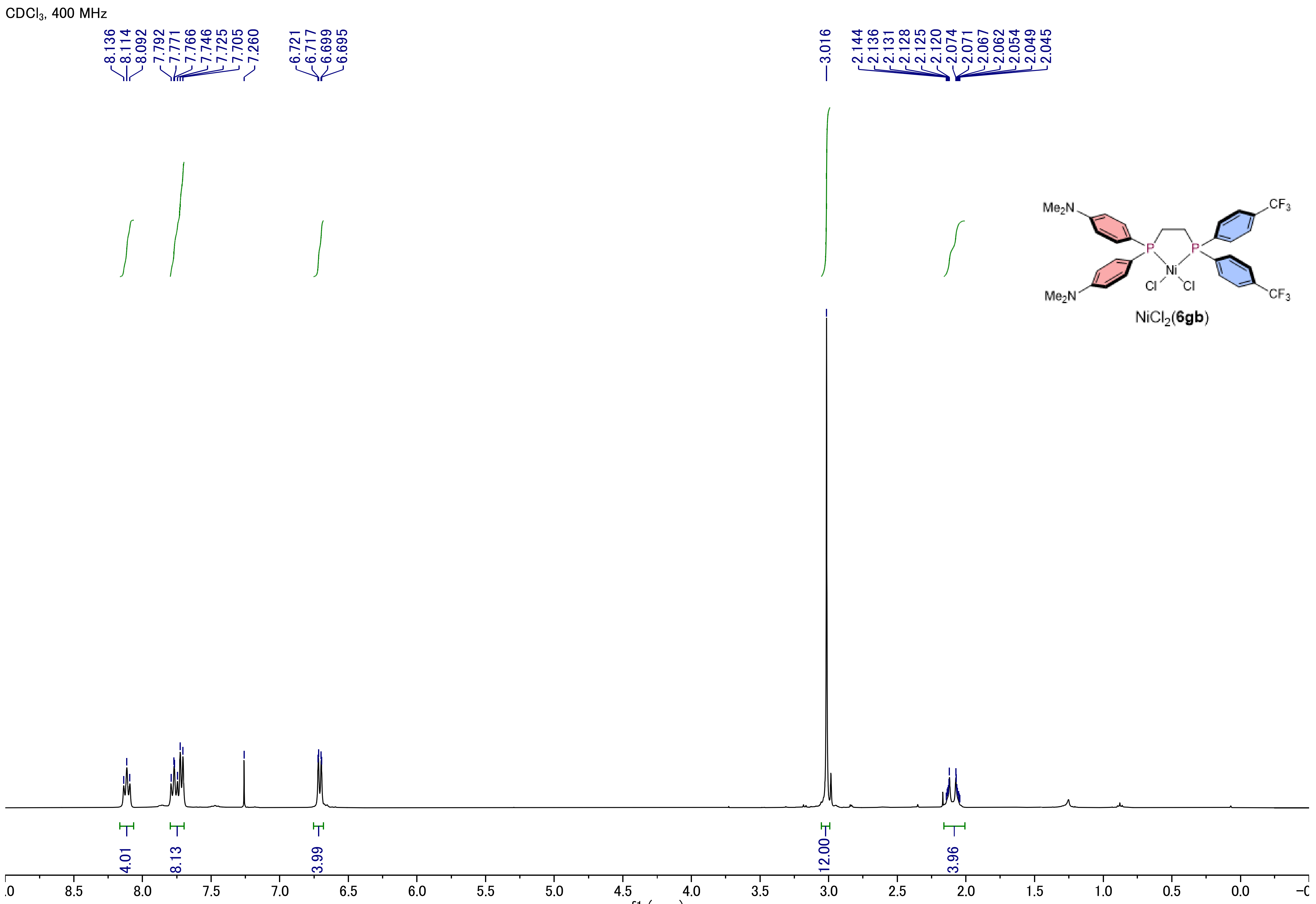

Supplementary Figure 199. ^{1}H NMR spectrum (400 MHz, $CDCl_3$) of $NiCl_2$(**6gb**)

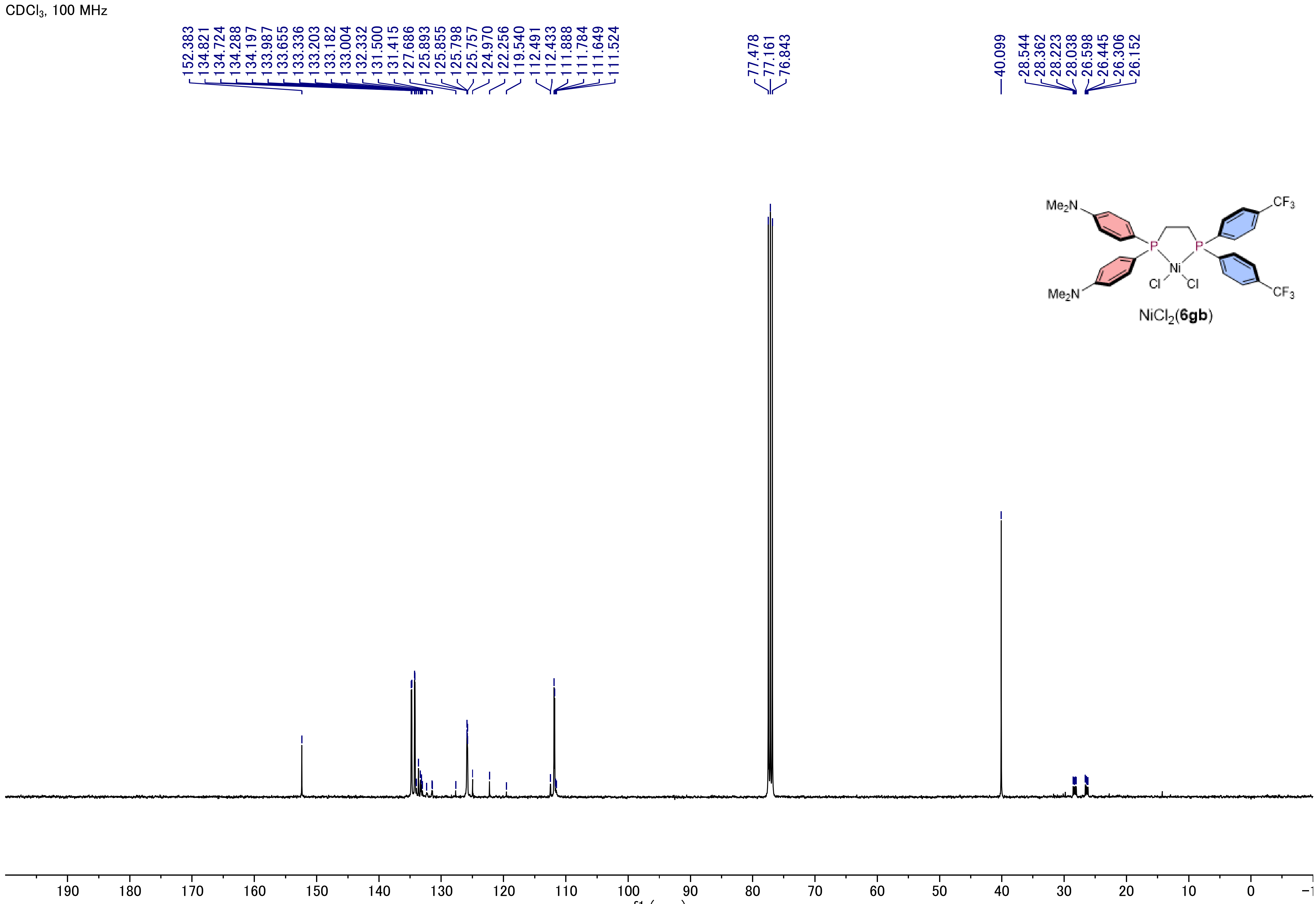

Supplementary Figure 200. ^{13}C NMR spectrum (100 MHz, $CDCl_3$) of $NiCl_2$(**6gb**)

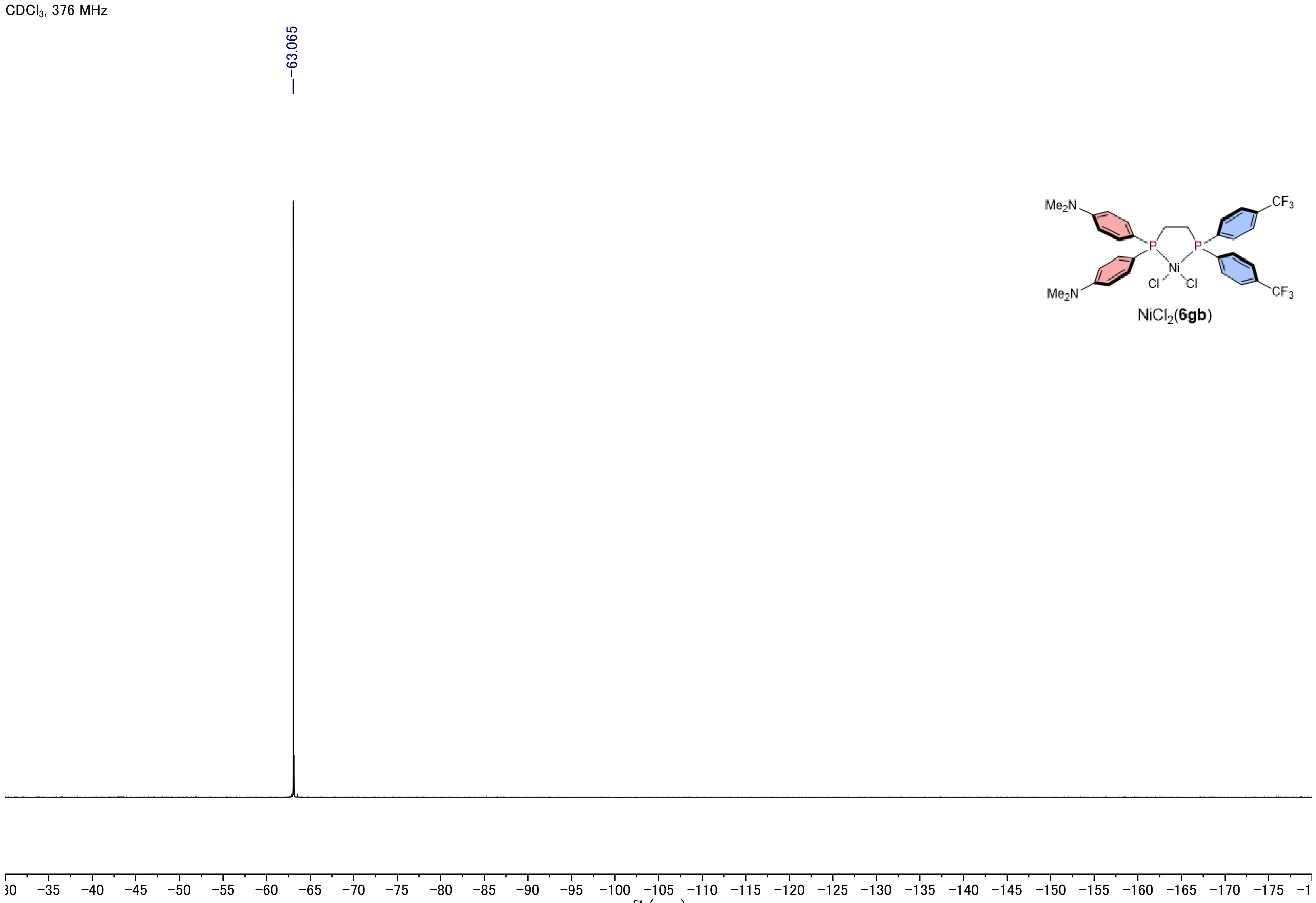

Supplementary Figure 201. ^{19}F NMR spectrum (376 MHz, $CDCl_3$) of $NiCl_2$(**6gb**)

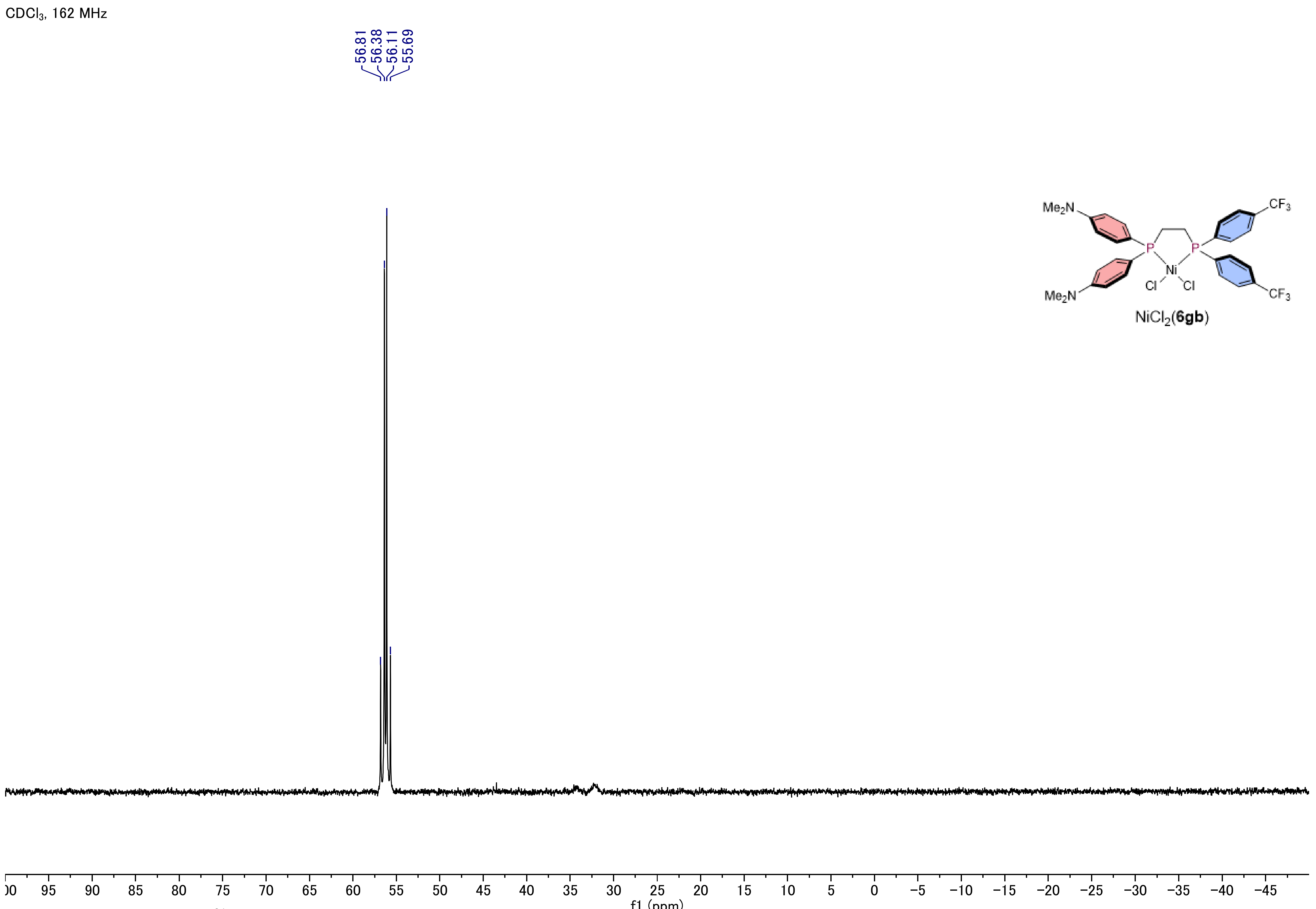

Supplementary Figure 202. ^{31}P NMR spectrum (162 MHz, $CDCl_3$) of $NiCl_2$(**6gb**)

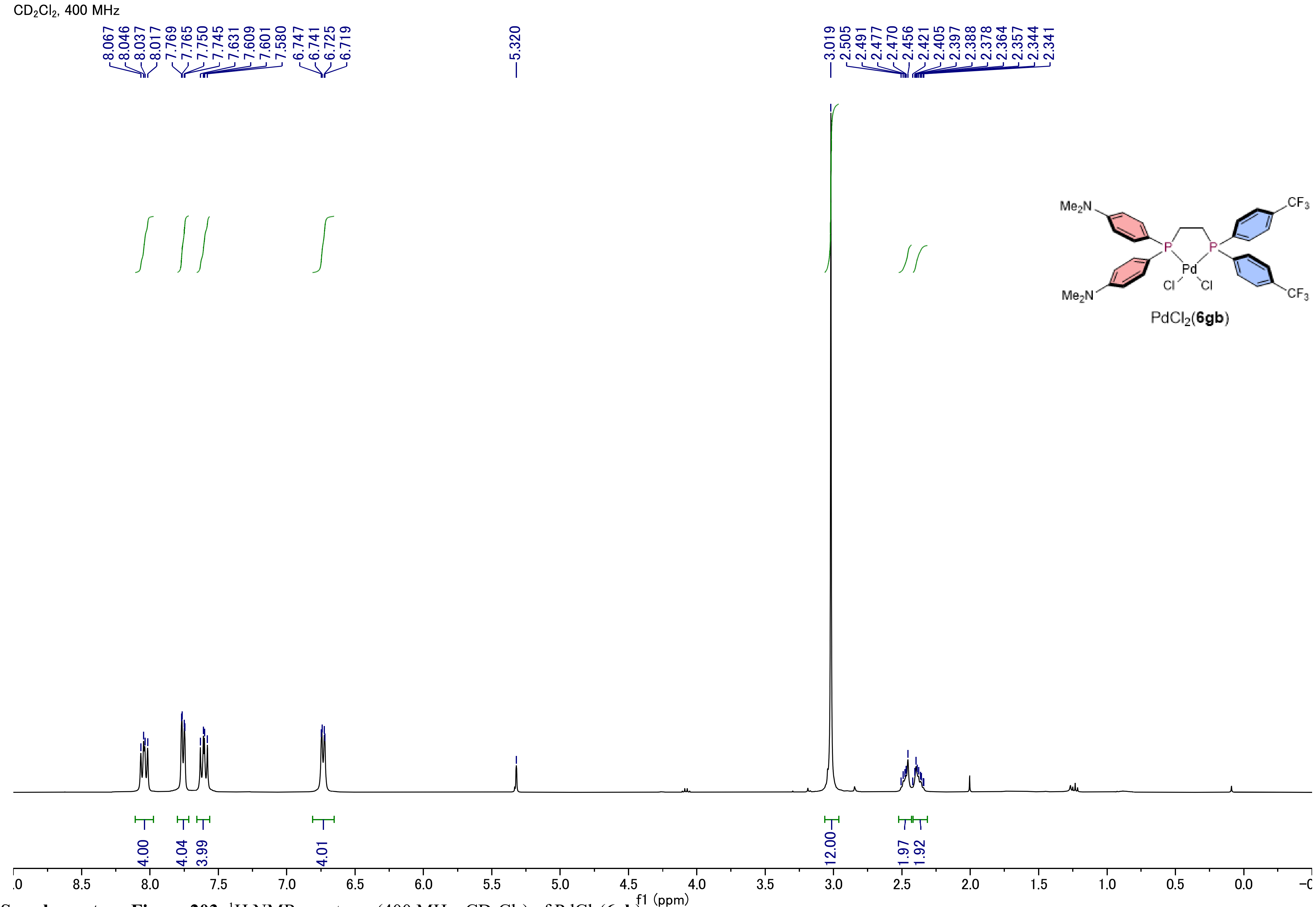

Supplementary Figure 203. ^{1}H NMR spectrum (400 MHz, CD_2Cl_2) of $PdCl_2$(**6gb**)

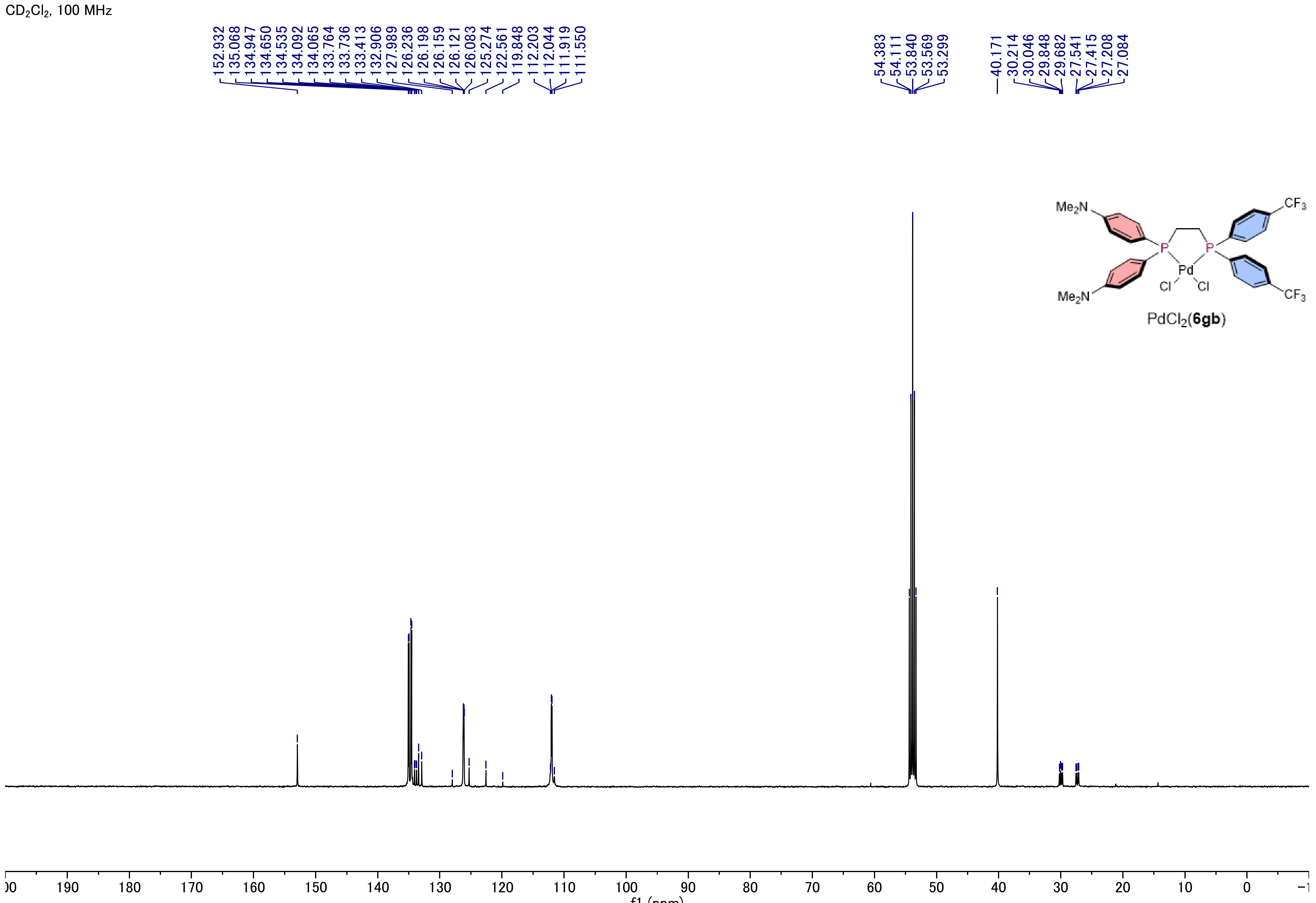

Supplementary Figure 204. ^{13}C NMR spectrum (100 MHz, CD_2Cl_2) of $PdCl_2$(**6gb**)

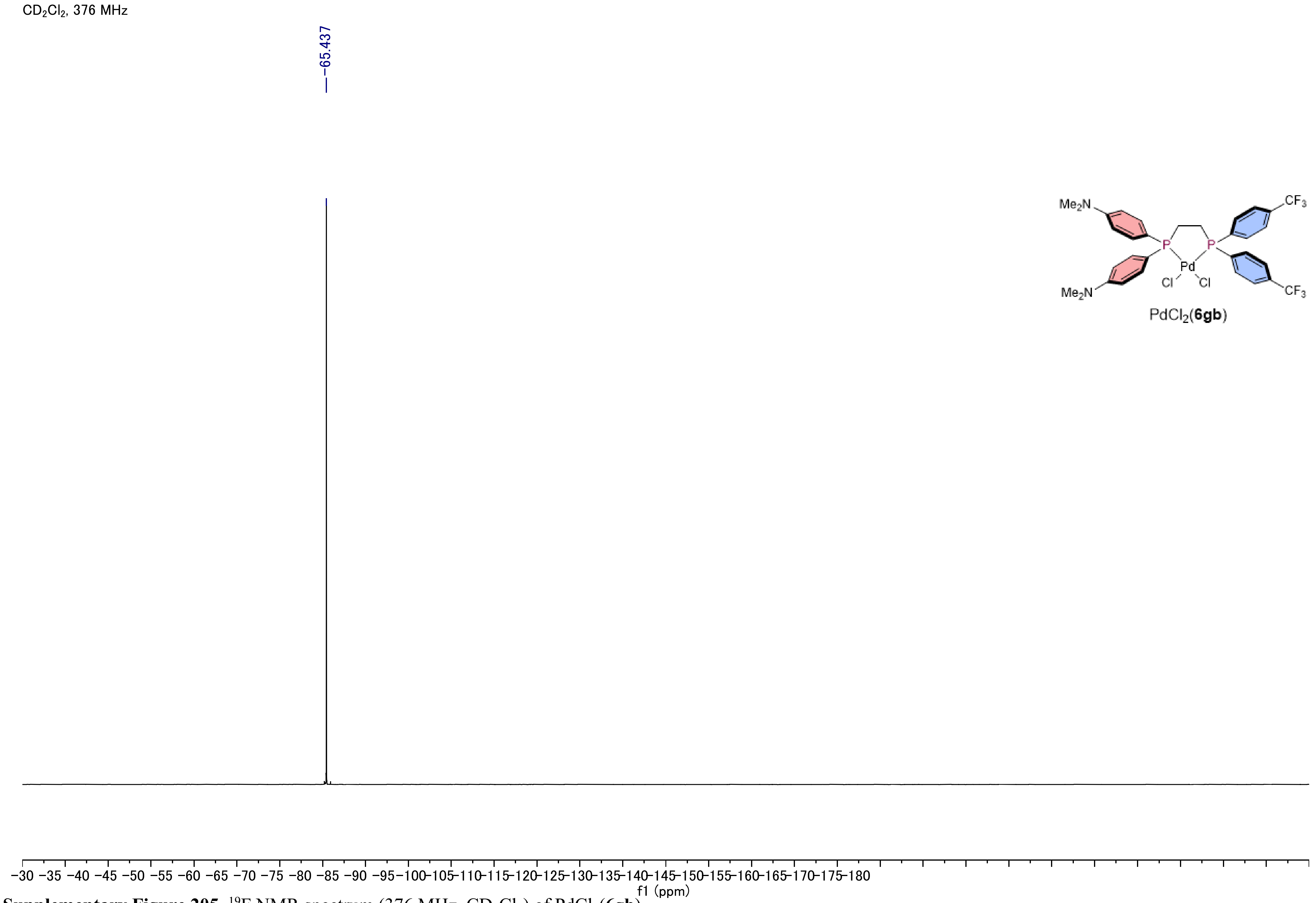

Supplementary Figure 205. ^{19}F NMR spectrum (376 MHz, CD_2Cl_2) of $PdCl_2$(**6gb**)

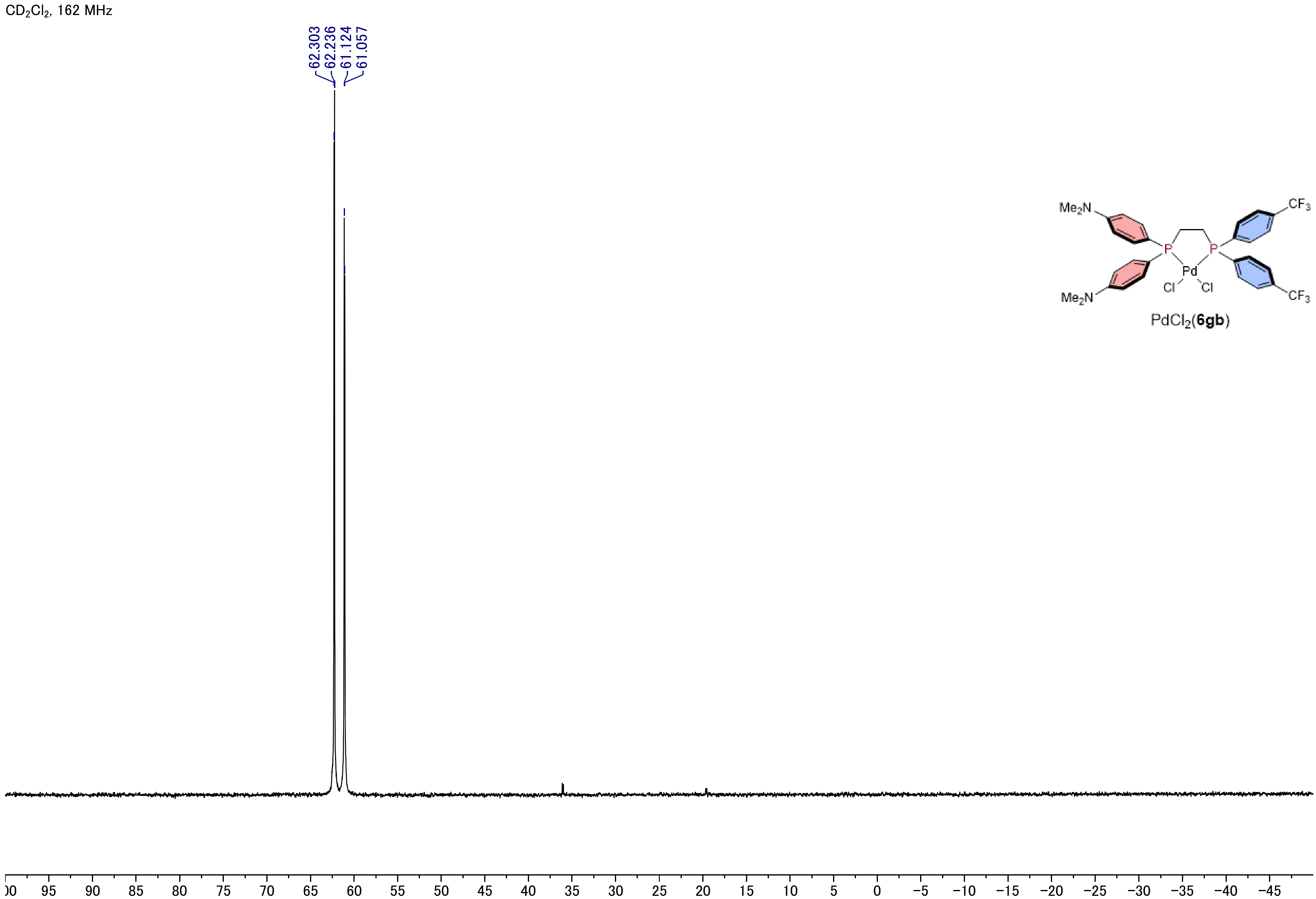

Supplementary Figure 206. ^{31}P NMR spectrum (162 MHz, CD_2Cl_2) of $PdCl_2$(**6gb**)

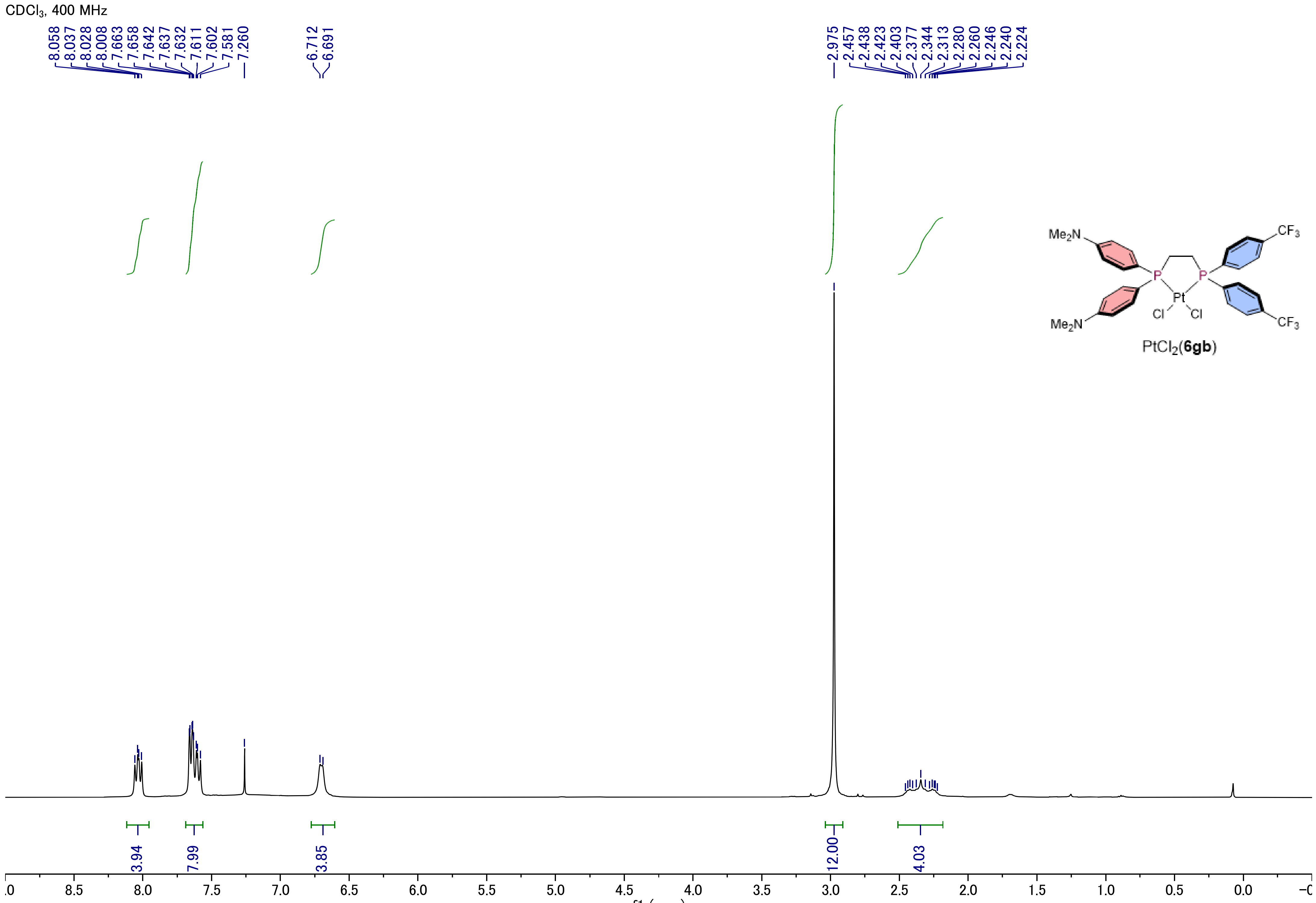

Supplementary Figure 207. ^1H NMR spectrum (400 MHz, $CDCl_3$) of $PtCl_2$(**6gb**)

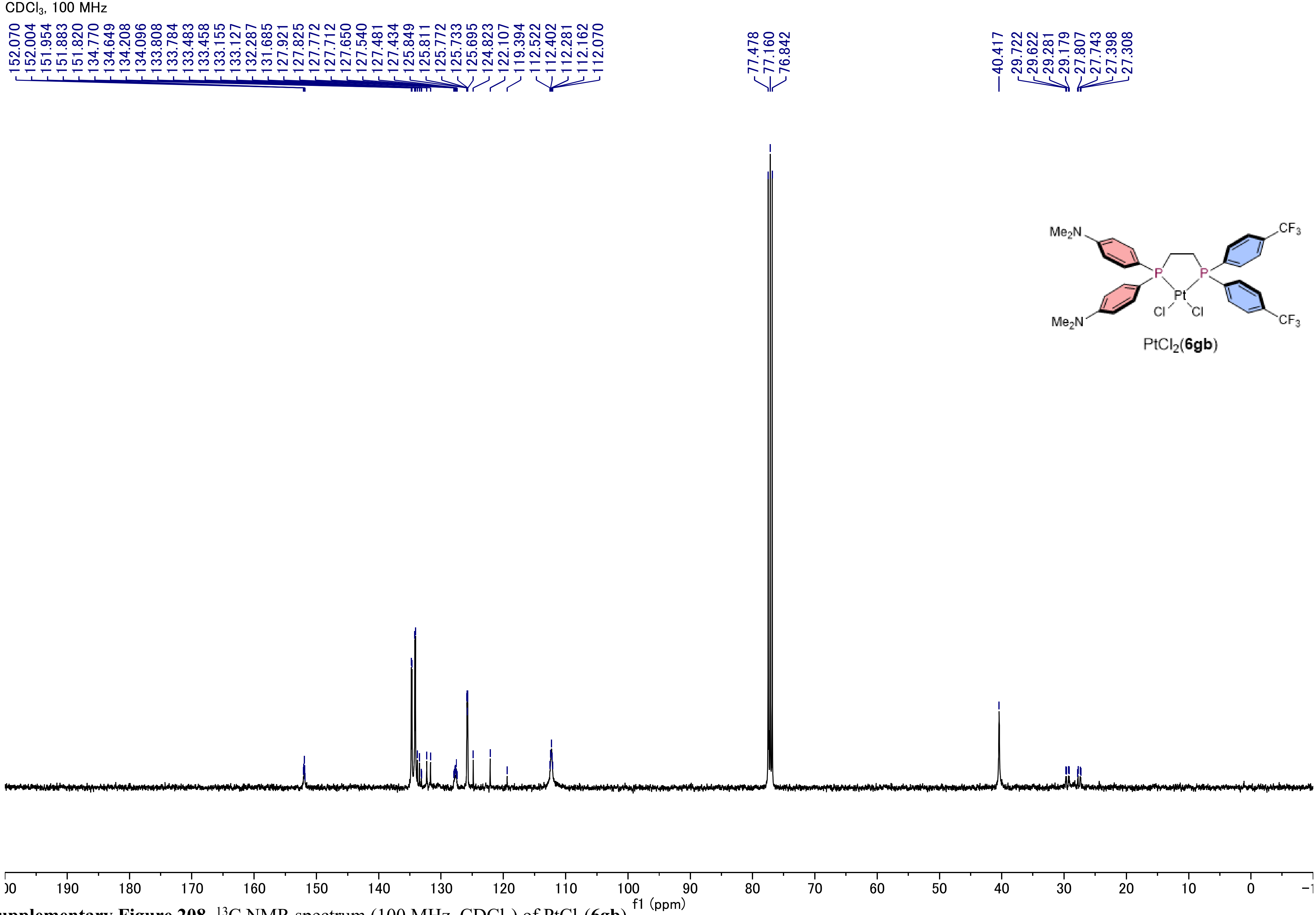

Supplementary Figure 208. ^{13}C NMR spectrum (100 MHz, $CDCl_3$) of $PtCl_2$(**6gb**)

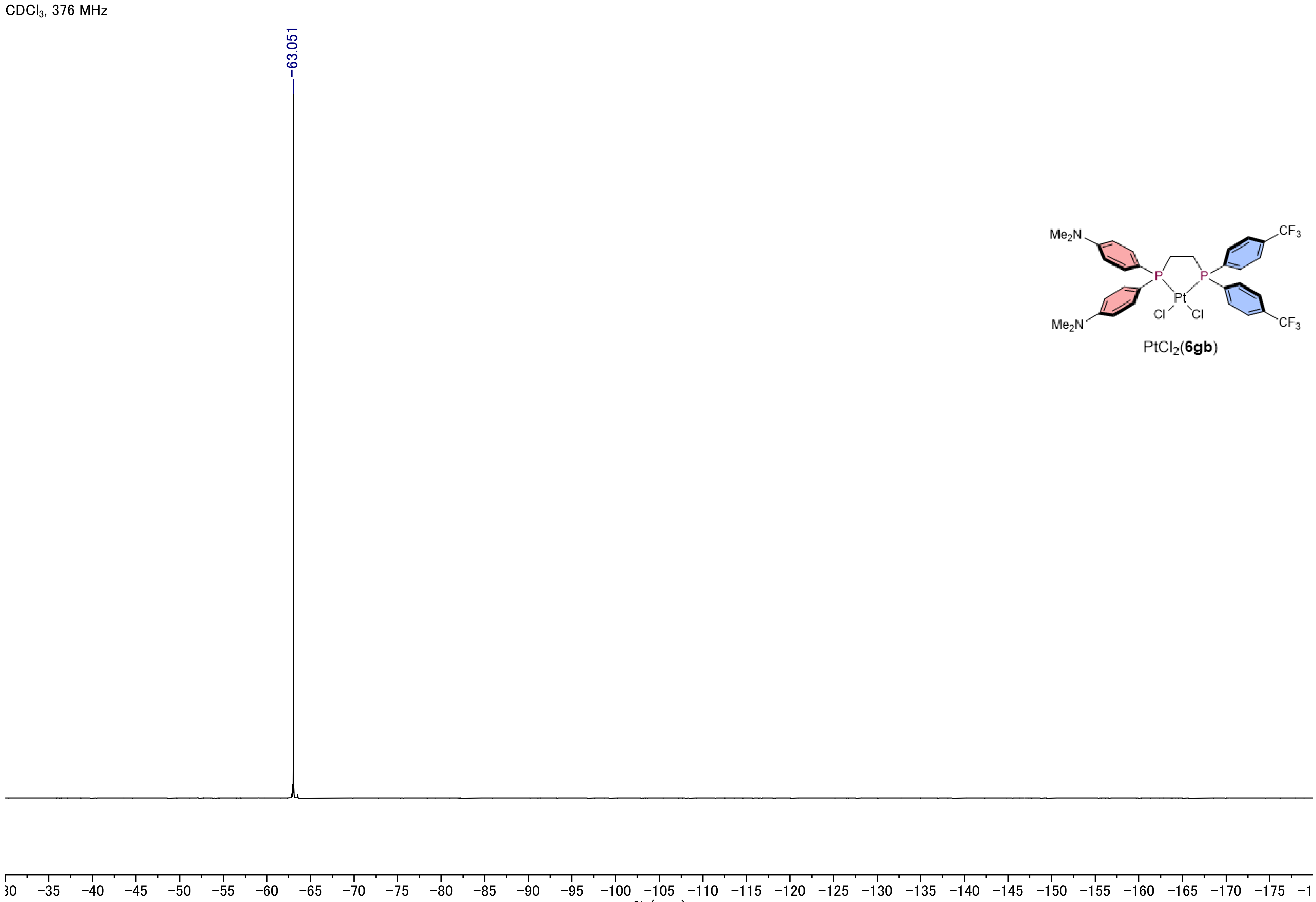

Supplementary Figure 209. ^{19}F NMR spectrum (376 MHz, $CDCl_3$) of $PtCl_2$(**6gb**)

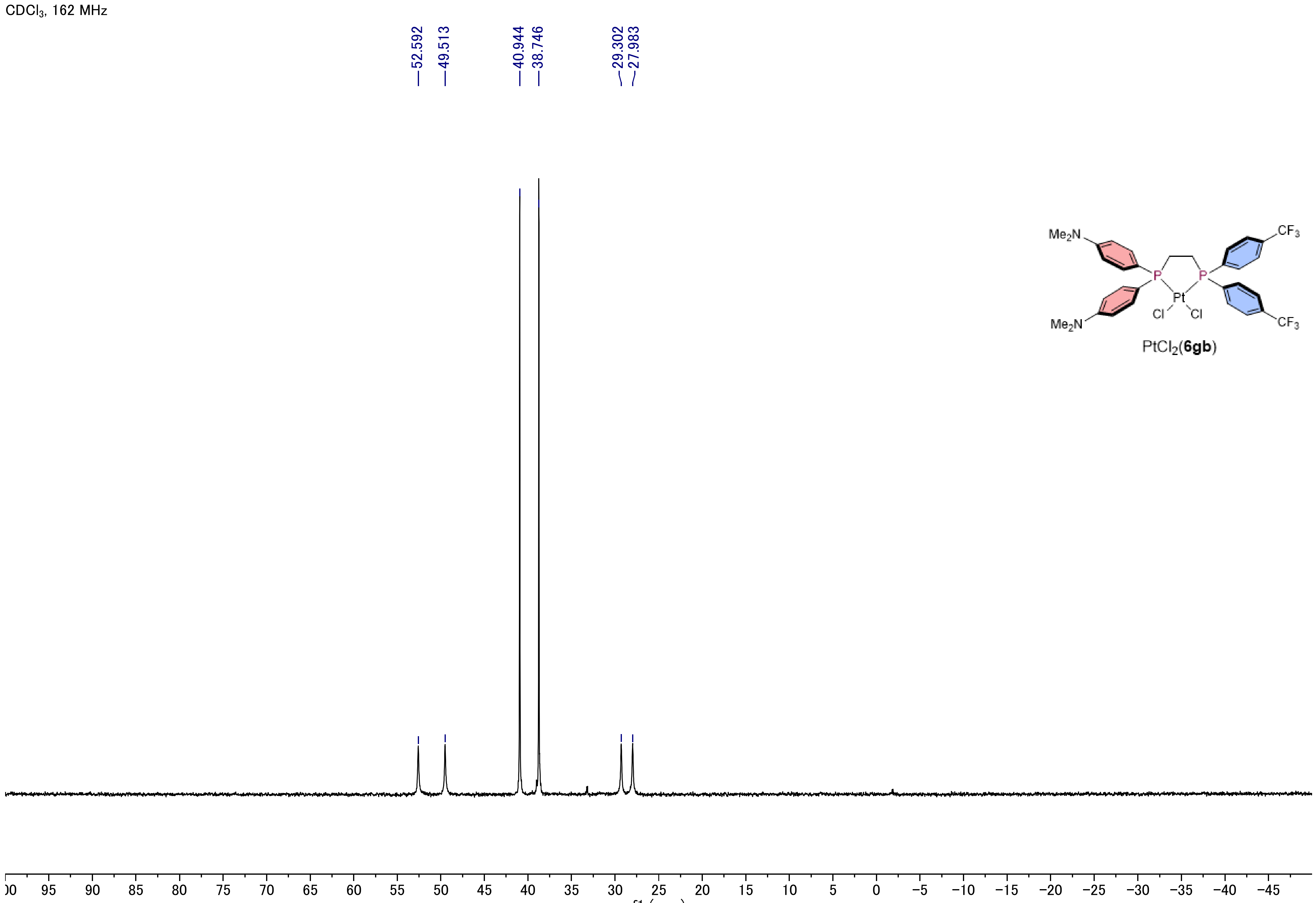

Supplementary Figure 210. ^{31}P NMR spectrum (162 MHz, $CDCl_3$) of $PtCl_2$(**6gb**)

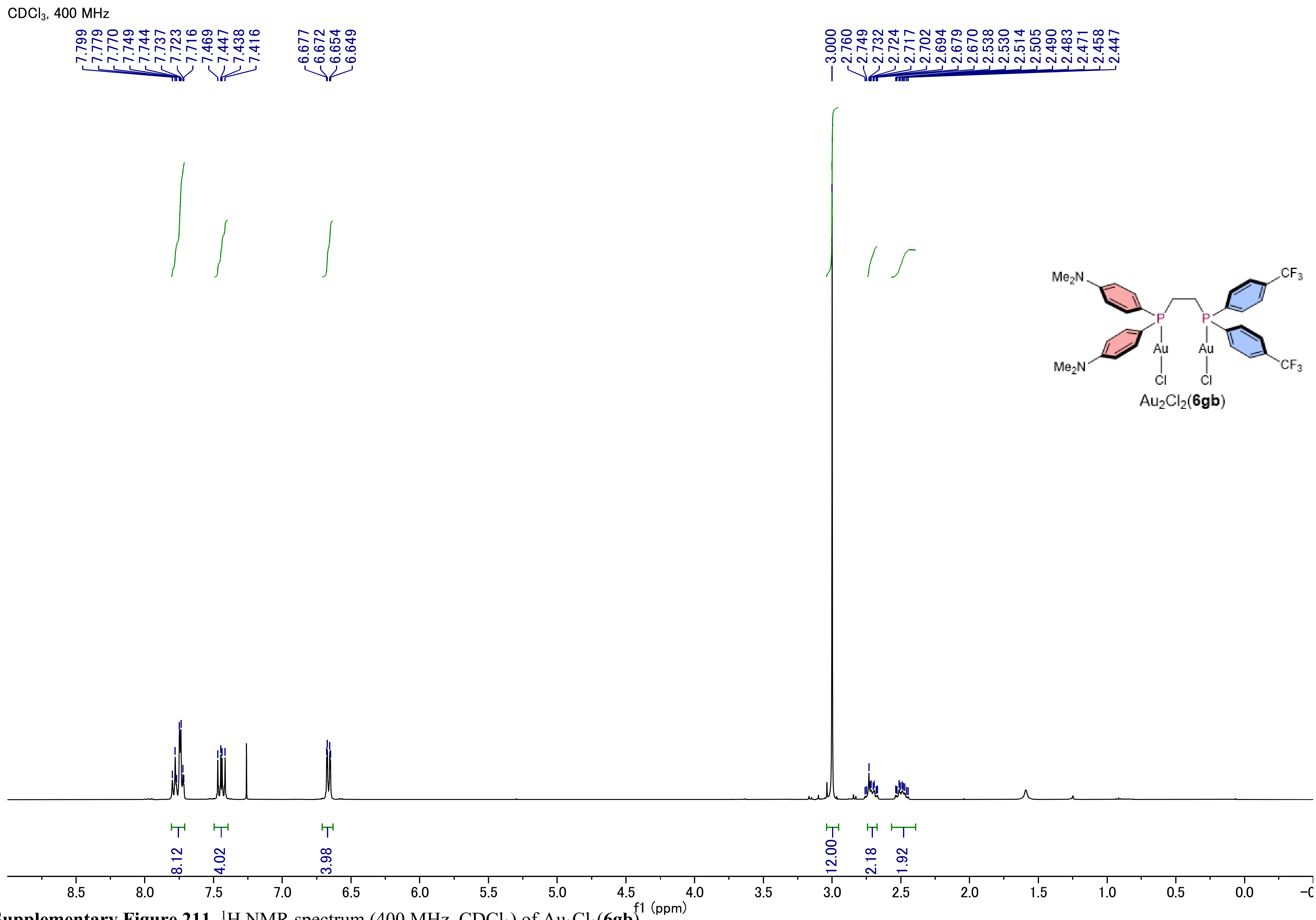

Supplementary Figure 211. ^{1}H NMR spectrum (400 MHz, $CDCl_3$) of Au_2Cl_2(**6gb**)

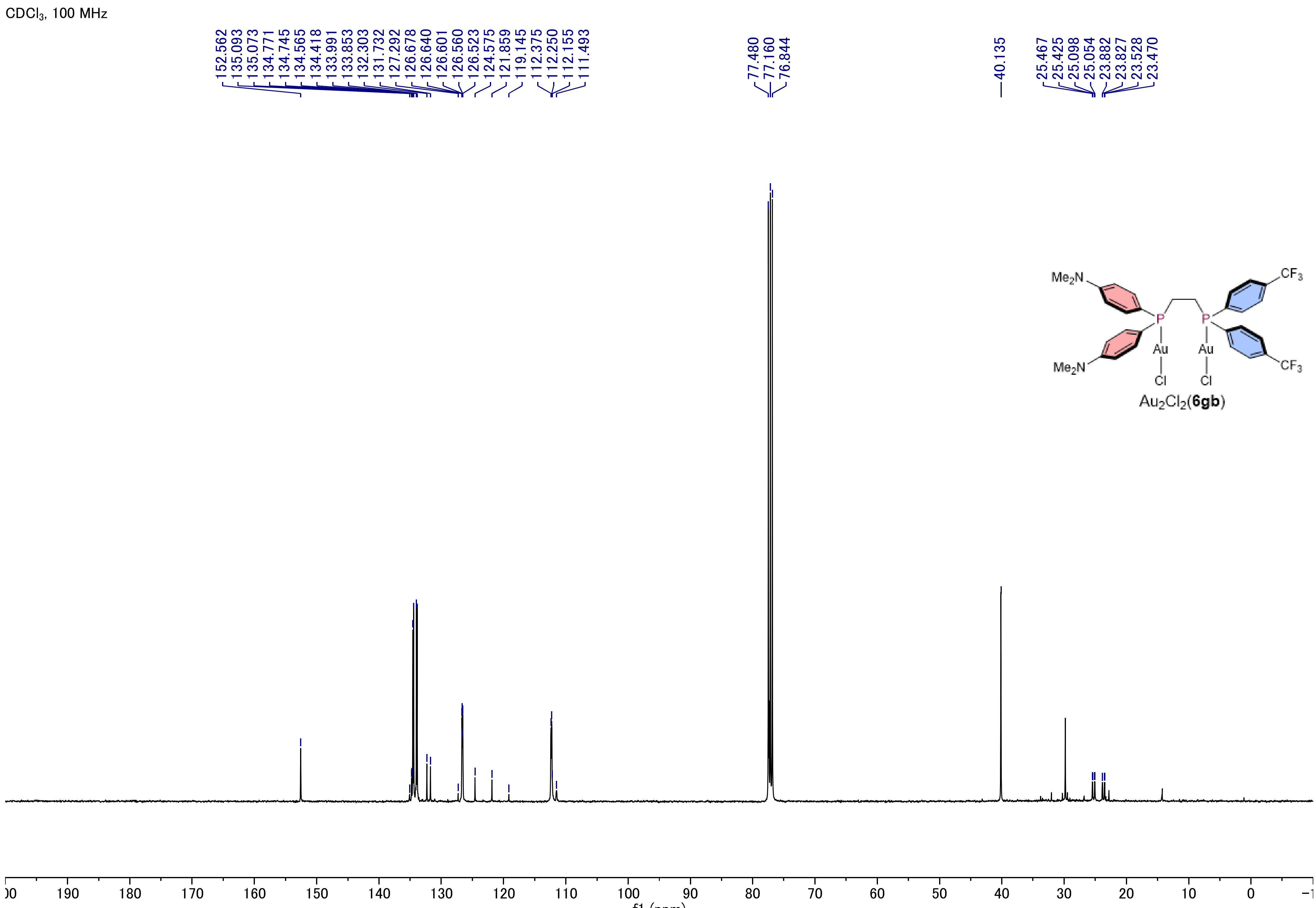

Supplementary Figure 212. ^{13}C NMR spectrum (100 MHz, $CDCl_3$) of Au_2Cl_2(**6gb**)

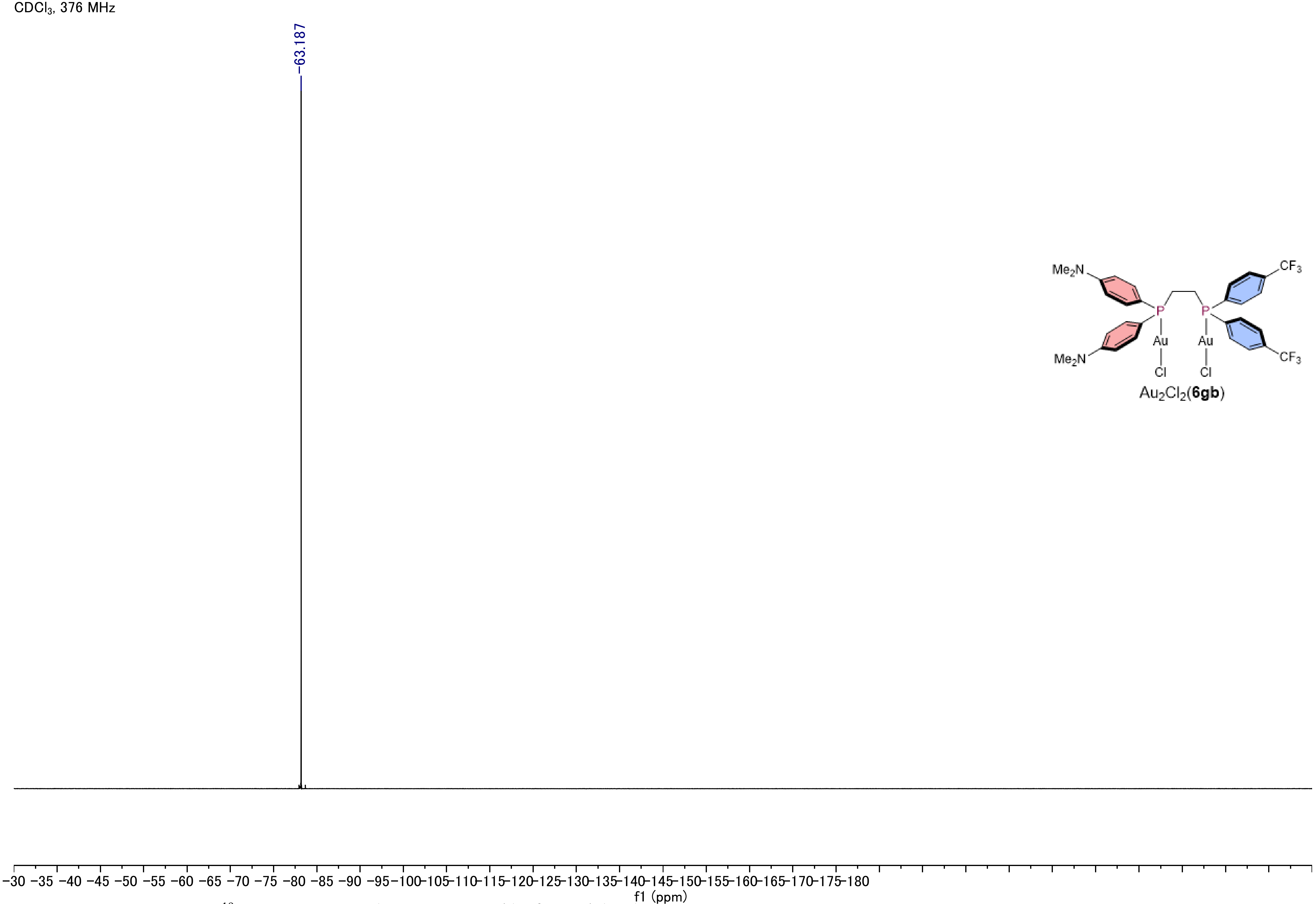

Supplementary Figure 213. ^{19}F NMR spectrum (376 MHz, $CDCl_3$) of Au_2Cl_2(**6gb**)

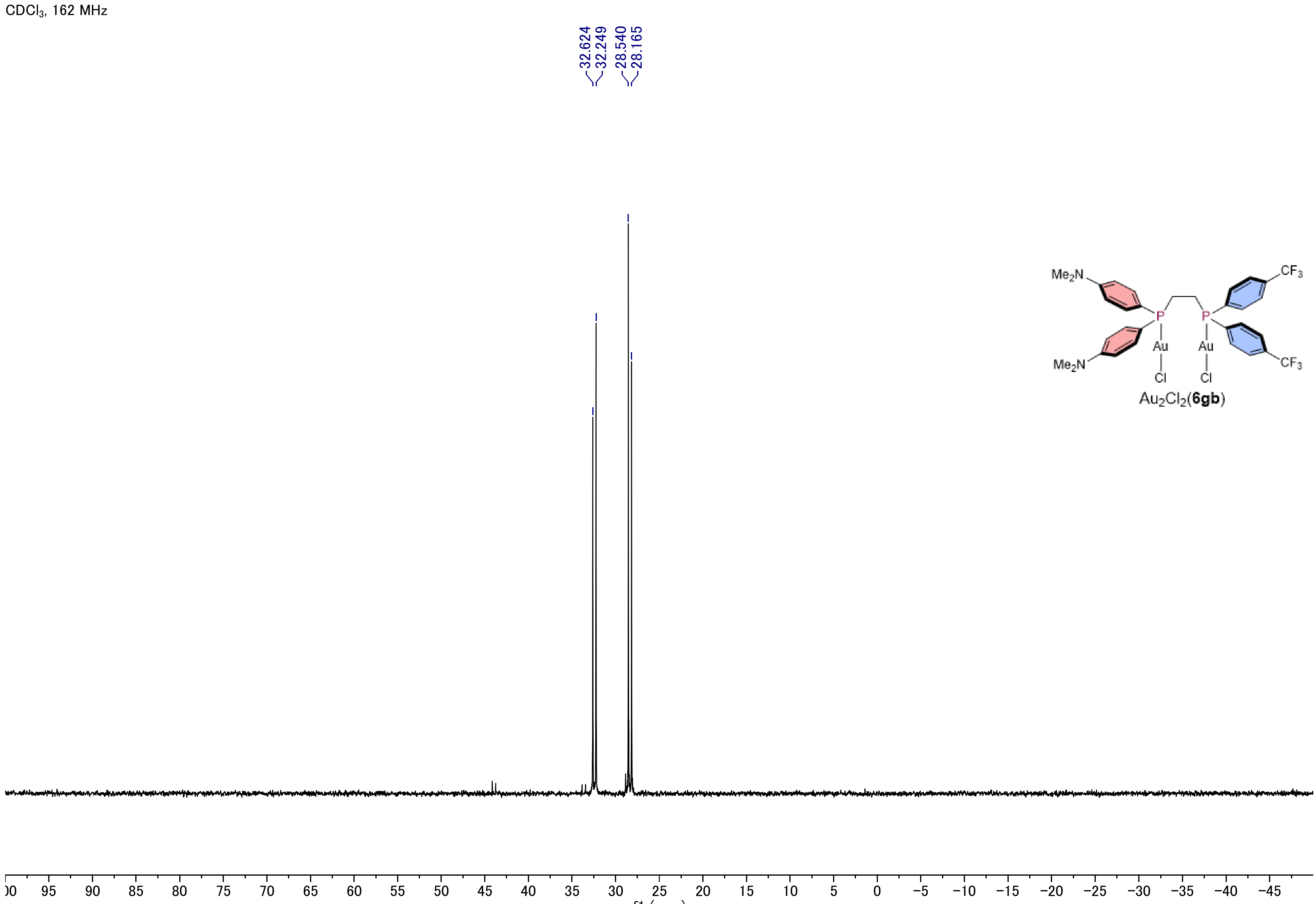

Supplementary Figure 214. ^{31}P NMR spectrum (162 MHz, $CDCl_3$) of Au_2Cl_2(**6gb**)
