# Supplementary material for: A theory-driven synthesis of symmetric and unsymmetric 1,2-bis(diphenylphosphino)ethane analogues via radical difunctionalization of ethylene
Source: Nat Commun. 2022 Nov 21;13:7034. doi: 10.1038/s41467-022-34546-5 (PMC9678890; doi:10.1038/s41467-022-34546-5)
Supplement: Supplementary file 3 — Description of Additional Supplementary Files [file 41467_2022_34546_MOESM3_ESM.pdf]

Description of Additional Supplementary Files

Supplementary Data 1: Calculation methods and cartesian coordinates of intermediates and transition states
