# Supplementary material for: A theory-driven synthesis of symmetric and unsymmetric 1,2-bis(diphenylphosphino)ethane analogues via radical difunctionalization of ethylene
Source: Nat Commun. 2022 Nov 21;13:7034. doi: 10.1038/s41467-022-34546-5 (PMC9678890; doi:10.1038/s41467-022-34546-5)
Supplement: Supplementary file 4 — Supplementary Data 1 [file 41467_2022_34546_MOESM4_ESM.pdf]

Supplementary Data 1

A theory-driven synthesis of symmetric and unsymmetric 1,2-bis(diphenylphosphino)ethane analogues via radical difunctionalization of ethylene

Hideaki Takano,[1,2] Hitomi Katsuyama,[1,2] Hiroki Hayashi,[1,2] Wataru Kanna,[3] Yu Harabuchi,[1,2,3] Satoshi Maeda,[*,1,2,3,4] and Tsuyoshi Mita[*,1,2]

[1] *Institute for Chemical Reaction Design and Discovery (WPI-ICReDD), Hokkaido University, Kita 21 Nishi 10, Kita-ku, Sapporo, Hokkaido 001-0021, Japan*

[2] *JST, ERATO Maeda Artificial Intelligence in Chemical Reaction Design and Discovery Project, Kita 10 Nishi 8, Kita-ku, Sapporo, Hokkaido 060-0810, Japan*

[3] *Department of Chemistry, Faculty of Science, Hokkaido University, Kita 10 Nishi 8, Kita-ku, Sapporo, Hokkaido 060-0810, Japan*

[4] *Research and Services Division of Materials Data and Integrated System (MaDIS), National Institute for Materials Science (NIMS), Tsukuba, Ibaraki 305-0044, Japan*

smaeda@eis.hokudai.ac.jp

tmita@icredd.hokudai.ac.jp

Table of Contents

(A)	AFIR-based calculations for backward reaction path search (Fig. 1)	3
(B)	Plausible reaction mechanism shown in Fig. 6	11
(C)	HOMO and LUMO of Pd complexes	22
(D)	References	26

(A) AFIR-based calculations for backward reaction path search (Fig. 1)

In this study, the artificial force induced reaction (AFIR) method implemented in the global reaction route mapping (GRRM) program[1] combined with the Gaussian 16 program[2] was used and all calculation results were obtained by following process. The single component artificial force induced reaction (SC-AFIR)[3,4] method with the pair of negative artificial force between the phosphorus atom and the methylene carbon on DPPE was conducted to obtain the equilibrium structures (EQs) and path-top structures of AFIR path (PTs) of decomposition of DPPE via the cleavage of the C–P bond with the UωB97X-D/Def2-SV(P) level of theory in dichloromethane (CPCM model), Grid = FineGrid, charge = 0, and spin = singlet with the option of Stable = Opt by which MO-stability check is performed at some geometries. In the SC-AFIR search, the model collision energy parameter, γ, was set to 500 kJ/mol. After SC-AFIR calculation, the AFIR path connected to diphosphine and ethylene was further optimized by the LUP[5,6] and IRC methods with UωB97X-D/Def2-SV(P) with Grid = UltraFine in dichloromethane (CPCM model) and charge = 0 with both spin states (spin = singlet with Stable = Opt, and spin = triplet) to afford transition-state structures (TSs) which were the first-order saddle point and EQs before and after the radical addition. Obtained EQs and TSs discussed in the text are local minima and first-order saddles, respectively.

Structures of EQs and TSs

DPPE

Electron Energy = –1686.625369560555

Free Energy = –1686.255407740128

C	0.650828806611	-1.457829046344	-0.874367766641
C	-0.826283942038	-1.737632198452	-0.576489209656
H	1.052523877256	-2.322784583320	-1.431092437991
H	0.749171736546	-0.578654681083	-1.534502291950
H	-1.380867138399	-1.827646738652	-1.527034392121
H	-0.923228143504	-2.713298833303	-0.067203860624
P	1.723207628327	-1.265683552698	0.637250258860
P	-1.677106580366	-0.521625900873	0.557429756094
C	1.625819388163	0.556510905972	0.953669724279
C	1.266272870365	0.993863382427	2.233250518046
C	1.919214202134	1.513621558763	-0.028078163630
C	1.197534689584	2.357405946597	2.528000752434
H	1.030267969015	0.259044128356	3.009473374950
C	1.849358083601	2.874029746963	0.261300416024
H	2.214812414161	1.196706490477	-1.033797818722
C	1.488703749980	3.298687372675	1.542267217704
H	0.913223828093	2.683375484245	3.532834331728
H	2.075574124372	3.608371743623	-0.517141465928
H	1.431558214278	4.367314200314	1.769303343067
C	3.417515449747	-1.343632579763	-0.095441911525
C	4.485968072208	-1.449772334732	0.808733166261

C	3.707414091498	-1.291616825669	-1.465828673884
C	5.805616827471	-1.487164205890	0.360674380694
H	4.281569051421	-1.502690790940	1.883877715545
C	5.028152196240	-1.339298801227	-1.917667672282
H	2.905116757714	-1.207561674743	-2.203557934117
C	6.080422464958	-1.433969963834	-1.007291780266
H	6.623308004944	-1.566077912219	1.083029915236
H	5.233625734679	-1.297963471580	-2.991394885243
H	7.114184770028	-1.469589355492	-1.362971349223
C	-1.616954092366	1.050156515799	-0.393965097570
C	-1.684643607446	2.240627962415	0.340676787285
C	-1.495283129790	1.128104529840	-1.788933072179
C	-1.638412613062	3.479844915400	-0.297975338803
H	-1.758810064669	2.198305894960	1.431929562785
C	-1.434922337214	2.365331872981	-2.429549794100
H	-1.452112702288	0.217393836322	-2.393251765353
C	-1.507977007095	3.544490172176	-1.684732016291
H	-1.688309570225	4.398652502980	0.293288821213
H	-1.333837199405	2.409016444070	-3.517911061278
H	-1.460075513385	4.514634690688	-2.187947556060
C	-3.416243976703	-1.085622247735	0.301371160778
C	-4.370629433079	-0.385390815362	-0.447862926829
C	-3.803127194221	-2.280010357284	0.930672500410
C	-5.673535175621	-0.872674401022	-0.574046130155
H	-4.100209869555	0.552149185811	-0.941753470143
C	-5.099436407323	-2.774551730701	0.793686728066
H	-3.081355247849	-2.834114466221	1.541532161375
C	-6.040854202763	-2.069543033520	0.040728567266
H	-6.405742181057	-0.311131075859	-1.161901494271
H	-5.378516263253	-3.710754622030	1.286076540524
H	-7.060886449198	-2.451031866820	-0.061674636326

INT-I-triplet

Electron Energy = –1686.529054122631

Free Energy = –1686.169301052489

C	0.009856420283	2.835442458690	-1.202793044428
C	-1.374737098739	2.344246746557	-1.020065944060
H	0.509568810180	2.721655764962	-2.170759635842
H	0.632950121950	3.103416719213	-0.342088405065
H	-1.897991207428	2.851708355040	-0.190099219110
H	-1.977929695383	2.500477482473	-1.933022674847

P	2.188684800800	-0.402591307549	-2.013118113980
P	-1.452725167542	0.475694140848	-0.750708316672
C	1.918611597474	-1.895485602307	-1.016962584880
C	0.892381271787	-2.760688863036	-1.443631964838
C	2.700318938288	-2.285402724524	0.086702725862
C	0.627221843554	-3.948872886385	-0.766193380944
H	0.278157449855	-2.486443269670	-2.307662885566
C	2.434807654510	-3.473683035455	0.763259400656
H	3.531900912494	-1.657980490392	0.418847954460
C	1.392274887735	-4.304635214270	0.346554183276
H	-0.184657453181	-4.598518396473	-1.105554219808
H	3.049526032185	-3.755555068527	1.623038414965
H	1.183227100819	-5.234678596683	0.882842234715
C	2.806841201938	0.850767455259	-0.861231878257
C	3.477534895217	1.944819570673	-1.448136391072
C	2.576711871943	0.883285735285	0.528225755989
C	3.911393596183	3.020490318137	-0.678774709161
H	3.656473898313	1.951639250765	-2.528958580077
C	3.011579322577	1.960916034872	1.296127656699
H	2.026782510769	0.074405600210	1.015167403693
C	3.680179339324	3.031720862157	0.699266757771
H	4.430880676555	3.856375950163	-1.156098062430
H	2.811676637229	1.967733135629	2.371419746047
H	4.016879774349	3.877107094819	1.306191898096
C	-0.803511151715	0.273848192217	0.959611861300
C	-0.502263454973	-1.034869148810	1.365986309251
C	-0.560778371086	1.322907573432	1.855678467848
C	0.030008324484	-1.289756382561	2.628830461460
H	-0.666076347977	-1.869672940036	0.677233190740
C	-0.021946721766	1.071867464495	3.118337608628
H	-0.781100839635	2.356602925519	1.577987966488
C	0.278841346315	-0.233679391061	3.507308305231
H	0.265379332933	-2.317553786525	2.919264146271
H	0.169084672190	1.905530405738	3.800367484676
H	0.708256069891	-0.428037184668	4.494379658969
C	-3.272701632885	0.298524591465	-0.484648201500
C	-3.908671194261	0.483681069059	0.750943572380
C	-4.057026771208	-0.018239622979	-1.603642414207
C	-5.293947526517	0.358469975864	0.862673079125
H	-3.317991745343	0.725981621902	1.639700008730
C	-5.443592771004	-0.133668712961	-1.496032059810

H	-3.576126486875	-0.180508492702	-2.574637902982
C	-6.064961789213	0.052258118850	-0.260343651551
H	-5.774890903563	0.502701857748	1.834714259247
H	-6.039623207566	-0.378946969153	-2.379967299017
H	-7.150827680168	-0.045469297258	-0.171065928350

TS-I-triplet

Electron Energy = – 1686.511891455041

Free Energy = – 1686.151842326329

C	1.555685187019	-4.737490959292	-0.447522725959
C	2.243334302416	-3.614392021955	-0.846549589021
H	1.064095092325	-5.393644752923	-1.175107240627
H	1.395326675358	-4.955587011965	0.614097476692
H	2.880914071242	-3.097244268380	-0.119503629585
H	2.546702813455	-3.511449067864	-1.895641555418
P	-0.985806701255	2.075228955125	-1.409171437812
P	0.796135270985	-1.693920469670	-1.008411707793
C	-0.365212970684	2.358101547845	0.273432432868
C	1.019349582526	2.573056248471	0.413690596938
C	-1.173852154808	2.458651441719	1.421906231827
C	1.582003189509	2.830509246055	1.662008452824
H	1.670983201558	2.511313006676	-0.463762187002
C	-0.611024191292	2.723163959042	2.667725978407
H	-2.257295175508	2.333788594128	1.342120118860
C	0.769253766137	2.899060588738	2.794976030247
H	2.663246317869	2.970976333373	1.748648594938
H	-1.255394915746	2.791399454504	3.548984183646
H	1.209101048581	3.097790614242	3.776502331930
C	-2.423259355663	0.987942381825	-1.212922563582
C	-3.346779136223	0.981822063641	-2.279767074522
C	-2.635217415712	0.088427280818	-0.148474882066
C	-4.446747398660	0.127707357107	-2.274416331545
H	-3.199497529777	1.661494764057	-3.126253599505
C	-3.732416114599	-0.769650200582	-0.148719796128
H	-1.925745083685	0.039318237473	0.680572484792
C	-4.644110983856	-0.751441223339	-1.206834596135
H	-5.153123616258	0.146071706332	-3.109331592074
H	-3.868745303157	-1.465255286668	0.684385893379
H	-5.504960660999	-1.426205029038	-1.202769751307
C	0.165233352118	-1.752260858199	0.700002453833
C	0.395217214630	-0.798124247546	1.704981601353

C	-0.745946951456	-2.791616082070	0.968702241904
C	-0.258535390952	-0.886076054451	2.934225656824
H	1.058579927442	0.049982193632	1.522766583864
C	-1.394405336308	-2.882122690383	2.197575118875
H	-0.962232078010	-3.535156134820	0.193597144158
C	-1.152973221702	-1.926572227840	3.187810859299
H	-0.072742213988	-0.121707882791	3.694131854111
H	-2.099654518040	-3.698112428895	2.380769029553
H	-1.666387396052	-1.990042688202	4.151511573122
C	2.161884245819	-0.482332154051	-0.997409498076
C	3.117661953827	-0.332312405947	0.022951005819
C	2.297419650554	0.304848816377	-2.154619223350
C	4.147407348152	0.599001180225	-0.094755549977
H	3.061152835850	-0.947547488014	0.925721516128
C	3.331805809585	1.232396585525	-2.277126765059
H	1.573137623564	0.196844087879	-2.968522709782
C	4.255173245855	1.388144999507	-1.242470594560
H	4.874973232715	0.707005764304	0.714852251989
H	3.412012323904	1.840633979605	-3.182588753591
H	5.063735610799	2.119180518403	-1.332257702573

TS-I-singlet

Electron Energy = – 1686.519813196482

Free Energy = – 1686.155441360108

C	-0.241030724956	-4.536591842050	-0.295333376539
C	-1.217698043774	-3.581649852753	0.099715094806
H	0.339758064635	-5.094906380256	0.447597883970
H	0.046036061845	-4.643347013224	-1.346427293831
H	-1.955770486707	-3.340988414802	-0.680516755819
H	-1.698040492174	-3.775966598289	1.070797001688
P	0.447785230162	0.827729404453	1.705586829025
P	-0.554677825765	-1.623525320703	0.508257370103
C	-0.010753257696	2.097970795603	0.483041567517
C	-1.390667056918	2.356749043877	0.340142901267
C	0.882775401466	2.879617149848	-0.273498442886
C	-1.857408644866	3.309149938486	-0.561565589019
H	-2.113691351192	1.790103329549	0.934599678825
C	0.414772194462	3.839471616916	-1.169670454419
H	1.961076972546	2.751084276287	-0.156651661460
C	-0.956033962149	4.049171812181	-1.330751434282
H	-2.933955453108	3.475332165141	-0.662653536219

H	1.131199894733	4.431224616175	-1.747340529885
H	-1.320018508599	4.796579715853	-2.041582539765
C	2.205654239927	0.445393627686	1.383974954111
C	2.982877351880	0.113015606587	2.508960724317
C	2.809012087489	0.362308733963	0.115861098670
C	4.317087103457	-0.270387998887	2.376738886681
H	2.534359921732	0.158717620532	3.507292003898
C	4.142787738745	-0.019644487671	-0.016926547728
H	2.229686494152	0.581840077234	-0.784012447760
C	4.902702531985	-0.334837085380	1.111304991175
H	4.902043635632	-0.518910389885	3.267178098694
H	4.587902587742	-0.081142549009	-1.014435254658
H	5.949022577050	-0.635390058170	1.003744346258
C	0.124466597925	-1.205398237487	-1.146466171765
C	-0.278668428885	-0.125629381763	-1.945485655774
C	1.246127291997	-1.948822164515	-1.550037804361
C	0.410465016269	0.188993233295	-3.117560283327
H	-1.119448657599	0.502700683478	-1.645176878378
C	1.925702163905	-1.645476083019	-2.727928157157
H	1.611871686547	-2.767240672926	-0.921362844550
C	1.509844374325	-0.571796565895	-3.516739548105
H	0.085259079506	1.043124854230	-3.718558549480
H	2.796165586941	-2.239715659364	-3.020392510672
H	2.047154433908	-0.322914126764	-4.436371583430
C	-2.266694979553	-0.950540973326	0.553234281717
C	-3.113661435568	-0.837042573524	-0.558143051770
C	-2.773785572556	-0.613826027498	1.817767194477
C	-4.414133880761	-0.351914453232	-0.415840601655
H	-2.763208891142	-1.128229619918	-1.552439813388
C	-4.076951129233	-0.139957138153	1.964182185367
H	-2.132214110003	-0.703965872255	2.700134760684
C	-4.897006363861	0.004929478534	0.843936556022
H	-5.055646084205	-0.257294699874	-1.296756466353
H	-4.450537510168	0.124442884487	2.957537546671
H	-5.915828849770	0.387364130805	0.953779578542

INT-II-triplet

Electron Energy = –1686.526839203476

Free Energy = – 1686.171317820780

C	4.277357547537	2.718585043650	-0.033130200078
C	4.292513728298	2.463148769645	1.275211643780

H	5.196619254940	2.961462572591	-0.580741348699
H	3.344704790419	2.693778162789	-0.610796573286
H	3.371594539393	2.218741873366	1.820753818704
H	5.222330604103	2.484356776958	1.857181305484
P	-2.137265594884	-1.997343540013	0.246962653372
P	0.638010153510	0.728121724261	1.842254102523
C	-1.123936564636	-1.787876850921	-1.247960588159
C	0.116613245742	-2.451799013875	-1.279810849563
C	-1.530907203631	-1.080745707158	-2.395529415852
C	0.942597338785	-2.373402933525	-2.399745222908
H	0.454174449961	-3.016314501535	-0.404643298692
C	-0.707283481269	-1.008185839575	-3.516277836392
H	-2.505232552179	-0.584364192016	-2.416459037981
C	0.536274831308	-1.645261366016	-3.519114420504
H	1.912645563913	-2.878482654127	-2.392267949180
H	-1.038331553855	-0.448680971621	-4.395923416422
H	1.184589684878	-1.579365362079	-4.397526927592
C	-2.973989493670	-0.407939497728	0.480334854846
C	-4.084209453363	-0.416020692461	1.352042081985
C	-2.556394847079	0.831433031918	-0.046152180570
C	-4.756579102084	0.760706460828	1.671621211421
H	-4.424728868161	-1.363049737478	1.784995903958
C	-3.225845897841	2.008036263387	0.280255775650
H	-1.685627199221	0.885019150167	-0.702791653099
C	-4.329494194944	1.978495143789	1.136117002085
H	-5.617791758777	0.728533869511	2.345140989517
H	-2.875093876656	2.958018374239	-0.133481888064
H	-4.853488392024	2.904361501635	1.390061070939
C	0.783119412665	1.693474683200	0.315362336653
C	0.954089878502	1.160233613450	-0.977337543004
C	0.585477932354	3.083537017216	0.439744574507
C	0.942833564050	1.989373631235	-2.096119244812
H	1.069055683530	0.082820244252	-1.118411759895
C	0.580151617193	3.911918910350	-0.679933654676
H	0.436511849192	3.522164308286	1.432287660208
C	0.760723168812	3.367128394109	-1.953254110252
H	1.069663649447	1.550720099082	-3.089865778742
H	0.433397296469	4.988893361035	-0.558482336144
H	0.753836563071	4.015098376457	-2.834270951762
C	1.536331178874	-0.814784735778	1.521843836511
C	2.679722257321	-0.932260816569	0.708186057303

C	1.091901119569	-1.957283248472	2.216256143643
C	3.328700475119	-2.156440571537	0.567650401795
H	3.076154005115	-0.054872483214	0.190043274474
C	1.741608324632	-3.181698976946	2.073227065517
H	0.215118249786	-1.887563139573	2.868626582664
C	2.858343889005	-3.286480151943	1.241514831091
H	4.213545566810	-2.228620967399	-0.071293873934
H	1.371674168363	-4.059336656852	2.610813243752
H	3.368473304892	-4.246914170441	1.125275661788

SM-singlet

Electron Energy = – 1686.588960264841

Free Energy = – 1686.227693568088

C	2.522576965649	-4.013321342266	-0.382201304401
C	3.572825330318	-3.232489648267	-0.635232586317
H	1.736583983342	-4.176565175870	-1.129557459362
H	2.397459282301	-4.516120663197	0.584641781078
H	4.356411999321	-3.060287401271	0.112656787960
H	3.689991622747	-2.719540115142	-1.597558470035
P	-0.808330455483	0.865410860393	-1.378798807097
P	0.504558870535	-0.883664200174	-0.871260087276
C	-0.691202776914	1.996659645318	0.066392807436
C	0.491553047975	2.750300775155	0.171141655913
C	-1.681180873246	2.169455769608	1.042215923781
C	0.700950164051	3.604214514518	1.251825061391
H	1.268135922103	2.658101672220	-0.594516704956
C	-1.477795853645	3.038574472646	2.115802897262
H	-2.624587999570	1.623098001844	0.976137085664
C	-0.282399954749	3.746722091409	2.233428361586
H	1.635684328181	4.167780372220	1.322942858934
H	-2.261732495537	3.156008181671	2.869665786057
H	-0.121096267639	4.418794893323	3.081114468881
C	-2.468617686021	0.077005197432	-1.260571265295
C	-3.286557105340	0.176100626780	-2.395874846760
C	-2.952689020604	-0.629411688465	-0.146670699619
C	-4.552372705593	-0.412182211246	-2.422251642854
H	-2.927101482284	0.720470466310	-3.274970526562
C	-4.216344004378	-1.217421750371	-0.171723465743
H	-2.342557364712	-0.724656147654	0.754562925920
C	-5.018934105510	-1.110969076668	-1.309525550310
H	-5.174529910688	-0.323217827639	-3.317362058438

H	-4.575176039377	-1.764582690050	0.704758105174
H	-6.009778133605	-1.574027385408	-1.327290971230
C	0.203494978624	-1.270004784360	0.901605378954
C	0.372141111804	-0.377516707448	1.972719744668
C	-0.304361585266	-2.550300465430	1.169951419936
C	0.032122284819	-0.755661831483	3.270723310065
H	0.768862545756	0.625127479458	1.798516936171
C	-0.646785882871	-2.929298874448	2.468188742012
H	-0.445181539281	-3.257869800989	0.346965200414
C	-0.481606816182	-2.029792823240	3.521415397993
H	0.165993995208	-0.045902587682	4.092098401341
H	-1.044693937649	-3.930622186420	2.655839629742
H	-0.750768467762	-2.321859853728	4.540637447730
C	2.129351126763	-0.017249047754	-0.910553819439
C	3.042001548837	-0.024008588680	0.151338567757
C	2.510466277138	0.598645617091	-2.114979357799
C	4.287157327314	0.595643261551	0.024967476547
H	2.793222430139	-0.523620010963	1.090127253695
C	3.750239949355	1.221545644066	-2.239242648488
H	1.824304994528	0.602118589405	-2.968311084936
C	4.643690118247	1.227022488282	-1.165308888772
H	4.983064291519	0.581296541682	0.868910716052
H	4.021691323964	1.703090594902	-3.183166845656
H	5.617451582580	1.715743373136	-1.260938369242

(B) Plausible reaction mechanism shown in Fig. 6

Geometry optimizations were performed at the with the (U)ωB97X-D/Def2-SV(P) level of theory with Grid = UltraFine in dichloromethane (CPCM model) and charge = 0 with both spin states (spin = singlet with Stable = Opt, and spin = triplet) to afford TSs and EQs.

Structures of EQs and TSs

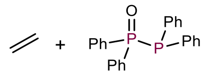

Free Energy = –1761.389938133459

C	3.765873533730	1.473977882464	-1.029787723673
C	4.074433135383	0.788920116292	-2.131174639147
H	3.938249727824	2.554555490548	-0.957278716259
H	3.321201963812	0.986909388030	-0.152238307279
H	4.515877027426	1.277070791611	-3.008888806089
H	3.896713088903	-0.291549310470	-2.201672051389
P	-0.915990489147	0.426468269683	1.628564134401

P	-0.009509138568	-1.397490821958	0.696180139881
C	-2.654609821727	0.269485213896	1.039653160531
C	-3.314573113811	-0.969396009312	1.075188419445
C	-3.391229644314	1.414786261724	0.699289505077
C	-4.665050484356	-1.061084204673	0.737879947228
H	-2.763870076450	-1.869834859200	1.361085345572
C	-4.742727748987	1.318633977287	0.368734713269
H	-2.912115396261	2.397909700001	0.688445100722
C	-5.383916775000	0.079491656473	0.379996240252
H	-5.158494237973	-2.037169252591	0.759397373994
H	-5.297140936934	2.222876063628	0.101374306670
H	-6.442988940416	0.004754272643	0.117385188501
C	-0.197803312127	1.775152758561	0.604540936675
C	0.801234291919	2.559758047594	1.199762503678
C	-0.583084595849	2.060943997964	-0.714013904969
C	1.410023789062	3.596219246473	0.492310214788
H	1.105109423027	2.364117934622	2.232794985544
C	0.020562590757	3.100167320644	-1.418871558928
H	-1.360563720354	1.466080404055	-1.199973117417
C	1.020077132691	3.867873663379	-0.818713416077
H	2.189932382582	4.195180409484	0.970597363047
H	-0.289068252271	3.306861556618	-2.447214106011
H	1.495082855680	4.680987867067	-1.374963213358
C	-0.221615444112	-1.328548942296	-1.104916309888
C	-1.264445516302	-2.081470925495	-1.659121656307
C	0.574065834574	-0.528055107422	-1.934653763213
C	-1.515599652006	-2.023378163515	-3.029695590994
H	-1.872975245428	-2.718054779332	-1.011299944387
C	0.325639919899	-0.478257406449	-3.304483838487
H	1.392242719715	0.063470231702	-1.516595385792
C	-0.721972721959	-1.221622223653	-3.852059915314
H	-2.331960698415	-2.611336348623	-3.458154681821
H	0.953149465355	0.147794392337	-3.945025150875
H	-0.918348870434	-1.179282242779	-4.927254958882
C	1.778056778070	-1.241150568799	1.008323269327
C	2.687011357369	-1.893583843613	0.162149803484
C	2.256999632032	-0.586996857458	2.150957580239
C	4.051585626801	-1.869795011331	0.442666815122
H	2.331653008385	-2.421725025039	-0.727511659135
C	3.624003419958	-0.556404901943	2.425271212218
H	1.562809800185	-0.098870170917	2.841539659760

C	4.522126499319	-1.195155319720	1.570197382950
H	4.751669373711	-2.376292887412	-0.227087862632
H	3.986850173065	-0.033650496387	3.314221936363
H	5.594133364655	-1.169743898723	1.784544560186
O	-0.636025400818	-2.632105585947	1.298296578540

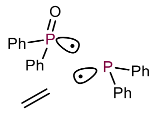

Free Energy = –1761.318559881216

C	-1.462350228004	-2.845315441280	0.693521972250
C	-2.627195616429	-3.117891584059	0.101926560785
H	-0.516087126055	-2.848752246855	0.138005178322
H	-1.402371509554	-2.614360415110	1.764037329162
H	-2.686298700871	-3.356328571793	-0.967608945342
H	-3.572541703215	-3.110439740494	0.657872886440
P	2.065568619670	-0.293738967971	2.034418576252
P	-1.491106737657	0.533761062414	0.551366543556
C	2.741197387167	1.090869036284	1.074655656582
C	2.260847825109	2.375749861926	1.391853562499
C	3.755245181917	0.978513308014	0.104245228620
C	2.749800192176	3.503867928080	0.736532814368
H	1.462206754571	2.483406224354	2.131416274989
C	4.242749708944	2.107366755950	-0.549816202599
H	4.174382269689	-0.001624430324	-0.140052299720
C	3.737248608841	3.372882248426	-0.241827685700
H	2.351234226629	4.491647609685	0.985593437708
H	5.027242558654	1.999319032780	-1.304484632313
H	4.119001987581	4.257494622027	-0.759729476355
C	2.111292204713	-1.710068020143	0.903666766767
C	2.123296664639	-2.989194854275	1.498174455889
C	2.033461459394	-1.632695324744	-0.500758897992
C	2.081361312549	-4.143987593746	0.720969406311
H	2.167767132897	-3.077620427154	2.589104620982
C	1.984868160858	-2.788842470881	-1.276107389038
H	1.990226738627	-0.658401201199	-0.994585548570
C	2.013104534151	-4.047514628238	-0.670961342856
H	2.097233321092	-5.125208766963	1.203811996419
H	1.914329366768	-2.703551590215	-2.364323293596
H	1.974322191330	-4.952825229619	-1.283378184906
C	-0.646645619566	0.599774093991	-1.052861099945

C	0.224907697236	1.672488300343	-1.286367846596
C	-0.746740001538	-0.431171236031	-1.998209230004
C	0.976961759989	1.719439876874	-2.458591149195
H	0.321641181920	2.463921145124	-0.540020445947
C	-0.007976198062	-0.369186531078	-3.177978658744
H	-1.376367126334	-1.303429706911	-1.805741310062
C	0.859118528118	0.701334091213	-3.406694170243
H	1.661733652723	2.555179591137	-2.626808954868
H	-0.092614888443	-1.174709343013	-3.912459468778
H	1.451070091616	0.737855205794	-4.325543710494
C	-3.291834339395	0.610064757903	0.327320881589
C	-3.966426247989	0.003927363865	-0.741925746113
C	-4.027387688684	1.258759243394	1.330326830275
C	-5.357518017083	0.057964229561	-0.809761360121
H	-3.415335996115	-0.504318653373	-1.536265297996
C	-5.418408402006	1.309075159296	1.257380333955
H	-3.501036536212	1.731782667488	2.163870052021
C	-6.085786443538	0.707944790024	0.188854838491
H	-5.875768056445	-0.410663453688	-1.650924975132
H	-5.983957962409	1.823064880590	2.039682349135
H	-7.177316193066	0.746478509103	0.132802819991
O	-0.997442451107	1.578925411976	1.532039468259

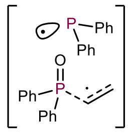

Free Energy = –1761.311006344158

C	-1.194573377432	-2.887597069265	0.535965298332
C	-1.317846562293	-1.982569527169	1.544876309308
H	-2.072464383216	-3.390508245818	0.112816711604
H	-0.220273602066	-3.100943705490	0.081230715380
H	-2.271100178154	-1.866851652410	2.075601778399
H	-0.427354519514	-1.594410685995	2.054416955425
P	2.147963893625	-0.177192328730	1.967754019635
P	-1.656887068333	0.356594266405	0.590178452467
C	2.671938473146	1.267605424265	1.005033123101
C	2.066732421731	2.497518208686	1.326044304783
C	3.682711424876	1.255103553922	0.025119073075
C	2.433218003688	3.667914353157	0.664895637815
H	1.266834665238	2.525479520319	2.072066613193
C	4.048797115398	2.426376844992	-0.633271239153

H	4.194108123212	0.320879342294	-0.223290521278
C	3.420935332238	3.634991414923	-0.321409746987
H	1.938687688302	4.610769489836	0.915740112442
H	4.833255274793	2.396883027307	-1.395067551344
H	3.707384832202	4.552686930272	-0.843166140932
C	2.305689923466	-1.579501521785	0.828735067090
C	2.451073821375	-2.854121472025	1.414597488529
C	2.191387245099	-1.501783508335	-0.572927535963
C	2.508053988248	-4.003107340794	0.629522127994
H	2.523063883871	-2.943961037124	2.503964586870
C	2.244955414046	-2.652439082586	-1.355786779771
H	2.037634438272	-0.533954799542	-1.057469371454
C	2.409066587880	-3.905403111208	-0.760521723132
H	2.627647508817	-4.980865107651	1.104781227716
H	2.145568069395	-2.568207692310	-2.441772700005
H	2.450349082992	-4.806621436674	-1.378833997583
C	-0.787218143490	0.407097996848	-1.003501939889
C	0.033417327797	1.517873491643	-1.243443085943
C	-0.845583401404	-0.625232955180	-1.949908149297
C	0.779672663168	1.599420419544	-2.417485593349
H	0.088537690883	2.315812464088	-0.500012501500
C	-0.105995789714	-0.534773230790	-3.128085367869
H	-1.450055313156	-1.517311937260	-1.770937276408
C	0.710682562398	0.573415014018	-3.361800523312
H	1.421487359210	2.468079558155	-2.589091051894
H	-0.156508511929	-1.344124107836	-3.861598831687
H	1.298467466079	0.634804368431	-4.282055521753
C	-3.448925337560	0.319671749497	0.289851418504
C	-4.069730253380	-0.645952239415	-0.515385167701
C	-4.234111780281	1.262390058936	0.967740203740
C	-5.456113736072	-0.650002439434	-0.658669159242
H	-3.480470471283	-1.404914588621	-1.035840661552
C	-5.620870809709	1.254321891763	0.820965515435
H	-3.747549574856	2.004863506364	1.606091824087
C	-6.233539245165	0.300303345297	0.007206612528
H	-5.932604262649	-1.403232348249	-1.292219502656
H	-6.226174276487	1.998773079106	1.345989991253
H	-7.321309540548	0.293535672799	-0.106150959834
O	-1.259265668069	1.493957829131	1.505817465081

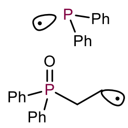

Free Energy = –1761.340130871588

C	-0.207158851134	-2.745765977545	1.142432532230
C	-1.600317011601	-2.282566749394	0.941015871781
H	0.409534402046	-3.060546832196	0.293934751869
H	0.286779269711	-2.583657884394	2.105108035613
H	-2.103134390025	-2.795267589194	0.102023642500
H	-2.219309457945	-2.440606127753	1.843795654100
P	2.467884351860	0.159146464938	1.936097096220
P	-1.717536210478	-0.462389034579	0.658047691566
C	2.422674460888	1.676277523809	0.937129672818
C	1.509942428368	2.674338019723	1.324411093241
C	3.295708552242	1.948218407802	-0.132821642648
C	1.450530541536	3.891012127885	0.647002127019
H	0.812625411287	2.476880484596	2.144248271539
C	3.237769049414	3.165787198695	-0.806915518865
H	4.034051565095	1.201700711277	-0.439883733125
C	2.311772568965	4.139058256690	-0.423339936523
H	0.722881498500	4.648167940069	0.953310245689
H	3.922509885900	3.358169640659	-1.638059767567
H	2.265542334089	5.093126186809	-0.956414711416
C	2.808789244392	-1.164315791568	0.749176445828
C	3.309755146958	-2.369666046105	1.284597658437
C	2.511790698898	-1.123044998178	-0.627279301192
C	3.517605702996	-3.484051953957	0.476855777956
H	3.535023848516	-2.432812716910	2.354859658774
C	2.720699312822	-2.239182198641	-1.433695019531
H	2.088246915097	-0.217803493406	-1.070291884052
C	3.224421644834	-3.422155714955	-0.888074215619
H	3.908721073610	-4.407405468441	0.913708479526
H	2.474773202968	-2.186345879020	-2.498122387281
H	3.384337190260	-4.297082278553	-1.524694355153
C	-0.888438429271	-0.114097383387	-0.922281636301
C	-0.412774369167	1.189711894734	-1.106956637008
C	-0.718006260895	-1.061281951390	-1.938826886937
C	0.231174026734	1.542810248747	-2.291152542566
H	-0.532988766434	1.926768189735	-0.309417111668
C	-0.078809284916	-0.705279926735	-3.126379370412
H	-1.067597220117	-2.089975726062	-1.813781254740

C	0.399896594883	0.594064413862	-3.301543524305
H	0.610551630400	2.560313970585	-2.418128957920
H	0.055697376239	-1.451335179525	-3.914392276118
H	0.909270274039	0.867717372104	-4.229891442704
C	-3.489093666319	-0.151959089369	0.367110929399
C	-4.166000900783	-0.661082029868	-0.748597629957
C	-4.183442441302	0.610755759122	1.311858535766
C	-5.527615368561	-0.412854719054	-0.913051859951
H	-3.631300144853	-1.247329047851	-1.503379416400
C	-5.546385135379	0.860019945209	1.144387117032
H	-3.644649503575	1.011139156639	2.175246427436
C	-6.218252555635	0.347568474044	0.034093110662
H	-6.052126227354	-0.810717302900	-1.786215073729
H	-6.085378713735	1.458926861250	1.883957376336
H	-7.286293836695	0.543628421354	-0.097452944010
O	-1.188417903839	0.367444205571	1.798286448422

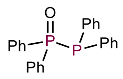

Free Energy = –1682.942006017792

P	-0.065971305342	0.739578406169	-0.583032036133
P	0.130844457441	-0.800161731993	1.015678591587
O	-0.096053858936	0.251903666024	-2.012437240387
C	1.680498404892	-1.649563216039	0.506228723573
C	2.412834091109	-2.261106510512	1.535250834264
C	2.178553079751	-1.703946504392	-0.806150258631
C	3.607980731881	-2.926434228521	1.261020063637
H	2.046013656203	-2.215525951426	2.565887965553
C	3.379038671275	-2.360823227469	-1.075496181527
H	1.624184836350	-1.228048049783	-1.619830445148
C	4.093661007120	-2.975371511522	-0.045826762227
H	4.164437979264	-3.401650521162	2.073814649957
H	3.757804826566	-2.393339817050	-2.101072187760
C	-1.239750579542	-1.853062470878	0.382492543859
C	-2.509225250752	-1.634888744654	0.938650611380
C	-1.095775006822	-2.808641060767	-0.631197672109
C	-3.616668501003	-2.347379733905	0.479376772900
H	-2.636148233422	-0.893379095940	1.734466905916
C	-2.201581619366	-3.530394535704	-1.079734991599
H	-0.115501703984	-2.994371811966	-1.077659559517
C	-3.463837829297	-3.298507757165	-0.529961706009

H	-4.601402464816	-2.161495175960	0.917369190099
H	-2.076440906347	-4.276302614638	-1.869848074271
C	1.292421966883	1.908711562590	-0.277254714765
C	1.873017794322	2.514528116688	-1.398360375516
C	1.781013037885	2.201284602891	1.003050819697
C	2.919956538088	3.421301783864	-1.238207135934
C	2.828152934874	3.108910827766	1.158618617436
H	1.357677194343	1.714368491561	1.887100696892
C	3.395573092582	3.720796143011	0.039470450871
H	3.206821543243	3.333716226589	2.159357786446
H	4.218520904470	4.430303862870	0.164048196784
C	-1.635497360997	1.534090758700	-0.120651892985
C	-1.805978933344	2.247209859785	1.072468906892
C	-2.722766321905	1.340737740142	-0.979298739359
C	-3.058504070290	2.762050241640	1.401860750058
C	-3.975276443112	1.856148789304	-0.645764518885
H	-2.579793513257	0.778990816014	-1.906253924417
C	-4.143794015468	2.564060513940	0.544576166731
H	-4.823770521914	1.701201923678	-1.317979203544
H	-5.126440579212	2.966086119484	0.807359658946
H	3.369606391463	3.893071141827	-2.116310277784
H	1.503346171525	2.264266236936	-2.396527401397
H	-0.964398098970	2.407395061497	1.752876686752
H	-3.188771950741	3.320343134471	2.332940458160
H	5.033682767037	-3.491014232887	-0.262418370679
H	-4.329711379463	-3.862989122038	-0.887745651167

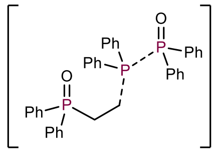

Free Energy = –2640.407027907527

O	4.266241130667	2.621489588677	-0.516712129448
C	1.183461445082	1.713218818827	-0.544931387784
C	2.221755340368	0.769005096017	-1.063809843832
H	0.956334588821	2.594182880859	-1.155555781545
H	1.139284310734	1.882971505402	0.539061225919
H	1.994790317796	-0.279397722521	-0.792427271358
H	2.292889736981	0.812817622012	-2.163979729667
P	3.872512296216	1.175839474748	-0.373070597430
C	3.742863654949	0.681835034415	1.379073288215

C	3.645246522620	1.702530698417	2.330756981814
C	3.698604911548	-0.655977589290	1.792263834833
C	3.496383424175	1.389521554020	3.682736317445
H	3.691435571707	2.744903580285	2.003150641925
C	3.558319492772	-0.966882181701	3.143724048729
H	3.784035070297	-1.467888252638	1.063271510837
C	3.454759620695	0.056068048349	4.089452670125
H	3.417529668855	2.191914622499	4.421552014465
H	3.529517988231	-2.013068800595	3.460691828356
H	3.340984457261	-0.189679550125	5.149108917659
C	5.058480243159	0.040173308366	-1.154981745045
C	4.733947635878	-1.262197066804	-1.558413457598
C	6.357565277202	0.522380272538	-1.359108572757
C	5.704072801718	-2.076276784973	-2.143009185431
H	3.720934147865	-1.654762716888	-1.430302536245
C	7.325590722060	-0.293612304410	-1.943140532351
H	6.601315492240	1.546696559803	-1.063276988253
C	7.000134163075	-1.593990212565	-2.332623688935
H	5.443610969497	-3.091037393856	-2.456065164574
H	8.338165479444	0.089088172204	-2.098705941996
H	7.758984176931	-2.233668465834	-2.792202185394
P	-2.701772313735	-0.900205845999	-1.306152937918
P	-1.049417190190	0.524755627640	-0.645364988452
C	-4.241286192933	-0.617104571567	-0.372762039230
C	-4.994947246575	0.507797562646	-0.743960252046
C	-4.683577892917	-1.415885481210	0.688544583844
C	-6.149127359423	0.845788811276	-0.040699460796
H	-4.673661466966	1.126398572558	-1.586317606174
C	-5.843081641100	-1.078771617292	1.386888901650
H	-4.132489183351	-2.312549185900	0.979694251472
C	-6.571283875560	0.056717787718	1.031369074182
H	-6.723902047714	1.728648355189	-0.333864049163
H	-6.178266814091	-1.710294093839	2.214248055429
H	-7.476081433535	0.322981749867	1.585089360640
C	-2.045370316533	-2.541416133555	-0.868618418012
C	-1.813600564347	-3.413694447935	-1.939654052551
C	-1.710787763038	-2.943758967154	0.433572558915
C	-1.264920760125	-4.675840447774	-1.713059225054
H	-2.065952836684	-3.090361403319	-2.953235426643
C	-1.160759286950	-4.205305788288	0.655388347540
H	-1.871277631853	-2.278877207728	1.285446129289

C	-0.937638315854	-5.072327608770	-0.416100707257
H	-1.090144462371	-5.351437986761	-2.555091092317
H	-0.903366399443	-4.510567078227	1.673347753154
H	-0.504821001007	-6.060938115259	-0.238199430952
C	-1.159399569121	0.303867858721	1.177907226291
C	-2.296044768645	0.617692660538	1.937978758202
C	-0.066529030396	-0.298657769247	1.819189378918
C	-2.342165824414	0.322672769461	3.299834212490
H	-3.161089268846	1.090874292857	1.466470994323
C	-0.112593360865	-0.596091721562	3.180209408362
H	0.832527819390	-0.556935529184	1.251425303475
C	-1.253208101580	-0.289213458797	3.922685320523
H	-3.238984724863	0.568685650268	3.875226361791
H	0.748468368942	-1.070224979737	3.658187968505
H	-1.292705067946	-0.524057025163	4.990124327578
C	-1.901490439051	2.112304621201	-1.008415676915
C	-2.001240686258	3.148046971547	-0.070206839985
C	-2.343559082009	2.341701558937	-2.321307754167
C	-2.563804876084	4.374510730360	-0.426697756262
H	-1.634773072911	3.008125100132	0.950297034644
C	-2.911686565708	3.565444498723	-2.671732891374
H	-2.270781616777	1.543056387832	-3.064554056738
C	-3.027184256692	4.585066154091	-1.725209611990
H	-2.637770640213	5.170865428646	0.319420492371
H	-3.263776371593	3.723297155927	-3.695237284803
H	-3.470827870981	5.545673604562	-2.002020741411
O	-2.903092581999	-0.744482159442	-2.795797584106

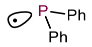

Free Energy = –803.858191061402

P	0.000219750812	-1.576892315810	0.000937092846
C	-1.434890806469	-0.465132681330	0.035207482839
C	-2.633085829047	-0.948210027151	-0.527306670180
C	-1.457352554571	0.793440651545	0.668726787752
C	-3.802444756031	-0.191443815085	-0.483790961519
H	-2.646282879323	-1.929826911281	-1.013188374465
C	-2.628504007494	1.545804565183	0.716324707306
H	-0.551005794484	1.184821187043	1.139532374077
C	-3.802982470190	1.059533430043	0.136182350143
H	-4.718959596814	-0.581053827562	-0.936031949846

H	-2.626212635102	2.519456832546	1.214843396701
H	-4.720302246089	1.653995955245	0.173212671419
C	1.434775701255	-0.464528169577	-0.033891085394
C	2.634325296654	-0.948559208304	0.525007896859
C	1.455902489303	0.795167059248	-0.665302592526
C	3.803721595719	-0.191938759034	0.479689427129
H	2.648573554161	-1.930986362142	1.009235179542
C	2.627042703134	1.547470472709	-0.714549678226
H	0.548752683350	1.187454388675	-1.133689196501
C	3.802944255057	1.060079407012	-0.138263187038
H	4.721251272879	-0.582623723610	0.928910624485
H	2.623641136065	2.521897507799	-1.211543335622
H	4.720320342357	1.654373370159	-0.176678175935

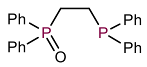

Free Energy = –1761.428138818596

C	0.706203383070	-1.502923659503	-0.838704585644
C	-0.772132722490	-1.793405684100	-0.558555224276
H	1.120849944477	-2.394624837747	-1.339793114315
H	0.803869246157	-0.664128982019	-1.548965556844
H	-1.295413252310	-1.985710972170	-1.511874463670
H	-0.881727169377	-2.711964503786	0.045880160991
P	1.767957296613	-1.225161558131	0.670232568672
P	-1.721987368474	-0.540302159982	0.373374787916
C	1.713360524300	0.614574611736	0.860868976861
C	1.377717756456	1.144898388035	2.110625000773
C	2.030804390541	1.495187464910	-0.183076010361
C	1.361237407556	2.526118337203	2.316374131768
H	1.112272344732	0.470269401674	2.928462600836
C	2.010234326591	2.873052488349	0.017040107232
H	2.308570137561	1.104544461377	-1.167786576135
C	1.677104999834	3.391472302922	1.270776467905
H	1.095561451355	2.926243310299	3.299368852278
H	2.254784732201	3.547043594804	-0.809062041323
H	1.659847930313	4.473899637235	1.429013894931
C	3.461453667699	-1.383521469854	-0.055375051975
C	4.522840978236	-1.473337472304	0.858811766398
C	3.760267901003	-1.403884908027	-1.424781712097
C	5.843528863577	-1.565020948676	0.421345786489
H	4.311844717316	-1.469998945008	1.934026418606

C	5.081465297340	-1.505898182005	-1.865927912347
H	2.964999967710	-1.334591105041	-2.171729381654
C	6.126617820064	-1.583621687243	-0.945727701710
H	6.655186802698	-1.630184222436	1.151883901416
H	5.293121082580	-1.520492892246	-2.939177241630
H	7.160928683495	-1.661967212079	-1.292890671590
C	-1.537152266037	1.040231903084	-0.505855807779
C	-1.561741409152	2.207206665976	0.264509215251
C	-1.389003207446	1.131205065079	-1.896182478021
C	-1.450401038149	3.453597361751	-0.348799816209
H	-1.649126324034	2.130293118267	1.351363215756
C	-1.274371996651	2.379193432999	-2.507278049033
H	-1.351743812403	0.231065733145	-2.517414787739
C	-1.308245083167	3.540982867720	-1.733764013491
H	-1.462523342693	4.361594156996	0.260338829943
H	-1.154582307092	2.443865661656	-3.592268934377
H	-1.215766508218	4.519322316614	-2.214049045318
C	-3.461997320764	-1.043472819358	0.155749266961
C	-4.092217665464	-1.088754009673	-1.094657523475
C	-4.177255781523	-1.399093081778	1.303892347245
C	-5.422692590717	-1.491589473528	-1.193795029334
H	-3.552407355526	-0.802393492275	-2.002878070559
C	-5.510077792922	-1.801128071051	1.203228988629
H	-3.679688692150	-1.354338102772	2.276670144850
C	-6.132270797012	-1.848672181138	-0.044374867931
H	-5.909668798098	-1.524532517126	-2.172344681132
H	-6.064726251541	-2.077463434457	2.104443997140
H	-7.176857107867	-2.163261194011	-0.123581602973
O	-1.361766851032	-0.450955444103	1.833644903139

(C) HOMO and LUMO of Pd complexes

Geometry optimizations and population analysis were performed at the with the M06/LanL08(f) for Pd and M06/6-31G(d,p) for other atoms with Grid = UltraFine in dichloromethane (CPCM model).

$PdCl_2$(**6gb**)

Electron Energy = –3675.975411

Pd	0.383460452436	-0.000983281476	1.309463436045
P	1.849102737113	-0.382427650618	-0.391882593011
P	-1.140667303342	0.362292862582	-0.330616095836
Cl	-1.309940388030	0.497917487847	2.962287443552
Cl	2.080861262008	-0.547134538139	2.914334560674

F	-5.751960834279	-4.373265193158	0.859293784377
F	-6.550211623238	-3.513136607981	-0.954690444383
C	2.061827758262	-2.140217840265	-0.723012671419
C	1.500306133322	-3.106879538361	0.120381021695
H	0.971751239043	-2.797522184812	1.017395240181
N	7.141246498626	2.393885387815	0.172479078587
C	5.869755712647	0.338389334194	-0.039168258482
H	6.769577247383	-0.261422737982	0.010002494592
C	2.747868091910	-2.582575777363	-1.864229371006
H	3.207594795587	-1.866107248655	-2.539878279454
F	-3.919510603156	6.379027476643	-1.541788007121
C	5.941317256189	1.747991135298	0.045580264792
N	2.408289473325	-6.241852612884	-1.592550766483
C	4.653080063029	-0.307714346017	-0.180221583487
H	4.640457787307	-1.391730495315	-0.234861601381
C	2.870251356732	-3.928404909985	-2.154244513685
H	3.412723902493	-4.219095946140	-3.044785216262
C	-2.642359461155	-0.651716849667	-0.170968651148
C	1.608533213554	-4.458126257094	-0.158780001786
H	1.156153314305	-5.165109681511	0.524827074371
C	2.297975529571	-4.910193930428	-1.308162505448
F	-1.865954760106	6.743422595319	-2.103206051839
C	-1.642771217438	2.099659490664	-0.533986384823
C	-0.389542422943	-0.102086902392	-1.955005652624
H	-0.485239086981	-1.186326628869	-2.068602124356
H	-0.931723799421	0.370431684752	-2.777290247231
C	1.080079773172	0.306087892500	-1.927303758482
H	1.184038510593	1.395261768757	-1.895132991288
H	1.615653013179	-0.054379968205	-2.809261464510
C	-4.850853748399	-2.310257783983	0.147102753711
C	-5.012358068408	-0.929057439194	0.183321580381
H	-5.993863135089	-0.497259939947	0.340459521563
C	-2.479151617969	2.475812447822	-1.593037080837
H	-2.879415843639	1.732079780279	-2.275279117286
C	3.456350888754	0.411379791517	-0.249204272768
C	-3.907297632003	-0.098719133906	0.027986596136
H	-4.040789967488	0.976331869286	0.069873396547
F	-7.045751607349	-2.639089307188	0.958687254958
C	-2.485076128455	-2.042866646871	-0.189228363703
H	-1.500526898484	-2.488261407720	-0.304878131717
C	-2.316822000708	4.772264637704	-0.897319812505

F	-2.475024746664	6.971561995487	-0.041075823838
C	4.722263873166	2.467168819991	-0.002957367271
H	4.716886872943	3.546679839872	0.077506158506
C	-1.150158107644	3.070559952370	0.339144328209
H	-0.513677216704	2.780842058881	1.168213855460
C	-2.818649170328	3.808719344066	-1.772215111958
H	-3.473051478445	4.096589298777	-2.587162846453
C	1.820841917183	-7.225551603508	-0.702208013702
H	2.250511555257	-7.168327850277	0.305411116467
H	2.013554133264	-8.223532617303	-1.093583337564
H	0.734467763539	-7.098960445999	-0.620361013333
C	3.515798074277	1.807863065700	-0.143367239016
H	2.602070914001	2.397474478511	-0.156319858001
C	-3.587551919253	-2.870751628480	-0.036966806863
H	-3.462662055776	-3.947350548546	-0.049332044398
C	-1.484817351152	4.408718938427	0.155561842973
H	-1.101478037544	5.160038106801	0.835645409869
C	-6.052256268110	-3.209793767477	0.259062931857
C	8.364645850473	1.627013631034	0.312605144246
H	8.350757784775	0.989648425553	1.206302882659
H	9.208306531351	2.310798383940	0.397585786384
H	8.538600293814	0.988769393181	-0.561465170151
C	-2.647190166329	6.221402444614	-1.139580058618
C	7.175279685260	3.833856805106	0.345979308247
H	6.722927202836	4.350150019169	-0.508596393261
H	8.211627753096	4.161018233723	0.420322724140
H	6.651204329136	4.151733317584	1.256729171459
C	3.130837097003	-6.677676005281	-2.772825048090
H	2.678447386353	-6.285907129947	-3.691981887960
H	3.107519936805	-7.765267379350	-2.825596026153
H	4.181352670630	-6.363070531043	-2.745135816814

$PdCl_2$(dppe)

Electron Energy = –2734.343470

Pd	0.000044000000	-0.000238000000	1.174307000000
P	1.550022000000	-0.014490000000	-0.501534000000
P	-1.550041000000	0.014243000000	-0.501442000000
Cl	-1.785663000000	0.084097000000	2.798883000000
Cl	1.786045000000	-0.084785000000	2.798555000000
C	2.290449000000	-1.649443000000	-0.775348000000
C	1.996323000000	-2.700039000000	0.098067000000

H	1.362071000000	-2.517487000000	0.964843000000
C	5.180849000000	1.849773000000	-0.073549000000
H	6.226106000000	1.569060000000	0.026752000000
C	3.110194000000	-1.878113000000	-1.888276000000
H	3.351112000000	-1.065752000000	-2.573077000000
C	4.811348000000	3.190741000000	-0.047812000000
C	4.213738000000	0.860349000000	-0.223217000000
H	4.506471000000	-0.187527000000	-0.234473000000
C	3.632700000000	-3.144902000000	-2.116635000000
H	4.271553000000	-3.318094000000	-2.978715000000
C	-2.871104000000	-1.217254000000	-0.356547000000
C	2.518687000000	-3.968039000000	-0.138877000000
H	2.286887000000	-4.782632000000	0.542209000000
C	3.335384000000	-4.189165000000	-1.242964000000
C	-2.290005000000	1.649434000000	-0.775170000000
C	-0.661556000000	-0.373769000000	-2.078852000000
H	-0.499314000000	-1.459448000000	-2.108793000000
H	-1.286554000000	-0.108622000000	-2.939426000000
C	0.661428000000	0.373218000000	-2.078978000000
H	0.499187000000	1.458886000000	-2.109225000000
H	1.286404000000	0.107797000000	-2.939484000000
C	-4.812601000000	-3.189772000000	-0.048157000000
C	-5.181652000000	-1.848699000000	-0.074630000000
H	-6.226875000000	-1.567606000000	0.024951000000
C	-3.108867000000	1.878738000000	-1.888617000000
H	-3.349310000000	1.066761000000	-2.574039000000
C	2.870673000000	1.217452000000	-0.356580000000
C	-4.214128000000	-0.859654000000	-0.224124000000
H	-4.506512000000	0.188312000000	-0.235979000000
C	-2.500422000000	-2.566626000000	-0.309474000000
H	-1.449790000000	-2.851360000000	-0.373526000000
C	-3.334466000000	4.189462000000	-1.242261000000
C	3.469714000000	3.549299000000	-0.163486000000
H	3.178340000000	4.595926000000	-0.132342000000
C	-1.996542000000	2.699561000000	0.099041000000
H	-1.362977000000	2.516526000000	0.966215000000
C	-3.631128000000	3.145675000000	-2.116723000000
H	-4.269278000000	3.319355000000	-2.979226000000
C	2.499537000000	2.566727000000	-0.310236000000
H	1.448868000000	2.851135000000	-0.375010000000
C	-3.471013000000	-3.548819000000	-0.162916000000

H	-3.180008000000	-4.595531000000	-0.131195000000
C	-2.518668000000	3.967707000000	-0.137637000000
H	-2.287377000000	4.781924000000	0.544071000000
H	-3.742260000000	5.180513000000	-1.425236000000
H	-5.571324000000	-3.959288000000	0.070098000000
H	3.743370000000	-5.180101000000	-1.426137000000
H	5.569754000000	3.960548000000	0.070581000000

(D) References

1. Maeda, S., Ohno, K., & Morokuma, K. Systematic exploration of the mechanism of chemical reactions: The global reaction route mapping (GRRM) strategy using the ADDF and AFIR methods. *Phys. Chem. Chem. Phys.* **15**, 3683−3701 (2013).
2. Frisch, M. J. *et al. Gaussian 16, Revision C.01*, Gaussian, Inc., Wallingford CT (2016).
3. Maeda, S. *et al.* Implementation and performance of the artificial force induced reaction method in the GRRM17 program. *J. Comput. Chem.* **39**, 233–251 (2018).
4. Maeda, S. & Harabuchi, Y. Exploring paths of chemical transformations in molecular and periodic systems: An approach utilizing force. *WIREs Comput. Mol. Sci.* **11**, e1538 (2021).
5. Choi, C. & Elber, R. Reaction path study of helix formation in tetrapeptides: Effect of side chains. *J. Chem. Phys*. 94, 751–760 (1991).
6. Ayala, P. Y. & Schlegel, H. B. A combined method for determining reaction paths, minima, and transition state geometries. *J. Chem. Phys.* **107**, 375–384 (1997).
